# Total Synthesis of Brevianamide S

**DOI:** 10.1021/acs.orglett.5c00860

**Published:** 2025-03-28

**Authors:** Adam R. Lockyer, Helen E. Jones, Nicholas J. Green, Robert C. Godfrey, Vera P. Demertzidou, Gary S. Nichol, Andrew L. Lawrence

**Affiliations:** EaStCHEM School of Chemistry, University of Edinburgh, Joseph Black Building, David Brewster Road, Edinburgh EH9 3FJ, U.K.

## Abstract

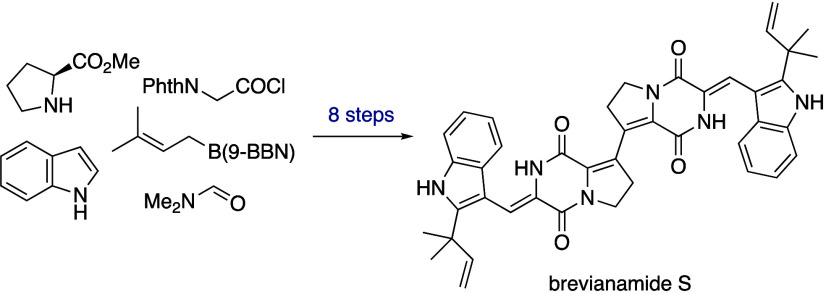

The first total synthesis of the alkaloid brevianamide S has been achieved in eight steps. This natural product, isolated from *Aspergillus versicolor*, exhibits selective antibacterial activity against Bacille Calmette-Guérin (BCG), a commonly used surrogate for *Mycobacterium tuberculosis*. Brevianamide S is proposed to act through a novel, yet-to-be-elucidated mechanism, making it a promising lead in the development of next-generation antitubercular agents. Our approach employs a bidirectional synthetic strategy, involving a bespoke alkenyl–alkenyl Stille cross-coupling reaction and a double aldol condensation. This represents a flexible and efficient platform for the future synthesis of structurally diverse analogues.

Tryptophan-derived diketopiperazine alkaloid dimers constitute a vast and diverse family of bioactive natural products.^1^ The biogenesis of these dimers is typically a result of the rich redox chemistry of the constituent tryptophan units, as reflected in their tryptophan-tryptophan linkages (Scheme 1a). Brevianamide S (**1**) is a dimeric diketopiperazine alkaloid produced by the fungus *Aspergillus versicolor* (MF030),^2^ which stands out due to its distinctive proline–proline linkage (Scheme 1b).^3^ It was discovered during a screen of marine-derived microbes against Bacille Calmette-Guérin (BCG), a weakened strain of the bovine tuberculosis bacillus *Mycobacterium bovis*.^2^ Brevianamide S (**1**) was found to selectively inhibit the growth of BCG (MIC 6.25 μg/mL), which is a valuable screening surrogate for *Mycobacterium tuberculosis*,^4^ without significant activity against other Gram-positive or Gram-negative bacteria tested (Scheme 1c).^2^ This specificity suggests that brevianamide S (**1**) may act through a novel mechanism of action, potentially making it a promising lead compound for the development of antitubercular drugs.^5^

**Scheme 1:**
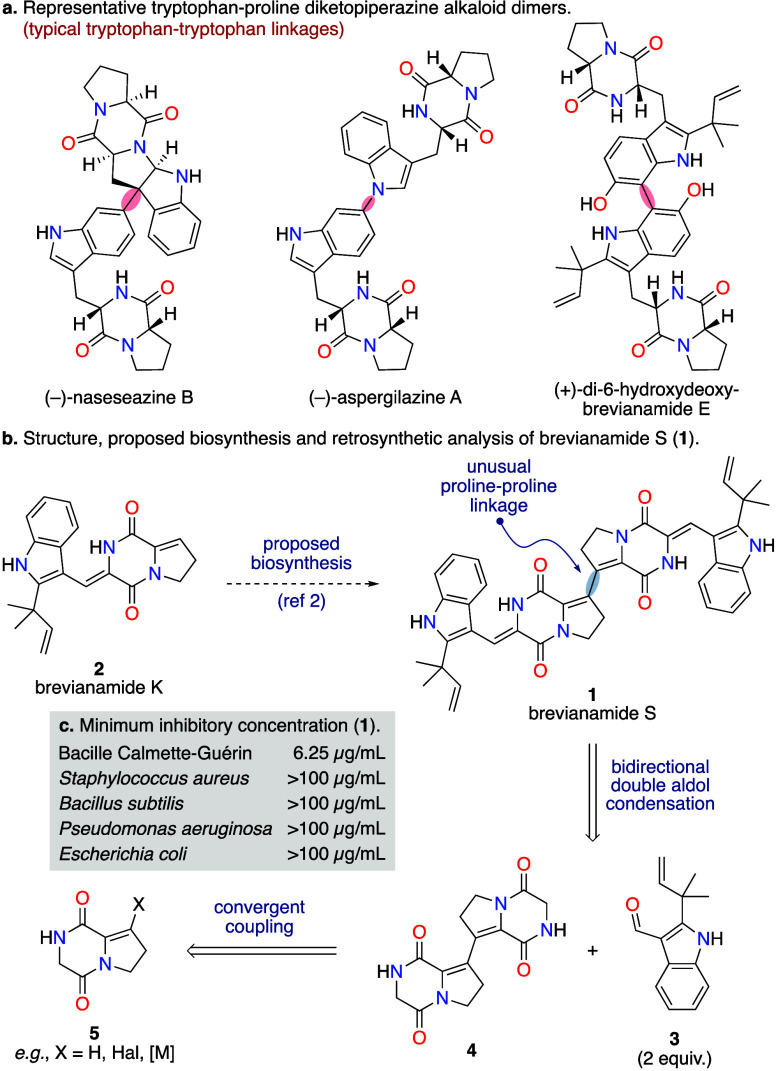
Brevianamide S (**1**) and Related Alkaloids

Brevianamide S (**1**) is proposed to form via oxidative homodimerization of brevianamide K (**2**),^2^ a coisolated natural product which has been isolated from various *Aspergillus* fungi (Scheme 1b).^3^ While a late-stage oxidative coupling strategy might represent an efficient route to brevianamide S (**1**), we opted to explore an ambitious three-component coupling strategy (Scheme 1b),^6^ which would lay the groundwork for a general synthetic strategy for accessing diverse structural analogues. Retrosynthetic analysis revealed that a double aldol transform of brevianamide S (**1**) generates known aldehyde **3** and a bis-diketopiperazine **4** (Scheme 1b).^7^ Although aware of the challenges associated with performing aldol condensations on similar diketopiperazines,^7,8^ we deemed the potential gain in step economy, and the future potential for exploring structural diversity, worth the challenge. A central disconnection of bis-diketopiperazine **4** gives a monomeric unsaturated diketopiperazine **5**. Although no direct literature precedent exists for this specific alkenyl–alkenyl coupling, many synthetic variants of the generalized building block **5** are conceivable. We ventured that successful implementation of this unique C–C coupling and the daunting double aldol condensation would enable a highly concise synthesis of brevianamide S (**1**) (Scheme 1b).

The synthesis began with a one-pot oxidation/*N*-acylation of proline methyl ester **6** (Scheme 2).^9^ The initial dehydrogenation to access dehydroproline **7** was achieved using *N*-chlorosuccinimide and triethylamine, which was followed by addition of phthalylglycyl chloride and 2,6-lutidine to give enamide **8** in 66% yield on multigram scale, following recrystallization. It should be noted that the choice of base for the imine acylation process is important, with stronger bases, such as triethylamine, resulting in an unwanted (2 + 2)-Staudinger synthesis of β-lactam **9** (Scheme 2a).^10^ Ammonia in methanol results in clean deprotection of the phthaloyl-protected amine **8**, with spontaneous cyclization giving diketopiperazine **10** in near quantitative yields, following Soxhlet extraction of phthalamide and subsequent recrystallization.^11^ Preliminary attempts at direct oxidative homocoupling of diketopiperazine **10** failed to give any promising results. However, when diketopiperazine **10** was exposed to iodine and pyridine, alkenyl iodide **11** could be obtained in 77% yield on multigram scale, with purification achieved by simple trituration (Scheme 2). Thus, we have developed an efficient, scalable, and chromatography-free three-step synthesis of alkenyl iodide **11**, a key intermediate in this synthesis that is also likely to serve as a broadly useful synthetic building block.

**Scheme 2:**
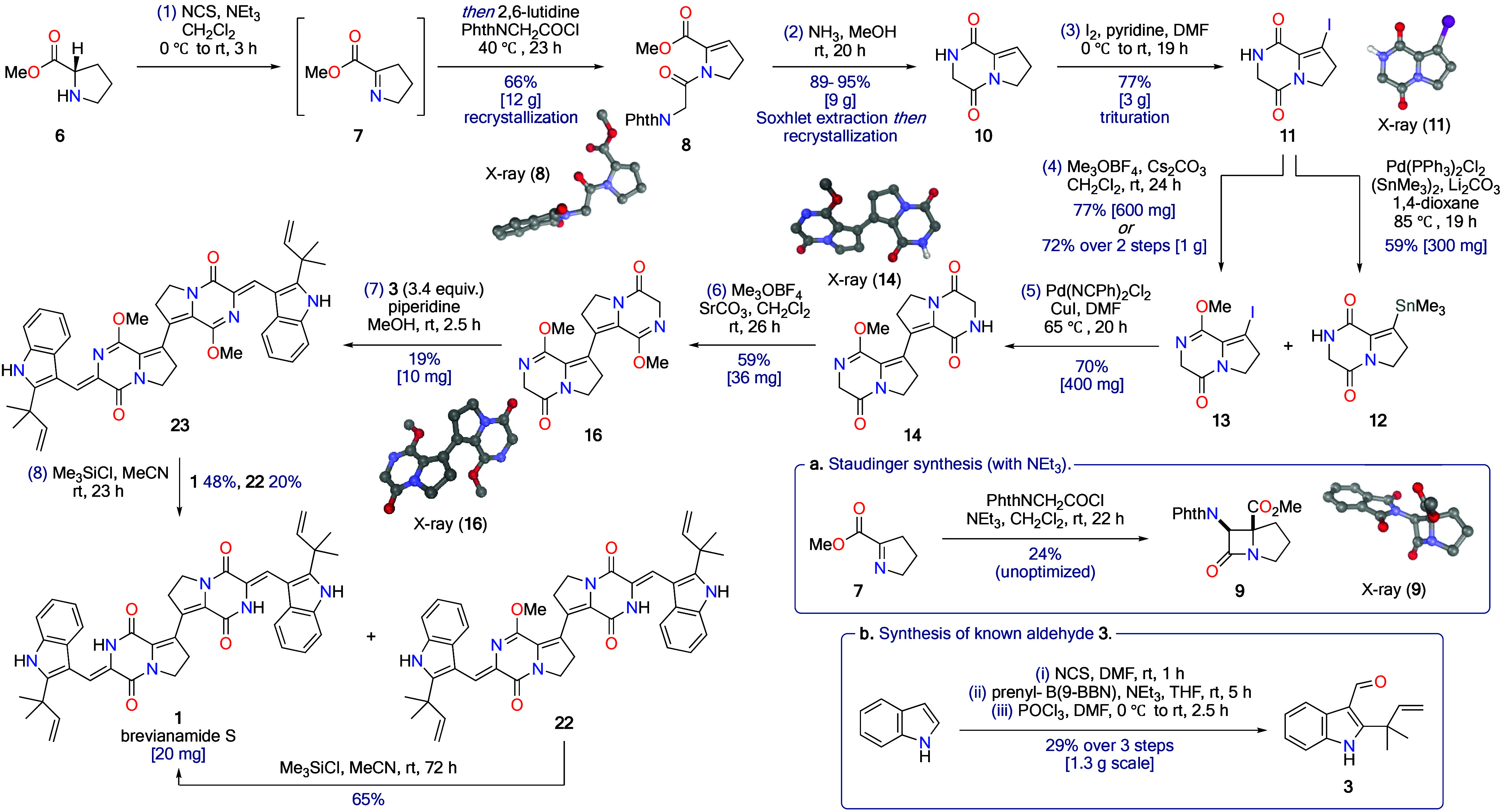
Total Synthesis of Brevianamide S (**1**)^*a*^ Reaction scales: The quantity of product made in the largest scale reactions conducted is shown in square brackets.

Attempts at Ullmann homocoupling of iodide **11** and Heck-type cross-coupling with the parent diketopiperazine **10** were unsuccessful. Many other tactics were also investigated, including numerous cross-coupling and homocoupling reactions of various intermediates, with frustratingly little success (see Tables S1–4 in the Supporting Information (SI) for full details). Organotin **12** could be accessed in 59% yield on 300 mg scale by coupling iodide **11** with hexamethylditin under standard Pd-catalyzed conditions (Scheme 2). Attempts at the cross-coupling of iodide **11** and organotin **12** gave tantalizing evidence that the key C–C bond formation was occurring (Scheme 3a). However, this Stille reaction was frustrated by the bis-diketopiperazine **4** being highly insoluble and difficult to purify. Therefore, the secondary lactam in iodo-diketopiperazine **11** was first converted into a methyl lactim ether **13** (Scheme 2).^12^ The hope was this would not only give more soluble, easy-to-handle compounds but also activate the neighboring methylene position for the challenging aldol chemistry later in the synthesis,^13^*vide infra*. A Stille cross-coupling of iodide **13** and organotin **12** was achieved using bis(benzonitrile)palladium dichloride as the precatalyst and copper(I) iodide as an additive to give over 400 mg of bis-diketopiperazine **14** in a single batch, in 70% yield (Scheme 2).^14^ The organotin methyl lactim ether **15** is also a competent coupling partner for a Stille cross-coupling reaction with iodide **13**, but the desired product **16** is not easily separated from a minor dimeric organotin product **17** (Scheme 3b). It is worth noting that many other attempts to access organometallic intermediates of methyl lactim ether **13**, *via* metalation or lithium-halogen exchange, were unproductive due to facile formation of pyrazinone **18**, which we attribute to the relatively acidic methylene position in iodide **13** (Scheme 3c) (see Table S7 in the SI for full details).

**Scheme 3:**
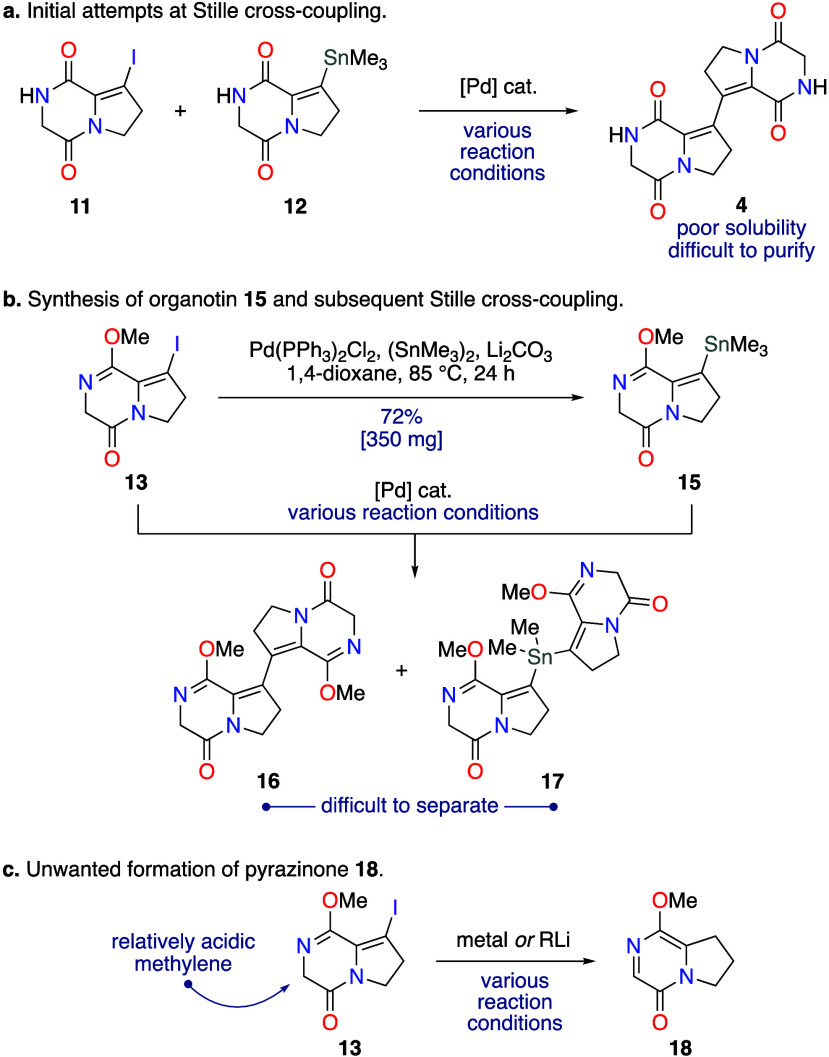
Attempted Functionalization and Coupling Reactions of Alkenyl Iodides **11** and **13**

With practical quantities of monomethylated bis-diketopiperazine **14** in-hand, the three-component double aldol condensation could be investigated (Scheme 4). The known aldehyde **3** was prepared on gram scale following literature procedures (Scheme 2b).^7^ Aldol condensations between diketopiperazines and aldehydes have been successfully exploited in several natural product syntheses,^15^ but they are often hampered by low yields. A particularly pertinent example can be found in the total synthesis of neochinulin B (**19**) by Kishi and co-workers,^8^ where a final aldol condensation between aldehyde **3** and unsaturated diketopiperazine **20** was used (Scheme 4a). This aldol condensation was also employed by Trauner and co-workers to prepare **19** as an intermediate in their synthesis of variecolortide B.^7^ The low yields of neochinulin B (**19**) achieved by both the Kishi and Trauner groups (25% and 36% yield, respectively) are typical of such aldol condensations,^15^ even when the diketopiperazine **20** is used in excess (Scheme 4a). Moreover, the tactic of using excess diketopiperazine to maximize yield is not amenable to a double aldol condensation, as it would likely result in single aldol condensation. Preliminary attempts at the double aldol condensation between bis-diketopiperazine **14** and excess aldehyde **3** in piperidine under reflux (i.e., the conditions used by Kishi and Trauner) led to very limited product formation, with extensive decomposition observed (Scheme 4b). Following a screen of reaction conditions, it was found that conducting the reaction at ambient temperature in methanol resulted in a selective single aldol condensation occurring exclusively adjacent to the lactim-ether, giving monoadduct **21** in 64% isolated yield (Scheme 4b). Even when the aldol condensation was left for several days and sodium sulfate was added as a dehydrating agent the double-aldol adduct **22** was only isolated in 4% yield, with the monoadduct **21** still representing the major product (Scheme 4b). Therefore, to enable a double aldol condensation, the remaining secondary lactam of bis-diketopiperazine **14** was first methylated to give double methyl lactim ether **16** in 59% yield (Scheme 2) (see Table S5 in the SI for full details). A double aldol condensation between bis-diketopiperazine **16** and aldehyde **3** was then achieved using piperidine in methanol at ambient temperature to give dimethyl-brevianamide S (**23**) in 19% yield. Although a seemingly modest yield, this equates to a 44% yield per-aldol condensation.^7,8^ Final deprotection of the secondary lactams using trimethylsilyl chloride in acetonitrile proceeded in 48% yield,^16^ thus completing the first total synthesis of brevianamide S (**1**) in 8 steps (longest linear sequence).^11^ The deprotection did not go to full conversion, with the monodeprotected material **22** isolated in 20% yield. Resubjecting this material to the deprotection conditions allowed for more brevianamide S (**1**) to be accessed, in 65% yield (Scheme 2). The spectroscopic data for our synthetic brevianamide S (**1**) matched perfectly with that reported by Capon and co-workers,^2^ thus confirming that the total synthesis had been achieved (see Table S6 in the SI for full details).

**Scheme 4:**
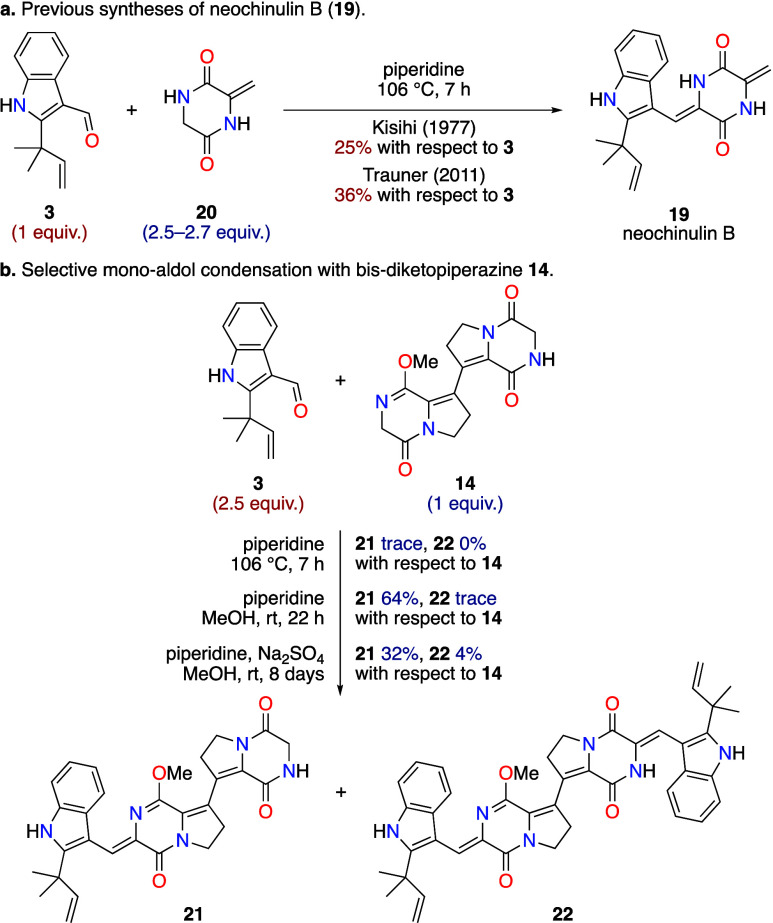
Attempted Aldol Condensations of Diketopiperazine **14**

In summary, the first total synthesis of a proline–proline linked diketopiperazine-alkaloid dimer has been achieved following a bidirectional three-component coupling strategy. To achieve this synthesis, a bespoke alkenyl–alkenyl Stille cross-coupling reaction and a double aldol condensation were developed. This represents a flexible synthetic strategy, which can now be leveraged to access diverse structural analogues. For example, the selective single aldol condensation of bis-diketopiperazine **14** (Scheme 4b), although not important for this target-orientated synthesis, will be of great utility for the future synthesis of nonsymmetrical analogues of brevianamide S (**1**).

## Data Availability

The data underlying this study are available in the published article, in its Supporting Information, and are openly available in ACS Chemistry Databank at DOI:10.5061/dryad.0rxwdbsbz.

## Supporting Information Available

Experimental procedures, optimization of reaction conditions, details of failed experiments, and NMR spectra for all new compounds. (PDF)
