# Supplementary material for: Total Synthesis of Brevianamide S
Source: Org Lett. 2025 Mar 28;27(14):3715–9. doi: 10.1021/acs.orglett.5c00860 (PMC11998070; doi:10.1021/acs.orglett.5c00860)
Supplement: Supplementary file 1 — ol5c00860_si_001.pdf [file ol5c00860_si_001.pdf]

Supplementary Information

Total Synthesis of Brevianamide S

Adam R. Lockyer, Helen E. Jones, Nicholas J. Green, Robert C. Godfrey, Vera P. Demertzidou, Gary S. Nichol, Andrew L. Lawrence*

EaStCHEM School of Chemistry, University of Edinburgh, Joseph Black Building, David Brewster Road, Edinburgh, EH9 3FJ, UK

Contents

1. Experimental 7

1.1. General experimental 7

1.2. Experimental Procedures for the Total Synthesis of Brevianamide S (1) 9

1.2.1. Experimental Procedure for phthalylglycyl chloride 9

1.2.2. Experimental Procedure for Enamide 8 10

1.2.3. Experimental Procedure for Diketopiperazine 10 12

1.2.4. Experimental Procedure for alkenyl iodide 11 13

1.2.5. Experimental Procedure for Lactim Ether 13 15

1.2.6. Telescoped Experimental Procedure for Lactim Ether 13 16

1.2.7. Experimental Procedure for Hexamethylditin 17

1.2.8. Experimental Procedure for Organotin 12 18

1.2.6. Experimental Procedure for Bis-Diketopiperazine 14 20

1.2.7. Experimental Procedure for Bis-Lactim Ether 16 23

1.2.8. Experimental Procedure for 3-chloroindole 25

1.2.9. Experimental Procedure for 3-Methyl-1,2-butadiene 26

1.2.10. Experimental Procedure for prenyl-9-BBN 27

1.2.11. Experimental Procedure for Indole S-1 28

1.2.12. Experimental Procedure for Aldehyde 3 30

1.2.13. Experimental Procedure for Enone 23 32

1.2.14. Experimental Procedure for Enone 22 34

1.2.15. Experimental Procedure for Brevianamide S (1) 36

1.2.16. Additional Experimental Procedure for Brevianamide S (1) 39

1.3. Other Experimental Procedures 40

1.3.1. Experimental Procedure for Dehydroproline 7 40

1.3.2. Experimental Procedure for β-lactam (±)-9 42

1.3.3. Experimental Procedure for Iodide S-2 44

1.3.4. Experimental Procedure for β-lactam (±)-S-3 45

1.3.5. Experimental Procedure for Hemiaminal S-4 47

1.3.6. Experimental Procedure for Lactim Ether S-5 49

1.3.7. Experimental Procedure for Pyrazinone 18 51

1.3.8. Experimental Procedure for Compound S-7 52

1.3.9. Experimental Procedure for Organotin S-9 53

1.3.10. Experimental Procedure for Organotin 15 55

1.3.11. Experimental Procedure for Mono-Adduct 21 57

2. NMR Spectra 59

2.1. ^{1}H NMR Spectrum for phthalylglycyl chloride (600 MHz, $CDCl_3$) 59

2.2. ^{13}C NMR Spectrum for phthalylglycyl chloride (126 MHz, $CDCl_3$) 59

2.3. ^{13}C DEPT-135 Spectrum for phthalylglycyl chloride ($CDCl_3$) 60

2.4. ^{1}H-^{1}H COSY Spectrum for phthalylglycyl chloride ($CDCl_3$) 60
2.5. ^{1}H-^{13}C HSQC Spectrum for phthalylglycyl chloride ($CDCl_3$) 61
2.6. ^{1}H-^{13}C HMBC Spectrum for phthalylglycyl chloride ($CDCl_3$) 61
2.7. ^{1}H NMR Spectrum for Enamide 8 (500 MHz, $CDCl_3$) 62
2.8. ^{13}C NMR Spectrum for Enamide 8 (126 MHz, $CDCl_3$) 62
2.9. ^{1}H-^{1}H COSY Spectrum for Enamide 8 ($CDCl_3$) 63
2.10. ^{1}H-^{13}C HSQC Spectrum for Enamide 8 ($CDCl_3$) 63
2.11. ^{1}H-^{13}C HMBC Spectrum for Enamide 8 ($CDCl_3$) 64
2.12. ^{1}H NMR Spectrum for Enamide 8 (500 MHz, $(CD_3)_2SO$) 64
2.13. ^{13}C NMR Spectrum for Enamide 8 (126 MHz, $(CD_3)_2SO$) 65
2.14. ^{1}H-^{1}H COSY Spectrum for Enamide 8 ($(CD_3)_2SO$) 65
2.15. ^{1}H-^{13}C HSQC Spectrum for Enamide 8 ($(CD_3)_2SO$) 66
2.16. ^{1}H-^{13}C HMBC Spectrum for Enamide 8 ($(CD_3)_2SO$) 66
2.17. ^{1}H NMR Spectrum for Enamide 8 (500 MHz, $(CD_3)_2SO$, 343 K) 67
2.18. ^{1}H-^{13}C HSQC Spectrum for Enamide 8 ($(CD_3)_2SO$, 343 K) 67
2.19. ^{1}H-^{13}C HMBC Spectrum for Enamide 8 ($(CD_3)_2SO$, 343 K) 68
2.20. ^{1}H NMR Spectrum for Diketopiperazine 10 (600 MHz, $CDCl_3$) 68
2.21. ^{13}C NMR Spectrum for Diketopiperazine 10 (151 MHz, $CDCl_3$) 69
2.22. ^{13}C DEPT-135 Spectrum for Diketopiperazine 10 ($CDCl_3$) 69
2.23. ^{1}H-^{1}H COSY Spectrum for Diketopiperazine 10 ($CDCl_3$) 70
2.24. ^{1}H-^{13}C HSQC Spectrum for Diketopiperazine 10 ($CDCl_3$) 70
2.25. ^{1}H-^{13}C HMBC Spectrum for Diketopiperazine 10 ($CDCl_3$) 71
2.26. ^{1}H NMR Spectrum for Alkenyl Iodide 11 (600 MHz, $CDCl_3$) 71
2.27. ^{13}C NMR Spectrum for Alkenyl Iodide 11 (126 MHz, $CDCl_3$) 72
2.28. ^{1}H-^{1}H COSY Spectrum for Alkenyl Iodide 11 ($CDCl_3$) 72
2.29. ^{1}H-^{13}C HSQC Spectrum for Alkenyl Iodide 11 ($CDCl_3$) 73
2.30. ^{1}H-^{13}C HMBC Spectrum for Alkenyl Iodide 11 ($CDCl_3$) 73
2.31. ^{1}H NMR Spectrum for Lactim Ether 13 (600 MHz, $CDCl_3$) 74
2.32. ^{13}C NMR Spectrum for Lactim Ether 13 (151 MHz, $CDCl_3$) 74
2.33. ^{13}C DEPT-135 Spectrum for Lactim Ether 13 ($CDCl_3$) 75
2.34. ^{13}C DEPT-Q135 Spectrum for Lactim Ether 13 ($CDCl_3$) 75
2.35. ^{1}H-^{1}H COSY Spectrum for Lactim Ether 13 ($CDCl_3$) 76
2.36. ^{1}H-^{13}C HSQC Spectrum for Lactim Ether 13 ($CDCl_3$) 76
2.37. ^{1}H-^{13}C HMBC Spectrum for Lactim Ether 13 ($CDCl_3$) 77
2.38. ^{1}H NMR Spectrum for hexamethylditin (600 MHz, $CDCl_3$) 77
2.39. ^{13}C NMR Spectrum for hexamethylditin (151 MHz, $CDCl_3$) 78
2.40. ^{119}Sn NMR Spectrum for hexamethylditin (149 MHz, $CDCl_3$) 78
2.41. ^{1}H NMR Spectrum for Organotin 12 (500 MHz, $CDCl_3$) 79
2.42. ^{13}C NMR Spectrum for Organotin 12 (126 MHz, $CDCl_3$) 79
2.43. ^{13}C DEPT-135 Spectrum for Organotin 12 ($CDCl_3$) 80

2.44. ^{13}C DEPT-Q135 Spectrum for Organotin 12 ($CDCl_3$) 80
2.45. ^{1}H-^{1}H COSY Spectrum for Organotin 12 ($CDCl_3$)....................81
2.46. ^{1}H-^{13}C HSQC Spectrum for Organotin 12 ($CDCl_3$)....................81
2.47. ^{1}H-^{13}C HMBC Spectrum for Organotin 12 ($CDCl_3$) 82
2.48. ^{119}Sn NMR Spectrum for Organotin 12 (149 MHz, $CDCl_3$) 82
2.49. ^{1}H NMR Spectrum for Bis-Diketopiperazine 14 (600 MHz, $CDCl_3$)....................83
2.50. ^{13}C NMR Spectrum for Bis-Diketopiperazine 14 (126 MHz, $CDCl_3$) 83
2.51. ^{1}H-^{1}H COSY Spectrum for Bis-Diketopiperazine 14 ($CDCl_3$)....................84
2.52. ^{1}H-^{13}C HSQC Spectrum for Bis-Diketopiperazine 14 ($CDCl_3$)....................84
2.53. ^{1}H-^{13}C HMBC Spectrum for Bis-Diketopiperazine 14 ($CDCl_3$)....................85
2.54. ^{1}H NMR Spectrum for Bis-Lactim Ether 16 (500 MHz, $CDCl_3$) 85
2.55. ^{13}C NMR Spectrum for Bis-Lactim Ether 16 (126 MHz, $CDCl_3$) 86
2.56. ^{1}H-^{1}H COSY Spectrum for Bis-Lactim Ether 16 ($CDCl_3$) 86
2.57. ^{1}H-^{13}C HSQC Spectrum for Bis-Lactim Ether 16 ($CDCl_3$) 87
2.58. ^{1}H-^{13}C HMBC Spectrum for Bis-Lactim Ether 16 ($CDCl_3$) 87
2.59. ^{1}H NMR Spectrum for 3-chloroindole (600 MHz, $CDCl_3$) 88
2.60. ^{13}C NMR Spectrum for 3-chloroindole (151 MHz, $CDCl_3$) 88
2.61. ^{1}H NMR Spectrum for 3-methyl-1,2-butadiene (500 MHz, $CDCl_3$) 89
2.62. ^{13}C NMR Spectrum for 3-methyl-1,2-butadiene (126 MHz, $CDCl_3$) 89
2.63. ^{1}H NMR Spectrum for Indole S-1 (500 MHz, $CDCl_3$)....................90
2.64. ^{13}C NMR Spectrum for Indole S-1 (126 MHz, $CDCl_3$)....................90
2.65. ^{1}H NMR Spectrum for Aldehyde 3 (600 MHz, $CDCl_3$) 91
2.66. ^{13}C NMR Spectrum for Aldehyde 3 (151 MHz, $CDCl_3$) 91
2.67. ^{1}H NMR Spectrum for Enone 23 (500 MHz, $CDCl_3$) 92
2.68. ^{13}C NMR Spectrum for Enone 23 (126 MHz, $CDCl_3$) 92
2.69. ^{1}H-^{1}H COSY Spectrum for Enone 23 ($CDCl_3$)....................93
2.70. ^{1}H-^{13}C HSQC Spectrum for Enone 23 ($CDCl_3$)....................93
2.71. ^{1}H-^{13}C HMBC Spectrum for Enone 23 ($CDCl_3$) 94
2.72. ^{1}H NMR Spectrum for Enone 22 (500 MHz, $CDCl_3$) 94
2.73. ^{13}C NMR Spectrum for Enone 22 (126 MHz, $CDCl_3$) 95
2.74. ^{1}H-^{1}H COSY Spectrum for Enone 22 ($CDCl_3$)....................95
2.75. ^{1}H-^{13}C HSQC Spectrum for Enone 22 ($CDCl_3$)....................96
2.76. ^{1}H-^{13}C HMBC Spectrum for Enone 22 ($CDCl_3$) 96
2.77. ^{1}H-^{13}C HMBC Spectrum for Enone 22 ($CDCl_3$, modified for 5 Hz coupling)....................97
2.78. ^{1}H-^{13}C HMBC Spectrum for Enone 22 ($CDCl_3$, modified for 2 Hz coupling)....................97
2.79. ^{1}H-^{1}H NOESY Spectrum for Enone 22 ($CDCl_3$)....................98
2.80. ^{1}H-^{15}N HMBC Spectrum for Enone 22 ($CDCl_3$) 98
2.81. ^{1}H NMR Spectrum for Brevianamide S (1) (500 MHz, $CDCl_3$) 99
2.82. ^{13}C NMR Spectrum for Brevianamide S (1) (126 MHz, $CDCl_3$) 99
2.83. ^{1}H-^{1}H COSY Spectrum for Brevianamide S (1) ($CDCl_3$)....................100

2.84. ^{1}H-^{13}C HSQC Spectrum for Brevianamide S (1) ($CDCl_3$) 100
2.85. ^{1}H-^{13}C HMBC Spectrum for Brevianamide S (1) ($CDCl_3$) 101
2.86. ^{1}H-^{1}H NOESY Spectrum for Brevianamide S (1) ($CDCl_3$) 101
2.87. ^{1}H NMR Spectrum for Brevianamide S (1) (500 MHz, CD_3OD) 102
2.88. ^{13}C NMR Spectrum for Brevianamide S (1) (126 MHz, CD_3OD) 102
2.89. ^{1}H-^{1}H COSY Spectrum for Brevianamide S (1) (CD_3OD) 103
2.90. ^{1}H-^{13}C HSQC Spectrum for Brevianamide S (1) (CD_3OD) 103
2.91. ^{1}H-^{13}C HMBC Spectrum for Brevianamide S (1) (CD_3OD) 104
2.92. ^{1}H NMR Spectrum for Dehydroproline 7 (500 MHz, $CDCl_3$) 104
2.93. ^{13}C NMR Spectrum for Dehydroproline 7 (126 MHz, $CDCl_3$) 105
2.94. ^{13}C DEPT-135 Spectrum for Dehydroproline 7 ($CDCl_3$) 105
2.95. 1H-1H COSY Spectrum for Dehydroproline 7 (CDCl3) 106
2.96. ^{1}H-^{13}C HSQC Spectrum for Dehydroproline 7 ($CDCl_3$) 106
2.97. ^{1}H-^{13}C HMBC Spectrum for Dehydroproline 7 ($CDCl_3$) 107
2.98. ^{1}H NMR Spectrum for β-Lactam (±)-9 (500 MHz, $CDCl_3$) 107
2.99. ^{13}C NMR Spectrum for β-Lactam (±)-9 (126 MHz, $CDCl_3$) 108
2.100. ^{13}C DEPT-135 Spectrum for β-Lactam (±)-9 ($CDCl_3$) 108
2.101. ^{1}H-^{1}H COSY Spectrum for β-Lactam (±)-9 ($CDCl_3$) 109
2.102. ^{1}H-^{13}C HSQC Spectrum for β-Lactam (±)-9 ($CDCl_3$) 109
2.103. ^{1}H-^{13}C HMBC Spectrum for β-Lactam (±)-9 ($CDCl_3$) 110
2.104. ^{1}H-^{1}H NOESY Spectrum for β-Lactam (±)-9 ($CDCl_3$) 110
2.105. ^{1}H NMR Spectrum for Iodide S-2 (500 MHz, $CDCl_3$) 111
2.106. ^{13}C NMR Spectrum for Iodide S-2 (126 MHz, $CDCl_3$) 111
2.107. ^{13}C DEPT-135 Spectrum for Iodide S-2 ($CDCl_3$) 112
2.108. ^{1}H-^{1}H COSY Spectrum for Iodide S-2 ($CDCl_3$) 112
2.109. ^{1}H-^{13}C HSQC Spectrum for Iodide S-2 ($CDCl_3$) 113
2.110. ^{1}H-^{13}C HMBC Spectrum for Iodide S-2 ($CDCl_3$) 113
2.111. ^{1}H NMR Spectrum for β-Lactam (±)-S-3 (500 MHz, $CDCl_3$) 114
2.112. ^{13}C NMR Spectrum for β-Lactam (±)-S-3 (126 MHz, $CDCl_3$) 114
2.113. ^{13}C DEPT-135 Spectrum for β-Lactam (±)-S-3 ($CDCl_3$) 115
2.114. ^{1}H-^{1}H COSY Spectrum for β-Lactam (±)-S-3 ($CDCl_3$) 115
2.115. ^{1}H-^{13}C HSQC Spectrum for β-Lactam (±)-S-3 ($CDCl_3$) 116
2.116. ^{1}H-^{13}C HMBC Spectrum for β-Lactam (±)-S-3 ($CDCl_3$) 116
2.117. ^{1}H-^{1}H NOESY Spectrum for β-Lactam (±)-S-3 ($CDCl_3$) 117
2.118. ^{1}H NMR Spectrum for Hemiaminal S-4 (500 MHz, $CDCl_3$) 117
2.119. ^{13}C NMR Spectrum for Hemiaminal S-4 (126 MHz, $CDCl_3$) 118
2.120. ^{13}C DEPT-135 Spectrum for Hemiaminal S-4 ($CDCl_3$) 118
2.121. ^{1}H-^{1}H COSY Spectrum for Hemiaminal S-4 ($CDCl_3$) 119
2.122. ^{1}H-^{13}C HSQC Spectrum for Hemiaminal S-4 ($CDCl_3$) 119
2.123. ^{1}H-^{13}C HMBC Spectrum for Hemiaminal S-4 ($CDCl_3$) 120

2.124. ^{1}H NMR Spectrum for Lactim Ether S-5 (500 MHz, $CDCl_3$) 120
2.125. ^{13}C NMR Spectrum for Lactim Ether S-5 (126 MHz, $CDCl_3$) 121
2.126. ^{1}H-^{1}H COSY Spectrum for Lactim Ether S-5 ($CDCl_3$) 121
2.127. ^{1}H-^{13}C HSQC Spectrum for Lactim Ether S-5 ($CDCl_3$) 122
2.128. ^{1}H-^{13}C HMBC Spectrum for Lactim Ether S-5 ($CDCl_3$) 122
2.129. ^{1}H NMR Spectrum for impure Tetrafluoroborate S-6 (500 MHz, $CDCl_3$) 123
2.130. ^{11}B NMR Spectrum for impure Tetrafluoroborate S-6 (128 MHz, CDCl3) 123
2.131. ^{13}C NMR Spectrum for impure Tetrafluoroborate S-6 (126 MHz, $CDCl_3$) 124
2.132. ^{1}H-^{1}H COSY Spectrum for impure Tetrafluoroborate S-6 ($CDCl_3$) 124
2.133. ^{1}H-^{13}C HSQC Spectrum for impure Tetrafluoroborate S-6 ($CDCl_3$) 125
2.134. ^{1}H-^{13}C HMBC Spectrum for impure Tetrafluoroborate S-6 ($CDCl_3$) 125
2.135. ^{19}F NMR Spectrum for impure Tetrafluoroborate S-6 (376 MHz, $CDCl_3$) 126
2.136. ^{1}H NMR Spectrum for impure Pyrazinone 18 (500 MHz, $CDCl_3$) 126
2.137. ^{13}C NMR Spectrum for impure Pyrazinone 18 (126 MHz, $CDCl_3$) 127
2.138. ^{1}H-^{1}H COSY Spectrum for impure Pyrazinone 18 ($CDCl_3$) 127
2.139. ^{1}H-^{13}C HSQC Spectrum for impure Pyrazinone 18 ($CDCl_3$) 128
2.140. ^{1}H-^{13}C HMBC Spectrum for impure Pyrazinone 18 ($CDCl_3$) 128
2.141. ^{1}H NMR Spectrum for impure Compound S-7 (600 MHz, $CDCl_3$) 129
2.142. ^{13}C NMR Spectrum for impure Compound S-7 (126 MHz, $CDCl_3$) 129
2.143. ^{13}C DEPT-135 Spectrum for impure Compound S-7 ($CDCl_3$) 130
2.144. ^{1}H-^{1}H COSY Spectrum for impure Compound S-7 ($CDCl_3$) 130
2.145. ^{1}H-^{13}C HSQC Spectrum for impure Compound S-7 ($CDCl_3$) 131
2.146. ^{1}H-^{13}C HMBC Spectrum for impure Compound S-7 ($CDCl_3$) 131
2.147. ^{1}H NMR Spectrum for Organotin S-9 (600 MHz, $CDCl_3$) 132
2.148. ^{13}C NMR Spectrum for Organotin S-9 (151 MHz, $CDCl_3$) 132
2.149. ^{1}H-^{1}H COSY Spectrum for Organotin S-9 ($CDCl_3$) 133
2.150. ^{1}H-^{13}C HSQC Spectrum for Organotin S-9 ($CDCl_3$) 133
2.151. ^{1}H-^{13}C HMBC Spectrum for Organotin S-9 ($CDCl_3$) 134
2.152. ^{119}Sn NMR Spectrum for Organotin S-9 (149 MHz, $CDCl_3$) 134
2.153. ^{1}H NMR Spectrum for Organotin 15 (500 MHz, $CDCl_3$) 135
2.154. ^{13}C NMR Spectrum for Organotin 15 (126 MHz, $CDCl_3$) 135
2.155. ^{1}H-^{1}H COSY Spectrum for Organotin 15 ($CDCl_3$) 136
2.156. ^{1}H-^{13}C HSQC Spectrum for Organotin 15 ($CDCl_3$) 136
2.157. ^{1}H-^{13}C HMBC Spectrum for Organotin 15 ($CDCl_3$) 137
2.158. ^{119}Sn NMR Spectrum for Organotin 15 (149 MHz, $CDCl_3$) 137
2.159. ^{1}H NMR Spectrum for Mono-Adduct 21 (500 MHz, CD_3OD) 138
2.160. ^{13}C NMR Spectrum for Mono-Adduct 21 (126 MHz, CD_3OD) 138
2.161. ^{1}H-^{1}H COSY Spectrum for Mono-Adduct 21 (CD_3OD) 139
2.162. ^{1}H-^{13}C HSQC Spectrum for Mono-Adduct 21 (CD_3OD) 139
2.163. ^{1}H-^{13}C HMBC Spectrum for Mono-Adduct 21 (CD_3OD) 140

2.164. ^{1}H-^{1}H NOESY Spectrum for Mono-Adduct 21 (CD_3OD)....................140
3. IR Spectra....................**141**
3.1. IR Spectrum for phthalylglycyl chloride....................141
3.2. IR Spectrum for Enamide 8....................141
3.3. IR Spectrum for Diketopiperazine 10....................142
3.4. IR Spectrum for Alkenyl Iodide 11....................142
3.5. IR Spectrum for Lactim Ether 13....................143
3.6. IR Spectrum for Organotin 12....................143
3.7. IR Spectrum for Bis-Diketopiperazine 14....................144
3.8. IR Spectrum for Bis-Lactim Ether 16....................144
3.9. IR Spectrum for 3-chloroindole....................145
3.10. IR Spectrum for Indole S-1....................145
3.11. IR Spectrum for Aldehyde 3....................146
3.12. IR Spectrum for Enone 23....................146
3.13. IR Spectrum for Enone 22....................147
3.14. IR Spectrum for Brevianamide S (1)....................147
3.15. IR Spectrum for Dehydroproline 7....................148
3.16. IR Spectrum for β-lactam (±)-9....................148
3.17. IR Spectrum for Iodide S-2....................149
3.18. IR Spectrum for β-Lactam (±)-S-3....................149
3.19. IR Spectrum for Hemiaminal S-4....................150
3.20. IR Spectrum for Lactim Ether S-5....................150
3.21. IR Spectrum for Pyrazinone 18....................151
3.22. IR Spectrum for Organotin S-9....................151
3.23. IR Spectrum for Organotin 15....................152
3.24. IR Spectrum for Mono-Adduct 21....................152
4. X-ray Crystal Structures....................**153**
4.1. X-ray Crystal Structure Data of Enamide 8: CCDC number 2426509....................153
4.2. X-ray Crystal Structure Data of Alkenyl Iodide 11: CCDC number 2426508....................164
4.3. X-ray Crystal Structure Data of Organotin 12: CCDC number 2426510....................173
4.4. X-ray Crystal Structure Data of Bis-Diketopiperazine 14: CCDC number 2426513....................187
4.5. X-ray Crystal Structure Data of Bis-Lactim Ether 16: CCDC number 2426512....................197
4.6. X-ray Crystal Structure Data of β-Lactam 9: CCDC number 2426514....................207
4.7. X-ray Crystal Structure Data of β-Lactam S-3: CCDC number 2426515....................217
4.8. X-ray Crystal Structure Data of Hemiaminal S-4: CCDC number 2426516....................227
4.9. X-ray Crystal Structure Data of Tetrafluoroborate S-6: CCDC number 2426511....................237
5. References....................**247**

1. Experimental

1.1. General experimental

NMR Spectroscopy: ^{1}H NMR spectra were recorded at 600 MHz, 500 MHz, and 400 MHz using a Bruker AVANCE 600, Bruker AVANCE 500, Bruker PRO 500, or Bruker AVANCE 400 spectrometer and referenced to residual solvent proton resonance ($CHCl_3$ δ 7.26 ppm, CD_3SOCHD_2 δ 2.50 ppm, CHD_2OD δ 3.31 ppm). Chemical shifts are given in ppm on a δ scale, and coupling constants (*J*) are reported to nearest 0.1 Hz. Peak multiplicities are defined as: s = singlet, d = doublet, t = triplet, q = quartet, qu = quintet, hpt = heptet, m = multiplet, app. = apparent, br. = broad. Assignment of proton signals was assisted by ^{1}H-^{1}H COSY, ^{1}H-^{13}C HSQC, ^{1}H-^{13}C HMBC and ^{1}H-^{1}H NOESY experiments. ^{11}B NMR spectra were recorded at 128 MHz using a Bruker AVANCE 400 spectrometer and referenced to an external standard (15% $BF_3{\cdot}Et_2O$ in $CDCl_3$ δ 0 ppm). ^{13}C NMR spectra were recorded at 151 MHz, 126 MHz or 101 MHz using a Bruker AVANCE 600, Bruker AVANCE 500, Bruker PRO 500 or Bruker AVANCE 400 spectrometers respectively and referenced to the solvent resonance ($CDCl_3$ δ 77.0 ppm, $(CD_3)_2SO$ δ 39.5 ppm, CD_3OD 49.0 ppm). Assignment of ^{13}C signals was assisted by ^{1}H-^{13}C HSQC and ^{1}H-^{13}C HMBC experiments. ^{15}N signals were measured indirectly for ^{1}H-^{15}N HSQC and ^{1}H-^{15}N HSQC experiments using a Bruker AVANCE 800 recorded at 800 MHz (^{1}H) and 81 MHz (^{15}N). ^{19}F NMR spectra were recorded at 376 MHz using a Bruker AVANCE 400 spectrometer and referenced to an external standard (neat $CFCl_3$ δ 0 ppm). ^{119}Sn NMR spectra were recorded at 149 MHz or 186 MHz using a Bruker AVANCE 400 or Bruker PRO 500 spectrometer and referenced to an external standard (neat $SnCl_4$ δ −150 ppm).

Mass Spectrometry: Accurate mass (HRMS) data was acquired on a Bruker MicroTOF instrument using electrospray ionisation (ESI+). High resolution values are calculated to 4 decimal places from the molecular formula, and all values are within a tolerance of 5 ppm.

Infrared Spectroscopy: Infrared spectra of solids and liquids were recorded as neat samples on a Bruker Tensor 27 FT-IR spectrometer fitted with an ATR attachment.

X-ray Crystallography: Single crystal X-ray diffraction data were measured on a Rigaku Oxford Diffraction SuperNova (**16**, **S-3**, **S-6**), Rigaku Oxford Diffraction Xcalibur (**9**, **S-4**), Bruker D8 Venture (**11**, **12**) or at beamline I-19, EH1 of Diamond Light Source (**8**, **14**). Wavelengths used: Mo Kα (λ = 0.71013 Å) **9**, **11**, **12**, **S-3**, **S-4**; Cu Kα (λ = 1.54184 Å) **16**, **S-6**; synchrotron Zr(III) edge (λ = 0.6889 Å)

8, **14**. In all cases a crystal temperature of 100 K (**8**, **11**, **12**, **14**) or 120 K (**9**, **11**, **S-3**, **S-4**, **S-6**) was maintained during data collection with an Oxford Cryosystems Cryostream 700+ (**8**, **9**, **14**, **16**, **S-3**, **S-4**, **S-6**) or 800 (**11**, **12**).

Melting points: Melting points were measured using a Bibby Scientific SMP10 melting point apparatus.

Reagents, solvents and techniques: All reagents, obtained from Acros, Alfa Aesar, Aldrich and Fluorochem fine chemicals suppliers, were used directly as supplied unless stated otherwise. NIS and NCS were used as supplied without recrystallization. The following anhydrous solvents were obtained from the University of Edinburgh School of Chemistry's communal solvent purification system, which were purified by filtration through activated alumina columns: acetonitrile (MeCN), dichloromethane (CH_2Cl_2) and tetrahydrofuran (THF). These solvents were transferred, stored, and used under a positive pressure of nitrogen in Young's valve-sealed, oven-dried and nitrogen-purged Strauss flasks. The following anhydrous solvents were purchased and used as received from Acros Organics: 1,4-dioxane [CAS: 123-91-1], *N*,*N* dimethylformamide (DMF) [CAS: 68-12-2], *N*-methyl-2-pyrrolidone (NMP) [872-50-4] and methanol (MeOH) [CAS: 67-56-1]. Piperidine was obtained from Aldrich and is described as "≥99.5% purified by redistillation". Anhydrous solvents were used for reactions but not workups or purifications. THF was distilled before use in column chromatography. Reactions were conducted at room temperature and stirred magnetically unless stated otherwise. The yields reported are shown as isolated yields unless otherwise stated. Reactions were monitored by thin layer chromatography on pre-coated aluminium-backed plates (Merck Kieselgel 60 with fluorescent indicator UV254, 0.2 mm). Spots were visualised by quenching of UV fluorescence (λ_{max} = 254 nm) or by staining with, $Ce(SO_4)_2$, $KMnO_4$, ninhydrin, *p*-anisaldehyde, PMA or vanillin. Yields greater than 95% are reported as >95%.

1.2. Experimental Procedures for the Total Synthesis of Brevianamide S (1)

1.2.1. Experimental Procedure for phthalylglycyl chloride

Phthalylglycyl chloride is a commercially available reagent but was prepared on large scale according to a literature procedure.[1]

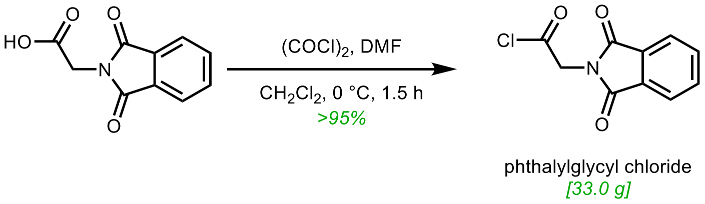

DMF (0.10 mL, 0.9 mol%., 1.30 mmol) was added to a solution of *N*-phthaloylglycine (30.6 g, 1.0 equiv., 149 mmol) in CH_2Cl_2 (300 mL) under N_2. The reaction was then cooled to 0 °C before $(COCl)_2$ (25.0 mL, 2.0 equiv., 291 mmol) was added dropwise. After 1.5 h of stirring at 0 °C the reaction was concentrated under reduced pressure to afford phthalylglycyl chloride (33.0 g, 148 mmol, >95%) as a colourless solid.

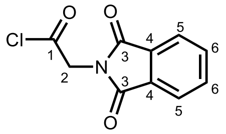

Data consistent with literature.[1]

MP 86–90 °C (CH_2Cl_2);

^{1}H NMR (600 MHz, $CDCl_3$): δ 7.92 (2H, dd, J = 5.5, 3.0 Hz, H-5), 7.79 (2H, dd, J = 5.5, 3.0 Hz, H-6), 4.82 (2H, s, H-2) ppm;

^{13}C NMR (126 MHz, $CDCl_3$): δ 169.2 (C-1), 166.7 (C-3), 134.8 (C-6), 131.7 (C-4), 124.1 (C-5), 47.7 (C-2) ppm;

IR (film) ν_{max}/cm^{-1} 1798, 1776, 1720, 1403, 1382, 713.

1.2.2. Experimental Procedure for Enamide 8

Compound **8** was prepared from a combination of two modified literature procedures.[2,3]

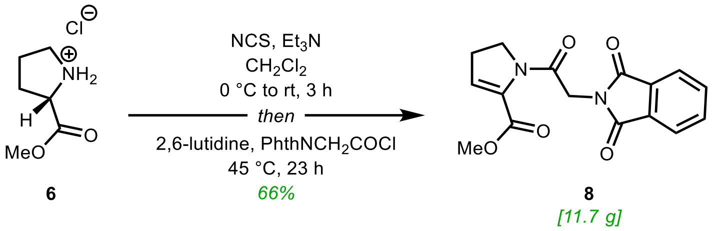

To a solution of L-proline methyl ester hydrochloride **6** (9.30 g, 1.0 equiv., 56.2 mmol) and Et_3N (15.5 mL, 2.0 equiv., 111 mmol) in CH_2Cl_2 (170 mL) at 0 °C under N_2 was added NCS (8.21 g, 1.1 equiv., 61.5 mmol) in ten equal portions over 1 h. The mixture was stirred at room temperature for 3 h before 2,6-lutidine (13.0 mL, 2.0 equiv., 113 mmol) was added dropwise. A solution of phthalylglycyl chloride (16.3 g, 1.3 equiv., 72.9 mmol) in CH_2Cl_2 (45 mL) was added dropwise to the reaction mixture. The reaction was then heated to 45 °C for 23 h before it was quenched with aq. (1 M) HCl (2 × 300 mL) then washed with saturated aq. $NaHCO_3$ (300 mL) and brine (300 mL), dried over Na_2SO_4 and concentrated under reduced pressure. The crude residue was purified by recrystallization from boiling MeOH (1 L) to give enamide **8** (11.7 g, 37.2 mmol, 66%) as a beige solid.

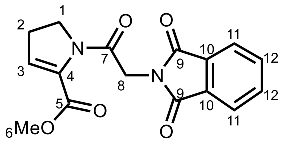

$\mathbf{R_f}$ = 0.50 (EtOAc:CH_2Cl_2, 1:1), [UV, $KMnO_4$];

MP 184 °C (MeOH);

*At room temperature enamide **8** exhibits some rotameric properties. The rotation is not of a speed at which sperate rotamers can be observed but certain peaks in the ^1H and ^{13}C spectra are broadened with some not being observable at all. Increasing the temperature to 343 K reduced this broadening and several previously unobserved correlations in 2D spectra are able to observed. The spectra recorded at 343 K can be found in chapters 2.17.–2.19..

^{1}H NMR (500 MHz, $CDCl_3$): δ 7.83 (2H, dd, J = 5.5, 3.2 Hz, H-11), 7.69 (2H, dd, J = 5.5, 3.0 Hz, H-12), 6.08 (1H, app. br. s, H-3), 4.45 (2H, s, H-8), 4.11 (2H, dd, J = 9.0, 8.2 Hz, H-1), 3.78 (3H, s, H-6), 2.75 (2H, td, J = 8.5, 2.8 Hz, H-2) ppm;

^{1}**H NMR** (500 MHz, $(CD_3)_2SO$): δ 7.93 (2H, dd, *J* = 5.4, 3.2 Hz, H-11), 7.89 (2H, dd, *J* = 5.5, 3.1 Hz, H-12), 6.01 (1H, app. br. s, H-3), 4.53 (2H, s, H-8), 4.12 (2H, app. t, *J* = 8.5 Hz, H-1), 3.64 (3H, s, H-6), 2.75 (2H, td, *J* = 8.7, 3.0 Hz, H-2) ppm;

^{13}**C NMR** (126 MHz, $CDCl_3$): δ 167.8 (C-9), 163.8 (C-7), 162.0 (C-5), 135.9 (C-4), 134.1 (C-12), 132.2 (C-10), 123.6 (C-11), 52.5 (C-6), 48.5 (C-1), 40.1 (C-8), 28.9 (C-2) ppm;

*C-3 could not be assigned in $CDCl_3$ as the peak was too broad to be observed.

^{13}**C NMR** (126 MHz, $(CD_3)_2SO$): δ 167.4 (C-9), 163.2 (C-7), 162.0 (C-5), 135.1 (C-4), 134.7 (C-12), 131.5 (C-10), 123.3 (C-11), 122.9 (C-3) 52.0 (C-6), 46.8 (C-1), 28.9 (C-2) ppm;

HRMS (ESI^+) calc for $C_{16}H_{15}N_2O_5$ ($[M+H]^+$): 315.0976; found: 315.0969;

IR (film) ν_{max}/cm^{-1} 1714, 1677, 1423, 1392, 1319, 1195, 1165, 1111;

scXRD: Evaporation of a saturated solution (Et_2O). Data collected remotely at beamline I-19 of Diamond Light Source.[4]

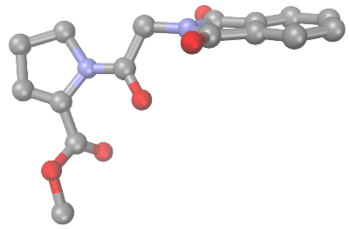

1.2.3. Experimental Procedure for Diketopiperazine 10

Diketopiperazine **10** was prepared according to a modified literature procedure.[5]

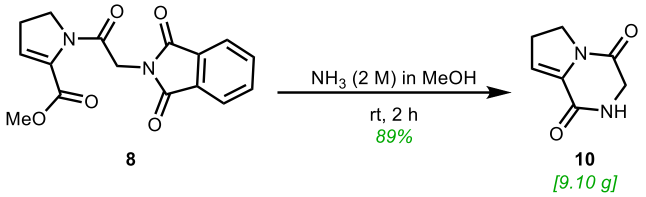

Enamide **8** (21.1 g, 67.0 mmol) was dissolved in a solution of NH_3 in MeOH (2 M, 560 mL). After 2 h of stirring, at room temperature under N_2, the resulting suspension was concentrated under reduced pressure. Stoichiometric phthalimide was removed from the crude residue by Soxhlet extraction with $CHCl_3$. The crude extract was then purified by recrystallization from hot MeOH (500 mL) to give diketopiperazine **10** (9.10 g, 59.8 mmol, 89%) as colourless needles.

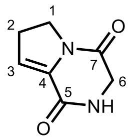

R$_f$ = 0.23 (CH_2Cl_2:MeOH, 10:1), [UV, $Ce(SO_4)_2$, $KMnO_4$, *p*-anisaldehyde, PMA, vanillin];

MP 206 °C (decomp.) (MeOH);

^{1}H NMR (600 MHz, $CDCl_3$): δ 6.44 (1H, br. s, N–H), 6.19 (1H, app. t, *J* = 2.9 Hz, H-3), 4.16 (2H, s, H-6), 4.05 (2H, app. t, *J* = 9.2 Hz, H-1), 2.80 (2H, td, *J* = 9.4, 2.8 Hz, H-2);

^{13}C NMR (151 MHz, $CDCl_3$): δ 159.7 (C-7), 157.6 (C-5), 132.8 (C-4), 119.7 (C-3), 46.9 (C-6), 45.6 (C-1), 27.9 (C-2);

HRMS (ESI$^+$) calc for $C_7H_8N_2O_2Na$ ($[M+Na]^+$): 175.0478; found: 175.0480;

IR (film) ν_{max}/cm^{-1} 3196, 1665, 1641, 1456, 1443, 1436, 1109, 1047, 762, 733.

1.2.4. Experimental Procedure for alkenyl iodide 11

Alkenyl iodide **11** was prepared according to a modified literature procedure.[6]

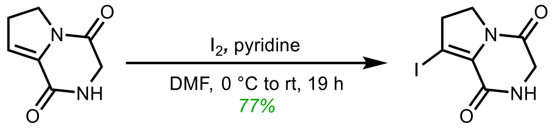

To a slurry of diketopiperazine **10** (2.07 g, 1.0 equiv., 13.6 mmol) in DMF (40 mL), at 0 °C under N_2 were added successively I_2 (13.7 g, 4.0 equiv., 54.0 mmol) and pyridine (2.20 mL, 2.0 equiv., 27.3 mmol). The reaction stirred at room temperature for 18 h before being diluted with CH_2Cl_2 (30 mL) and quenched with saturated aq. $NaHCO_3$ (30 mL). After thorough mixing, saturated aq. $Na_2S_2O_3$ (30 mL) was added. The aqueous layer was extracted with CH_2Cl_2 (8 × 100 mL). The combined organics were dried over Na_2SO_4 and concentrated under reduced pressure. The crude residue was suspended in Et_2O (500 mL) and stirred at room temperature for 18 h. The solid was collected by vacuum filtration to give alkenyl iodide **11** (2.92 g, 10.5 mmol, 77%) as a beige solid.

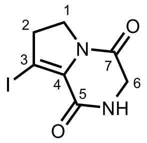

R_f = 0.30 (CH_2Cl_2:MeOH, 10:1), [UV, $KMnO_4$];

MP 197 °C (decomp.) (MeOH);

^{1}H NMR (600 MHz, $CDCl_3$): δ 6.05 (1H, app. s, N–H), 4.14 (2H, d, J = 1.9 Hz, H-6), 4.02 (2H, app. t, J = 9.3 Hz, H-1), 3.06 (2H, dd, J = 9.9, 9.1 Hz, H-2) ppm;

^{13}C NMR (126 MHz, $CDCl_3$): δ 158.9 (C-5 or C-7), 156.8 (C-5 or C-7), 130.4 (C-4), 81.5 (C-3), 47.0 (C-6), 45.8 (C-1), 40.4 (C-2) ppm;

HRMS (ESI$^+$) calc for $C_7H_8IN_2O_2$ ($[M+H]^+$): 278.9625; found: 278.9628;

IR (film) ν_{max}/cm^{-1} 1680, 1649, 1617, 1449, 1115, 833;

scXRD: Evaporation of a saturated solution (Et_2O).

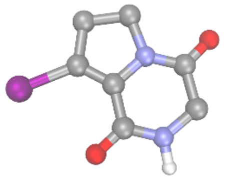

Table S1 – Halogenation conditions for diketopiperazine **10**.

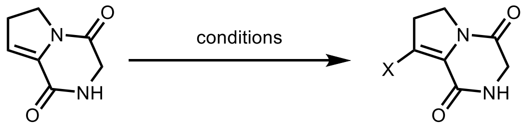

Entry	X	Halogen source	Conditions	Isolated yield /%
1	I	NIS	$HOOCCF_3$<br>CH_2Cl_2<br>rt, 2.5 h	22
2	I	DIH	$HOOCCF_3$<br>CH_2Cl_2<br>rt, 5 h	25
3	I	I_2	pyridine, DMF<br>0 °C to rt, 19 h	77
4	Br	Br_2	pyridine, DMF<br>0 °C to rt, 20 min	decomp.

1.2.5. Experimental Procedure for Lactim Ether 13

Lactim ether **13** was prepared according to a literature procedure.[7]

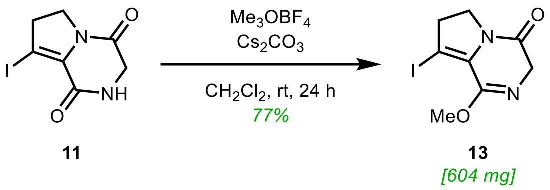

To a solution of alkenyl iodide **11** (742 mg, 1.0 equiv., 2.67 mmol) in CH_2Cl_2 (75 mL) under N_2 were added successively Cs_2CO_3 (2.21 g, 2.5 equiv., 6.79 mmol) and Me_3OBF_4 (2.06 g, 5.2 equiv., 13.9 mmol). After 24 h of stirring at room temperature, the reaction was quenched with ice water (100 mL). The aqueous layer was extracted with CH_2Cl_2 (3 × 100 mL). The combined organics were dried over Na_2SO_4 and concentrated under reduced pressure. The crude residue was purified by column chromatography (silica gel, EtOAc) to give lactim ether **13** (604 mg, 2.07 mmol, 77%) as a beige solid.

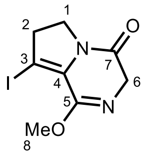

R_f = 0.56 (CH_2Cl_2:MeOH, 10:1), 0.26 (EtOAc), [UV, $KMnO_4$, *p*-anisaldehyde, vanillin];

MP 164 °C (decomp.) (EtOAc);

^{1}H NMR (600 MHz, $CDCl_3$): δ 4.30 (2H, app. t, *J* = 1.6 Hz, H-6), 3.92 (2H, dd, *J* = 10.0, 8.8 Hz, H-1), 3.79 (3H, s, H-8), 3.00 (2H, app. tt, *J* = 9.6, 1.6 Hz, H-2) ppm;

^{13}C NMR (151 MHz, $CDCl_3$): δ 163.5 (C-7), 151.9 (C-5), 130.3 (C-4), 72.2 (C-3), 53.1 (C-6), 53.0 (C-8), 44.8 (C-1), 40.7 (C-2) ppm;

HRMS (ESI^+) calc for $C_8H_9IN_2O_2$ ($[M+H]^+$): 292.9782; found: 292.9778;

IR (film) ν_{max}/cm^{-1} 1662, 1644, 1597, 1429, 1401, 1316, 1021, 747.

1.2.6. Telescoped Experimental Procedure for Lactim Ether 13

Lactim ether **13** was prepared from a combination of two modified literature procedures.[6–7]

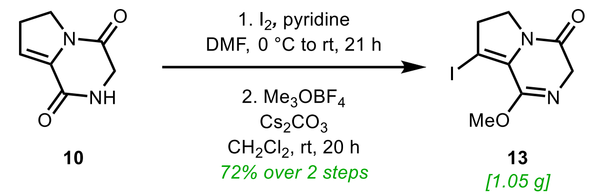

To a slurry of diketopiperazine **10** (759 mg, 1.0 equiv., 4.99 mmol) in DMF (15 mL), at 0 °C under N_2 were added successively I_2 (5.07 g, 4.0 equiv., 20.0 mmol) and pyridine (0.80 mL, 2.0 equiv., 9.91 mmol). The reaction stirred at room temperature for 21 h before being diluted with CH_2Cl_2 (30 mL) and quenched with a mixture of saturated aq. $NaHCO_3$ (15 mL) and saturated aq. $Na_2S_2O_3$ (15 mL). The aqueous layer was extracted with CH_2Cl_2 (4 × 80 mL). The combined organics were dried over Na_2SO_4 and concentrated under reduced pressure. The crude residue was suspended in CH_2Cl_2 (100 mL) under N_2. Cs_2CO_3 (4.38 g, 2.7 equiv., 13.4 mmol) and Me_3OBF_4 (4.15 g, 5.6 equiv., 28.1 mmol) were added successively to the suspension. After 20 h of stirring at room temperature, the reaction was quenched with water (100 mL). The aqueous layer was extracted with CH_2Cl_2 (3 × 100 mL). The combined organics were dried over Na_2SO_4 and concentrated under reduced pressure. The crude residue was purified by column chromatography (silica gel, EtOAc) to give lactim ether **13** (1.05 g, 3.59 mmol, 72%) as a beige solid.

See previous page for characterization of lactim ether **13**.

1.2.7. Experimental Procedure for Hexamethylditin

Hexamethylditin is a commercially available reagent but was prepared on large scale according to a literature procedure.[8]

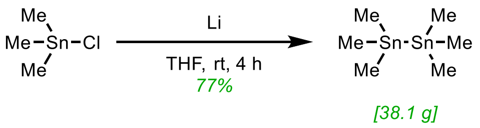

A solution of Me_3SnCl (1.0 M, 1.0 equiv., 300 mmol) in THF (300 mL) was added dropwise over 30 min to a suspension of lithium strips (approximately: 2.5 g, 1.2 equiv., 360 mmol) in THF (300 mL) under N_2. The mixture was then stirred at room temperature for 4 h. Vacuum distillation (80 mbar, 40 °C) removed the majority of the THF. The remaining residue was stirred in *n*-hexane for 14 h, then filtered to remove the solids. Further vacuum distillation (20 mbar, 67 °C), collecting at −78 °C, gave hexamethylditin (38.1 g, 116 mmol, 77%) as a cloudy liquid, partially solidified at room temperature.

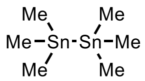

Data consistent with literature.[9]

BP 67 °C (20 mbar);

^{1}H NMR (600 MHz, $CDCl_3$): δ 0.20 ppm;

*Sn satellites observed.

^{13}C NMR (151 MHz, $CDCl_3$): δ −10.0 ppm;

*Sn satellites observed (^{13}C–^{119}Sn J = 102 Hz, ^{13}C–^{117}Sn J = 97 Hz, ^{13}C–Sn–^{119}Sn J = 23.5 Hz, ^{13}C–Sn–^{117}Sn J = 22.6 Hz).

^{119}Sn NMR (149 MHz, $CDCl_3$): δ −108.39 ppm.

*Sn satellites observed (^{119}Sn–^{117}Sn J = 2112 Hz).

1.2.8. Experimental Procedure for Organotin 12

Organotin **12** was prepared according to a modified literature procedure.[10]

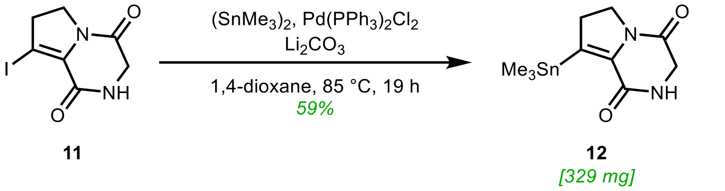

To a slurry of alkenyl iodide **11** (495 mg, 1.0 equiv., 1.78 mmol), Li_2CO_3 (338 mg, 2.6 equiv., 4.59 mmol), $(SnMe_3)_2$ (0.80 mL, 2.2 equiv., 3.86 mmol) in 1,4-dioxane (50 mL) under N_2 was added $Pd(PPh_3)_2Cl_2$ (63.6 mg, 5 mol%, 90.6 µmol). The reaction was stirred at 85 °C for 19 h before being passed through a small pad of celite and washed with EtOAc (500 mL), then concentrated under reduced pressure. The crude residue was purified by column chromatography (silica gel, CH_2Cl_2:MeOH, 30:1) to give organotin **12** (329 mg, 1.04 mmol, 59%) as colourless needles.

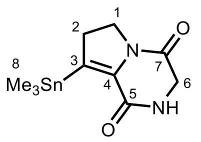

R_f = 0.29 (CH_2Cl_2:MeOH, 10:1), 0.21 (EtOAc), [UV, $KMnO_4$];

MP 156 °C (decomp.) (EtOAc);

^{1}H NMR (600 MHz, $CDCl_3$): δ 7.25 (1H, br. s, N–H), 4.14 (2H, s, H-6), 3.99 (2H, app. t, J = 9.0 Hz, H-1), 2.83 (2H, app. t, J = 8.9 Hz, H-2), 0.21 (9H, s, H-8) ppm;

*Sn satellites observed at H-8 (^{1}H–C–^{115}Sn J = 63.5 Hz, ^{1}H–C–^{119}Sn J = 29.0 Hz, ^{1}H–C–^{117}Sn J = 27.7 Hz).

^{13}C NMR (126 MHz, $CDCl_3$): δ 159.30 (C-5 or C-7), 159.25 (C-5 or C-7), 138.2 (C-3), 136.1 (C-4), 46.9 (C-6), 46.4 (C-1), 34.2 (C-2), −8.21 (C-8) ppm;

*Sn satellites observed at C-8 (^{13}C–^{119}Sn J = 193 Hz, ^{13}C–^{117}Sn J = 184 Hz).

^{119}Sn NMR (149 MHz, $CDCl_3$): δ −43.08 ppm;

HRMS (ESI$^+$) calc for $C_{10}H_{17}N_2O_2Sn$ ([M+H]$^+$): 317.0307; found: 317.0310;

IR (film) ν_{max}/cm^{-1} 1674, 1652, 1613, 1455, 1325, 766;

scXRD: Evaporation of a saturated solution (EtOAc).

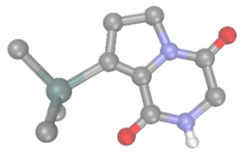

Table S2 – Metallation conditions for alkenyl iodide **11**. All yields in this table are isolated yields.

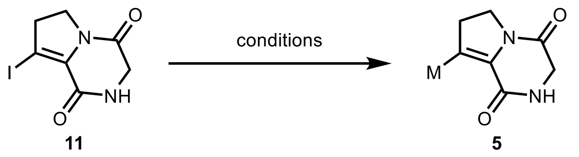

Entry	M	Metal source	Conditions	Result
1	Bpin	B_2pin_2	$Pd(dppf)Cl_2{\cdot}CH_2Cl_2$ KOAc, 1,4-dioxane 80 °C, 5 d	starting material
2	Bpin	B_2pin_2	$Pd(OAc)_2$, SPhos KOAc, 1,4-dioxane 80 °C, 2 d	starting material
3	$B(OH)_2$	$B_2(OH)_4$	$Ni(dppp)Cl_2$, PPh_3 DIPEA, EtOH rt to 50 °C, 2 d	starting material
4	$SiMe_3$	$(SiMe_3)_2$	$Pd(PPh_3)_2Cl_2$ K_2CO_3 DMF 80 °C, 22 h	starting material
5	$SnMe_3$	$(SnMe_3)_2$	$Pd(PPh_3)_2Cl_2$ Li_2CO_3 1,4-dioxane 85 °C, 19 h	59%
6	$SnMe_3$	$(SnMe_3)_2$	$Pd(OAc)_2$ SPhos, Li_2CO_3 1,4-dioxane 100 °C, 18 h	48%[1]
7	*n*-Bu_3Sn	(*n*-$Bu_3Sn)_2$	$Pd(OAc)_2$, CuI SPhos, Li_2CO_3 1,4-dioxane 100 °C, 17.5 h	28%

[1]While appearing pure based on NMR data, a minor impurity was observed by TLC.

1.2.6. Experimental Procedure for Bis-Diketopiperazine 14

Bis-diketopiperazine **14** was prepared according to a modified literature procedure.[10]

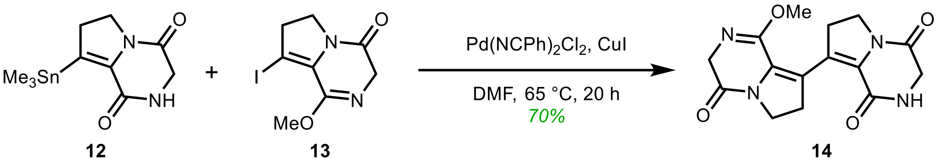

$Pd(NCPh)_2Cl_2$ (43.9 mg, 6 mol%, 114 µmol) and CuI (120 mg, 34 mol%, 631 µmol) were added sequentially to a solution of organotin **12** (763 mg, 1.3 equiv., 2.42 mmol) and alkenyl iodide **13** (547 mg, 1.0 equiv., 1.87 mmol) in DMF (30 mL) under N_2. The reaction was stirred at 65 °C for 20 h before being concentrated under reduced pressure. The crude residue was purified by column chromatography (silica gel, CH_2Cl_2:MeOH, 12:1) to give bis-diketopiperazine **14** (415 mg, 1.31 mmol, 70%) as a yellow solid.

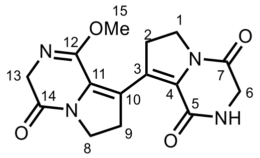

$\mathbf{R_f}$ = 0.08 ($CHCl_3$:MeOH, 10:1), [UV, $KMnO_4$, ninhydrin, *p*-anisaldehyde, PMA, vanillin];

MP 258 °C (decomp.) (MeOH);

^{1}H NMR (600 MHz, $CDCl_3$): δ 5.92 (1H, app. s, N–H), 4.38 (2H, t, J = 1.7 Hz, H-13), 4.18 (2H, d, J = 1.9 Hz, H-6), 4.01 (2H, app. t, J = 9.2 Hz, H-1), 3.97 (2H, app. t, J = 8.9 Hz, H-8), 3.74 (3H, s, H-15), 2.99 (2H, app. tt, J = 9.9 Hz, 1.6 Hz, H-9), 2.93 (2H, dd, J = 10.2, 8.5 Hz, H-2) ppm;

^{13}C NMR (126 MHz, $CDCl_3$): δ 164.4 (C-14), 159.9 (C-7), 157.2 (C-5), 152.9 (C-12), 127.8 (C-3), 126.9 (C-4), 125.8 (C-11), 118.6 (C-10), 53.3 (C-15), 53.0 (C-13), 46.7 (C-6), 44.0 (C-1), 43.4 (C-8), 32.6 (C-2), 31.6 (C-9) ppm;

HRMS (ESI$^+$) calc for $C_{15}H_{17}N_4O_4$ ([M+H]$^+$): 317.1244; found: 317.1245;

IR (film) ν_{max}/cm^{-1} 1667, 1433, 1319;

scXRD: Slow cooling of a boiling, saturated solution (MeOH). Data collected remotely at beamline I-19 of Diamond Light Source.[4]

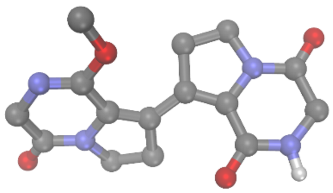

Table S3 – Coupling conditions towards bis-diketopiperazine **14**. All yields in this table are isolated yields.

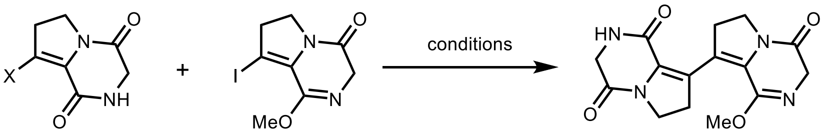

Entry	Reaction Name	X	Conditions	Result
1	Mizoroki–Heck	H	$Pd(PPh_3)_2Cl_2$<br>Ag_2CO_3, THF<br>50 °C, 26 h	starting material
2	Mizoroki–Heck	H	$Pd(NCPh)_2Cl_2$<br>Ag_2CO_3, THF<br>50 °C, 27 h	starting material
3	Mizoroki–Heck	H	$Pd(PPh_3)_2Cl_2$<br>Cs_2CO_3, *n*-Bu_4NOAc<br>DMF, 70 °C, 23 h	**10**: unreacted<br>**13**: decomp.
4	Mizoroki–Heck	H	$Pd(NCPh)_2Cl_2$<br>Cs_2CO_3, *n*-Bu_4NOAc<br>DMF, 70 °C, 23 h	**10**: unreacted<br>**13**: decomp.
5	Mizoroki–Heck	H	$Pd(OAc)_2$<br>BippyPhos, HTMP<br>DMF, 70 °C, 17 h	**10**: unreacted<br>**13**: decomp.
6	Migita–Kosugi–Stille	$SnMe_3$	$Pd(NCPh)_2Cl_2$<br>CuI, DMF<br>65 °C, 20 h	70%

Table S4 – Coupling conditions towards bis-lactim ether **16**. Ratios were obtained through NMR analysis of crude material. All yields in this table were acquired using durene as an internal standard.

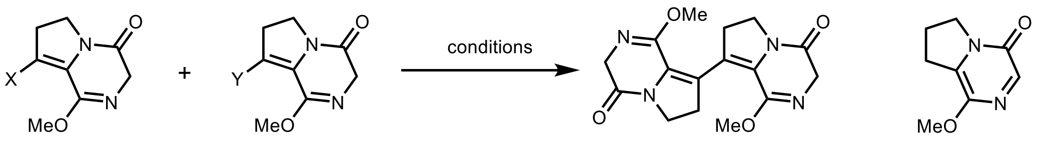

16 **18**

Entry	Reaction Name	X	Y	Conditions	Result
1	oxidative coupling	H	H	$Cu(OTf)_2$<br>MeCN, rt, 21 h	starting material
2	oxidative coupling	H	H	CAN<br>MeCN, rt, 22 h	decomp.
3	oxidative coupling	H	H	$PhI(OAc)_2$, $BF_3{\cdot}OEt_2$<br>CH_2Cl_2, rt, 20 h	decomp.
4	Ullmann	I	I	CuTC<br>NMP<br>70 °C, 21 h	mixture of **16** and protodehalogenation<br>no efficent way to remove the excess copper
5	Liu	I	I	Sm powder, CuI<br>THF<br>50 °C, 23 h	starting material
6	Murahashi–Ferringa	I	I	PdPEPSI-IPr<br>*n*-BuLi in hexanes<br>toluene, rt, 2 h	**18** alongside<br>partial decomp.
7	Wurtz	I	I	*i*-PrMgCl, THF<br>rt, 26 h	protodehalogenation<br>starting material<br>product (13.4:10.7:1.0)
8	Kumada–Tamao–Corriu	I	I	*i*-PrMgCl, THF, –20 °C, 30 min<br>*then*<br>$Pd(OAc)_2$, SPhos, rt, 20 min	protodehalogenation<br>and<br>starting material
9	Kumada–Tamao–Corriu	I	I	*i*-PrMgCl, THF, –20 °C, 40 min<br>*then*<br>$Pd(NCPh)_2Cl_2$, CuI, rt, 40 min	protodehalogenation<br>starting material<br>product (2.6:2.2:1.0)
10	Kumada–Tamao–Corriu	I	I	*i*-PrMgCl, THF, –20 °C, 40 min<br>*then*<br>$Pd(NCPh)_2Cl_2$, CuI, rt, 21 h	protodehalogenation<br>and<br>starting material
11	Negishi	I	I	$Pd(NCPh)_2Cl_2$<br>Zn, DMF<br>65 °C, 19 h	18%
12	Migita–Kosugi–Stille	I	$SnMe_3$	$Pd(NCPh)_2Cl_2$<br>CuI, DMF<br>65 °C, 24 h	unable to seperate product from organotin side product
13	oxidative coupling	$SnMe_3$	$SnMe_3$	$Cu(NO_3)_2{\cdot}3H_2O$<br>THF, rt, 2 h	protodestannylation

1.2.7. Experimental Procedure for Bis-Lactim Ether 16

Bis-lactim ether **16** was prepared according to a literature procedure.[7]

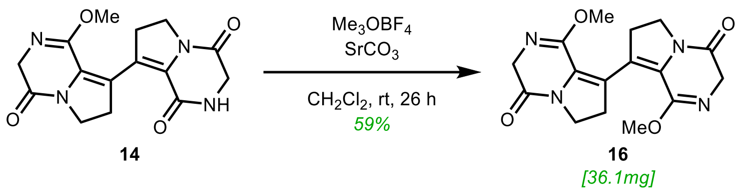

To a solution of bis-diketopiperazine **14** (58.9 mg, 1.0 equiv., 186 µmol) in CH_2Cl_2 (12 mL) under N_2 were added successively $SrCO_3$ (65.7 mg, 2.4 equiv., 444 µmol) and Me_3OBF_4 (143 mg, 5.2 equiv., 965 µmol). After 26 h of stirring at room temperature, the reaction was quenched with saturated aq. NH_4Cl (10 mL). The aqueous layer was extracted with CH_2Cl_2 (3 × 15 mL). The combined organics were dried over Na_2SO_4 and concentrated under reduced pressure. The crude residue was purified by column chromatography (silica gel, $CHCl_3$:MeOH, 50:1) to give bis-lactim ether **16** (36.1 mg, 109 µmol, 59%) as a brown solid.

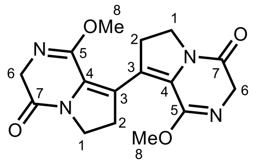

R$_f$ = 0.23 (CH_2Cl_2:MeOH, 10:1), [UV, $KMnO_4$, ninhydrin, *p*-anisaldehyde, PMA];

MP 153 °C (decomp.) ($CHCl_3$);

^{1}H NMR (500 MHz, $CDCl_3$): δ 4.37 (4H, app. t, *J* = 1.8 Hz, H-6), 3.93 (4H, app. t, *J* = 9.1 Hz, H-1), 3.68 (6H, s, H-8), 2.84 (4H, app. t, *J* = 9.7 Hz, H-2) ppm;

^{13}C NMR (126 MHz, $CDCl_3$): δ 164.5 (C-7), 153.2 (C-5), 125.2 (C-3 or C-4), 118.6 (C-3 or C-4), 53.1 (C-8), 53.0 (C-6), 43.2 (C-1), 32.3 (C-2) ppm;

HRMS (ESI^+) calc for $C_{16}H_{19}N_4O_4$ ($[M+H]^+$): 331.1401; found: 331.1397;

IR (film) ν_{max}/cm^{-1} 1669, 1607, 1432, 1405, 1328, 1252;

scXRD: Evaporation from a CH_2Cl_2 solution.

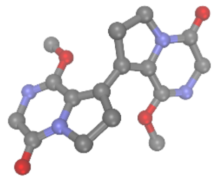

Table S5 – Base screen for the methylation of bis-diketopiperazine **14**. All the entries in this table follow the procedure outlined in chapter 1.2.5.. The internal standard used was durene. Entries 1–3, 5–6 and 12 were carried out at 60 mg scale and the remaining entries in the table were carried out at 10 mg scale. Entry 7 was carried out in the absence of light. TTBP = 2,4,6-tri-tert-butylpyrimidine.

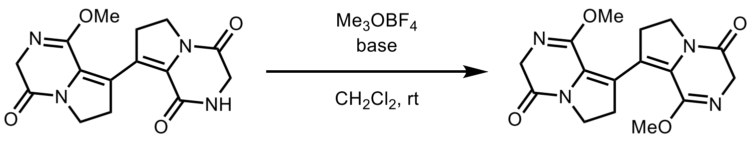

entry	base	reaction time /h	internal standard yield /%	isolated yield /%
1	Li_2CO_3	23	44	39
2	Na_2CO_3	22	–	38
3	K_2CO_3	20	–	47
4	Rb_2CO_3	23	46	–
5	Cs_2CO_3	27	–	27
6	$(NH_4)_2CO_3$	23	31	26
7	Ag_2CO_3	23	42	–
8	$SrCO_3$	23	61	–
9	$BaCO_3$	24	53	–
10	K_3PO_4	23	45	–
11	$Ca_2P_2O_7$	24	44	–
12	proton sponge	24	–	27
13	TTBP	25	37	–

1.2.8. Experimental Procedure for 3-chloroindole

3-Chloroindole is a commercially available reagent but was prepared on large scale according to a literature procedure.[11]

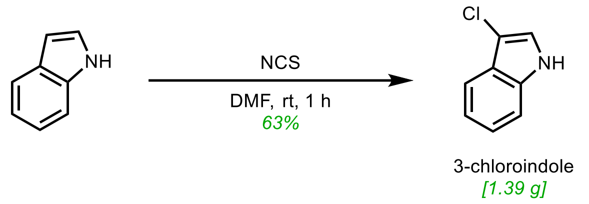

NCS (2.08 g, 1.1 equiv., 15.6 mmol) was added to a solution of indole (1.71 g, 1.0 equiv., 14.6 mmol) in DMF (60 mL) under N_2. The reaction stirred at room temperature for 1 h before being quenched with brine (30 mL), extracted with EtOAc (3 × 50 mL). The combined organics were dried over Na_2SO_4 and concentrated under reduced pressure. The crude residue was purified by column chromatography (silica gel, EtOAc:*n*-hexane, 1:10) to give 3-chloroindole (1.39 g, 9.16 mmol, 63%) as a colourless solid.

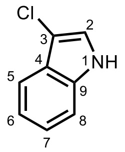

Data consistent with literature.[11]

$\mathbf{R_f}$ = 0.16 (EtOAc:*n*-hexane, 1:5), [UV, $Ce(SO_4)_2$, $KMnO_4$, *p*-anisaldehyde, PMA, vanillin];

MP 93 °C (decomp.) (EtOAc);

^{1}H NMR (600 MHz, $CDCl_3$): δ 7.87 (1H, app. s, H-1), 7.74 (1H, dm, *J* = 7.9 Hz, H-5), 7.34 (1H, dm, *J* = 8.1 Hz, H-8), 7.32 (1H, td, *J* = 8.0, 1.2 Hz, H-6), 7.28 (1H, td, *J* = 6.5, 1.5 Hz, H-7), 7.12 (1H, d, 2.6 Hz, H-2) ppm;

^{13}C NMR (151 MHz, $CDCl_3$): δ 135.0 (C-4), 125.4 (C-9), 123.2 (C-6), 121.0 (C-2), 120.6 (C-7), 118.3 (C-8), 11.6 (C-5), 106.4 (C-3) ppm;

HRMS (ESI^+) calc for C_8H_7ClN ($[M+H]^+$): 152.0262; found: 152.0260;

IR (film) ν_{max}/cm^{-1} 3407, 1454, 1333, 1202, 1087, 998, 738.

1.2.9. Experimental Procedure for 3-Methyl-1,2-butadiene

3-Methyl-1,2-butadiene is a commercially available reagent but was prepared on large scale according to a literature procedure.[12]

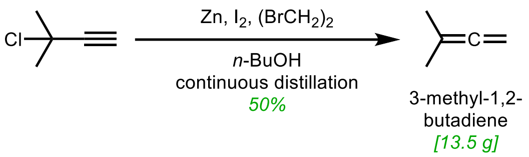

Zinc powder (52.3 g, 2.0 equiv., 800 mmol) was washed sequentially with aq. (1 M) HCl (4 × 40 mL), aq. (2%) $CuSO_4$ (2 × 60 mL), EtOH (2 × 60 mL) and *n*-BuOH (3 × 60 mL). The treated zinc was suspended in *n*-BuOH (110 mL) in a three-neck RBF with a dropping funnel, magnetic stirrer bar and a distillation head fitted at the top of a 30 cm Vigreux column. 3-Chloro-3-methyl-1-butyne (45.0 mL, 1.0 equiv., 396 mmol) and 1,2-dibromoethane (2.50 mL, 0.073 equiv., 29.0 mmol) were added to the dropping funnel. To the zinc slurry was added a few crystals of I_2 and 1,2-dibromoethane (2.50 mL, 0.073 equiv., 29.0 mmol). Then a portion of the alkyne mixture (3 mL) was added dropwise to the zinc slurry. The mixture was heated cautiously and when distillation began, the remaining alkyne mixture was added dropwise to the zinc slurry. External heating was applied, *via* a temperature-controlled heat gun, during the course of the reaction, keeping the boiling point of the distillate below 60 °C. The crude distillate was redistilled to give 3-methyl-1,2-butadiene (13.5 g, 198 mmol, 50%) as a colourless liquid.

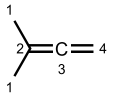

Data consistent with literature.[12]

BP 30–35 °C

^{1}H NMR (500 MHz, $CDCl_3$): δ 4.52 (2H, hpt, *J* = 3.2 Hz, H-4), 1.69 (6H, t, *J* = 3.1 Hz, H-1) ppm;

^{13}C NMR (126 MHz, $CDCl_3$): δ 206.8 (C-3), 94.2 (C-2), 72.7 (C-4), 20.3 (C-1) ppm.

1.2.10. Experimental Procedure for prenyl-9-BBN

A solution of prenyl-9-BBN was prepared according to a literature procedure.[11]

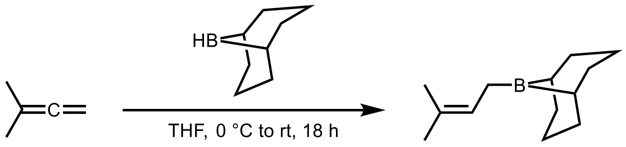

prenyl-9-BBN

3-Methyl-1,2-butadiene (5.00 mL, 1.0 equiv., 50.9 mmol) was added to a solution of 9-BBN in THF (130 mL, 1.3 equiv., 0.50 M, 65.0 mmol) under N_2 at 0 °C. The reaction stirred at room temperature for 18 h and was used in the procedure detailed in the following page without further purification.

1.2.11. Experimental Procedure for Indole S-1

Indole **S-1** was prepared according to a modified literature procedure.[13]

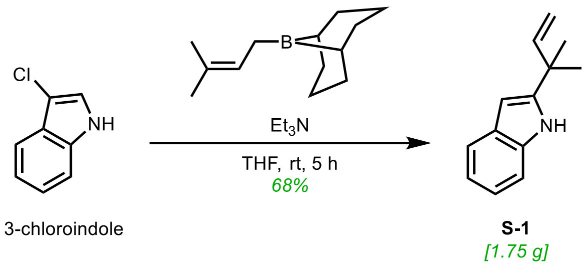

Et_3N (6.5 mL, 3.4 equiv., 46.6 mmol) was added dropwise to a solution of 3-chloroindole (2.10 g, 1.0 equiv., 13.8 mmol) in THF (40 mL) under N_2. After 25 minutes a freshly prepared solution of prenyl-9-BBN in THF (135 mL, 3.7 equiv., 50.9 mmol) was added. The reaction stirred at room temperature for 5 h before being concentrated under reduced pressure. The resulting mixture was triturated in *n*-hexane for 3 h and the solid impurities were removed by gravity filtration. The *n*-hexane solution was then concentrated under reduced pressure. The crude residue was purified by column chromatography (silica gel, EtOAc:*n*-hexane, 1:100) to give indole **S-1** (1.75 g, 9.42 mmol, 68%) as a yellow liquid.

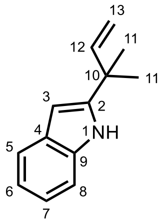

Data consistent with literature.[13]

R$_f$ = 0.14 (EtOAc:*n*-hexane, 1:30), 0.29 (EtOAc:*n*-hexane, 1:10), [UV, $Ce(SO_4)_2$, $KMnO_4$, *p*-anisaldehyde, PMA, vanillin];

^{1}H NMR (500 MHz, $CDCl_3$): δ 7.87 (1H, app. s, H-1), 7.56 (1H, dq, *J* = 7.9, 0.9 Hz, H-8), 7.30 (1H, dq, *J* = 8.0, 1.1 Hz, H-5), 7.13 (1H, ddd, *J* = 8.1, 7.1, 1.1 Hz, H-7), 7.07 (1H, ddd, *J* = 7.9, 7.1, 1.1 Hz, H-6), 6.32 (1H, dd, *J* = 2.2, 1.0 Hz, H-3), 6.05 (1H, dd, *J* = 17.2, 10.9 Hz, H-12), 5.14 (1H, dd, *J* = 7.4, 1.1 Hz, H-13), 5.11 (1H, app. s, H-13′), 1.49 (6H, s, H-11) ppm;

^{13}C NMR (126 MHz, $CDCl_3$): δ 146.2 (C-12), 145.9 (C-2), 136.0 (C-9), 128.7 (C-4), 121.4 (C-7), 120.3 (C-8), 119.8 (C-6), 112.4 (C-13), 110.6 (C-5), 98.1 (C-3), 38.3 (C-10), 27.6 (C-11) ppm;

HRMS (ESI$^+$) calc for $C_{13}H_{16}N$ ($[M+H]^+$): 186.1277; found: 186.1268;

IR (film) ν_{max}/cm^{-1} 3424, 1460, 1289.

1.2.12. Experimental Procedure for Aldehyde 3

Aldehyde **3** was prepared according to a modified literature procedure.[13]

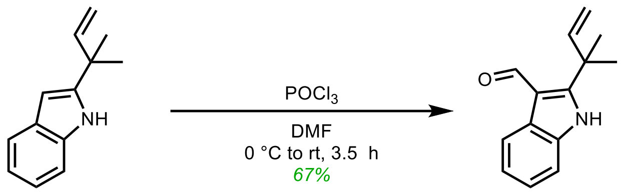

S-1 **3** [1.35 g]

$POCl_3$ (1.2 mL, 1.4 equiv., 12.9 mmol) was added dropwise to a DMF (4 mL) under N_2 at 0 °C. After 30 minutes of stirring at 0 °C a solution of indole **S-1** (1.74 g, 1.0 equiv., 9.42 mmol) in DMF (40 mL) was added dropwise. The reaction stirred at room temperature for 3.5 h before being cooled to 0 °C where the pH was adjusted to 9 by the addition of aq. (1 M) NaOH (40 mL). This mixture was extracted with EtOAc (3 × 200 mL) and the combined organics were washed with aq. (10%) LiCl (4 × 100 mL) before being concentrated under vacuum. The crude residue was purified by recrystallization from $CHCl_3$ to give aldehyde **3** (1.35 g, 6.33 mmol, 67%) as pale grey crystals.

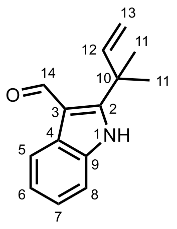

Data consistent with literature.[13]

$\mathbf{R_f}$ = 0.74 (CH_2Cl_2:MeOH, 10:1), 0.66 (EtOAc), [UV, $KMnO_4$, PMA, vanillin];

MP 189 °C ($CHCl_3$);

^{1}H NMR (600 MHz, $CDCl_3$): δ 10.48 (1H, s, H-14), 9.36 (1H, br. S, H-1), 8.38 (1H, dd, J = 8.2, 1.5 Hz, H-8), 7.42 (1H, dm, J = 7.8 Hz, H-5), 7.27 (1H, td, J = 7.1, 1.3 Hz, H-6), 7.26 (1H, td, J = 7.2, 1.4 Hz, H-7), 6.25 (1H, dd, J = 17.4, 10.5 Hz, H-12), 5.28 (1H, dd, J = 17.4, 0.7 Hz, H-$13_{\text{trans to H-12}}$), 5.25 (1H, dd, J = 10.6, 0.6 Hz, H-$13_{\text{cis to H-12}}$), 1.70 (6H, s, H-11) ppm;

^{13}C NMR (151 MHz, $CDCl_3$): δ 186.8 (C-14), 155.6 (C-2), 145.2 (C-12), 134.2 (C-9), 127.2 (C-3), 123.6 (C-7), 123.2 (C-6), 122.0 (C-8), 114.0 (C-4), 113.9 (C-13), 111.3 (C-5), 39.4 (C-10), 29.0 (C-11) ppm;

HRMS (ESI^+) calc for $C_{14}H_{16}NO$ ($[M+H]^+$): 214.1227; found: 214.1222;

IR (film) ν_{max}/cm^{-1} 2924, 1621, 1442, 906, 729.

1.2.13. Experimental Procedure for Enone 23

Enone **23** was prepared according to a modified literature procedure.[13]

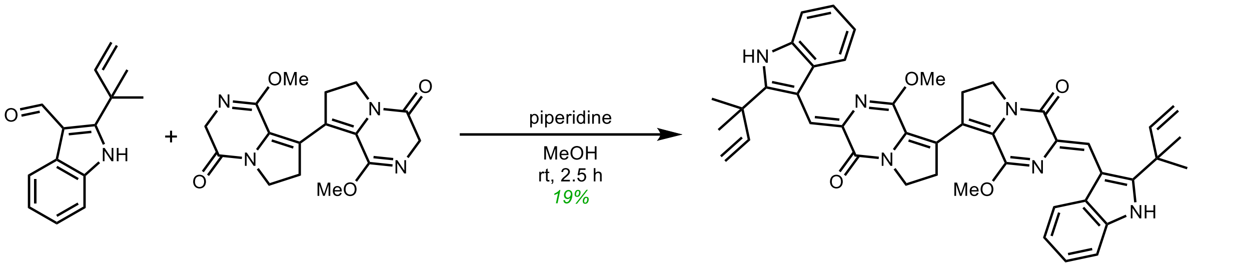

3 **16** **23** *[9.5 mg]*

A solution of aldehyde **3** (50.1 mg, 3.4 equiv., 235 µmol), bis-lactim ether **16** (23.1 mg, 1.0 equiv., 69.9 µmol) and piperidine (0.04 mL, 6.1 equiv., 425 µmol) in MeOH (2.5 mL) was stirred at room temperature for 2.5 h under N_2 before being concentrated under reduced pressure at room temperature. The crude residue was purified by column chromatography (silica gel, $CHCl_3$:acetone, 12:1) to give enone **23** (9.5 mg, 13.2 µmol, 19%) as a red solid.

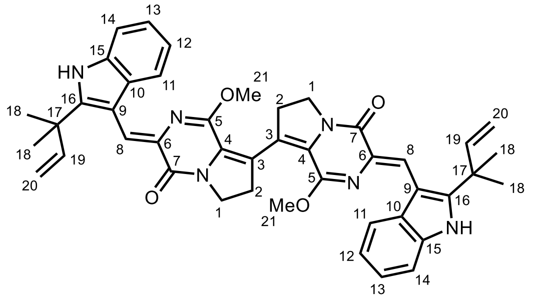

R$_f$ = 0.28 (Et_2O:THF, 1:2), 0.23 (EtOAc), 0.40 (Et_2O:MeOH, 10:1), 0.49 (CH_2Cl_2:MeOH, 15:1), 0.23 ($CHCl_3$:acetone, 5:1), [red with no stain, $KMnO_4$];

^{1}H NMR (500 MHz, $CDCl_3$): δ 8.33 (2H, s, N–H), 7.92 (2H, d, *J* = 8.3 Hz, H-14), 7.83 (2H, s, H-8), 7.32 (2H, d, *J* = 8.2 Hz, H-11), 7.16 (2H, ddd, *J* = 8.1, 6.9, 1.2 Hz, H-12), 7.06 (2H, ddd, *J* = 8.1, 7.1, 1.1 Hz, H-13), 6.15 (2H, dd, *J* = 17.4, 10.6 Hz, H-19), 5.30–5.24 (4H, m, H-20), 4.10 (4H, dd, *J* = 10.0, 8.6 Hz, H-1), 3.70 (6H, s, H-21), 2.98 (4H, dd, 9.9, 8.5 Hz, H-2), 1.62 (12H, s, H-18) ppm;

^{13}C NMR (126 MHz, $CDCl_3$): δ 159.4 (C-7), 150.9 (C-5), 145.6 (C-16), 144.8 (C-19), 134.4 (C-15), 130.0 (C-6), 128.0 (C-10), 127.3 (C-4), 124.1 (C-14), 124.0 (C-8), 121.9 (C-12), 119.5 (C-13), 118.1 (C-3), 113.4 (C-20), 110.6 (C-11), 109.1 (C-9), 54.0 (C-21), 43.9 (C-1), 39.5 (C-17), 32.5 (C-2), 28.0 (C-18) ppm;

HRMS (ESI^+) calc for $C_{44}H_{45}N_6O_4$ ($[M+H]^+$): 721.3497; found: 721.3483;

IR (film) ν_{max}/cm^{-1} 3293, 1643, 1572, 1455, 1388, 1331.

1.2.14. Experimental Procedure for Enone 22

Enone **22** was prepared according to a modified literature procedure.[13]

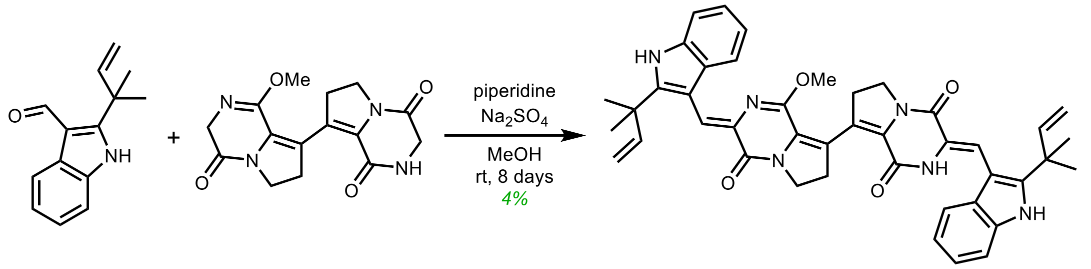

3 **14** **22**

[4.0 mg]

A suspension of aldehyde **3** (80.0 mg, 2.6 equiv., 375 µmol), bis-diketopiperazine **14** (45.1 mg, 1.0 equiv., 143 µmol), Na_2SO_4 (56.6 mg, 2.8 equiv., 398 µmol) and piperidine (0.05 mL, 3.7 equiv., 531 µmol) in MeOH (5 mL) was stirred at room temperature for 8 days under N_2 before being concentrated under reduced pressure at room temperature. The crude residue was purified by column chromatography (silica gel, CH_2Cl_2:MeOH, 40:1) to give enone **22** (4.0 mg, 5.66 µmol, 4%) as a red solid and mono-adduct **21** (23.1 mg, 45.2 µmol, 32%) as a orange solid (see chapter 1.3.11. for characterization of mono-adduct **21**)

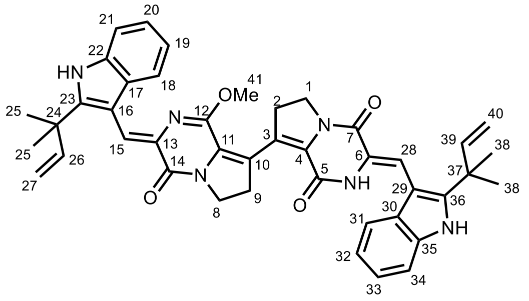

R$_f$ = 0.51 (CH_2Cl_2:MeOH, 10:1), 0.16 (CH_2Cl_2:THF, 10:1), [red with no stain, UV, $KMnO_4$]

^{1}H NMR (500 MHz, $CDCl_3$): δ 8.31 (1H, br. s, N–H between C-35 and C-36), 8.30 (1H, br. s, N–H between C-22 and C-23), 7.91 (1H, app. d, *J* = 8.2 Hz, H-18), 7.82 (1H, s, H-15), 7.54 (1H, br. s, N–H between C-5 and C-6), 7.36 (1H, dt, *J* = 8.0, 0.9 Hz, H-31), 7.31 (1H, dt, *J* = 8.0, 0.9 Hz, H-34), 7.30 (2H, app. d, *J* = 7.8 Hz, H-21), 7.20 (1H, ddd, *J* = 8.1, 7.1, 1.2 Hz, H-32), 7.17 (1H, s, H-28), 7.16–7.10 (2H, m, H-20 and H-33), 7.05 (1H, ddd, *J* = 8.1, 7.0, 1.1 Hz, H-19), 6.13 (1H, dd, *J* = 17.4, 10.5 Hz, H-26), 6.08 (1H, dd, *J* = 17.4, 10.6 Hz, H-39), 5.29–5.17 (4H, m, H-27, H-40), 4.16 (2H, dd, *J* = 10.0, 8.6 Hz, H-8), 4.09 (2H, dd, *J* = 10.0, 8.7 Hz, H-1), 3.72 (3H, s, H-41), 3.11 (2H, dd, *J* = 9.9, 8.6 Hz, H-2), 3.07 (2H, dd, *J* = 10.1, 8.8 Hz, H-9), 1.60 (6H, s, H-25), 1.54 (6H, s, H-38) ppm;

^{13}C NMR (126 MHz, $CDCl_3$): δ 159.3 (C-14), 155.4 (C-7), 154.0 (C-5), 150.3 (C-12), 145.7 (C-23), 144.7 (C-26), 144.4 (C-39), 144.1 (C-36), 134.5 (C-35), 134.4 (C-22), 129.8 (C-6), 128.4 (C-11) 128.3 (C-4), 127.9 (C-17), 126.2 (C-30), 126.1 (C-13), 124.7 (C-15), 124.4 (C-18), 122.6 (C-32), 121.9 (C-20), 121.3 (C-33), 119.6 (C-19), 119.1 (C-21), 117.4 (C-3), 113.6 (C-40), 113.4 (C-27), 111.5 (C-31), 110.5 (C-34), 110.3 (C-28), 109.1 (C-16), 103.6 (C-29), 54.2 (C-41), 44.4 (C-8), 44.1 (C-1), 39.5 (C-24), 39.4 (C-37), 32.8 (C-9), 31.8 (C-2), 28.0 (C-25), 27.5 (C-38) ppm;

*C-10 was not observed, it was presumably obscured by another peak.

*Additional NMR experiments (^{1}H-^{13}C HMBC [modified for 5 Hz and 2 Hz coupling] and ^{1}H-^{15}N HMBC) were performed to assign this structure. These additional experiments were performed on a Bruker AVANCE 800 spectrometer. See ^{15}N assignments below.

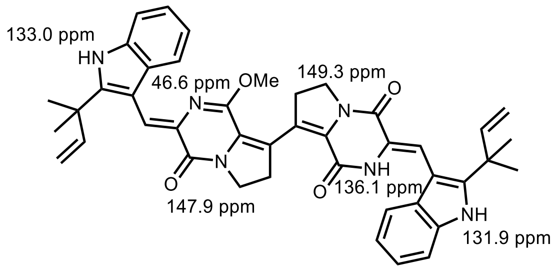

HRMS (ESI$^+$) calc for $C_{43}H_{43}N_6O_4$ ($[M+H]^+$): 707.3340; found: 707.3340;

IR (film) ν_{max}/cm^{-1} 3300, 1651, 1615, 1409, 1345, 1333, 1281, 1247.

1.2.15. Experimental Procedure for Brevianamide S (1)

Brevianamide S (**1**) was prepared according to a modified literature procedure.[14]

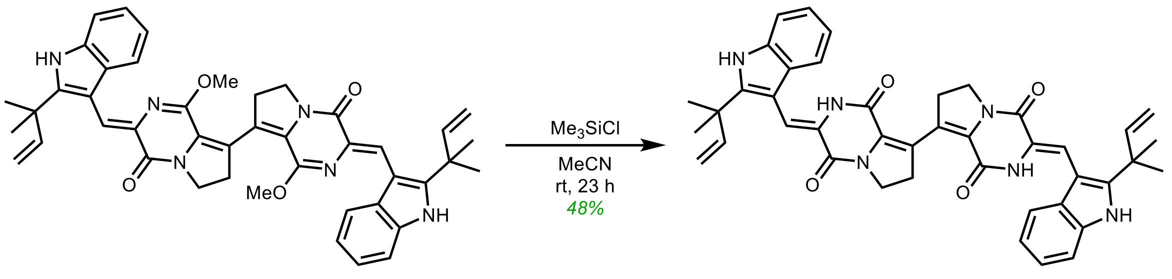

Me_3SiCl (40 µL, 6.2 equiv., 315 µmol) was added to a solution of enone **23** (36.8 mg, 1.0 equiv., 51.0 µmol) in MeCN (20 mL). The solution was stirred at room temperature for 21 h under N_2 before being exposed to air and stirred for a further 2 h. The solution was then concentrated under reduced pressure at room temperature. The crude residue was purified by column chromatography (silica gel, CH_2Cl_2:THF, 15:1) to give brevianamide S (**1**) (16.8 mg, 24.2 µmol, 48%) as an orange solid and enone **22** (7.2 mg, 10.2 µmol, 20%) as a red solid (see previous page for spectroscopic data regarding enone **22**).

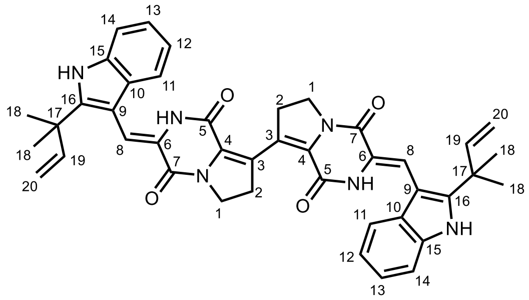

Data consistent with literature.[15]

R$_f$ = 0.27 (CH_2Cl_2:THF, 10:1), [yellow with no stain, UV, $KMnO_4$, PMA];

^{1}H NMR (500 MHz, $CDCl_3$): δ 8.37 (2H, br. s, N–H between C-15 and C-16), 7.57 (2H, br. s, N–H between C-5 and C-6), 7.35 (2H, ddd, J = 8.1, 1.0, 1.0 Hz, H-11), 7.30 (2H, dd, 7.7, 1.2 Hz, H-14), 7.18 (2H, ddd, J = 8.3, 7.1, 1.3 Hz, H-12), 7.17 (2H, s, H-8), 7.14 (2H, ddd, J = 8.1, 7.1, 1.2 Hz, H-13), 6.06 (2H, dd, J = 17.4, 10.6 Hz, H-19), 5.22 (2H, dd, J = 10.5, 0.9 Hz, H-20$_{\text{cis to H-19}}$), 5.20 (2H, dd, J = 17.4, 0.9 Hz, H-20$_{\text{trans to H-19}}$), 4.16 (4H, dd, J = 10.1, 8.6 Hz, H-1), 3.21 (4H, dd, J = 10.1, 8.6 Hz), 1.52 (12H, s, H-18) ppm;

^{1}**H NMR** (500 MHz, CD_3OD): δ 7.43 (2H, ddd, *J* = 8.1, 0.9, 0.9 Hz, H-14), 7.29 (2H, ddd, *J* = 8.0, 1.0, 1.0 Hz, H-11), 7.18 (2H, s, H-8), 7.12 (2H, ddd, *J* = 8.1, 7.1, 1.2 Hz, H-13), 7.03 (2H, ddd, *J* = 8.1, 7.1, 1.1 Hz, H-12), 6.11 (2H, dd, *J* = 17.3, 10.6 Hz, H-19), 5.11 (2H, dd, *J* = 10.7, 1.1 Hz, $H\text{-}20_{\text{cis to H-19}}$), 5.10 (2H, dd, *J* = 17.4, 1.1 Hz, $H\text{-}20_{\text{trans to H-19}}$), 4.10 (4H, dd, *J* = 9.9, 8.5 Hz, H-1), 3.13 (4H, dd, *J* = 9.9, 8.4 Hz, H-2), 1.54 (12H, s, H-18) ppm;

^{13}**C NMR** (126 MHz, $CDCl_3$): δ 155.3 (C-5), 154.0 (C-7), 144.4 (C-19), 144.2 (C-16), 134.5 (C-15), 129.0 (C-3 or C-4), 127.9 (C-3 or C-4), 126.2 (C-10), 125.8 (C-6), 122.5 (C-12), 121.3 (C-13), 119.2 (C-14), 113.5 (C-20), 111.4 (C-11), 110.9 (C-8), 103.9 (C-9), 44.6 (C-1), 39.4 (C-17), 31.8 (C-2), 27.5 (C-18) ppm;

^{13}**C NMR** (126 MHz, CD_3OD): δ 157.5 (C-7), 155.9 (C-5), 146.3 (C-16), 146.2 (C-19), 136.8 (C-15), 129.9 (C-3), 129.2 (C-4), 127.2 (C-10), 125.8 (C-6), 122.6 (C-13), 121.4 (C-12), 119.9 (C-11), 113.5 (C-8), 112.7 (C-14), 112.6 (C-20), 104.6 (C-9), 45.6 (C-1), 40.5 (C-17), 32.5 (C-2), 28.2 (C-18) ppm;

HRMS (ESI^+) calc for $C_{42}H_{41}N_6O_4$ ($[M+H]^+$): 693.3184; found: 693.3175;

IR (film) ν_{max}/cm^{-1} 3357, 1663, 1616, 1390, 1337.

Table S6 - Comparative NMR data for synthetic and natural brevianamide S (**1**) in CD_3OD.[14]

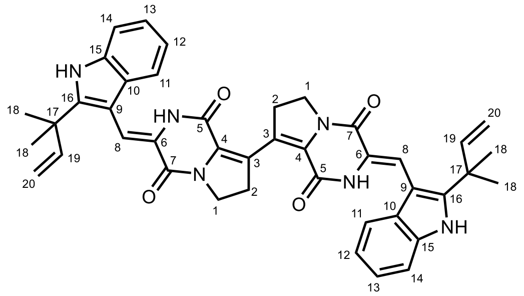

C	Synthetic δH	Synthetic δC	Natural δH	Natural δC	ΔδC
1	4.10, dd (9.9, 8.5)	45.6	4.11, dd (9.6, 9.0)	45.6	0
2	3.13, dd (9.9, 8.4)	32.5	3.13, dd (9.6, 9.0)	32.5	0
3	–	129.9	–	129.9	0
4	–	129.2	–	129.2	0
5	–	155.9	–	156.0	0.1
6	–	125.8	–	125.8	0
7	–	157.5	–	157.5	0
8	7.18, s	113.5	7.18, s	113.5	0
9	–	104.6	–	104.6	0
10	–	127.2	–	127.2	0
11	7.29, ddd (8.0, 1.0, 1.0)	119.9	7.29, ddd (7.8, 1.2, 0.6)	119.9	0
12	7.03, ddd (8.1, 7.1, 1.1)	121.4	7.04, ddd (7.8, 7.8, 1.2)	121.4	0
13	7.12, ddd (8.1, 7.1, 1.2)	122.6	7.13, ddd (7.8, 7.8, 1.2)	122.6	0
14	7.43, ddd (8.1, 0.9, 0.9)	112.7	7.43, ddd (7.8, 1.2, 0.6)	112.7	0
15	–	136.8	–	136.9	0.1
16	–	146.3	–	146.3	0
17	–	40.5	–	40.5	0
18	1.54, s	28.2	1.54, s	28.2	0
19	6.11, dd (17.3, 10.6)	146.2	6.11, dd (17.4, 10.8)	146.2	0
20A	5.10, dd (17.4, 1.1)	112.6	5.10, dd (17.4, 1.2)	112.6	0
20B	5.11, dd (10.7, 1.1)	112.6	5.11, dd (10.8, 1.2)	112.6	0

1.2.16. Additional Experimental Procedure for Brevianamide S (1)

Brevianamide S (**1**) was prepared according to a modified literature procedure.[14]

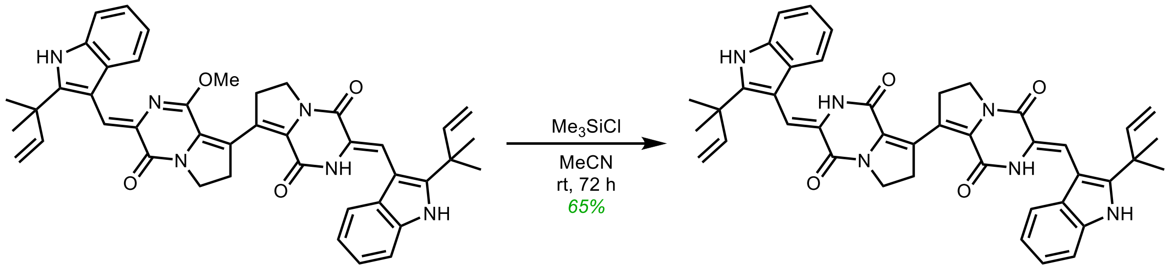

22

1
brevianamide S
[3.2 mg]

Me_3SiCl (40 µL, 44.6 equiv., 315 µmol) was added to a solution of enone **22** (5.0 mg, 1.0 equiv., 7.07 µmol) in MeCN (15 mL). The solution was stirred at room temperature for 72 h under N_2 before being exposed to air and stirred for a further 4 h. The solution was then concentrated under reduced pressure at room temperature. The crude residue was purified by column chromatography (silica gel, CH_2Cl_2:THF, 15:1) to give brevianamide S (**1**) (3.2 mg, 4.61 µmol, 65%) as an orange solid.

See previous page for characterization of brevianamide S (**1**).

1.3. Other Experimental Procedures

1.3.1. Experimental Procedure for Dehydroproline 7

Dehydroproline **7** was prepared according to a literature procedure.[2]

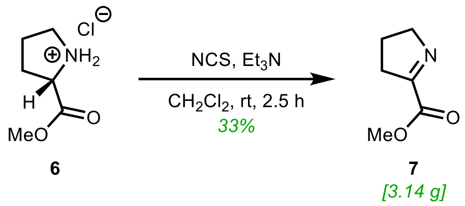

To a solution of L-proline methyl ester hydrochloride **6** (12.5 g, 1.0 equiv., 75.5 mmol) and Et_3N (24.5 mL, 2.3 equiv., 175.8 mmol) in CH_2Cl_2 (225 mL) at 0 °C under N_2 was added NCS (11.2 g, 1.1 equiv., 83.6 mmol) in ten equal portions over an hour. The mixture was then stirred at room temperature for 2.5 h and then diluted with CH_2Cl_2 (175 mL). A mixture of saturated aq. NH_4Cl (100 mL) and water (100 mL) was added to the reaction mixture and the layers separated. The aqueous phase was then extracted with CH_2Cl_2 (2 × 100 mL). The combined organics were washed successively with a mixture of saturated aq. NH_4Cl (100 mL) and water (100 mL), saturated aq. $NaHCO_3$ (200 mL) and brine (200 mL) before being dried over Na_2SO_4 and concentrated under reduced pressure (100 mbar, 40 °C). Vacuum distillation (4 mbar, 64–68 °C) gave dehydroproline **7** (3.14 g, 24.7 mmol, 33%) as a clear colourless oil.

*This colourless oil slowly turned yellow over several days. ^1H and ^{13}C NMR spectra of this yellow oil were identical to those of the product.

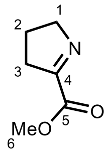

Data consistent with literature.[16]

BP 64–68 °C (4 mbar);

^{1}H NMR (600 MHz, $CDCl_3$): δ 4.08 (2H, tt, J = 7.5, 2.5 Hz, H-1), 3.84 (3H, s, H-6), 2.80 (2H, tt, J = 8.1, 2.5 Hz, H-3), 1.96 (2H, dddd, J = 9.1, 7.8, 7.7, 7.7 Hz, H-2) ppm;

^{13}C NMR (126 MHz, $CDCl_3$): δ 168.4 (C-4), 163.3 (C-5), 62.6 (C-1), 52.6 (C-6), 35.3 (C-3), 22.2 (C-2) ppm;

HRMS (ESI^+) calc for $C_6H_{10}NO_2$ ($[M+H]^+$): 128.0706; found: 128.0707;

IR (film) ν_{max}/cm^{-1} 1720, 1252, 1105.

1.3.2. Experimental Procedure for β-lactam (±)-9

β-lactam (±)-**9** was prepared according to a modified literature procedure.[2]

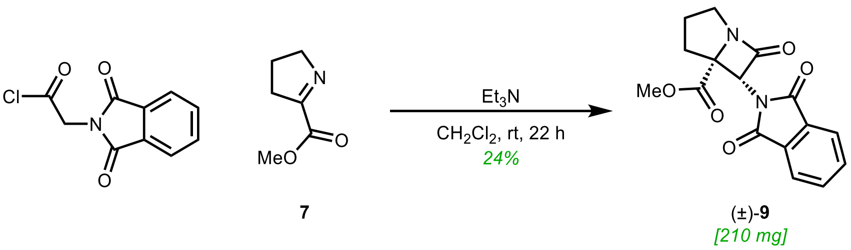

A solution of dehydroproline **7** (510 mg, 1.4 equiv., 4.0 mmol) and Et_3N (0.47 mL, 1.1 equiv., 3.4 mmol) in CH_2Cl_2 (3 mL) was added with a syringe pump over 1 h to a precooled (0 °C) solution of phthalylglycyl chloride (600 mg, 1.0 equiv., 2.7 mmol) in CH_2Cl_2 (14 mL) under N_2. The solution was stirred at room temperature for 22 h before being quenched with aq. (1 M) HCl (20 mL). The aqueous layer was then extracted with CH_2Cl_2 (3 × 20 mL). The combined organic layers were successively washed with saturated aq. $NaHCO_3$ (100 mL) and brine (100 mL), dried over Na_2SO_4 and concentrated under reduced pressure. The crude residue was purified by column chromatography (silica gel, Et_2O:MeOH, 10:1) to give β-lactam (±)-**9** (210 mg, 0.65 mmol, 24%) as a white solid.

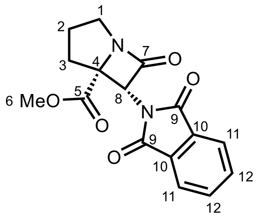

R_f = 0.23 (Et_2O), [UV, ninhydrin];

MP 195 °C (Et_2O);

^{1}H NMR (500 MHz, $CDCl_3$): δ 7.88 (2H, dd, J = 5.5, 2.9 Hz, H-11), 7.75 (2H, dd, J = 5.4, 3.1 Hz, H-12), 5.20 (1H, s, H-8), 3.87 (1H, ddd, J = 11.2, 7.3, 5.7 Hz, H-1), 3.63 (3H, s, H-6), 3.23 (1H, ddd, J = 11.3, 7.5, 5.4 Hz, H-1′), 2.35-2.12 (4H, m, H-2, H-3) ppm;

^{13}C NMR (126 MHz, $CDCl_3$): δ 170.6 (C-5), 170.1 (C-7), 166.8 (C-9), 134.6 (C-12), 131.7 (C-10), 124.0 (C-11), 69.1 (C-4), 61.0 (C-8), 52.7 (C-6), 46.7 (C-1), 34.0 (C-3), 29.1 (C-2) ppm;

HRMS (ESI^+) calc for $C_{16}H_{15}N_2O_5$ ($[M+H]^+$): 315.0976; found: 315.0965;

IR (film) ν_{max}/cm^{-1} 1770, 1717, 1386, 1299, 1202, 1060, 910, 896, 717;

scXRD: Slow evaporation of a saturated solution (Et_2O).

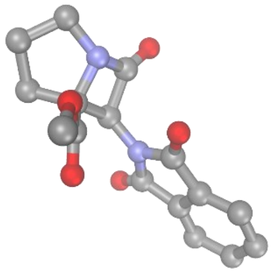

1.3.3. Experimental Procedure for Iodide S-2

Iodide **S-2** was prepared according to a modified literature procedure.[17]

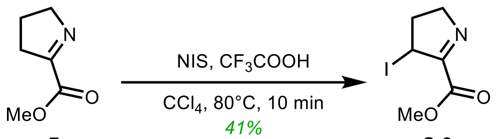

To a stirred mixture of dehydroproline **7** (0.51 g, 1.0 equiv., 4.0 mmol) in CCl_4 (6 mL) under N_2, was added NIS (0.89 g, 1.0 equiv., 3.9 mmol) and CF_3COOH (30 µL, 1.0 equiv., 3.9 mmol). The reaction mixture was heated to 80 °C for 10 minutes before being cooled to room temperature, then quenched with water (20 mL) and diluted with $CHCl_3$ (20 mL). The aqueous layer was extracted with $CHCl_3$ (3 × 20 mL). The combined organic layers were washed with saturated aq. $Na_2S_2O_3$ (100 mL), water (3 × 100 mL), dried over Na_2SO_4 and concentrated under reduced pressure. The crude residue was purified by column chromatography (silica gel, CH_2Cl_2:MeOH, 99:1) to give iodinide **S-2** (0.41 g, 1.62 mmol, 41%) as a black oil.

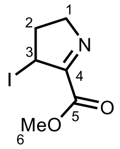

R_f = 0.21 (CH_2Cl_2:MeOH, 99:1), [UV, $KMnO_4$];

^{1}H NMR (500 MHz, $CDCl_3$): δ 5.18 (1H, ddd, J = 7.4, 1.7, 1.2 Hz, H-3), 4.16 (1H, ddd, J = 18.0, 7.5, 1.3 Hz, H-1), 3.93 (3H, s, H-6), 3.97-3.89 (1H, m, H-1′), 2.44 (1H, ddt, J =14.9, 6.4, 1.2 Hz, H-2), 2.36 (1H, ddt, J = 14.9, 9.0 7.4 Hz, H-2′) ppm;

^{13}C NMR (126 MHz, $CDCl_3$): δ 168.1 (C-4), 161.1 (C-5), 60.5 (C-1), 53.3 (C-6), 38.0 (C-2), 22.3 (C-3) ppm;

HRMS (ESI$^+$) calc for $C_6H_8INO_2Na$ ($[M+Na]^+$): 275.9492; found: 275.9488;

IR (film) ν_{max}/cm^{-1} 2951, 1736, 1433, 1289, 1235, 1204, 780.

1.3.4. Experimental Procedure for β-lactam (±)-S-3

B-lactam (±)-**S-3** was prepared according to a modified literature procedure.[2]

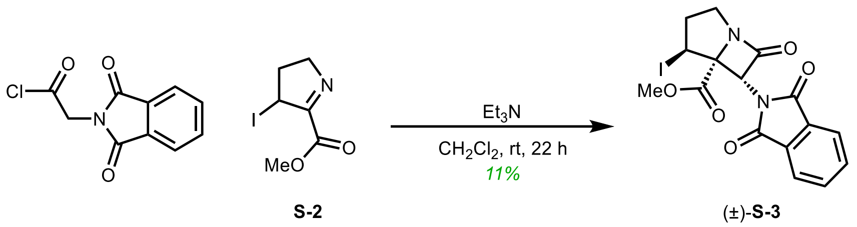

A solution of iodide **S-2** (120 mg, 1.0 equiv., 480 μmol) and Et_3N (0.10 mL, 1.5 equiv., 720 μmol) in CH_2Cl_2 (1.1 mL) was added with a syringe pump over 1 h at 0 °C to a solution of a phthalylglycyl chloride (0.26 g, 2.4 equiv., 1.17 mmol) in CH_2Cl_2 (6 mL) under N_2. The solution was stirred at room temperature for 22 h before being quenched with aq. (1 M) HCl (20 mL). The aqueous layer was then extracted with CH_2Cl_2 (3 × 20 mL). The combined organic layers were successively washed with saturated aq. $NaHCO_3$ (100 mL) and brine (100 mL), dried over Na_2SO_4 and concentrated under reduced pressure. The crude residue was purified by column chromatography (silica gel, EtOAc:*n*-hexane, 1:1) to give β-lactam (±)-**S-3** (23 mg, 52 μmol, 11%) as a colourless solid.

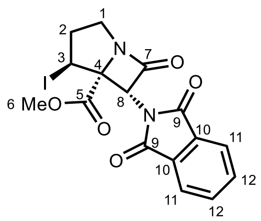

$\mathbf{R_f}$ = 0.56 (Et_2O:CH_2Cl_2, 1.1), 0.44 (EtOAc:*n*-hexane, 3:1), [UV, $KMnO_4$];

MP 199 °C (decomp.) (Et_2O);

^{1}H NMR (600 MHz, $CDCl_3$): δ 7.86 (2H, dd, *J* = 5.3, 3.1 Hz, H-11), 7.76 (2H, dd, *J* = 5.4, 3.0 Hz, H-12), 5.80 (1H, s, H-8), 4.62 (1H, t, *J* = 5.4 Hz, H-3), 3.92 (1H, dt, *J* = 11.3, 6.7 Hz, H-1), 3.63 (3H, s, H-6), 3.24 (1H, ddd, *J* = 11.2, 7.1, 5.6 Hz, H-1′), 2.85 (1H, app. dq, *J* = 13.3, 6.4 Hz, H-2), 2.52 (1H, ddt, *J* = 14.0, 6.8, 5.5 Hz, H-2’) ppm;

^{13}C NMR (151 MHz, $CDCl_3$): δ 169.2 (C-7), 167.8 (C-5), 166.5 (C-9), 134.8 (C-12), 131.6 (C-10), 124.0 (C-11), 72.7 (C-4), 66.3 (C-8), 53.3 (C-6), 46.3 (C-1), 42.2 (C-2), 23.4 (C-3) ppm;

HRMS (ESI$^+$) calc for $C_{16}H_{14}IN_2O_5$ ($[M+H]^+$): 440.9942; found: 440.9957;

IR (film) ν_{max}/cm^{-1} 1779, 1723, 1386, 1295, 1053, 719;

scXRD: Slow evaporation of a saturated solution (Et_2O).

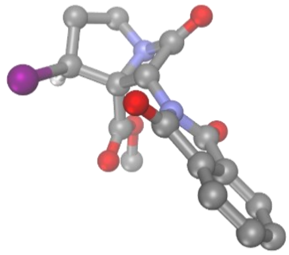

1.3.5. Experimental Procedure for Hemiaminal S-4

Hemiaminal **S-4** was prepared according to a modified literature procedure.[18]

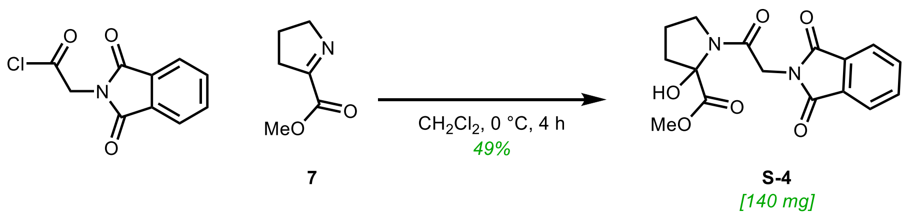

A solution of phthalylglycyl chloride (190 mg, 1.0 equiv., 840 µmol) in CH_2Cl_2 (5 mL) was added, dropwise over 10 min to a precooled (0 °C) solution of dehydroproline **7** (110 mg, 1.0 equiv., 860 µmol) in CH_2Cl_2 (25 mL) under N_2. The reaction mixture was stirred at 0 °C for 4 h before it was quenched with saturated aq. $NaHCO_3$ (50 mL). The aqueous layer was extracted with CH_2Cl_2 (3 × 50 mL) and the combined organic layers were washed with brine (200 mL), dried over Na_2SO_4 and concentrated under reduced pressure. The crude residue was purified by column chromatography (silica gel, EtOAc:CH_2Cl_2, 1:3) to give hemiaminal **S-4** (140 mg, 400 µmol, 49%) as a white solid.

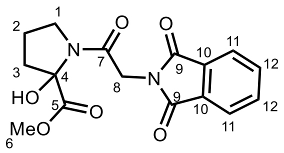

R_f = 0.29 (EtOAc:CH_2Cl_2, 1:1), [UV, $KMnO_4$];

MP 109 –116 °C (EtOAc);

^{1}H NMR (500 MHz, $CDCl_3$): δ 7.86 (2H, dd, J = 5.3, 3.0 Hz, H-11), 7.72 (2H, dd, J = 5.4, 3.0 Hz, H-12), 4.52 (1H, app. s, O–H), 4.42 (2H, ABq, J = 16.6 Hz, H-8), 3.83 (1H, m, H-1), 3.76 (3H, s, H-6), 3.72 (1H, m, H-1′), 2.25 (2H, m, H-2, H-3), 2.14 (2H, m, H-2′, H-3′) ppm;

^{13}C NMR (126 MHz, $CDCl_3$): δ 172.1 (C-5), 161.8 (C-9), 165.5 (C-7), 134.3 (C-12), 132.3 (C-10), 123.7 (C-11), 89.6 (C-4), 53.5 (C-6), 46.8 (C-1), 39.7 (C-8), 37.4 (C-2), 23.8 (C-3) ppm;

HRMS (ESI$^+$) calc for $C_{16}H_{17}N_2O_6$ ([M+H]$^+$): 333.1081; found: 333.1089;

IR (film) ν_{max}/cm^{-1} 3443, 1717, 1654, 1418, 1389, 1325, 1137, 1117, 953, 769, 713;

scXRD: Evaporation of a saturated solution (Et_2O).

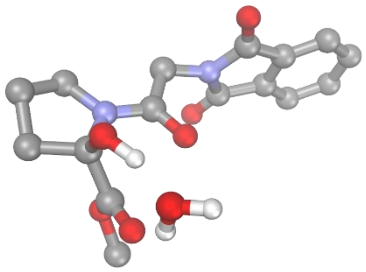

1.3.6. Experimental Procedure for Lactim Ether S-5

Lactim ether **S-5** was prepared according to a modified literature procedure.[7]

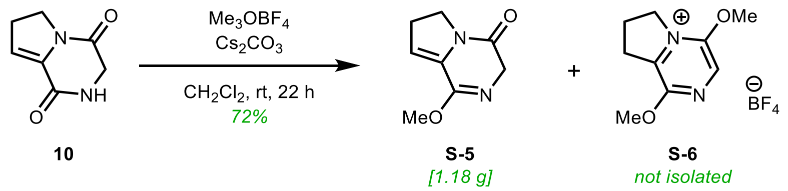

To a solution of diketopiperazine **10** (1.50 g, 1.0 equiv., 9.84 mmol) in CH_2Cl_2 (200 mL) under N_2 were added successively Cs_2CO_3 (1.63 g, 0.5 equiv., 5.01 mmol) and Me_3OBF_4 (1.48 g, 1.0 equiv., 10.0 mmol). After 22 h of stirring at room temperature, the reaction was quenched with water (50 mL). The aqueous layer was extracted with CH_2Cl_2 (3 × 300 mL). The combined organics were dried over Na_2SO_4 and concentrated under reduced pressure. The crude residue was purified by column chromatography (silica gel, EtOAc) to give lactim ether **S-5** (1.18 g, 7.10 mmol, 72%) as colourless needles.

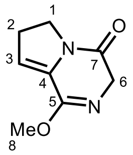

$\mathbf{R_f}$ = 0.30 (CH_2Cl_2:MeOH, 10:1), 0.16 (EtOAc), [UV, $Ce(SO_4)_2$, $KMnO_4$, PMA];

MP 89–99 °C (EtOAc);

^{1}H NMR (500 MHz, $CDCl_3$): δ 5.58 (1H, tt, *J* = 3.0, 1.1 Hz, H-3), 4.32 (2H, app. q, *J* = 1.6 Hz, H-6), 3.92 (2H, dd, *J* = 9.9, 8.5 Hz, H-1), 3.74 (3H, s, H-8), 2.75–2.69 (2H, m, H-2) ppm;

^{13}C NMR (126 MHz, $CDCl_3$): δ 164.1 (C-7), 152.9 (C-5), 130.0 (C-4), 111.6 (C-3), 53.2 (C-6), 53.0 (C-8), 44.3 (C-1), 27.9 (C-2) ppm;

HRMS (ESI$^+$) calc for $C_8H_{11}N_2O_2$ ($[M+H]^+$): 167.0815; found: 167.0818;

IR (film) ν_{max}/cm^{-1} 1679, 1653, 1624, 1450, 1433, 1403, 1332, 1247.

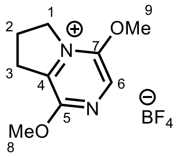

^{1}H NMR (500 MHz, $CDCl_3$): δ 8.18 (1H, s, H-6), 4.78 (2H, tt, J = 7.8, 1.1 Hz, H-1), 4.29 (3H, s, H-8 or H-9), 4.09 (3H, s, H-8 or H-9), 3.50 (2H, tt, J = 8.0 Hz, 1.0 Hz, H-3), 2.58 (2H, app. qu, J = 7.9 Hz, H-2) ppm;

^{11}B NMR (128 MHz, $CDCl_3$): δ −1.54 ppm;

^{13}C NMR (126 MHz, $CDCl_3$): δ 154.2 (C-5 or C-7), 149.0 (C-5 or C-7), 140.4 (C-4), 127.5 (C-6), 59.4 (C-8 or C-9), 56.1 (C-1), 55.9 (C-8 or C-9), 30.1 (C-3) 20.4 (C-2) ppm;

^{19}F NMR (376 MHz, $CDCl_3$): δ −155.1 ppm;

HRMS (ESI^+) calc for $C_9H_{13}N_2O_2$ ($[M-BF_4]^+$): 181.0972; found: 181.0973;

scXRD: Evaporation of a solution ($CDCl_3$).

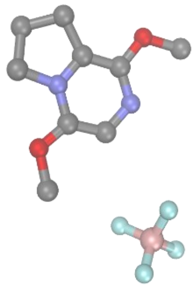

1.3.7. Experimental Procedure for Pyrazinone 18

An authentic sample of pyrazinone **18** was prepared according to a modified literature procedure.[7]

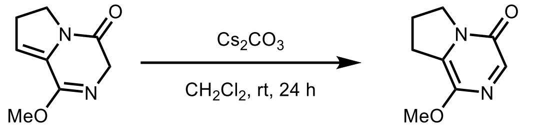

S-5 **18**
[21.1 mg (impure)]

Cs_2CO_3 (134.4 mg, 2.8 equiv., 412 μmol) was added to a solution of lactim ether **S-5** (24.6 mg, 1.0 equiv., 148 μmol) in CH_2Cl_2 (4.5 mL) under N_2. After 24 h of stirring at room temperature, the reaction was concentrated under reduced pressure. The resulting solid was diluted with CH_2Cl_2 (25 mL) and water (15 mL). The aqueous layer was extracted with CH_2Cl_2 (2 × 20 mL). The combined organics were dried over Na_2SO_4 and concentrated under reduced pressure to give pyrazinone **18** (21.1 mg) as an impure amorphous yellow solid.

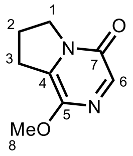

$\mathbf{R_f}$ = 0.45 (CH_2Cl_2:MeOH, 10:1), [UV];

^{1}H NMR (500 MHz, $CDCl_3$): δ 7.68 (1H, t, J = 1.3 Hz, H-6), 4.16 (2H, t, J = 7.5 Hz, H-1), 3.86 (3H, s, H-8), 3.08 (2H, td, J = 7.8, 1.3 Hz, H-3), 2.24 (2H, app. qu, J = 7.5 Hz, H-2) ppm;

^{13}C NMR (126 MHz, $CDCl_3$): δ 155.2 (C-7), 144.3 (C-5), 140.3 (C-6), 127.8 (C-4), 54.9 (C-8), 49.7 (C-1), 28.5 (C-3), 21.2 (C-2) ppm;

HRMS (ESI$^+$) calc for $C_8H_{11}N_2O_2$ ($[M+H]^+$): 167.0815; found: 167.0810;

IR (film) ν_{max}/cm^{-1} 1676, 1660, 1570, 1301, 1165, 1061.

1.3.8. Experimental Procedure for Compound S-7

To test the competency of alkenyl iodide **11** in a palladium catalysed cross-coupling reaction, compound **S-7** was prepared according to a modified literature procedure.[19]

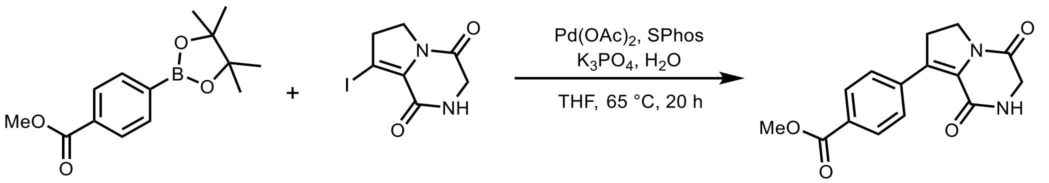

S-8 **11** **S-7**

[23.8 mg (impure)]

To a solution of $Pd(OAc)_2$ (2.7 mg, 7 mol%, 12.0 μmol) and SPhos (7.1 mg, 10 mol%, 17.3 μmol) in THF (1 mL) under N_2 were added successively boronic ester **S-8** (114 mg, 2.4 equiv., 436 μmol), alkenyl iodide **11** (50.7 mg, 1.0 equiv., 182 μmol), K_3PO_4 (115 mg, 3.0 equiv., 540 μmol) and H_2O (30 μL, 9.0 equiv., 1.66 mmol). After 3.5 h of stirring at room temperature the reaction mixture was heated to 65 °C for 20 h. Then the reaction was cooled and passed through a small pad of celite and washed with THF:H_2O (20:1) (80 mL) before being concentrated under reduced pressure. The crude residue was partially purified by column chromatography (silica gel, CH_2Cl_2:MeOH, 20:1) to give compound **S-7** (23.8 mg) as an impure, colourless solid.

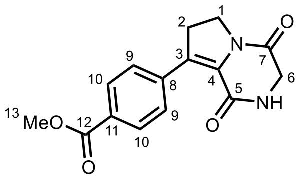

$\mathbf{R_f}$ = 0.26 (CH_2Cl_2:MeOH, 10:1), [UV, $KMnO_4$];

^{1}H NMR (600 MHz, $CDCl_3$) NMR data reported for compound **57**, impurities not listed: δ 8.02 (2H, d, J = 8.1 Hz, H-10), 7.64 (2H, d, J = 8.5 Hz, H-9), 6.04 (1H, br. s, N–H), 4.20 (2H, s, H-6), 4.07 (2H, app, t, J = 9.4 Hz, H-1), 3.92 (3H, s, H-13), 3.13 (2H, app. t, J = 9.3 Hz, H-2) ppm;

^{13}C NMR (126 MHz, $CDCl_3$) NMR data reported for compound **57**, impurities not listed: δ 166.8 (C-12), 160.4 (C-5 or C-7), 157.6 (C-5 or C-7), 137.6 (C-11), 133.5 (C-8), 130.2 (C-4), 129.2 (C-9 or C-10), 129.1 (C-9 or C-10), 125.5 (C-3), 52.3 (C-13), 46.4 (C-6), 43.4 (C-1), 33.1 (C-2) ppm;

HRMS (ESI$^+$) calc for $C_{15}H_{15}N_2O_4$ ([M+H]$^+$): 287.1026; found: 287.1025;

IR (film) ν_{max}/cm^{-1} (for an impure compound) 3220, 1690, 1674, 1603, 1436, 1279, 1111.

1.3.9. Experimental Procedure for Organotin S-9

Organotin **S-9** was prepared according to a modified literature procedure.[10]

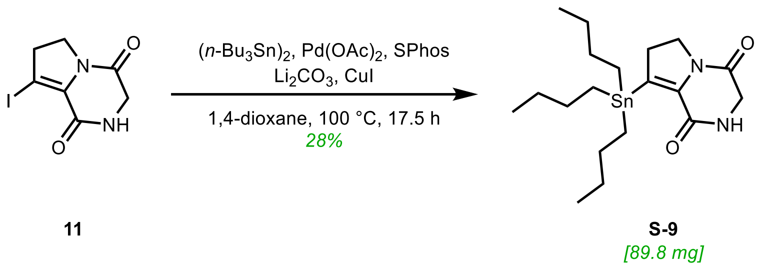

To a slurry of alkenyl iodide **11** (202 mg, 1.0 equiv., 726 µmol), Li_2CO_3 (151 mg, 2.8 equiv., 2.04 mmol), (n-$Bu_3Sn)_2$ (0.72 mL, 2.0 equiv., 1.42 mmol) in 1,4-dioxane (20 mL) under N_2 were added successively $Pd(OAc)_2$ (36.2 mg, 22 mol%, 161 µmol), SPhos (128 mg, 43 mol%, 313 µmol) and CuI (47.6 mg, 34 mol%, 250 µmol). The reaction was stirred at 100 °C for 17.5 h before being passed through a small pad of celite and washed with EtOAc (200 mL), then concentrated under reduced pressure. The crude residue was purified by column chromatography (silica gel, EtOAc) to give organotin **S-9** (89.8 mg, 204 µmol, 28%) as an amber liquid.

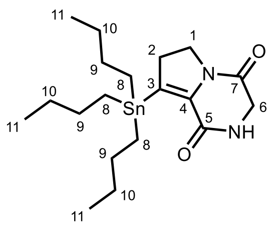

$\mathbf{R_f}$ = 0.37 (EtOAc), [UV, $KMnO_4$];

^{1}H NMR (600 MHz, $CDCl_3$): δ 6.46 (1H, br. s, N–H), 4.15 (2H, s, H-6), 3.99 (2H, t, J = 9.0 Hz, H-1), 2.84 (2H, app. t, J = 9.0 Hz, H-2), 1.56–1.44 (6H, m, H-9), 1.34–1.26 (6H, m, H-10), 1.08–0.93 (6H, m, H-8), 0.89 (9H, t, J = 7.3 Hz, H-11) ppm;

*Sn satellites observed at H-2, H-8 and H-9.

^{13}C NMR (151 MHz, $CDCl_3$): δ 159.1 (C-5 or C-7), 159.0 (C-5 or C-7), 138.9 (C-3), 135.7 (C-4), 47.0 (C-6), 46.4 (C-1), 34.7 (C-2), 29.3 (C-9), 27.5 (C-10), 13.9 (C-11), 10.9 (C-8) ppm;

*Sn satellites observed at C-8 (^{13}C–^{119}Sn J = 184 Hz, ^{13}C–^{117}Sn J = 176 Hz).

^{119}Sn NMR (149 MHz, $CDCl_3$): δ −53.67 ppm;

HRMS (ESI$^+$) calc for $C_{19}H_{34}N_2O_2SnNa$ ([M+Na]$^+$): 465.1538; found: 465.1551;

IR (film) ν_{max}/cm^{-1} 1680, 1613, 1452.

1.3.10. Experimental Procedure for Organotin 15

Organotin **15** was prepared according to a modified literature procedure.[10]

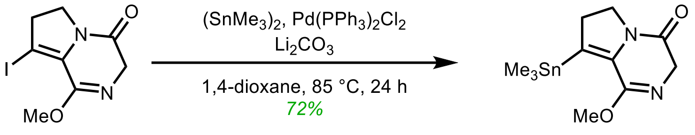

13 **15** [348 mg]

$Pd(PPh_3)_2Cl_2$ (25.4 mg, 2 mol%, 36.2 μmol) was added to a slurry of alkenyl iodide **13** (425 mg, 1.0 equiv., 1.46 mmol), Li_2CO_3 (266 mg, 2.5 equiv., 3.60 mmol), $(SnMe_3)_2$ (0.60 mL, 2.0 equiv., 2.89 mmol) in 1,4-dioxane (45 mL) under N_2. The reaction was stirred at 85 °C for 24 h before being passed through a small pad of celite and washed with EtOAc (600 mL), then concentrated under reduced pressure. The crude residue was purified by column chromatography (silica gel, EtOAc:*n*-hexane, 1:4) to give organotin **15** (348 mg, 1.05 mmol, 72%) as a colourless crystalline solid.

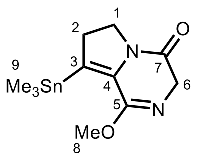

$\mathbf{R_f}$ = 0.41 (EtOAc), [UV, $KMnO_4$];

MP 93–95 °C (CH_2Cl_2);

^{1}H NMR (500 MHz, $CDCl_3$): δ 4.36 (2H, t, J = 1.6 Hz, H-6), 3.88 (2H, dd, J = 9.8, 8.3 Hz, H-1), 3.74 (3H, s, H-8), 2.79 (2H, app. tt, J = 8.2, 1.8 Hz, H-2), 0.19 (9H, s, H-9) ppm;

*Sn satellites observed at H-9 (^{1}H–C–^{115}Sn J = 63.3 Hz, ^{1}H–C–^{119}Sn J = 28.7 Hz, ^{1}H–C–^{117}Sn J = 27.3 Hz).

^{13}C NMR (126 MHz, $CDCl_3$): δ 163.7 (C-7), 153.8 (C-5), 134.1 (C-4), 127.8 (C-3), 53.3 (C-6), 52.7 (C-8), 44.7 (C-1), 34.6 (C-2), −8.2 (C-9) ppm;

*Sn satellites observed at C-9 (^{13}C–^{119}Sn J = 190 Hz, ^{13}C–^{117}Sn J = 182 Hz).

^{119}Sn NMR (149 MHz, $CDCl_3$): δ −39.96 ppm;

HRMS (ESI^+) calc for $C_{11}H_{19}N_2O_2Sn$ ($[M+H]^+$): 331.0463; found: 331.0461;

IR (film) ν_{max}/cm^{-1} 1679, 1595, 1433, 1324, 1061, 773.

Table S7 – Metallation conditions for alkenyl iodide **13**. All yields in this table are isolated yields. Ratios were obtained through NMR analysis of crude material.

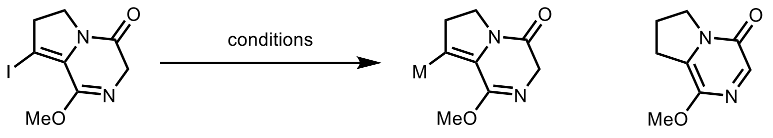

13 **18**

Entry	M	Metal source	Conditions	Result
1	Bpin	B_2pin_2	$Pd(dppf)Cl_2 \cdot CH_2Cl_2$ KOAc, 1,4-dioxane 80 °C, 22 h	starting material
2	Bpin	B_2pin_2	$Pd(OAc)_2$, SPhos KOAc, 1,4-dioxane 80 °C, 22 h	starting material
3	Bpin	B_2pin_2	$Pd(OAc)_2$, XantPhos KOAc, 1,4-dioxane 80 °C, 21 h	starting material
4	Bpin	B_2pin_2	$Pd(OAc)_2$, dppb KOAc, 1,4-dioxane 80 °C, 21 h	starting material
5	$SiMe_3$	$(SiMe_3)_2$	$Pd(PPh_3)_2Cl_2$ K_2CO_3, DMF 80 °C, 22 h	decomp.
6	$SiMe_2OH$	$(SiMe_2O)_3$	*i*-PrMgCl THF −20 °C to rt, 19 h	protodehalogenation
7	$SO_2(CH_2)_2CO_2Me$	$NaOSO(CH_2)_2CO_2Me$	CuI, DMEDA DMF 50 °C, 20 h	starting material
8	$GeMe_3$	$(GeMe_3)_2$	$Pd(PPh_3)_2Cl_2$ Cs_2CO_3 1,4-dioxane 85 °C, 25 h	starting material
9	$SnMe_3$	Me_3SnCl	*n*-BuLi in hexanes THF –78 °C to rt, 23 h	**18**
10	$SnMe_3$	Me_3SnCl	*n*-BuLi in hexanes TMEDA, THF –78 °C to rt, 21 h	**18**
11	$SnMe_3$	Me_3SnCl	*n*-BuLi in hexanes PMDTA, THF –78 °C to rt, 22 h	**18**
12	$SnMe_3$	Me_3SnCl	*i*-PrMgCl THF rt, 4 h	protodehalogenation starting material product (1.0:3.9:1.9)
13	$SnMe_3$	$(SnMe_3)_2$	$Pd(PPh_3)_2Cl_2$ Li_2CO_3 1,4-dioxane 85 °C, 24 h	72%

1.3.11. Experimental Procedure for Mono-Adduct 21

Mono-adduct **21** was prepared according to a modified literature procedure.[13]

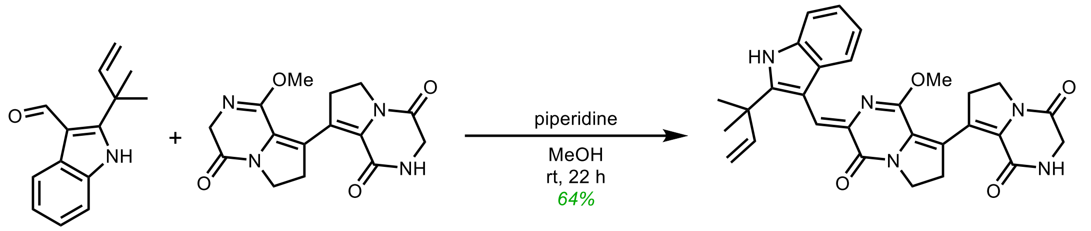

3 **14** **21** [48 mg]

Piperidine (0.05 mL, 3.7 equiv., 531 μmol) was added to a solution of aldehyde **3** (78.0 mg, 2.5 equiv., 366 μmol) and bis-diketopiperazine **14** (45.8 mg, 1.0 equiv., 145 μmol) in MeOH (5 mL). This solution was stirred at room temperature for 22 h under N_2 before being concentrated under reduced pressure at room temperature. The crude residue was purified by column chromatography (silica gel, CH_2Cl_2:MeOH, 20:1) to give mono-adduct **21** (48 mg, 93.2 μmol, 64%) as a orange solid.

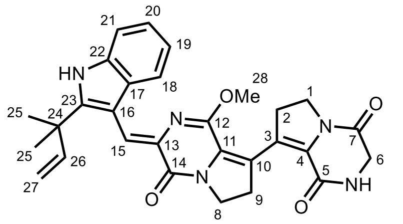

R$_f$ = 0.09 (CH_2Cl_2:MeOH, 10:1), [amber with no stain, UV, $KMnO_4$, PMA, vanillin];

MP 210 °C (decomp.) ($CHCl_3$);

^{1}H NMR (500 MHz, CD_3OD): δ 7.88 (1H, dt, *J* = 8.0 , 1.1 Hz, H-18), 7.79 (1H, s, H-15), 7.36 (1H, dt, *J* = 8.0, 0.9 Hz, H-21), 7.06 (1H, ddd, *J* = 8.1, 7.0, 1.2 Hz, H-20), 6.99 (1H, ddd, *J* = 8.1, 7.0, 1.1 Hz, H-19), 6.14 (1H, dd, *J* = 17.4, 10.6 Hz, H-26), 5.19–5.12 (2H, m, H-27), 4.07 (2H, s, H-6), 4.00 (2H, dd, *J* = 9.9, 8.4 Hz, H-8), 3.82 (2H, app. t, *J* = 9.3 Hz, H-1), 3.67 (3H, s, H-28), 2.99 (2H, dd, *J* = 9.9, 8.4 Hz, H-9), 2.84 (2H, app. t, *J* = 9.1 Hz, H-2), 1.57 (6H, s, H-25) ppm;

^{13}C NMR (126 MHz, CD_3OD): δ 162.8 (C-5 or C-7), 161.2 (C-14), 159.4 (C-5 or C-7), 151.7 (C-12), 148.0 (C-23), 146.5 (C-26), 136.8 (C-22), 129.8 (C-13), 128.7 (C-3 and C-17), 128.3 (C-4), 128.1 (C-11), 126.5 (C-15), 124.9 (C-18), 122.4 (C-20), 120.04 (C-19), 119.95 (C-10), 112.7 (C-27), 112.0 (C-21), 109.5 (C-16), 54.4 (C-28), 47.0 (C-6), 45.1 (C-8), 44.8 (C-1), 40.6 (C-24), 32.8 (C-2), 32.6 (C-9), 28.7 (C-25) ppm;

HRMS (ESI^+) calc for $C_{29}H_{30}N_5O_4$ ($[M+H]^+$): 512.2292; found: 512.2280;

IR (film) ν_{max}/cm^{-1} 3271, 1678, 1651, 1584, 1570, 1453, 1420, 1398.

2. NMR Spectra

2.1. ^{1}H NMR Spectrum for phthalylglycyl chloride (600 MHz, $CDCl_3$)

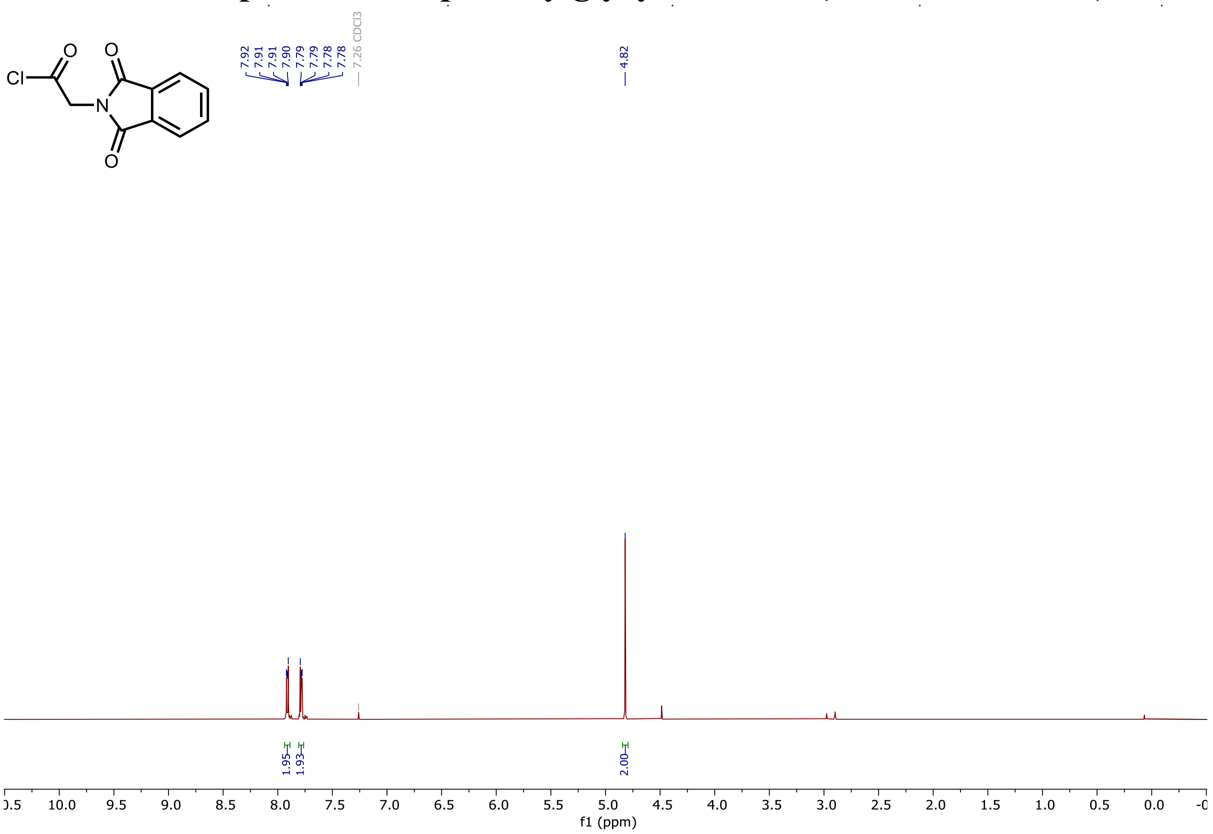

2.2. ^{13}C NMR Spectrum for phthalylglycyl chloride (126 MHz, $CDCl_3$)

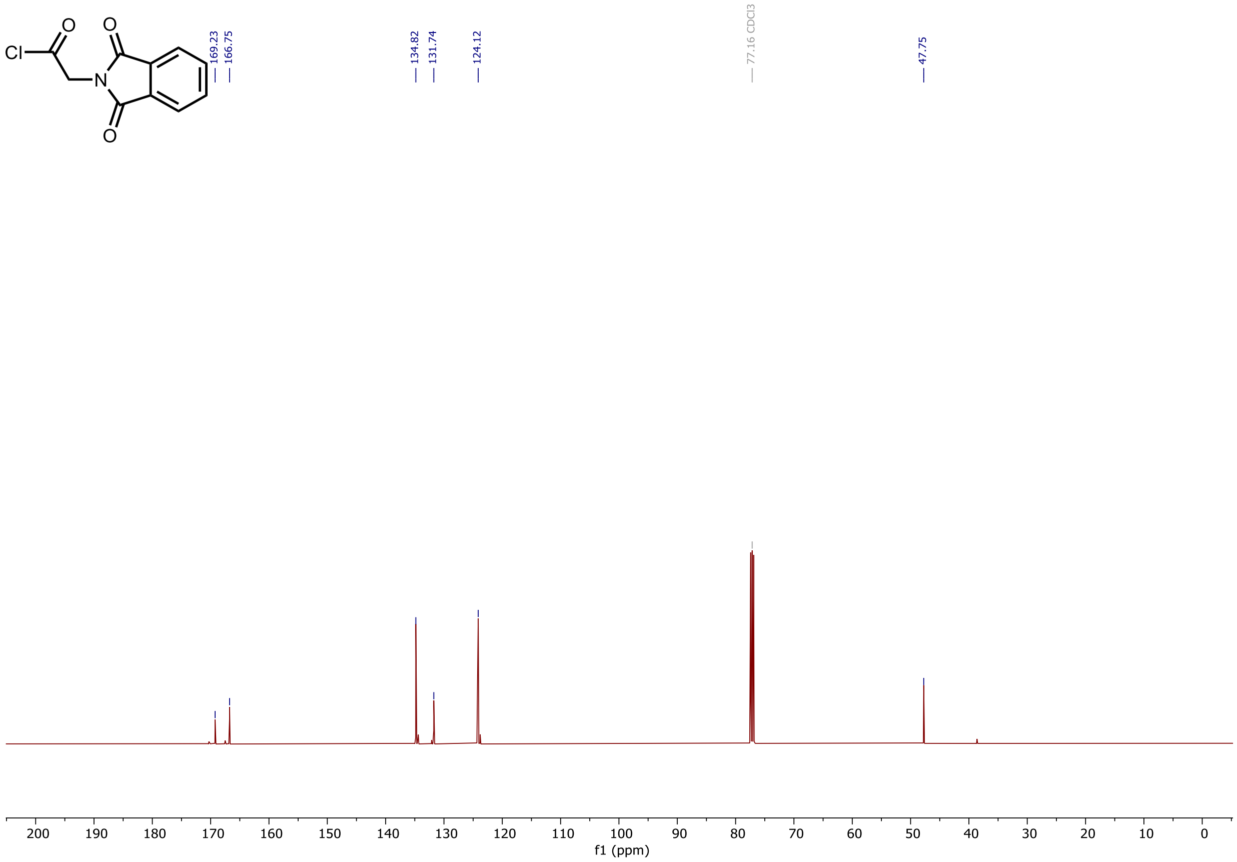

2.3. ^{13}C DEPT-135 Spectrum for phthalylglycyl chloride ($CDCl_3$)

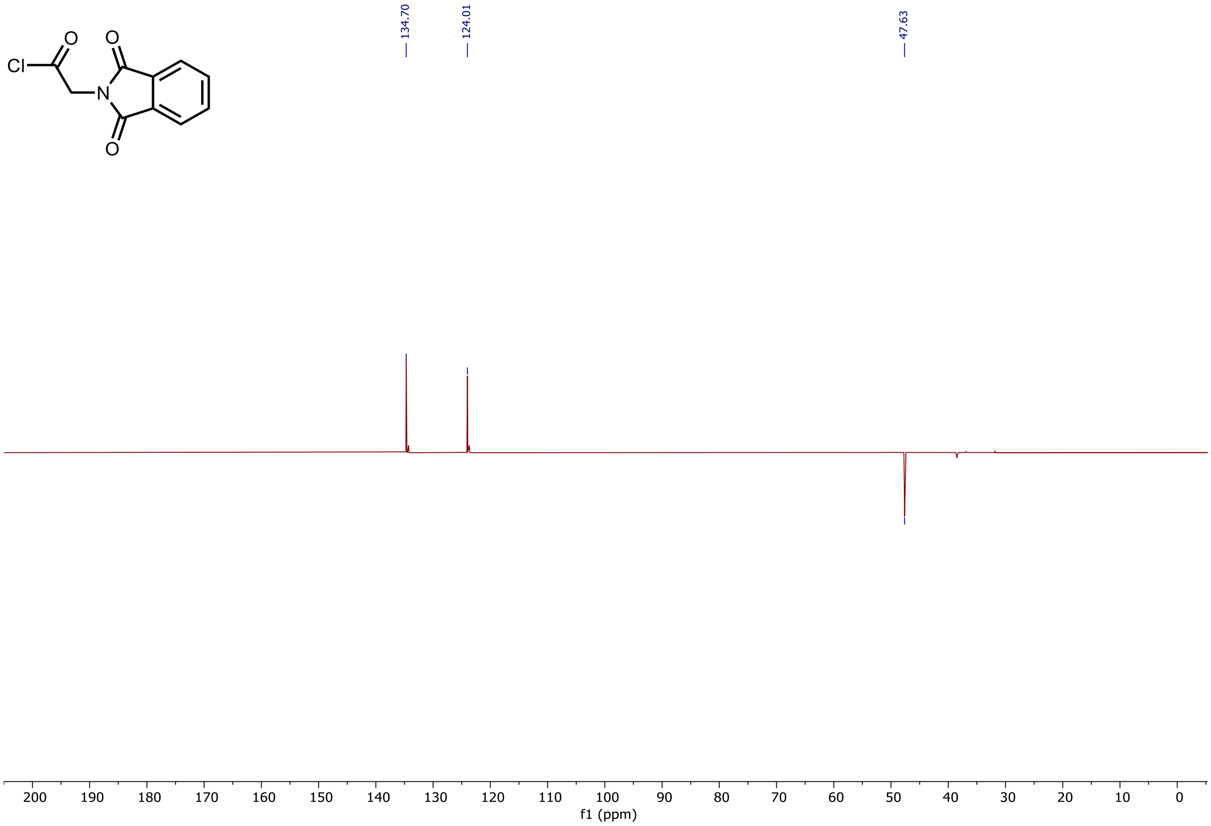

2.4. ^{1}H-^{1}H COSY Spectrum for phthalylglycyl chloride ($CDCl_3$)

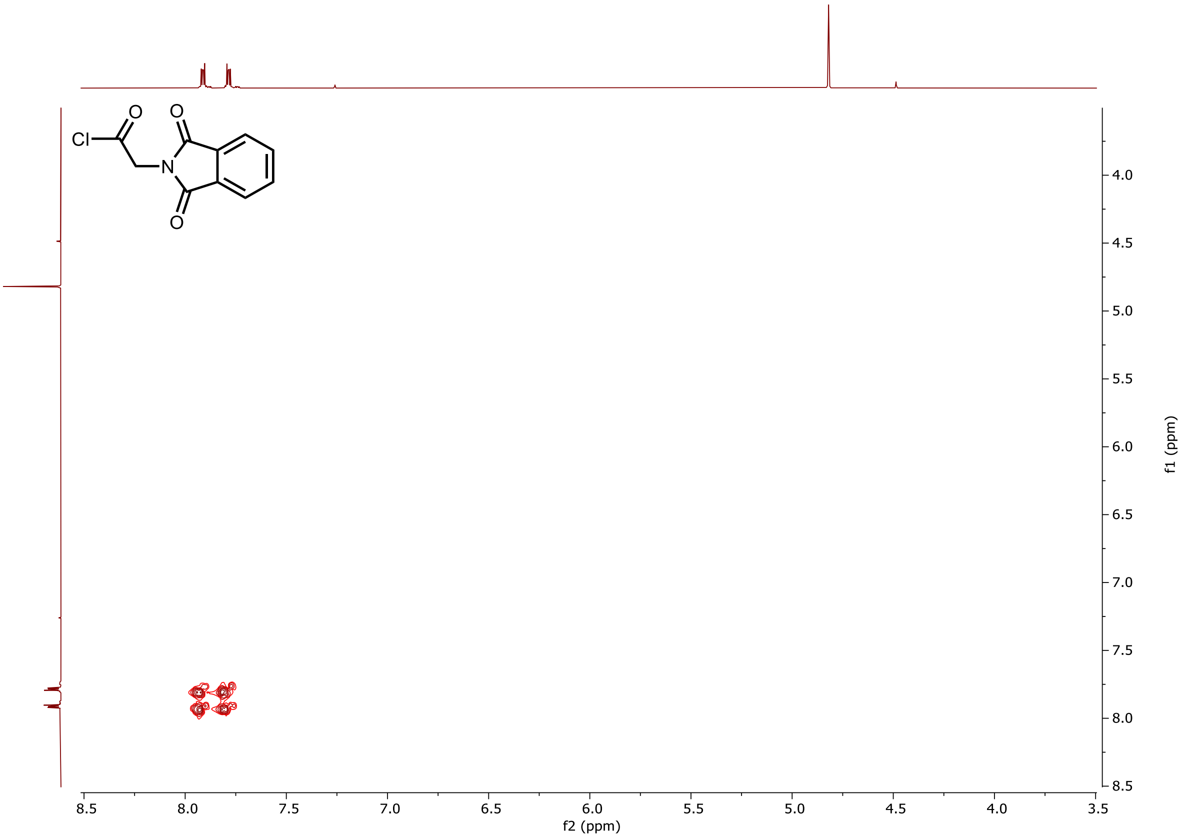

2.5. ^{1}H-^{13}C HSQC Spectrum for phthalylglycyl chloride ($CDCl_3$)

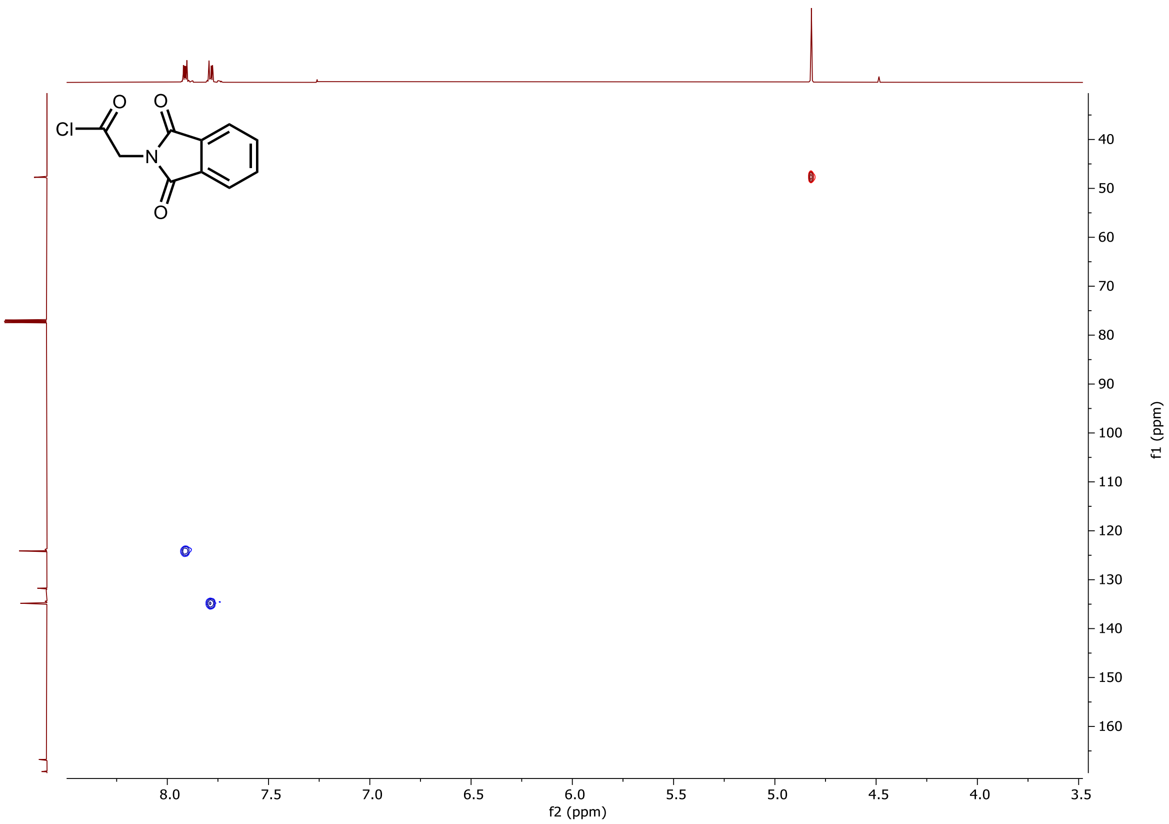

2.6. ^{1}H-^{13}C HMBC Spectrum for phthalylglycyl chloride ($CDCl_3$)

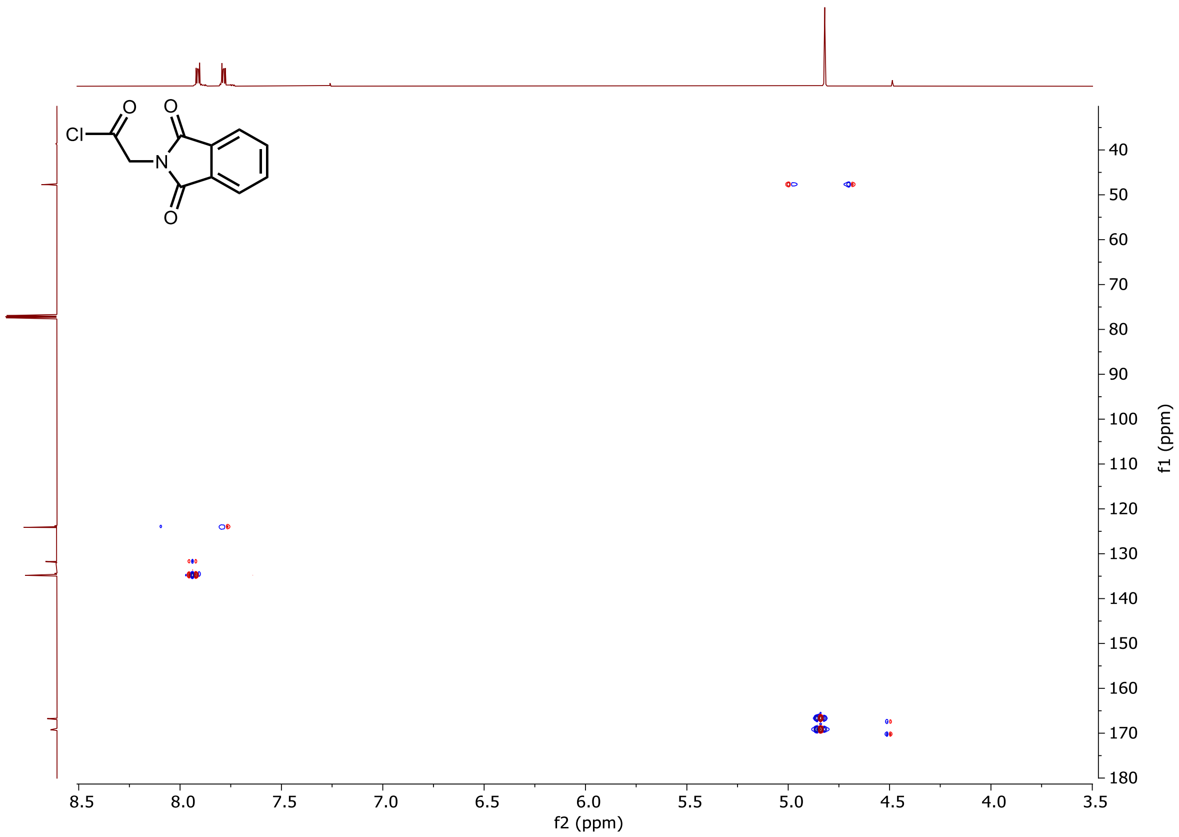

2.7. ^{1}H NMR Spectrum for Enamide 8 (500 MHz, $CDCl_3$)

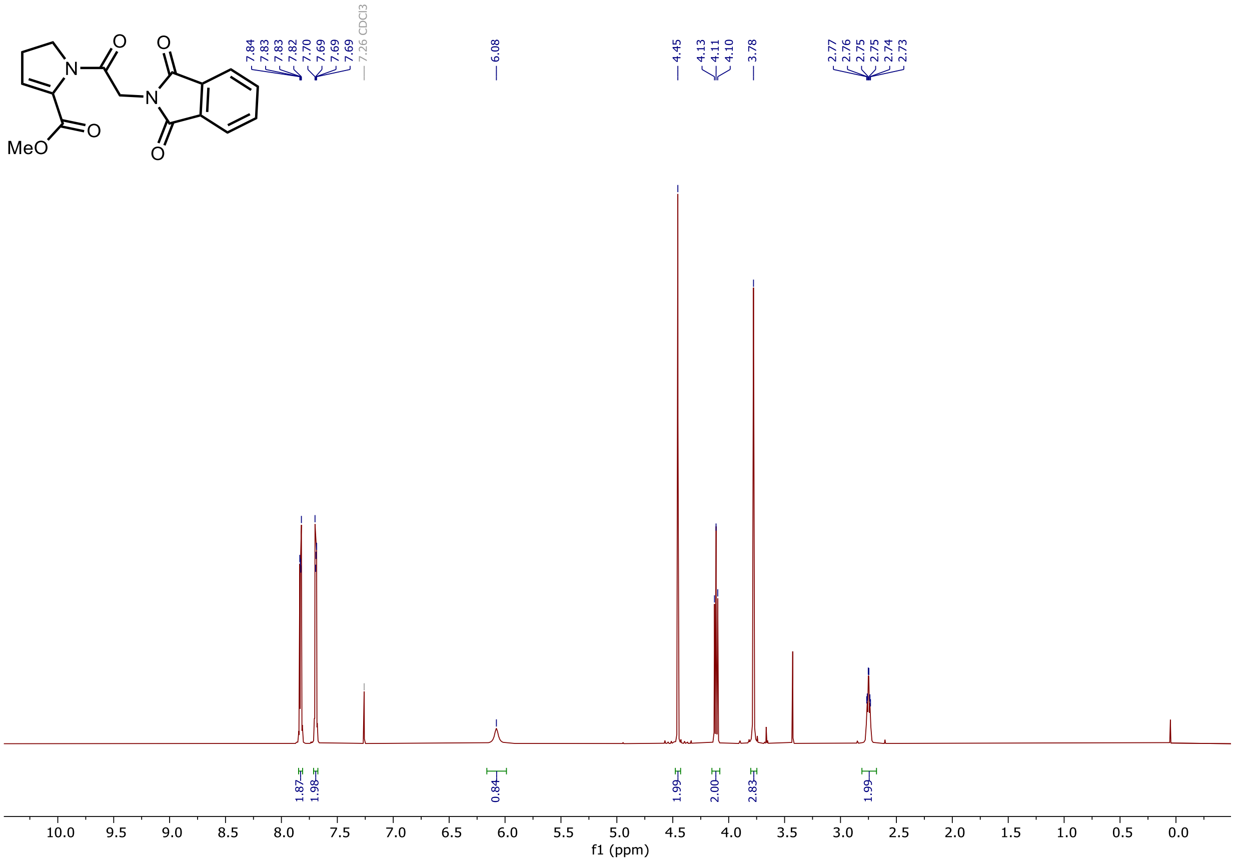

2.8. ^{13}C NMR Spectrum for Enamide 8 (126 MHz, $CDCl_3$)

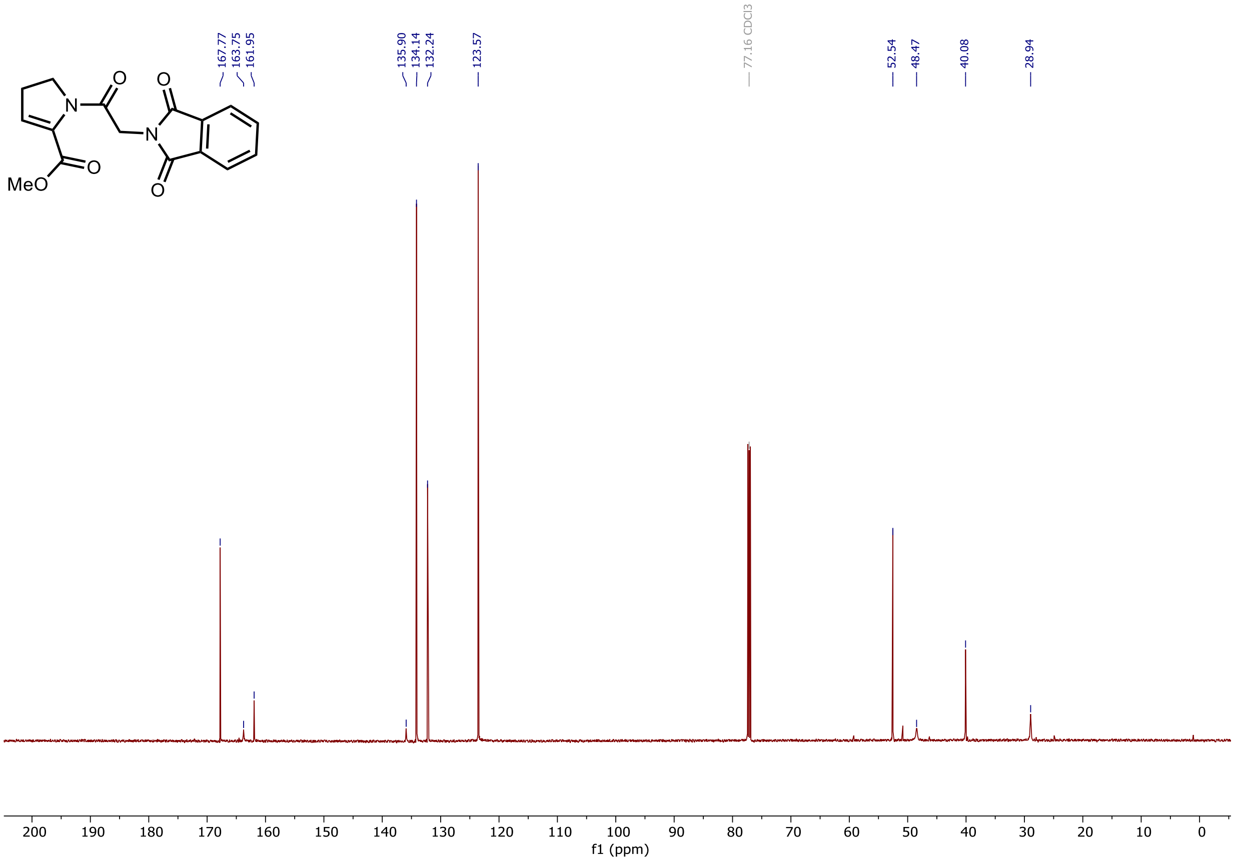

2.9. ^{1}H-^{1}H COSY Spectrum for Enamide 8 ($CDCl_3$)

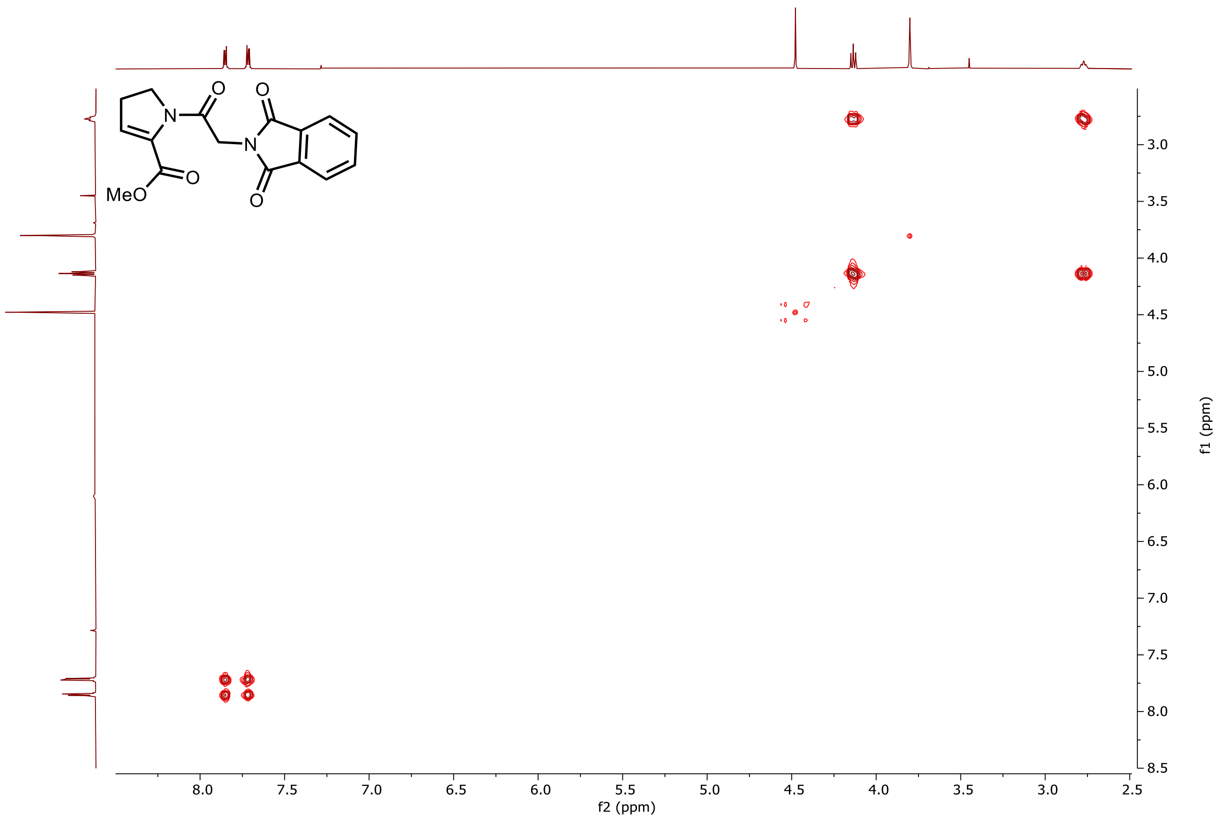

2.10. ^{1}H-^{13}C HSQC Spectrum for Enamide 8 ($CDCl_3$)

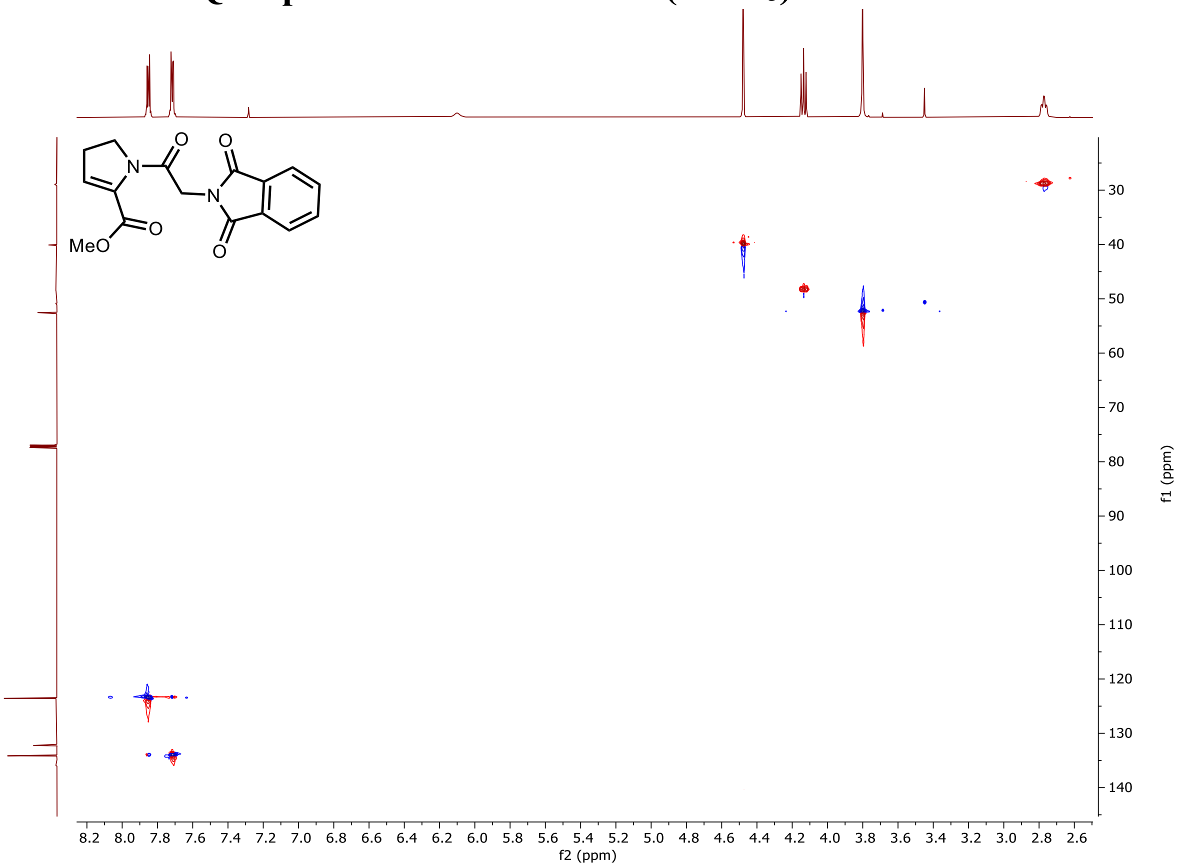

2.11. ^{1}H-^{13}C HMBC Spectrum for Enamide 8 ($CDCl_3$)

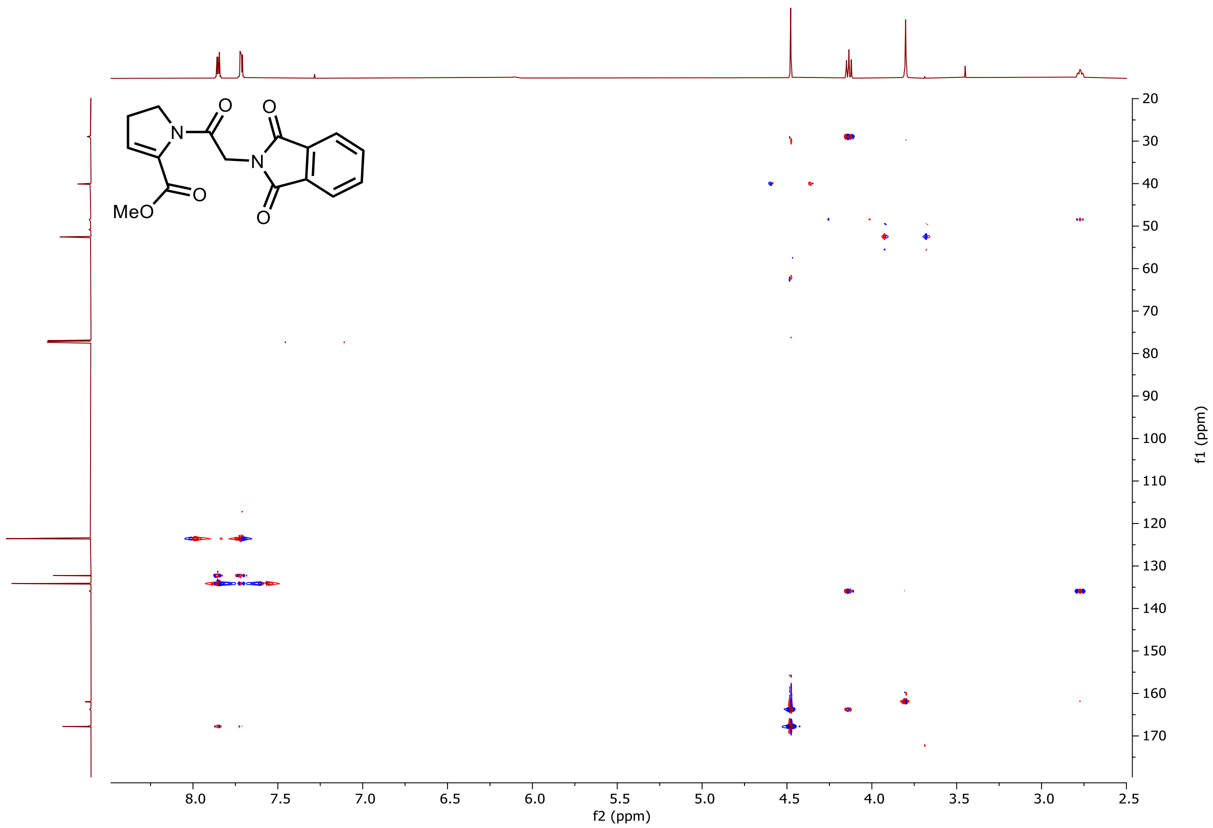

2.12. ^{1}H NMR Spectrum for Enamide 8 (500 MHz, $(CD_3)_2SO$)

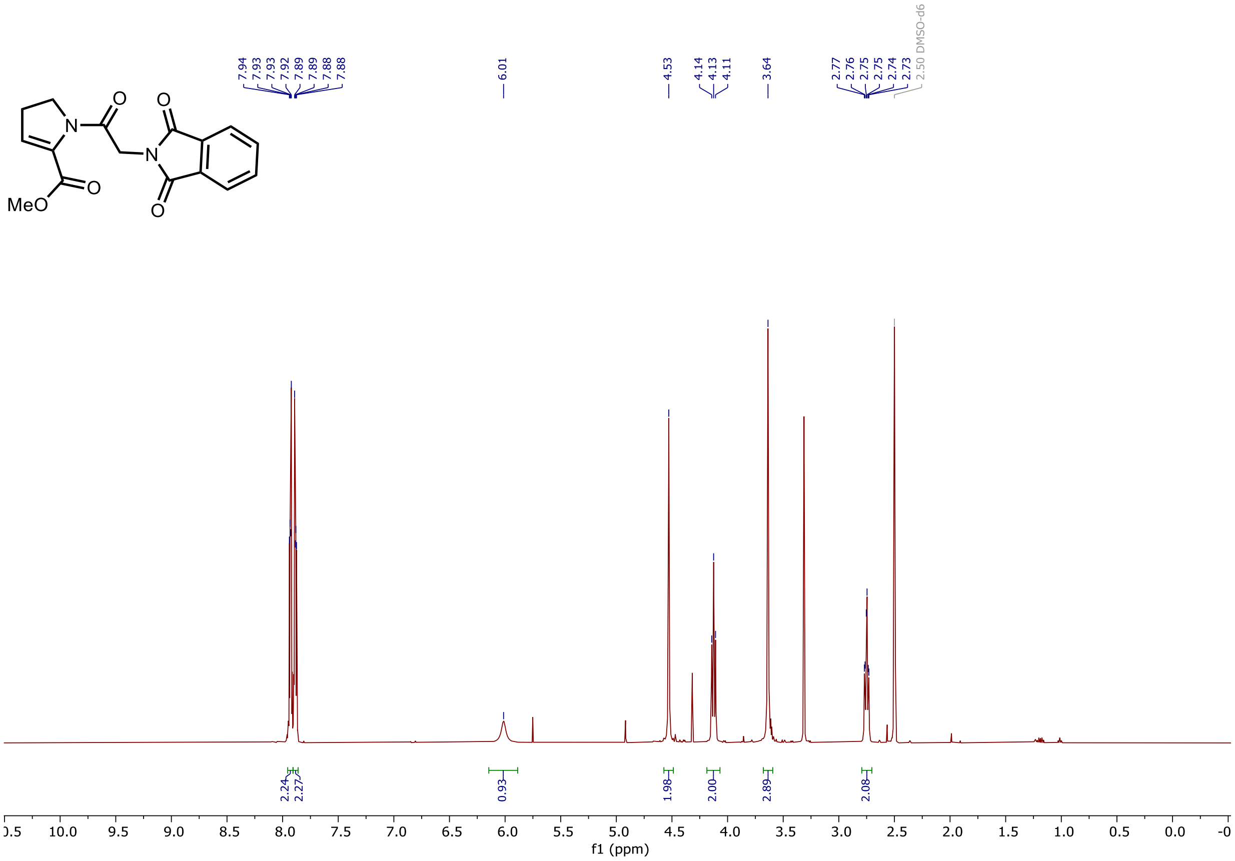

2.13. ^13C NMR Spectrum for Enamide 8 (126 MHz, $(CD_3)_2SO$)

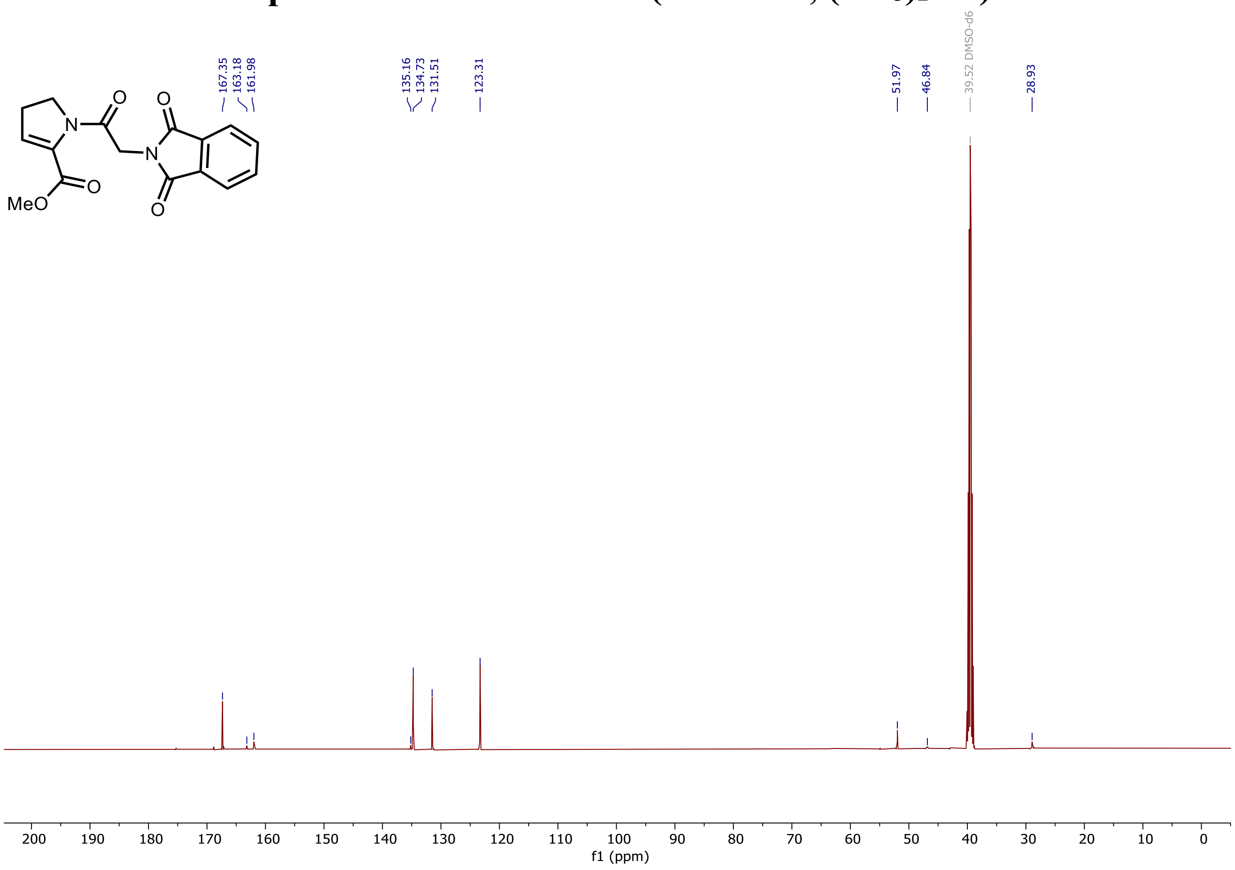

2.14. ^1H-^1H COSY Spectrum for Enamide 8 ($(CD_3)_2SO$)

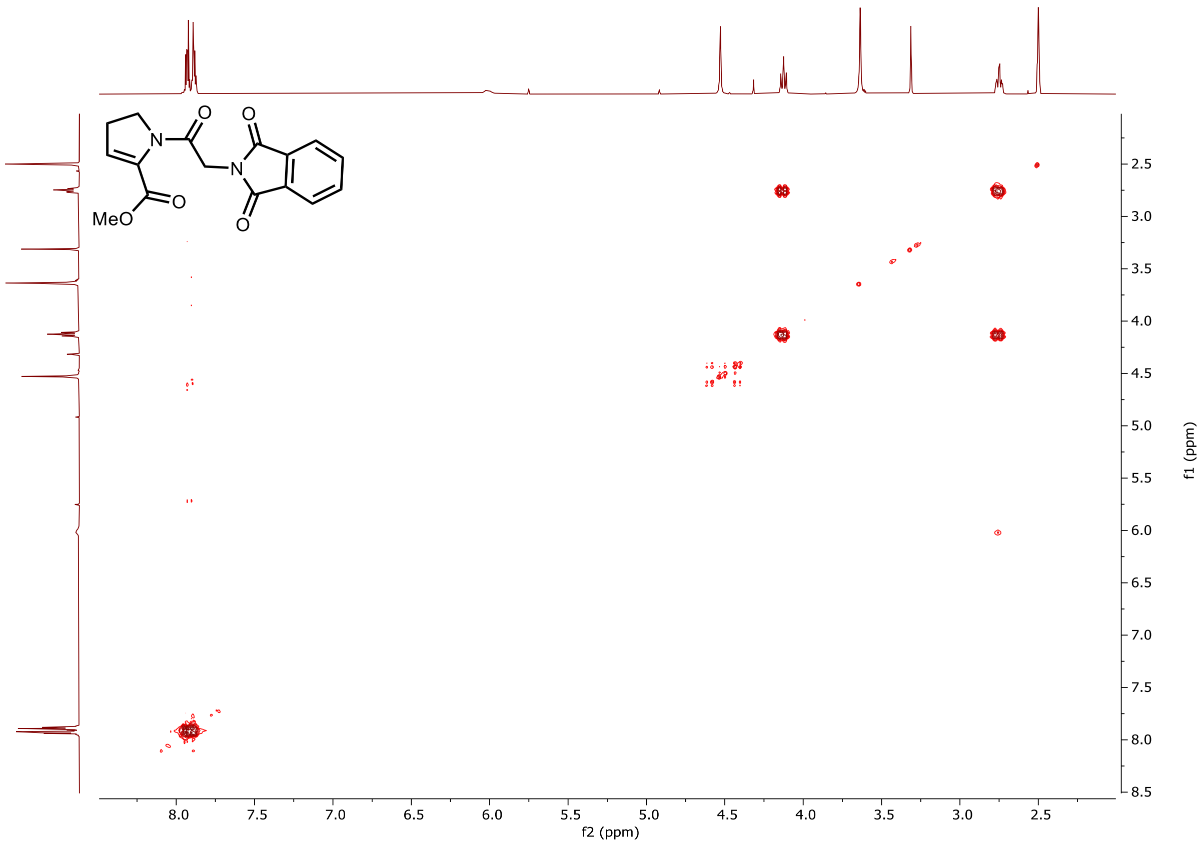

2.15. ^{1}H-^{13}C HSQC Spectrum for Enamide 8 ((CD$_3$)$_2$SO)

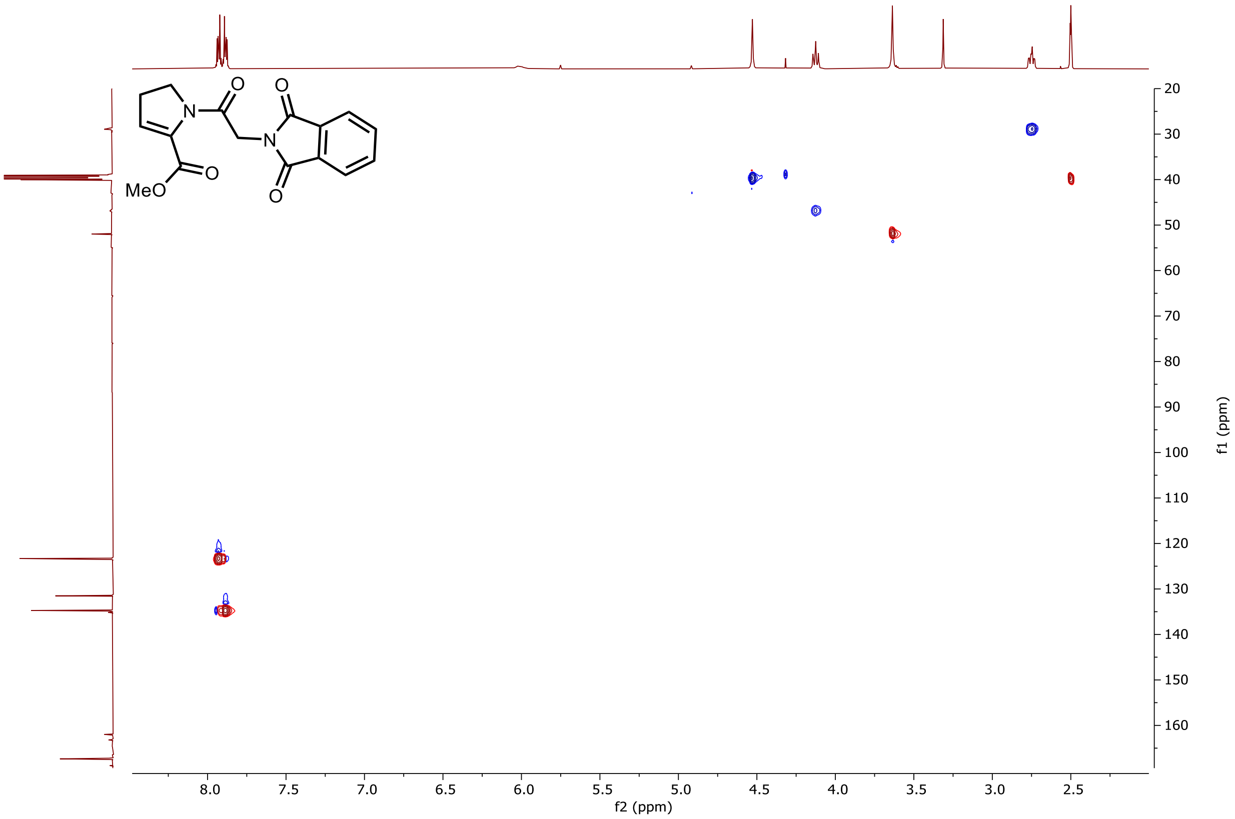

2.16. ^{1}H-^{13}C HMBC Spectrum for Enamide 8 ((CD$_3$)$_2$SO)

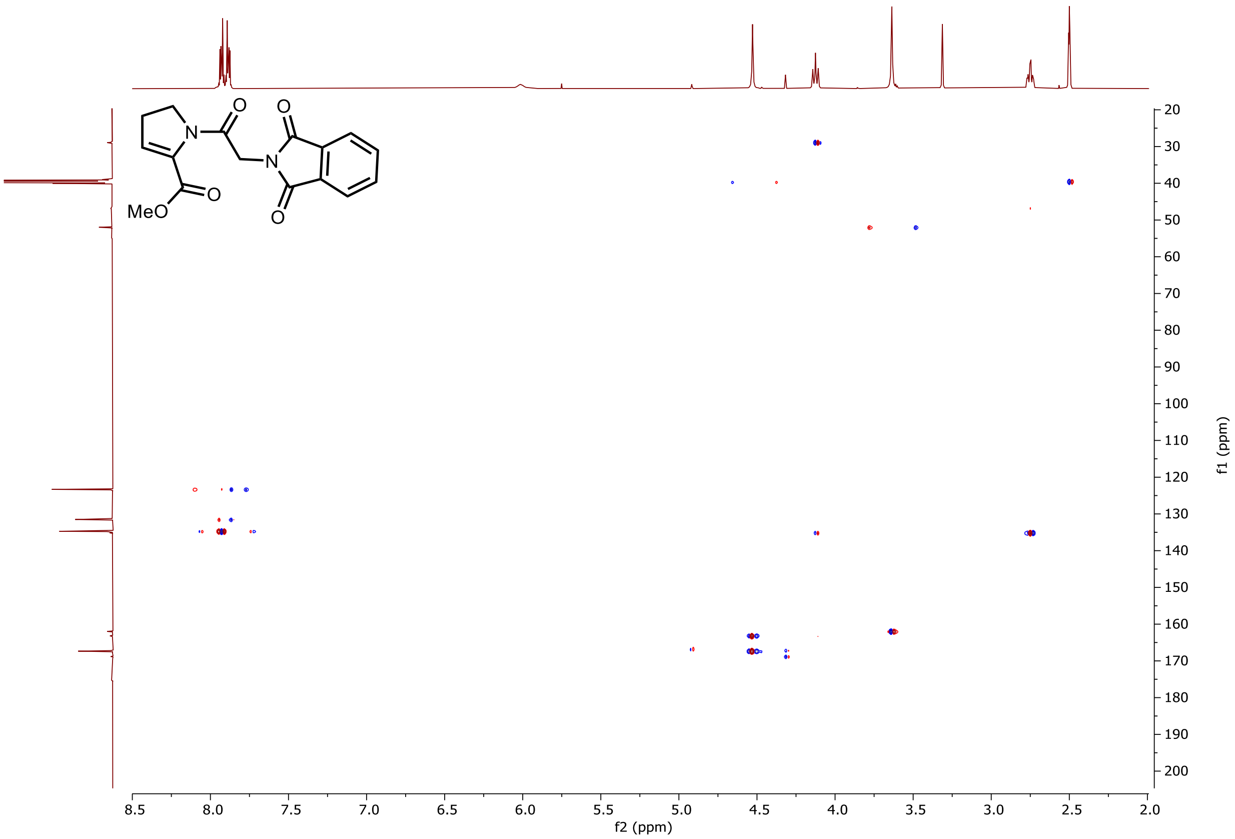

2.17. ^{1}H NMR Spectrum for Enamide 8 (500 MHz, $(CD_3)_2SO$, 343 K)

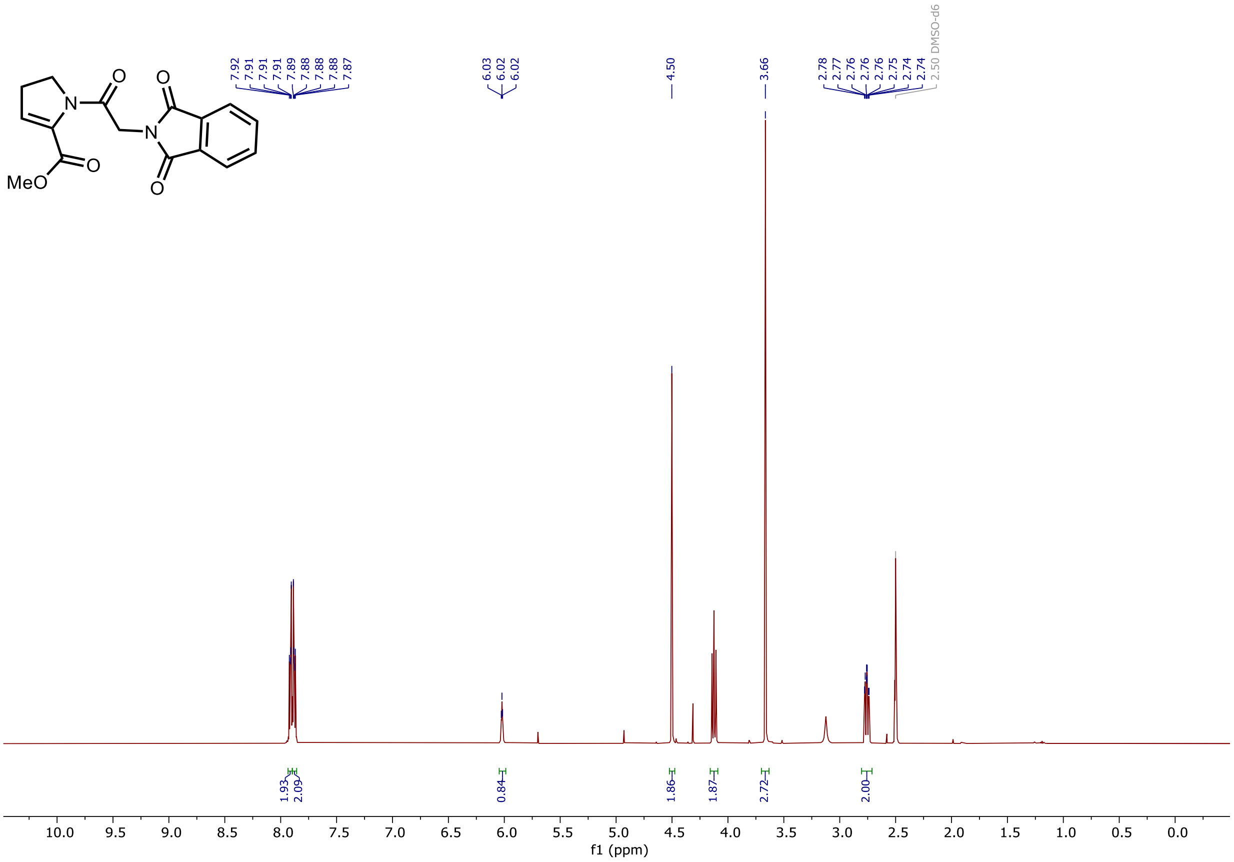

2.18. ^{1}H-^{13}C HSQC Spectrum for Enamide 8 ($(CD_3)_2SO$, 343 K)

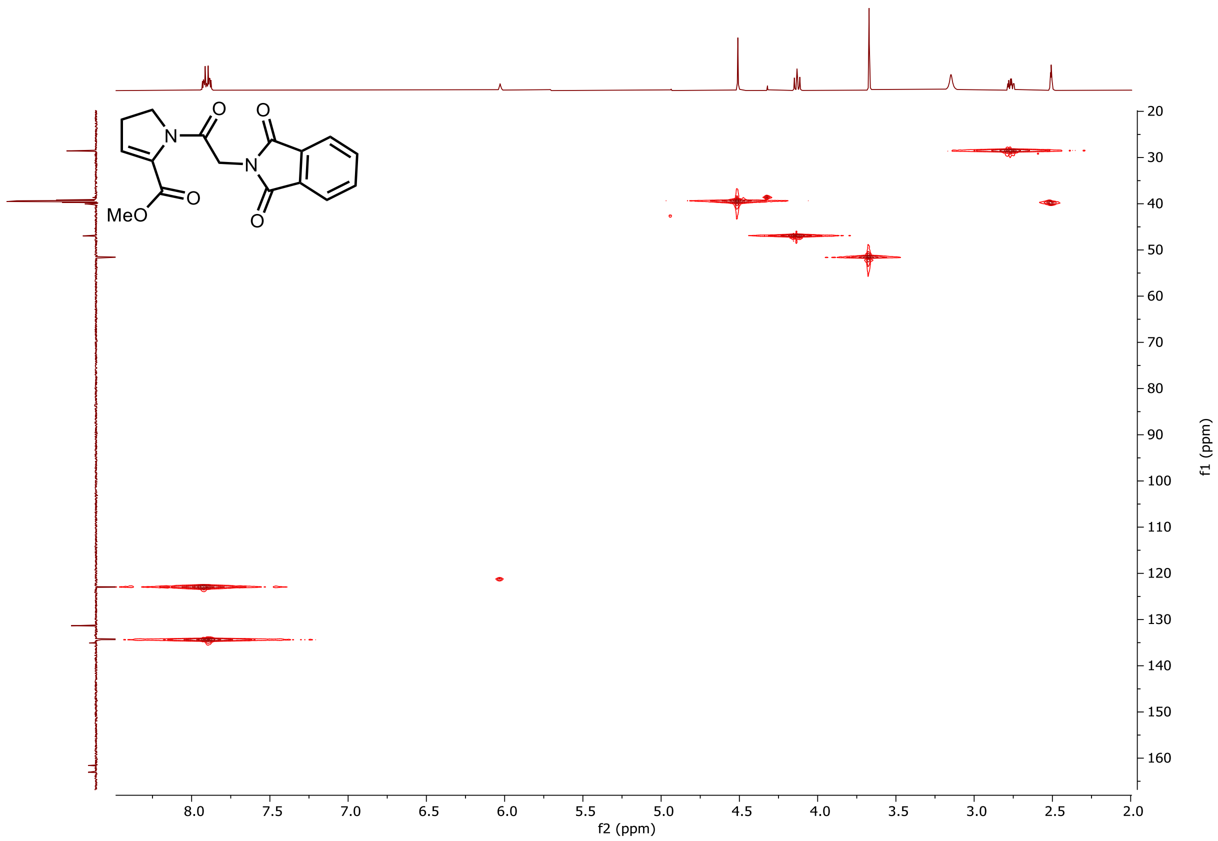

2.19. ^{1}H-^{13}C HMBC Spectrum for Enamide 8 ((CD_3)$_2$SO, 343 K)

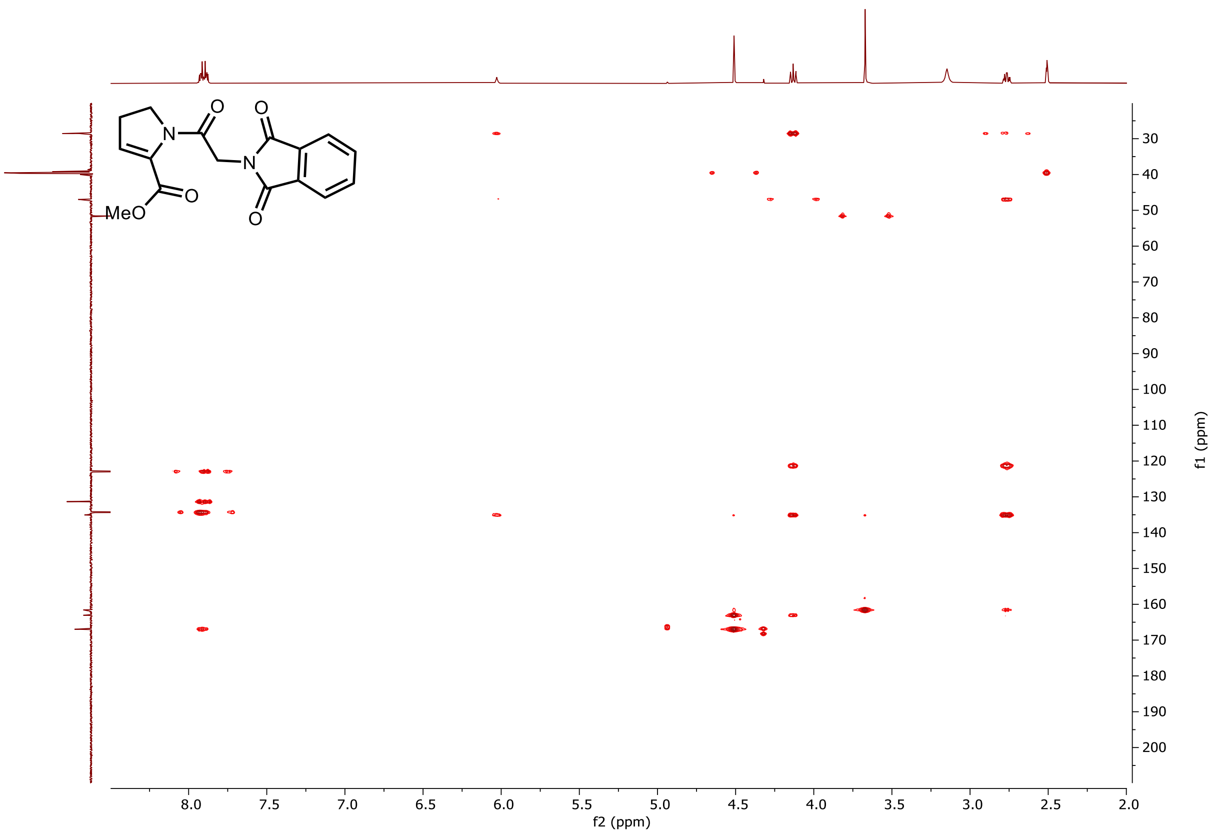

2.20. ^{1}H NMR Spectrum for Diketopiperazine 10 (600 MHz, $CDCl_3$)

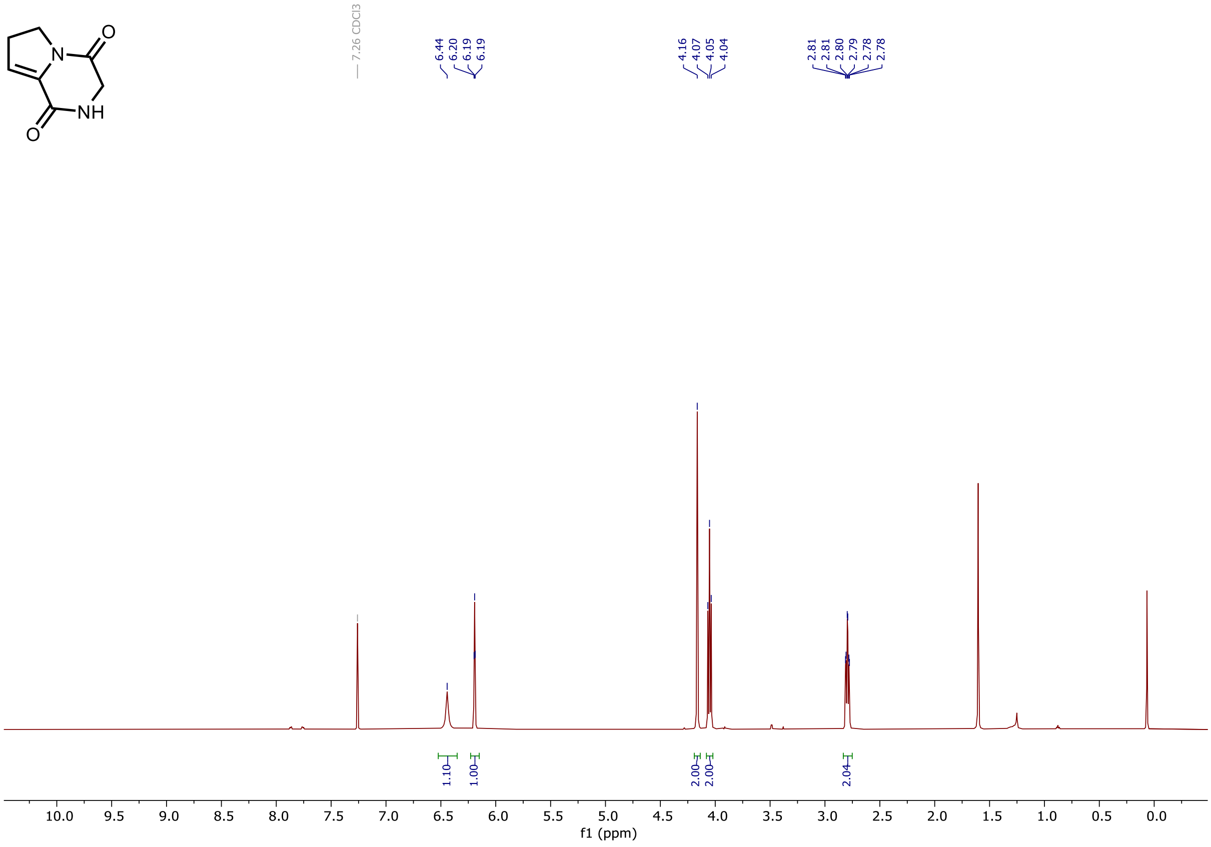

2.21. ^{13}C NMR Spectrum for Diketopiperazine 10 (151 MHz, $CDCl_3$)

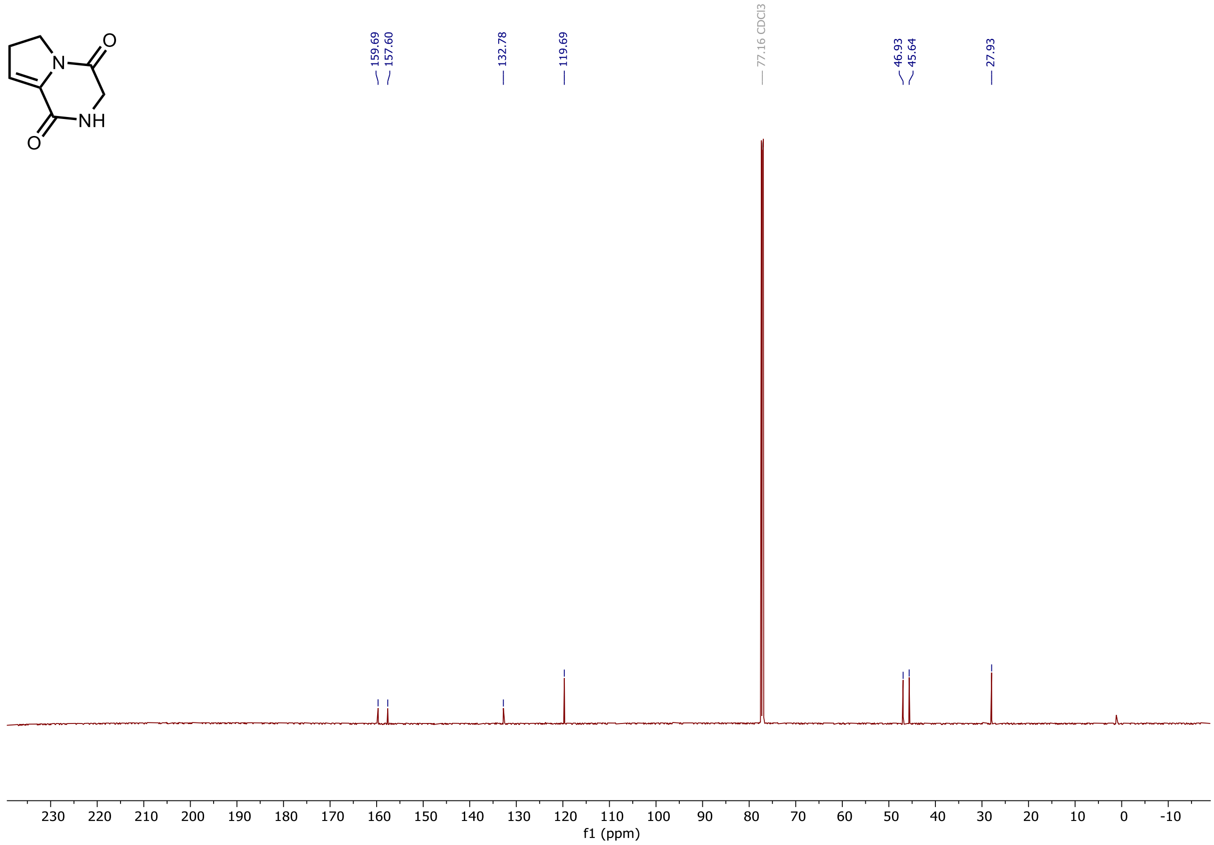

2.22. ^{13}C DEPT-135 Spectrum for Diketopiperazine 10 ($CDCl_3$)

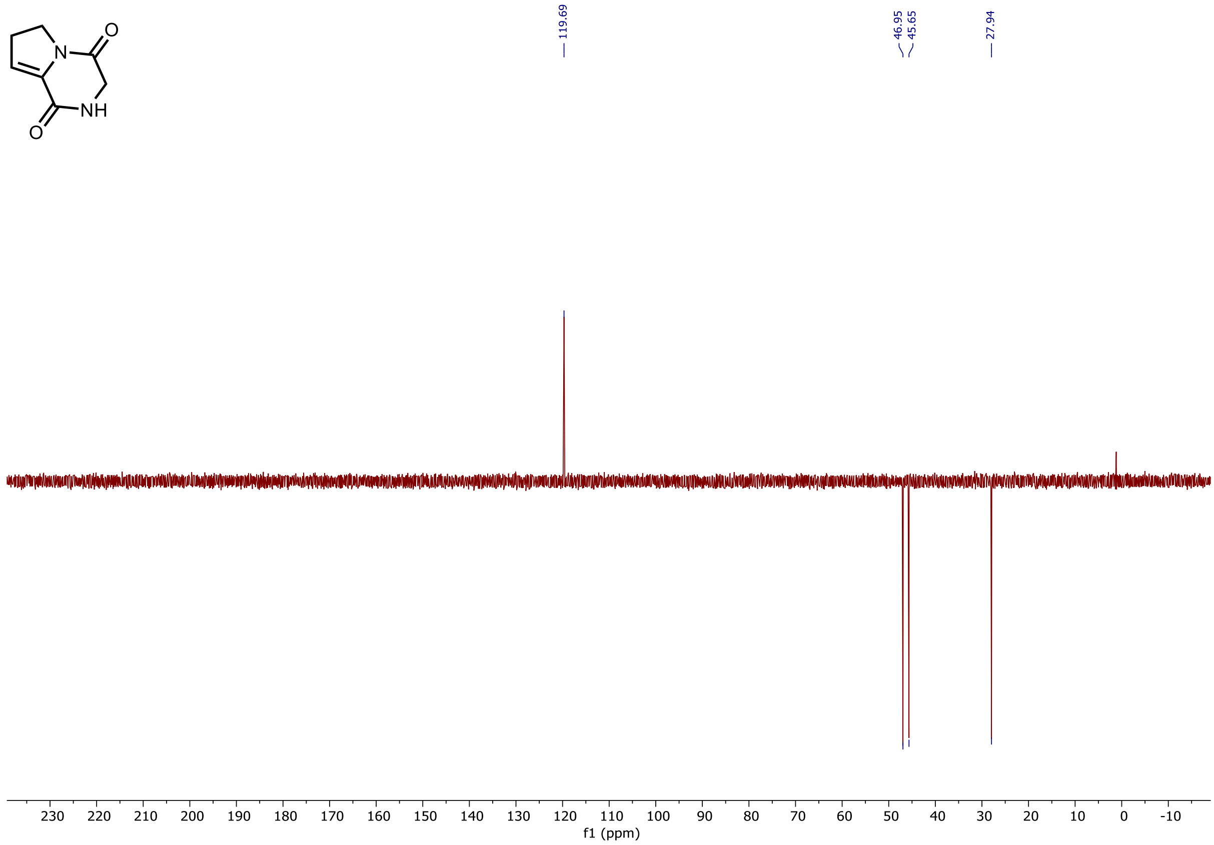

2.23. ^{1}H-^{1}H COSY Spectrum for Diketopiperazine 10 ($CDCl_3$)

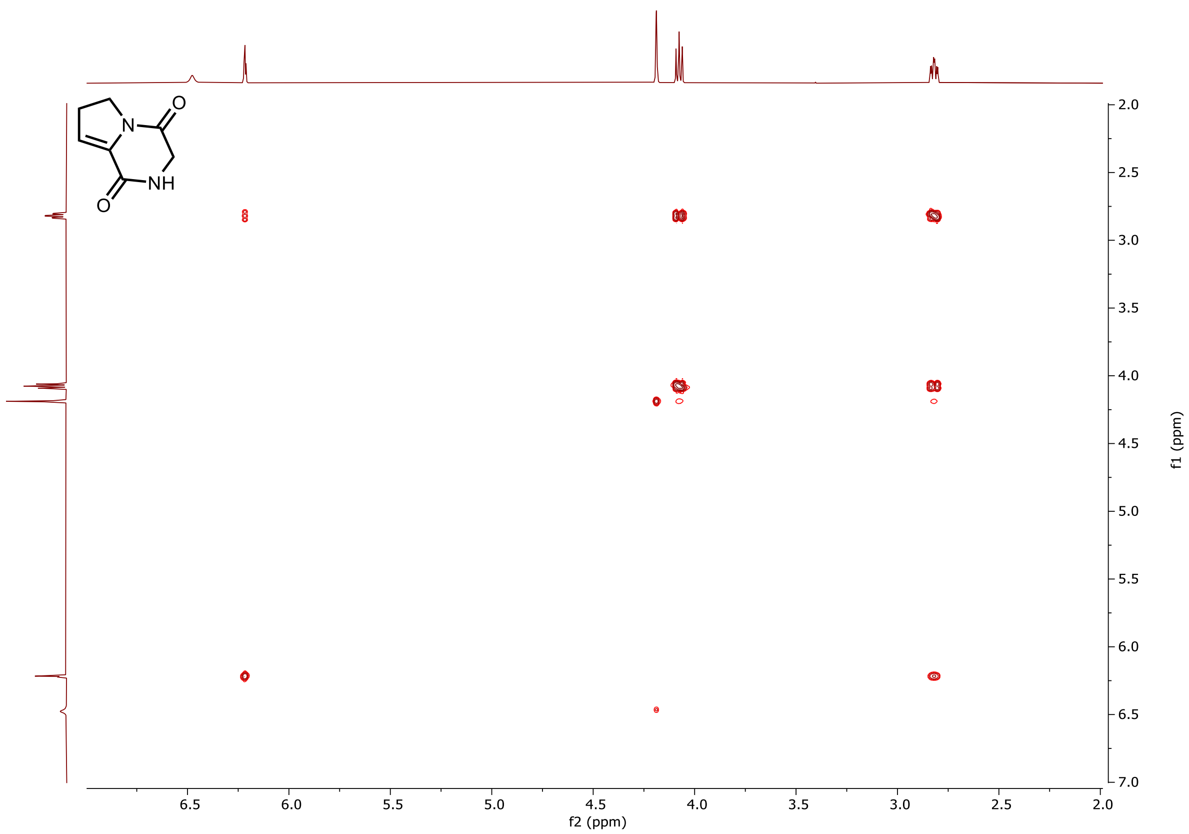

2.24. ^{1}H-^{13}C HSQC Spectrum for Diketopiperazine 10 ($CDCl_3$)

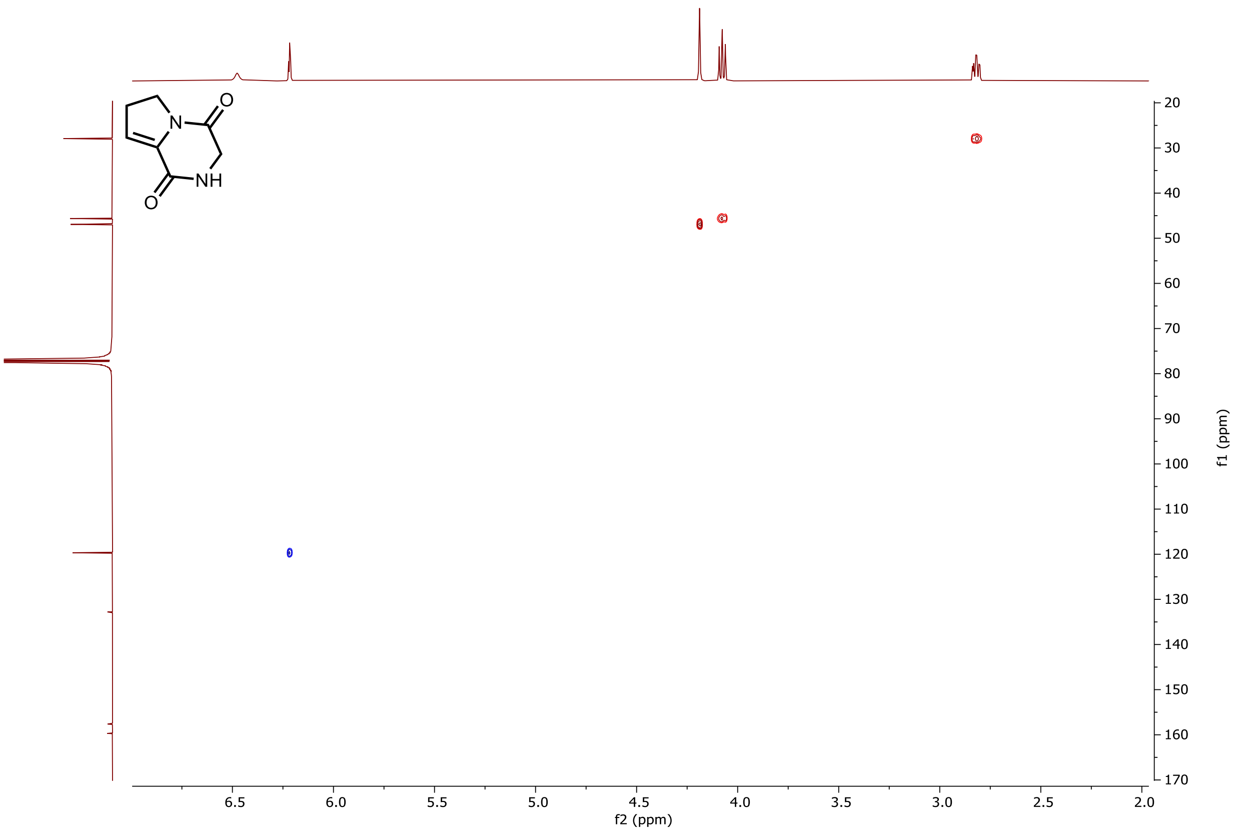

2.25. ^{1}H-^{13}C HMBC Spectrum for Diketopiperazine 10 ($CDCl_3$)

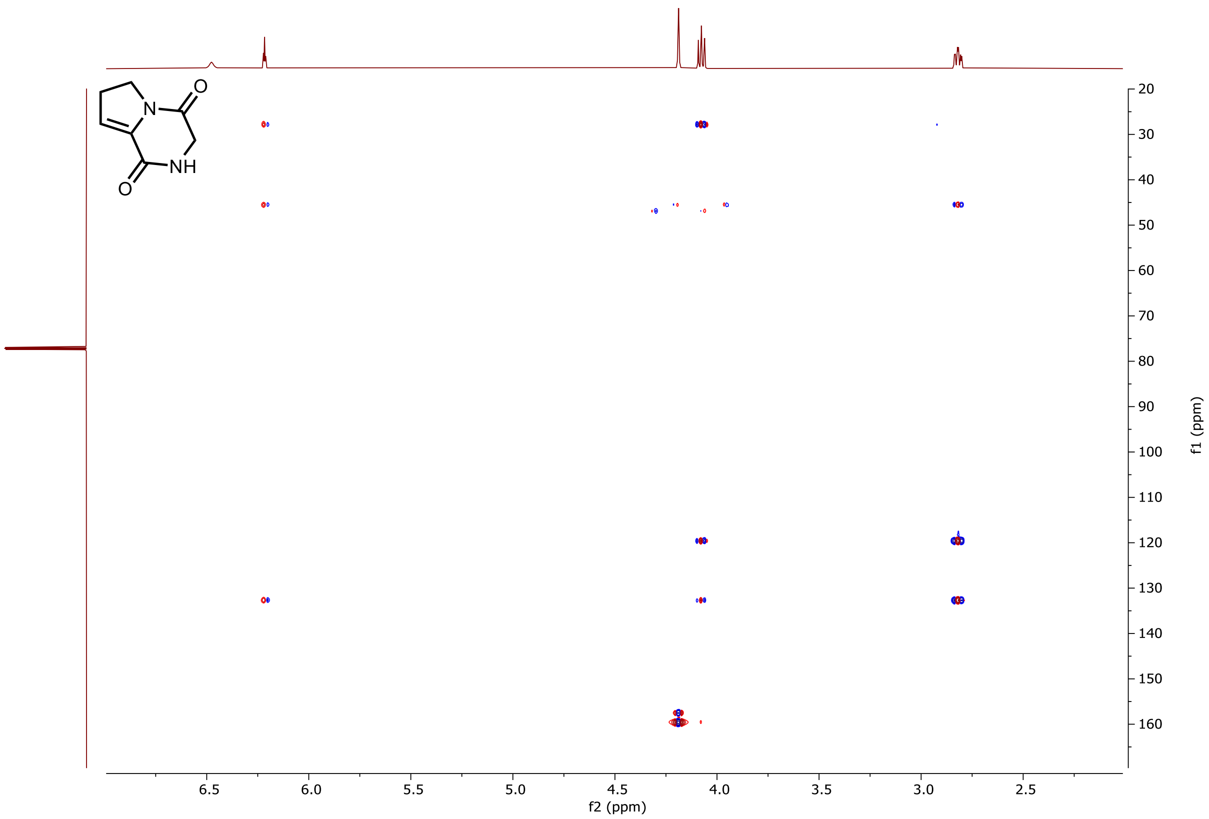

2.26. ^{1}H NMR Spectrum for Alkenyl Iodide 11 (600 MHz, $CDCl_3$)

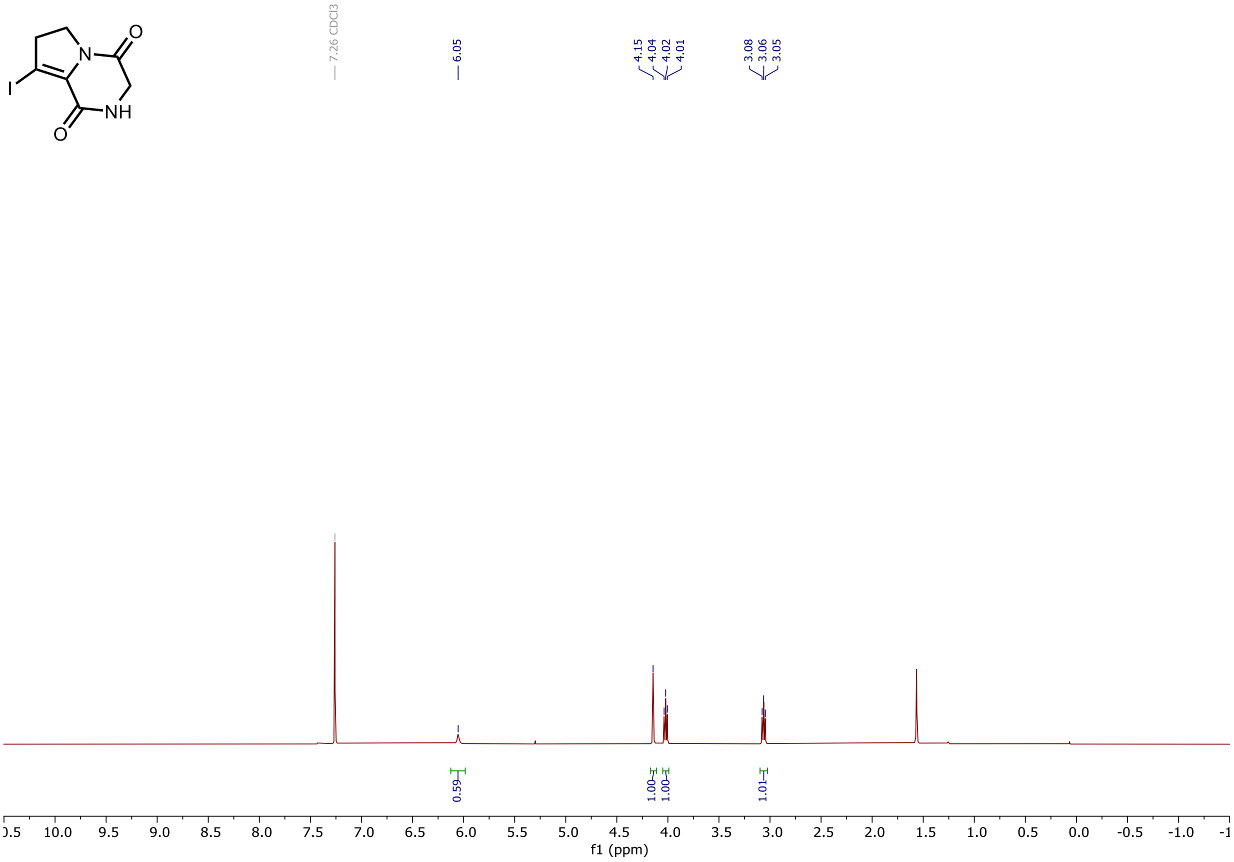

2.27. ^{13}C NMR Spectrum for Alkenyl Iodide 11 (126 MHz, $CDCl_3$)

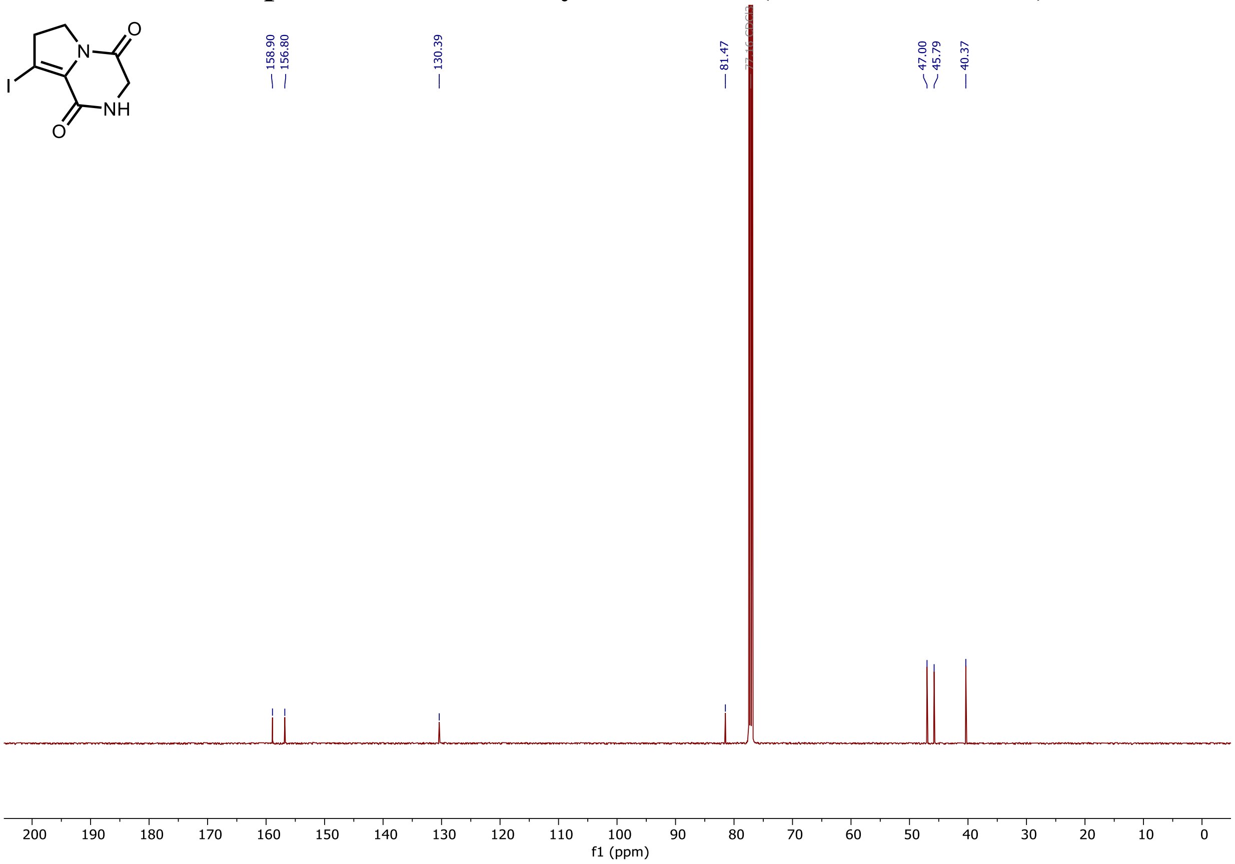

2.28. ^{1}H-^{1}H COSY Spectrum for Alkenyl Iodide 11 ($CDCl_3$)

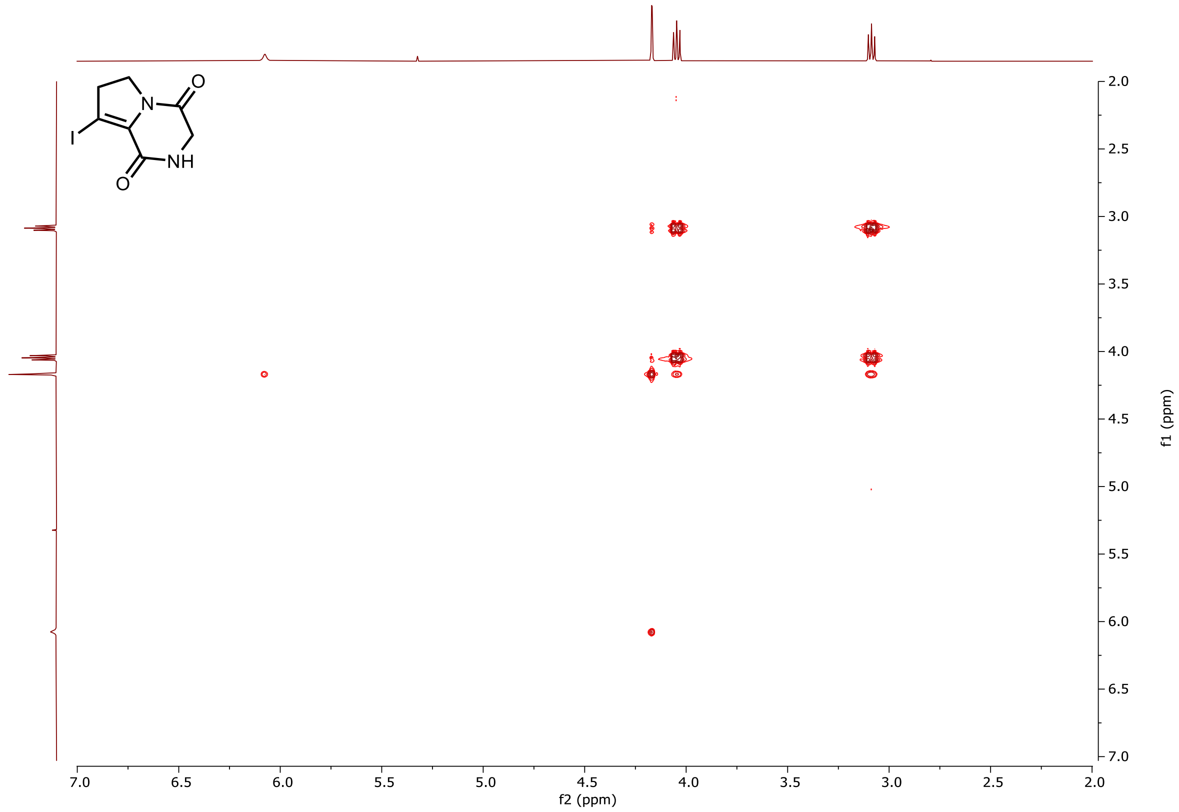

2.29. ^{1}H-^{13}C HSQC Spectrum for Alkenyl Iodide 11 ($CDCl_3$)

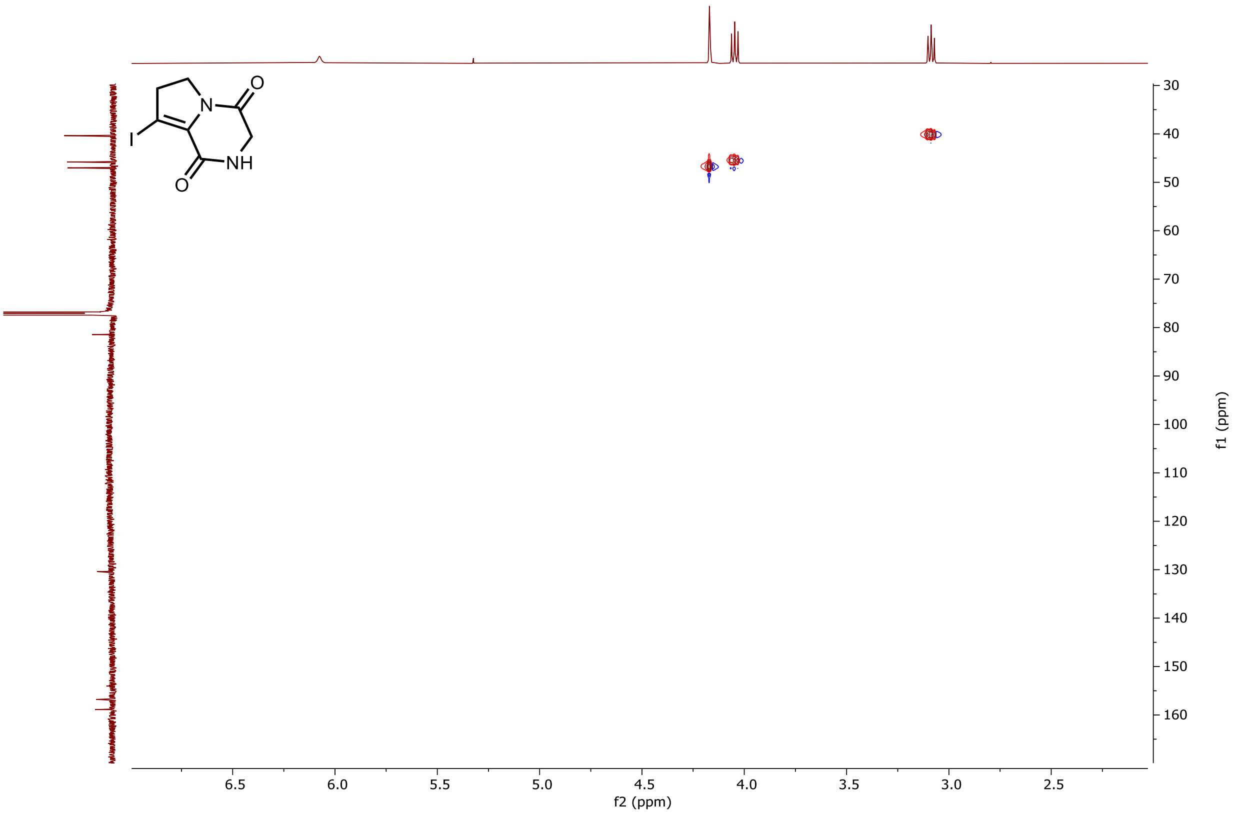

2.30. ^{1}H-^{13}C HMBC Spectrum for Alkenyl Iodide 11 ($CDCl_3$)

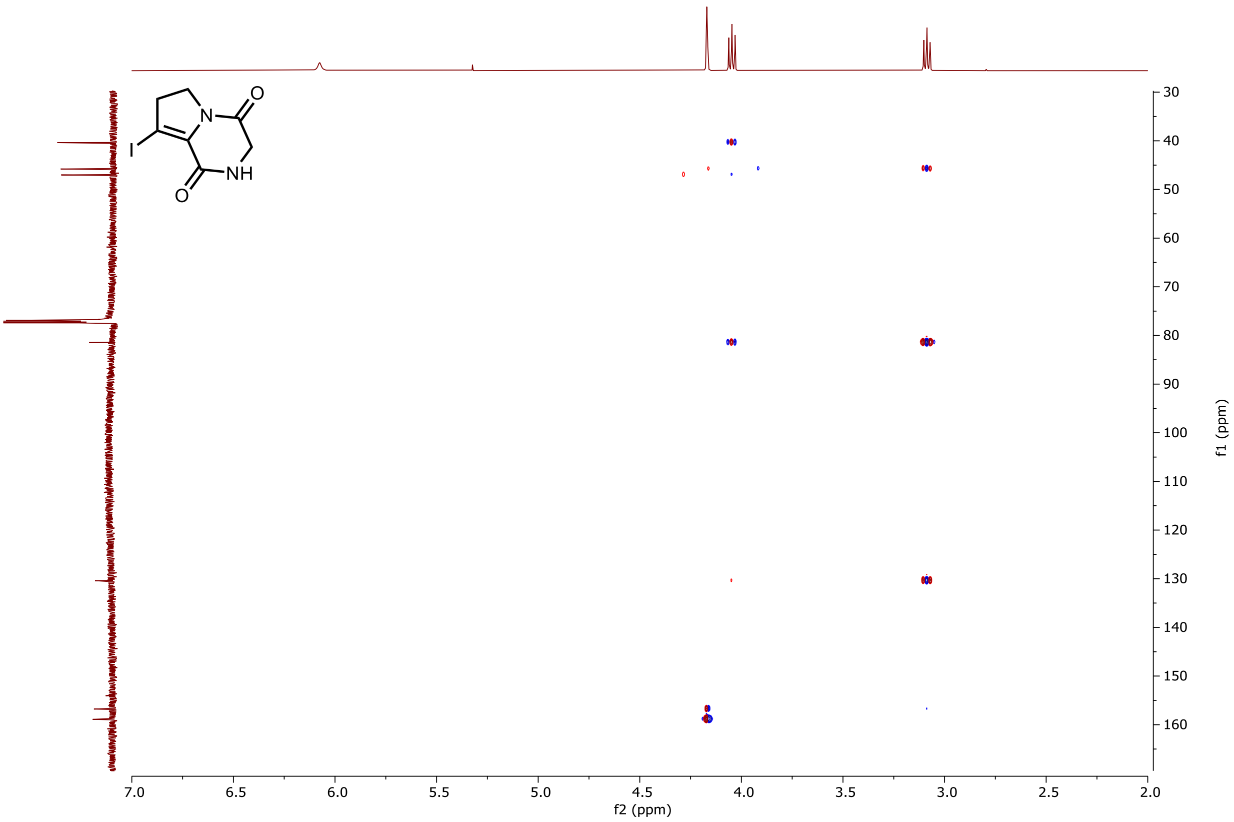

2.31. ^{1}H NMR Spectrum for Lactim Ether 13 (600 MHz, $CDCl_3$)

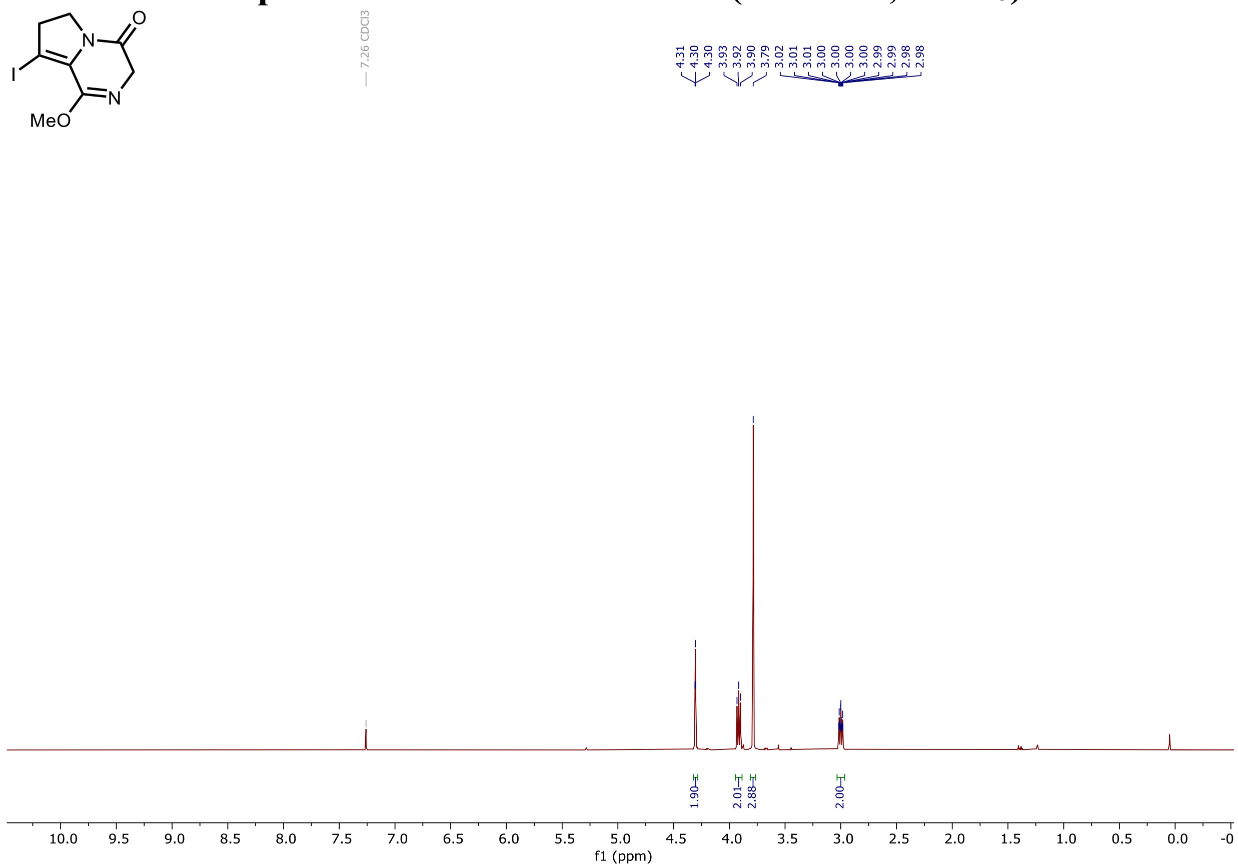

2.32. ^{13}C NMR Spectrum for Lactim Ether 13 (151 MHz, $CDCl_3$)

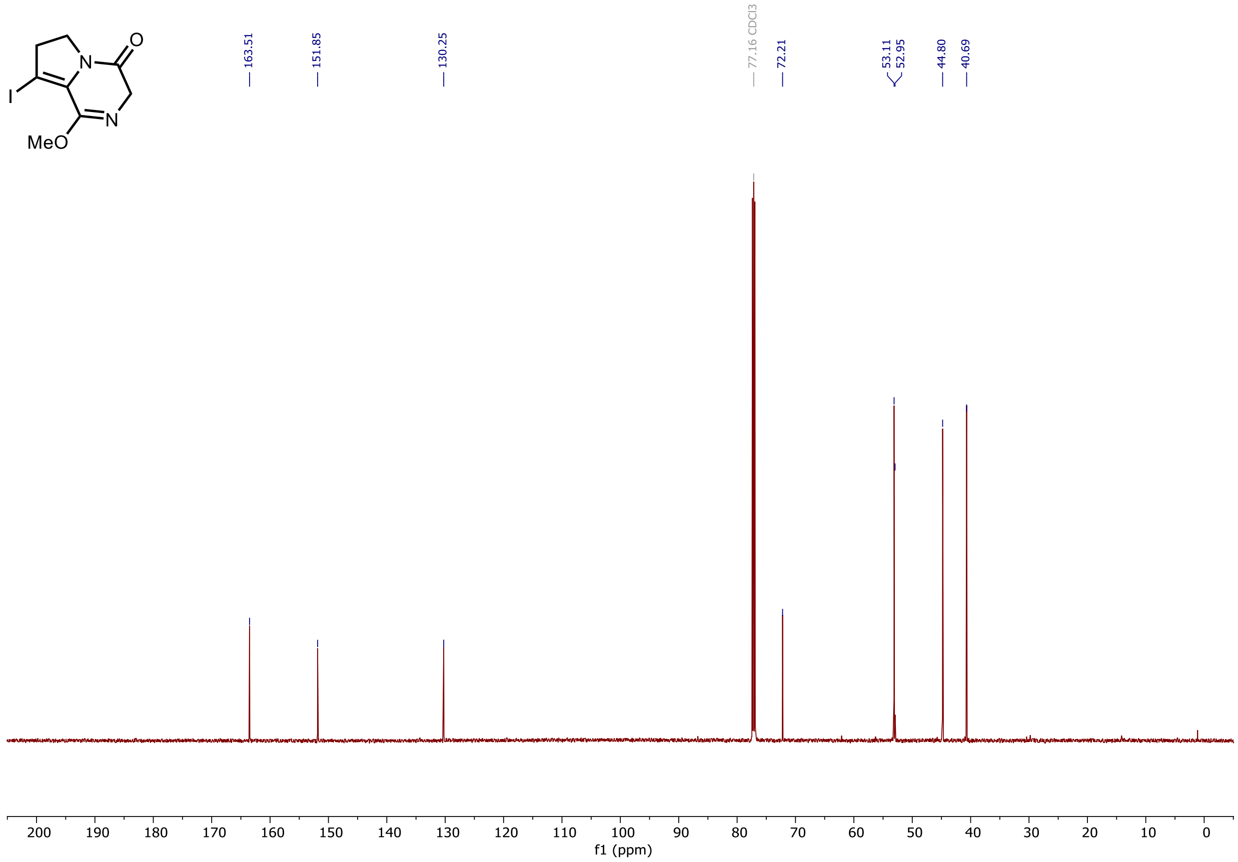

2.33. ^{13}C DEPT-135 Spectrum for Lactim Ether 13 ($CDCl_3$)

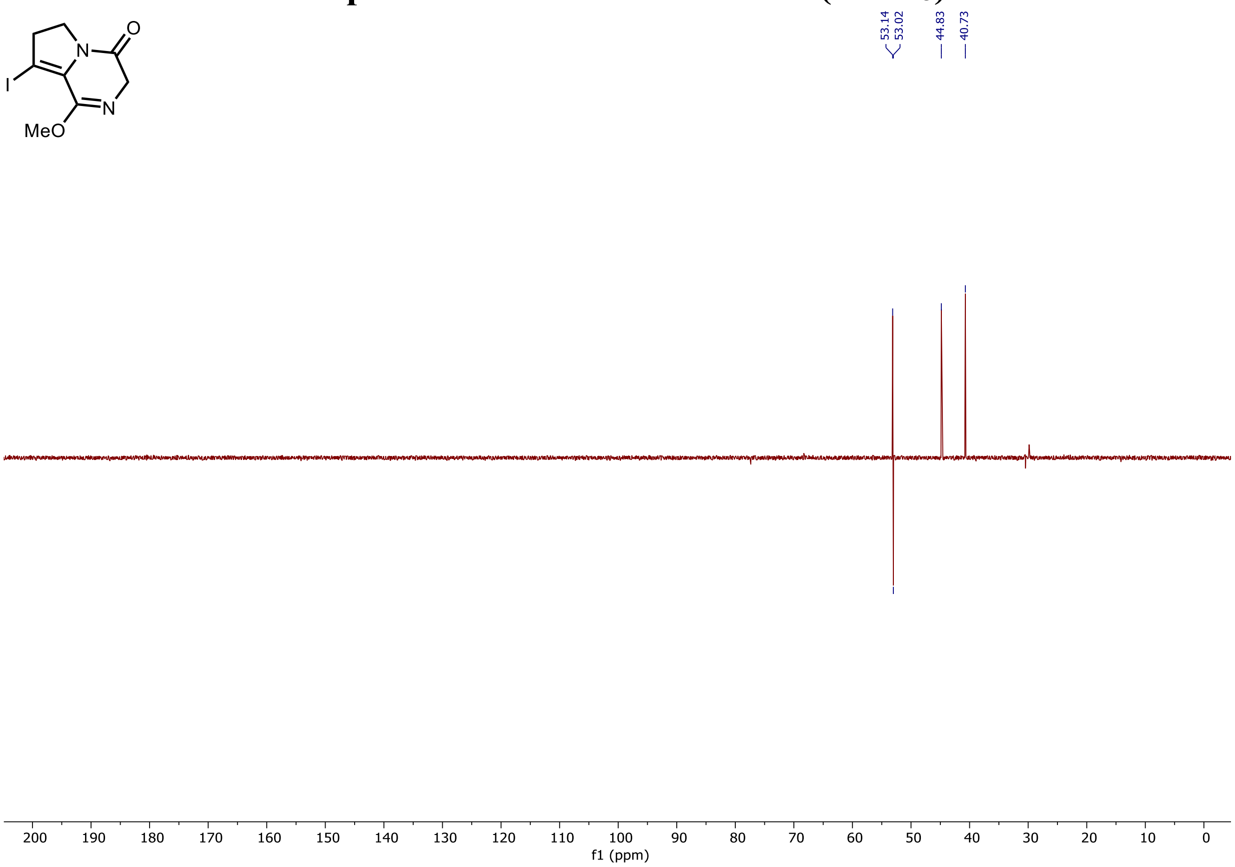

2.34. ^{13}C DEPT-Q135 Spectrum for Lactim Ether 13 ($CDCl_3$)

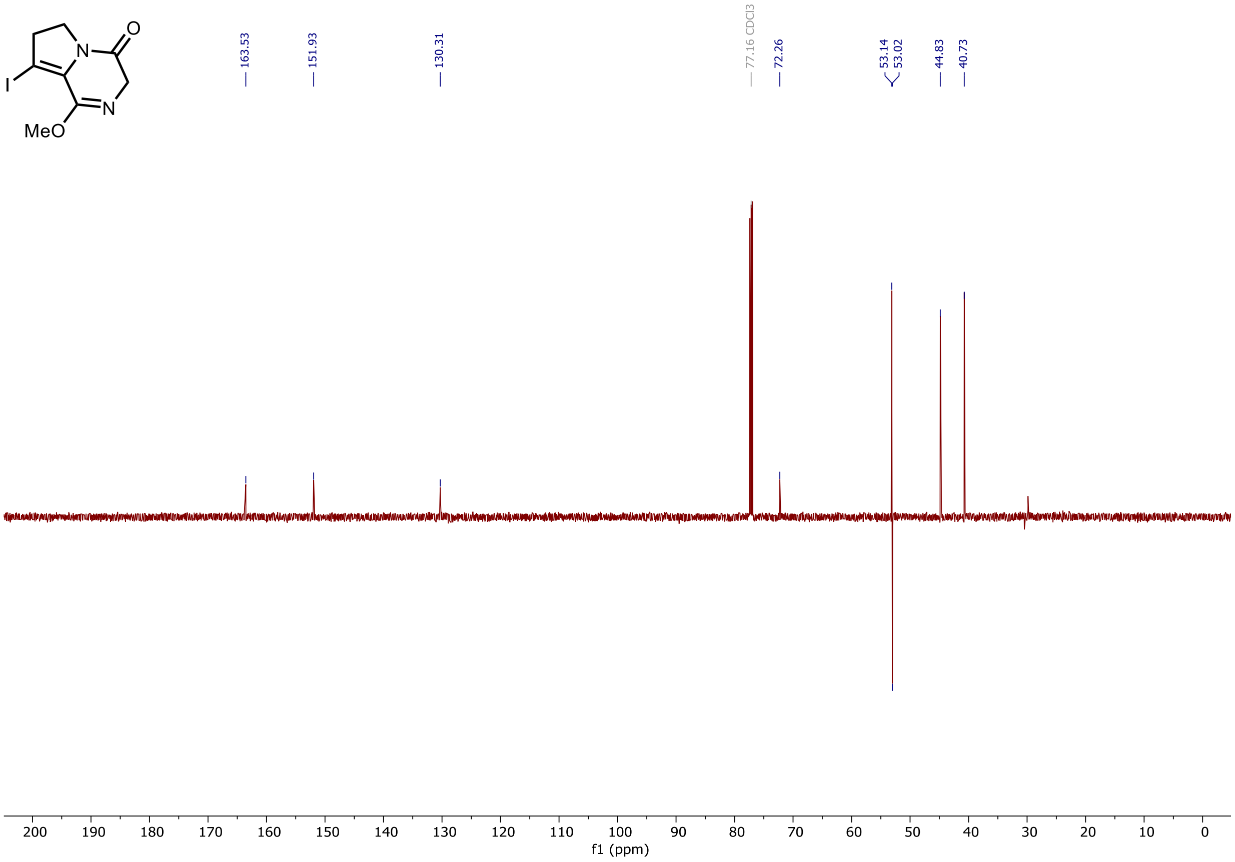

2.35. ^{1}H-^{1}H COSY Spectrum for Lactim Ether 13 ($CDCl_3$)

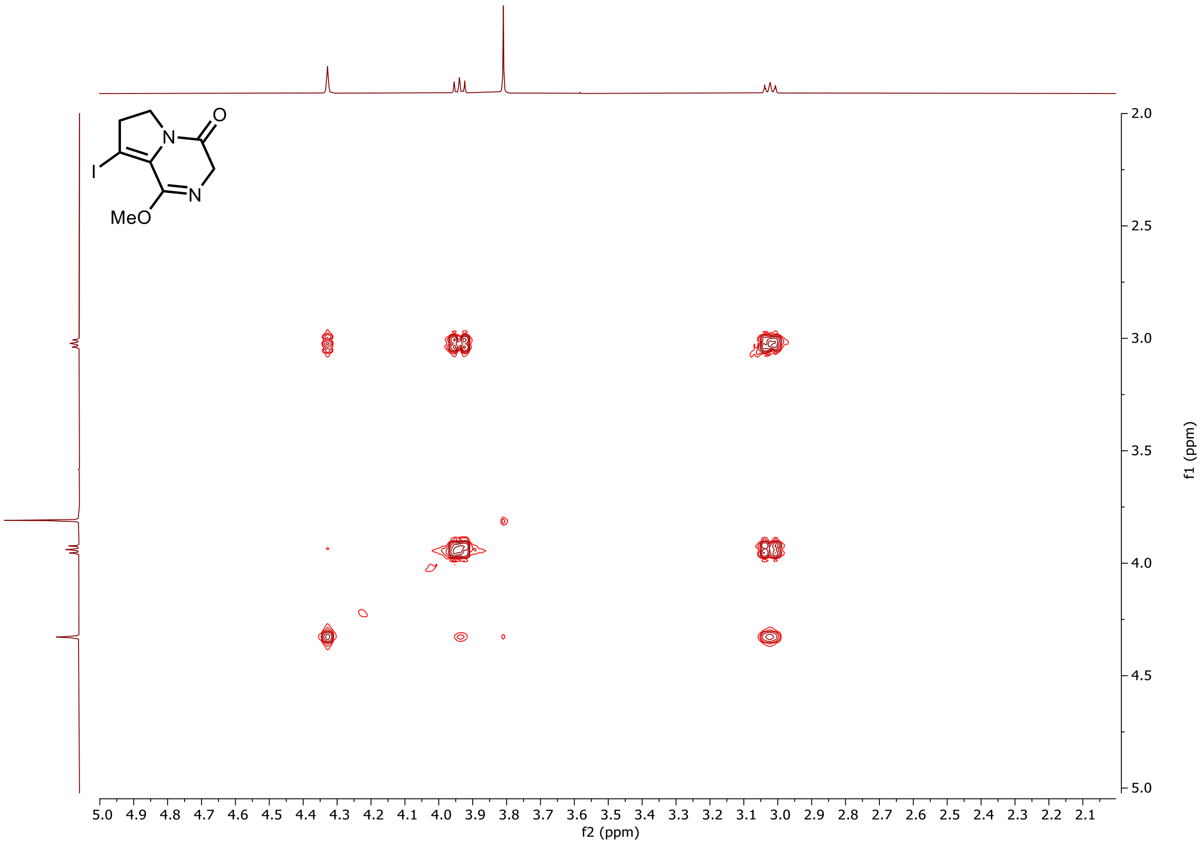

2.36. ^{1}H-^{13}C HSQC Spectrum for Lactim Ether 13 ($CDCl_3$)

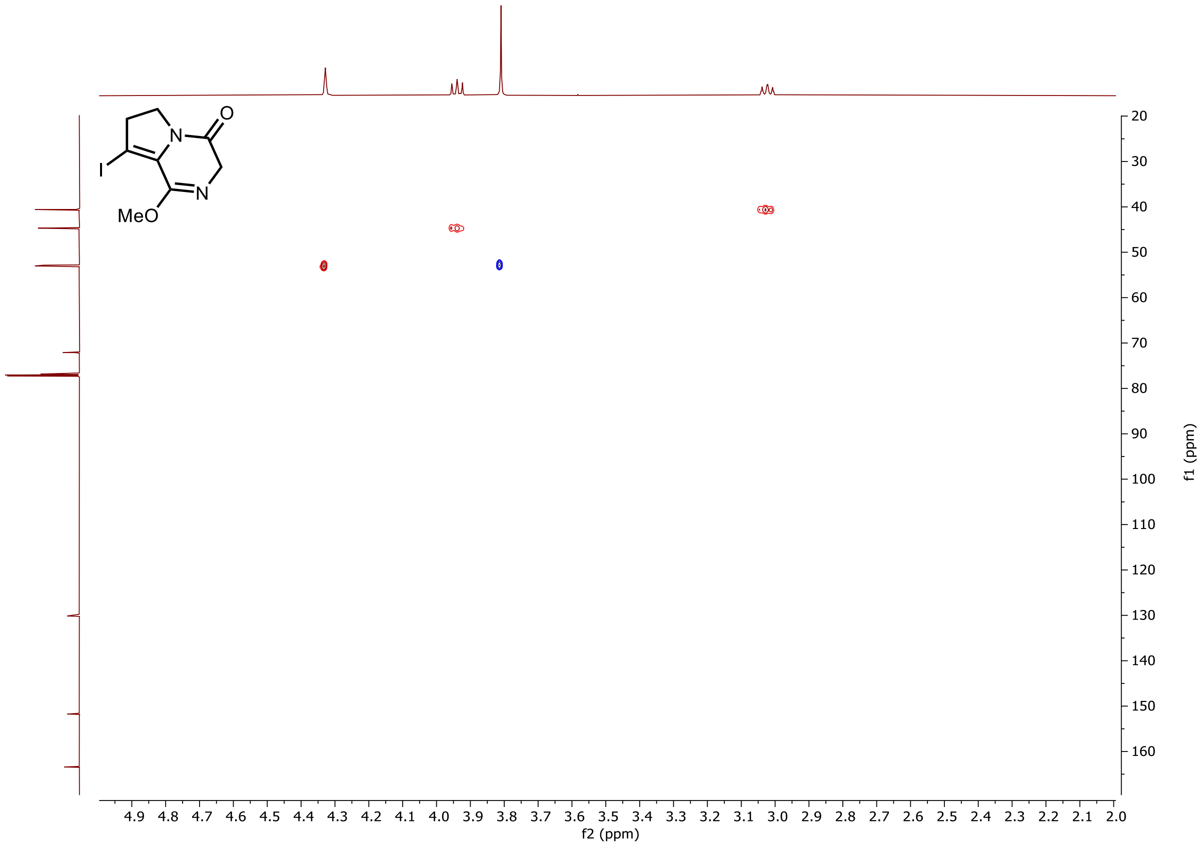

2.37. ^{1}H-^{13}C HMBC Spectrum for Lactim Ether 13 ($CDCl_3$)

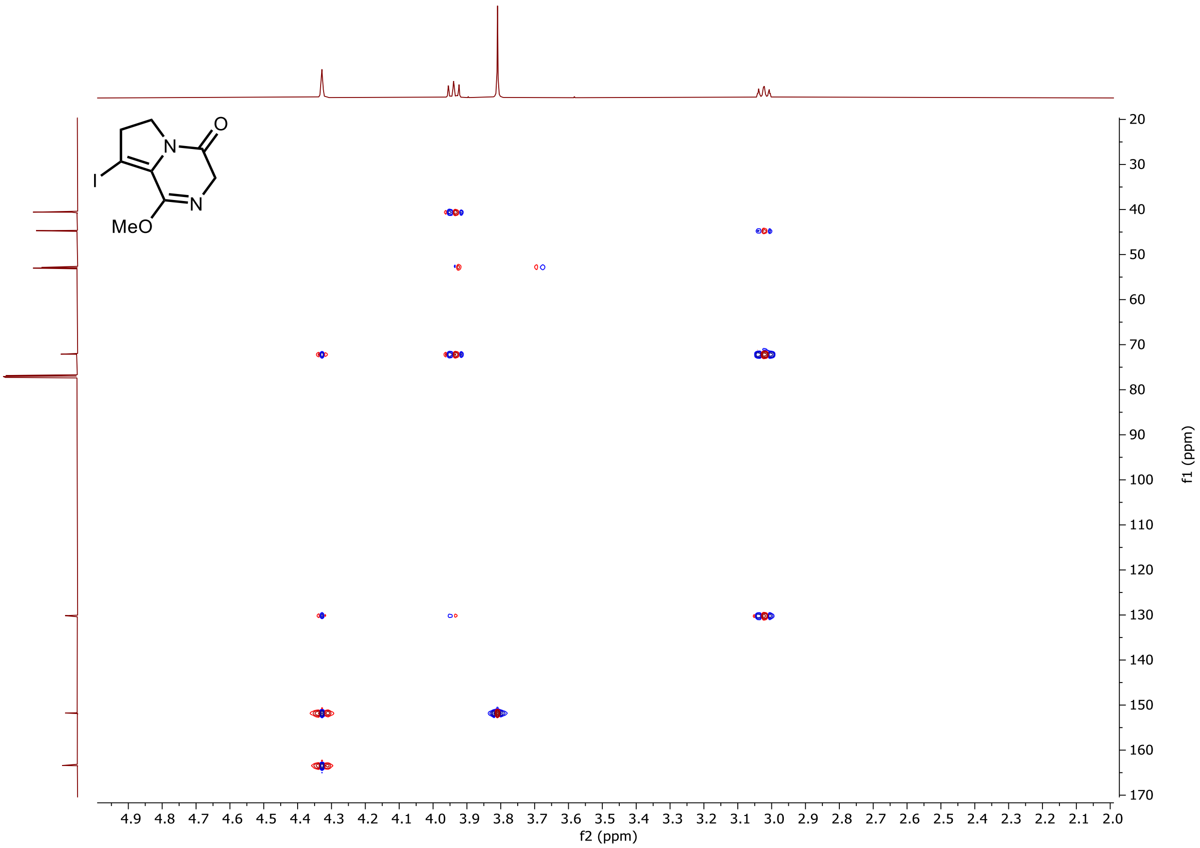

2.38. ^{1}H NMR Spectrum for hexamethylditin (600 MHz, $CDCl_3$)

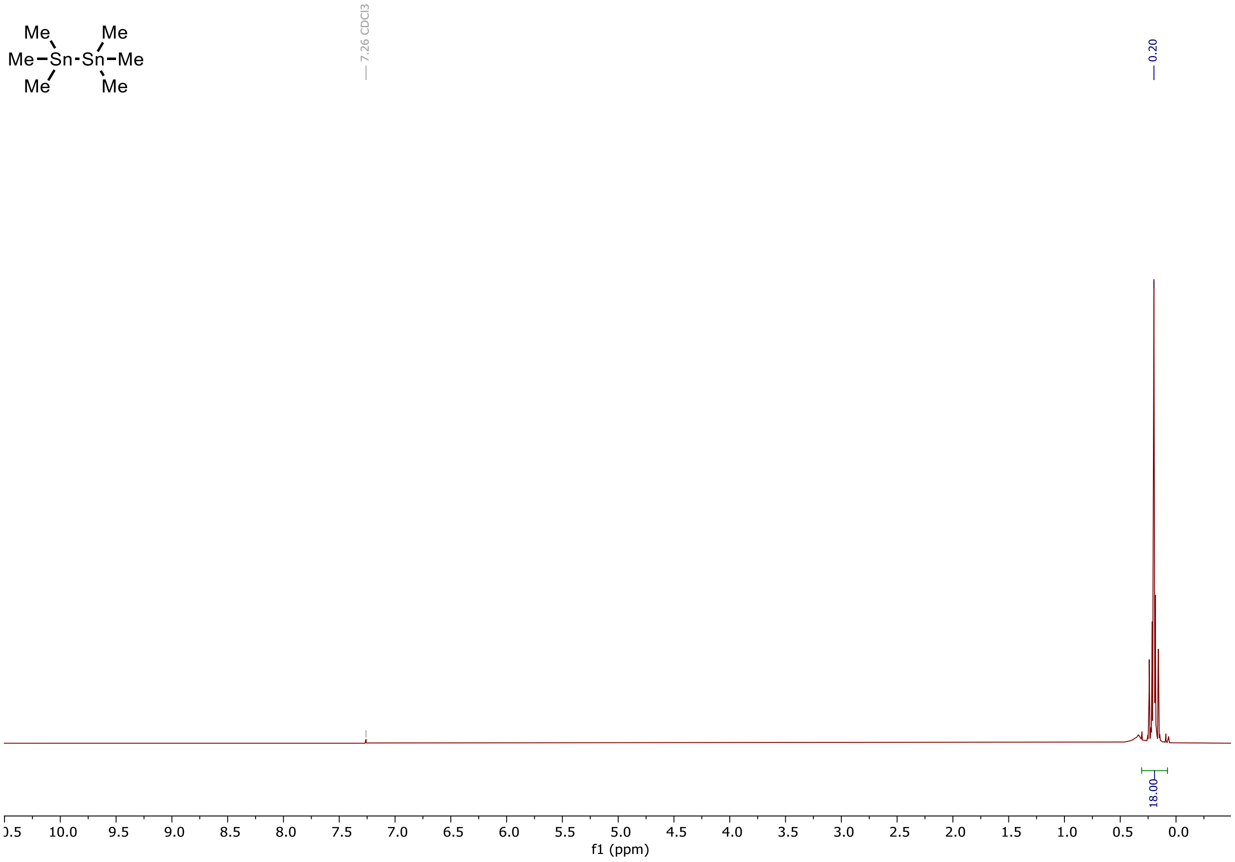

2.39. ^{13}C NMR Spectrum for hexamethylditin (151 MHz, $CDCl_3$)

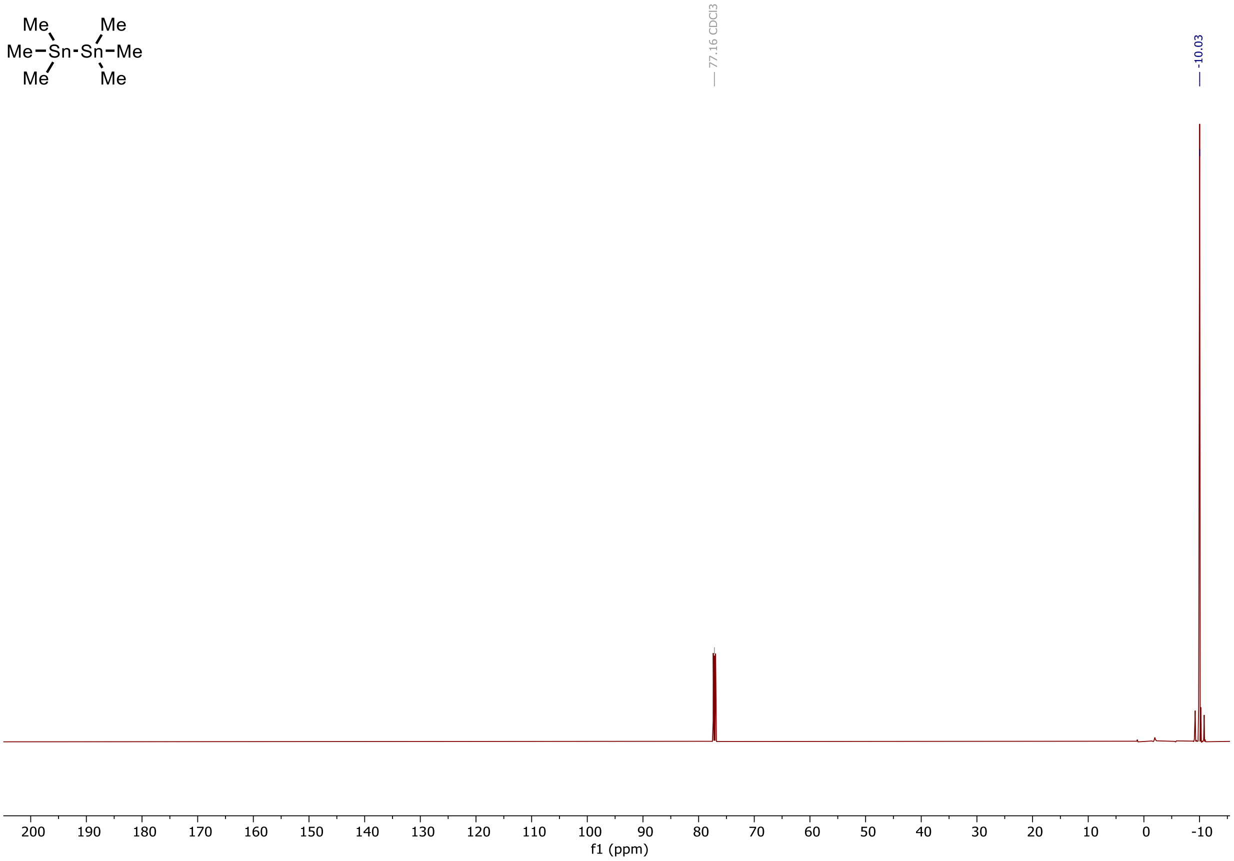

2.40. ^{119}Sn NMR Spectrum for hexamethylditin (149 MHz, $CDCl_3$)

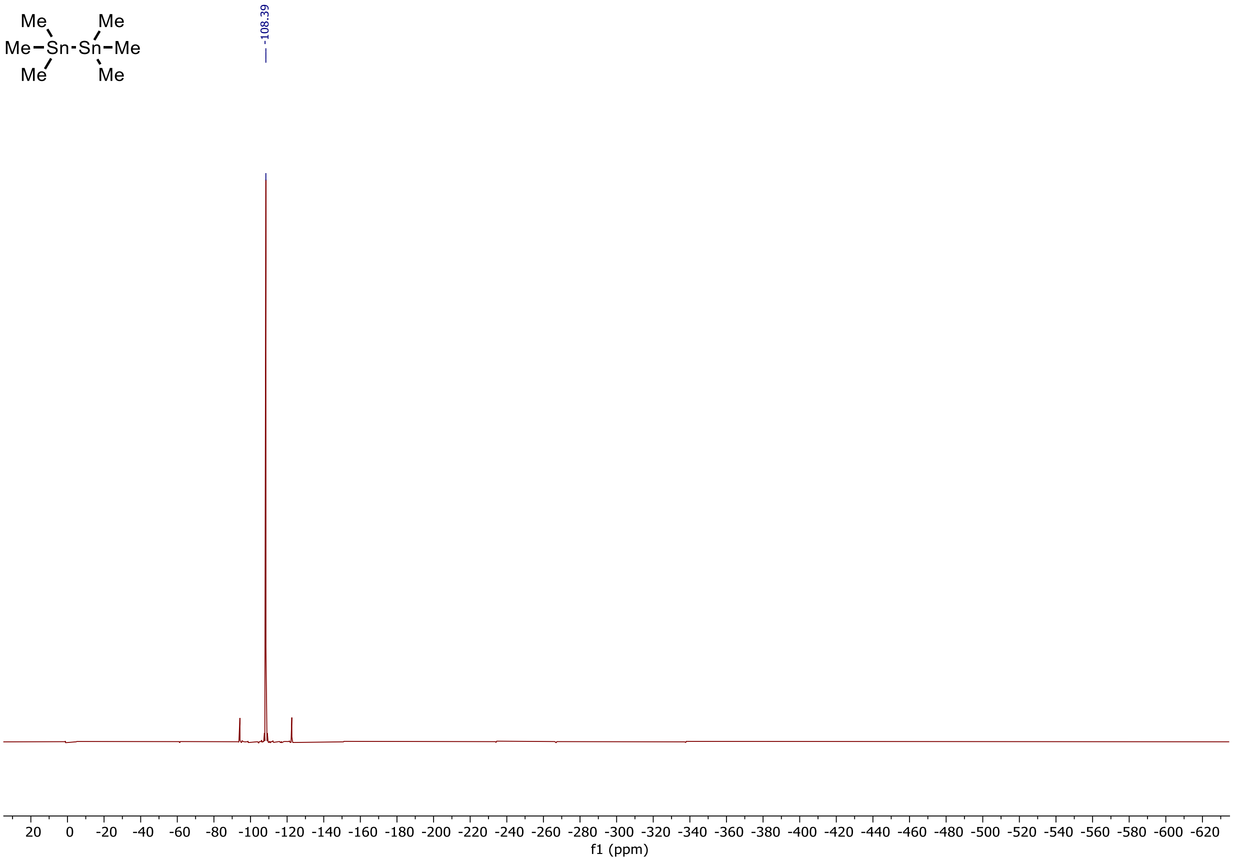

2.41. ^{1}H NMR Spectrum for Organotin 12 (500 MHz, $CDCl_3$)

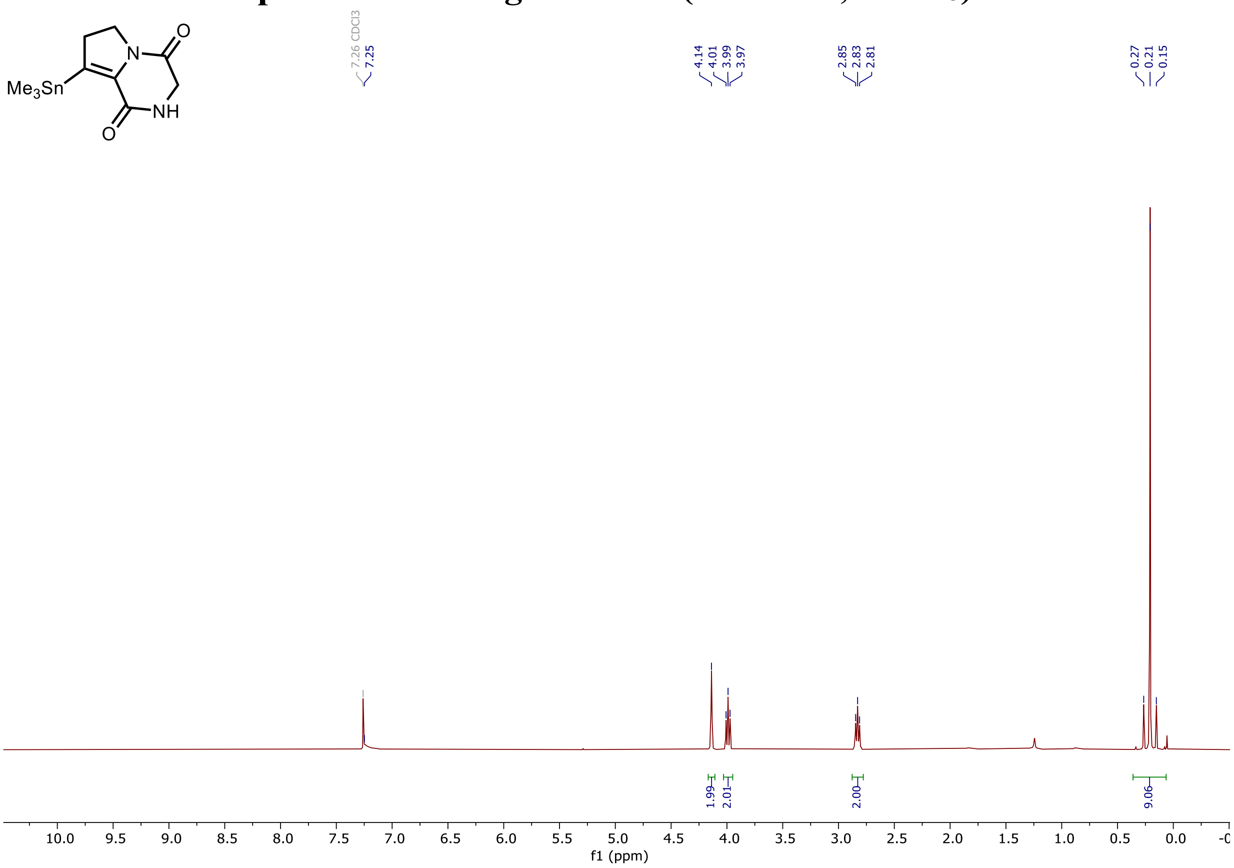

2.42. ^{13}C NMR Spectrum for Organotin 12 (126 MHz, $CDCl_3$)

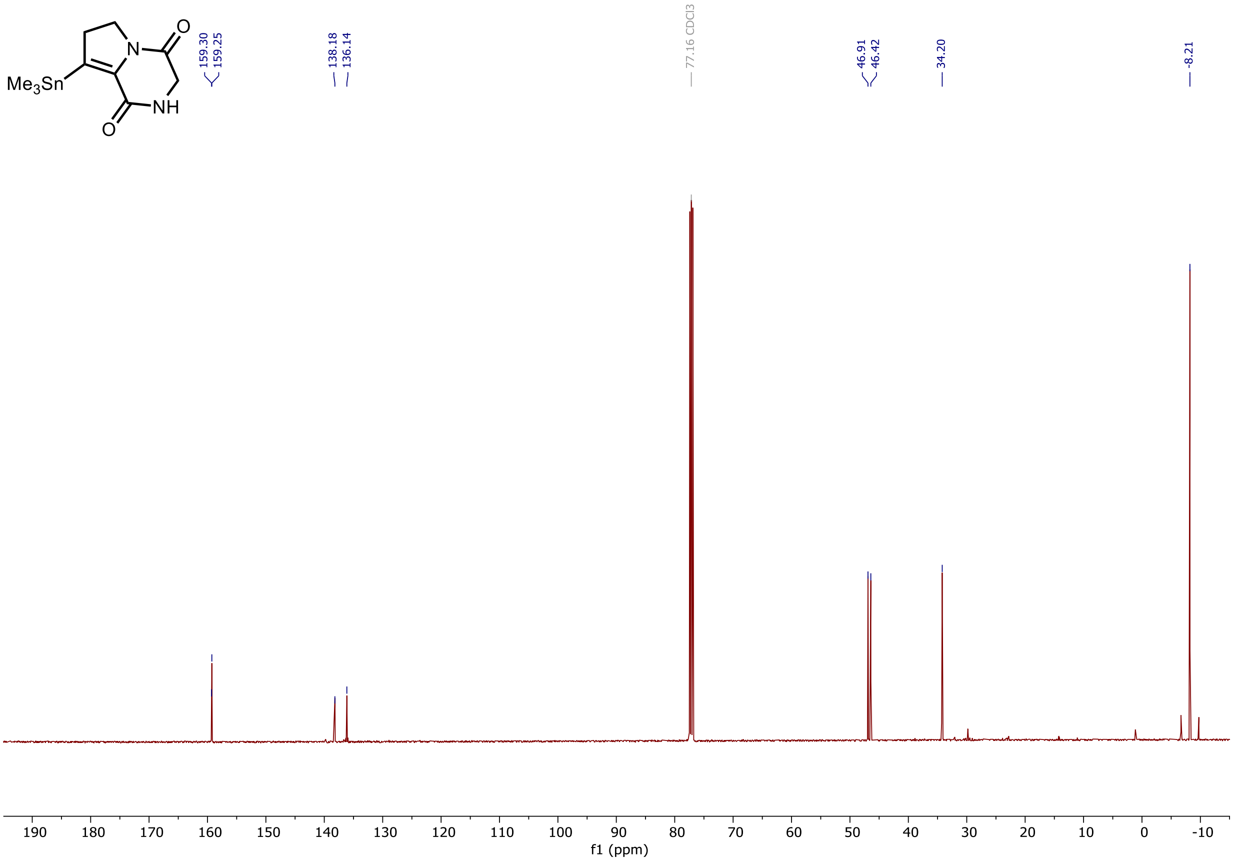

2.43. ^{13}C DEPT-135 Spectrum for Organotin 12 ($CDCl_3$)

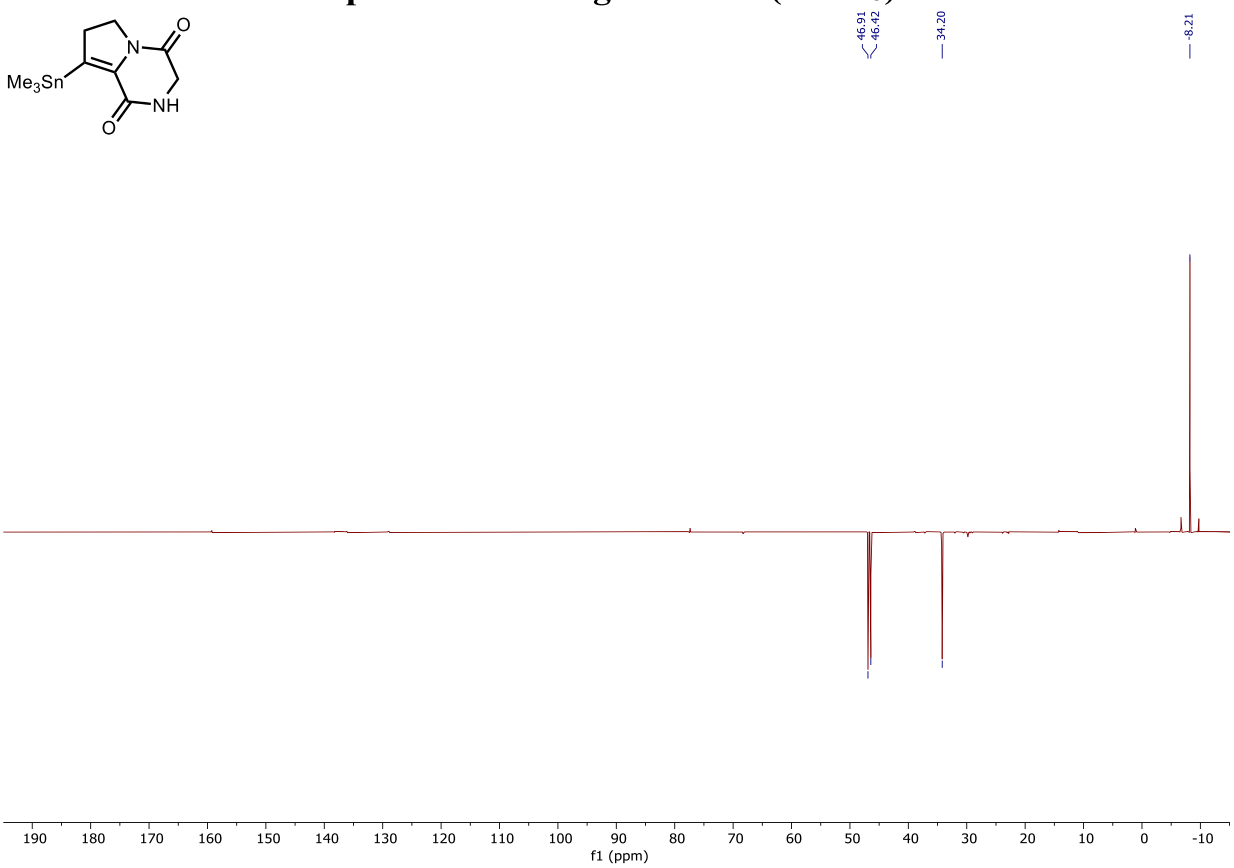

2.44. ^{13}C DEPT-Q135 Spectrum for Organotin 12 ($CDCl_3$)

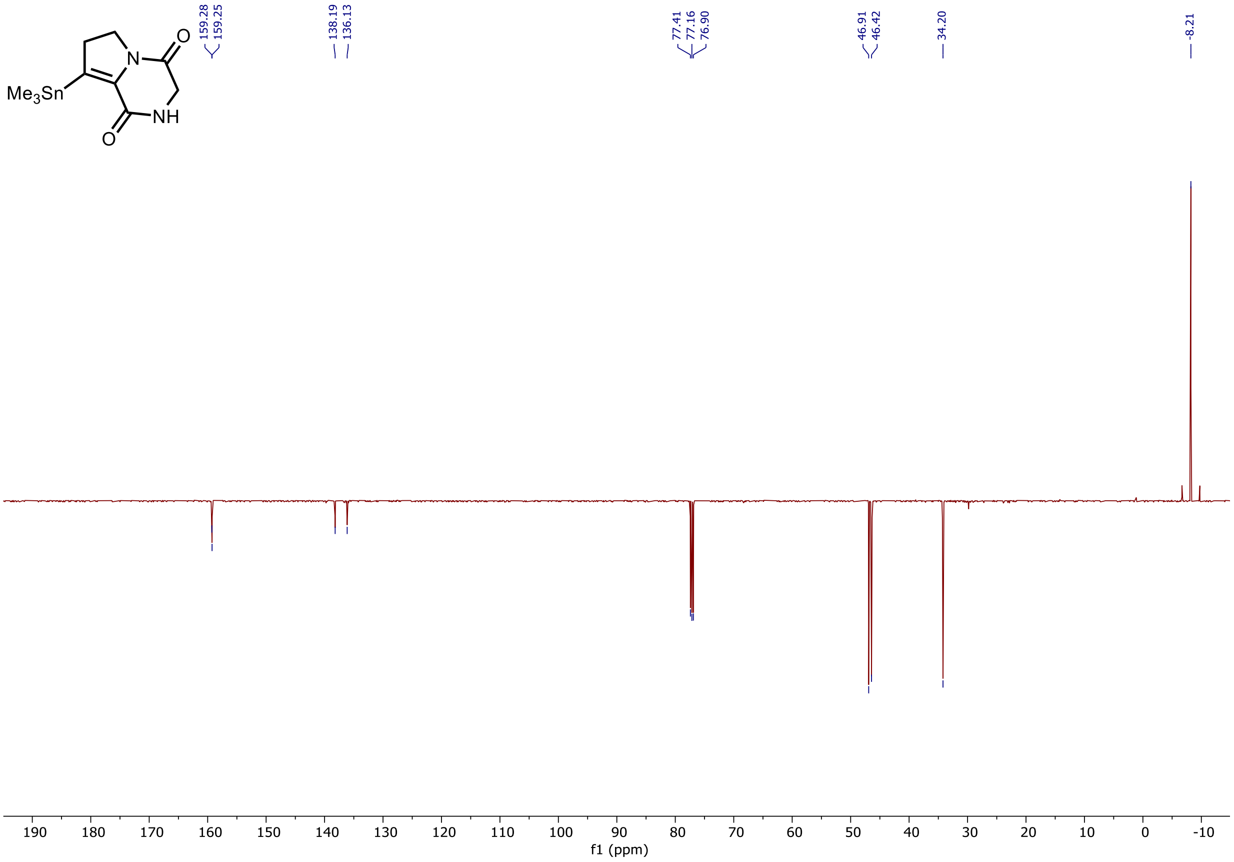

2.45. ^{1}H-^{1}H COSY Spectrum for Organotin 12 ($CDCl_3$)

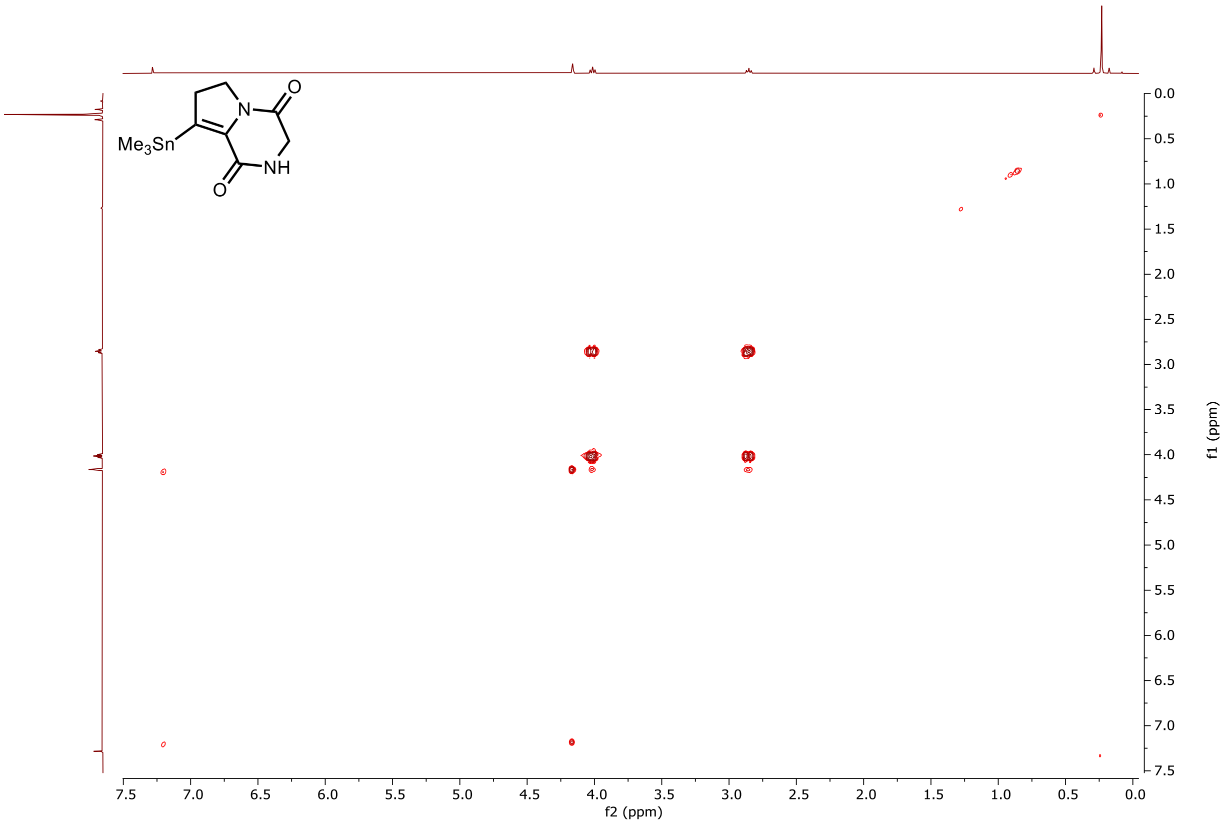

2.46. ^{1}H-^{13}C HSQC Spectrum for Organotin 12 ($CDCl_3$)

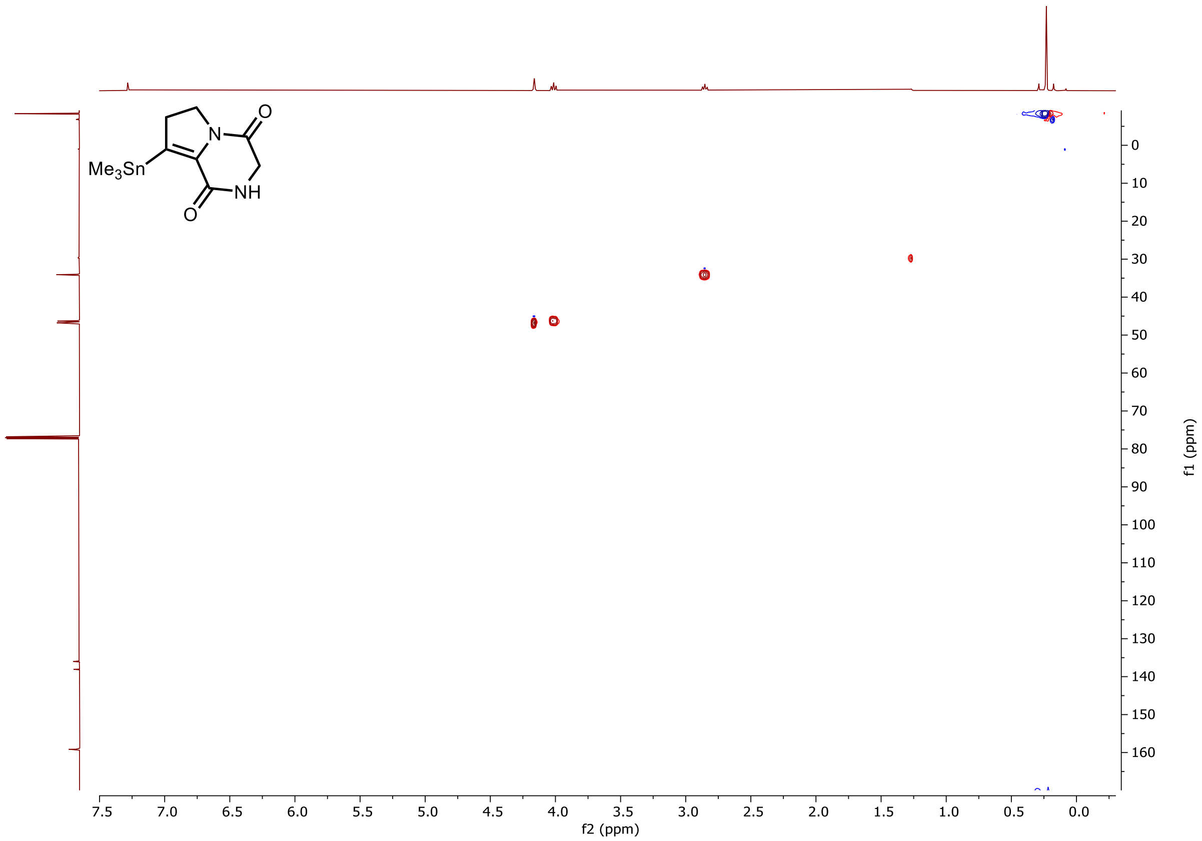

2.47. ^{1}H-^{13}C HMBC Spectrum for Organotin 12 ($CDCl_3$)

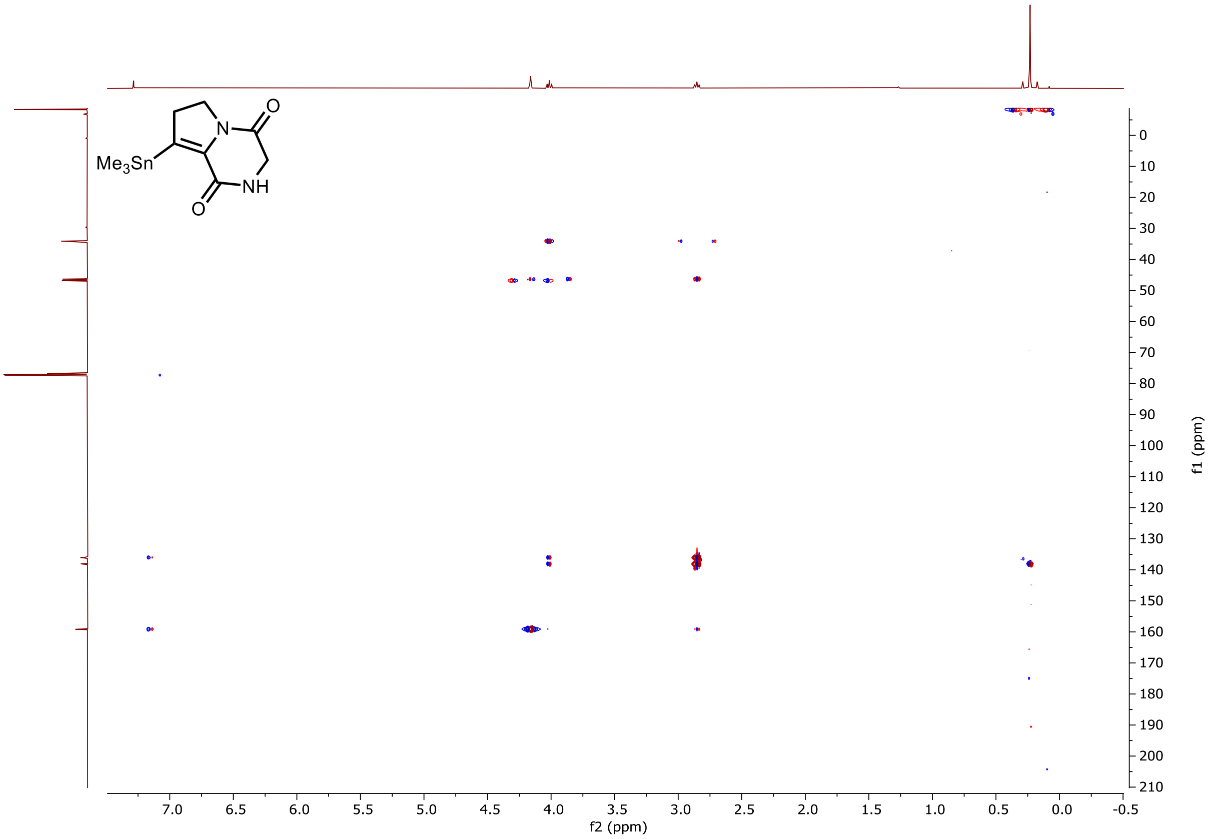

2.48. ^{119}Sn NMR Spectrum for Organotin 12 (149 MHz, $CDCl_3$)

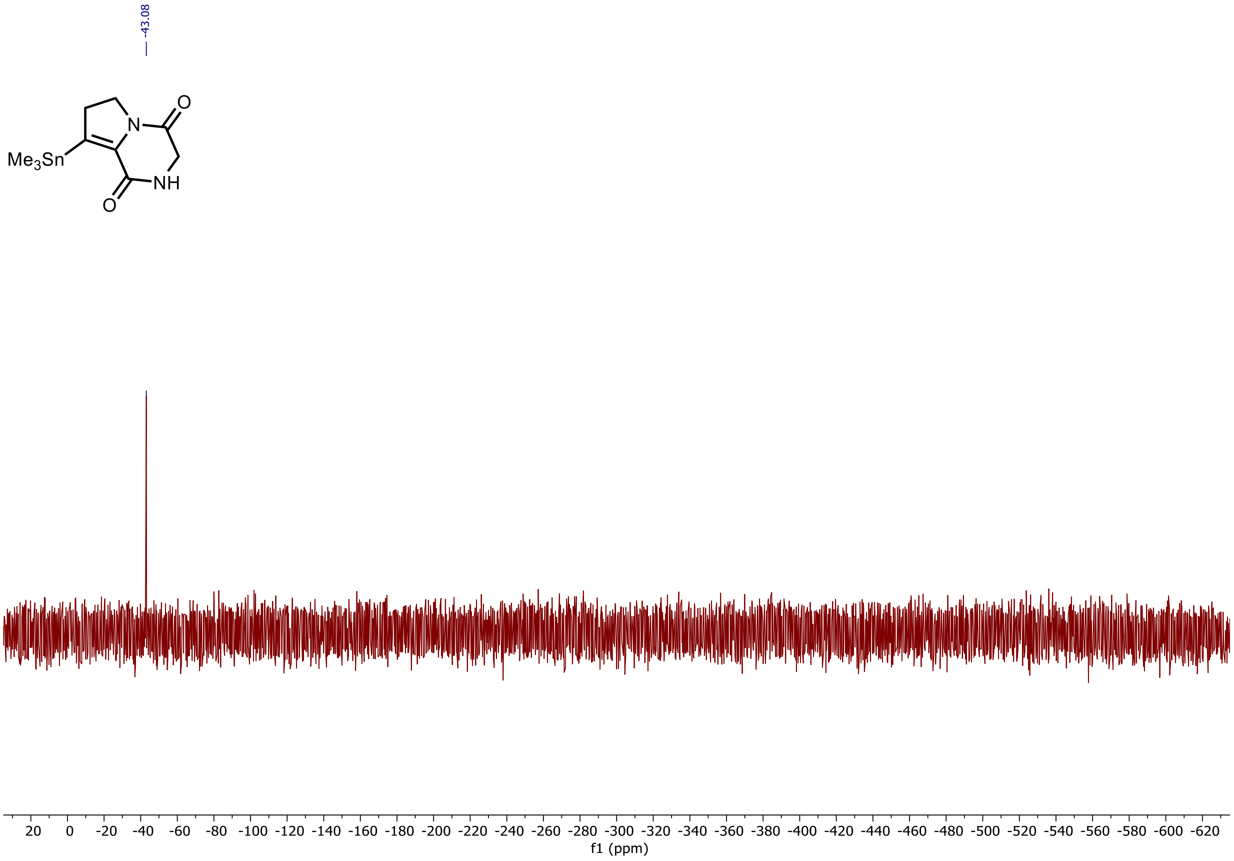

2.49. ^{1}H NMR Spectrum for Bis-Diketopiperazine 14 (600 MHz, $CDCl_3$)

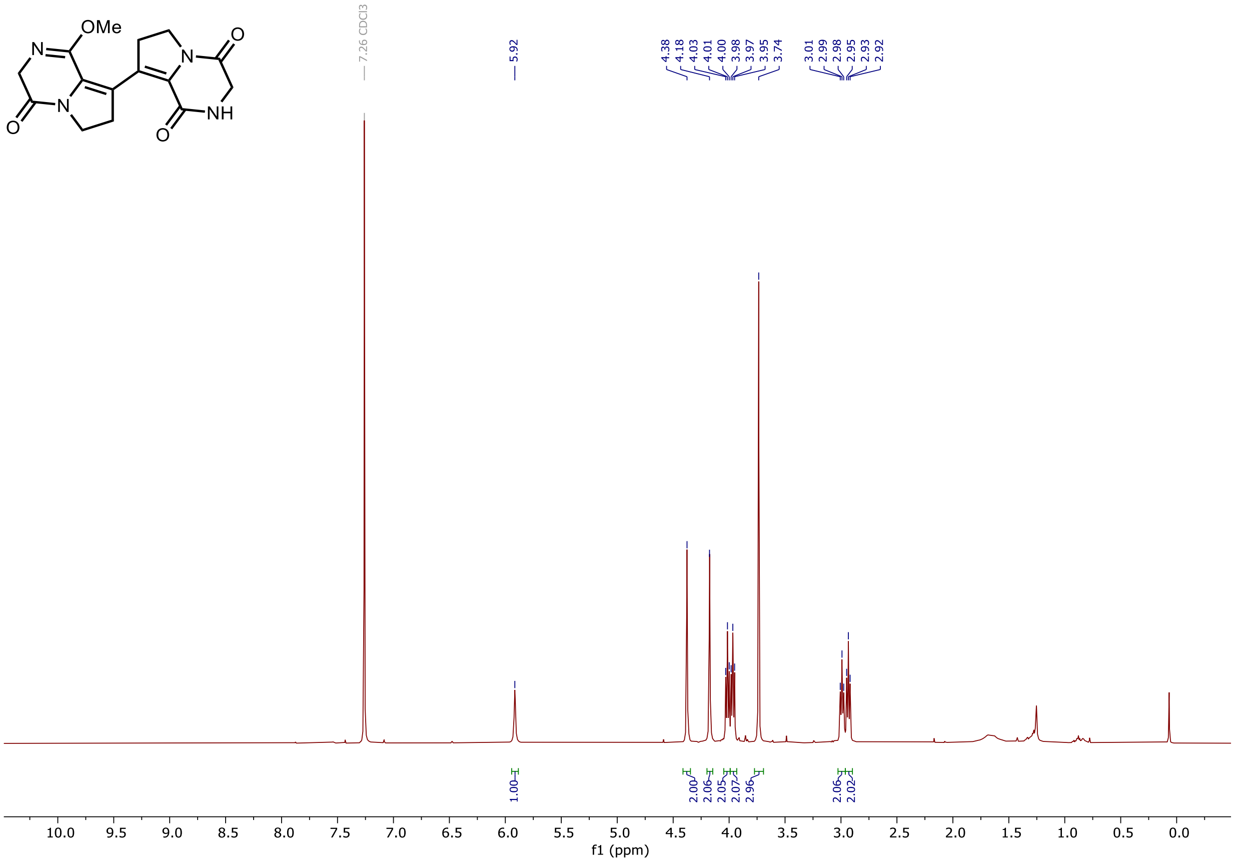

2.50. ^{13}C NMR Spectrum for Bis-Diketopiperazine 14 (126 MHz, $CDCl_3$)

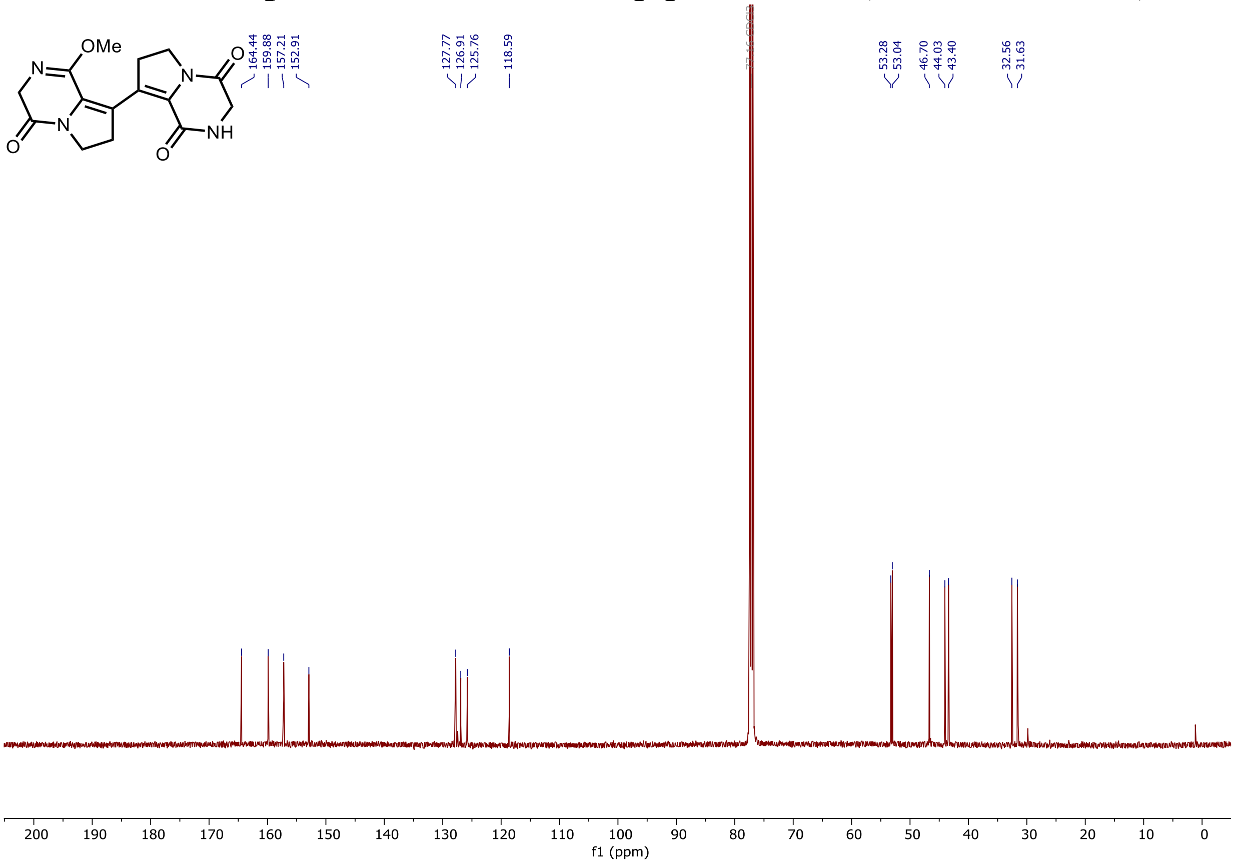

2.51. ^{1}H-^{1}H COSY Spectrum for Bis-Diketopiperazine 14 (CDCl$_3$)

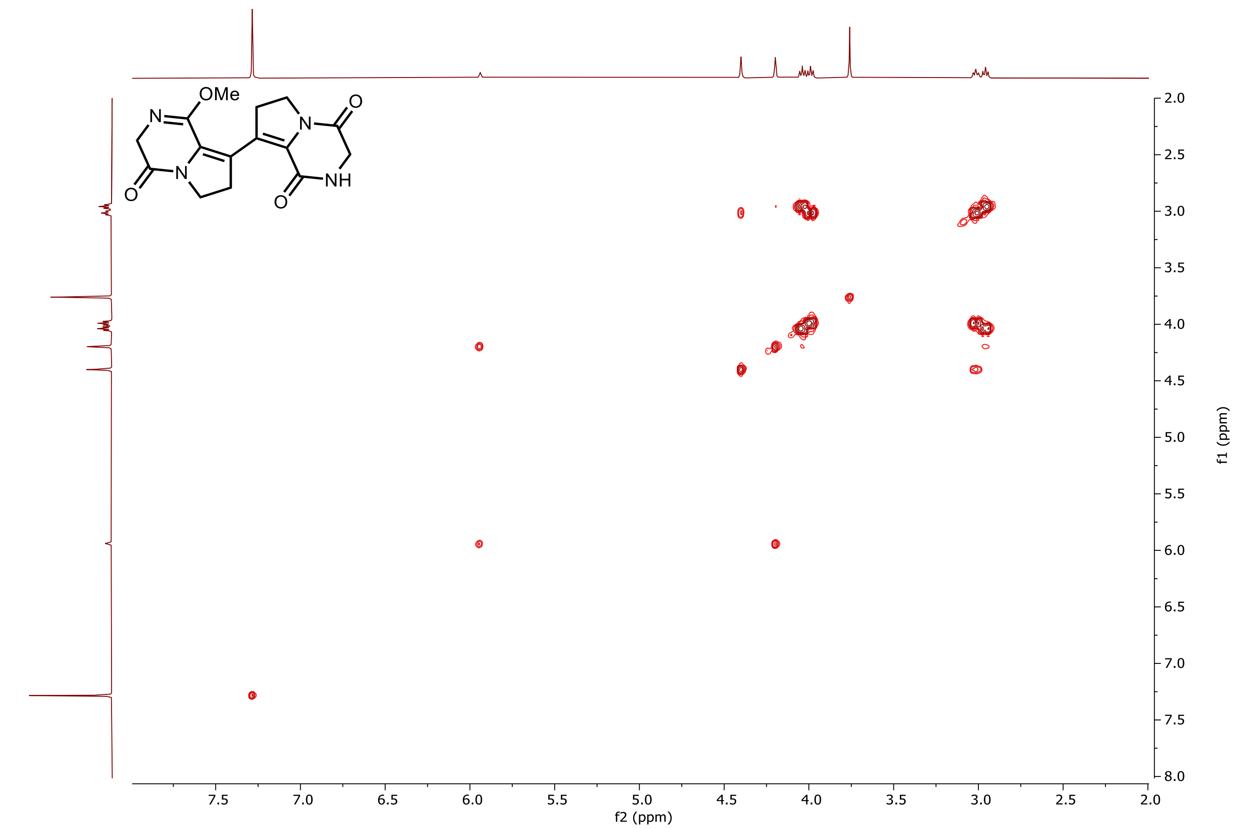

2.52. ^{1}H-^{13}C HSQC Spectrum for Bis-Diketopiperazine 14 (CDCl$_3$)

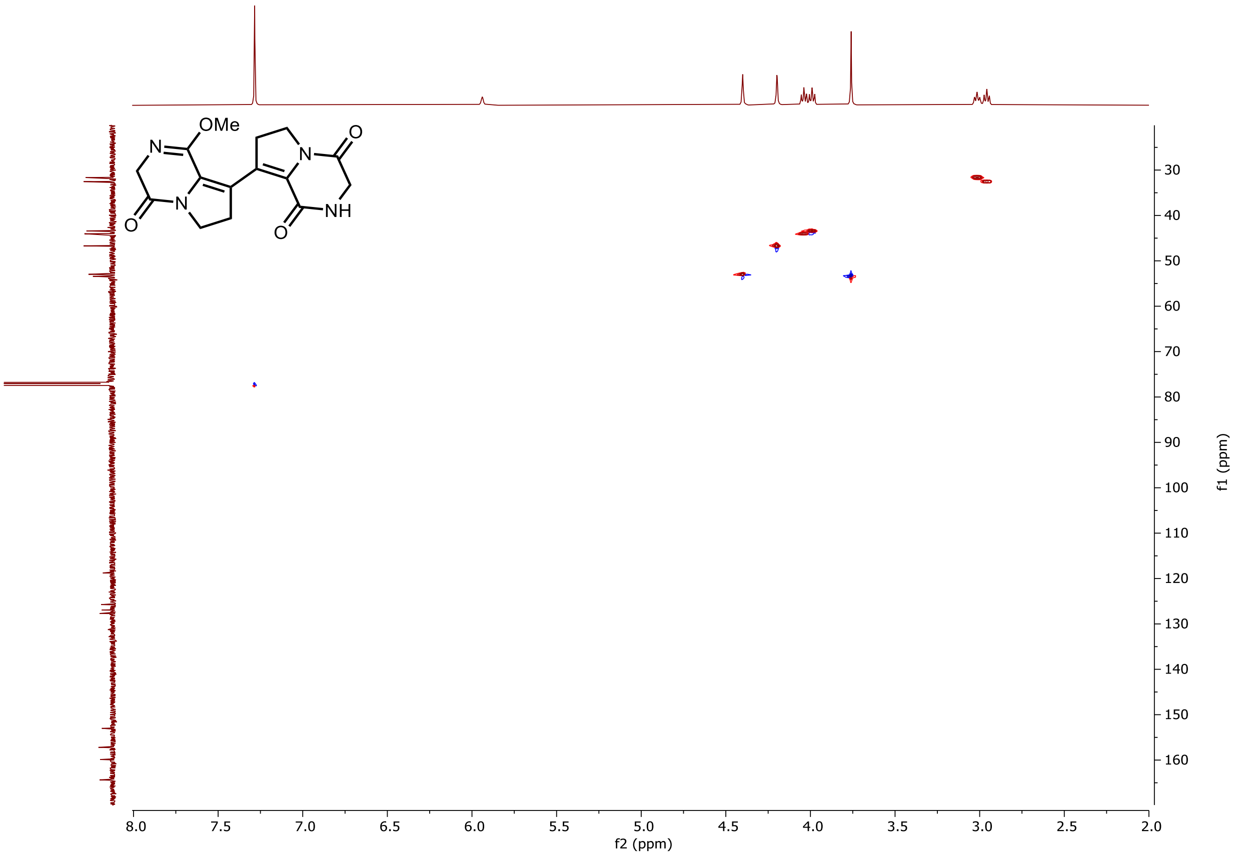

2.53. ^{1}H-^{13}C HMBC Spectrum for Bis-Diketopiperazine 14 ($CDCl_3$)

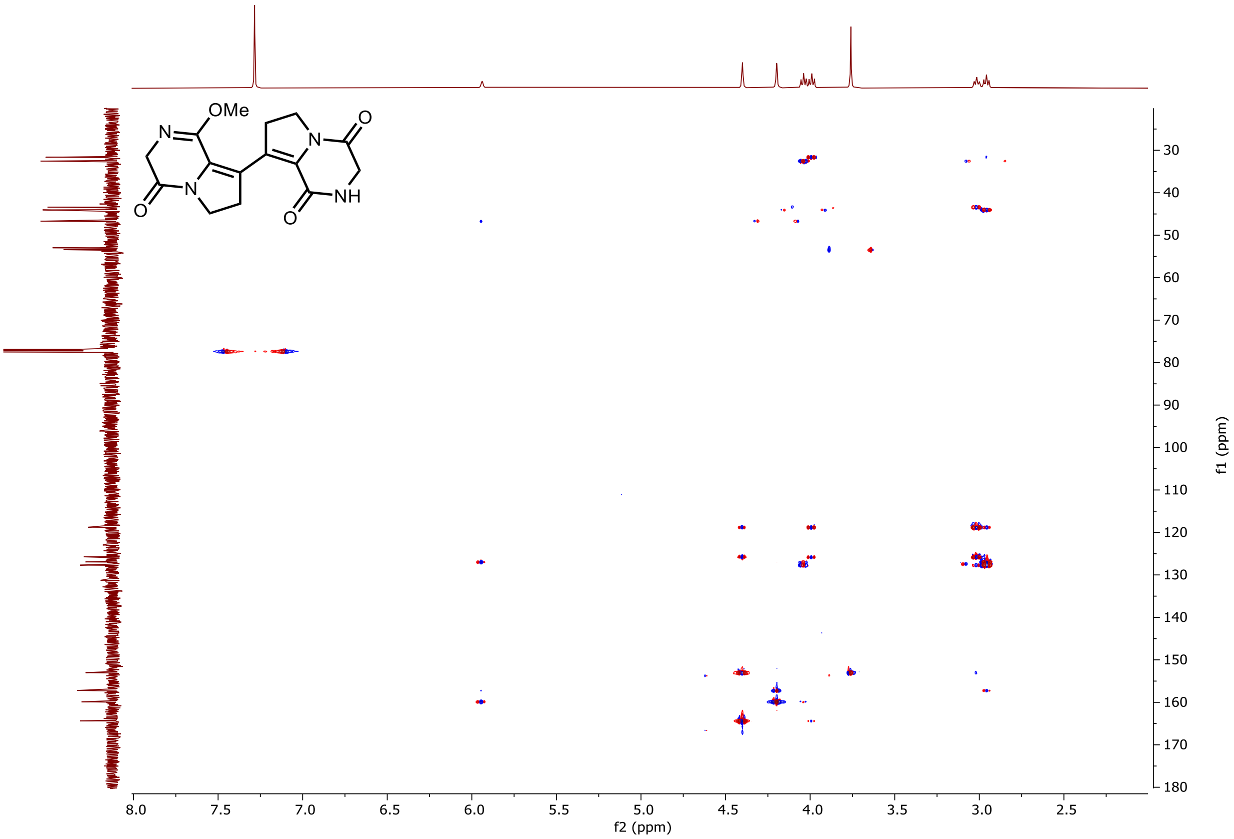

2.54. ^{1}H NMR Spectrum for Bis-Lactim Ether 16 (500 MHz, $CDCl_3$)

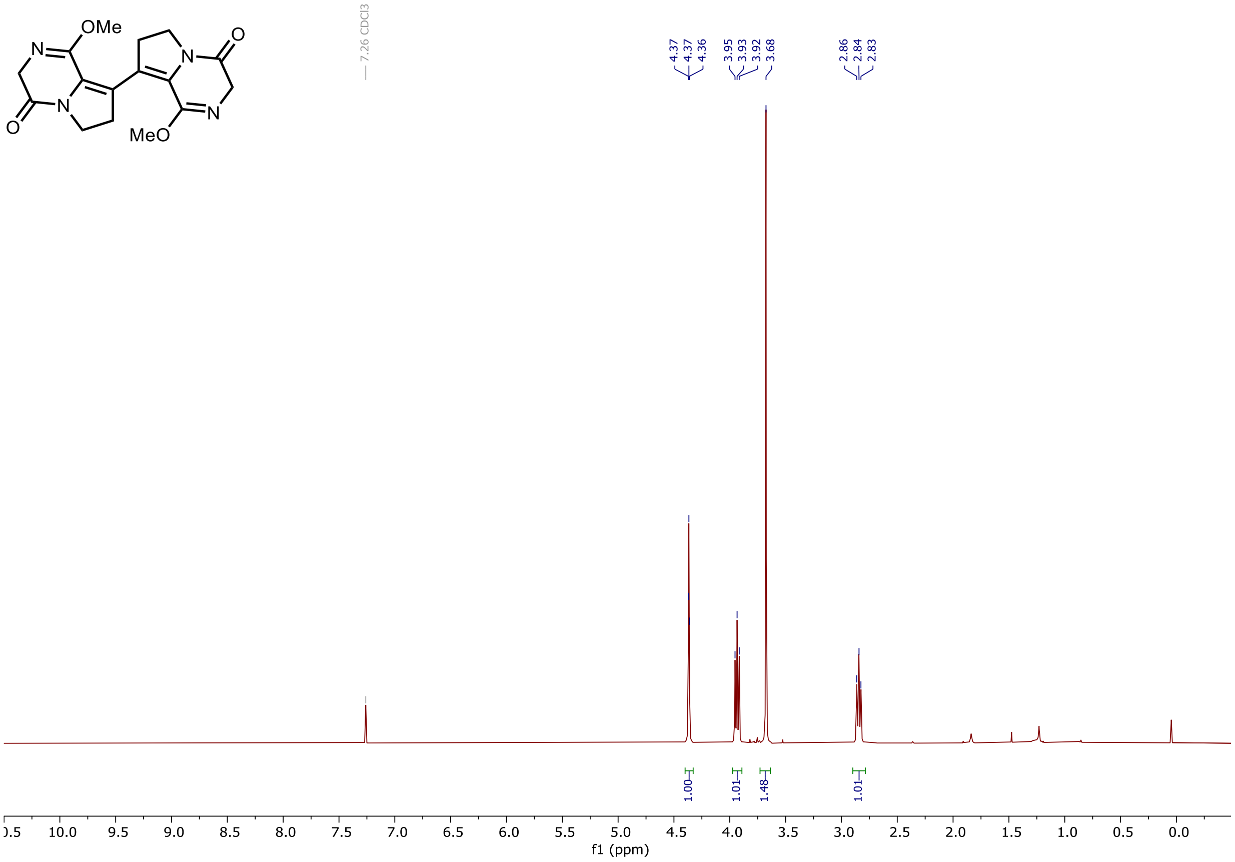

2.55. ^{13}C NMR Spectrum for Bis-Lactim Ether 16 (126 MHz, $CDCl_3$)

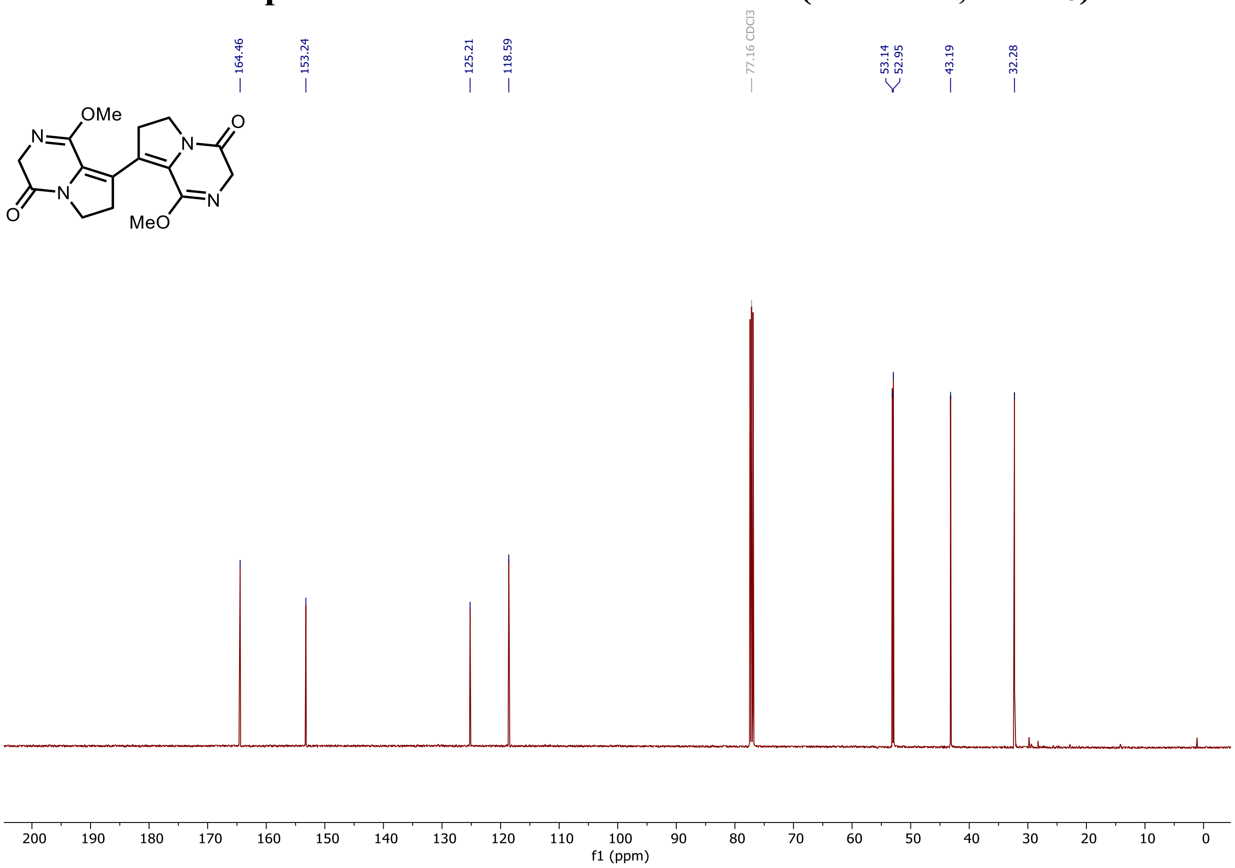

2.56. ^{1}H-^{1}H COSY Spectrum for Bis-Lactim Ether 16 ($CDCl_3$)

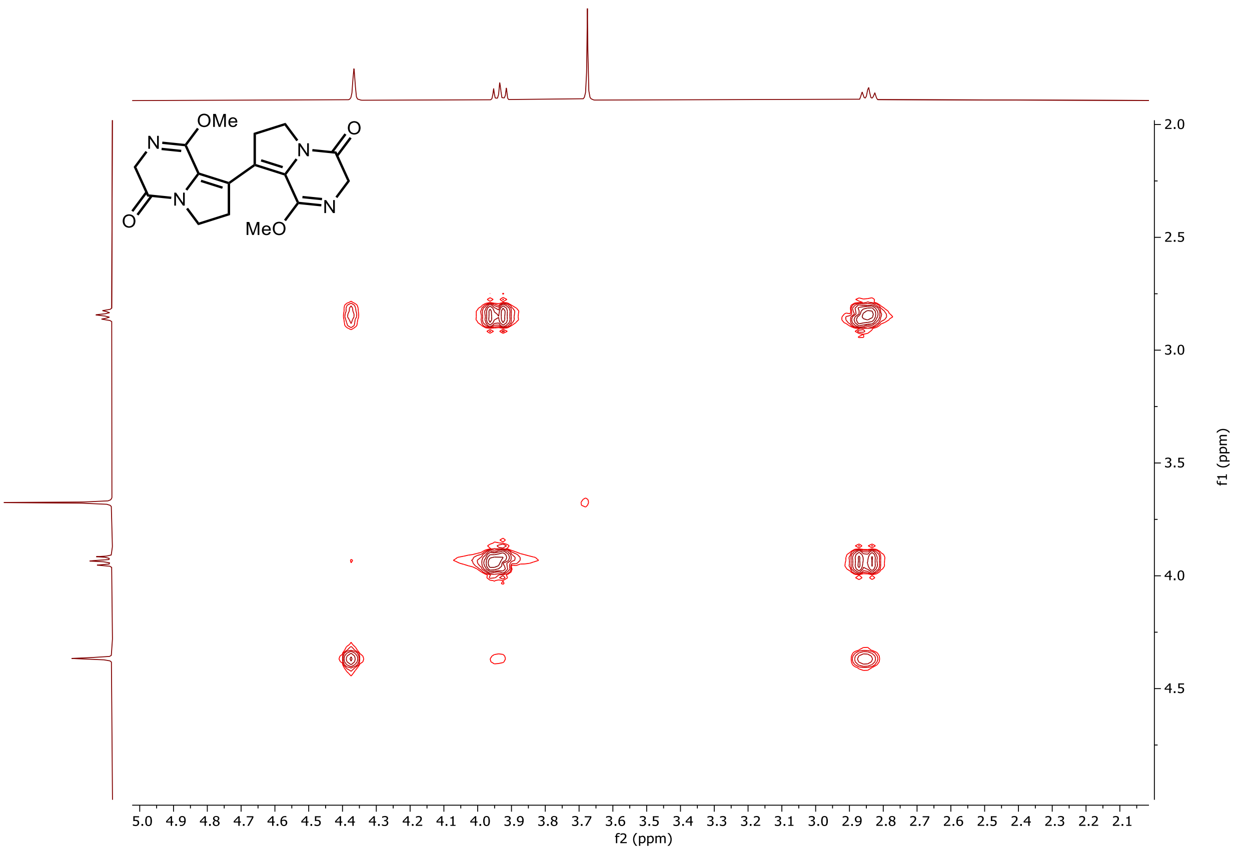

2.57. ^{1}H-^{13}C HSQC Spectrum for Bis-Lactim Ether 16 ($CDCl_3$)

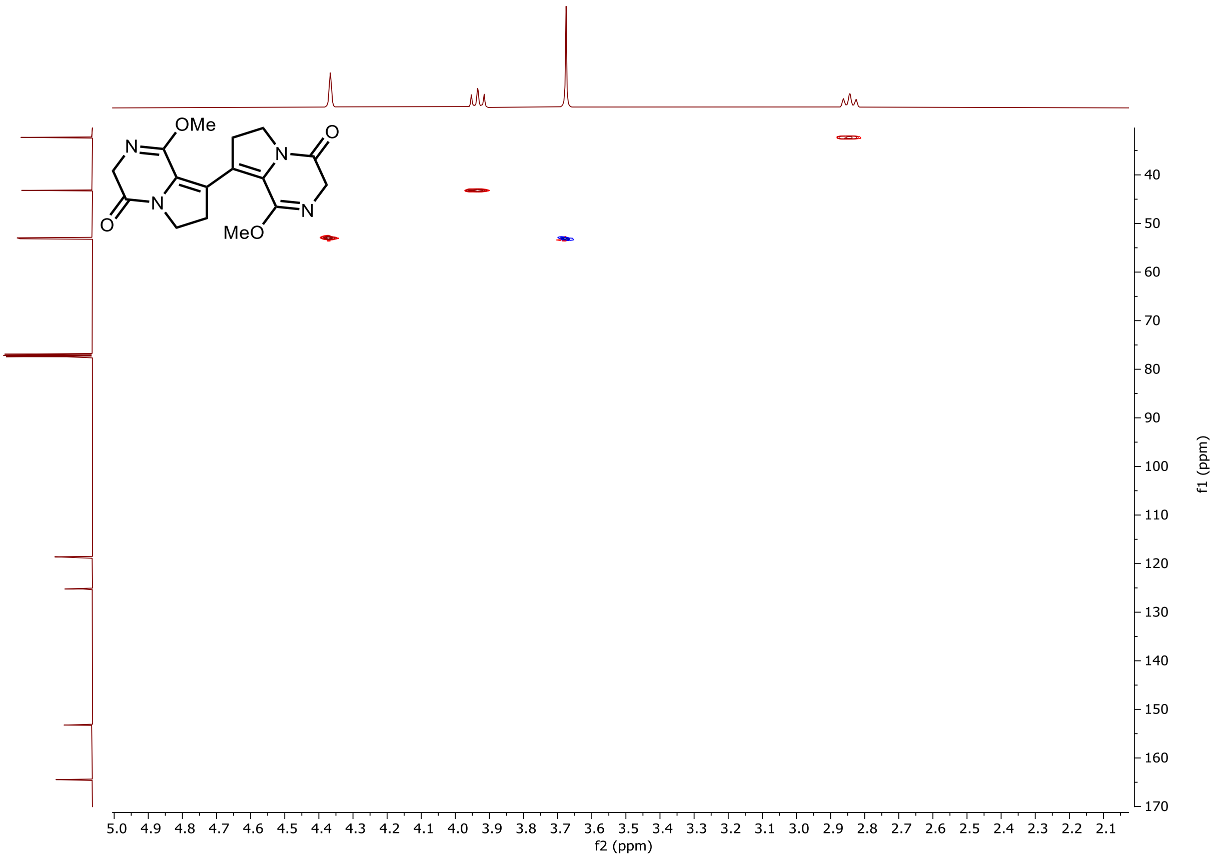

2.58. ^{1}H-^{13}C HMBC Spectrum for Bis-Lactim Ether 16 ($CDCl_3$)

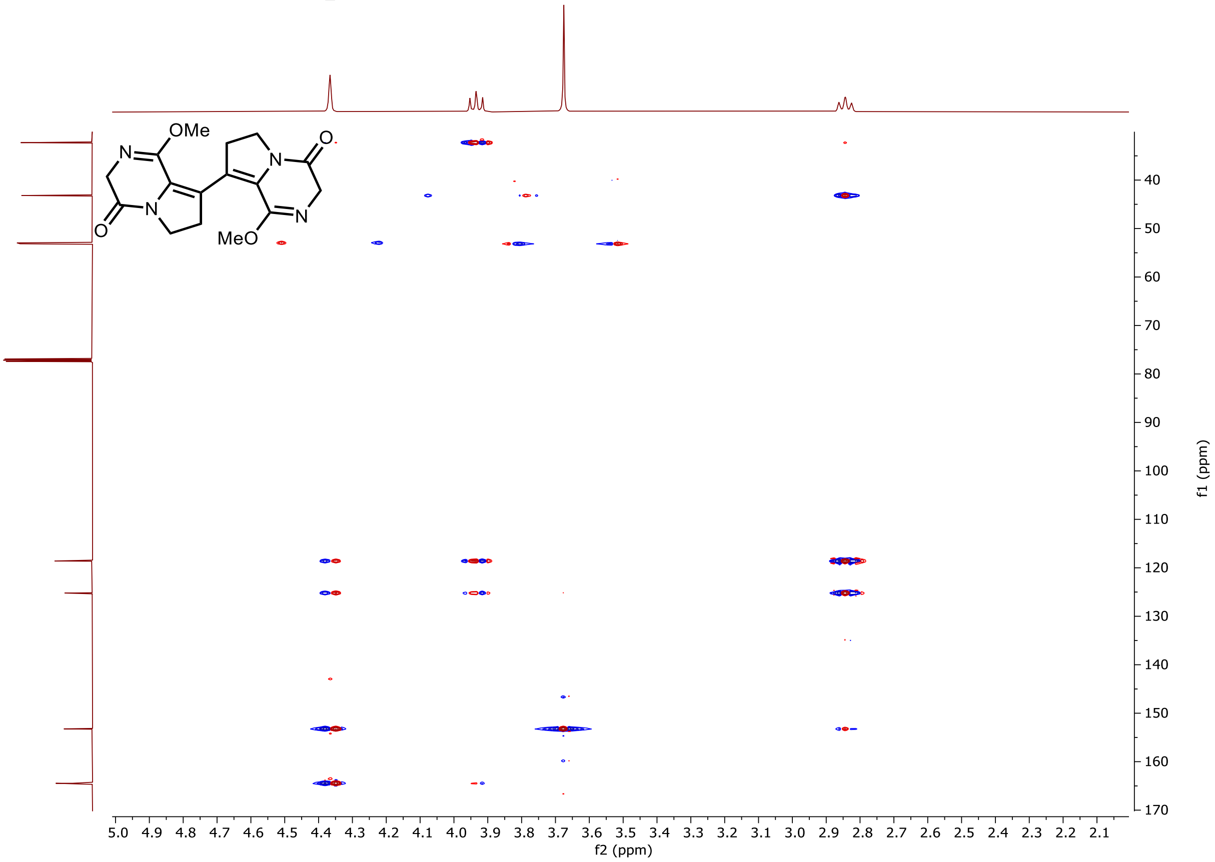

2.59. ^{1}H NMR Spectrum for 3-chloroindole (600 MHz, $CDCl_3$)

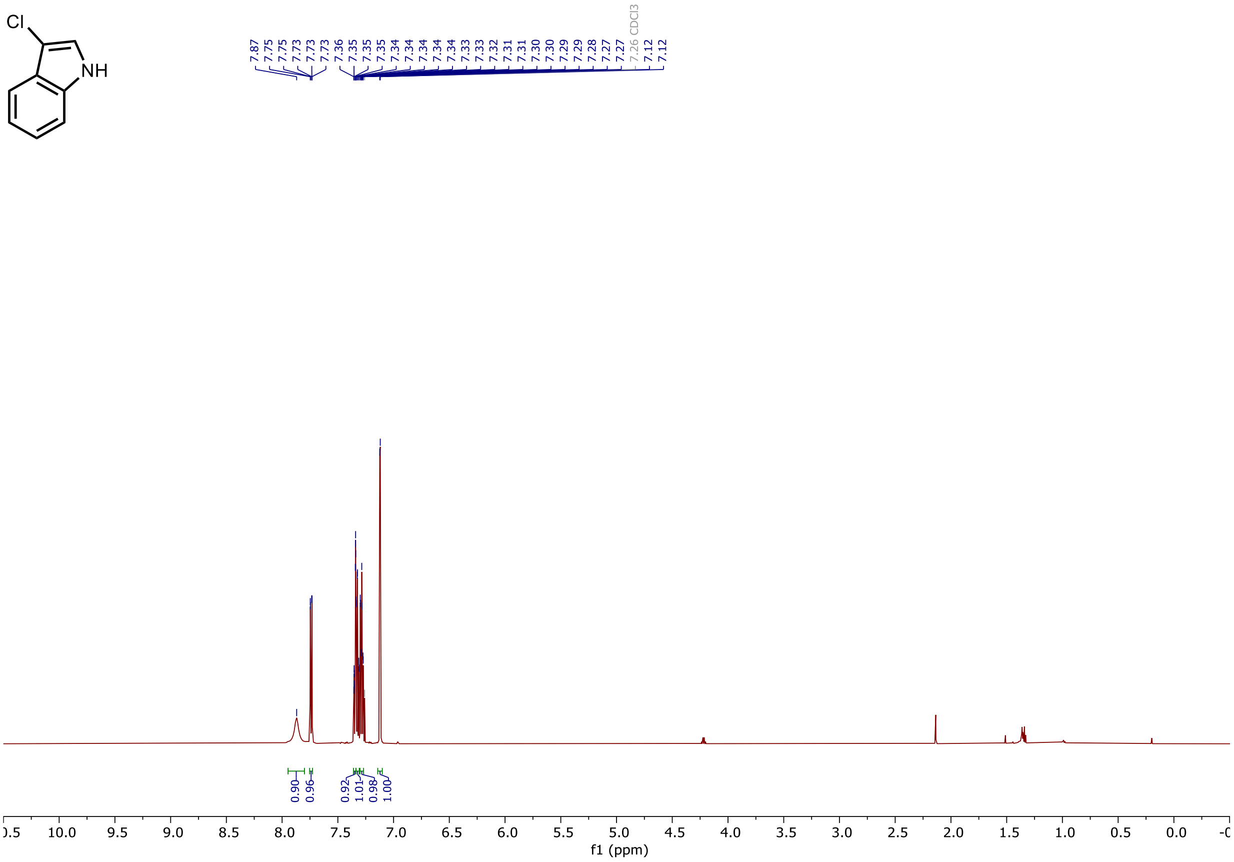

2.60. ^{13}C NMR Spectrum for 3-chloroindole (151 MHz, $CDCl_3$)

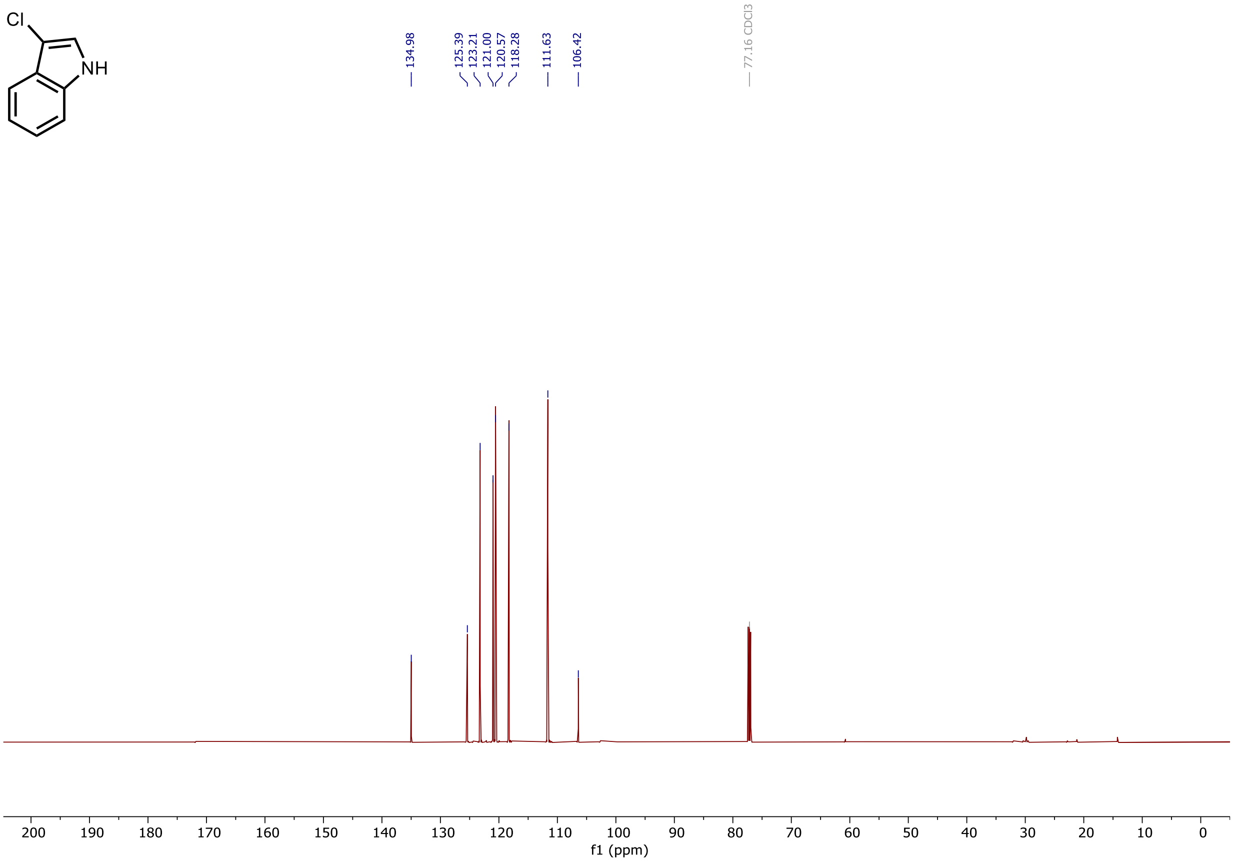

2.61. ^{1}H NMR Spectrum for 3-methyl-1,2-butadiene (500 MHz, $CDCl_3$)

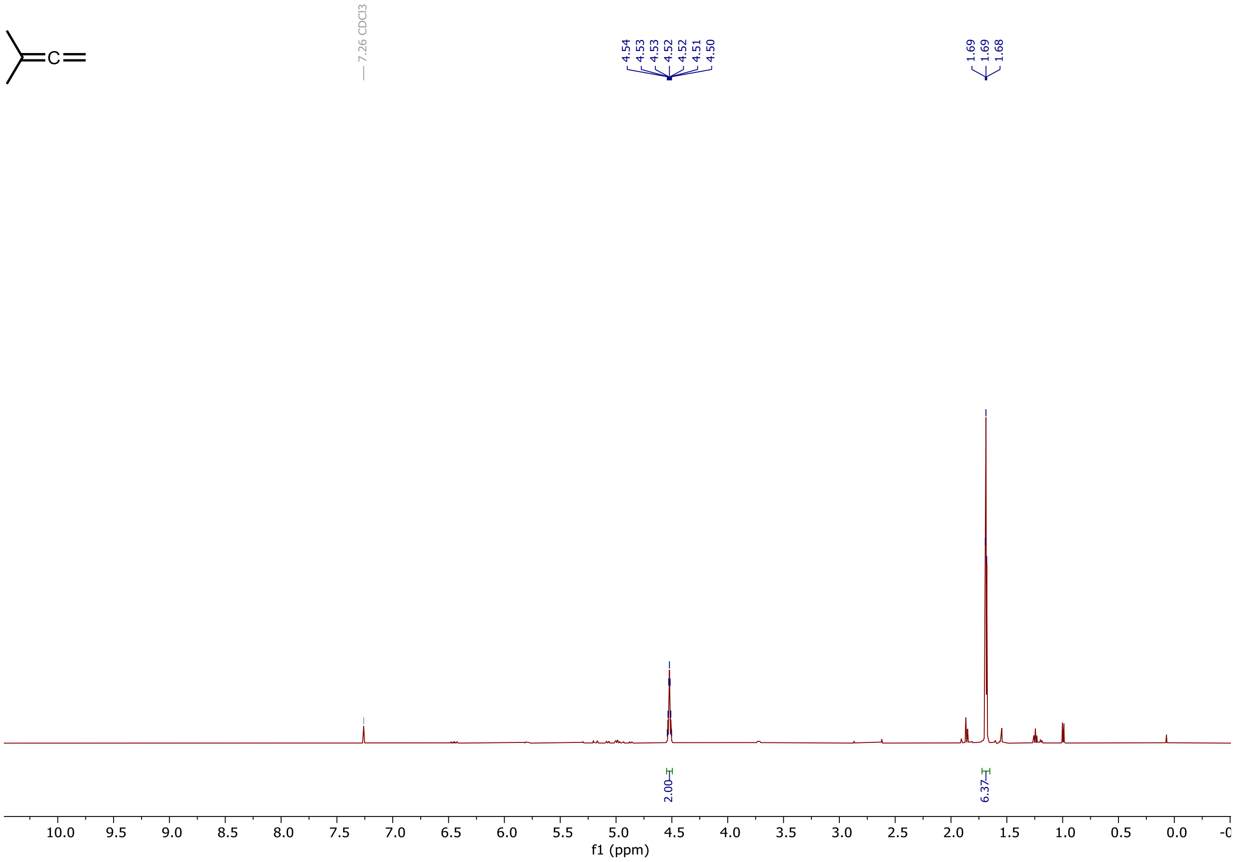

2.62. ^{13}C NMR Spectrum for 3-methyl-1,2-butadiene (126 MHz, $CDCl_3$)

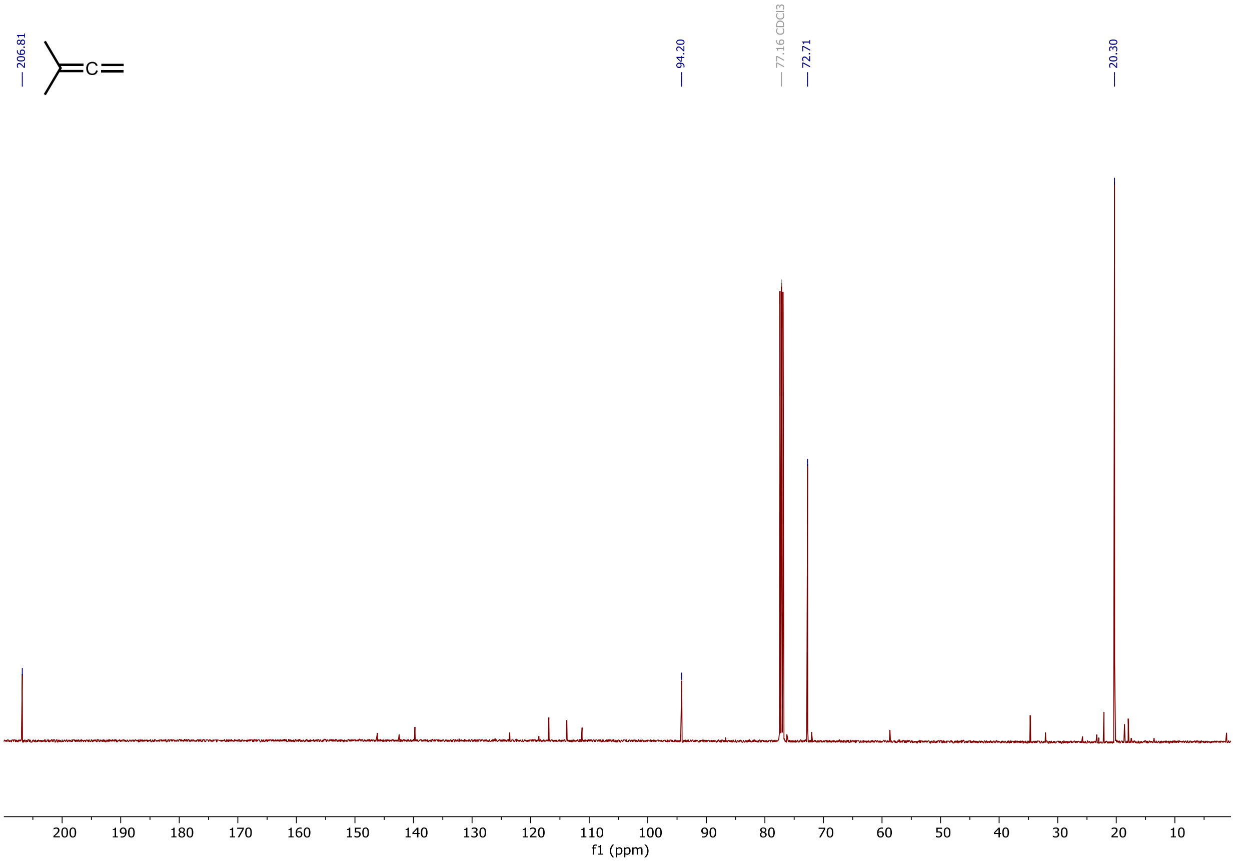

2.63. ^{1}H NMR Spectrum for Indole S-1 (500 MHz, $CDCl_3$)

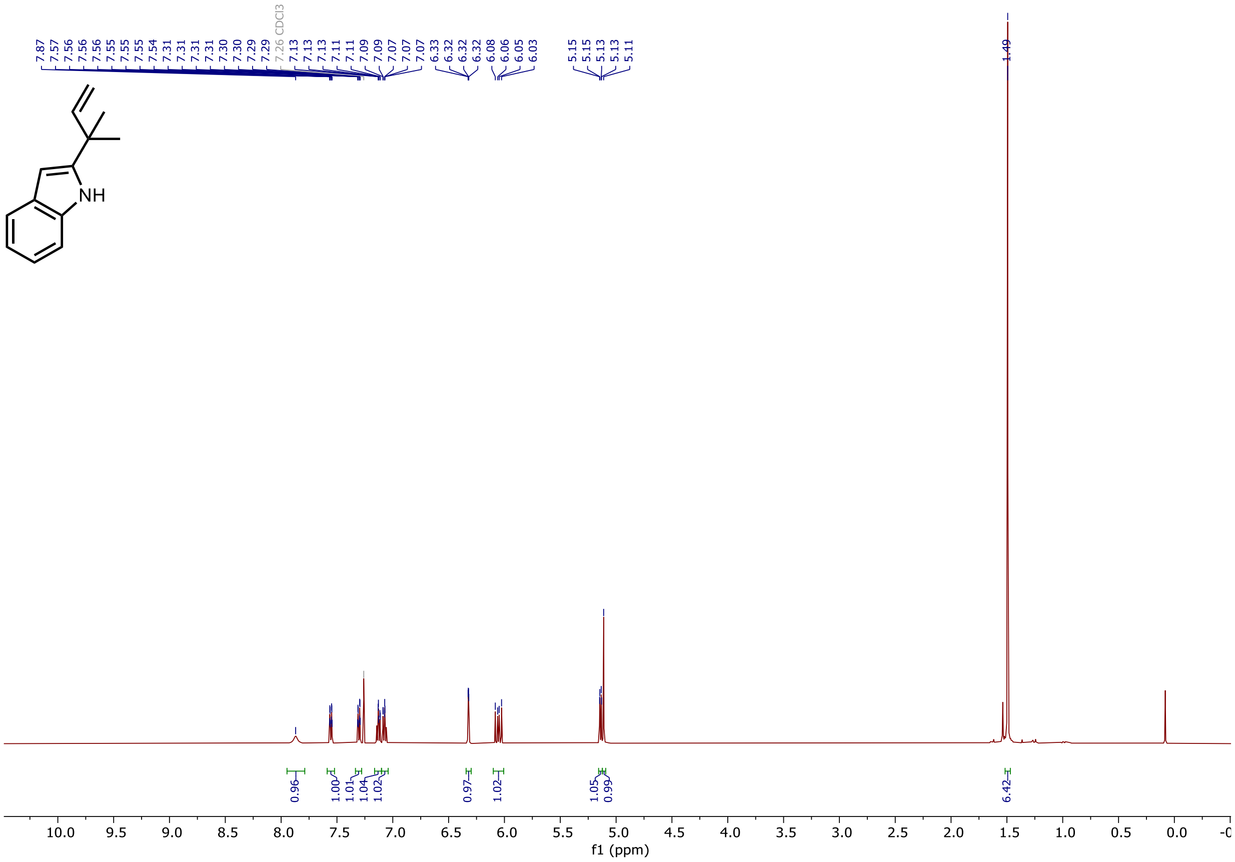

2.64. ^{13}C NMR Spectrum for Indole S-1 (126 MHz, $CDCl_3$)

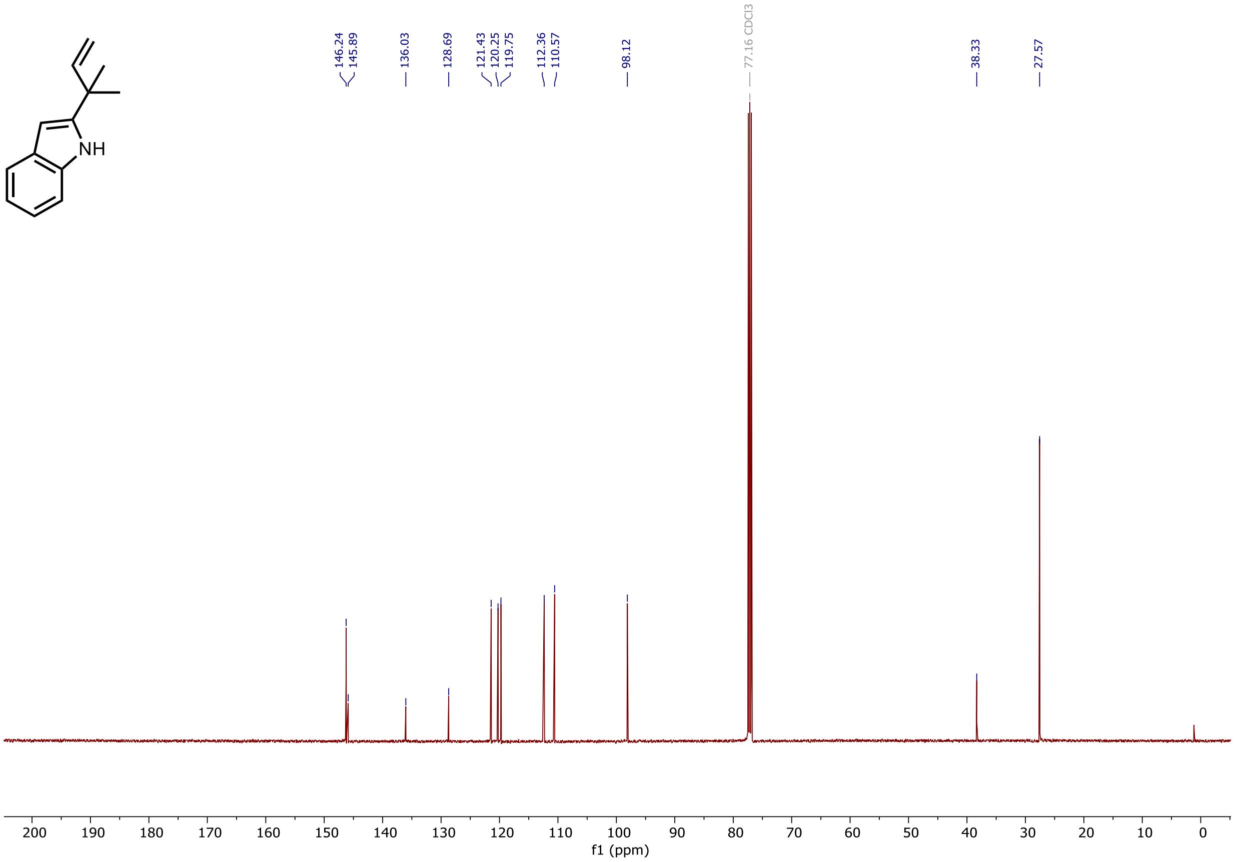

2.65. ^{1}H NMR Spectrum for Aldehyde 3 (600 MHz, $CDCl_3$)

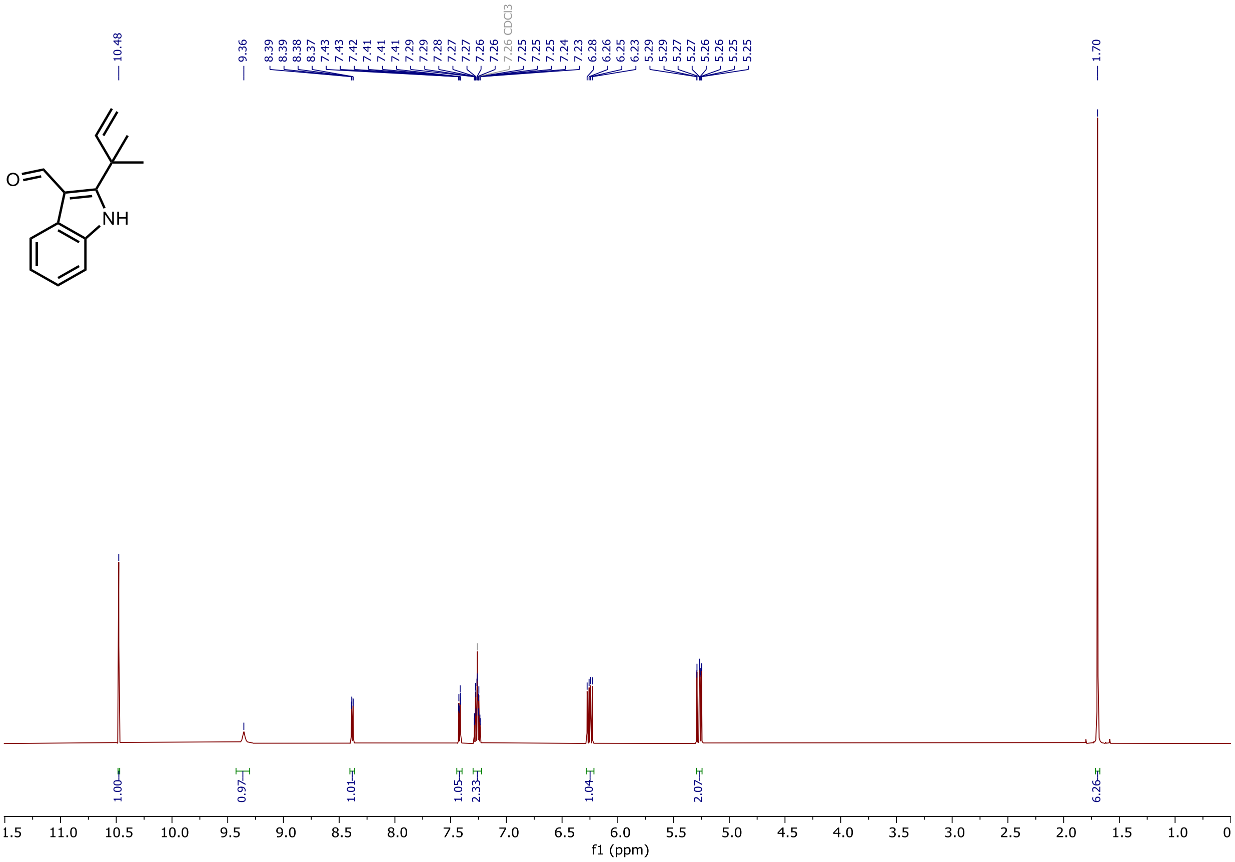

2.66. ^{13}C NMR Spectrum for Aldehyde 3 (151 MHz, $CDCl_3$)

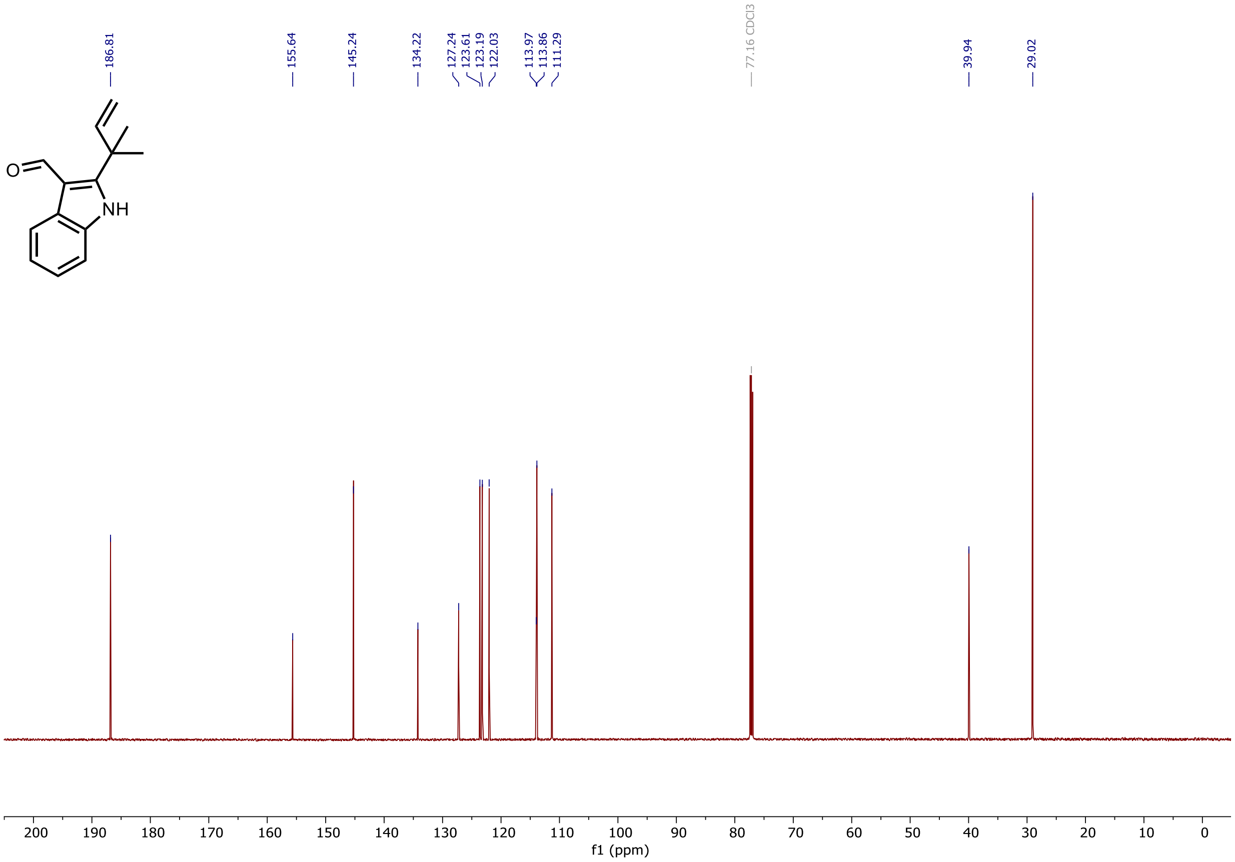

2.67. ^{1}H NMR Spectrum for Enone 23 (500 MHz, $CDCl_3$)

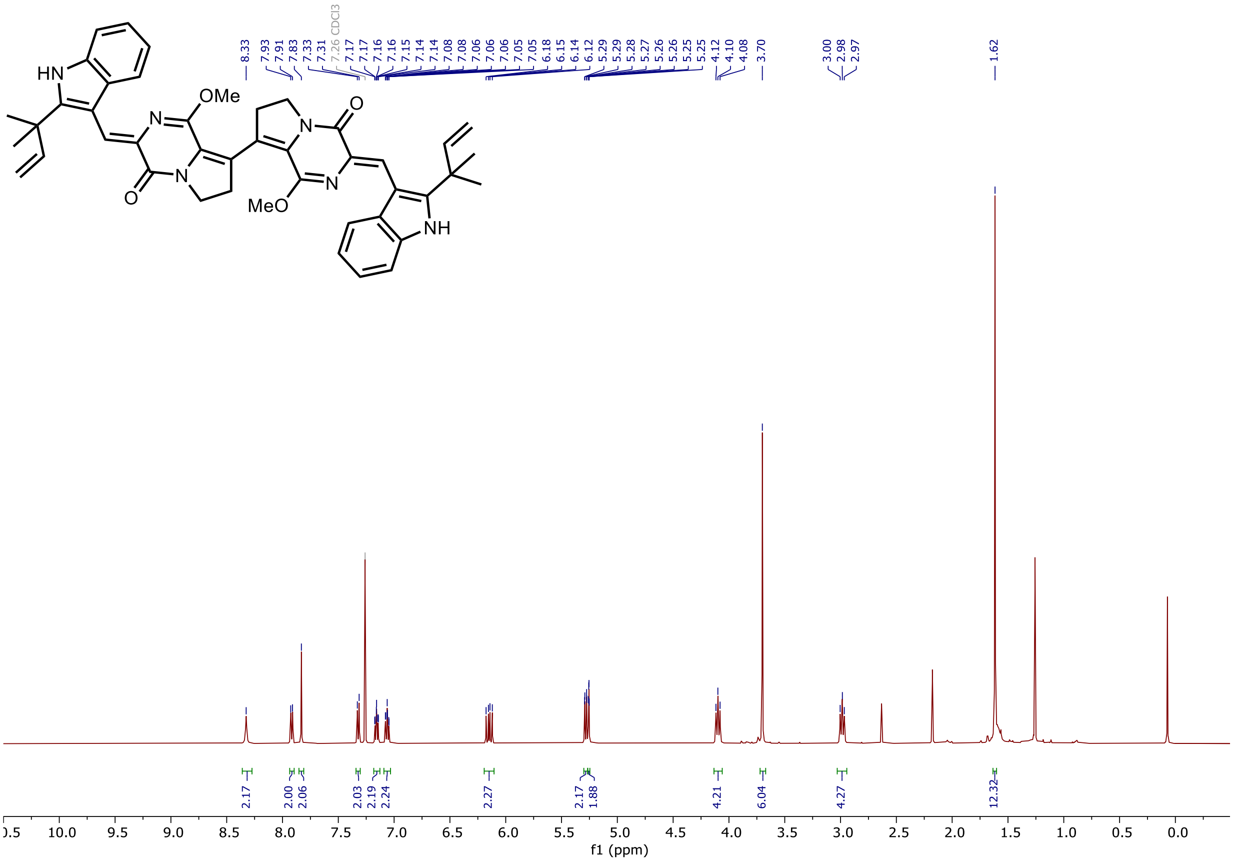

2.68. ^{13}C NMR Spectrum for Enone 23 (126 MHz, $CDCl_3$)

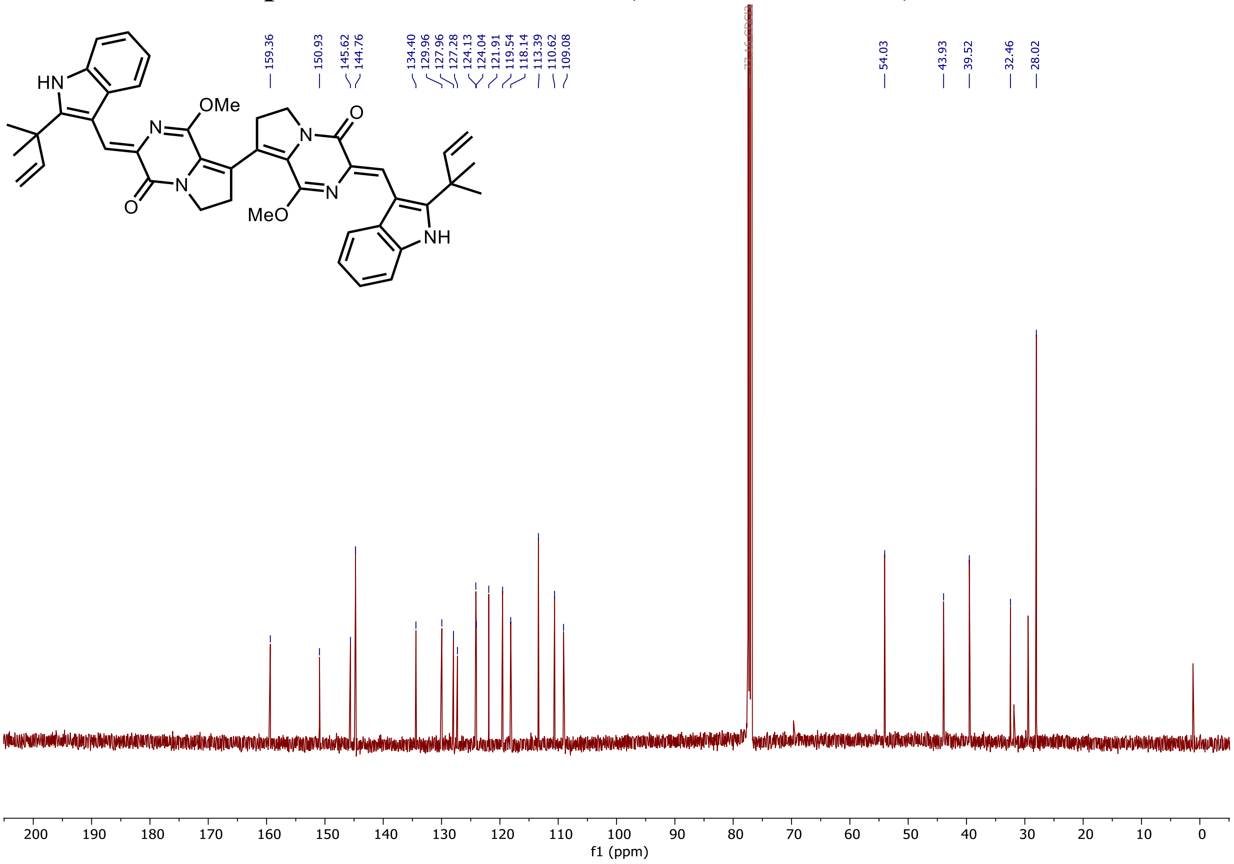

2.69. ^{1}H-^{1}H COSY Spectrum for Enone 23 ($CDCl_3$)

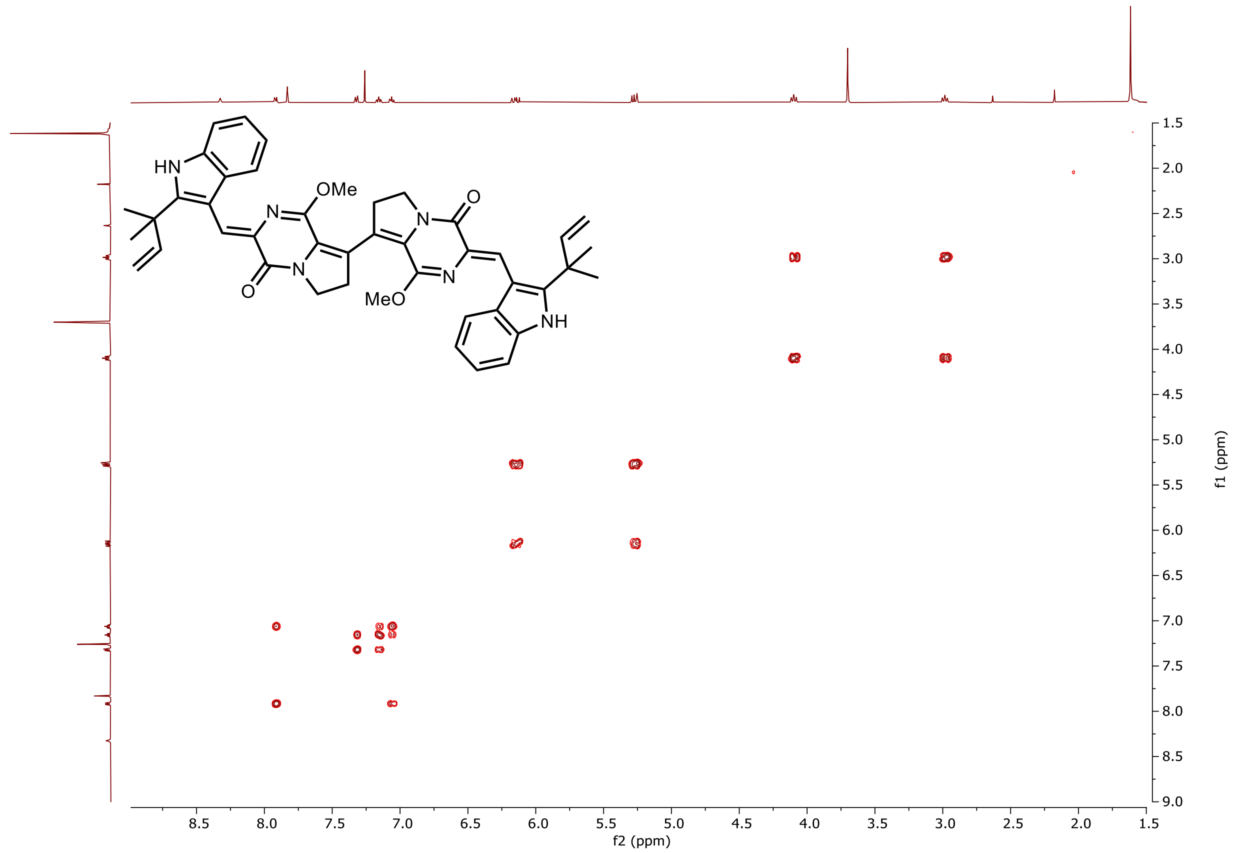

2.70. ^{1}H-^{13}C HSQC Spectrum for Enone 23 ($CDCl_3$)

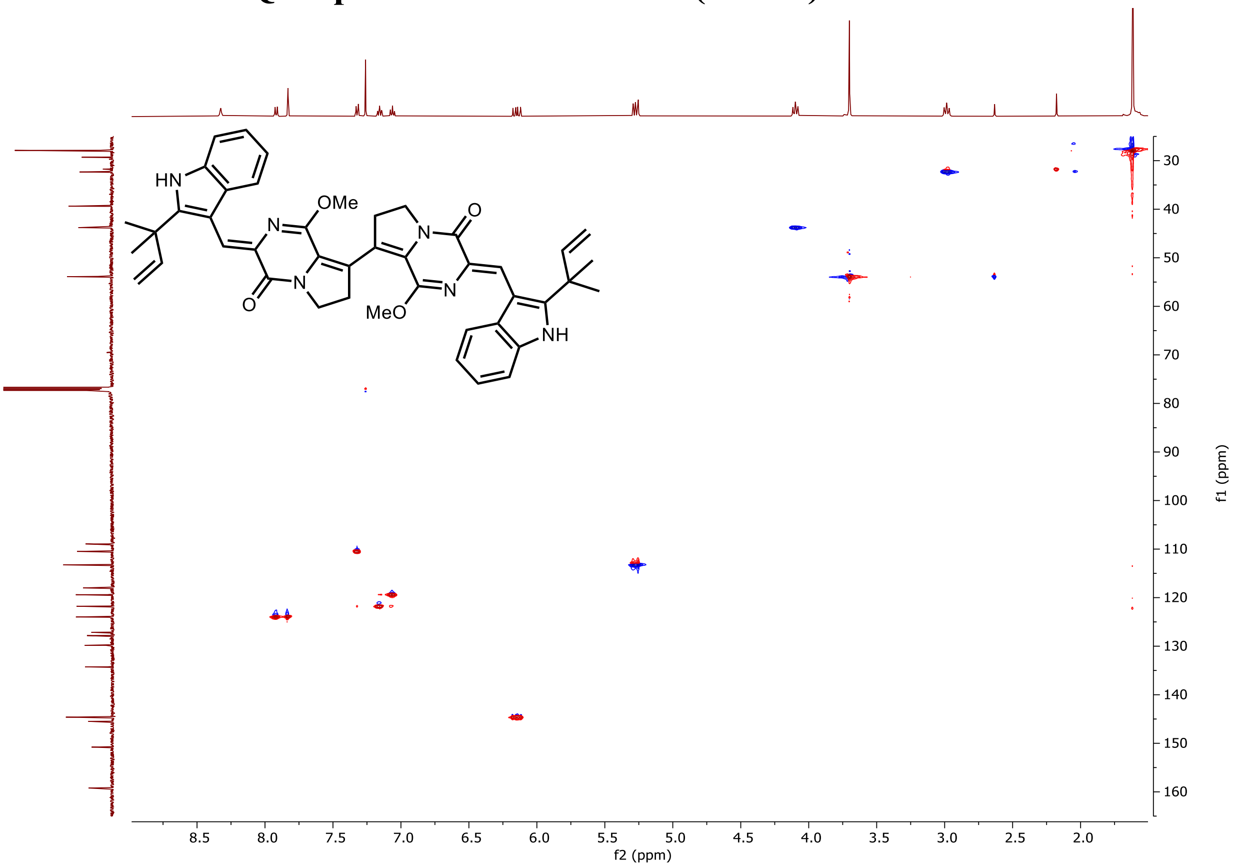

2.71. ^{1}H-^{13}C HMBC Spectrum for Enone 23 ($CDCl_3$)

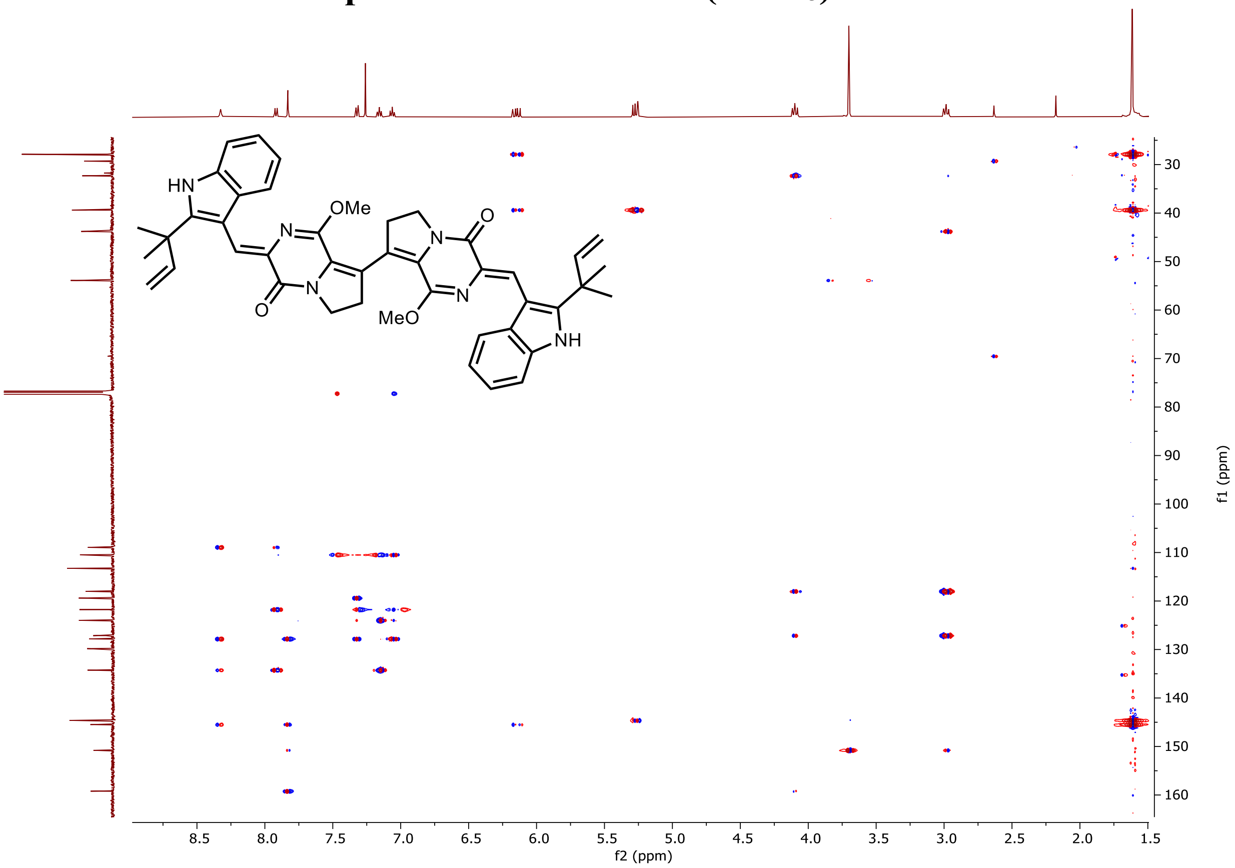

2.72. ^{1}H NMR Spectrum for Enone 22 (500 MHz, $CDCl_3$)

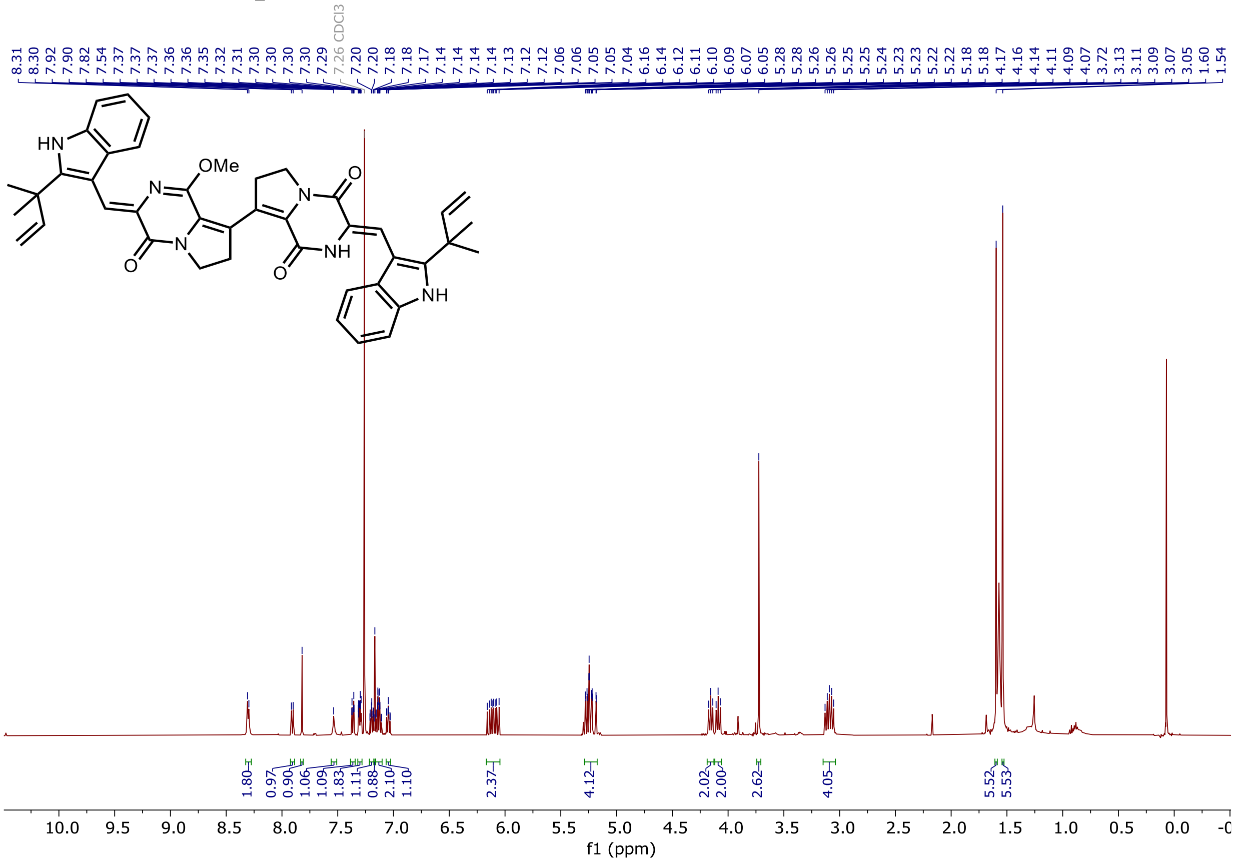

2.73. ^{13}C NMR Spectrum for Enone 22 (126 MHz, $CDCl_3$)

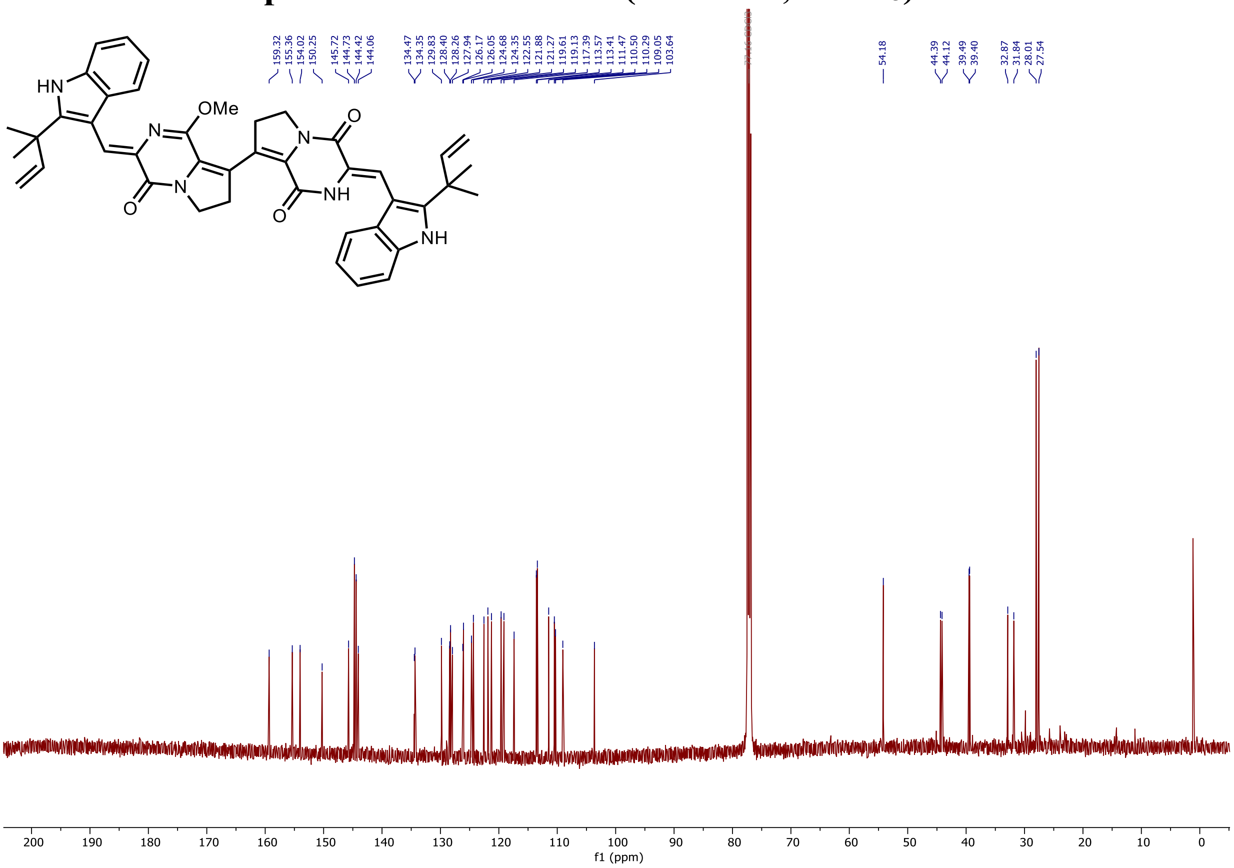

2.74. ^{1}H-^{1}H COSY Spectrum for Enone 22 ($CDCl_3$)

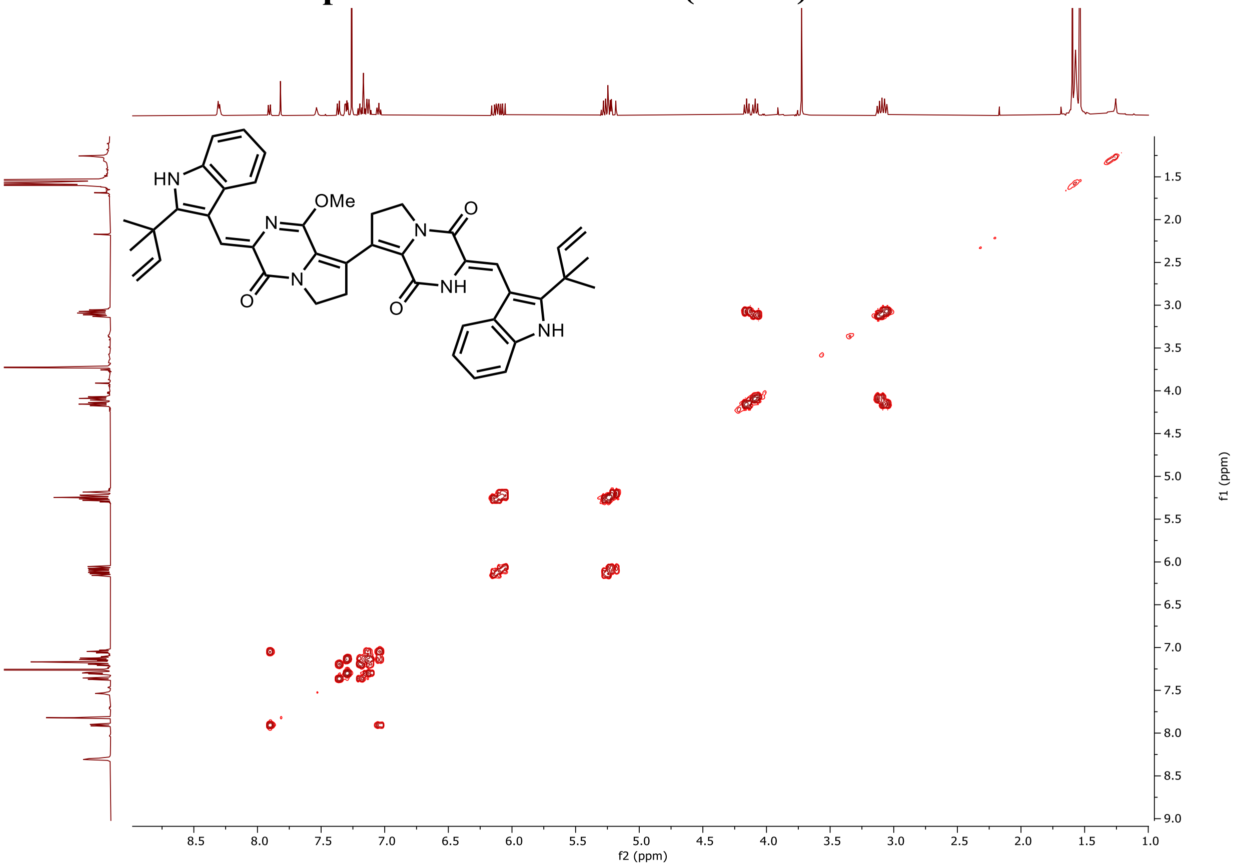

2.75. ^{1}H-^{13}C HSQC Spectrum for Enone 22 ($CDCl_3$)

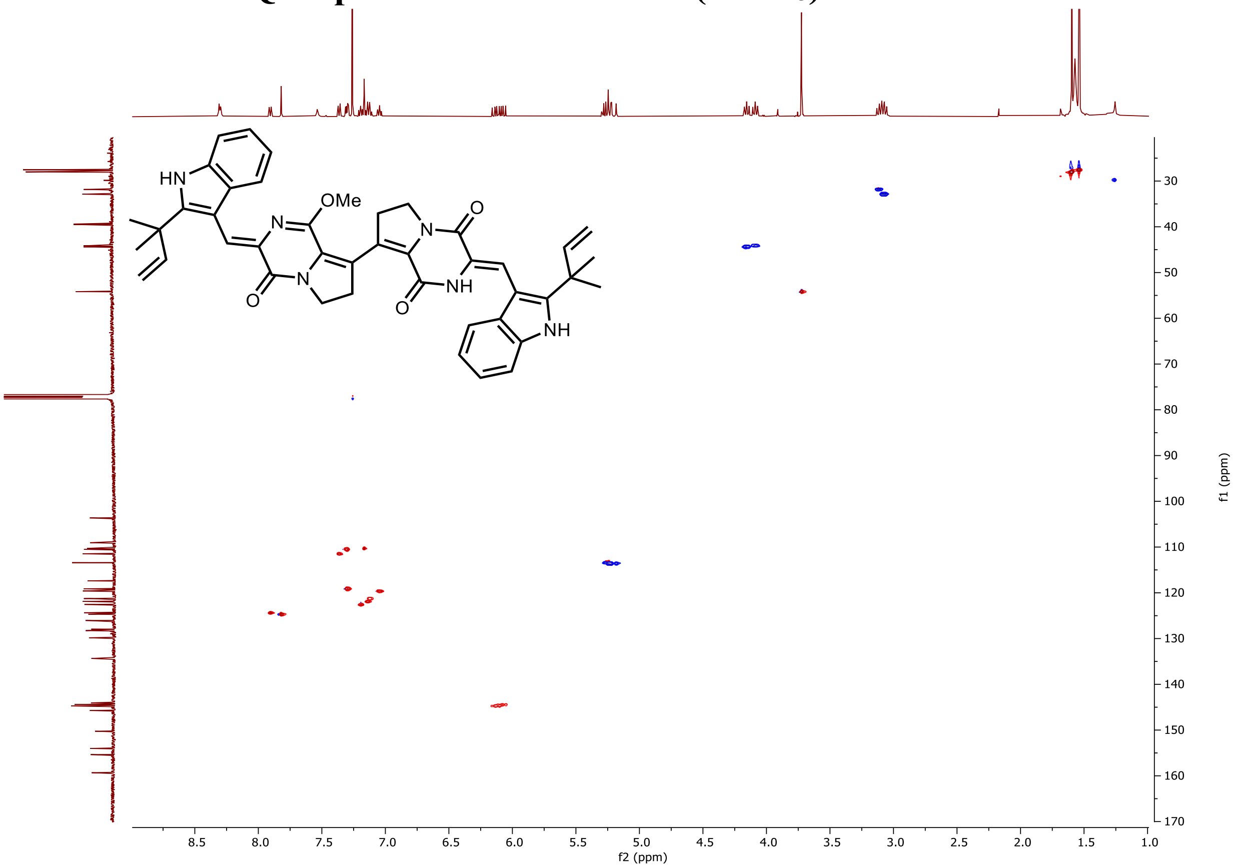

2.76. ^{1}H-^{13}C HMBC Spectrum for Enone 22 ($CDCl_3$)

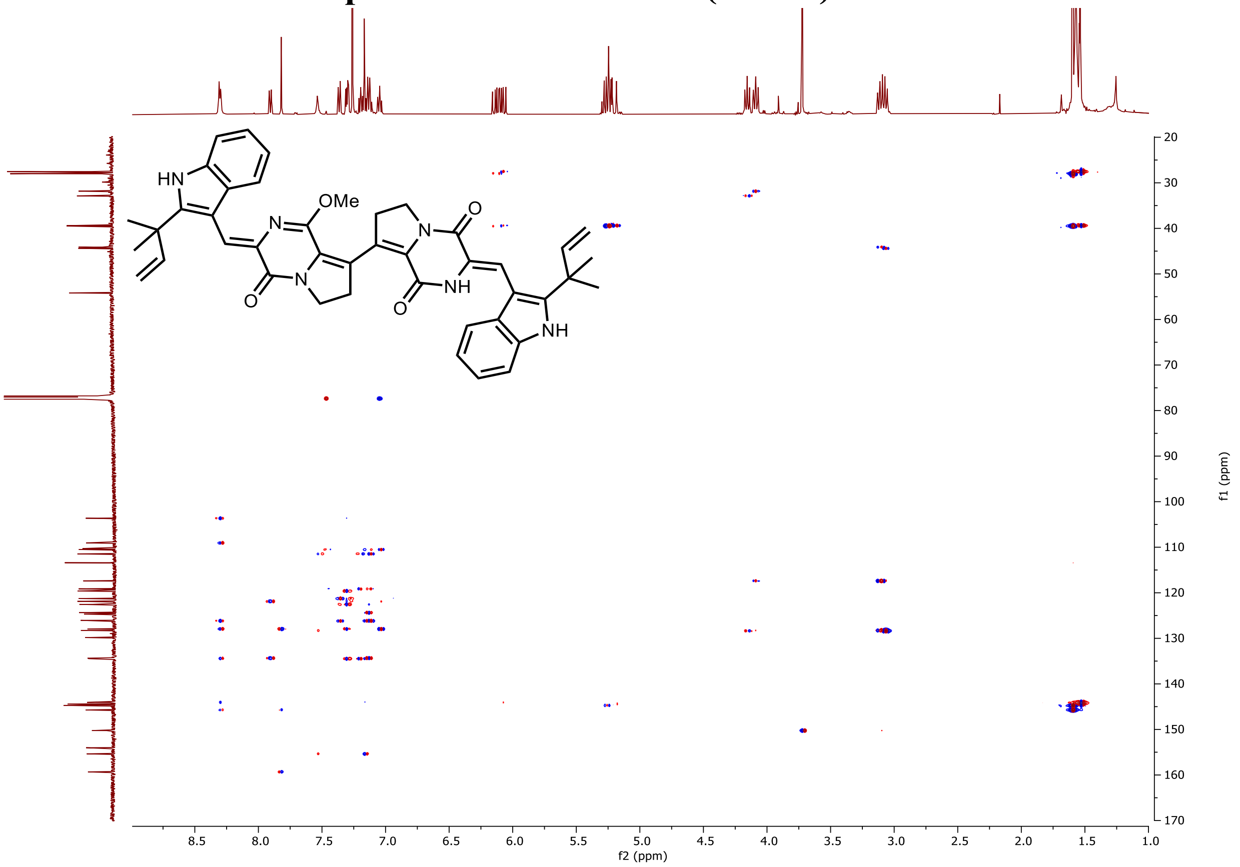

2.77. ^1H-^{13}C HMBC Spectrum for Enone 22 ($CDCl_3$, modified for 5 Hz coupling)

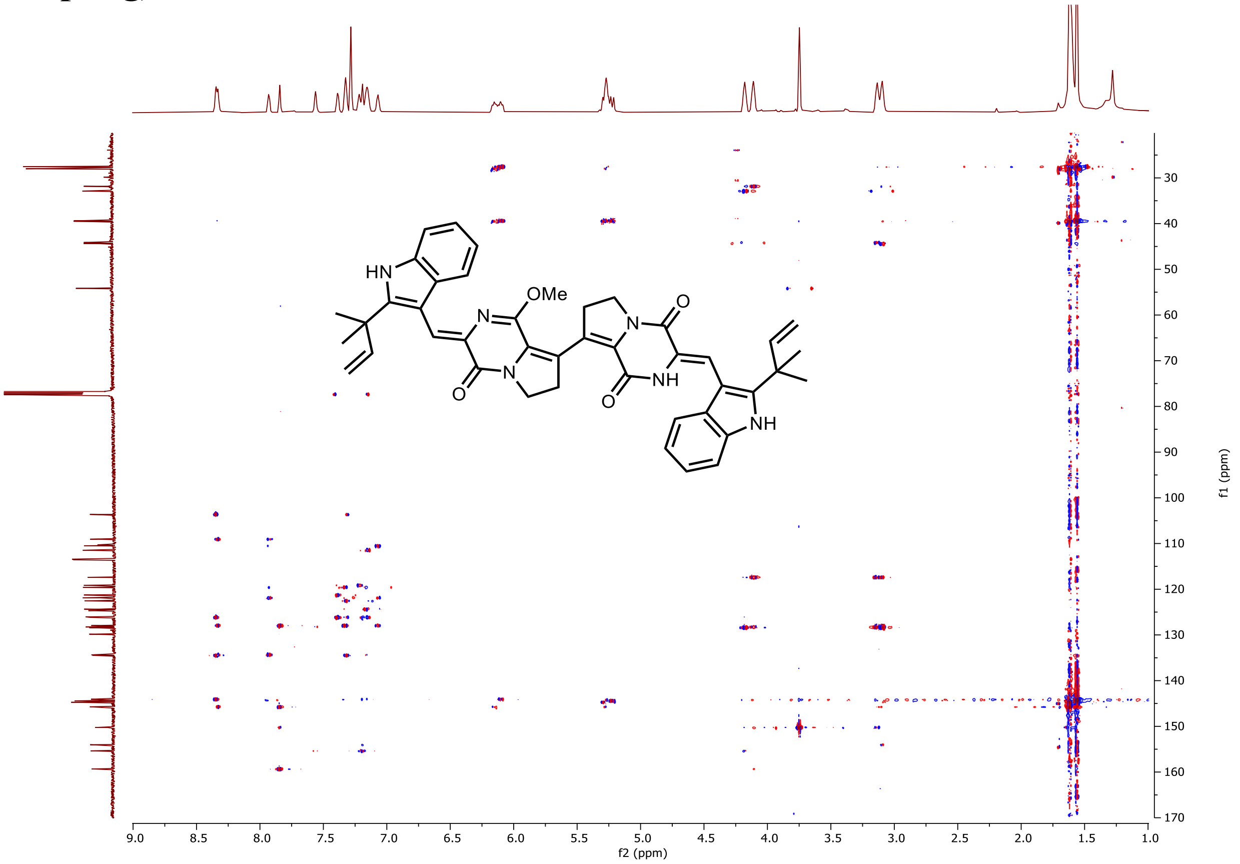

2.78. ^1H-^{13}C HMBC Spectrum for Enone 22 ($CDCl_3$, modified for 2 Hz coupling)

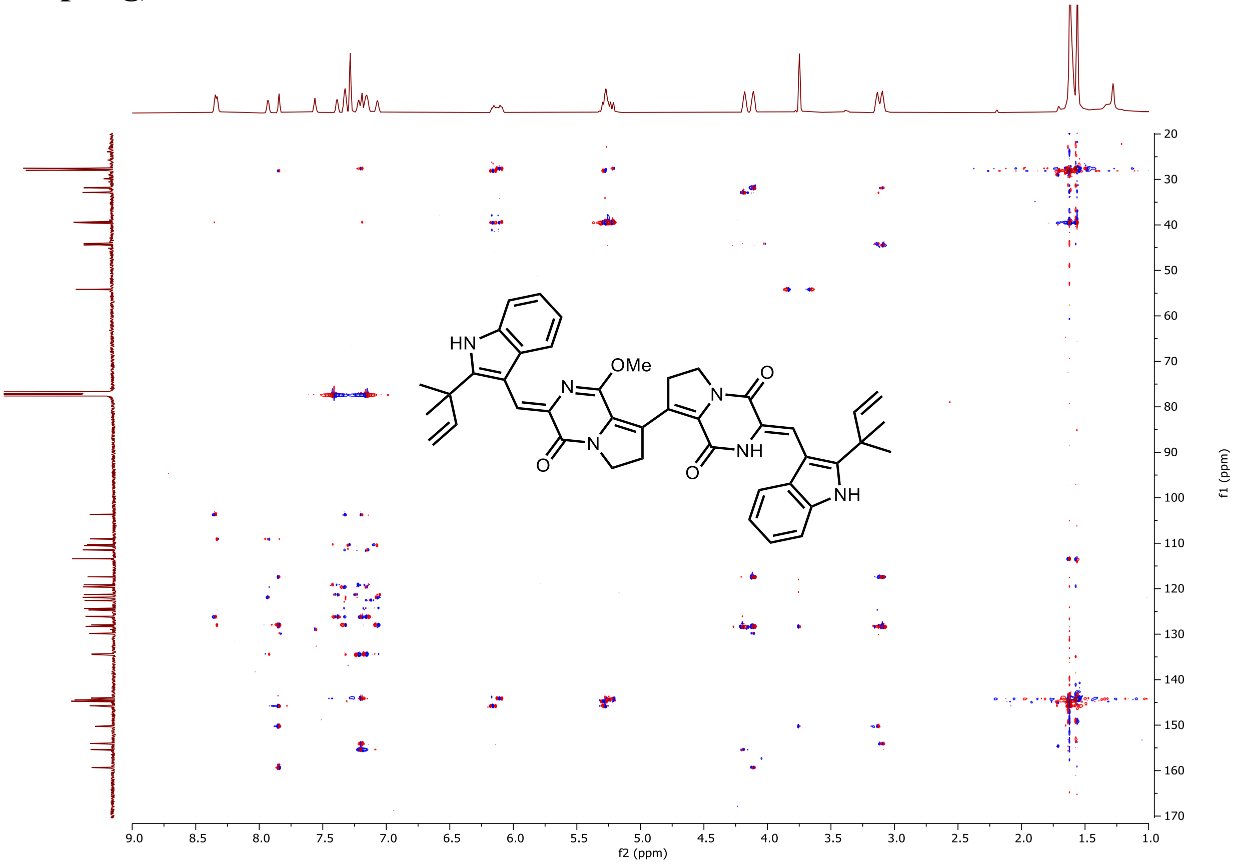

2.79. ^{1}H-^{1}H NOESY Spectrum for Enone 22 ($CDCl_3$)

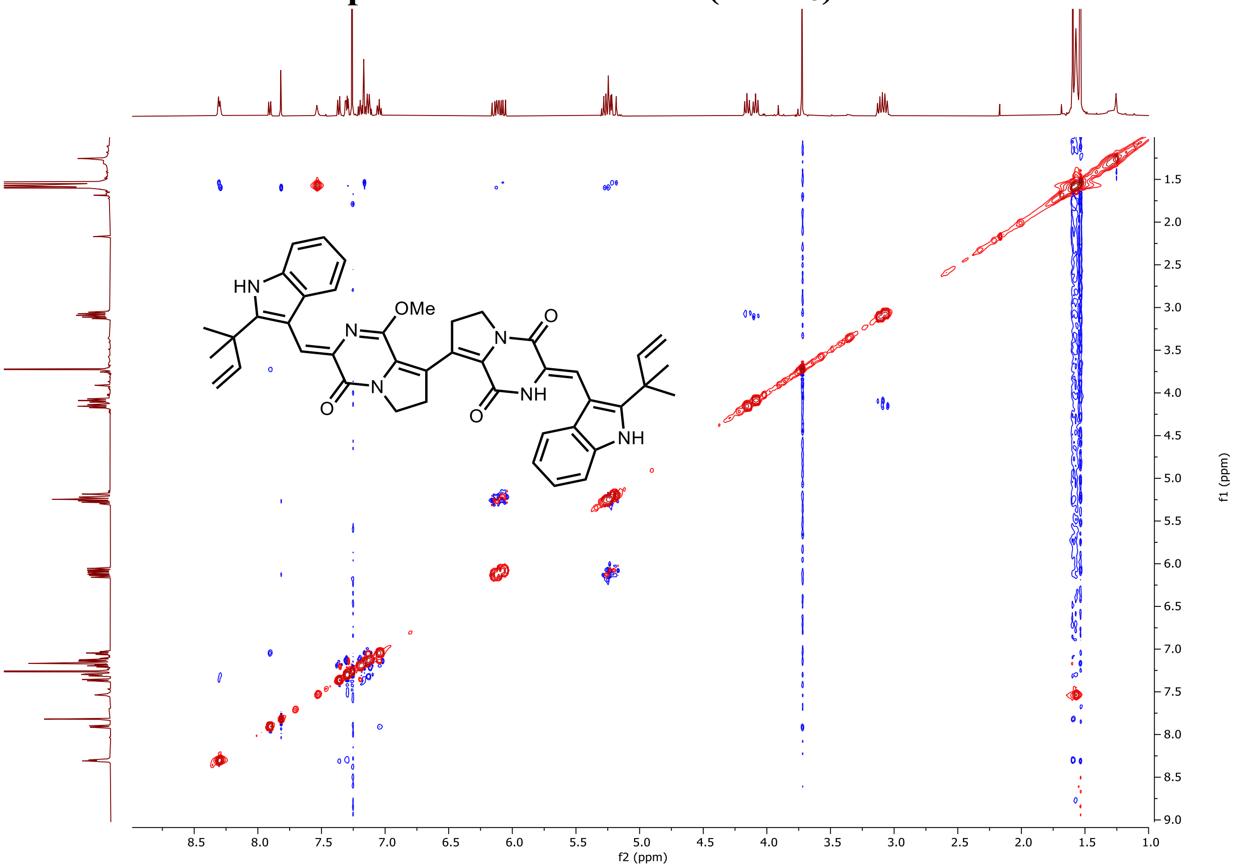

2.80. ^{1}H-^{15}N HMBC Spectrum for Enone 22 ($CDCl_3$)

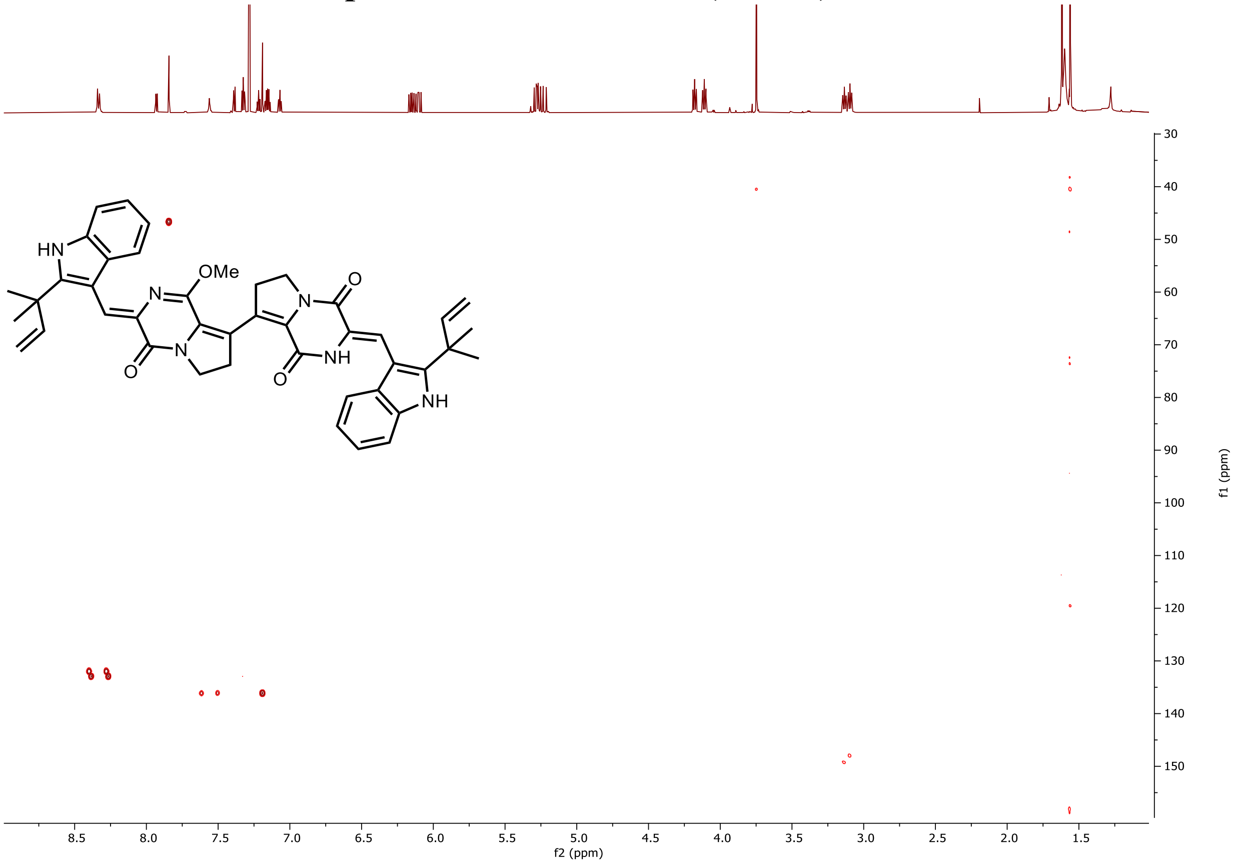

2.81. ^{1}H NMR Spectrum for Brevianamide S (1) (500 MHz, $CDCl_3$)

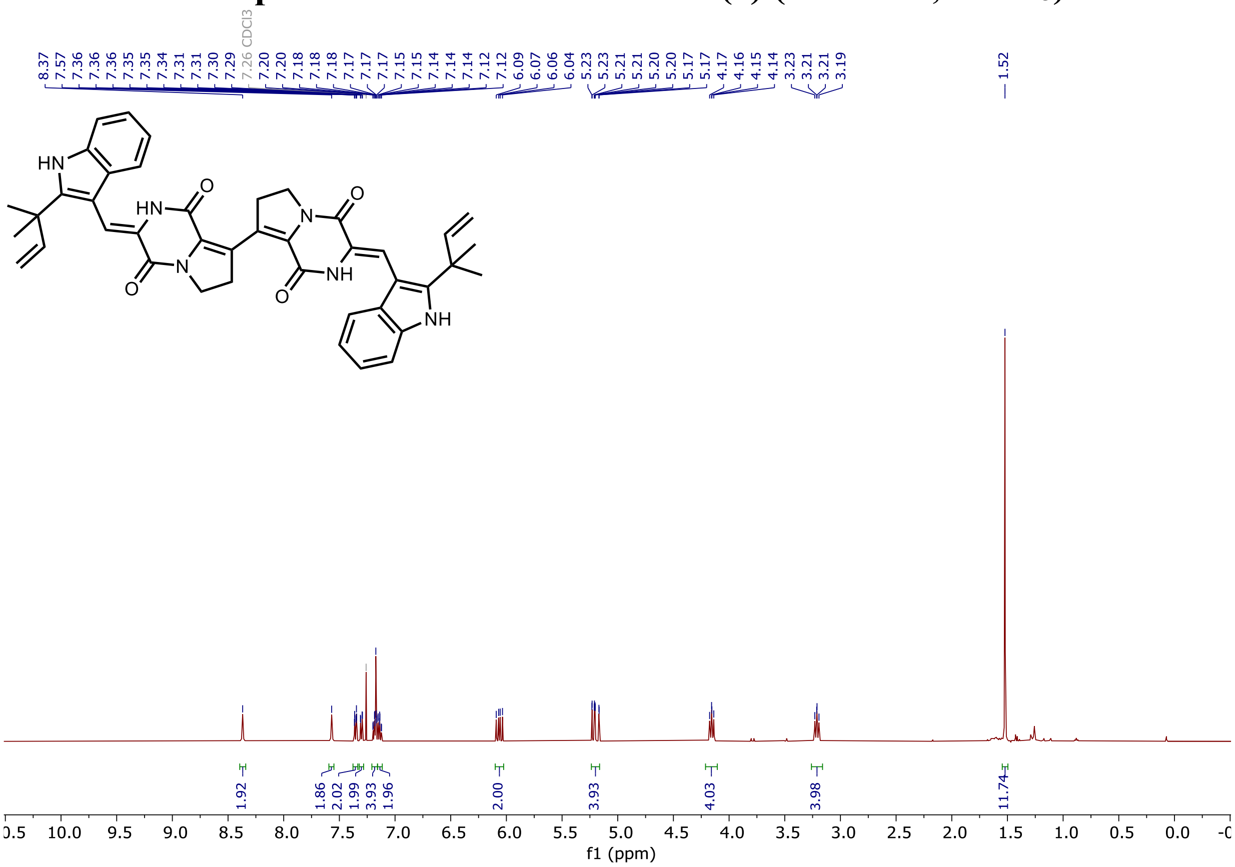

2.82. ^{13}C NMR Spectrum for Brevianamide S (1) (126 MHz, $CDCl_3$)

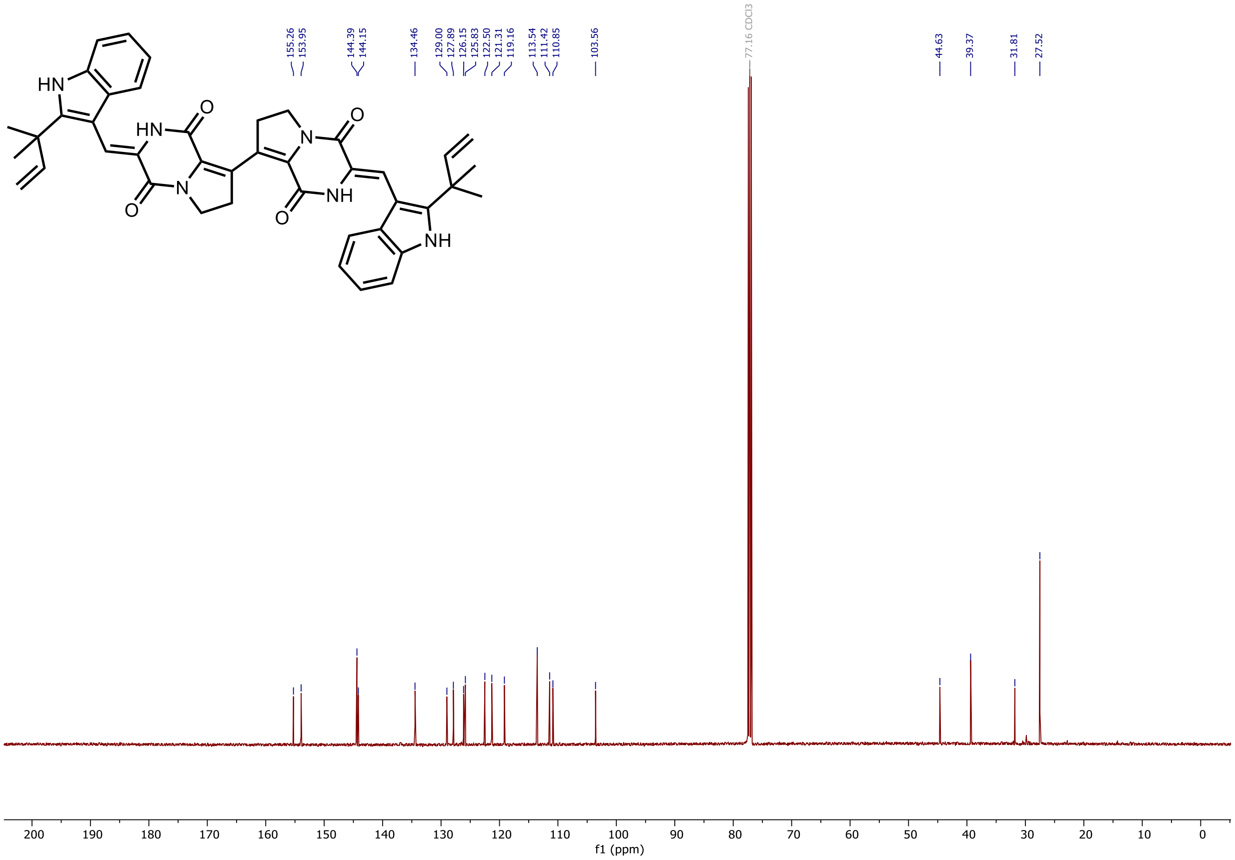

2.83. ¹H-¹H COSY Spectrum for Brevianamide S (1) (CDCl₃)

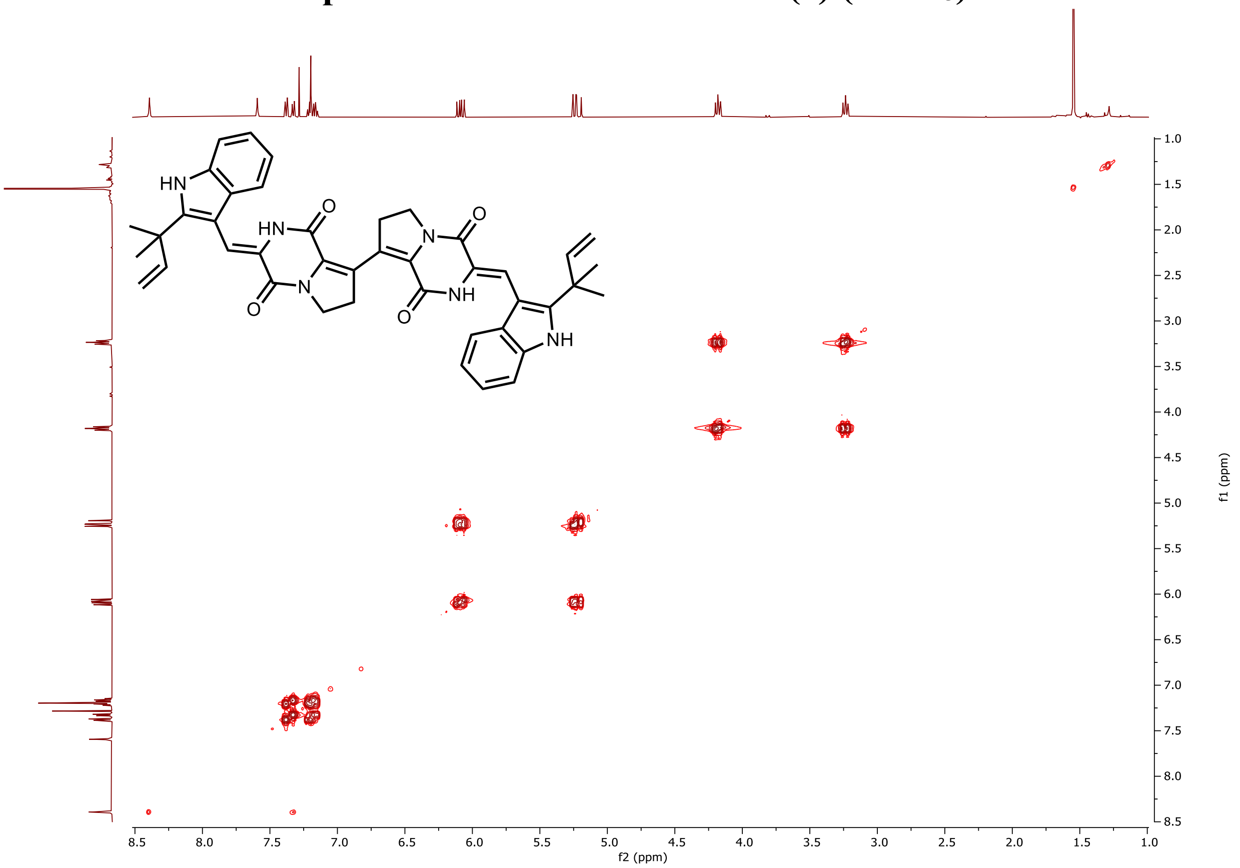

2.84. ¹H-¹³C HSQC Spectrum for Brevianamide S (1) (CDCl₃)

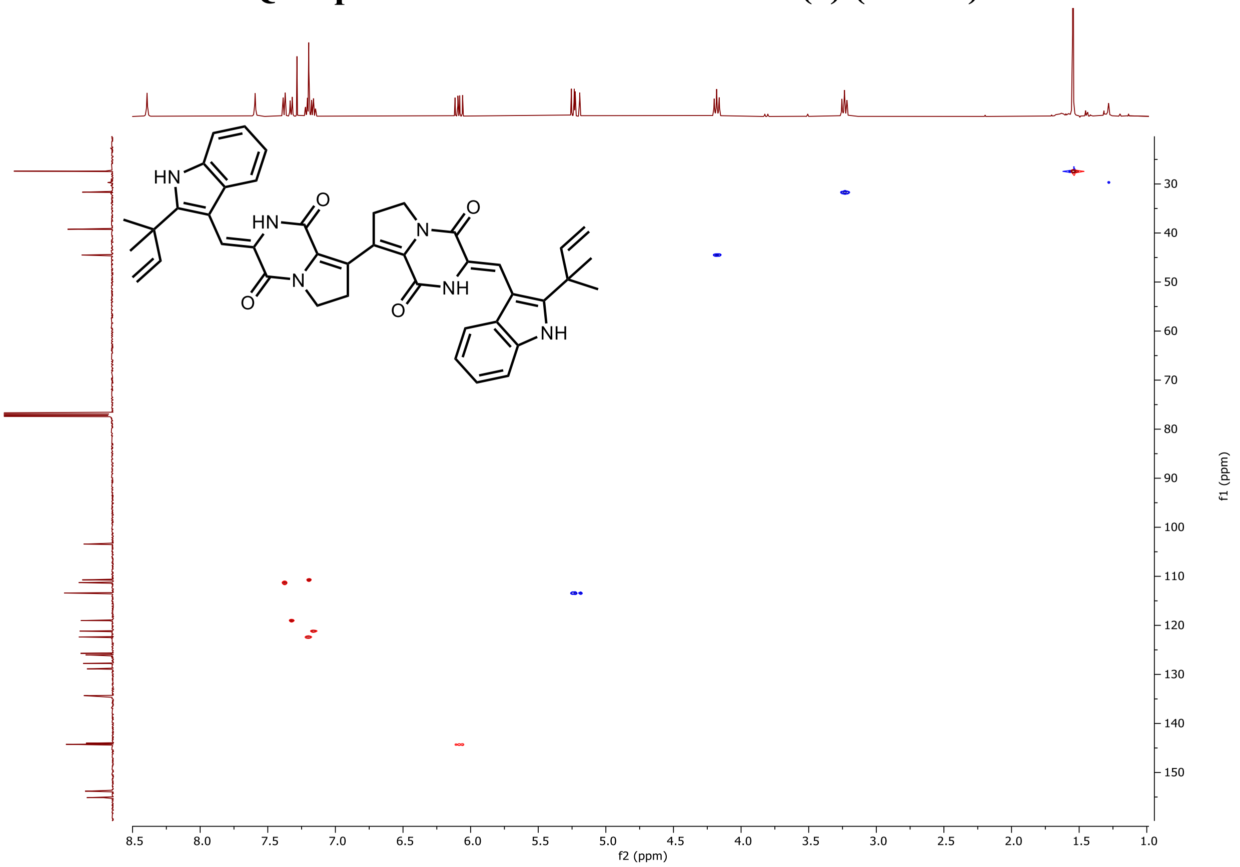

2.85. ^{1}H-^{13}C HMBC Spectrum for Brevianamide S (1) ($CDCl_3$)

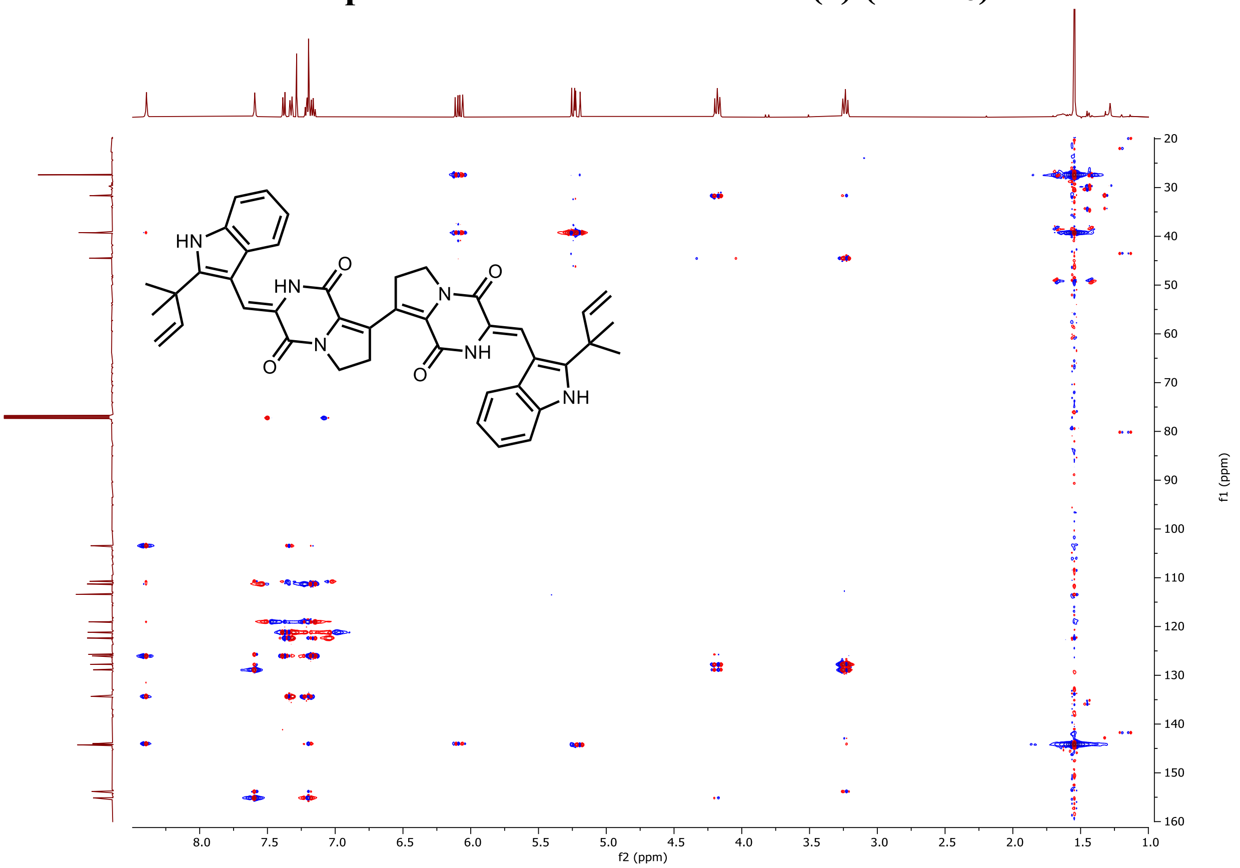

2.86. ^{1}H-^{1}H NOESY Spectrum for Brevianamide S (1) ($CDCl_3$)

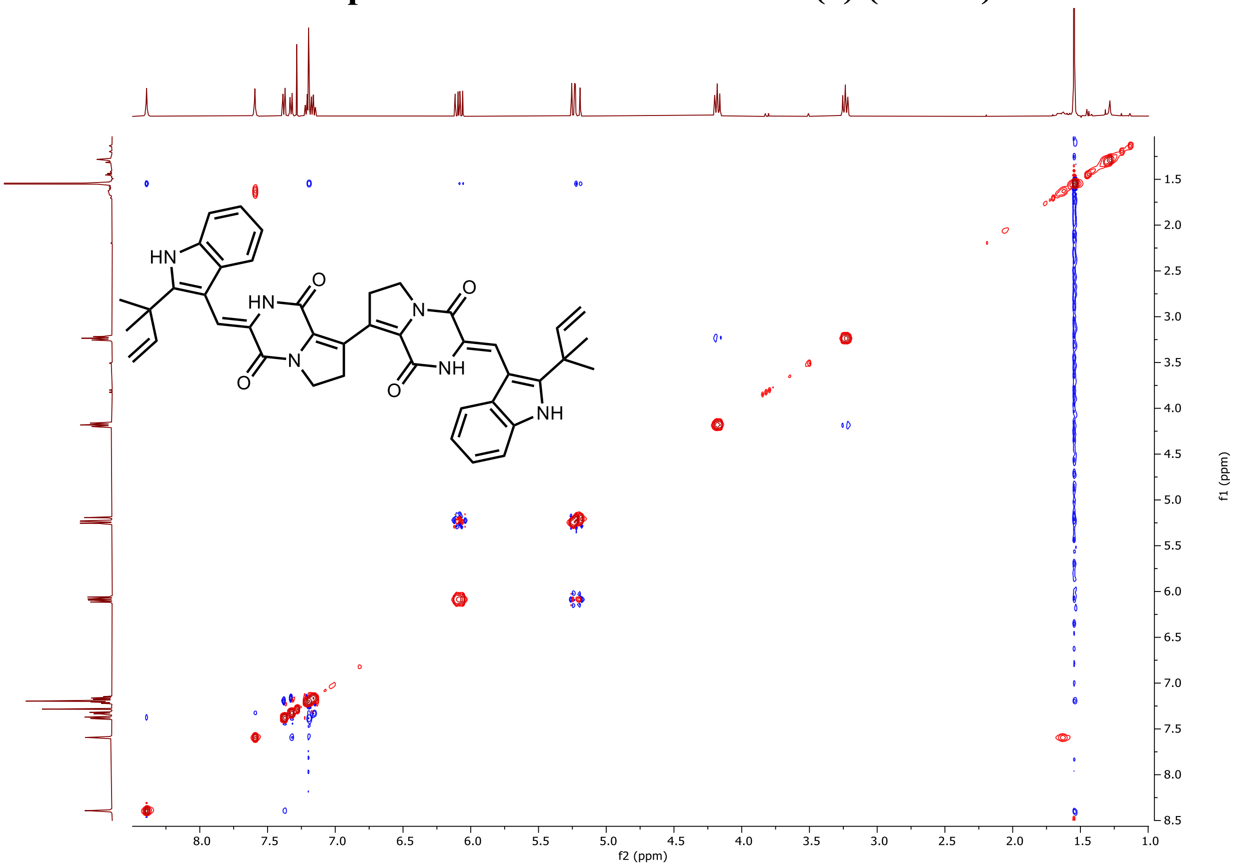

2.87. ^{1}H NMR Spectrum for Brevianamide S (1) (500 MHz, CD_3OD)

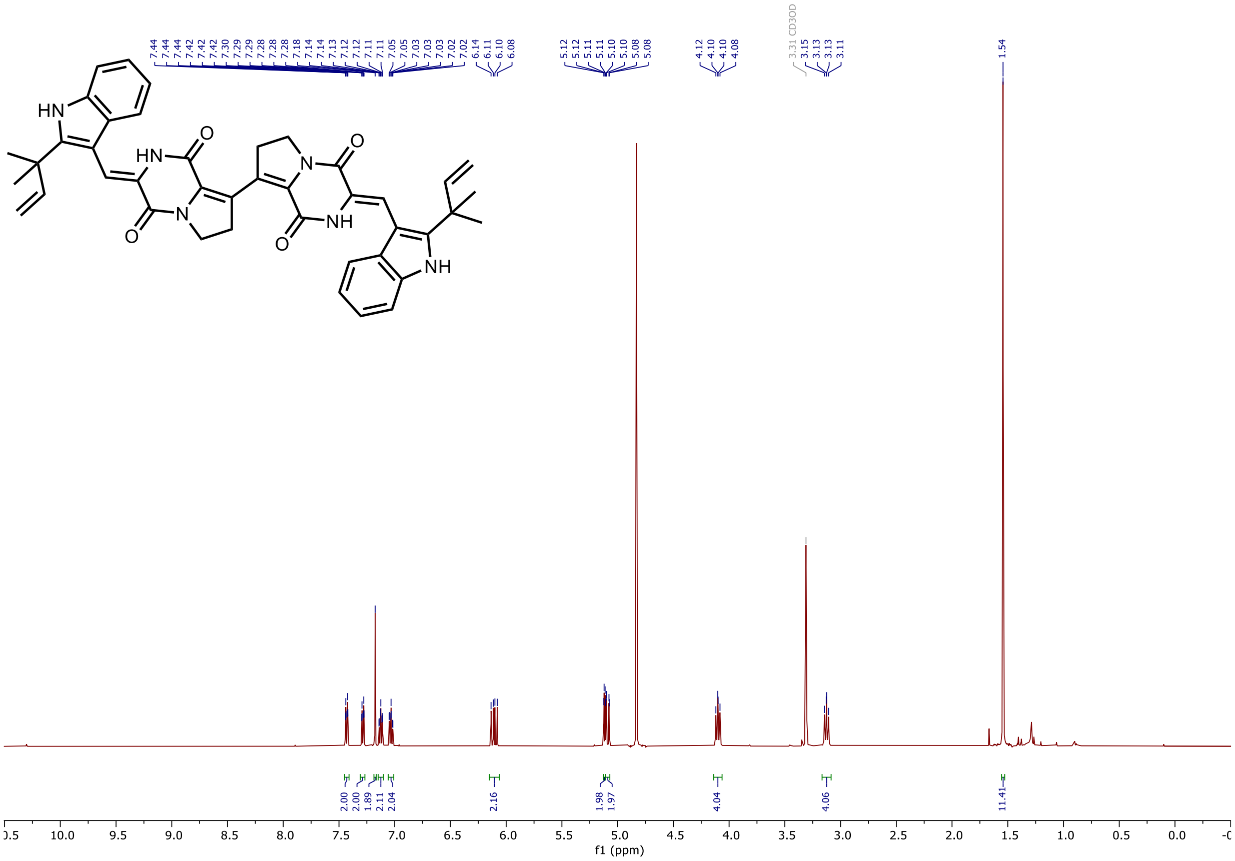

2.88. ^{13}C NMR Spectrum for Brevianamide S (1) (126 MHz, CD_3OD)

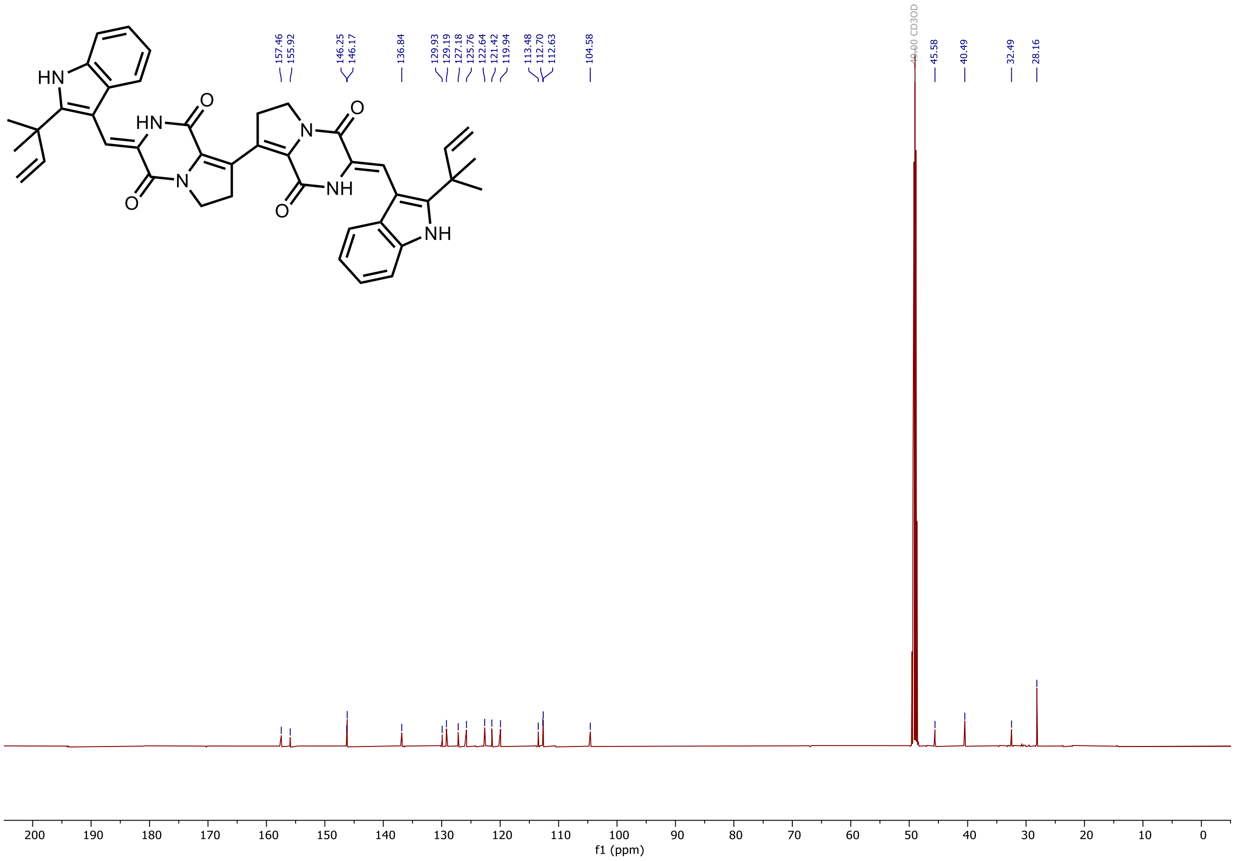

2.89. ^{1}H-^{1}H COSY Spectrum for Brevianamide S (1) (CD_3OD)

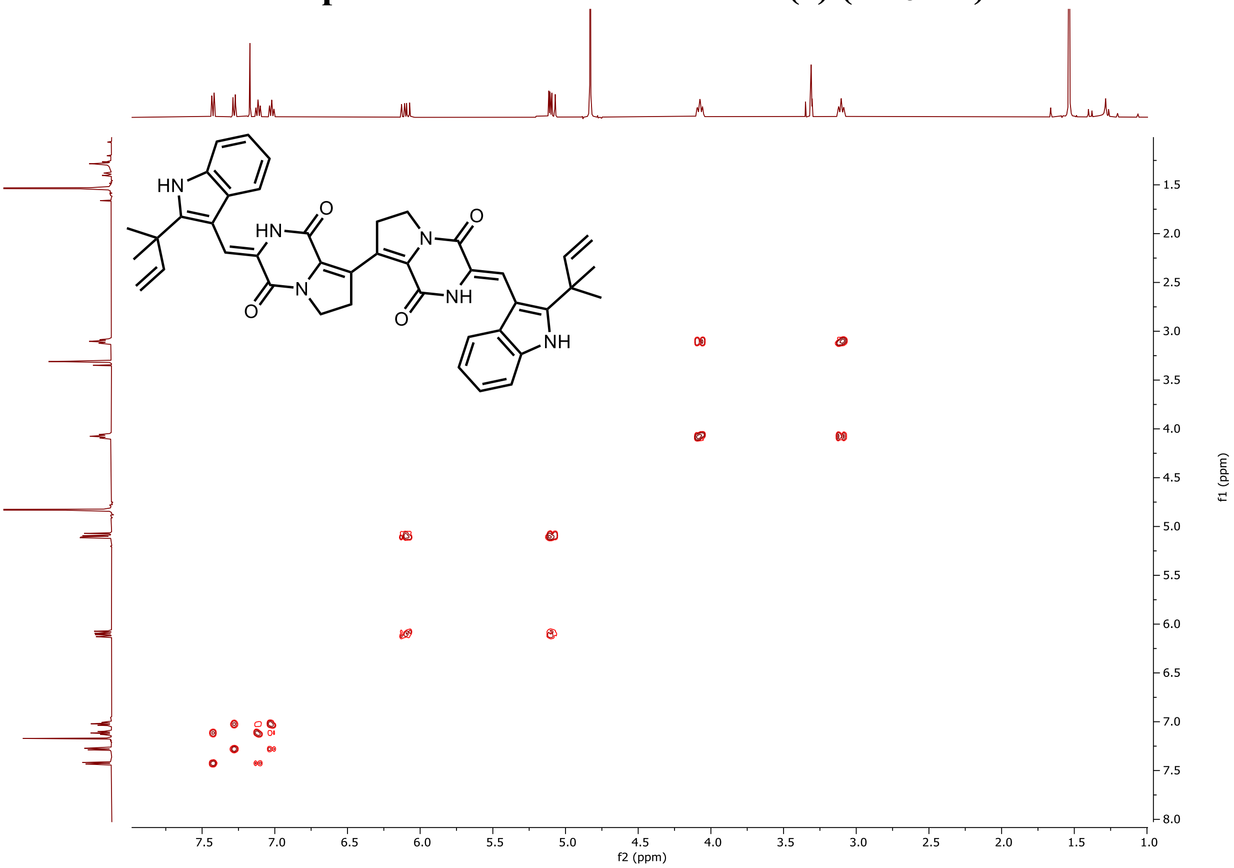

2.90. ^{1}H-^{13}C HSQC Spectrum for Brevianamide S (1) (CD_3OD)

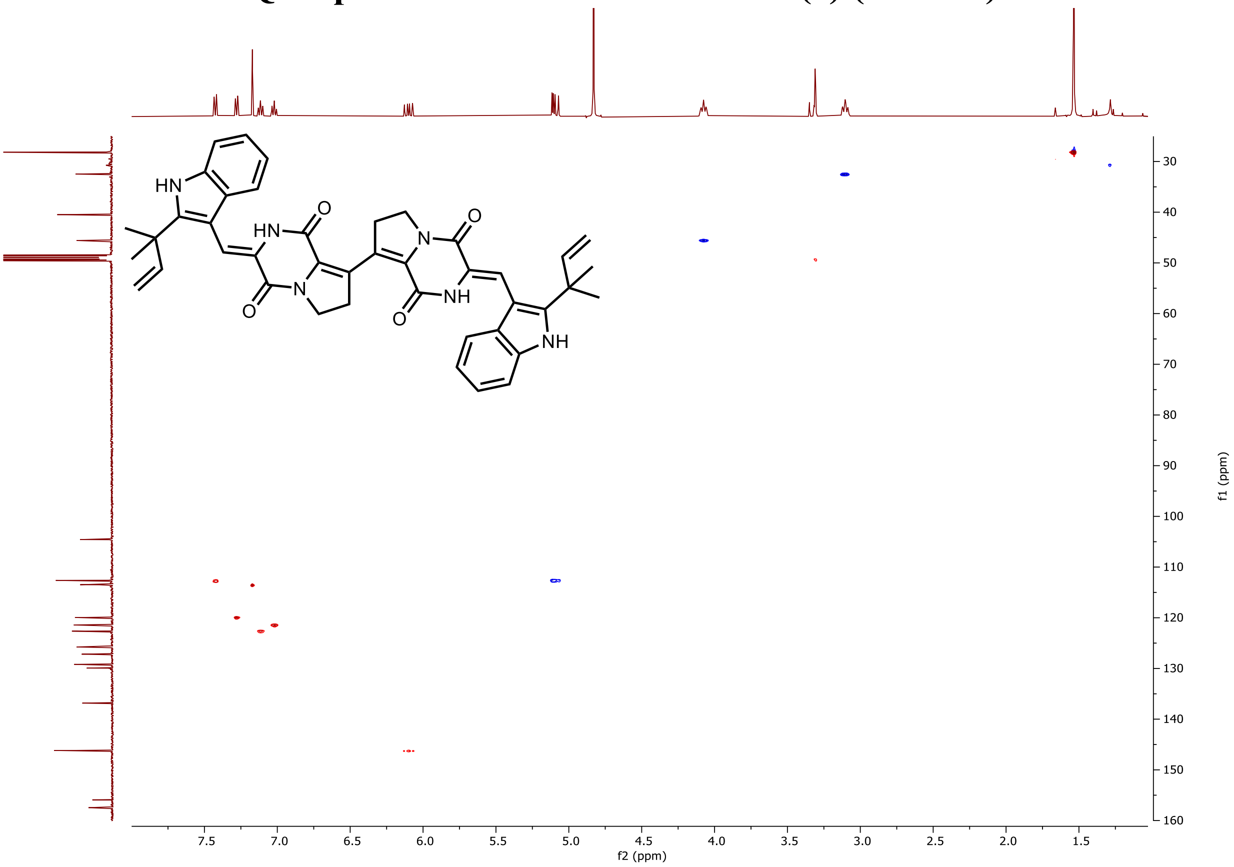

2.91. ^{1}H-^{13}C HMBC Spectrum for Brevianamide S (1) (CD$_3$OD)

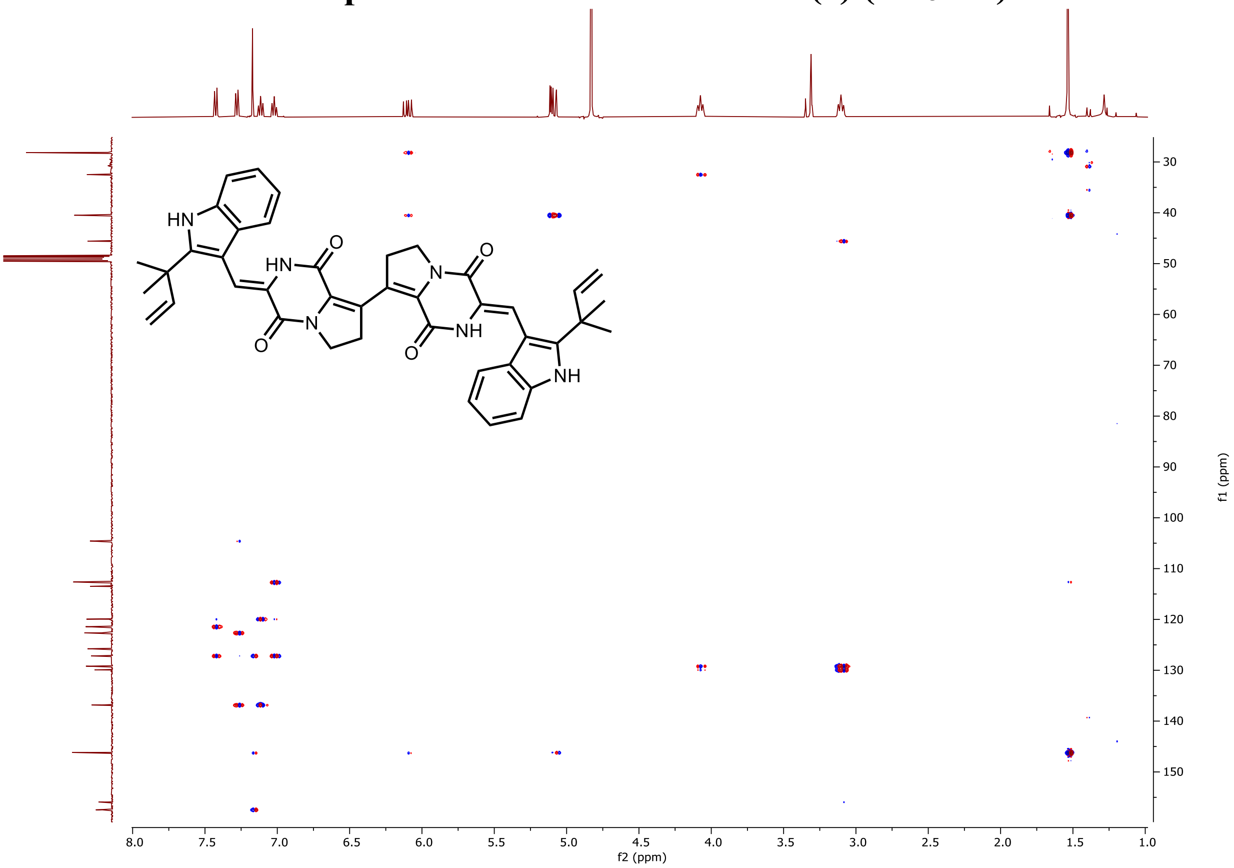

2.92. ^{1}H NMR Spectrum for Dehydroproline 7 (500 MHz, CDCl$_3$)

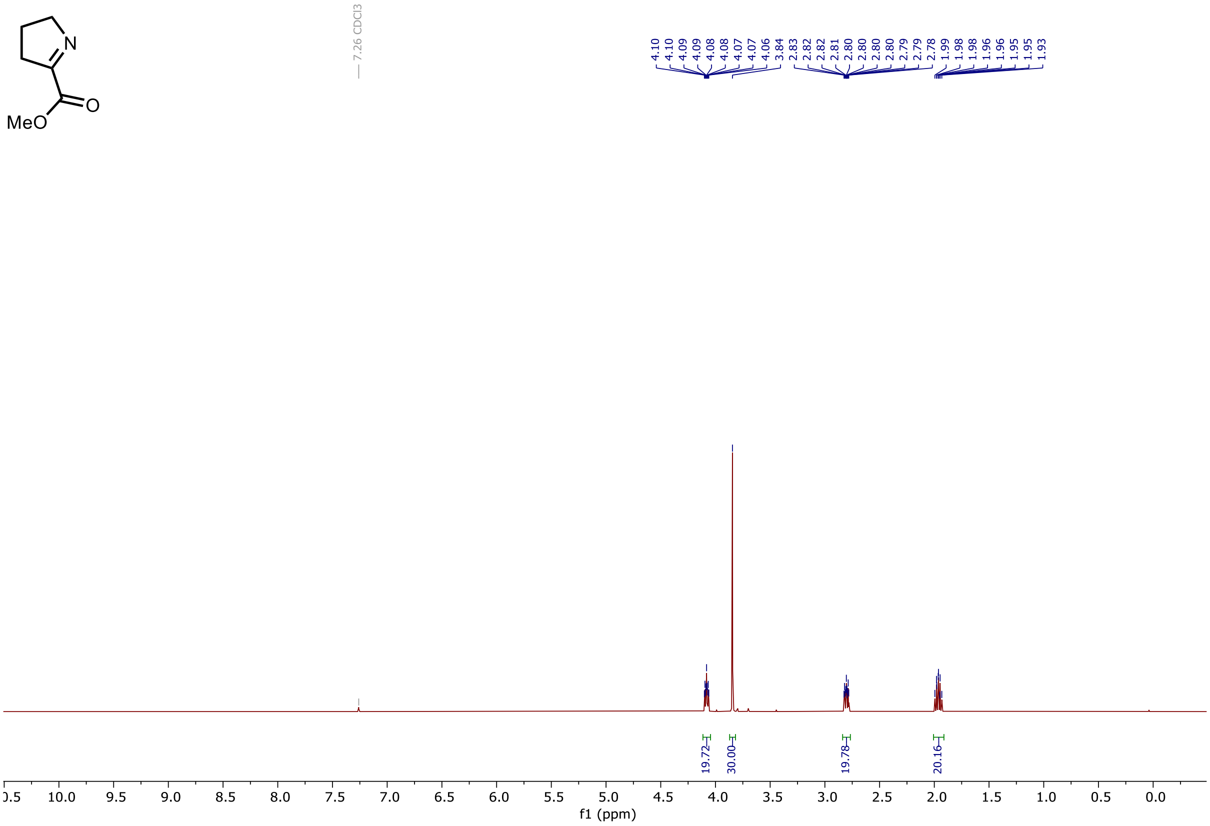

2.93. ^{13}C NMR Spectrum for Dehydroproline 7 (126 MHz, $CDCl_3$)

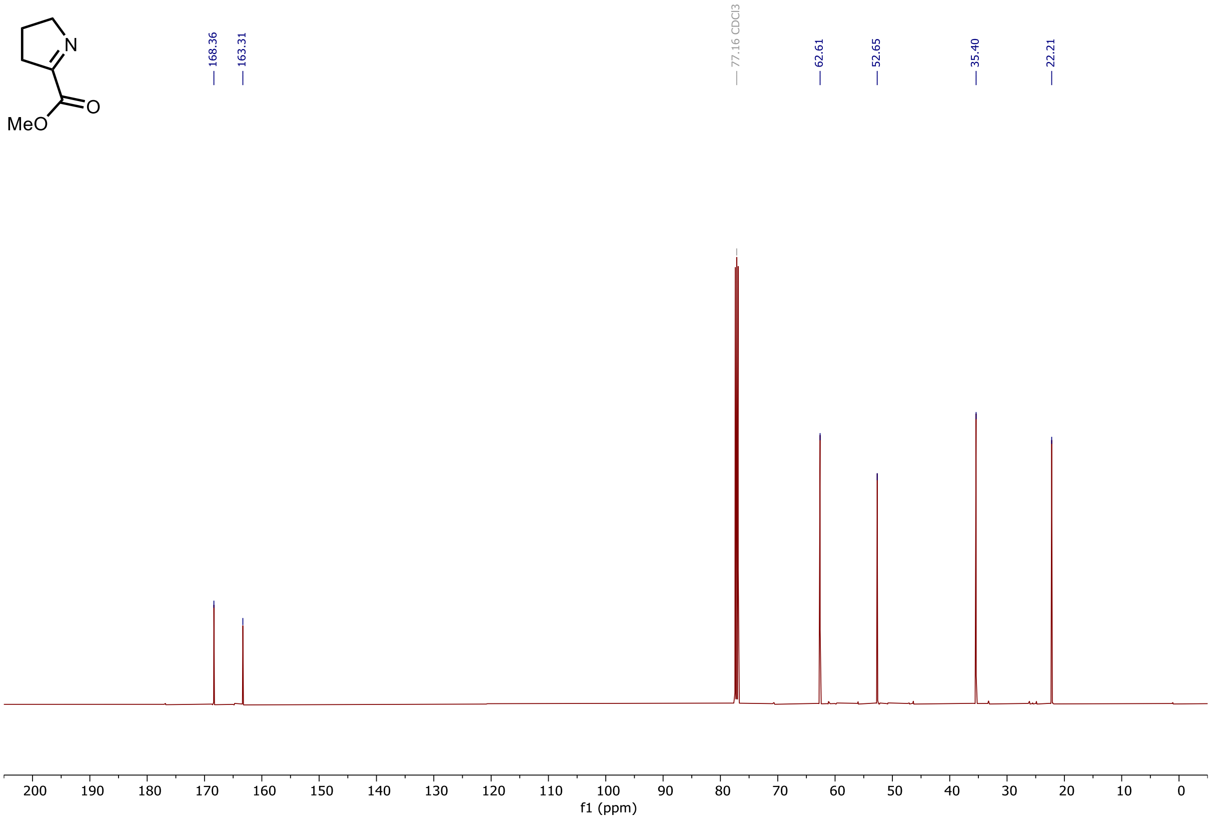

2.94. ^{13}C DEPT-135 Spectrum for Dehydroproline 7 ($CDCl_3$)

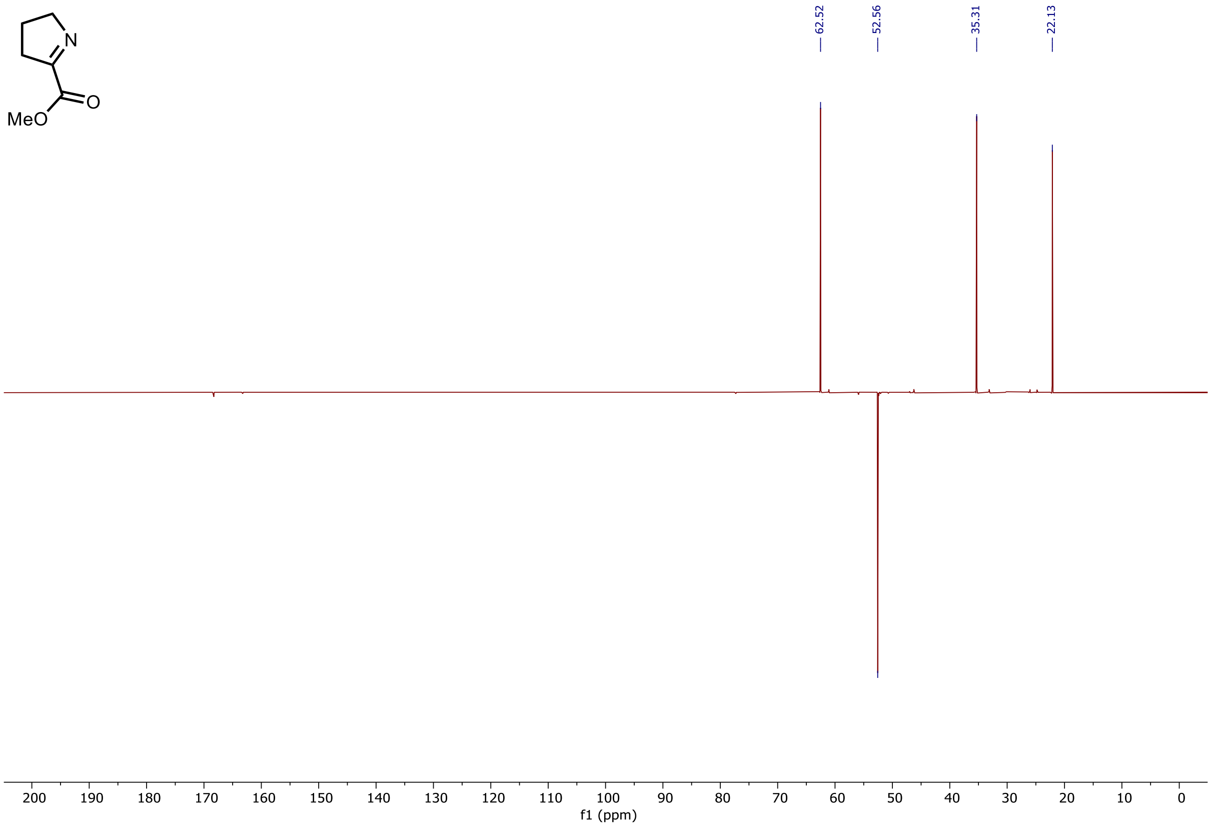

2.95. 1H-1H COSY Spectrum for Dehydroproline 7 (CDCl3)

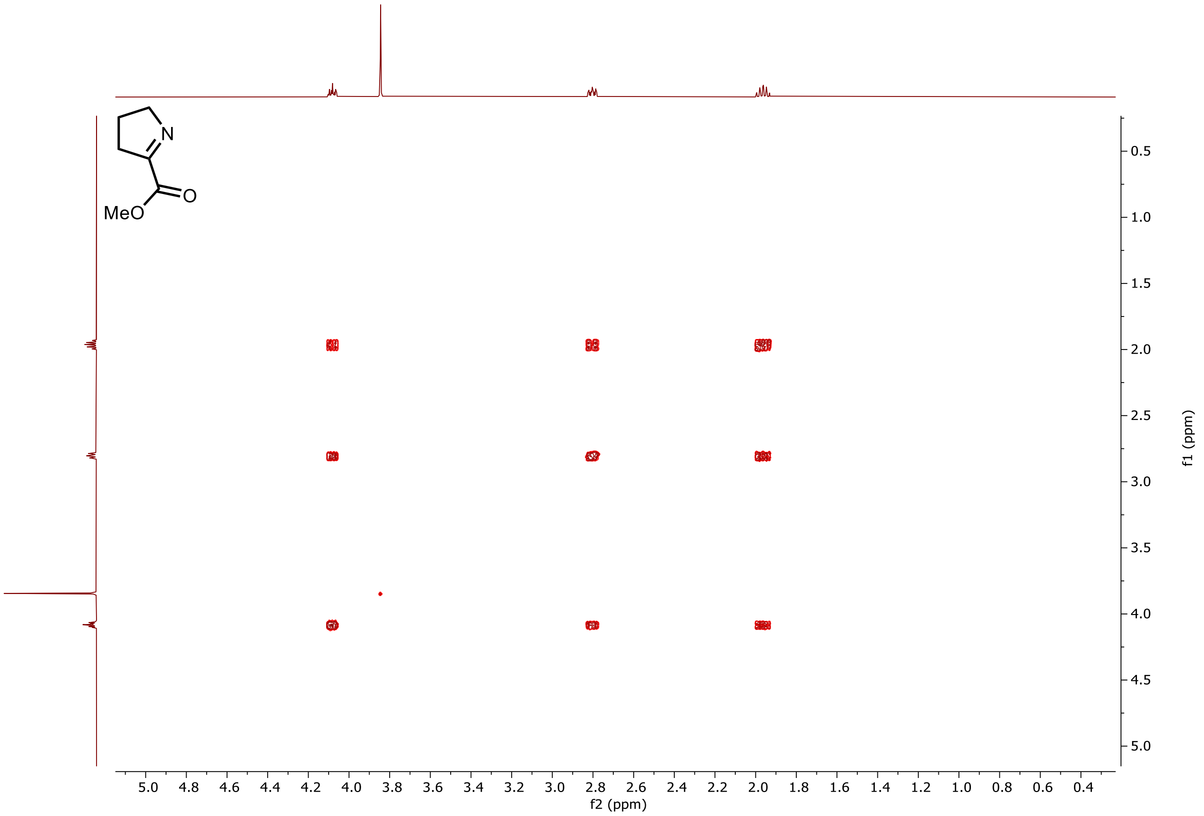

2.96. ^{1}H-^{13}C HSQC Spectrum for Dehydroproline 7 ($CDCl_3$)

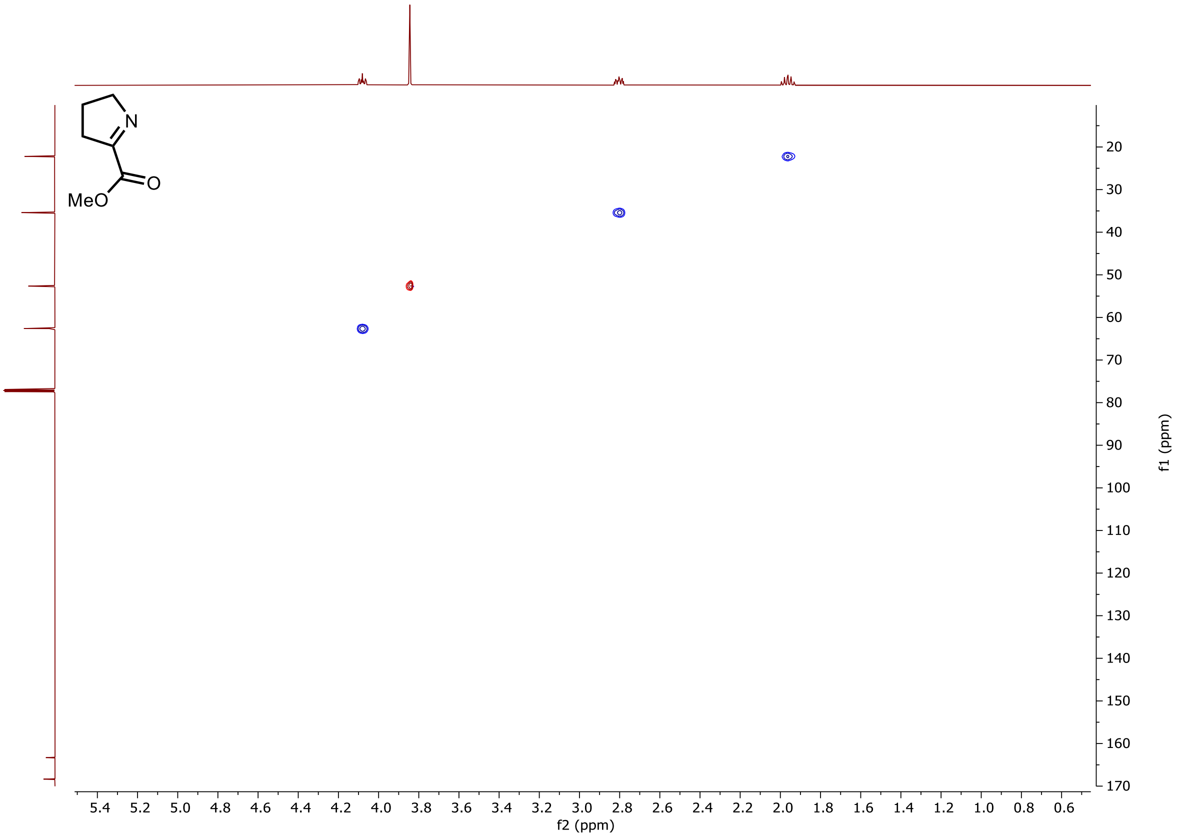

2.97. ^{1}H-^{13}C HMBC Spectrum for Dehydroproline 7 ($CDCl_3$)

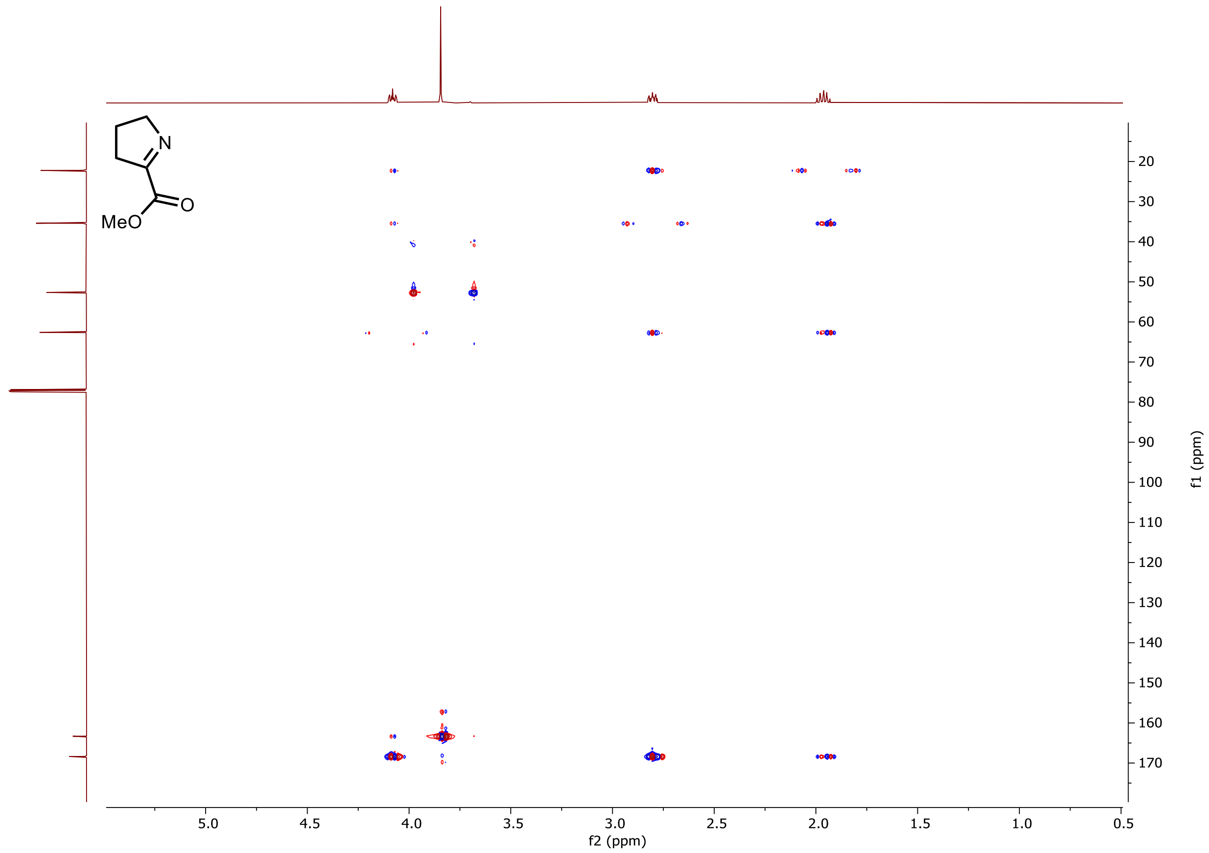

2.98. ^{1}H NMR Spectrum for β-Lactam (±)-9 (500 MHz, $CDCl_3$)

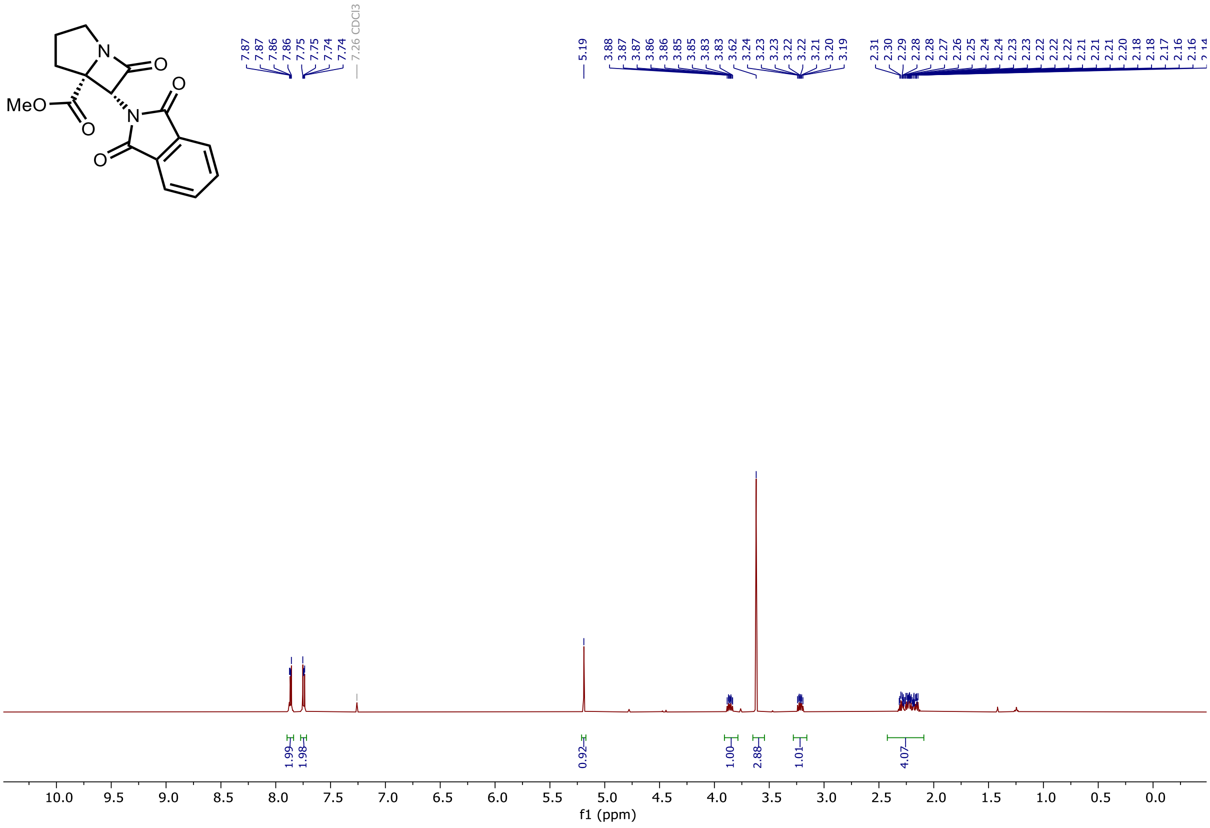

2.99. ^{13}C NMR Spectrum for β-Lactam (±)-9 (126 MHz, CDCl$_3$)

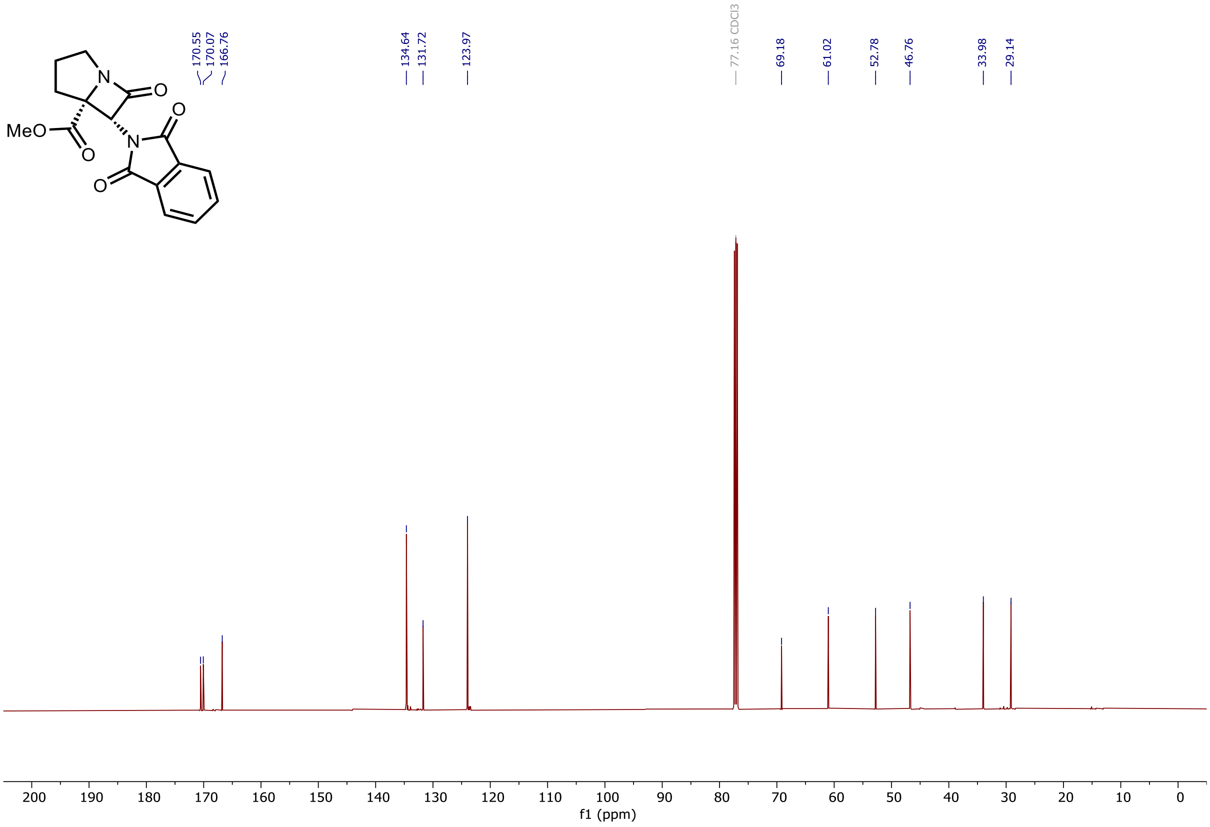

2.100. ^{13}C DEPT-135 Spectrum for β-Lactam (±)-9 (CDCl$_3$)

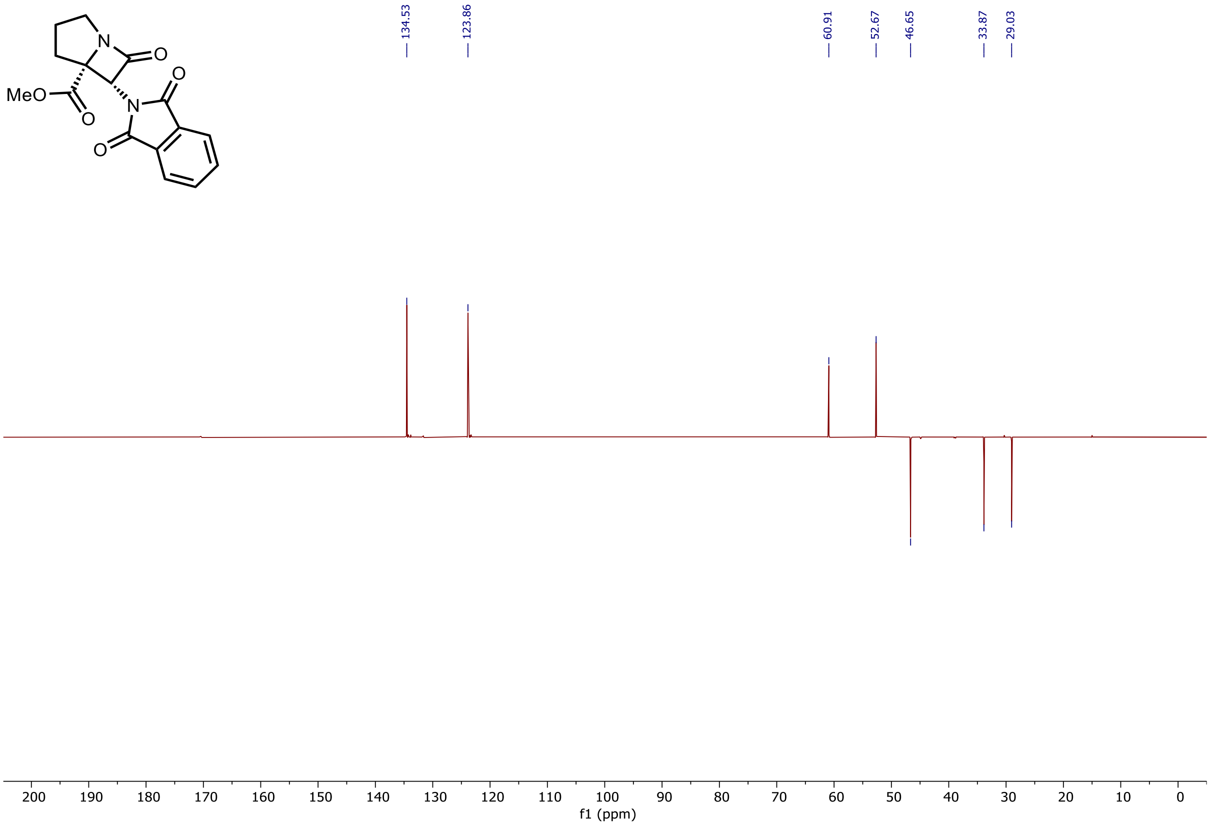

2.101. ^{1}H-^{1}H COSY Spectrum for β-Lactam (±)-9 ($CDCl_3$)

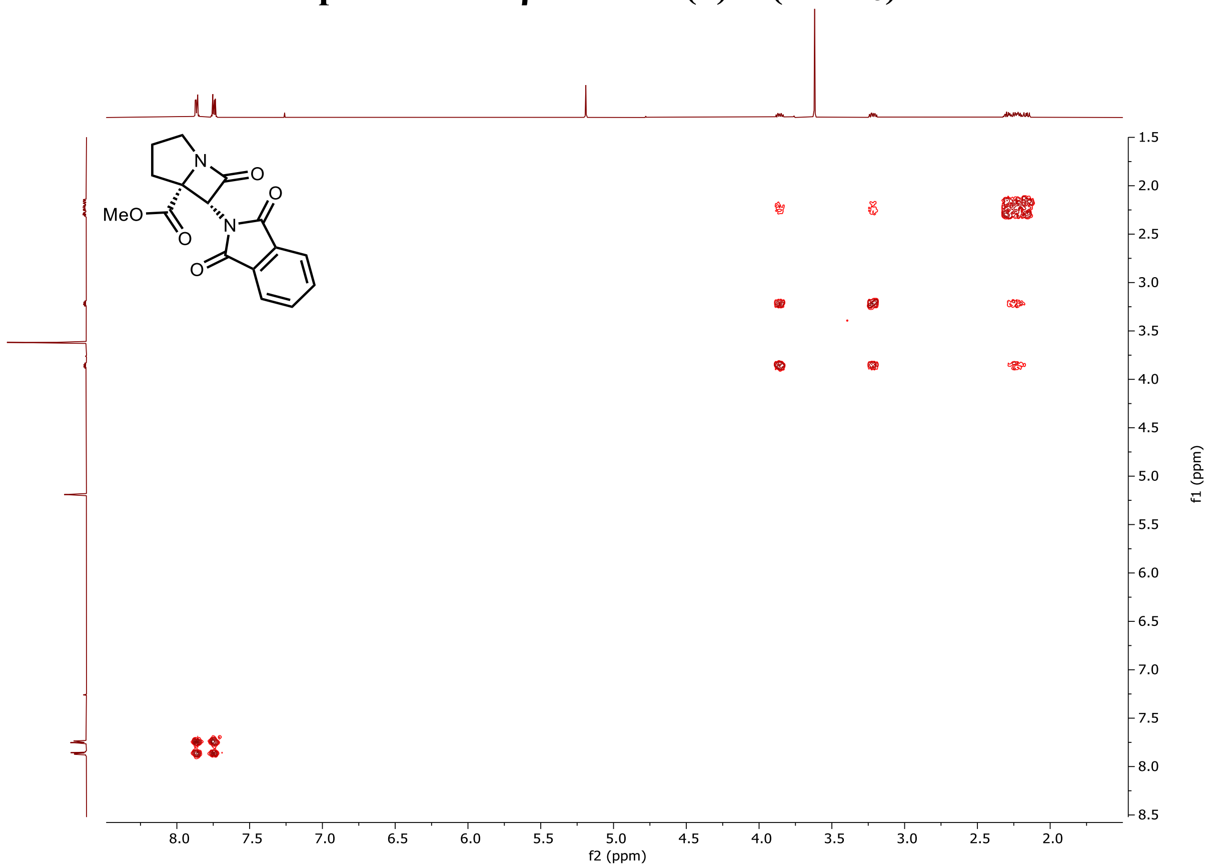

2.102. ^{1}H-^{13}C HSQC Spectrum for β-Lactam (±)-9 ($CDCl_3$)

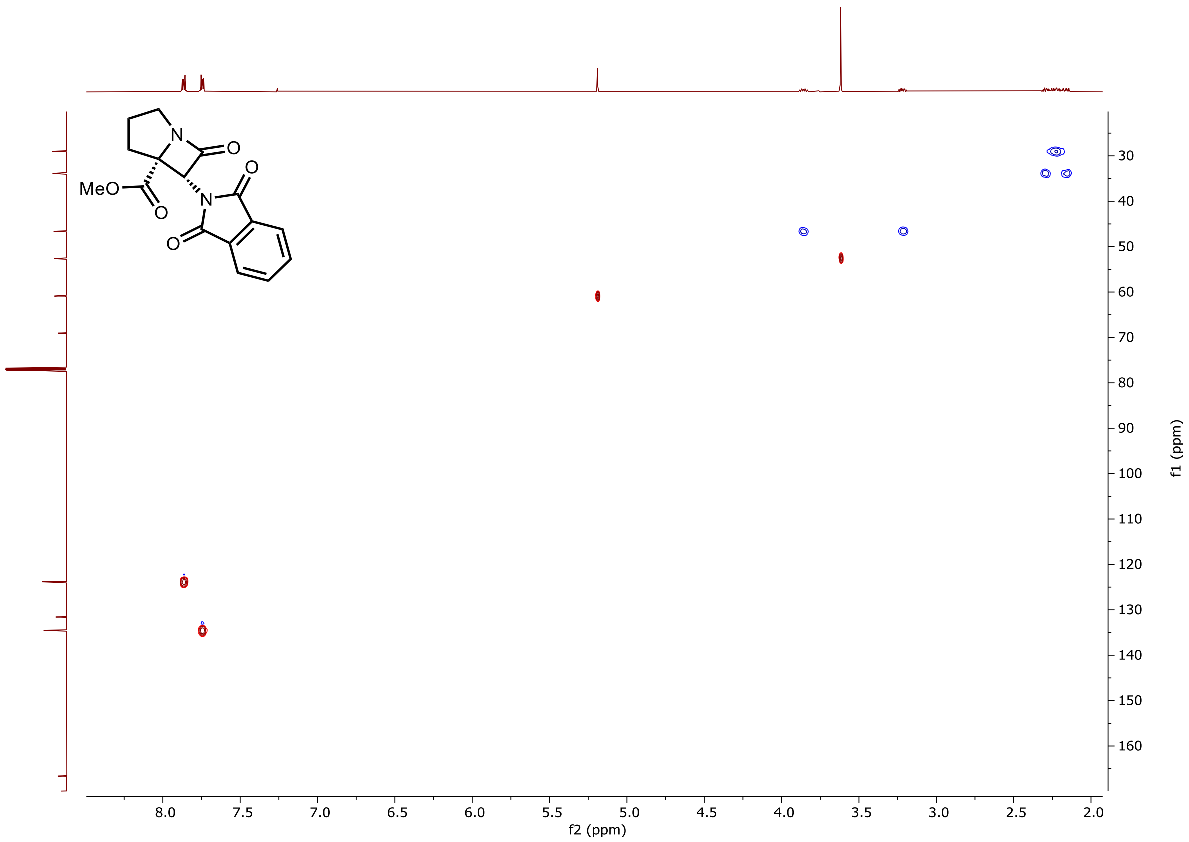

2.103. ^{1}H-^{13}C HMBC Spectrum for β-Lactam (±)-9 ($CDCl_3$)

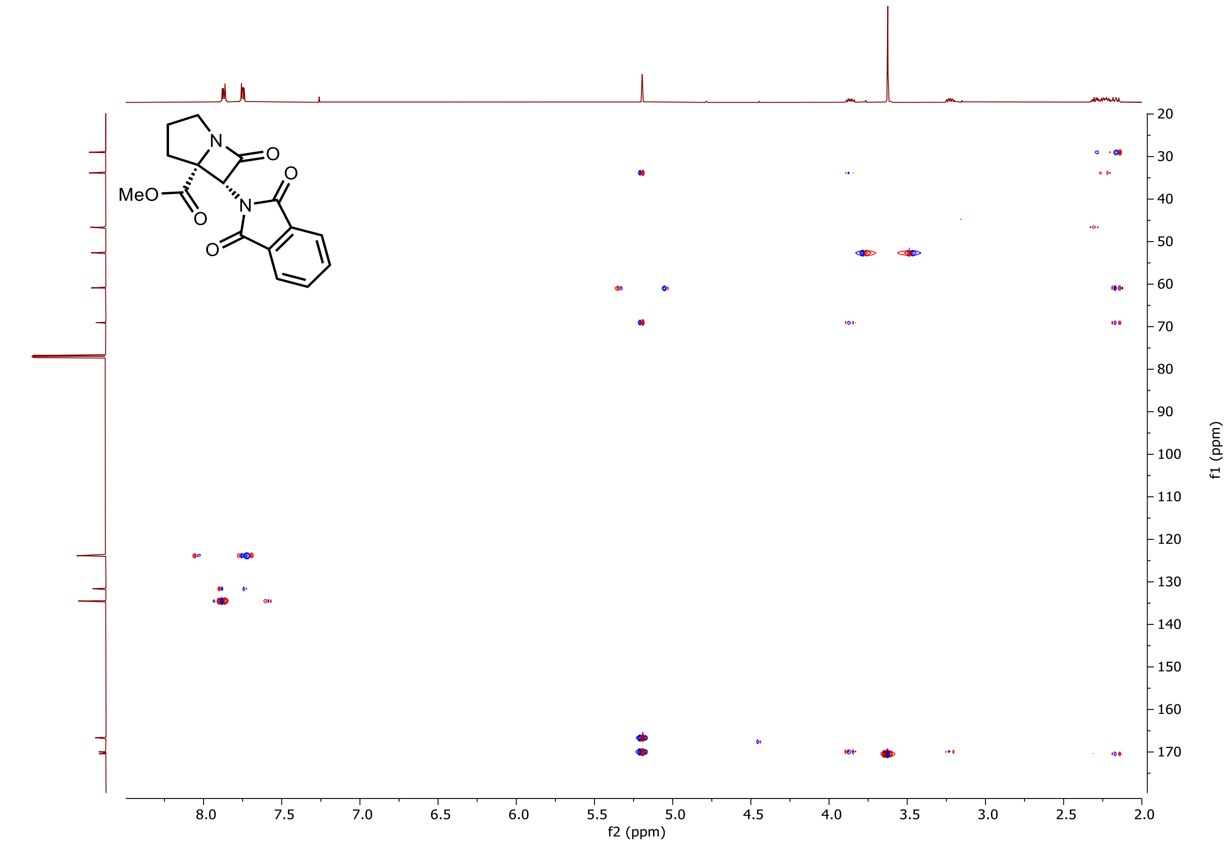

2.104. ^{1}H-^{1}H NOESY Spectrum for β-Lactam (±)-9 ($CDCl_3$)

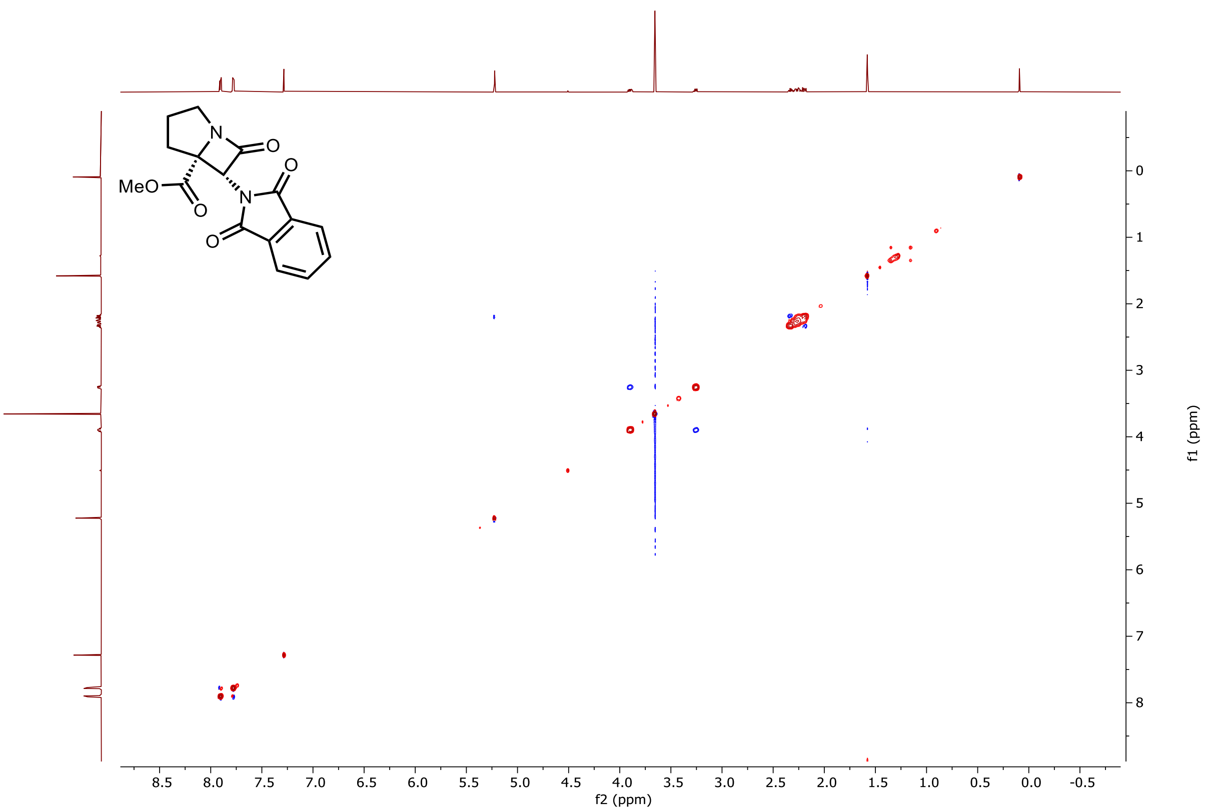

2.105. ^{1}H NMR Spectrum for Iodide S-2 (500 MHz, $CDCl_3$)

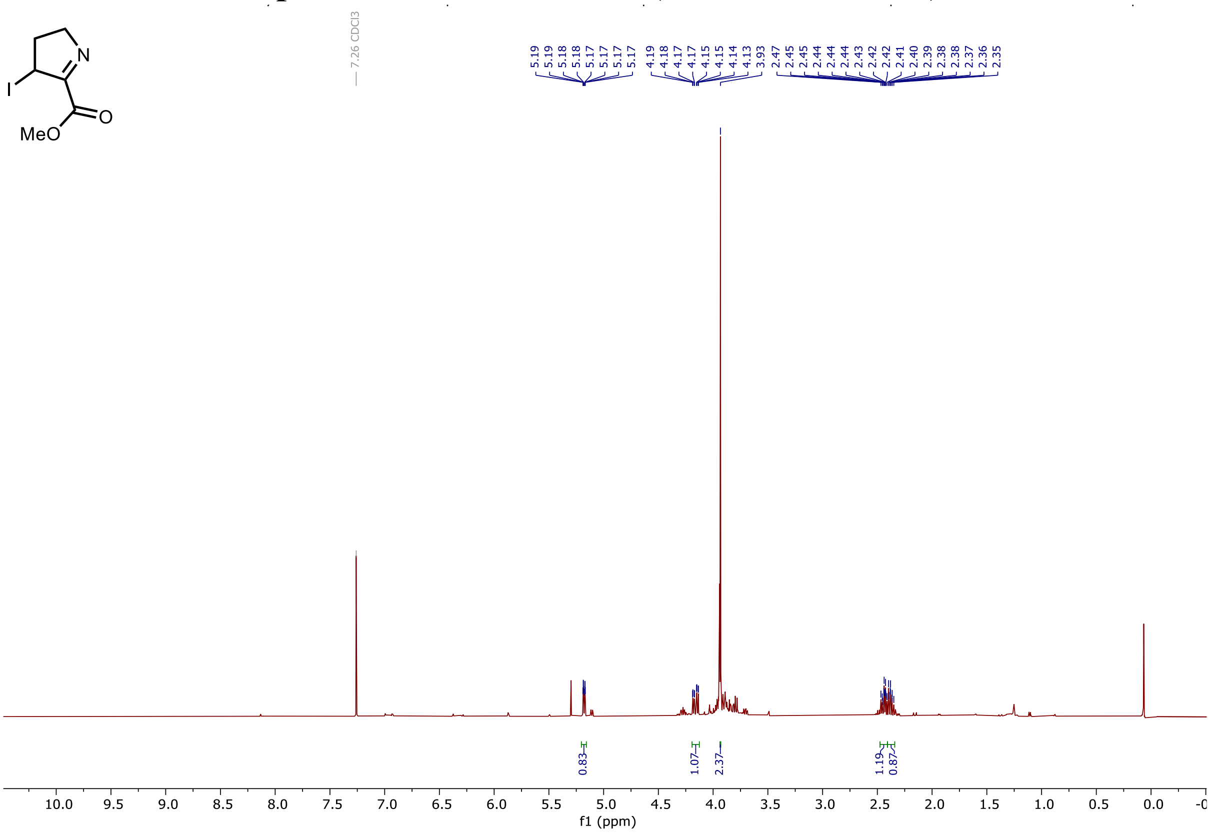

2.106. ^{13}C NMR Spectrum for Iodide S-2 (126 MHz, $CDCl_3$)

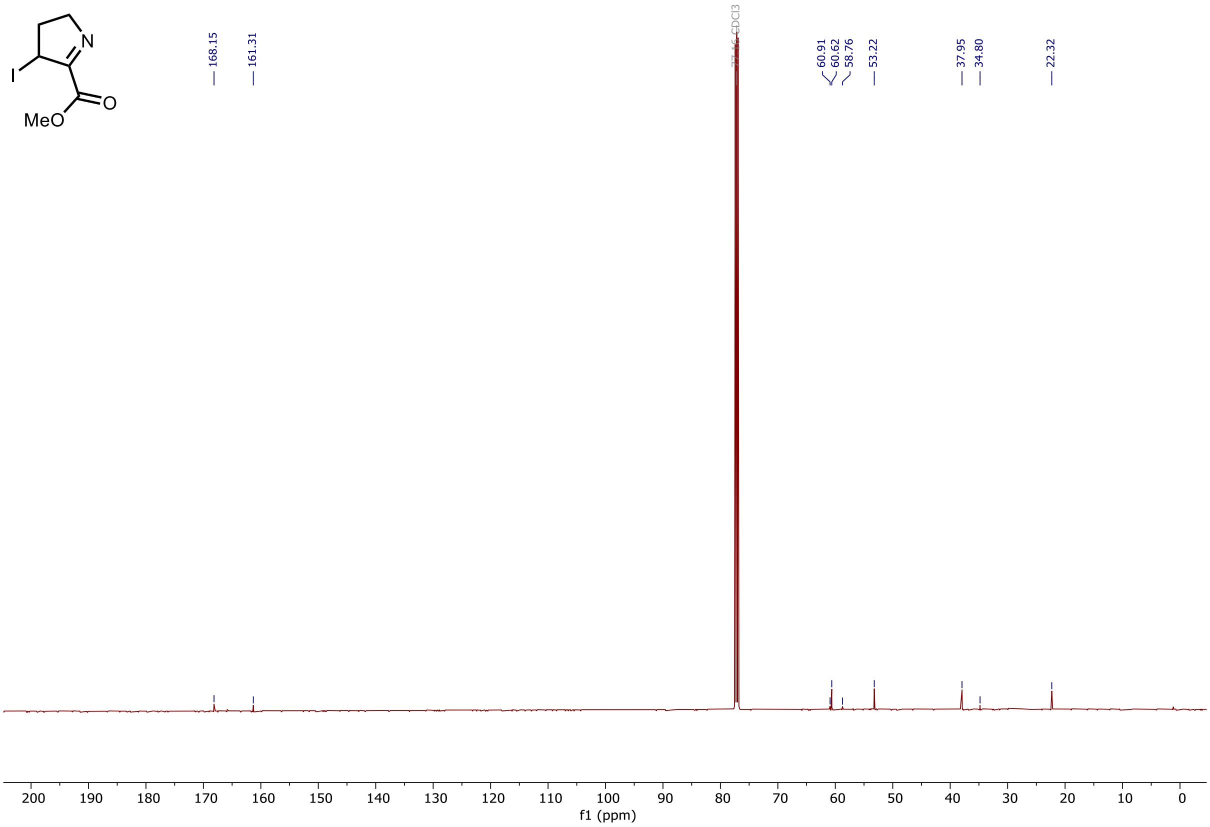

2.107. ^{13}C DEPT-135 Spectrum for Iodide S-2 ($CDCl_3$)

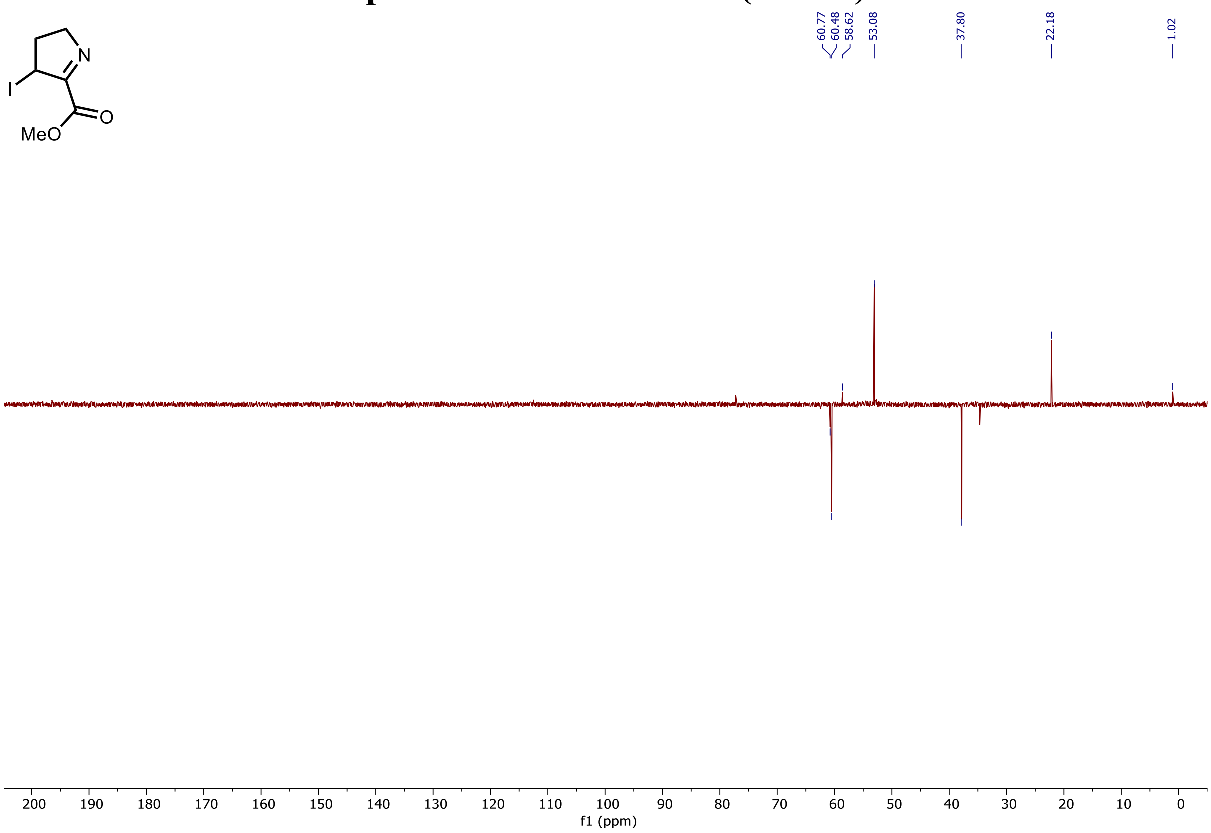

2.108. ^{1}H-^{1}H COSY Spectrum for Iodide S-2 ($CDCl_3$)

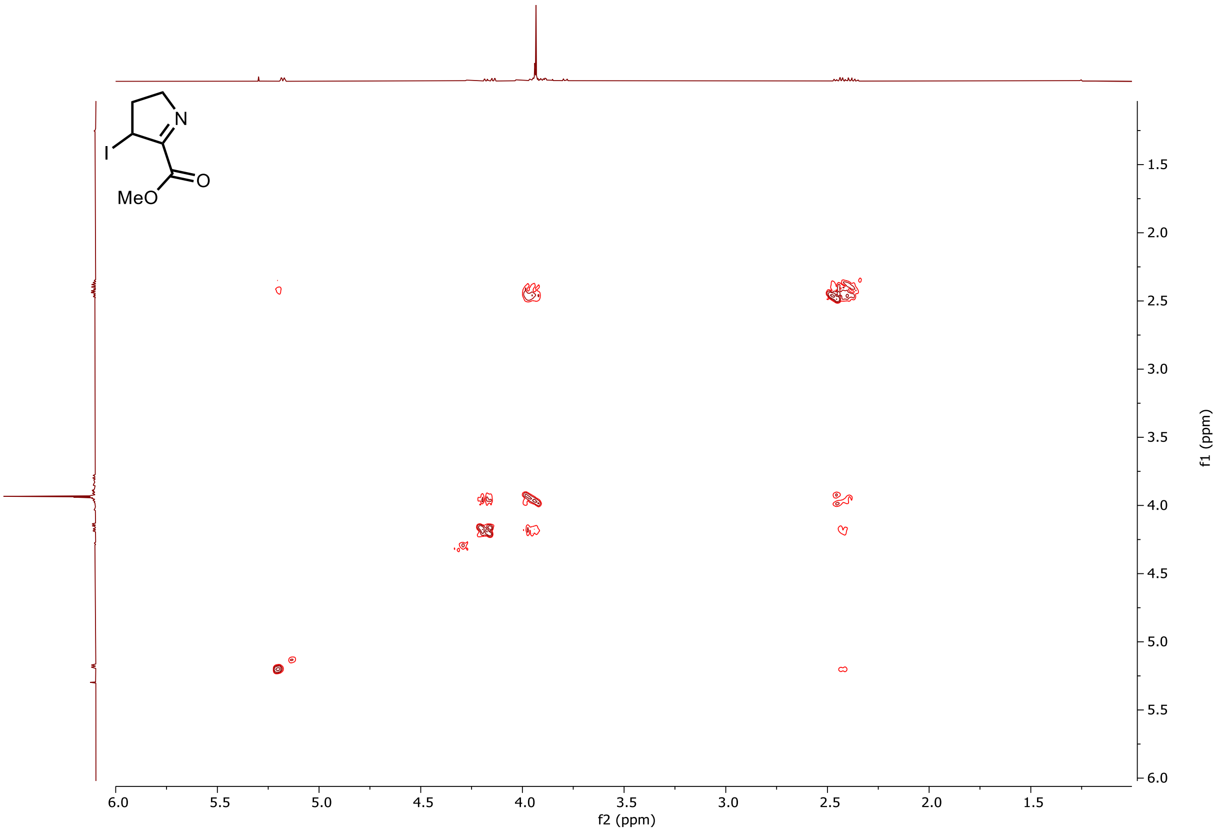

2.109. ^{1}H-^{13}C HSQC Spectrum for Iodide S-2 ($CDCl_3$)

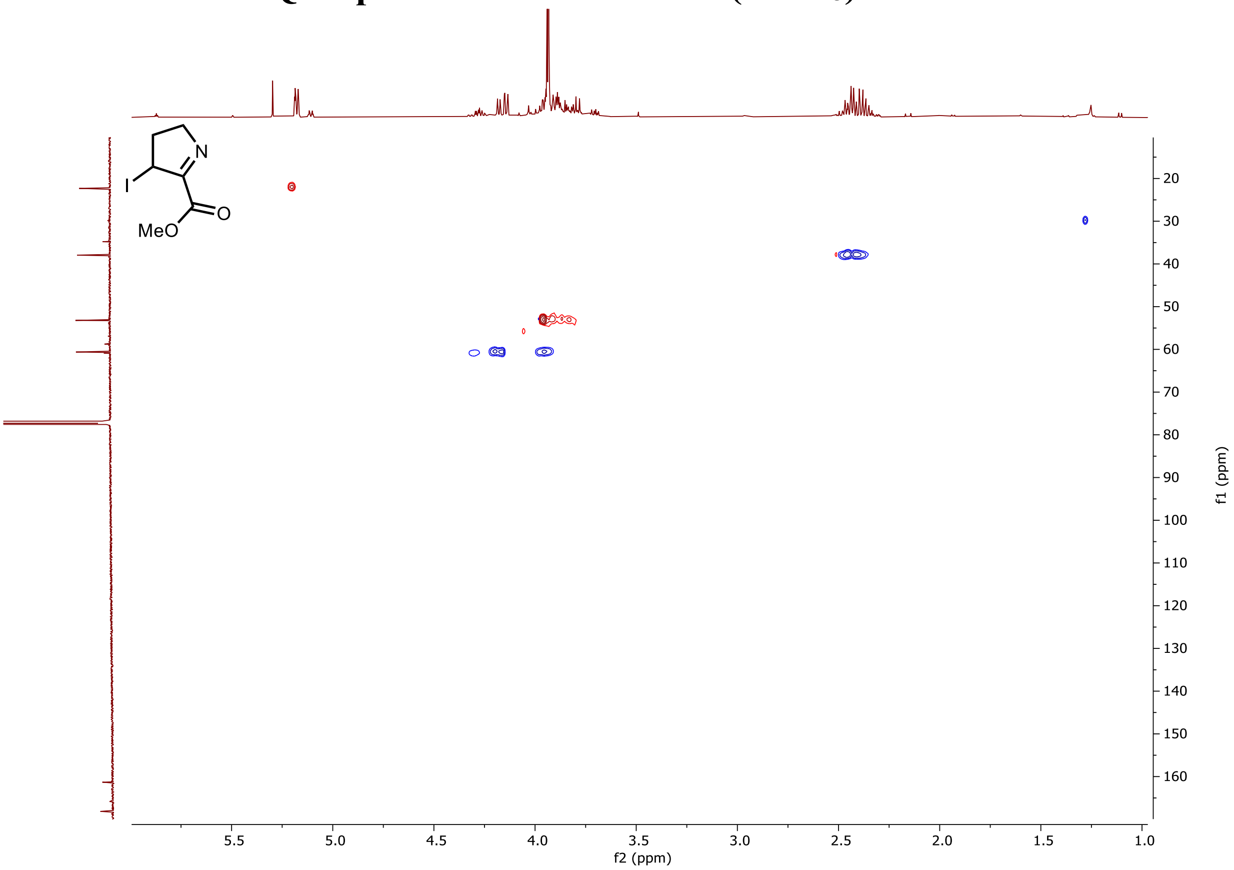

2.110. ^{1}H-^{13}C HMBC Spectrum for Iodide S-2 ($CDCl_3$)

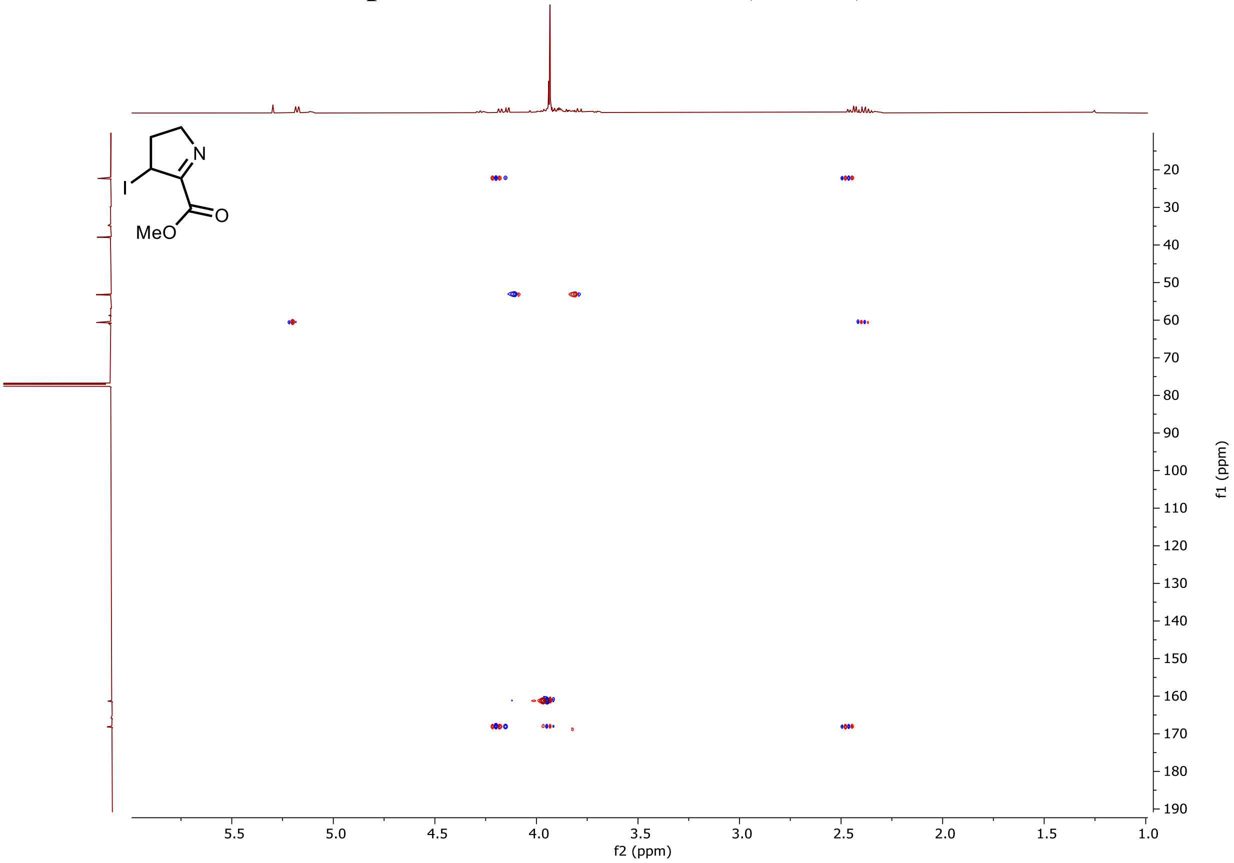

2.111. ^{1}H NMR Spectrum for β-Lactam (±)-S-3 (500 MHz, $CDCl_3$)

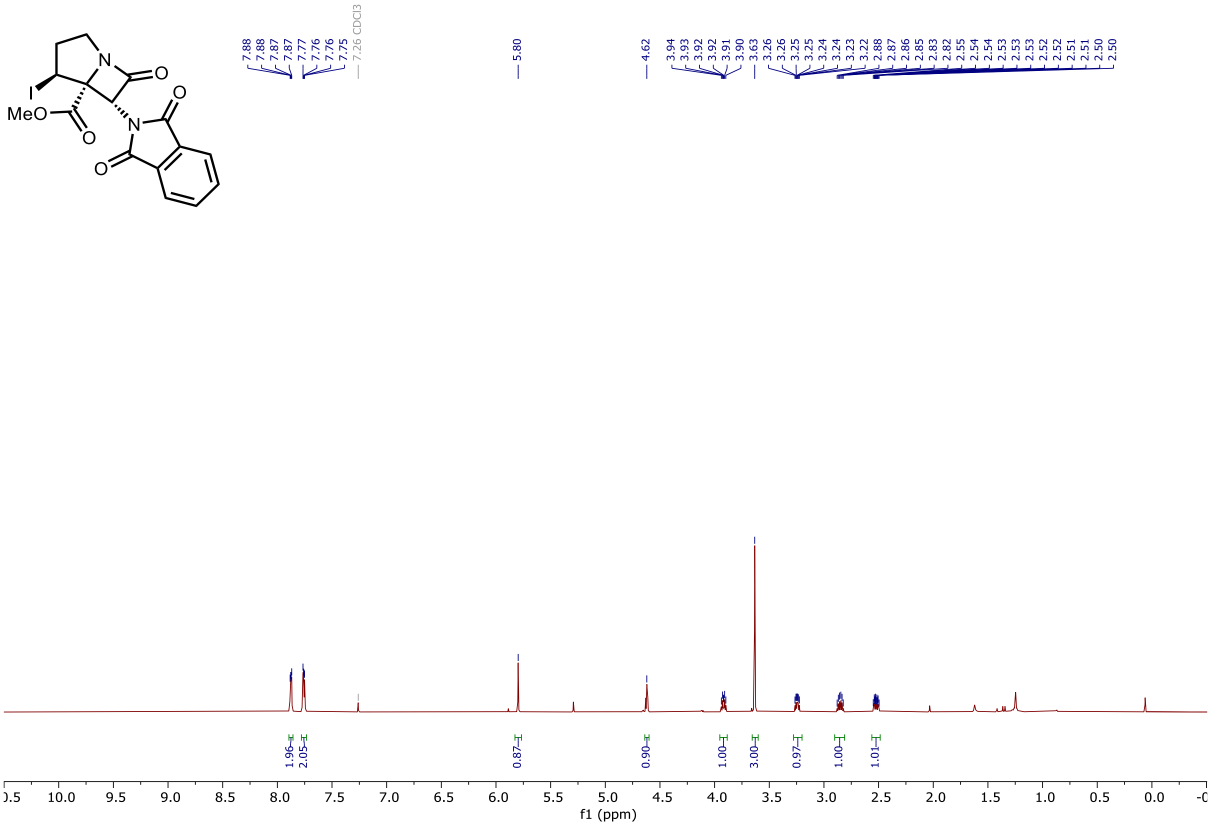

2.112. ^{13}C NMR Spectrum for β-Lactam (±)-S-3 (126 MHz, $CDCl_3$)

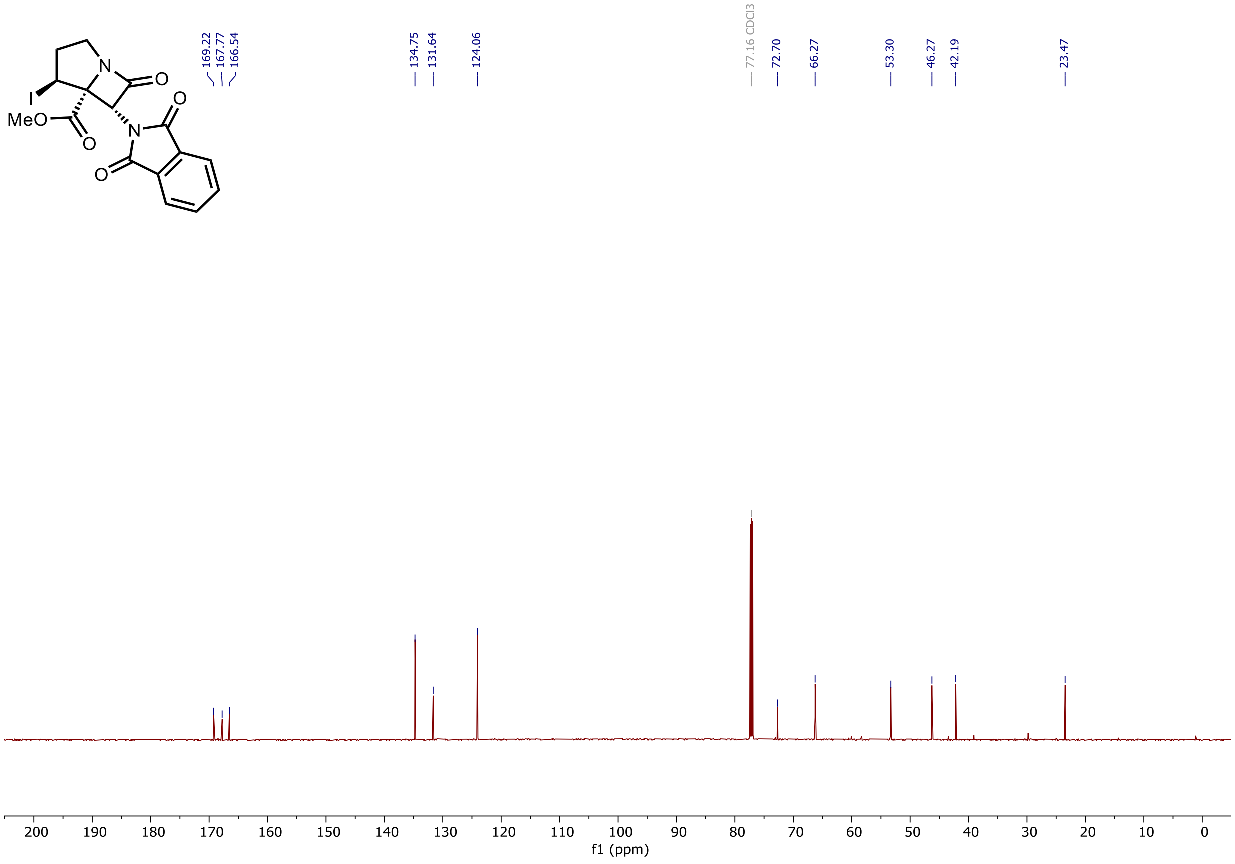

2.113. ^{13}C DEPT-135 Spectrum for β-Lactam (±)-S-3 (CDCl$_3$)

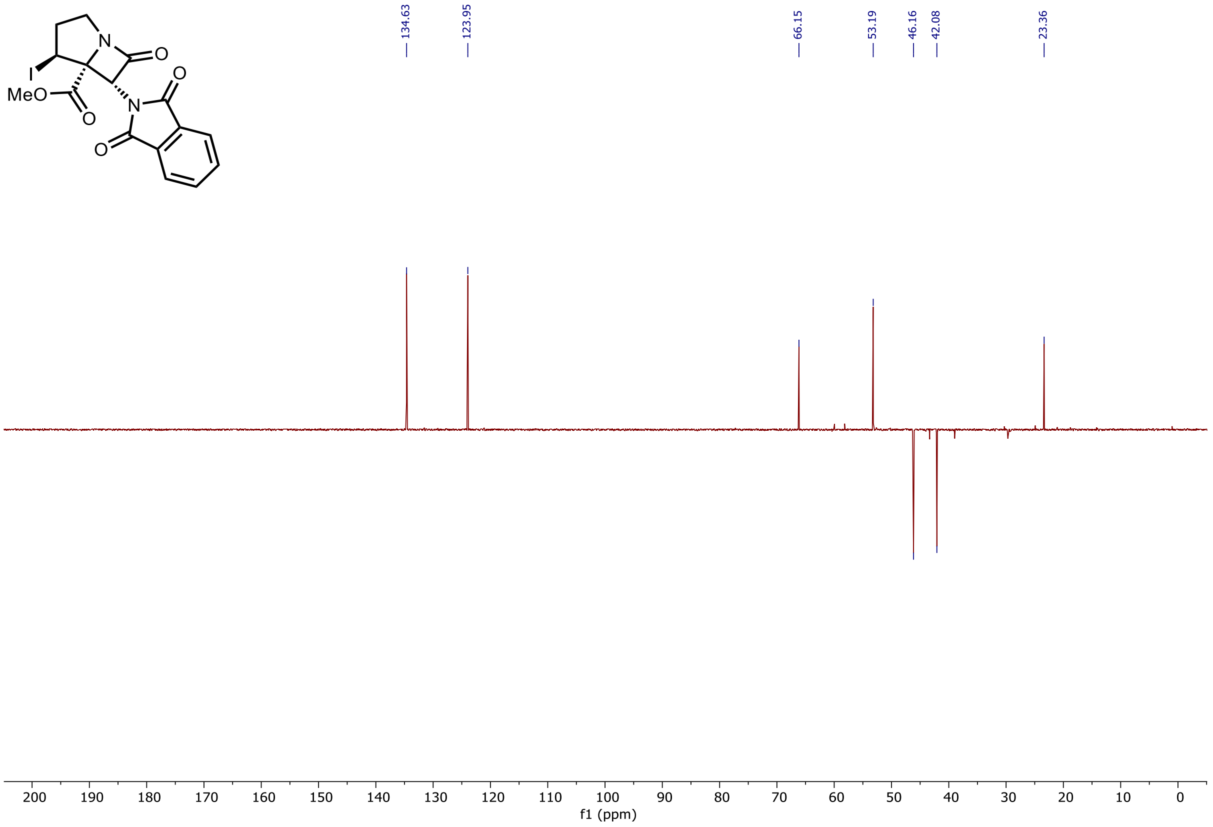

2.114. ^{1}H-^{1}H COSY Spectrum for β-Lactam (±)-S-3 (CDCl$_3$)

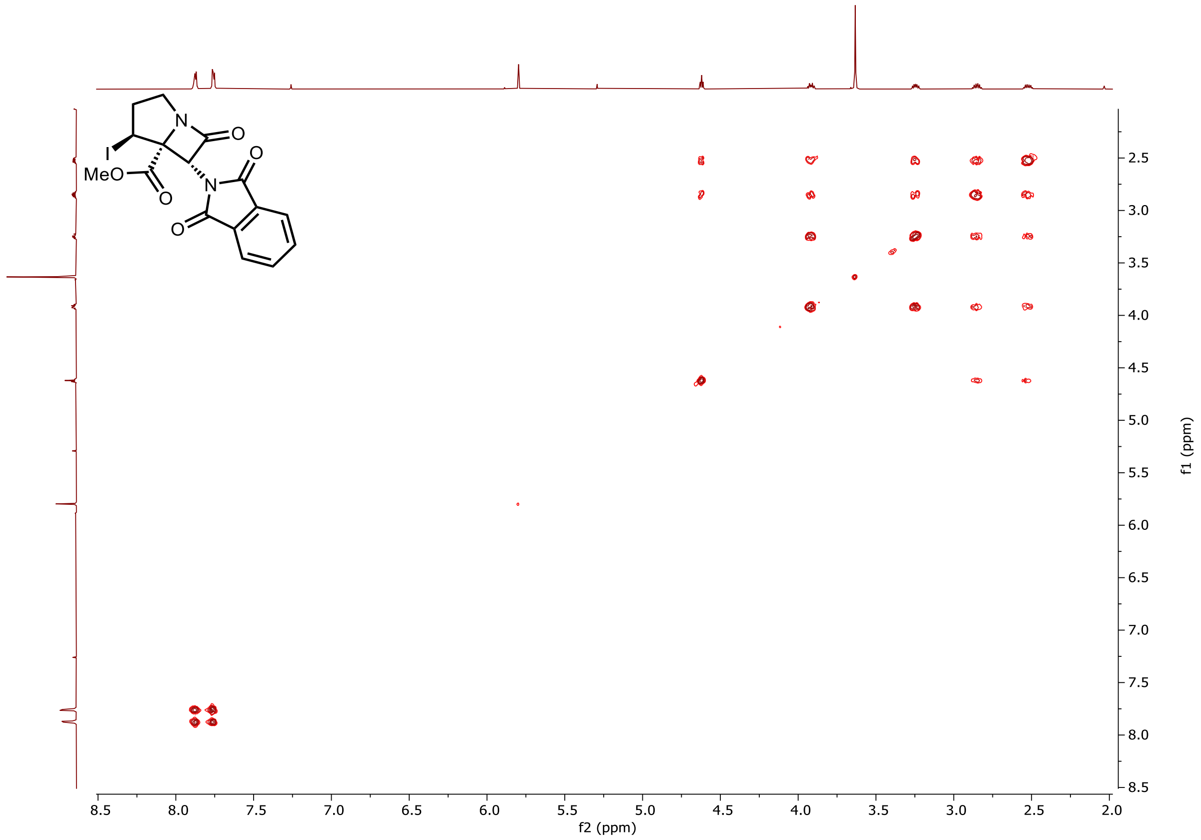

2.115. ^{1}H-^{13}C HSQC Spectrum for β-Lactam (±)-S-3 (CDCl$_3$)

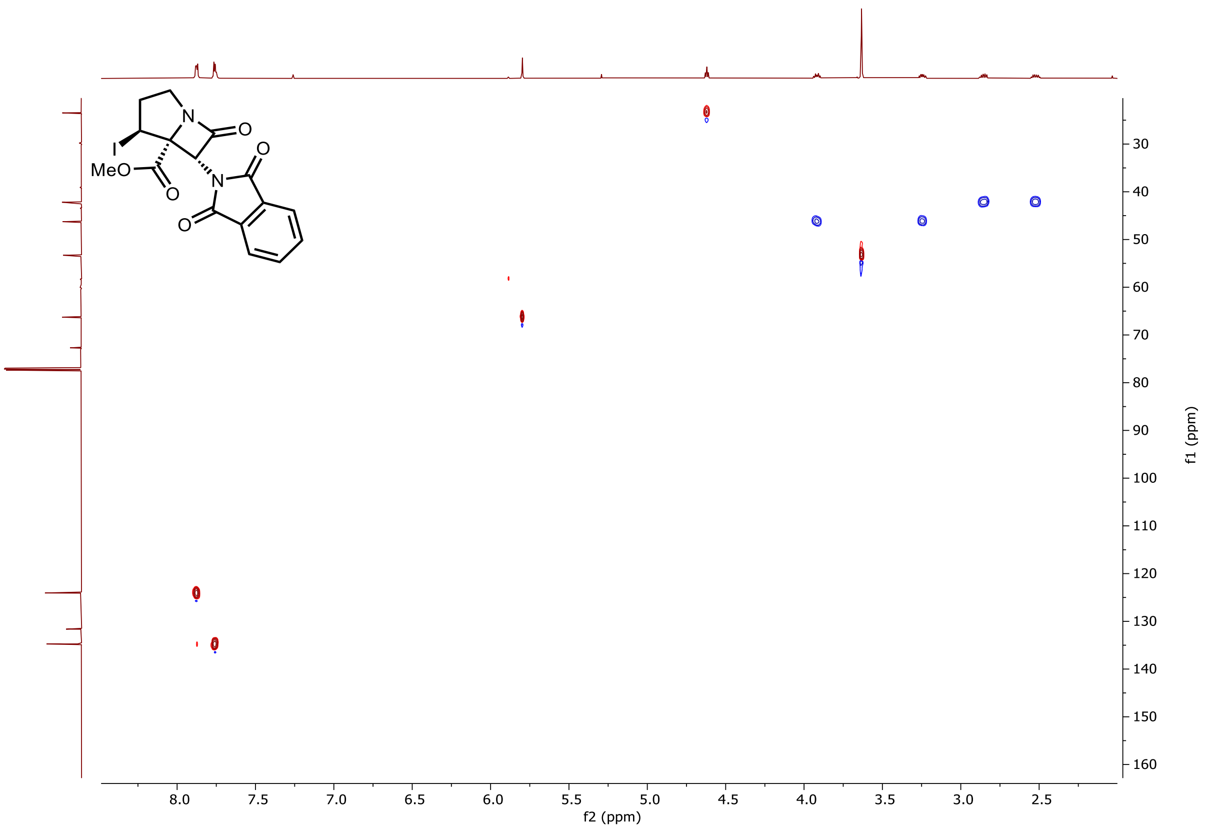

2.116. ^{1}H-^{13}C HMBC Spectrum for β-Lactam (±)-S-3 (CDCl$_3$)

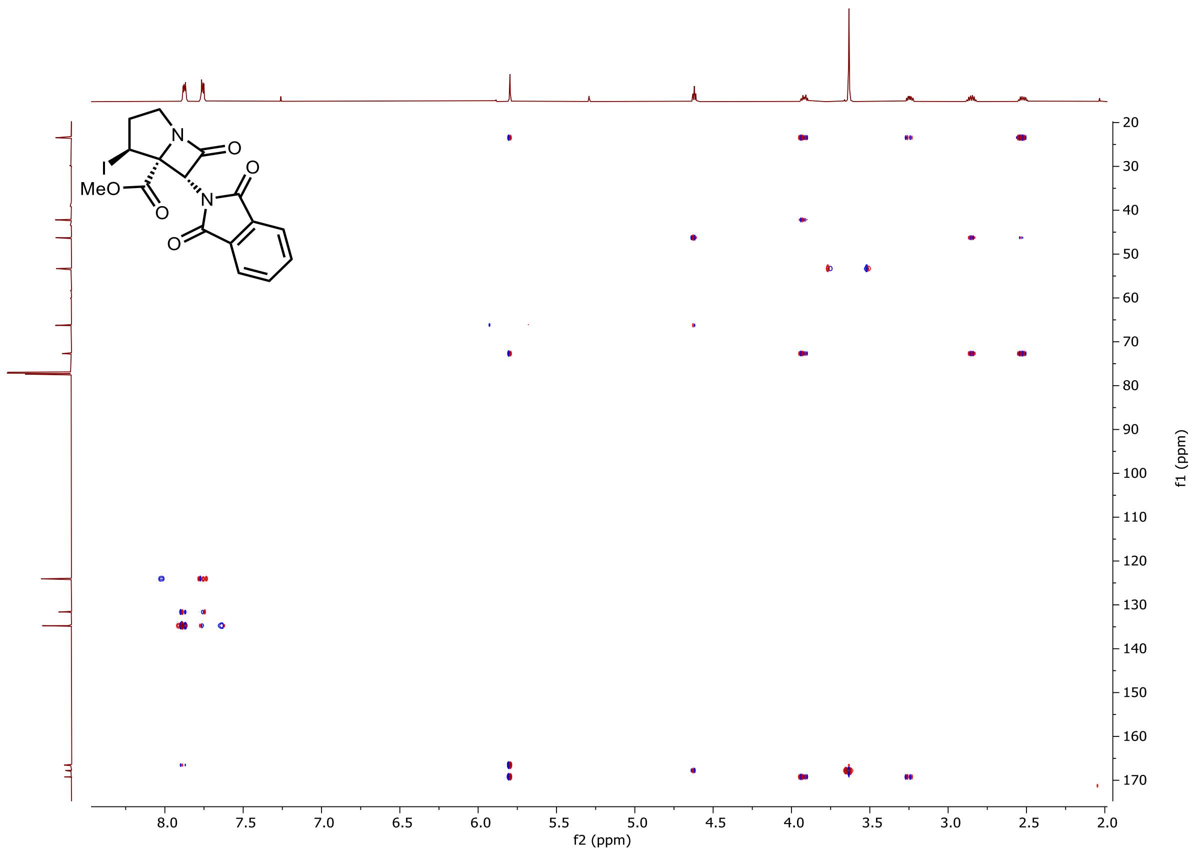

2.117. ^{1}H-^{1}H NOESY Spectrum for β-Lactam (±)-S-3 (CDCl₃)

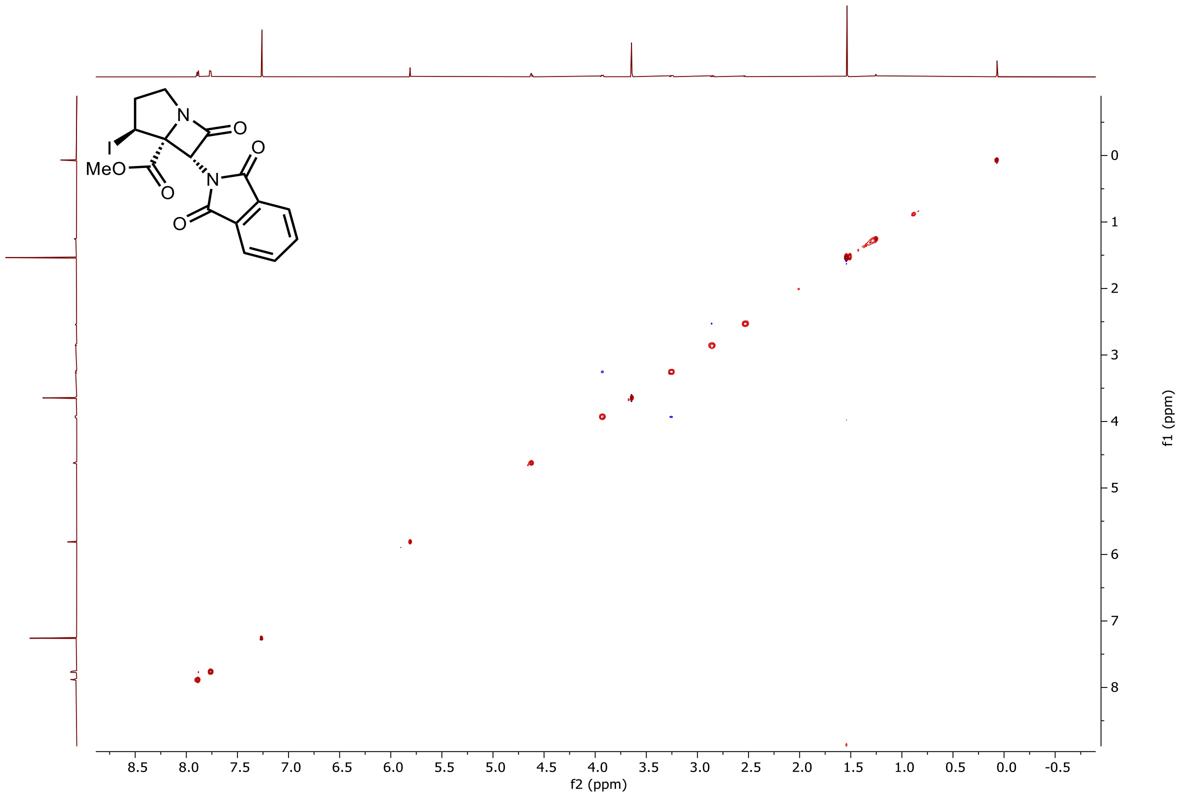

2.118. ^{1}H NMR Spectrum for Hemiaminal S-4 (500 MHz, CDCl₃)

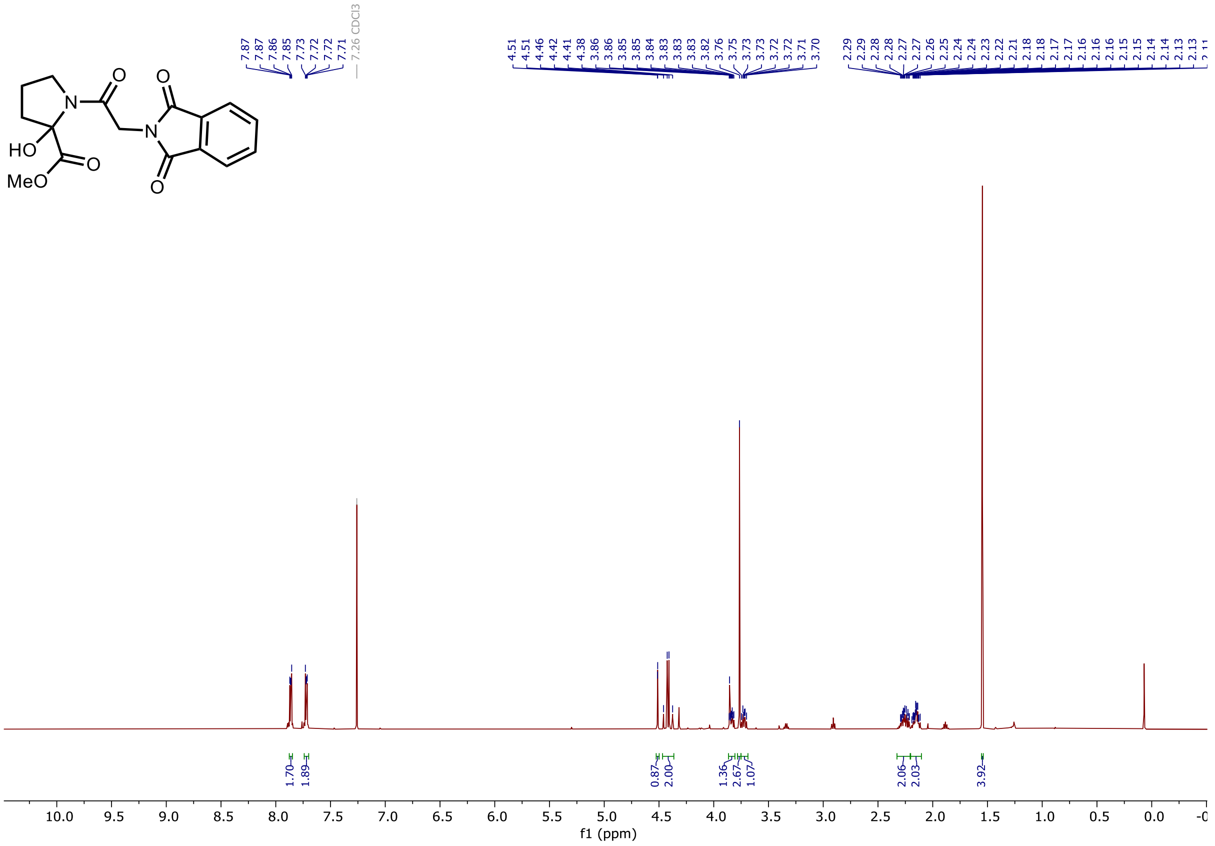

2.119. ^{13}C NMR Spectrum for Hemiaminal S-4 (126 MHz, $CDCl_3$)

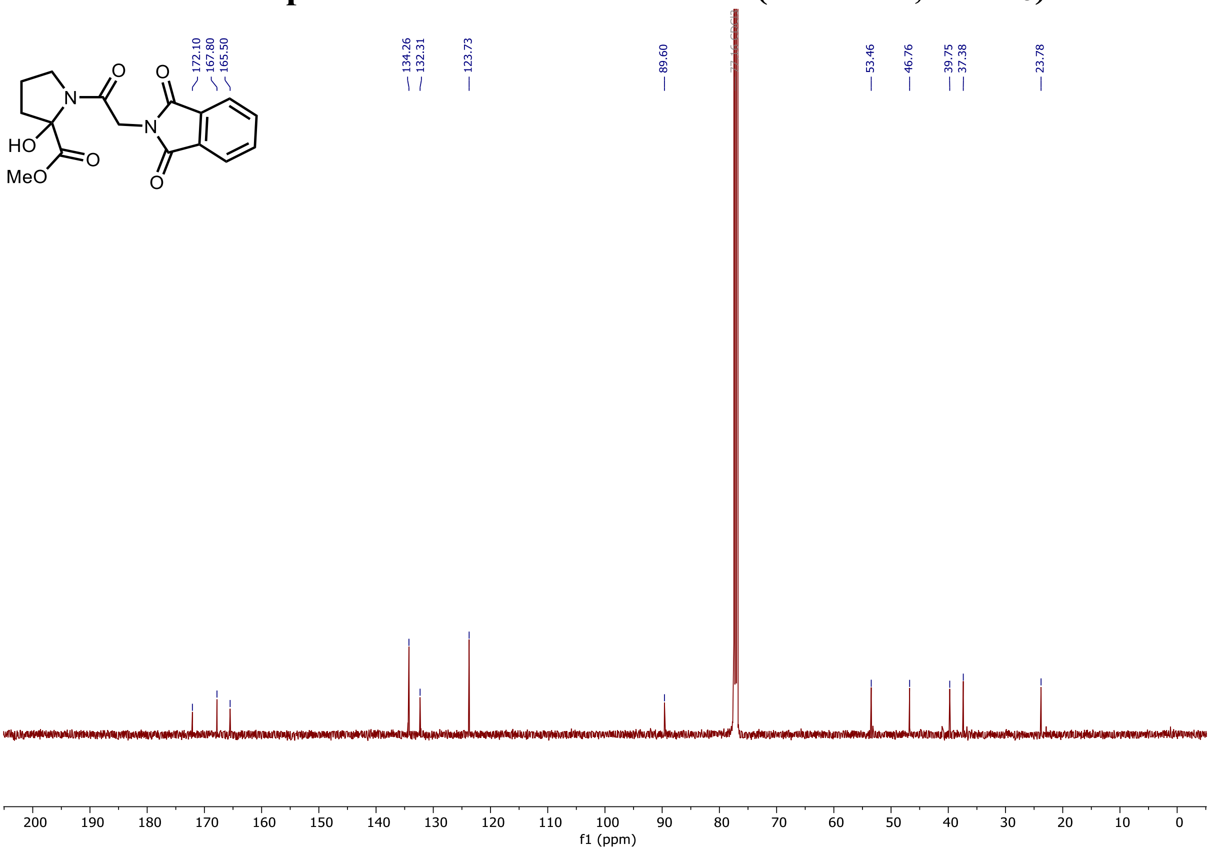

2.120. ^{13}C DEPT-135 Spectrum for Hemiaminal S-4 ($CDCl_3$)

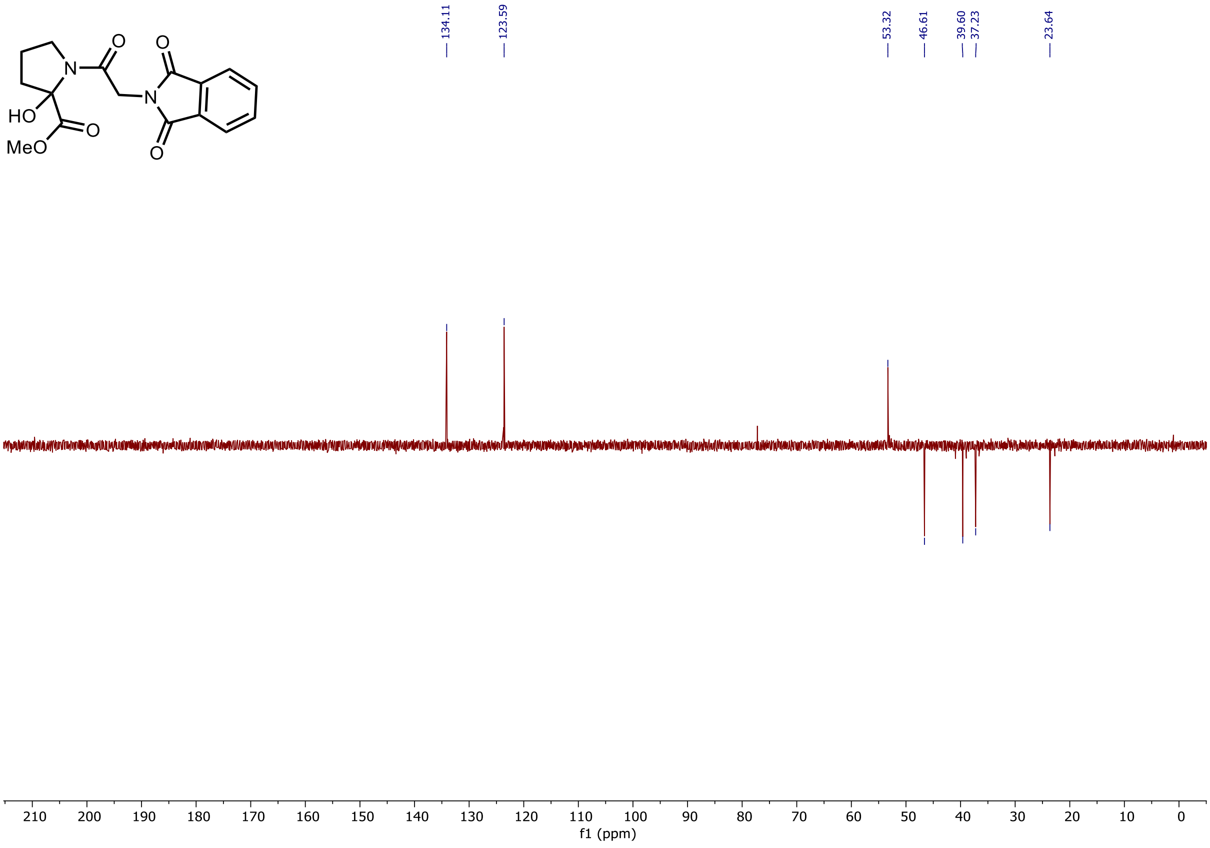

2.121. ^{1}H-^{1}H COSY Spectrum for Hemiaminal S-4 ($CDCl_3$)

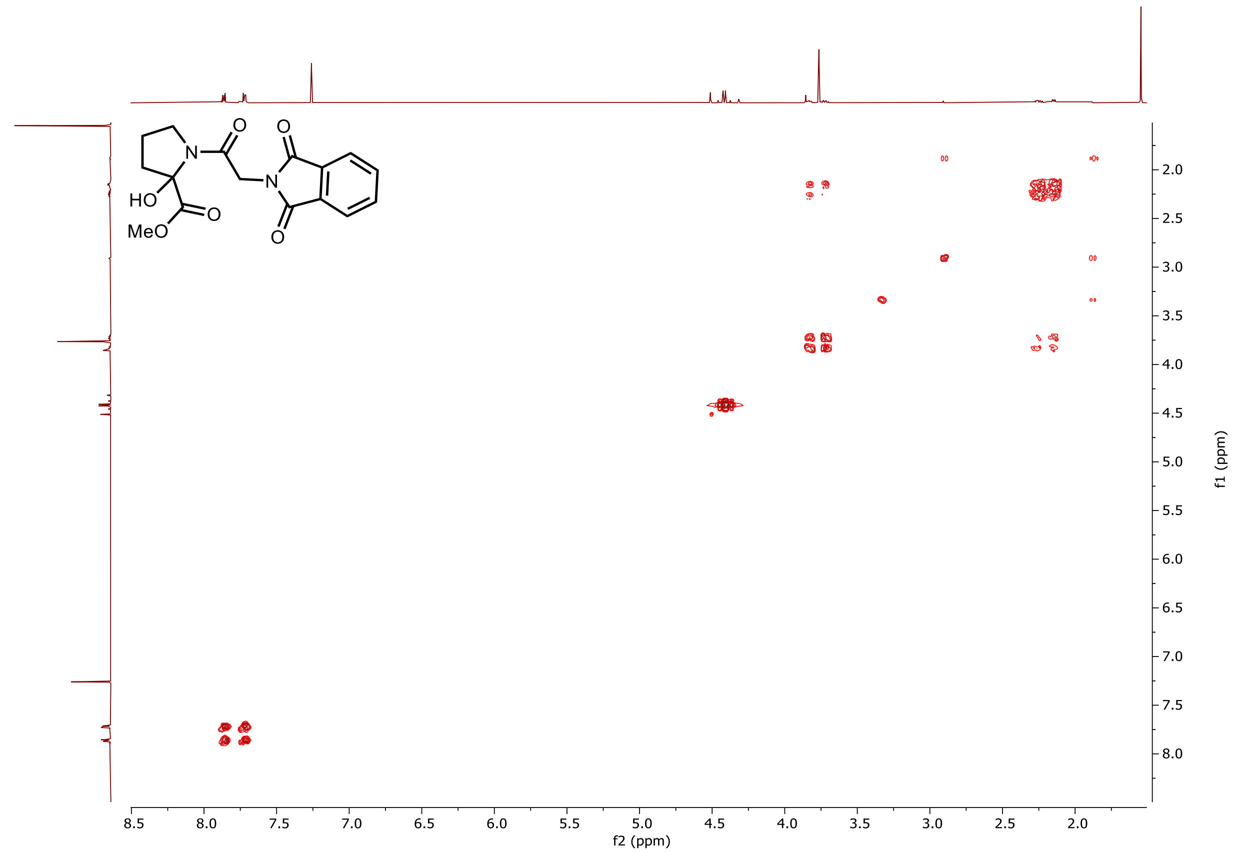

2.122. ^{1}H-^{13}C HSQC Spectrum for Hemiaminal S-4 ($CDCl_3$)

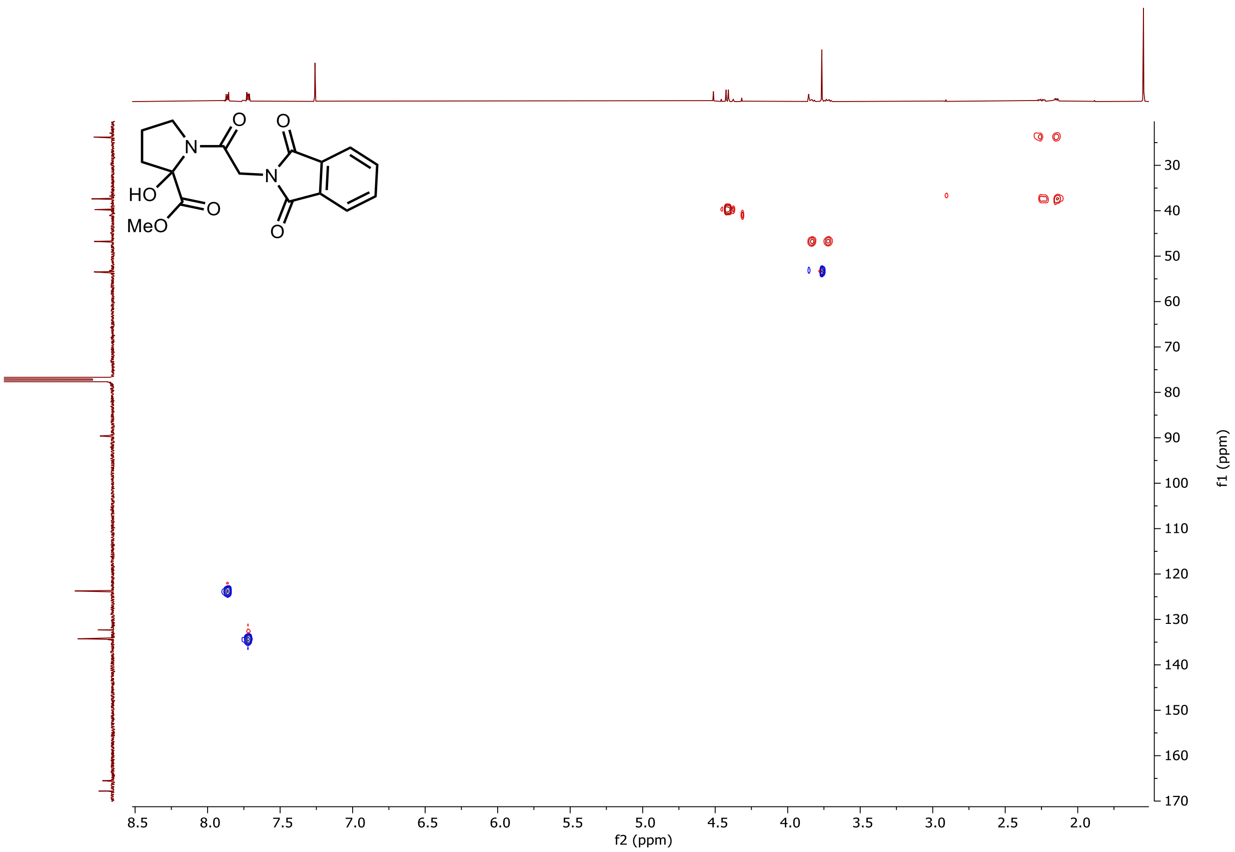

2.123. ^{1}H-^{13}C HMBC Spectrum for Hemiaminal S-4 ($CDCl_3$)

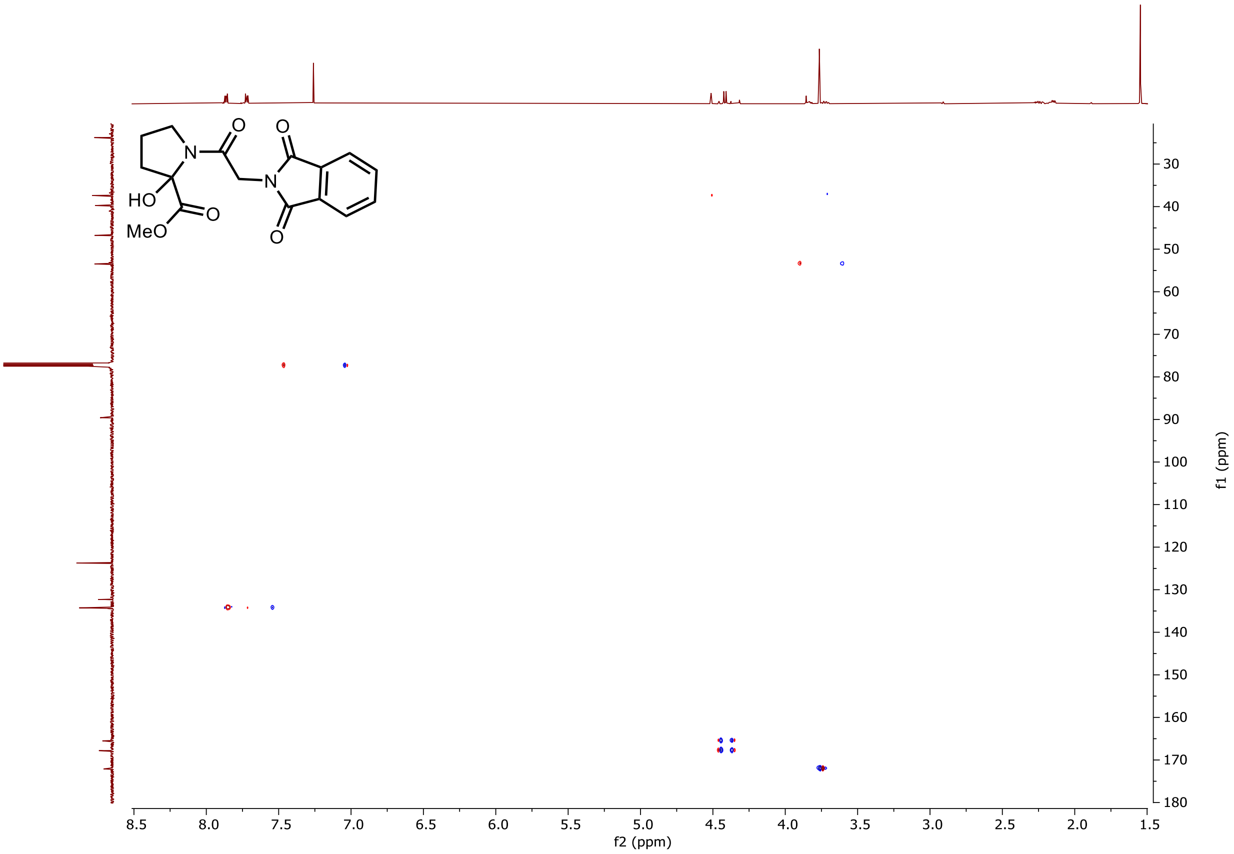

2.124. ^{1}H NMR Spectrum for Lactim Ether S-5 (500 MHz, $CDCl_3$)

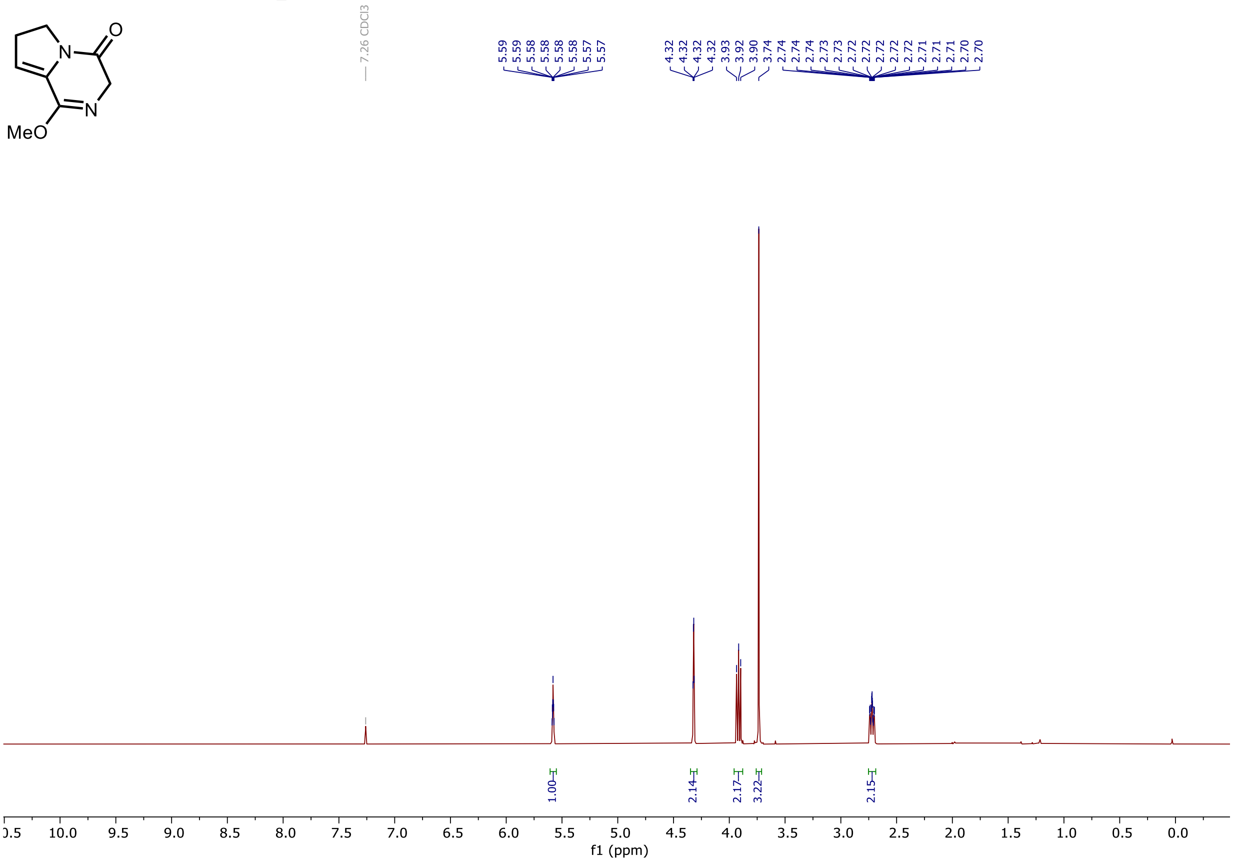

2.125. ^{13}C NMR Spectrum for Lactim Ether S-5 (126 MHz, $CDCl_3$)

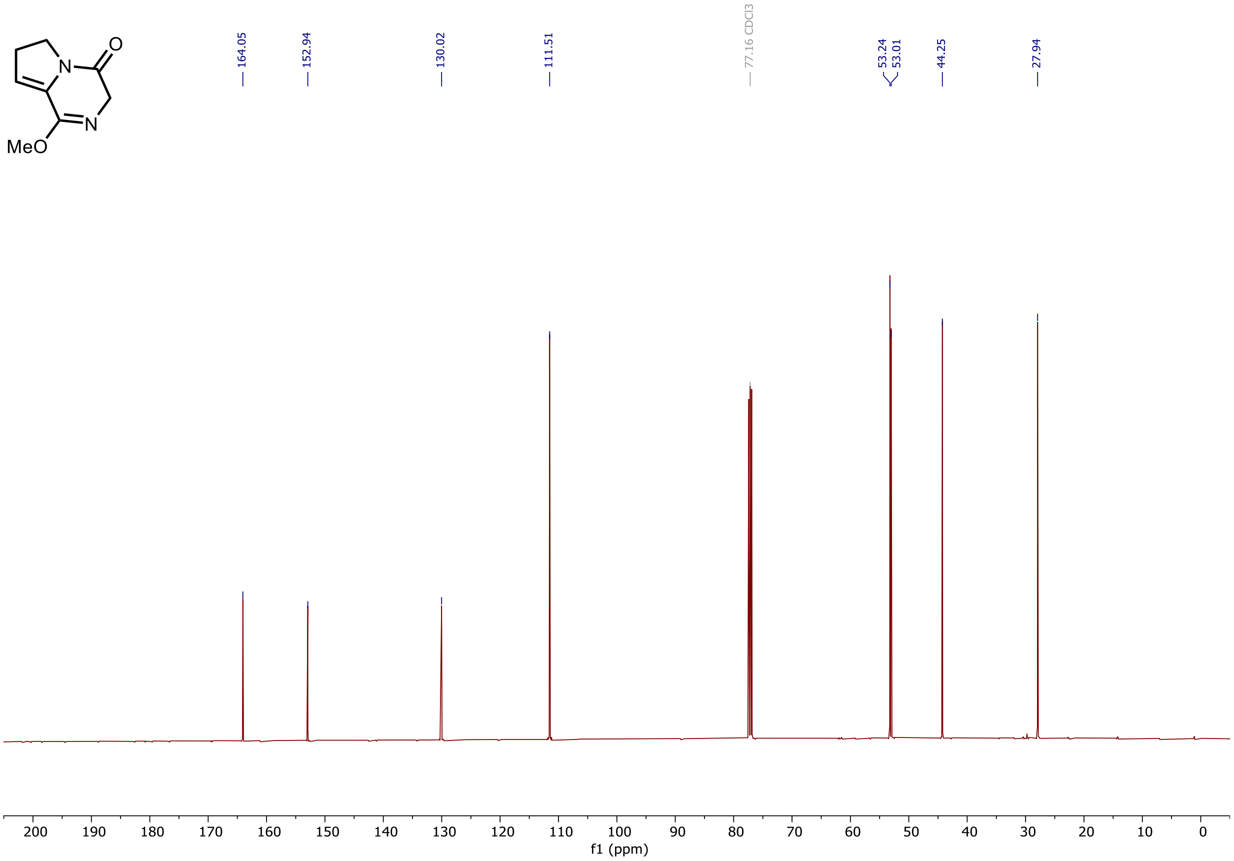

2.126. ^{1}H-^{1}H COSY Spectrum for Lactim Ether S-5 ($CDCl_3$)

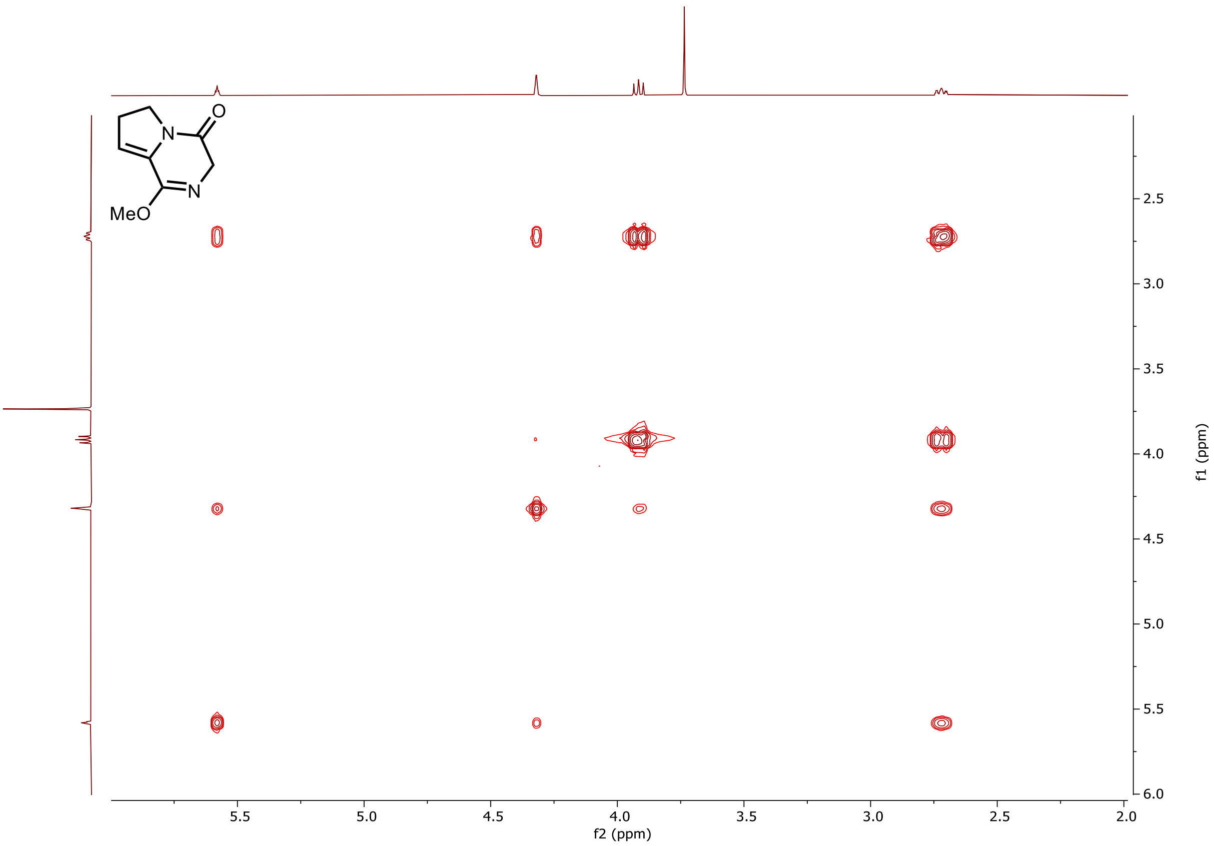

2.127. ^{1}H-^{13}C HSQC Spectrum for Lactim Ether S-5 ($CDCl_3$)

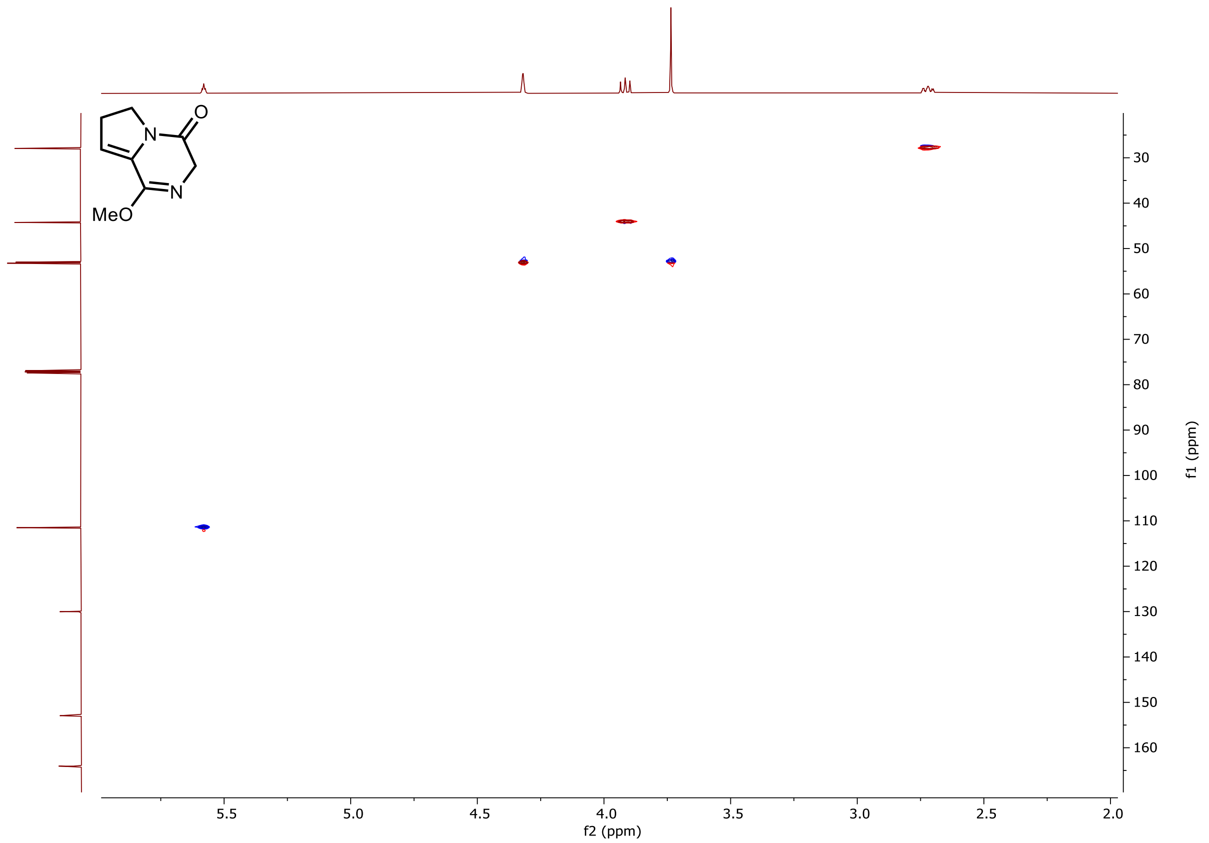

2.128. ^{1}H-^{13}C HMBC Spectrum for Lactim Ether S-5 ($CDCl_3$)

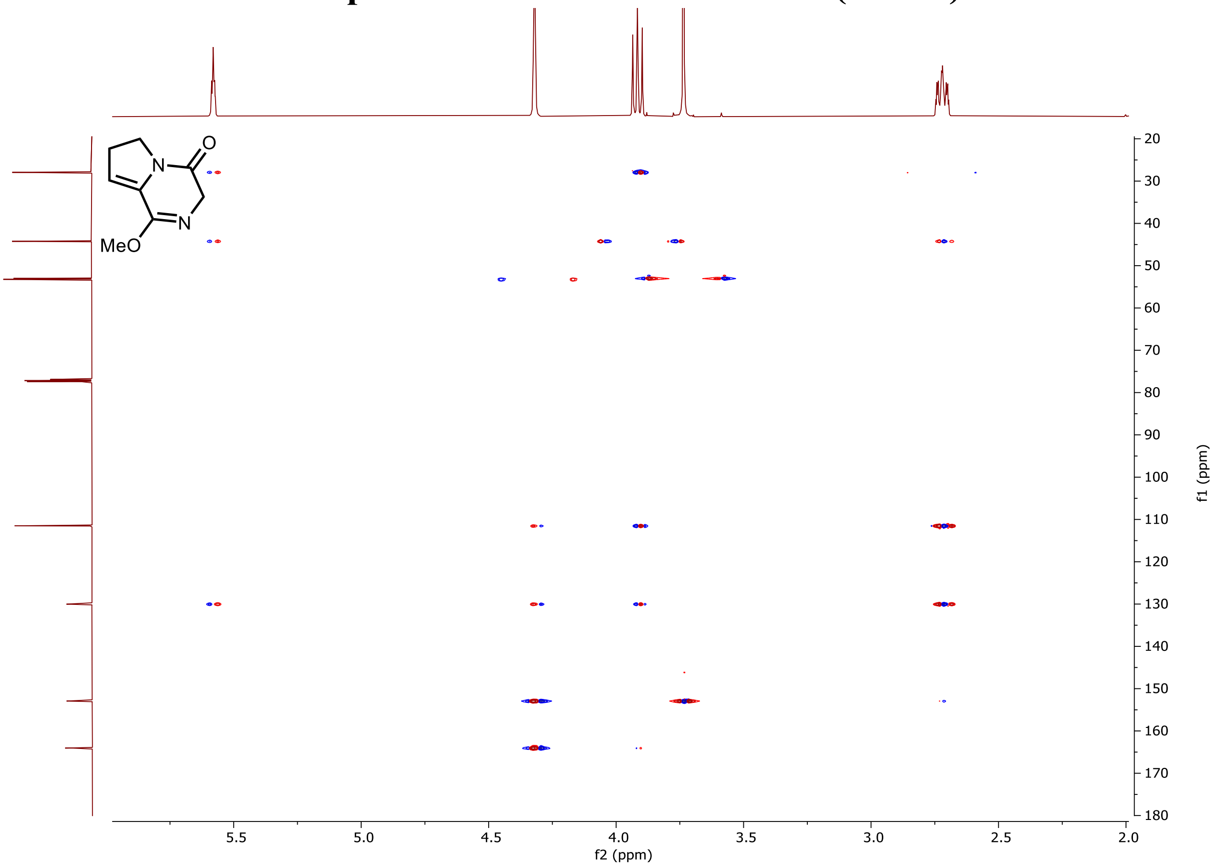

2.129. ^{1}H NMR Spectrum for impure Tetrafluoroborate S-6 (500 MHz, $CDCl_3$)

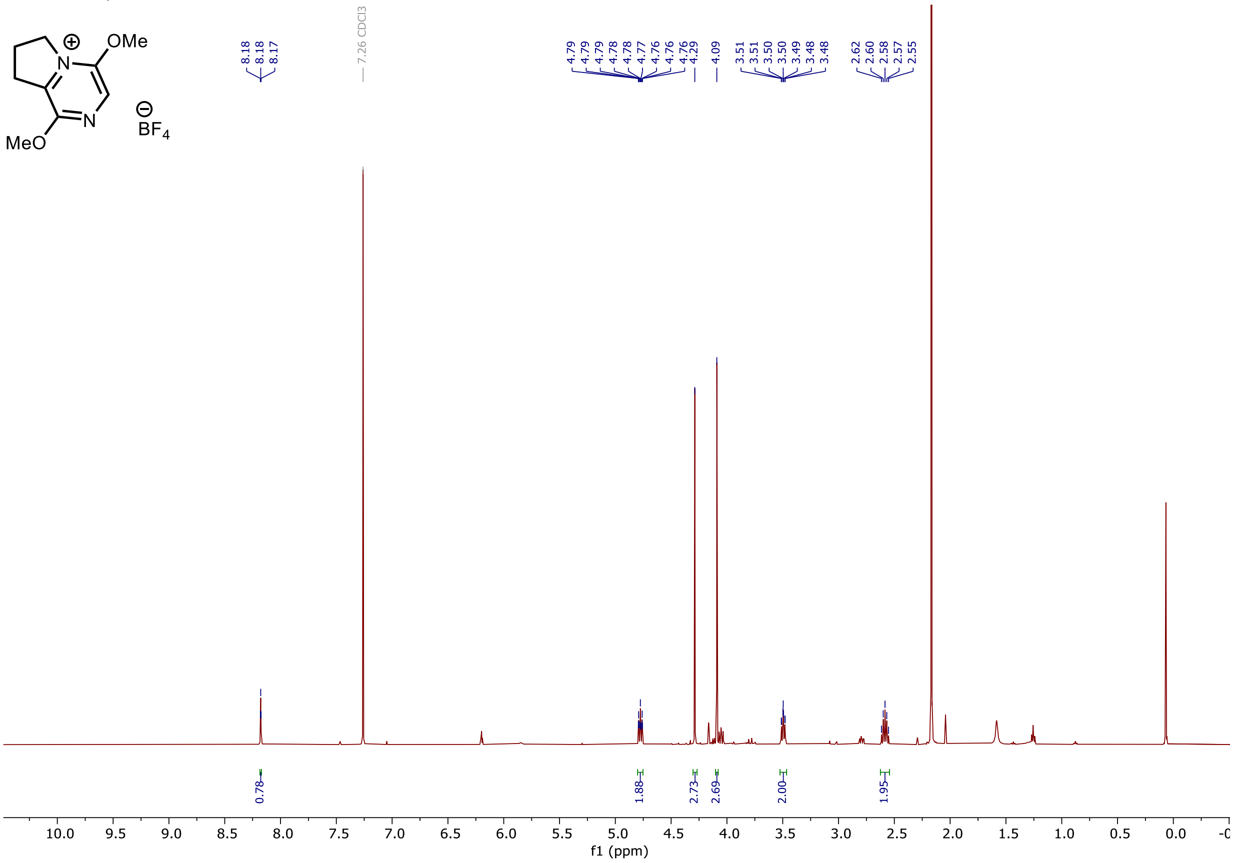

2.130. ^{11}B NMR Spectrum for impure Tetrafluoroborate S-6 (128 MHz, CDCl3)

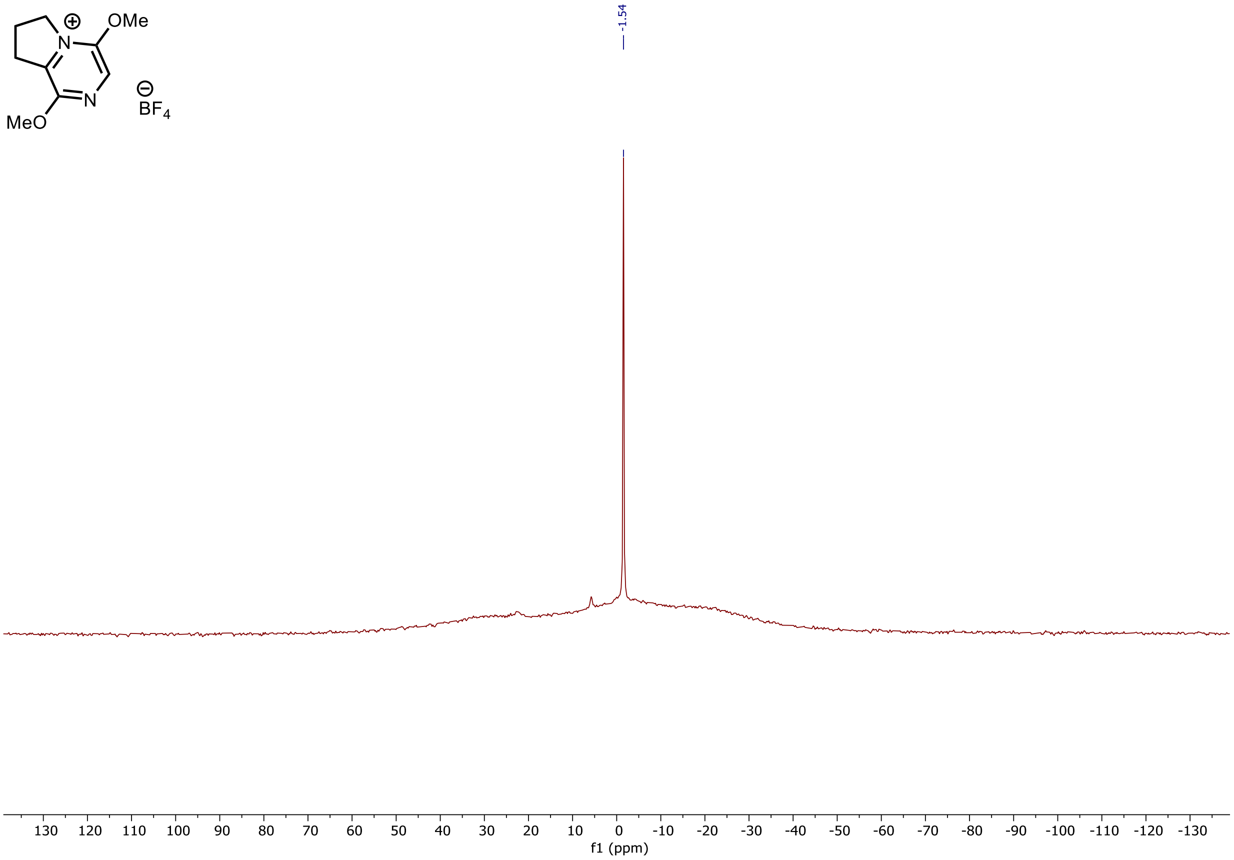

2.131. ^{13}C NMR Spectrum for impure Tetrafluoroborate S-6 (126 MHz, $CDCl_3$)

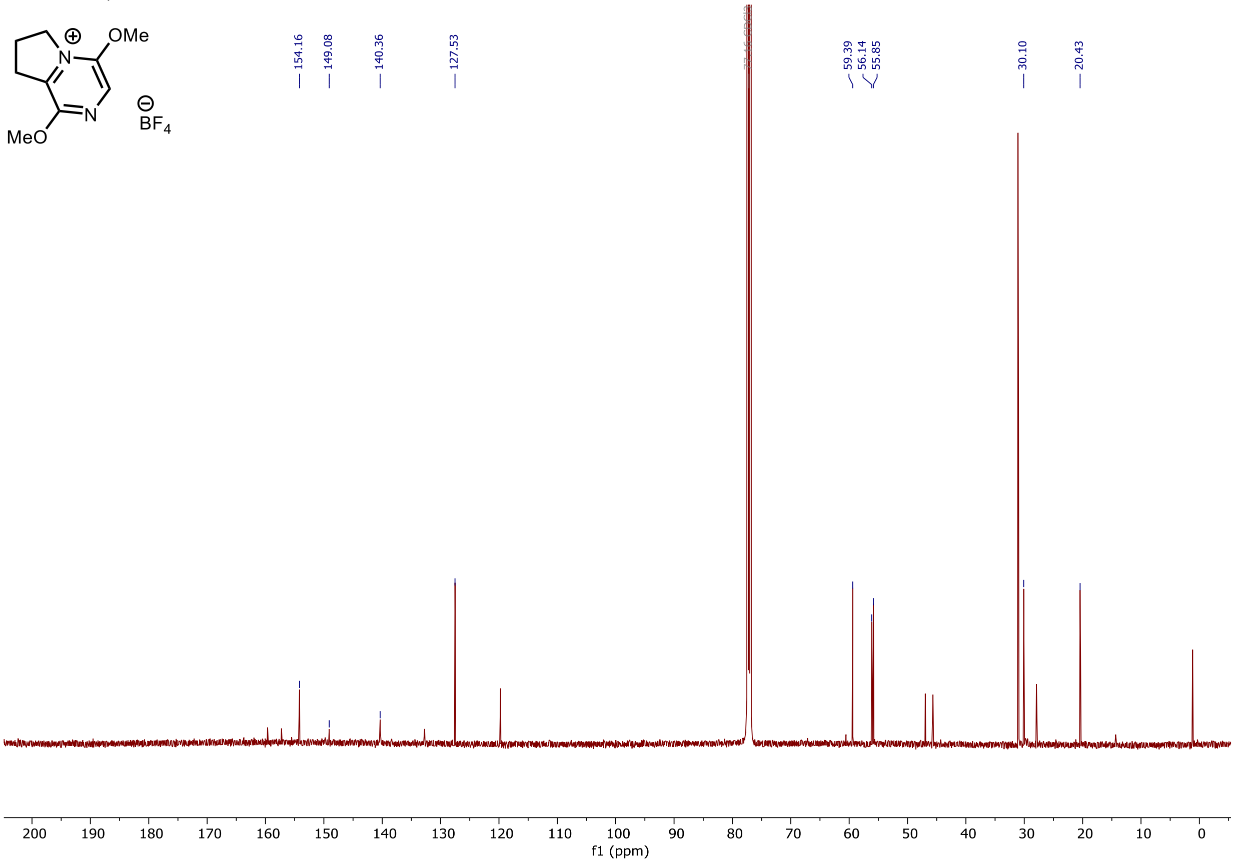

2.132. ^{1}H-^{1}H COSY Spectrum for impure Tetrafluoroborate S-6 ($CDCl_3$)

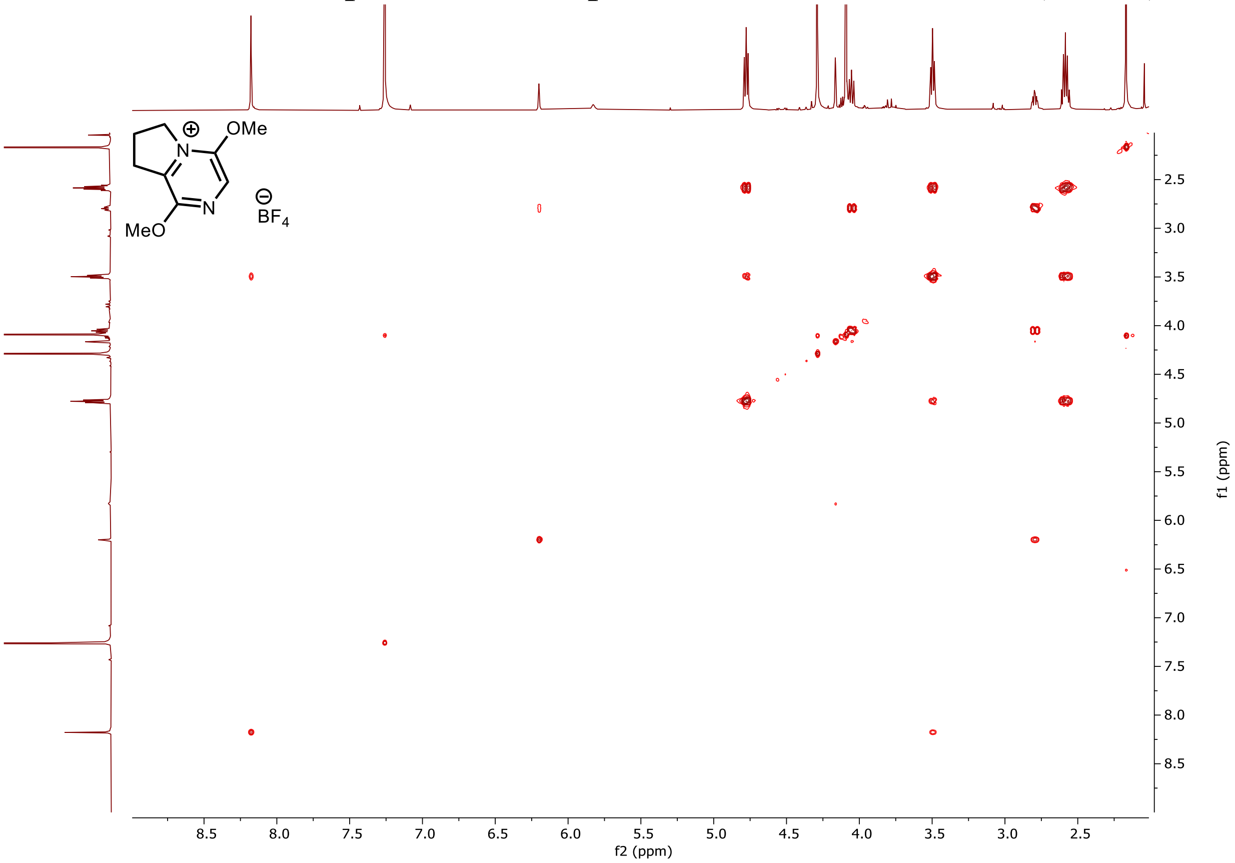

2.133. ^{1}H-^{13}C HSQC Spectrum for impure Tetrafluoroborate S-6 ($CDCl_3$)

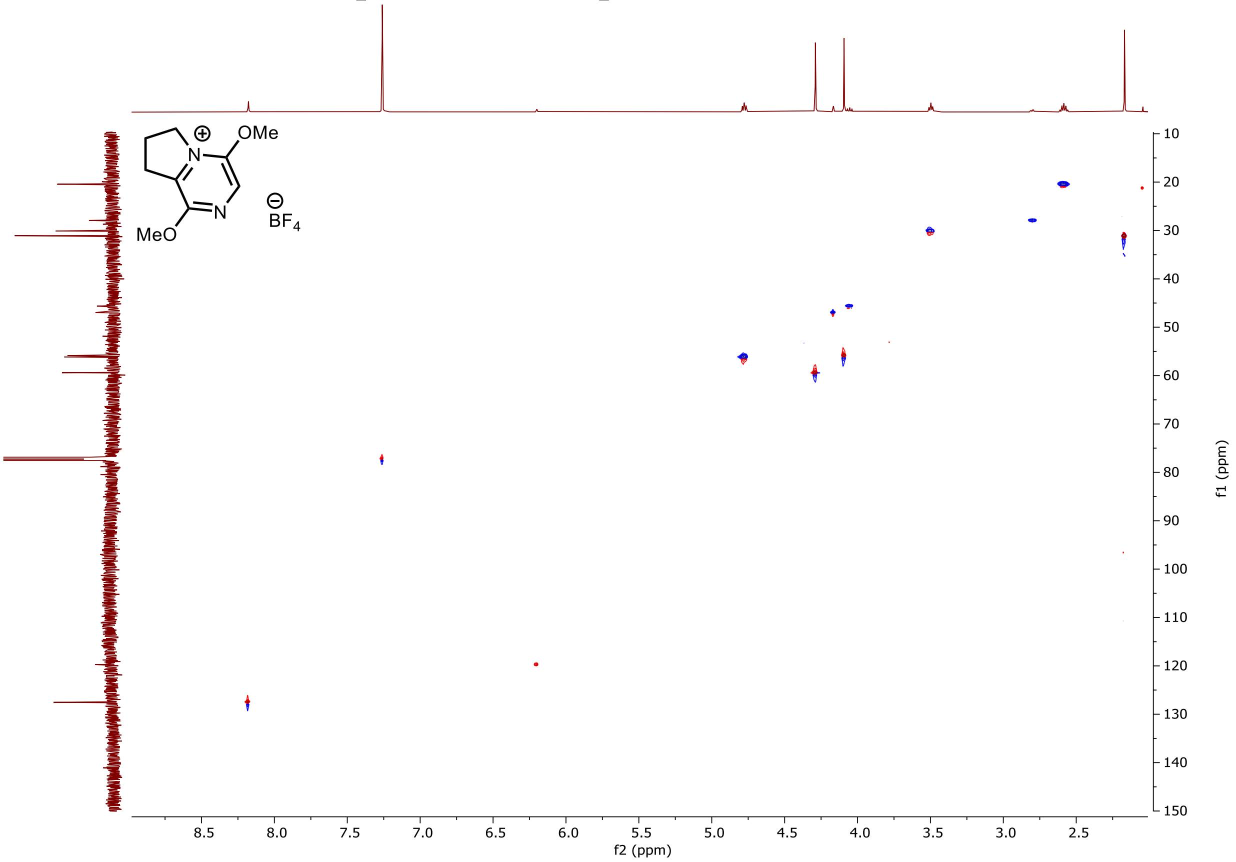

2.134. ^{1}H-^{13}C HMBC Spectrum for impure Tetrafluoroborate S-6 ($CDCl_3$)

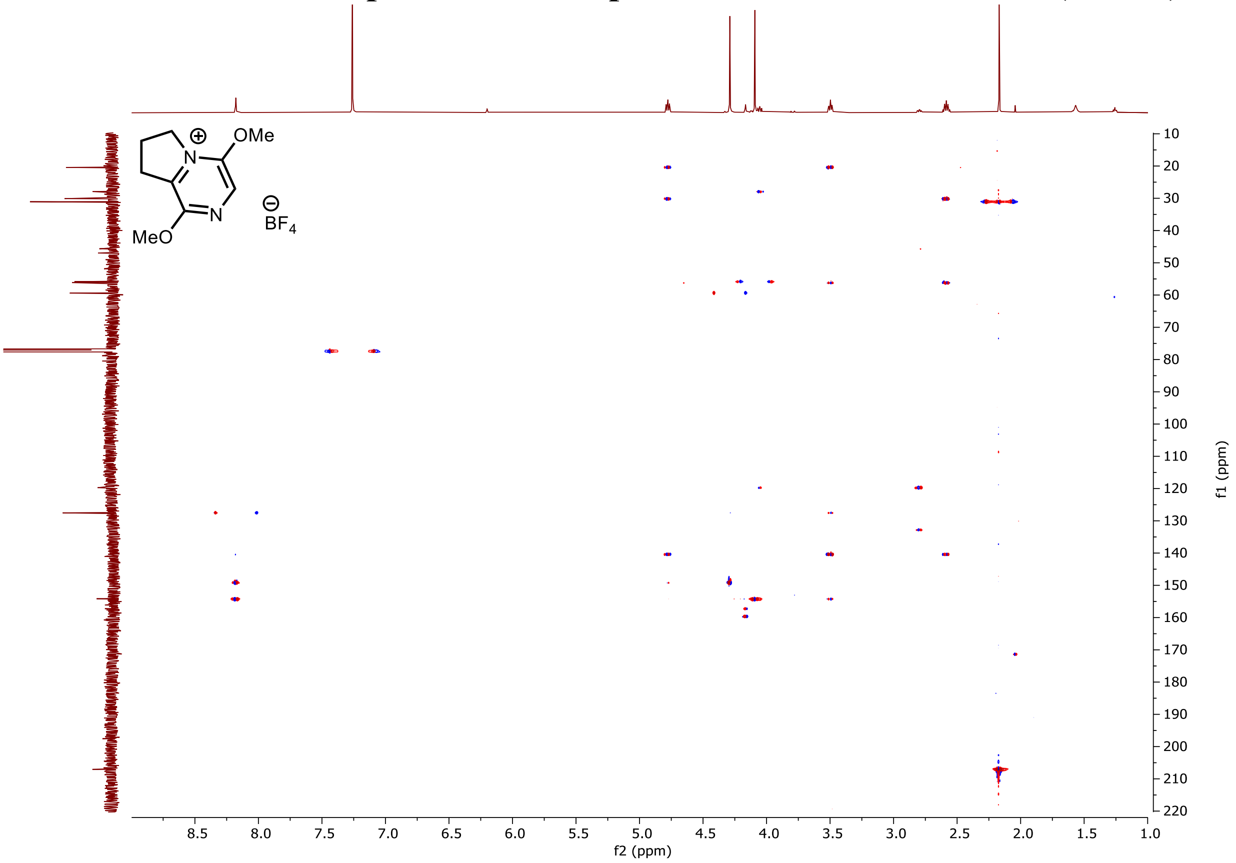

2.135. ^{19}F NMR Spectrum for impure Tetrafluoroborate S-6 (376 MHz, $CDCl_3$)

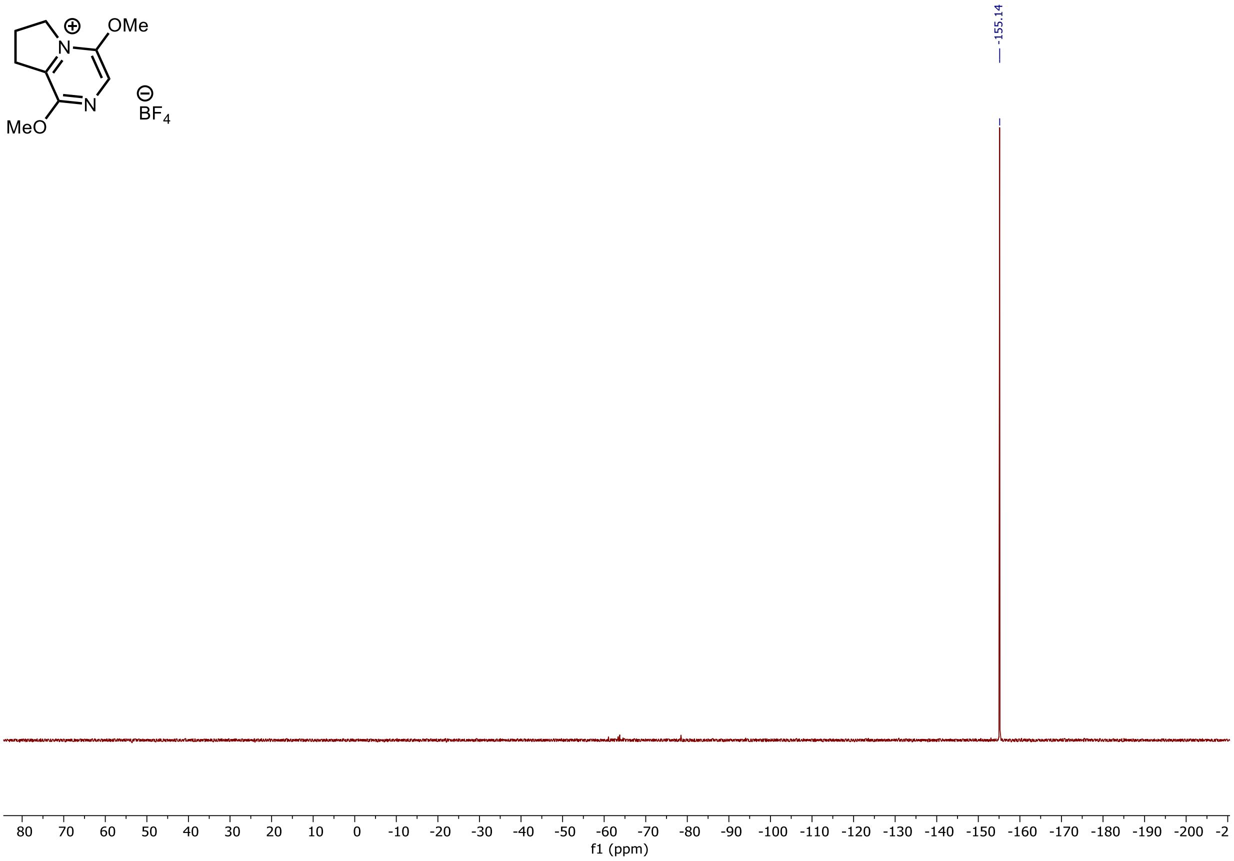

2.136. ^{1}H NMR Spectrum for impure Pyrazinone 18 (500 MHz, $CDCl_3$)

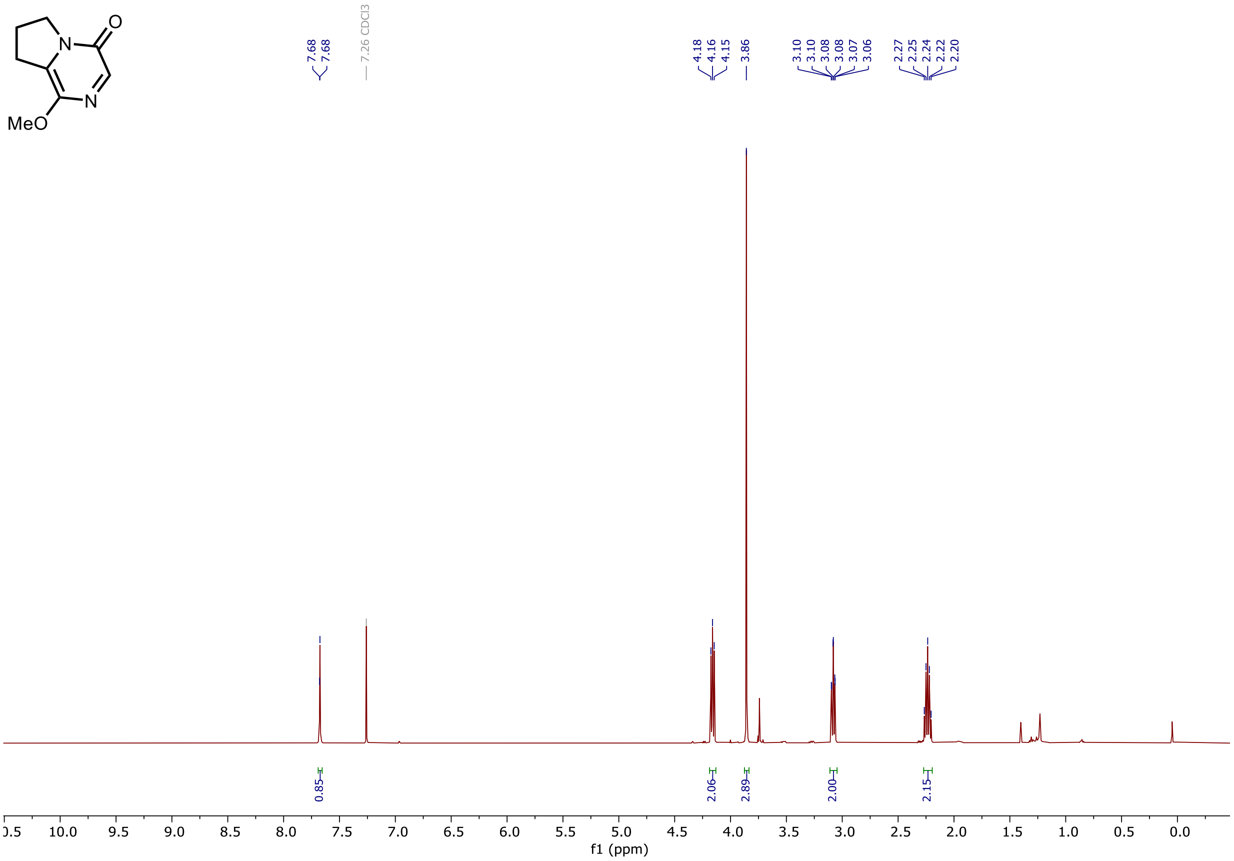

2.137. ^{13}C NMR Spectrum for impure Pyrazinone 18 (126 MHz, $CDCl_3$)

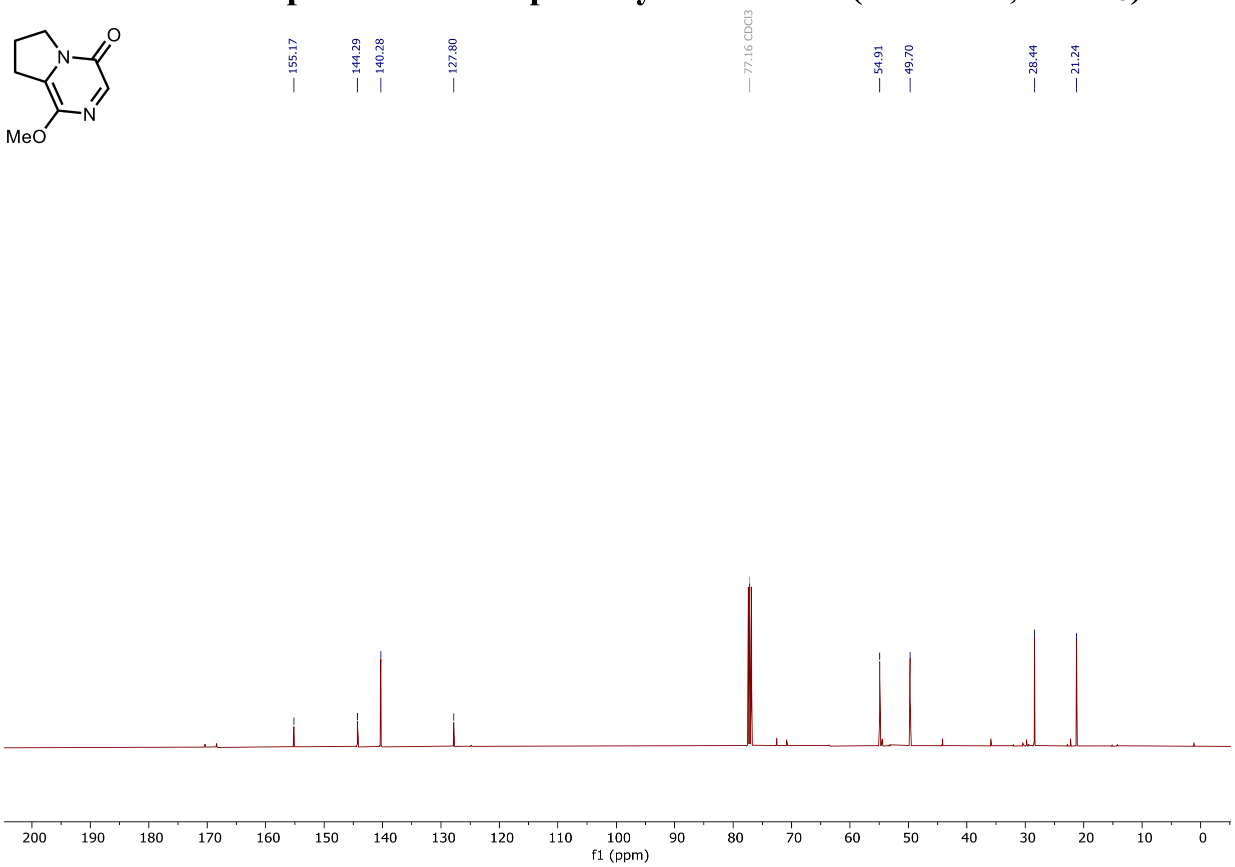

2.138. ^1H-^1H COSY Spectrum for impure Pyrazinone 18 ($CDCl_3$)

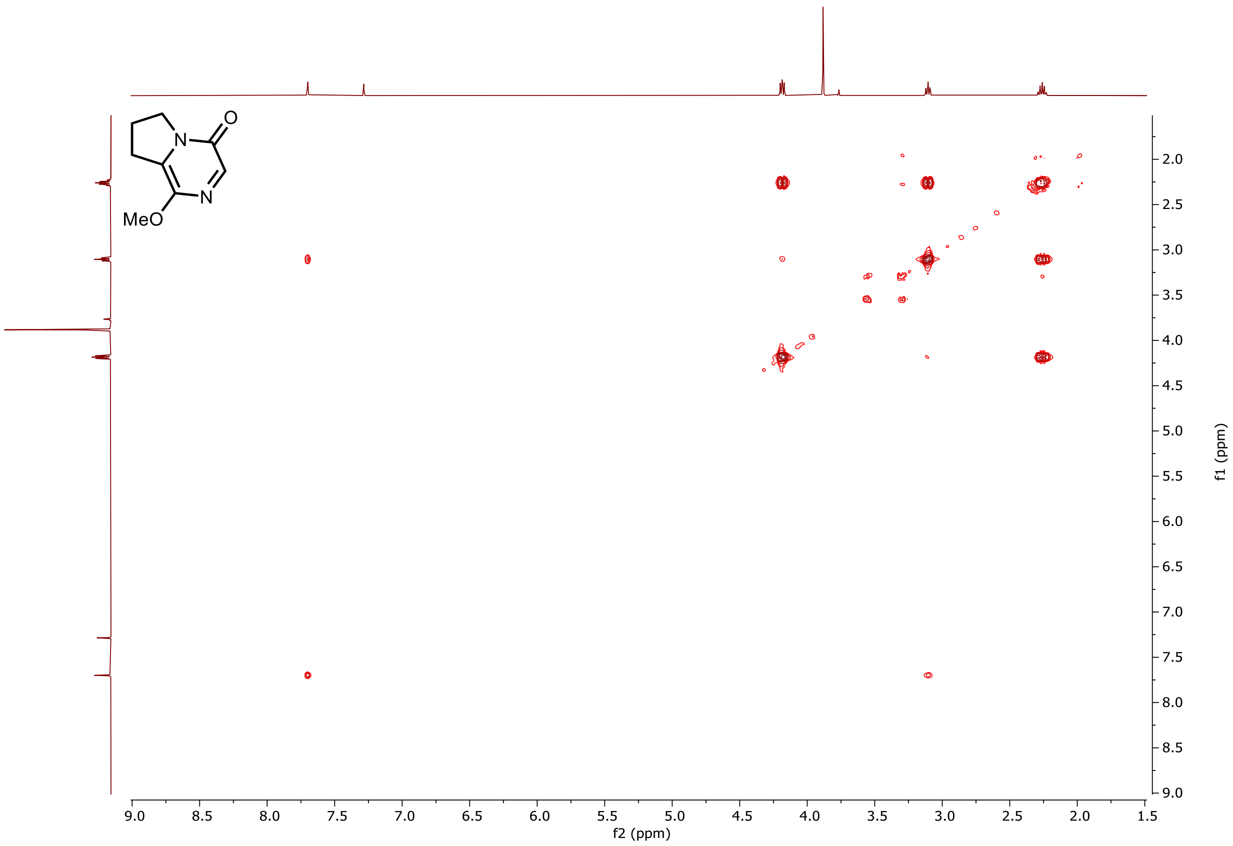

2.139. ^{1}H-^{13}C HSQC Spectrum for impure Pyrazinone 18 ($CDCl_3$)

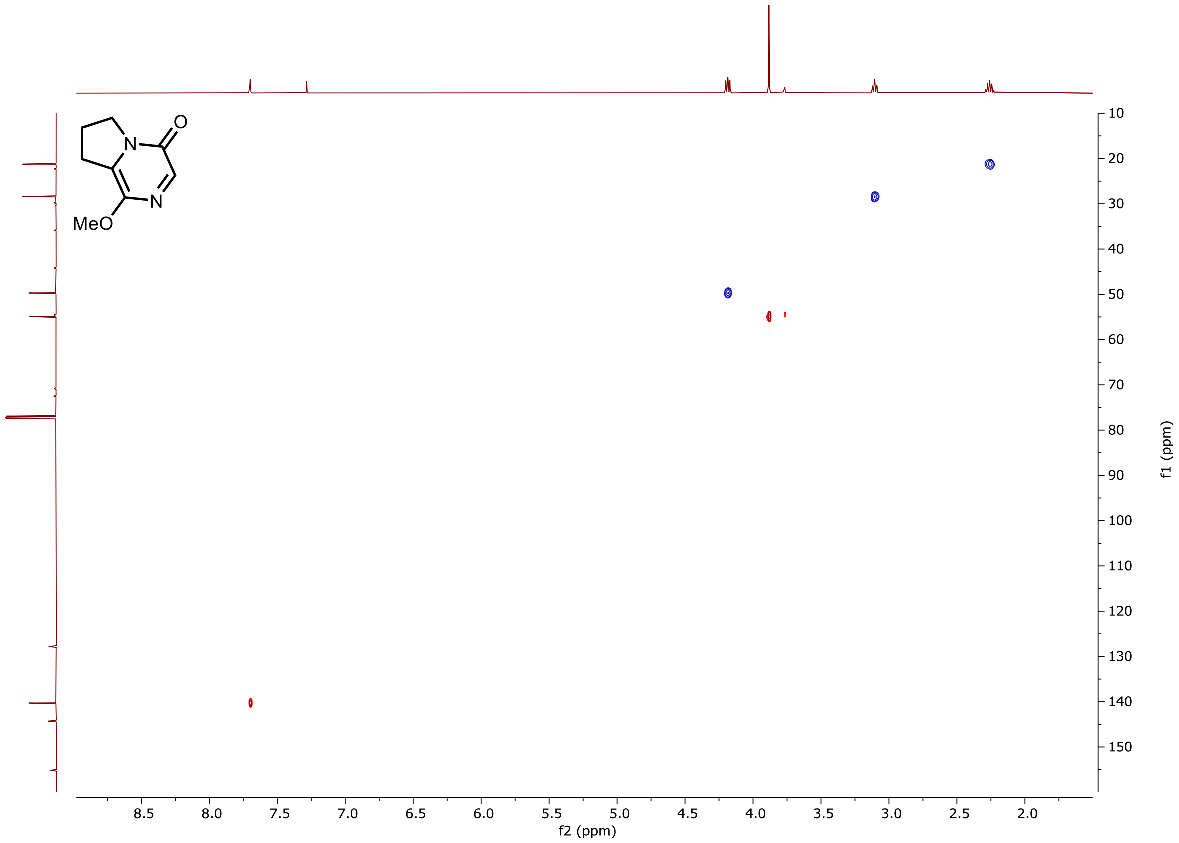

2.140. ^{1}H-^{13}C HMBC Spectrum for impure Pyrazinone 18 ($CDCl_3$)

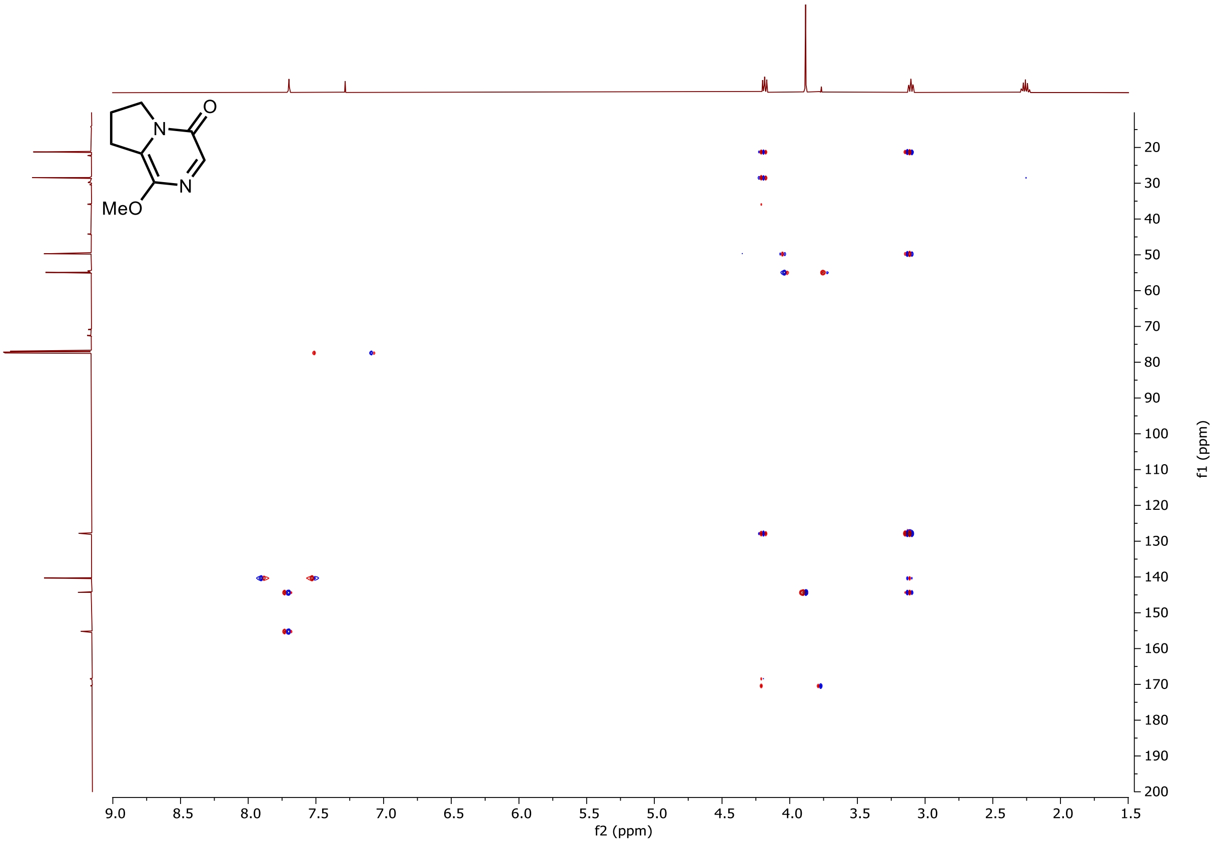

2.141. ^{1}H NMR Spectrum for impure Compound S-7 (600 MHz, $CDCl_3$)

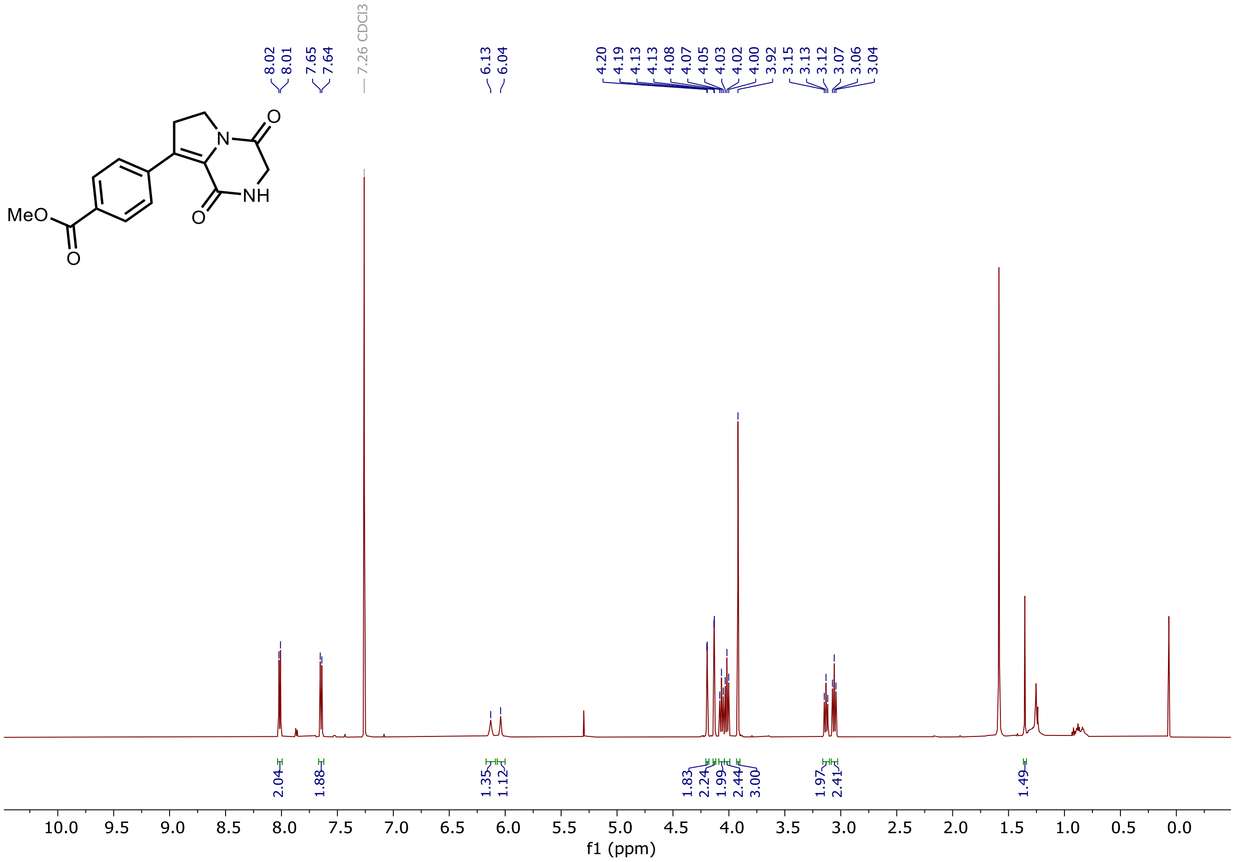

2.142. ^{13}C NMR Spectrum for impure Compound S-7 (126 MHz, $CDCl_3$)

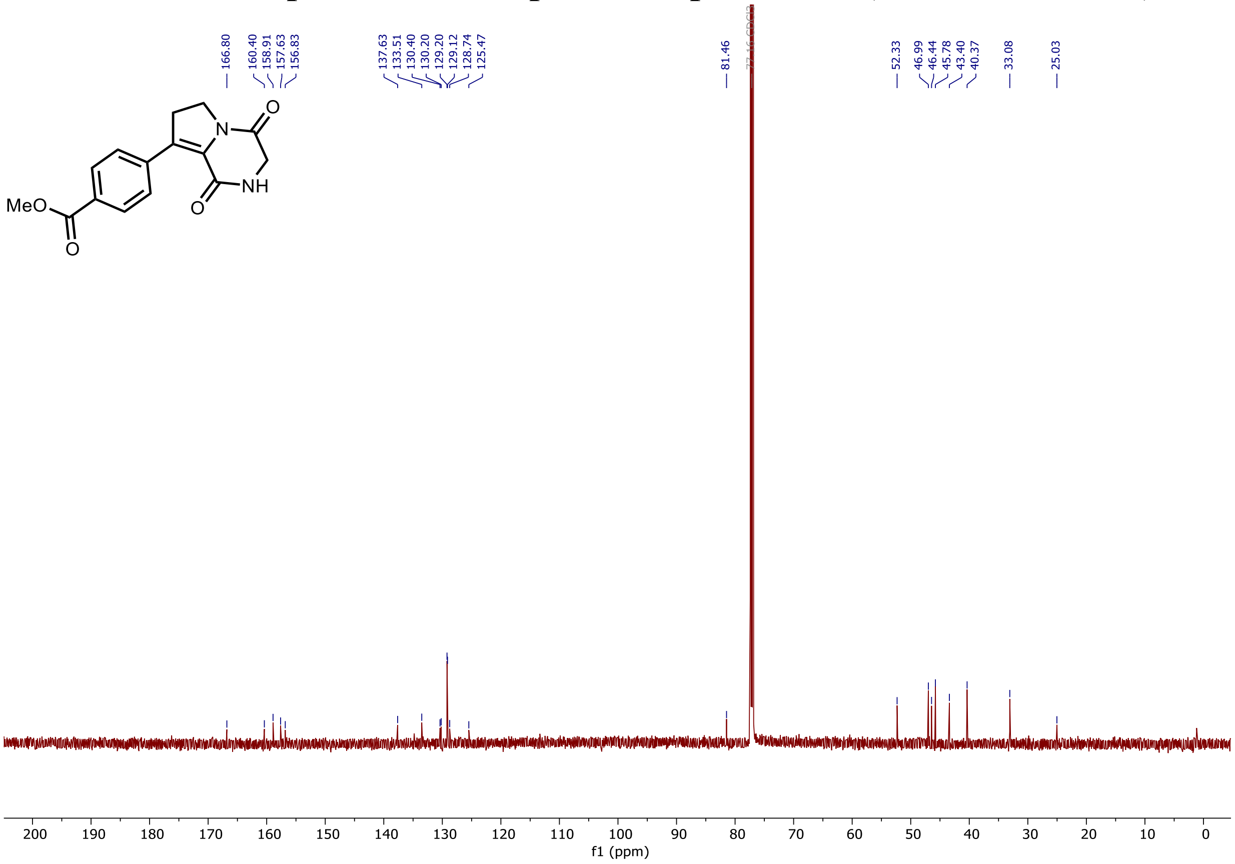

2.143. ^{13}C DEPT-135 Spectrum for impure Compound S-7 ($CDCl_3$)

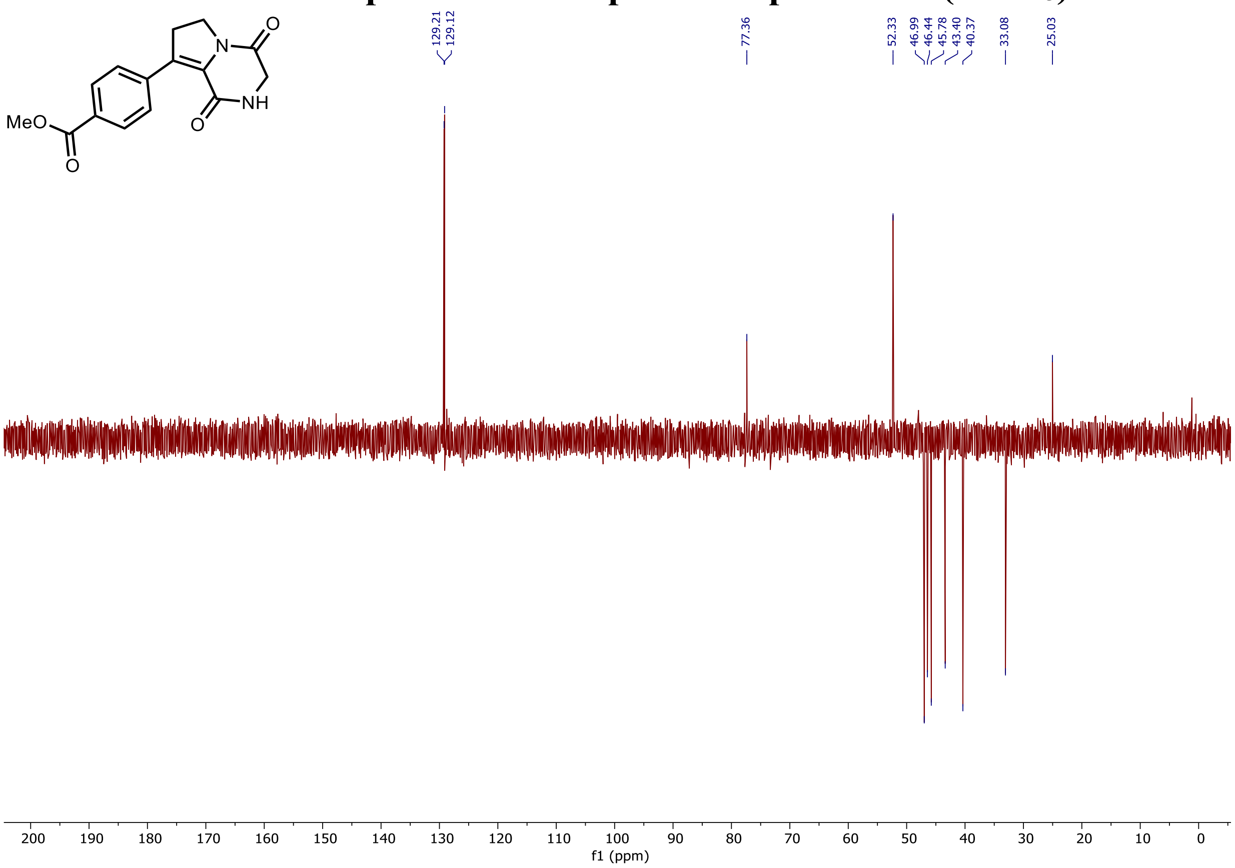

2.144. ^{1}H-^{1}H COSY Spectrum for impure Compound S-7 ($CDCl_3$)

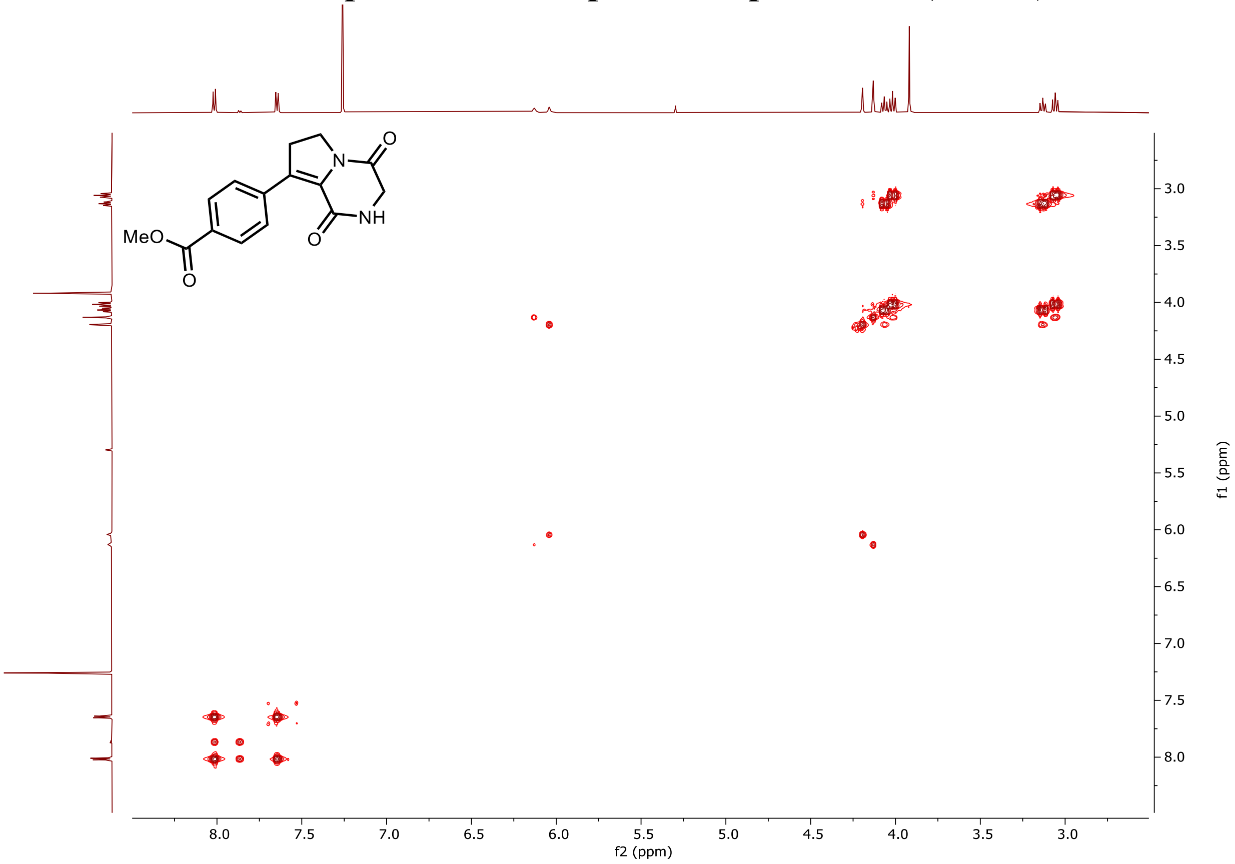

2.145. ^{1}H-^{13}C HSQC Spectrum for impure Compound S-7 ($CDCl_3$)

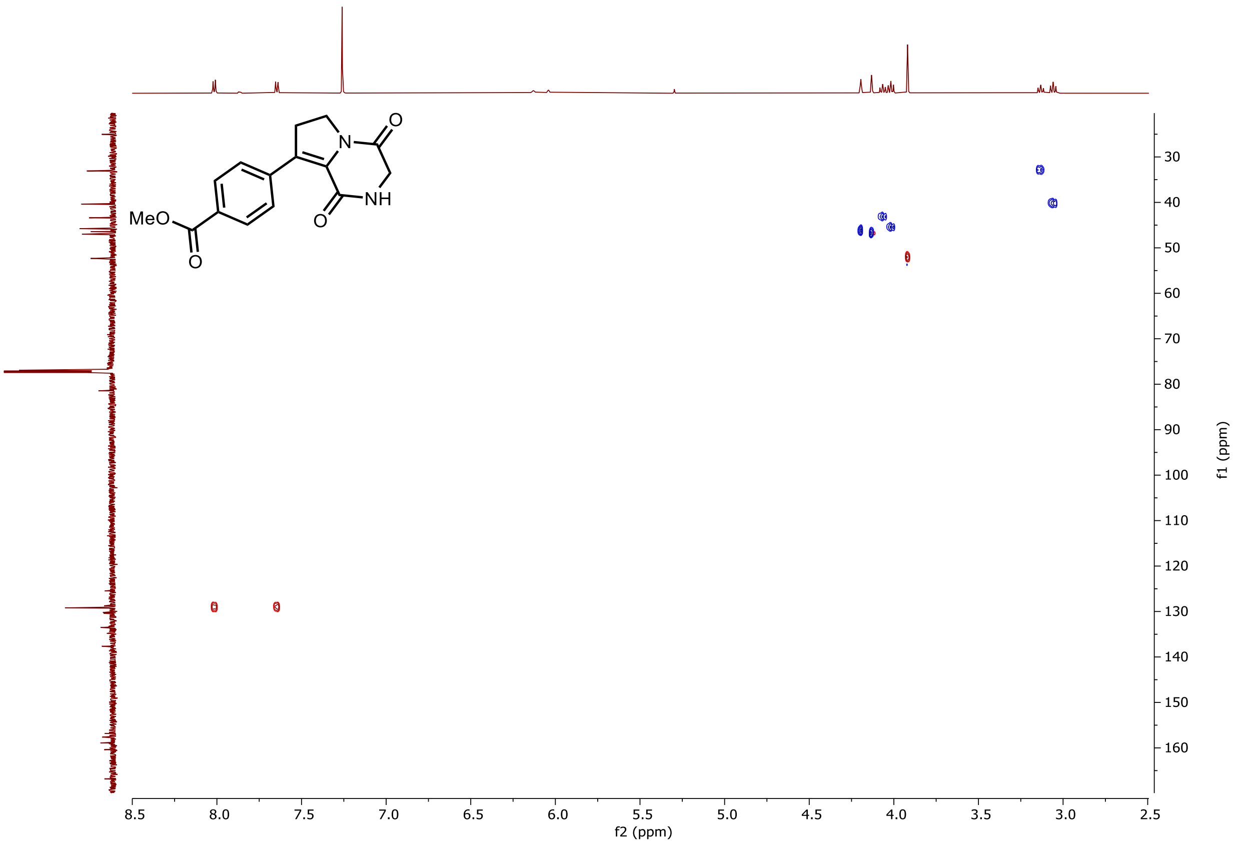

2.146. ^{1}H-^{13}C HMBC Spectrum for impure Compound S-7 ($CDCl_3$)

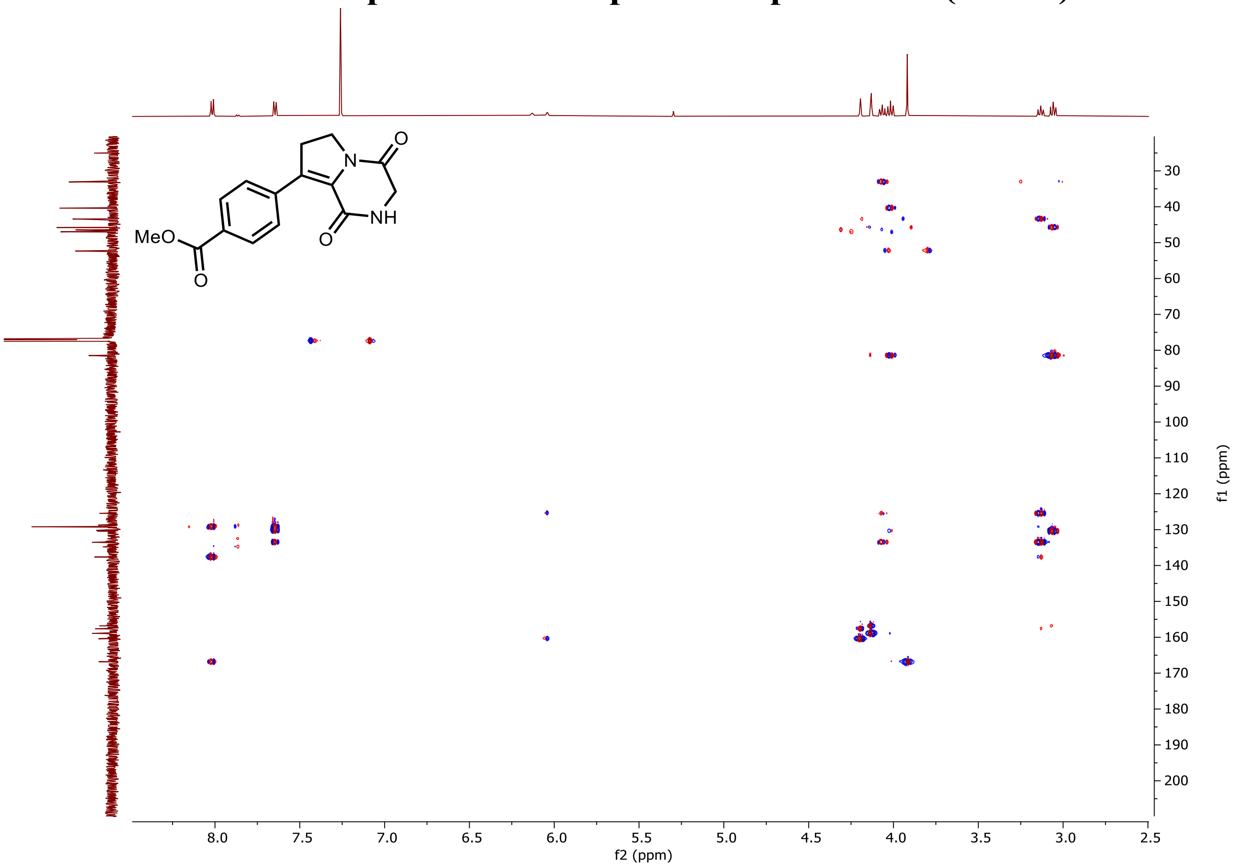

2.147. ^{1}H NMR Spectrum for Organotin S-9 (600 MHz, $CDCl_3$)

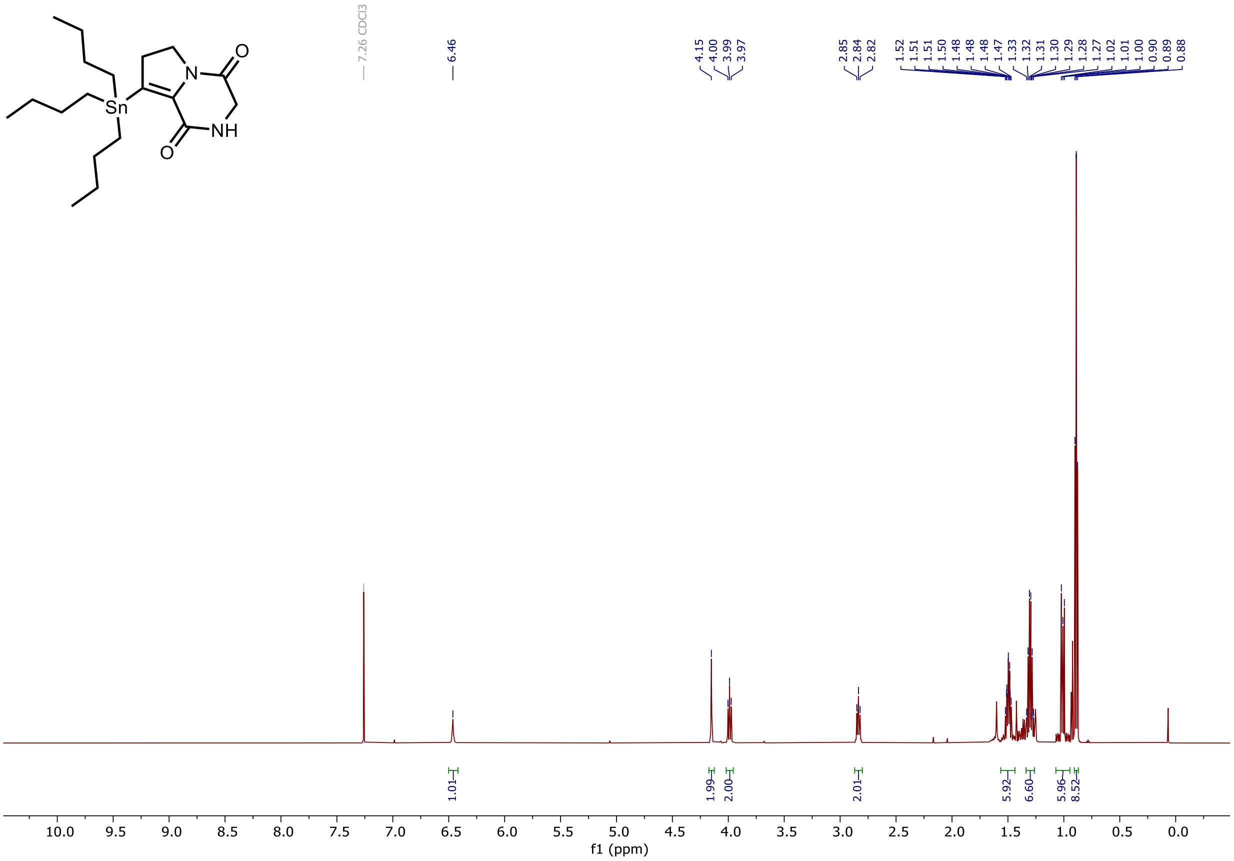

2.148. ^{13}C NMR Spectrum for Organotin S-9 (151 MHz, $CDCl_3$)

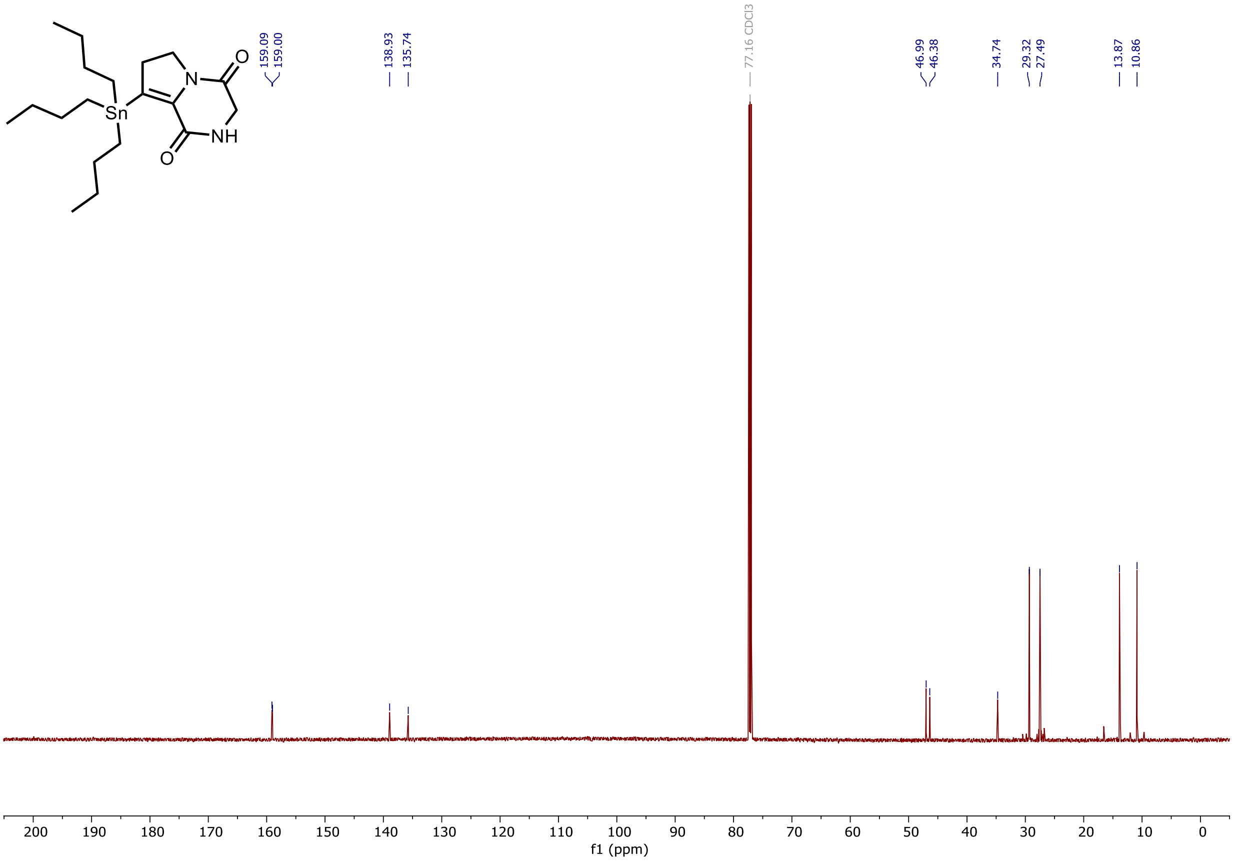

2.149. ^{1}H-^{1}H COSY Spectrum for Organotin S-9 ($CDCl_3$)

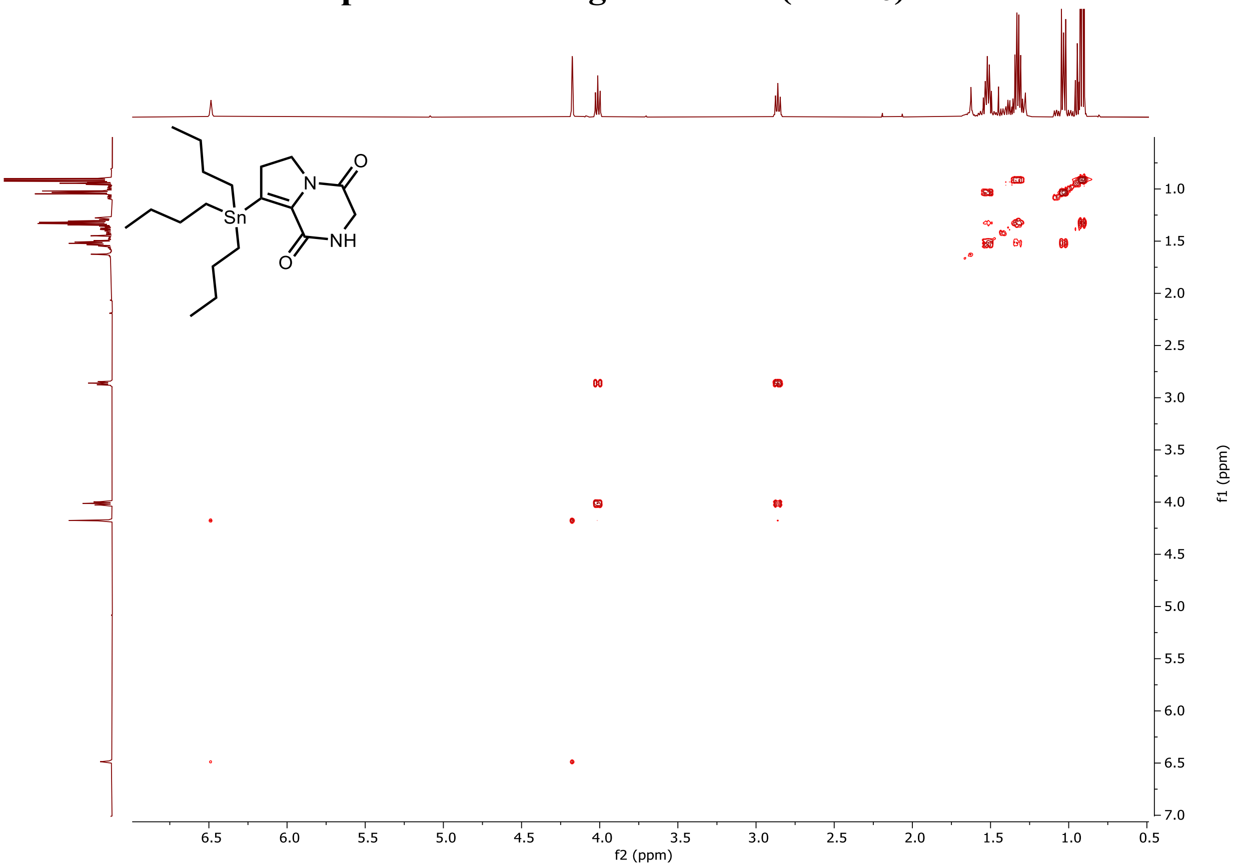

2.150. ^{1}H-^{13}C HSQC Spectrum for Organotin S-9 ($CDCl_3$)

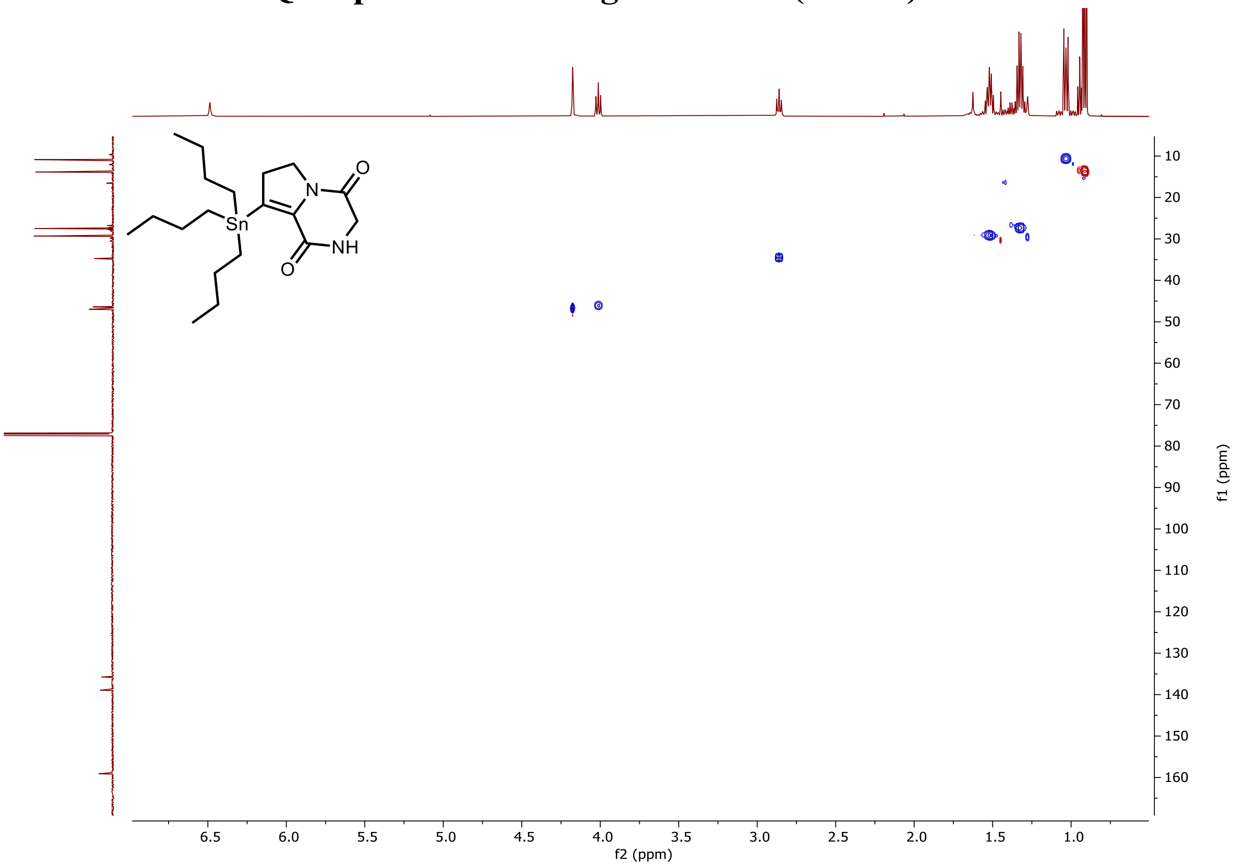

2.151. ^{1}H-^{13}C HMBC Spectrum for Organotin S-9 ($CDCl_3$)

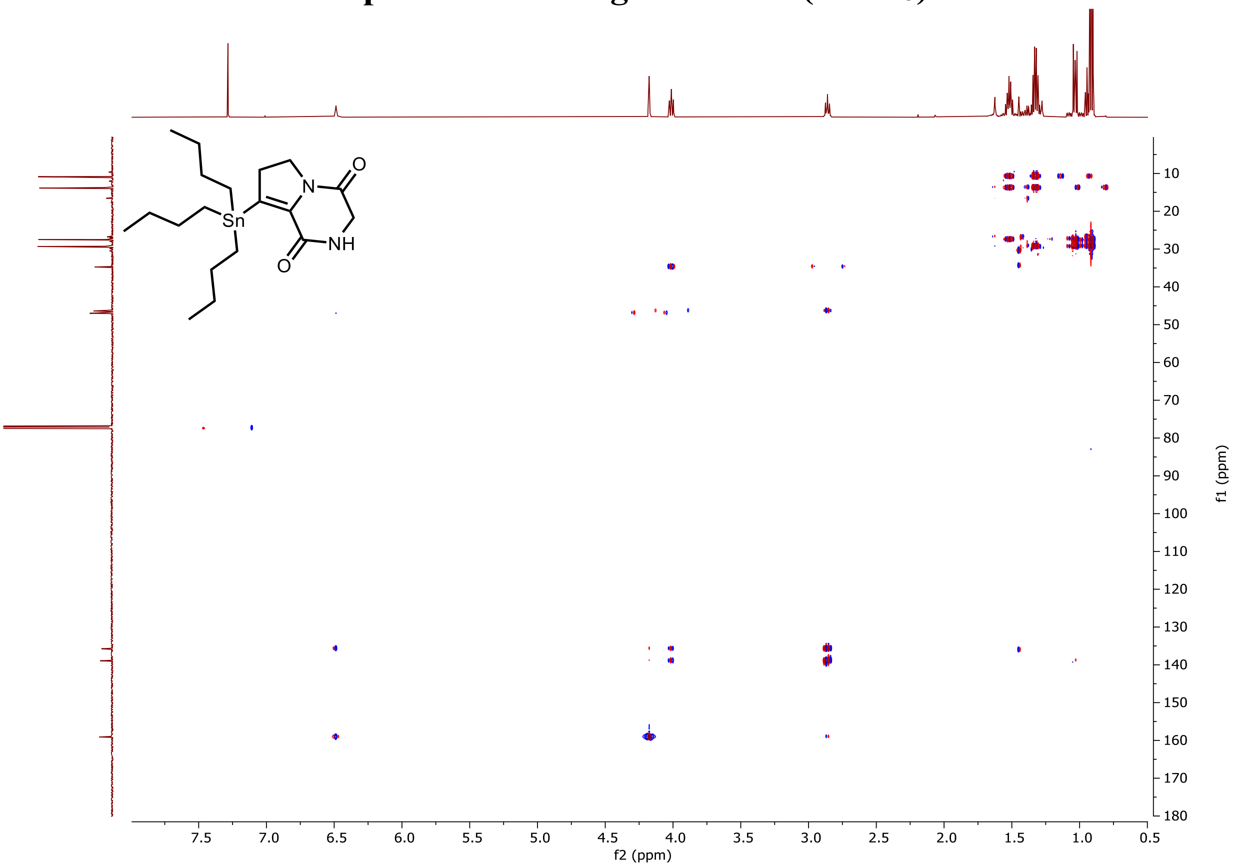

2.152. ^{119}Sn NMR Spectrum for Organotin S-9 (149 MHz, $CDCl_3$)

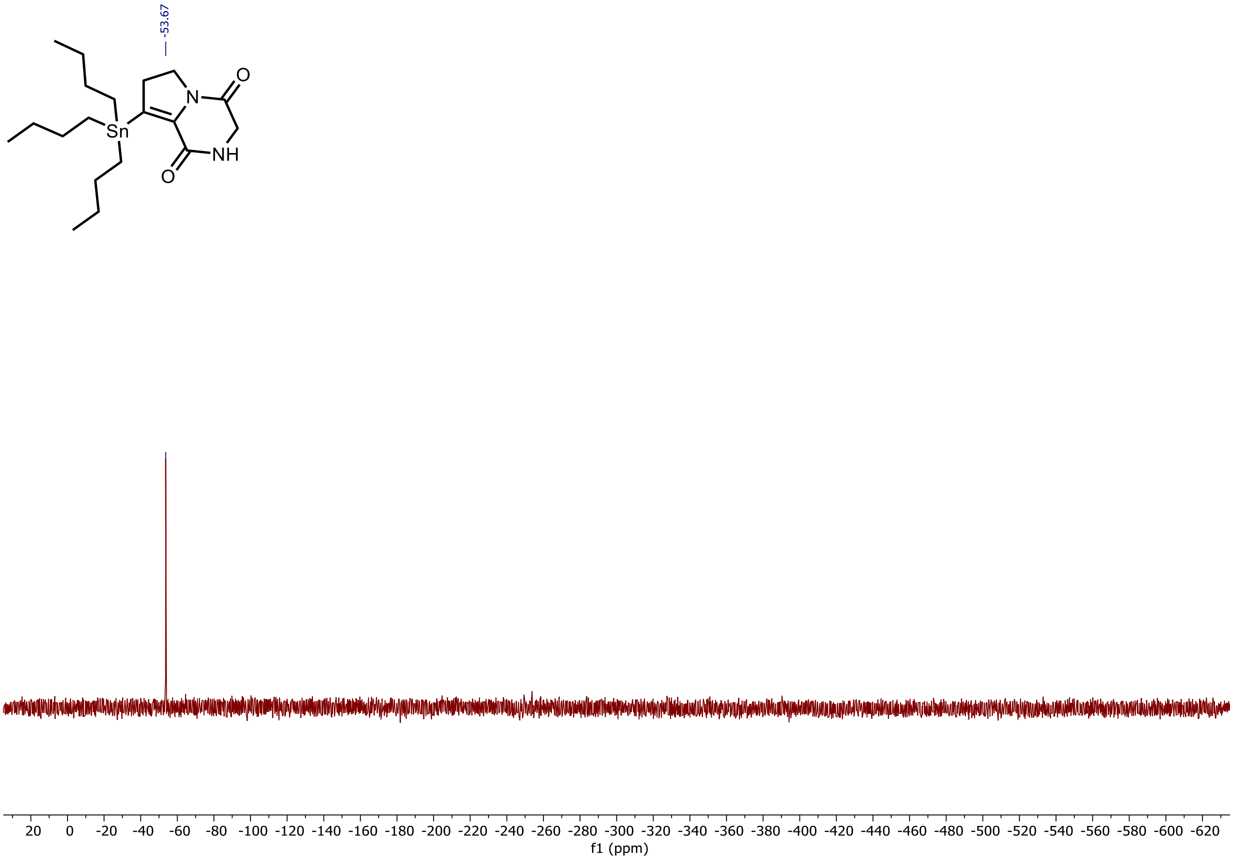

2.153. ¹H NMR Spectrum for Organotin 15 (500 MHz, $CDCl_3$)

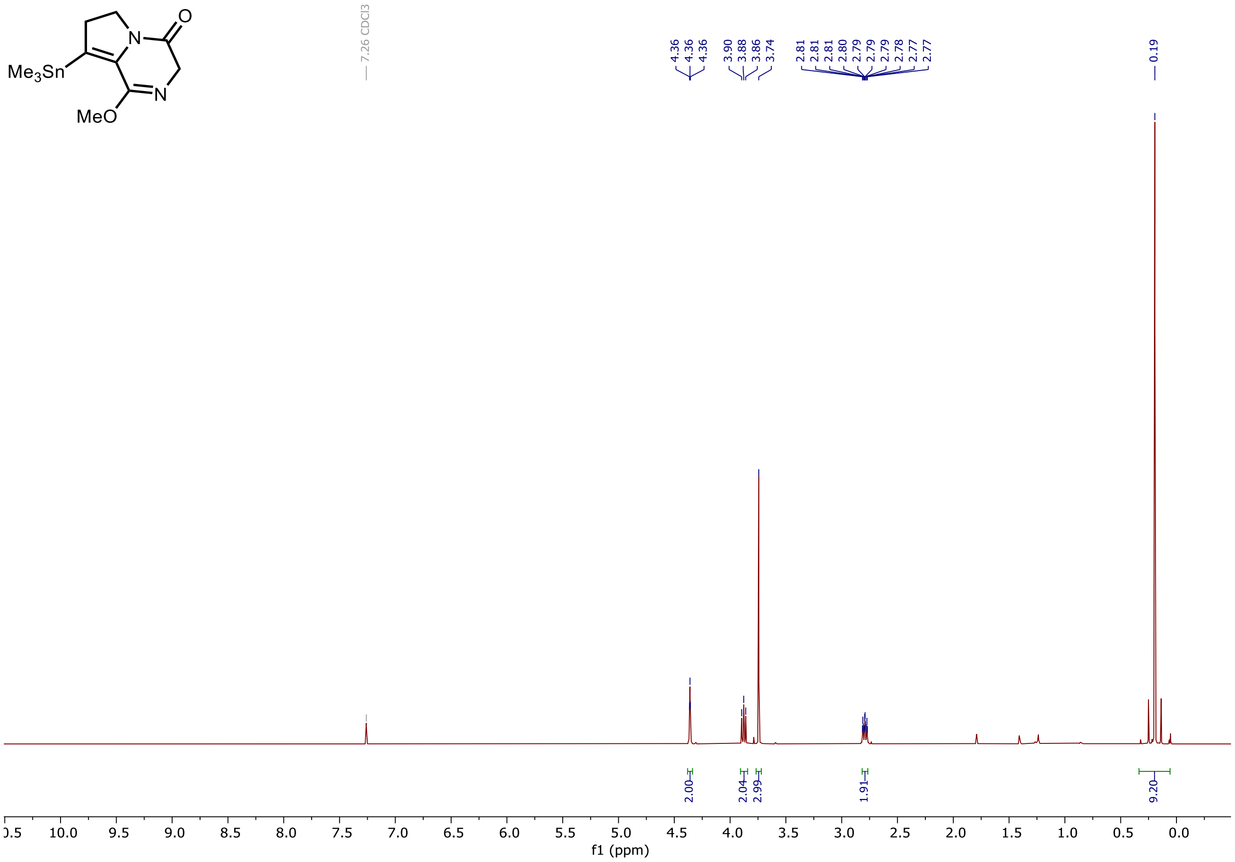

2.154. ¹³C NMR Spectrum for Organotin 15 (126 MHz, $CDCl_3$)

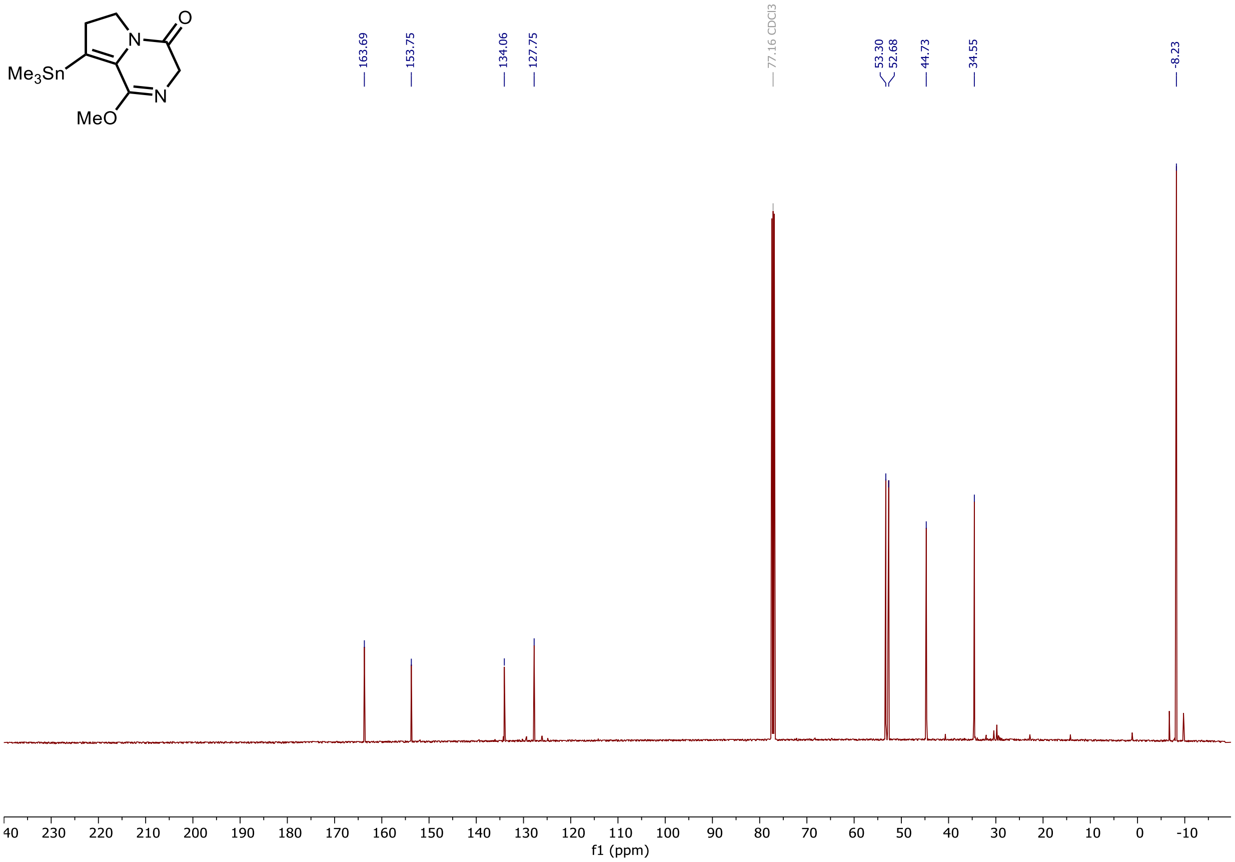

2.155. ^{1}H-^{1}H COSY Spectrum for Organotin 15 ($CDCl_3$)

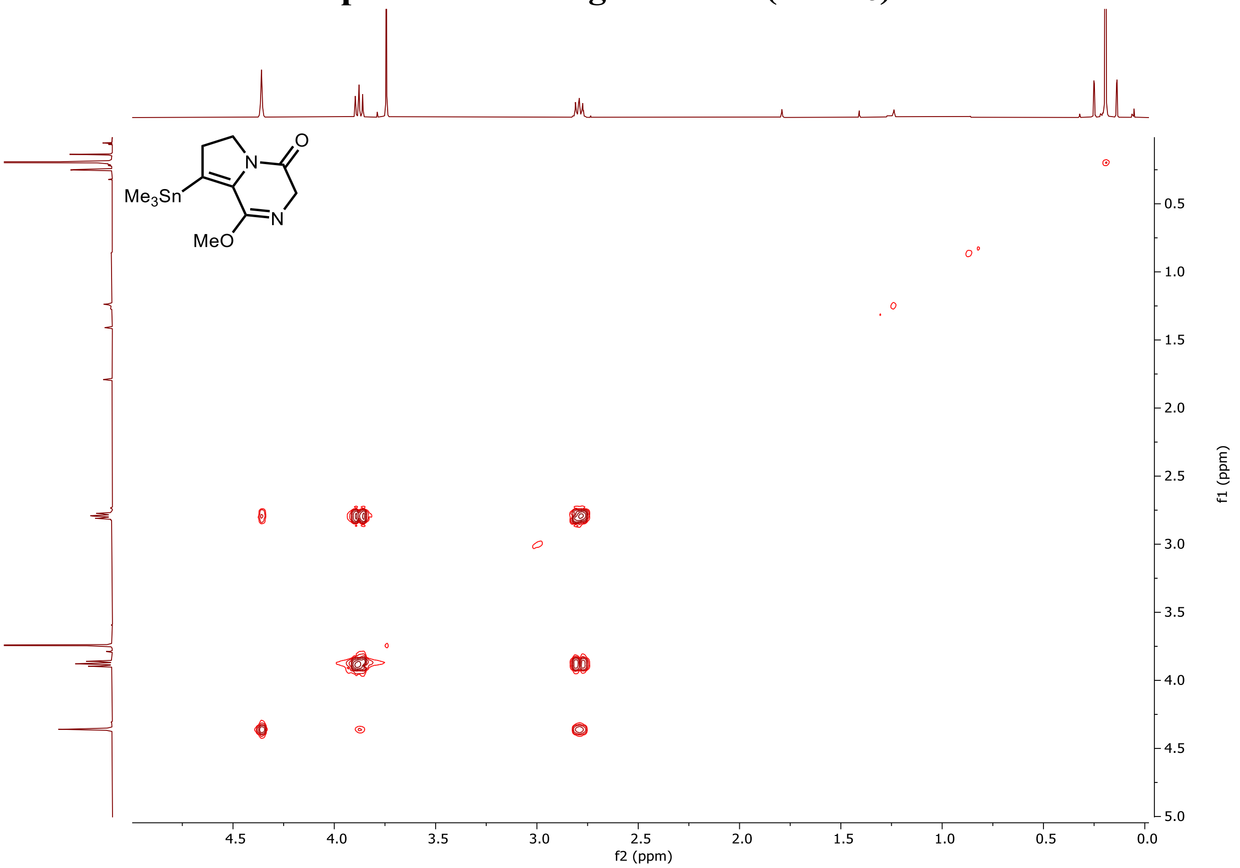

2.156. ^{1}H-^{13}C HSQC Spectrum for Organotin 15 ($CDCl_3$)

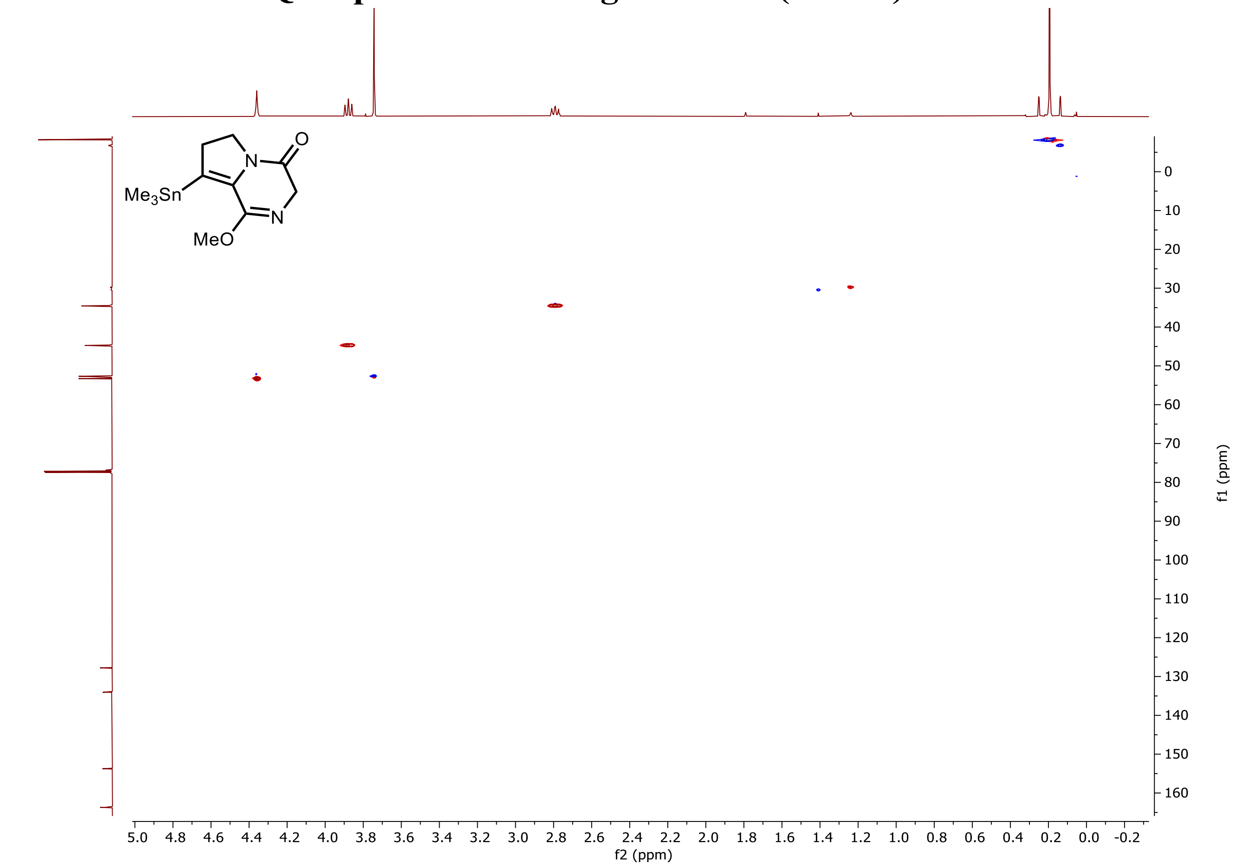

2.157. ^{1}H-^{13}C HMBC Spectrum for Organotin 15 ($CDCl_3$)

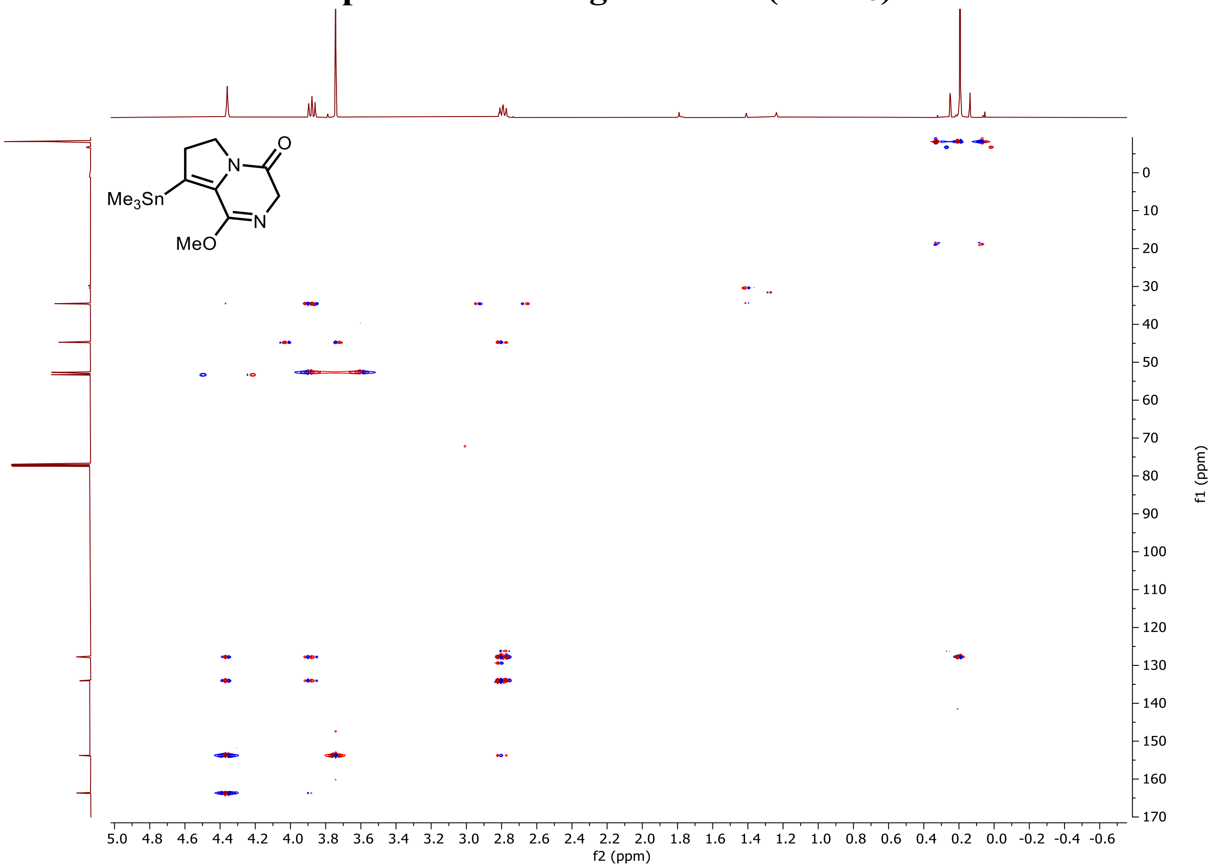

2.158. ^{119}Sn NMR Spectrum for Organotin 15 (149 MHz, $CDCl_3$)

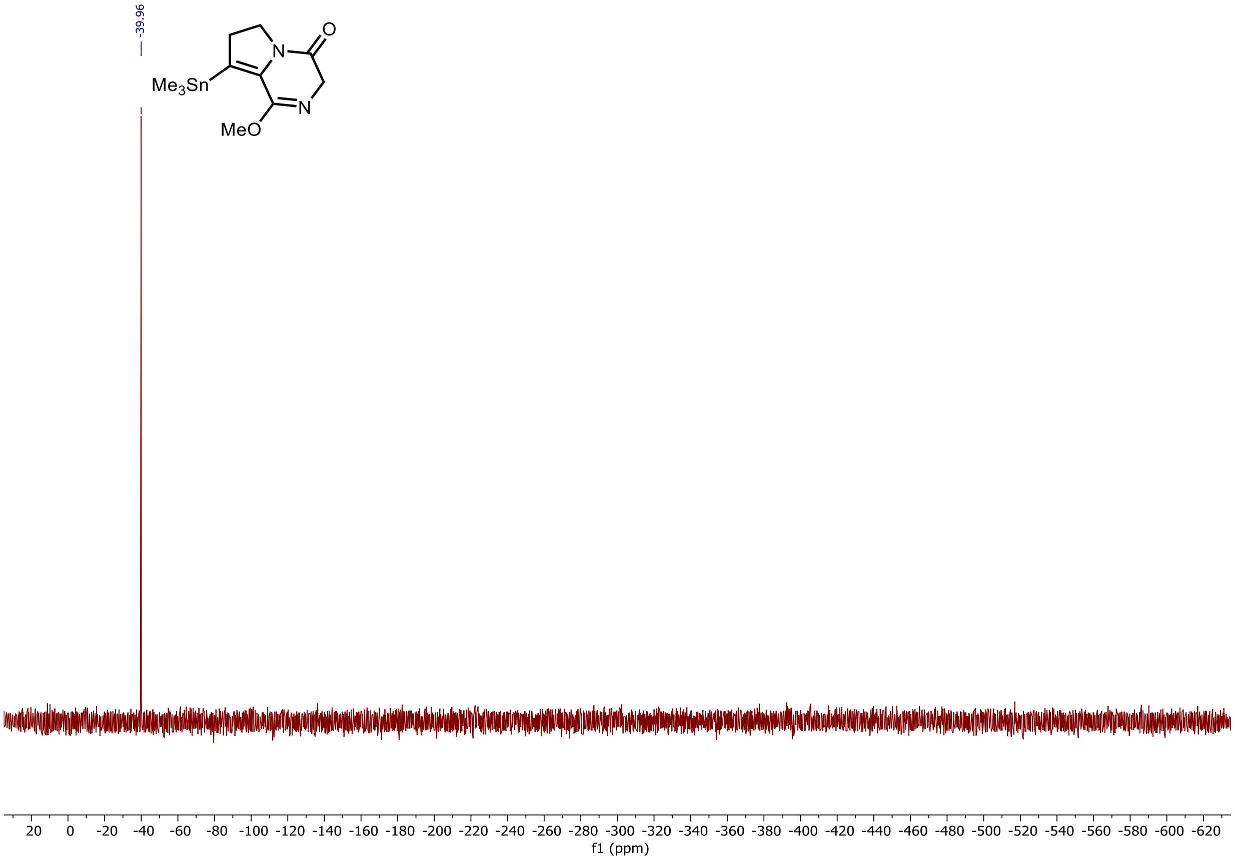

2.159. ^{1}H NMR Spectrum for Mono-Adduct 21 (500 MHz, CD_3OD)

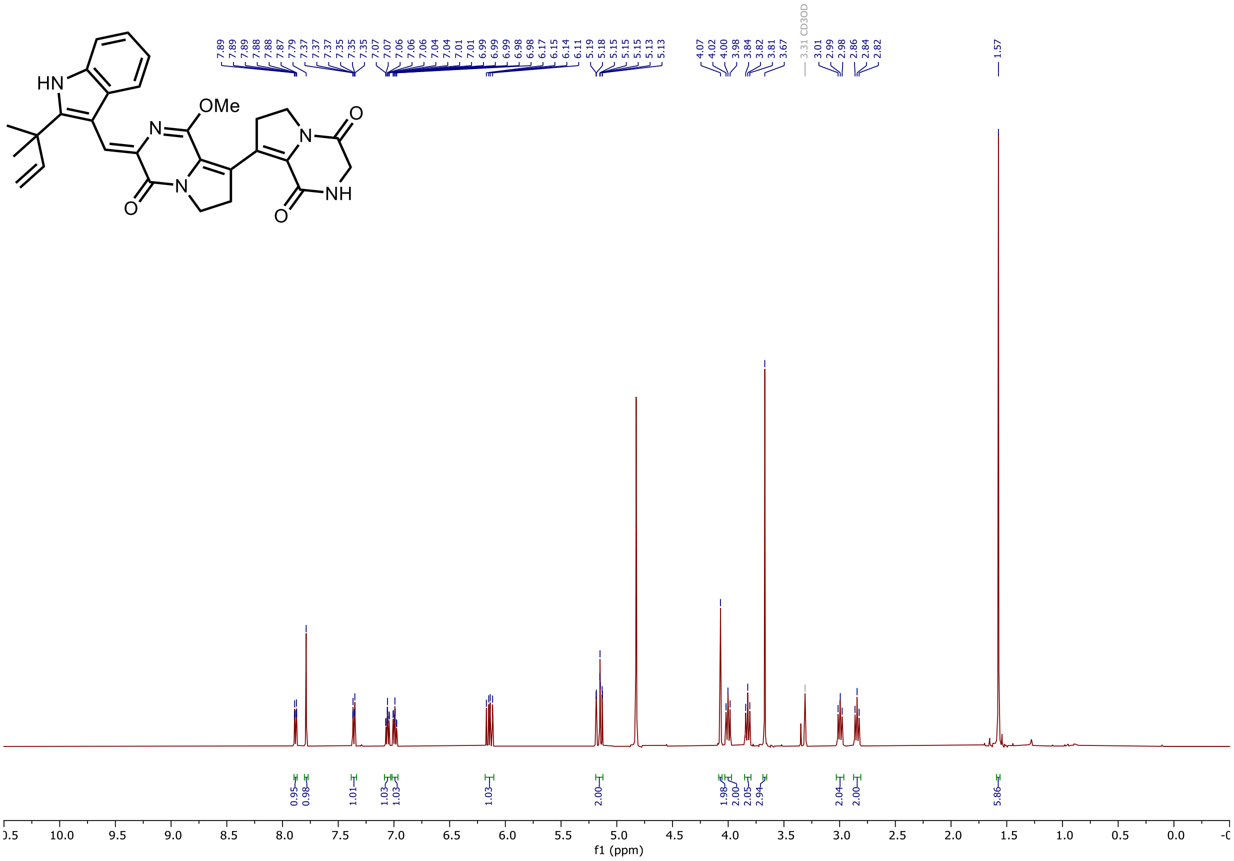

2.160. ^{13}C NMR Spectrum for Mono-Adduct 21 (126 MHz, CD_3OD)

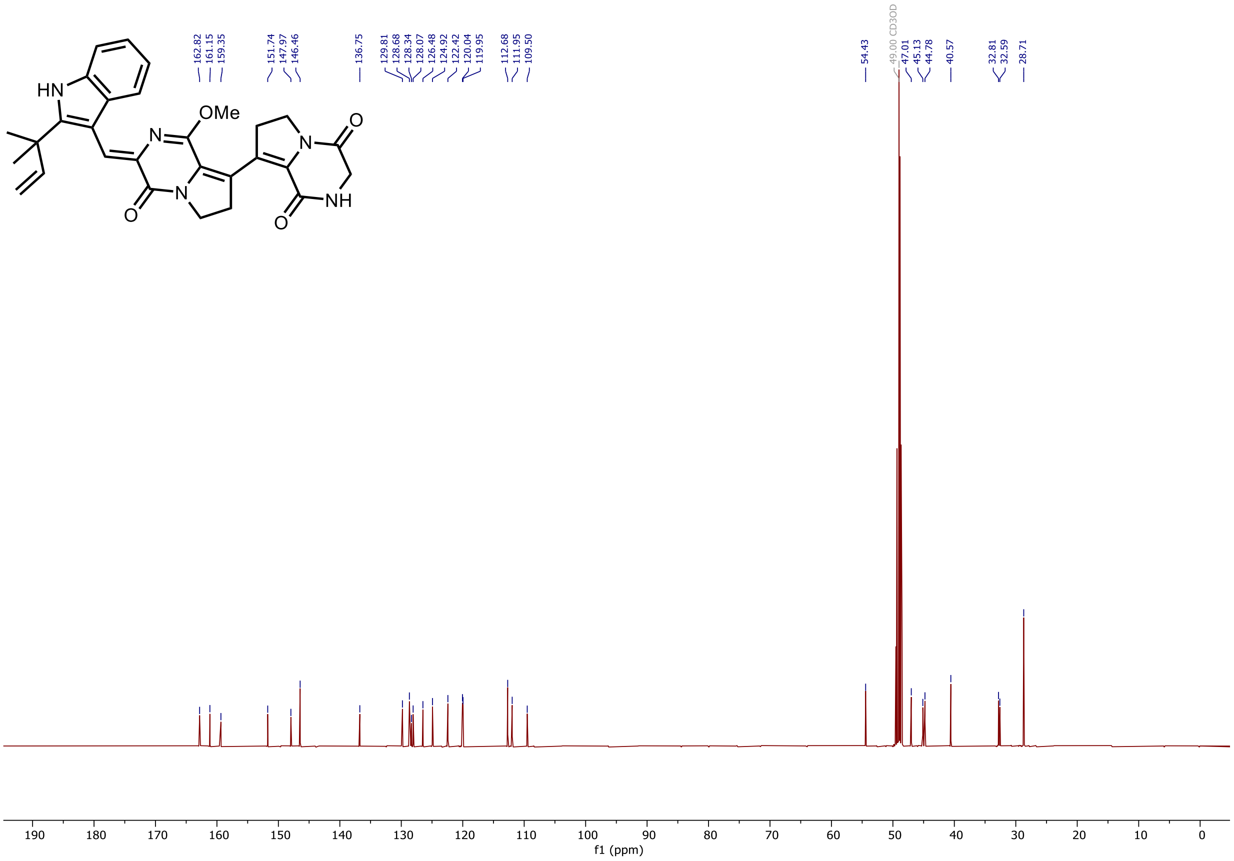

2.161. ^{1}H-^{1}H COSY Spectrum for Mono-Adduct 21 (CD_3OD)

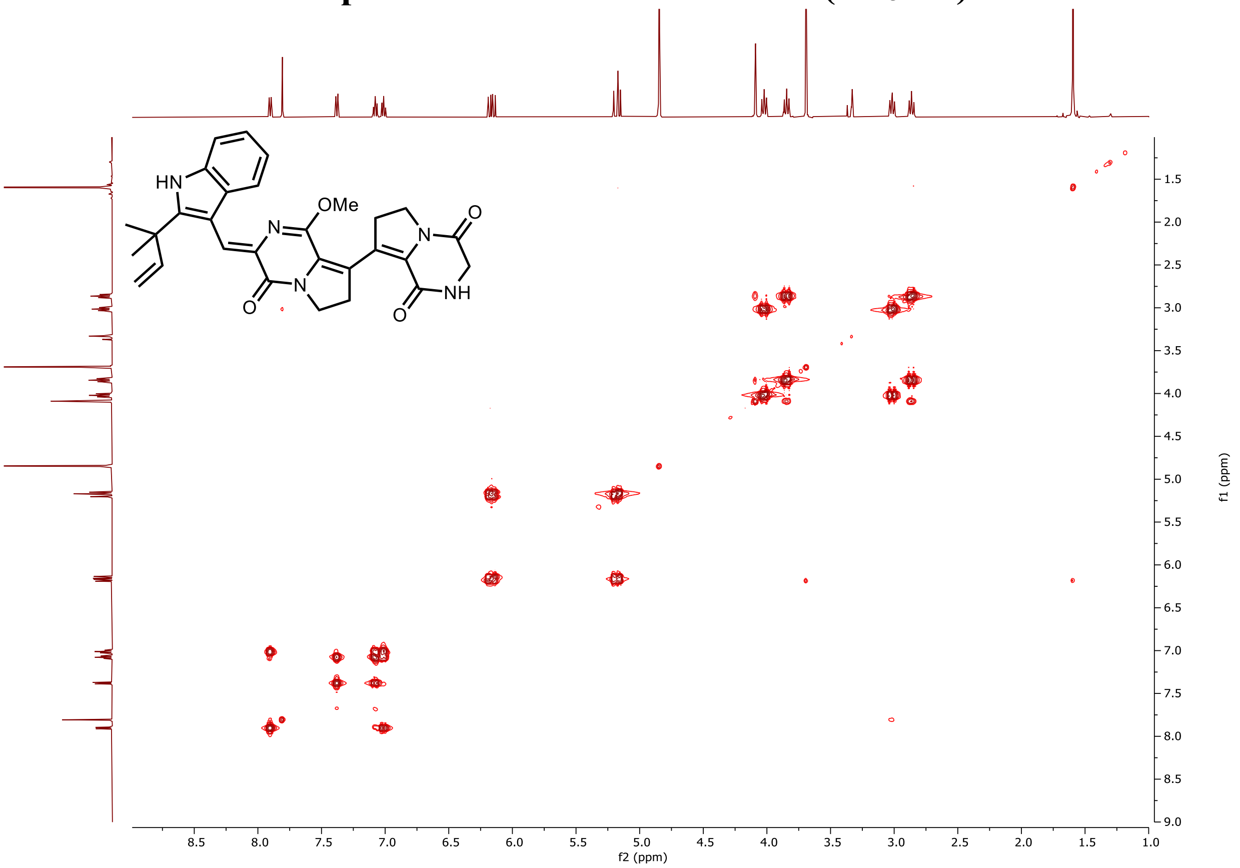

2.162. ^{1}H-^{13}C HSQC Spectrum for Mono-Adduct 21 (CD_3OD)

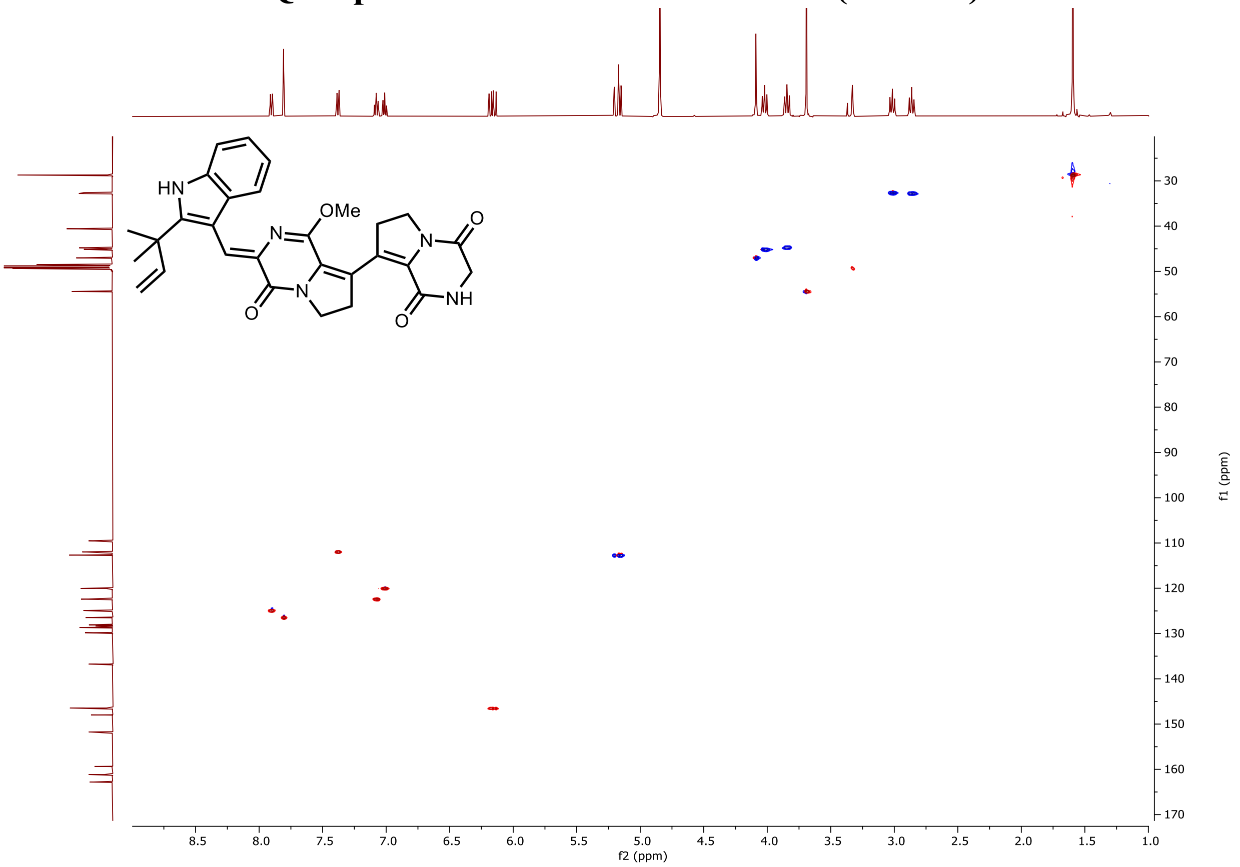

2.163. ^{1}H-^{13}C HMBC Spectrum for Mono-Adduct 21 (CD_3OD)

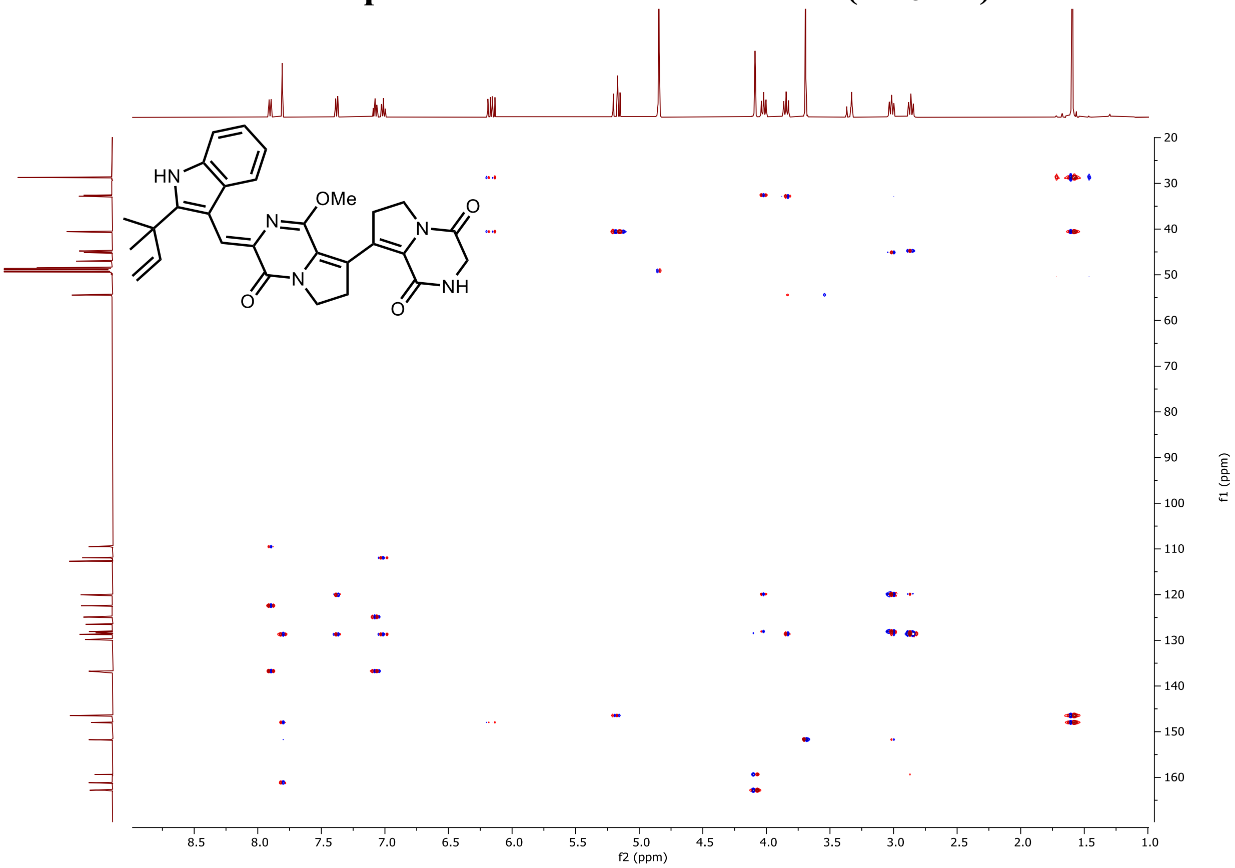

2.164. ^{1}H-^{1}H NOESY Spectrum for Mono-Adduct 21 (CD_3OD)

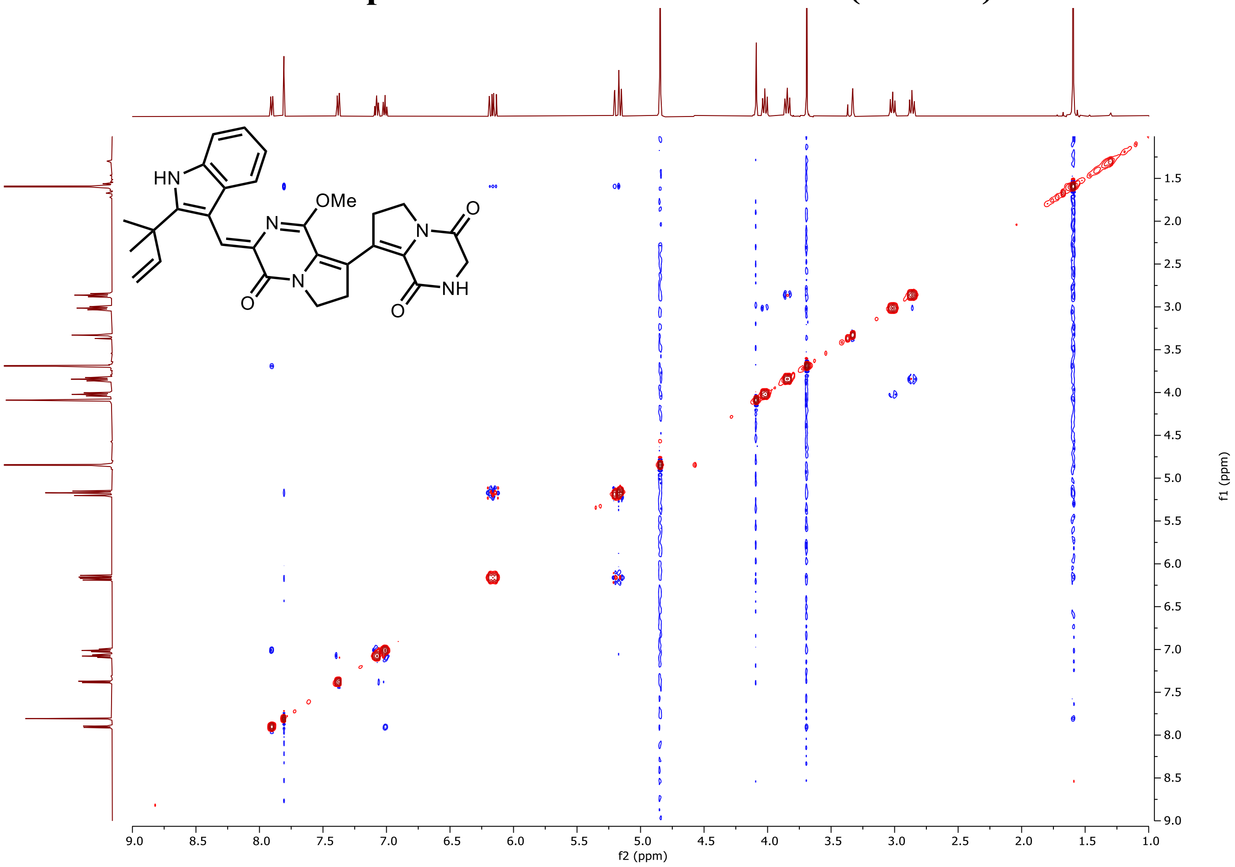

3. IR Spectra

3.1. IR Spectrum for phthalylglycyl chloride

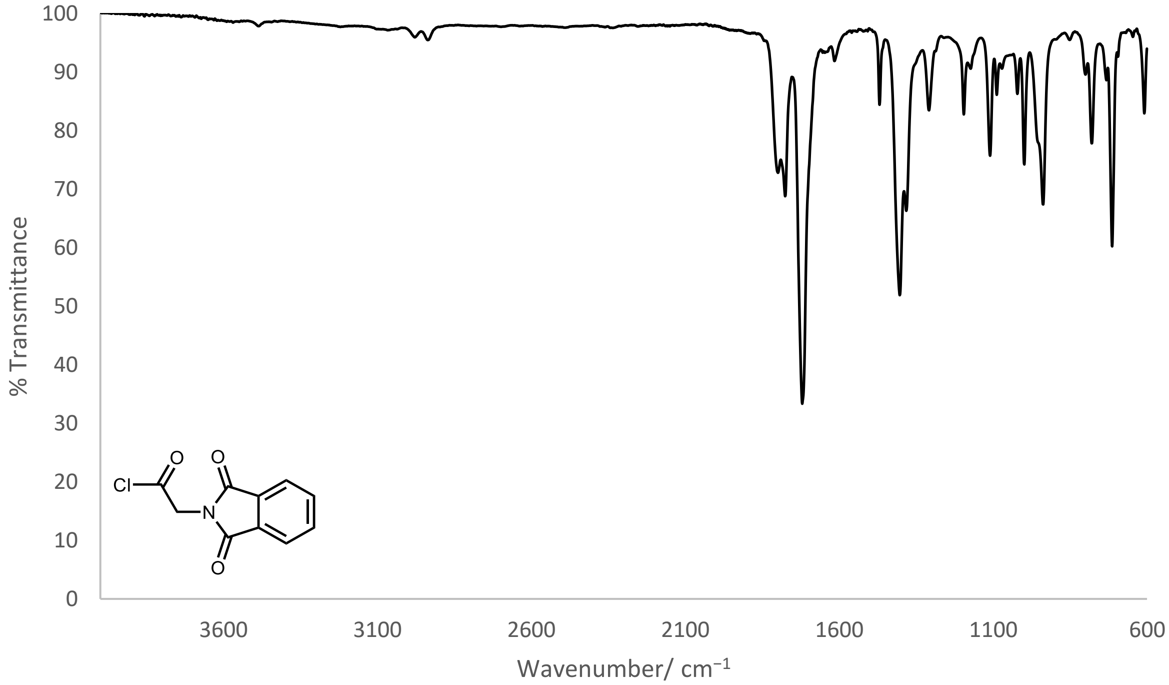

3.2. IR Spectrum for Enamide 8

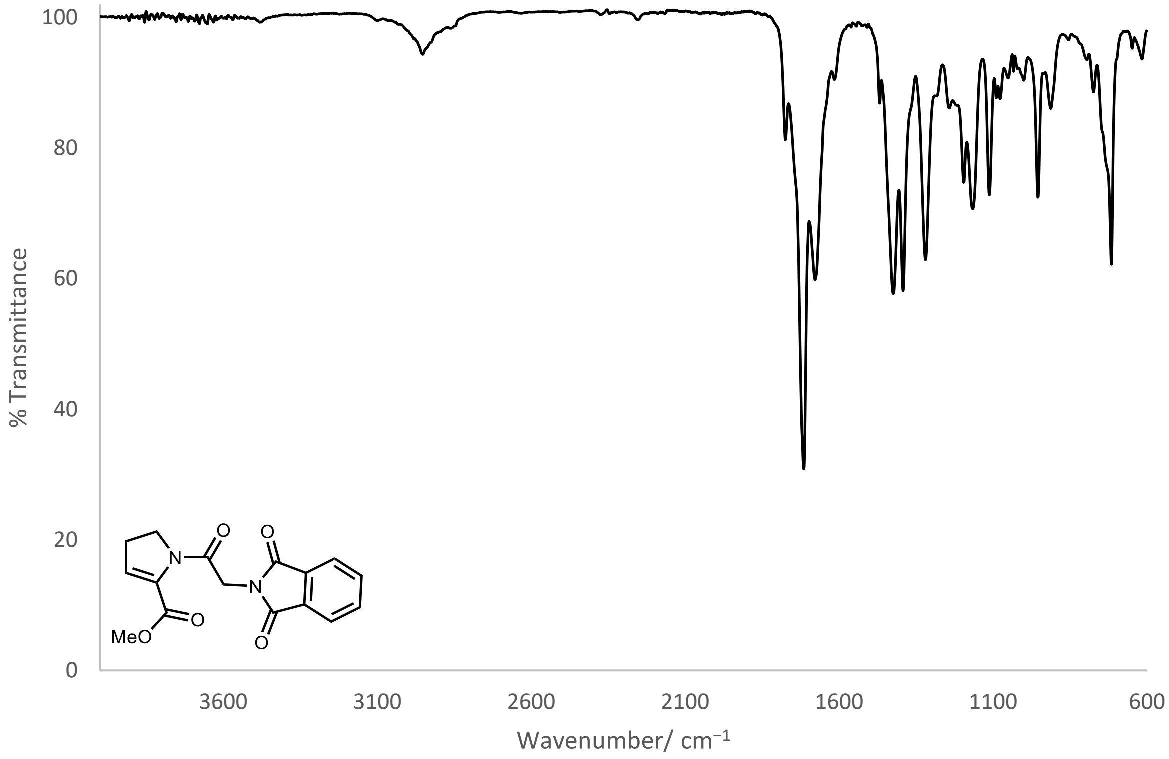

3.3. IR Spectrum for Diketopiperazine 10

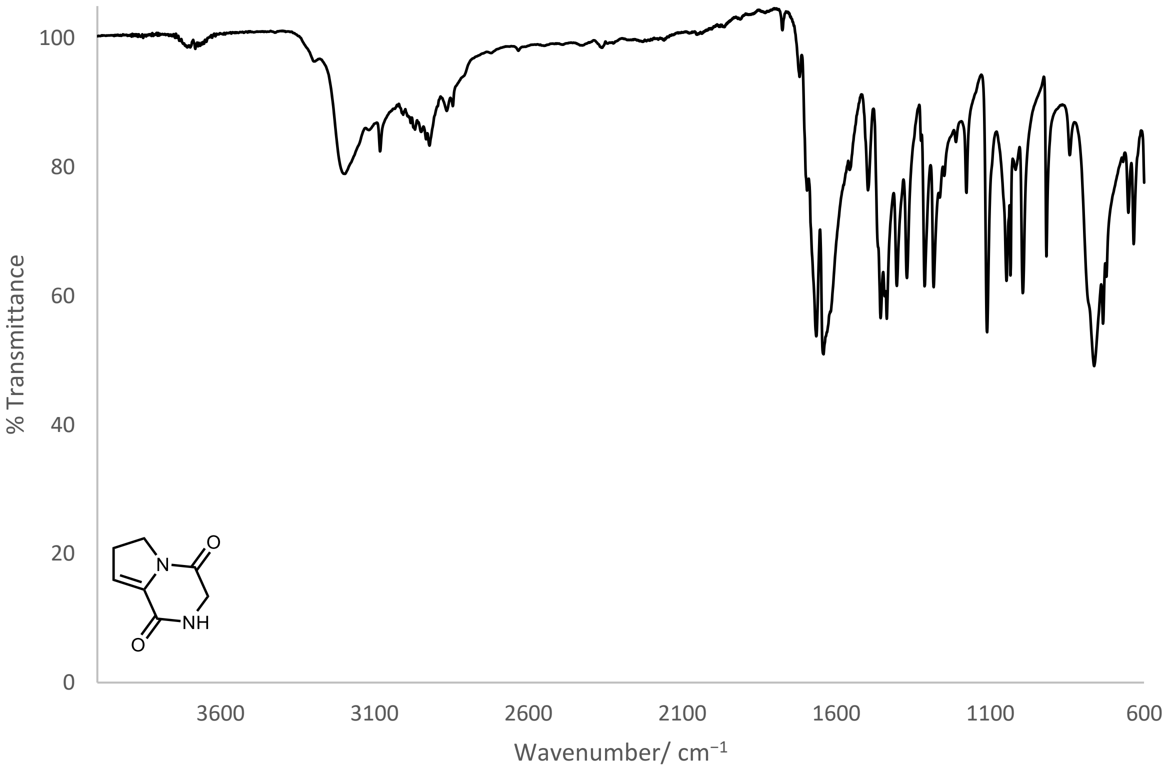

3.4. IR Spectrum for Alkenyl Iodide 11

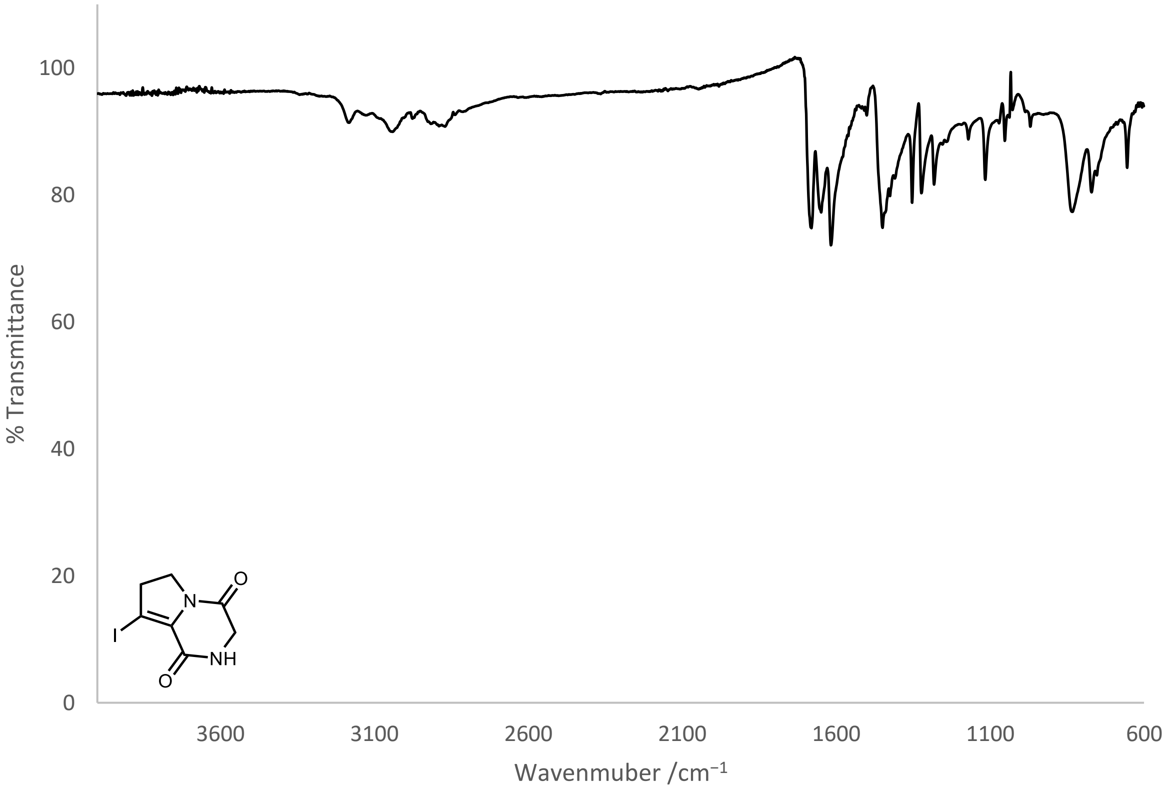

3.5. IR Spectrum for Lactim Ether 13

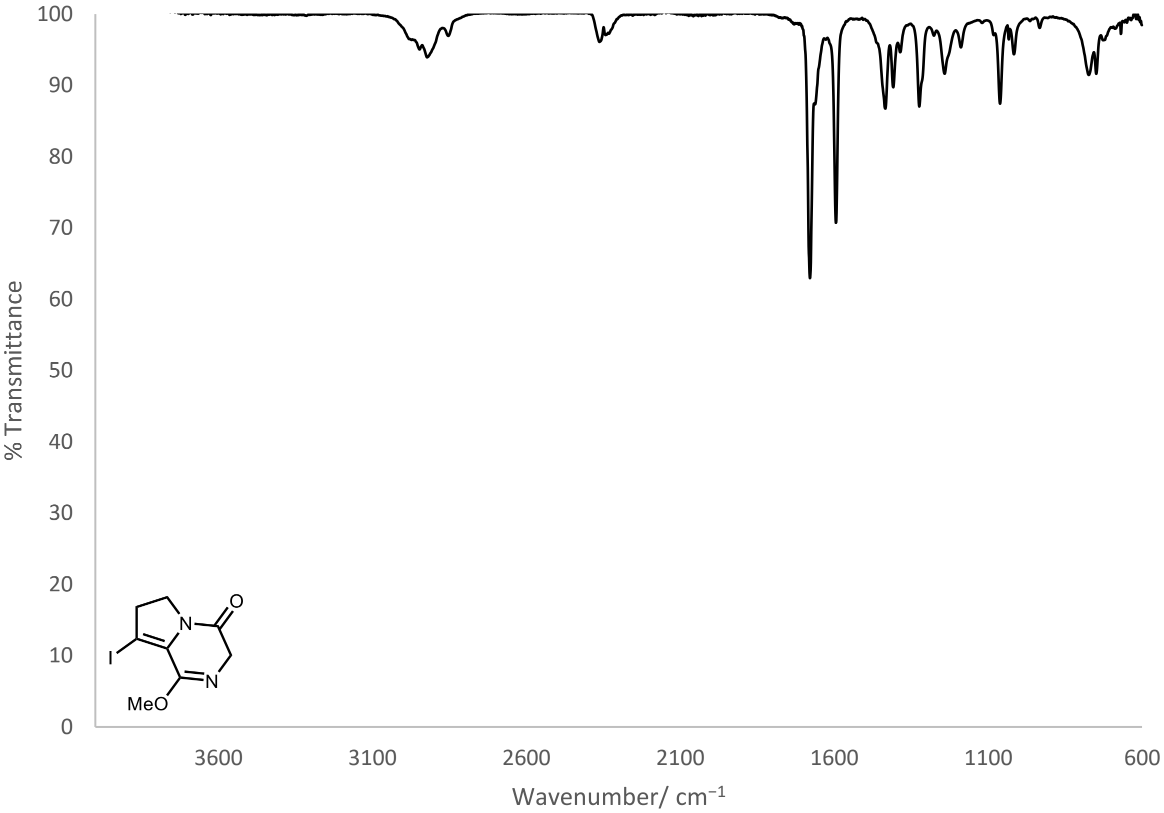

3.6. IR Spectrum for Organotin 12

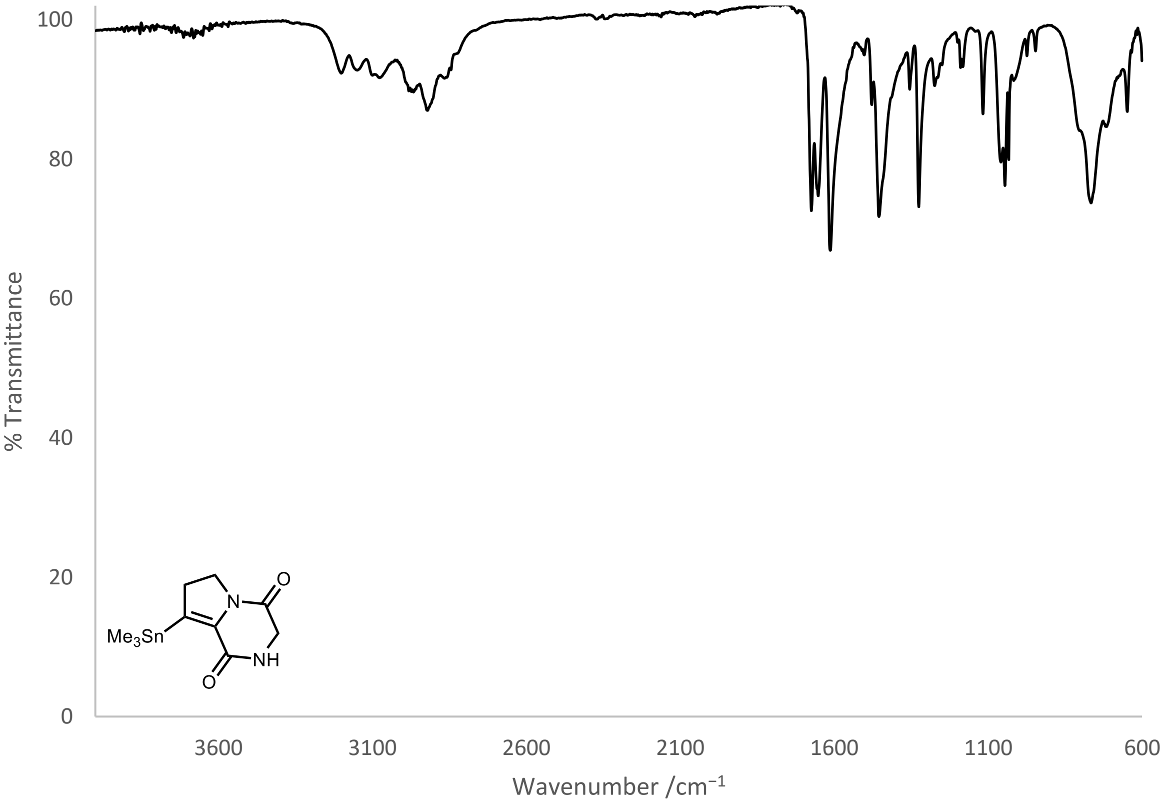

3.7. IR Spectrum for Bis-Diketopiperazine 14

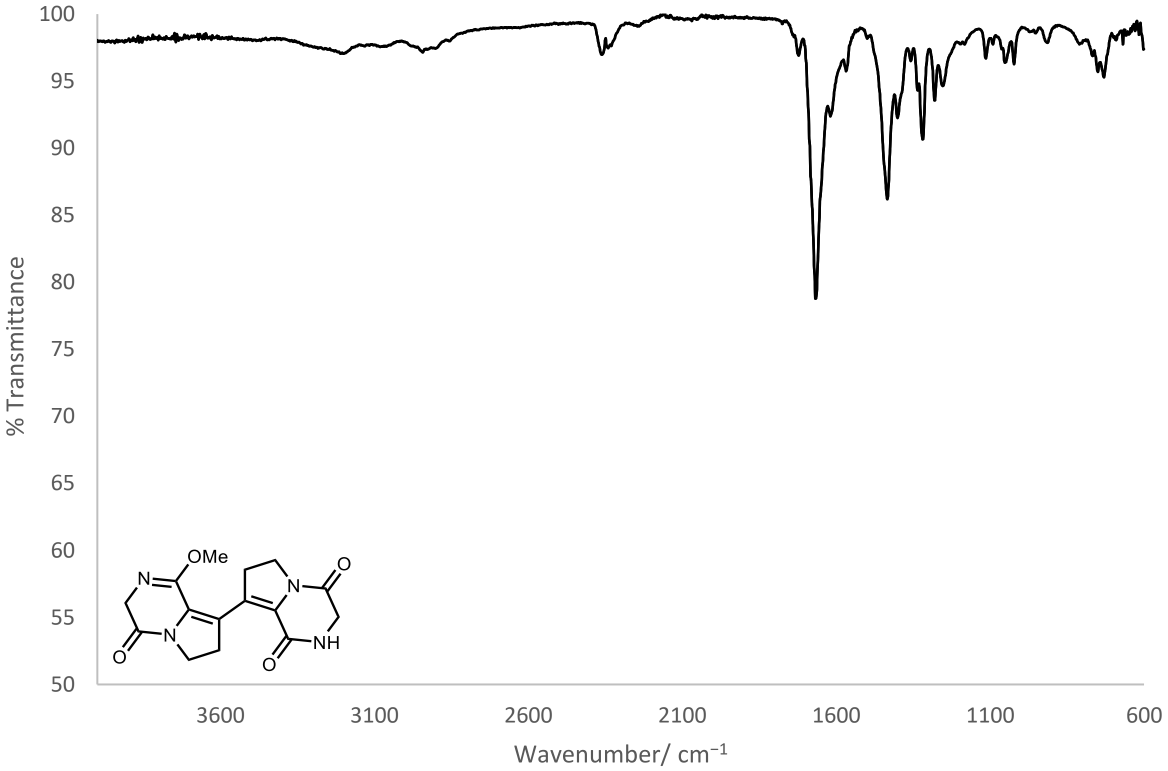

3.8. IR Spectrum for Bis-Lactim Ether 16

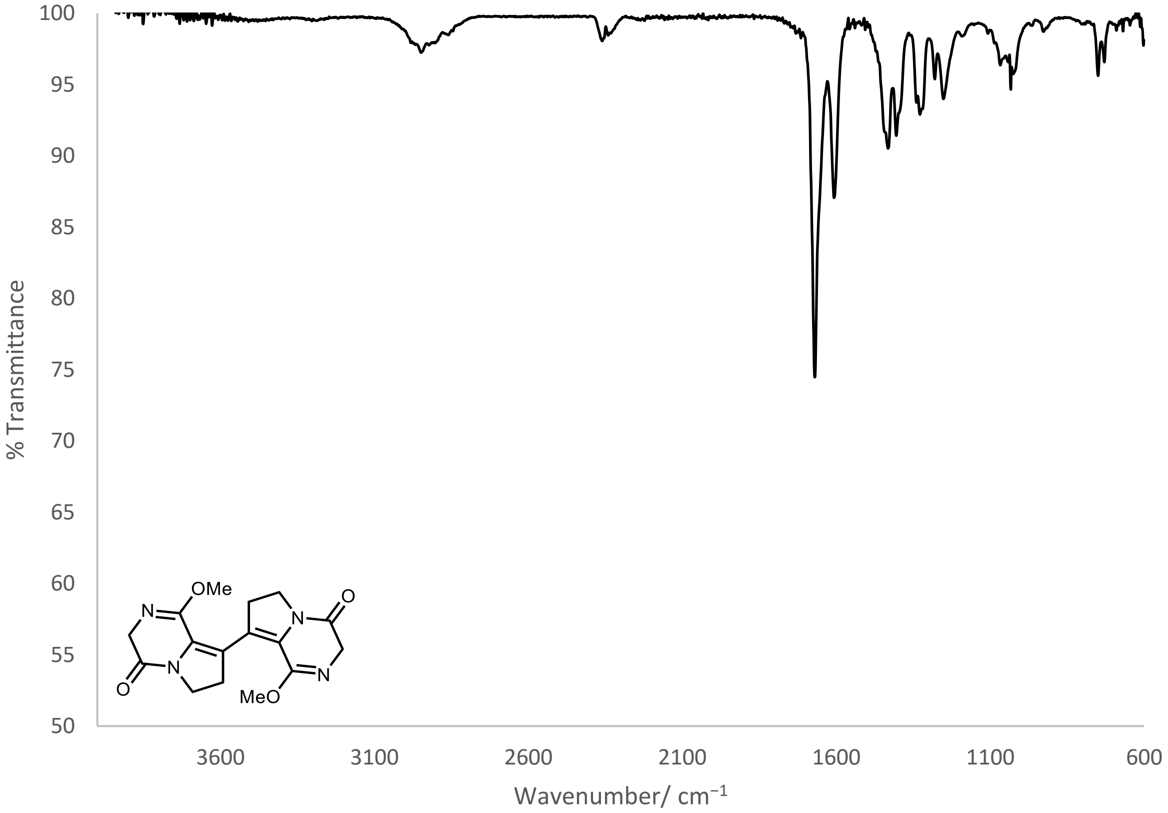

3.9. IR Spectrum for 3-chloroindole

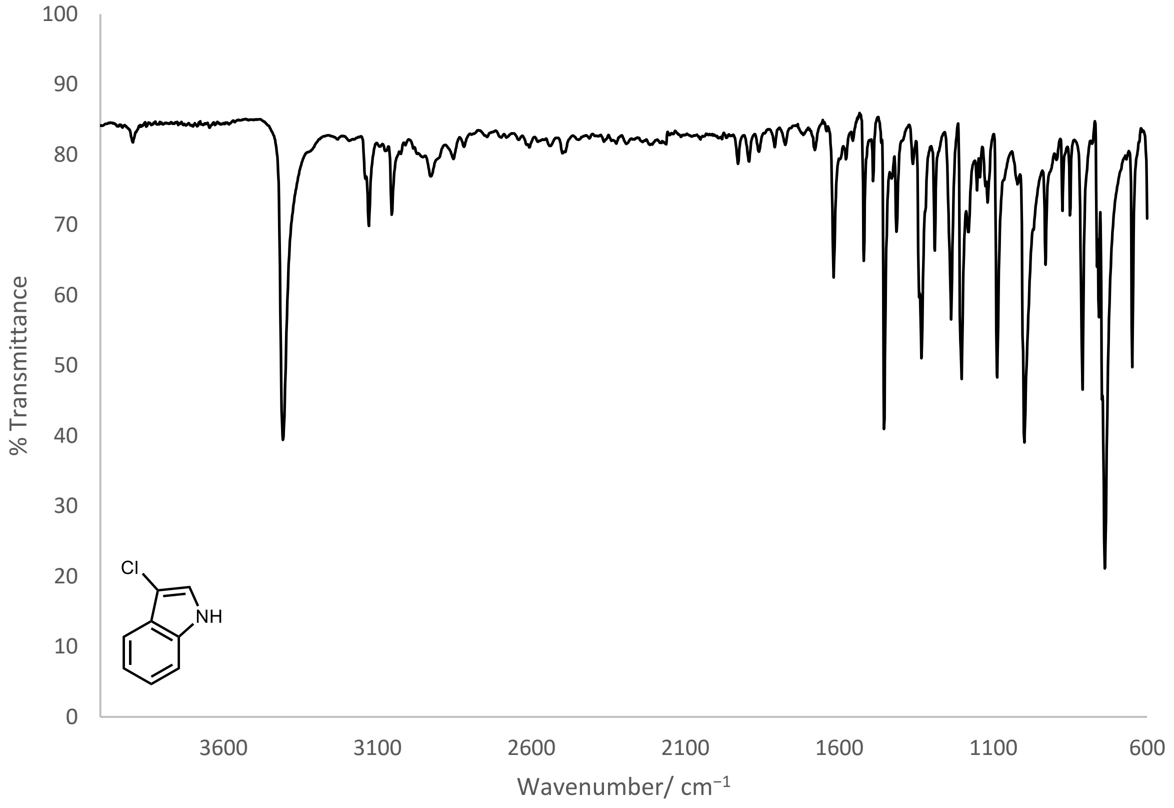

3.10. IR Spectrum for Indole S-1

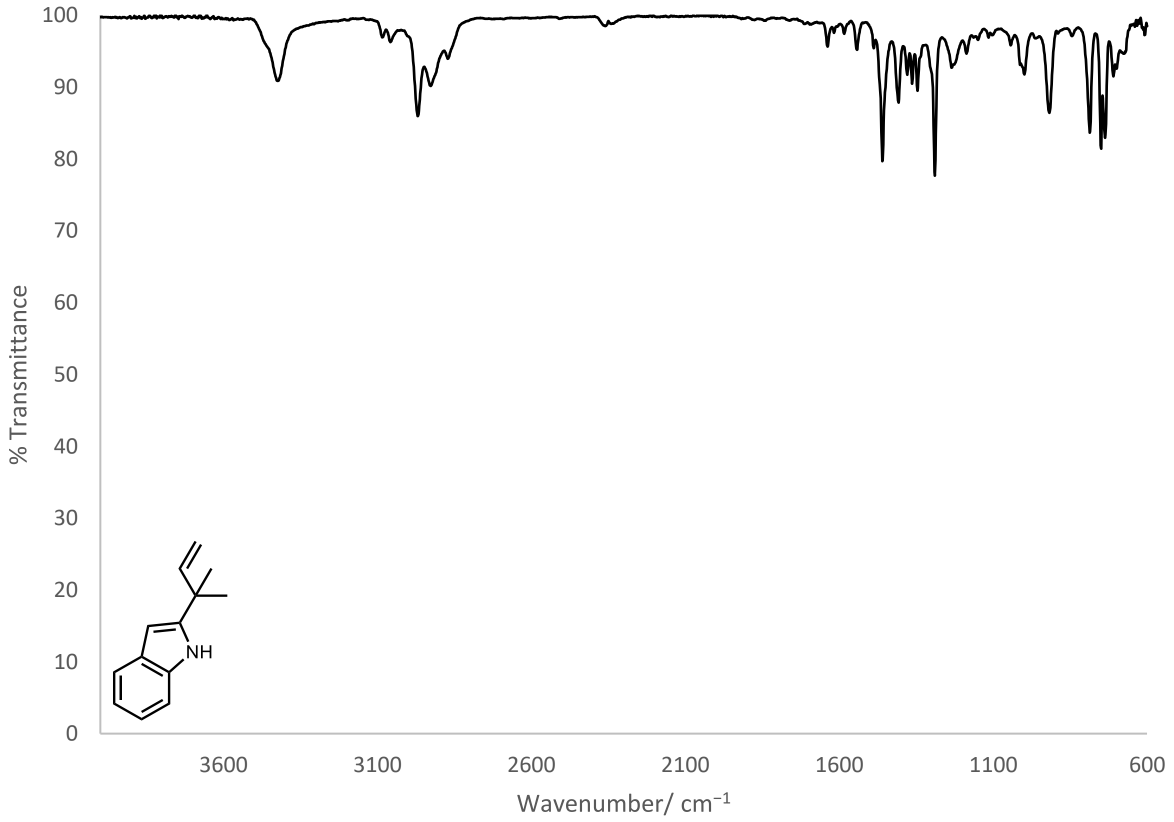

3.11. IR Spectrum for Aldehyde 3

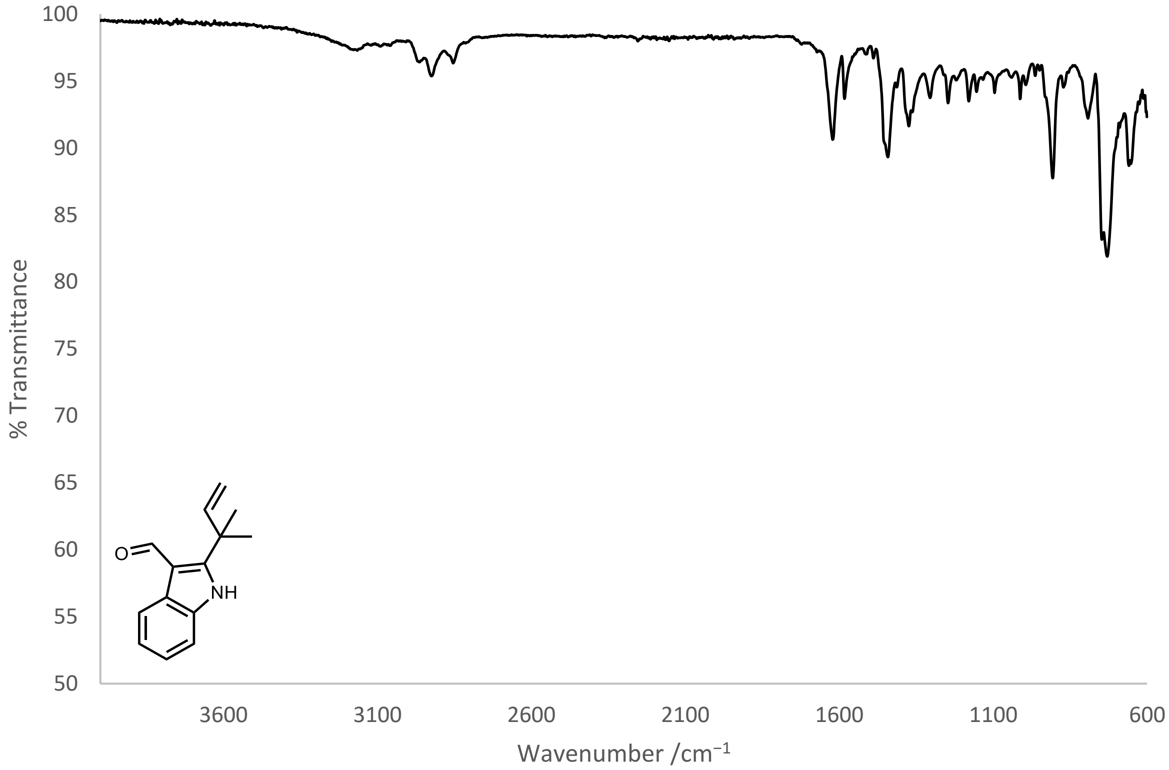

3.12. IR Spectrum for Enone 23

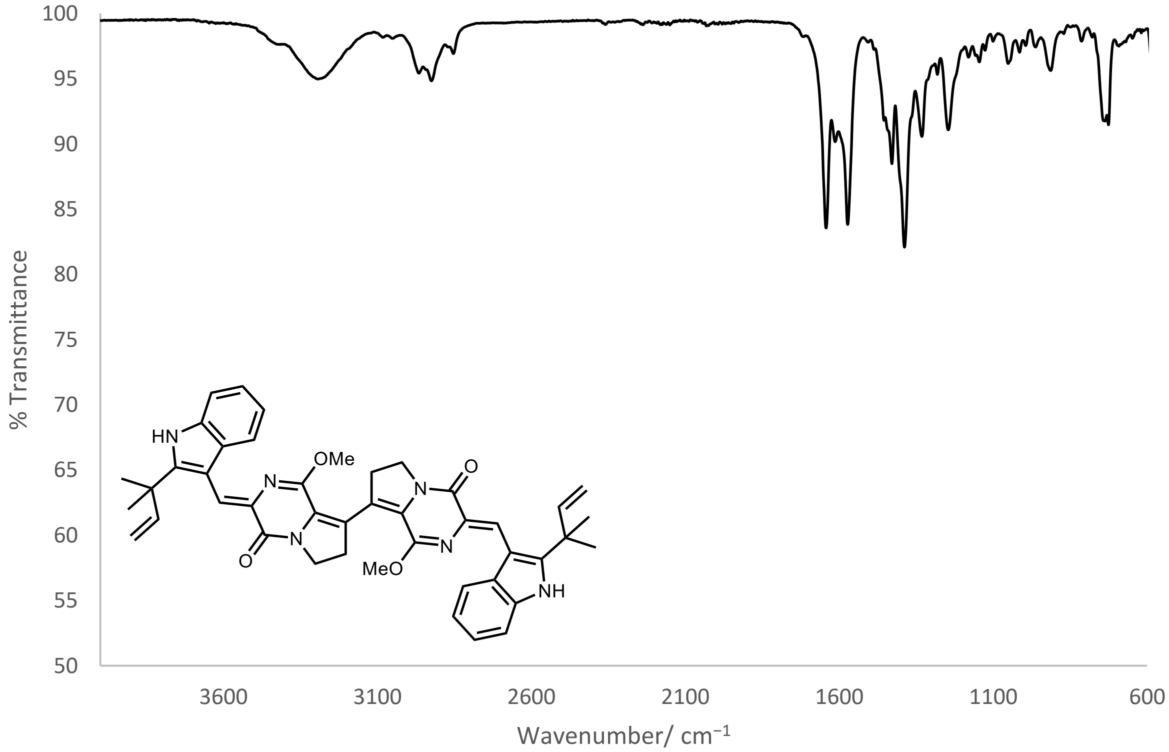

3.13. IR Spectrum for Enone 22

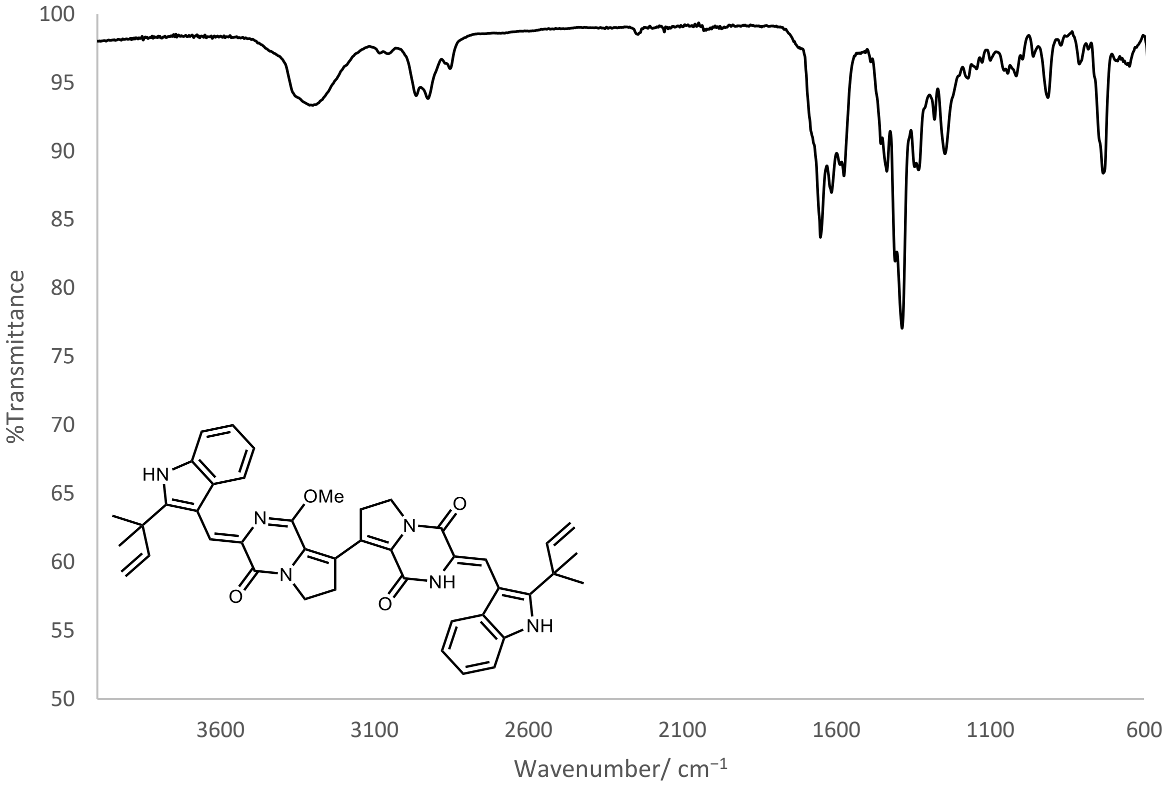

3.14. IR Spectrum for Brevianamide S (1)

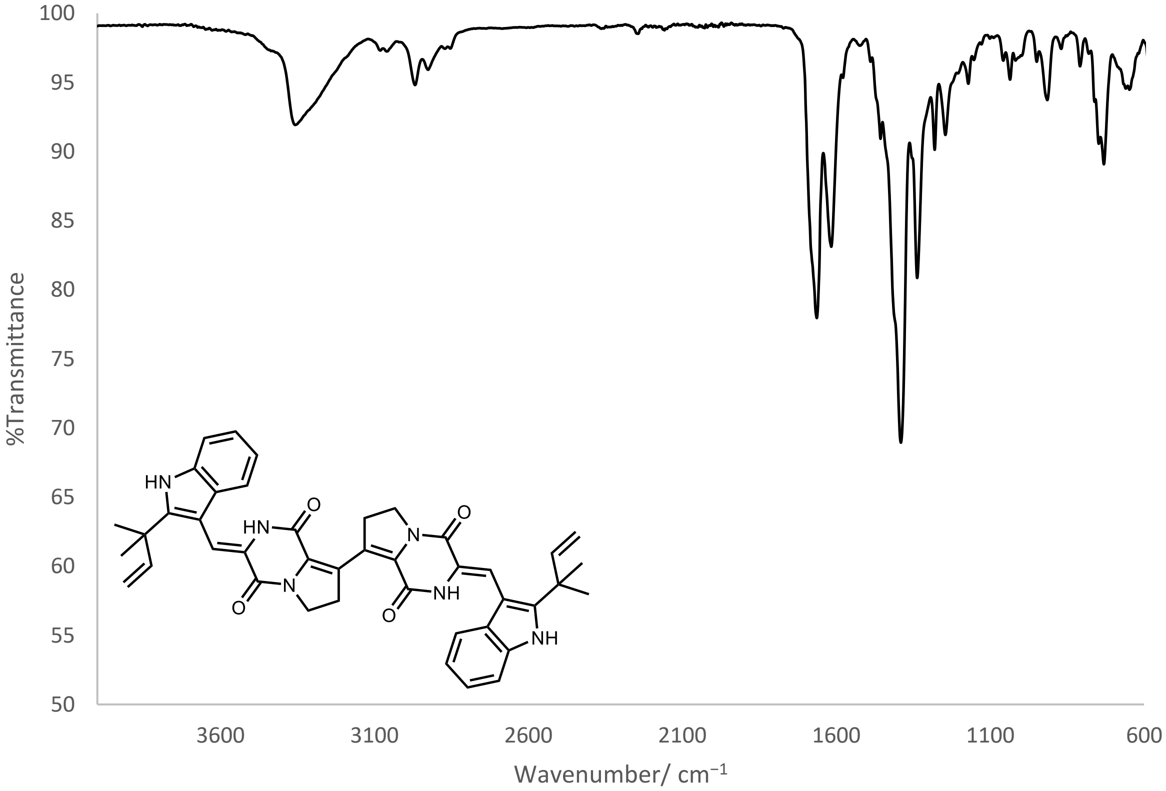

3.15. IR Spectrum for Dehydroproline 7

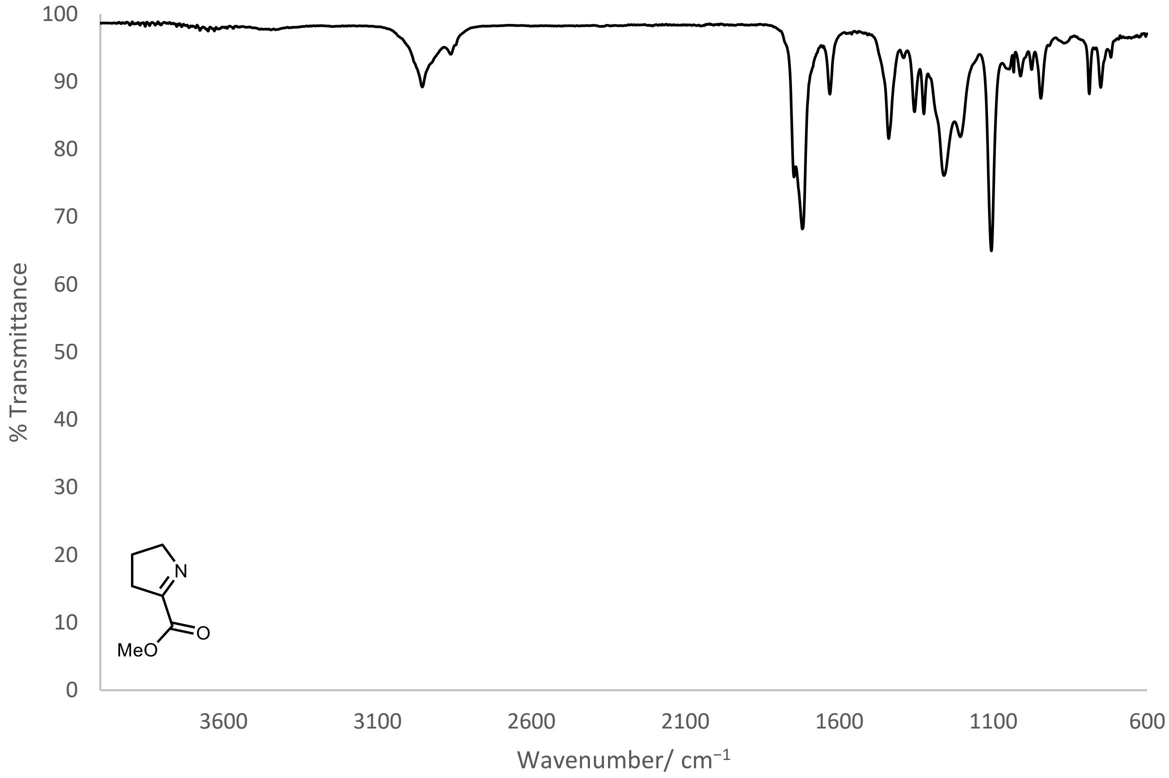

3.16. IR Spectrum for β-lactam (±)-9

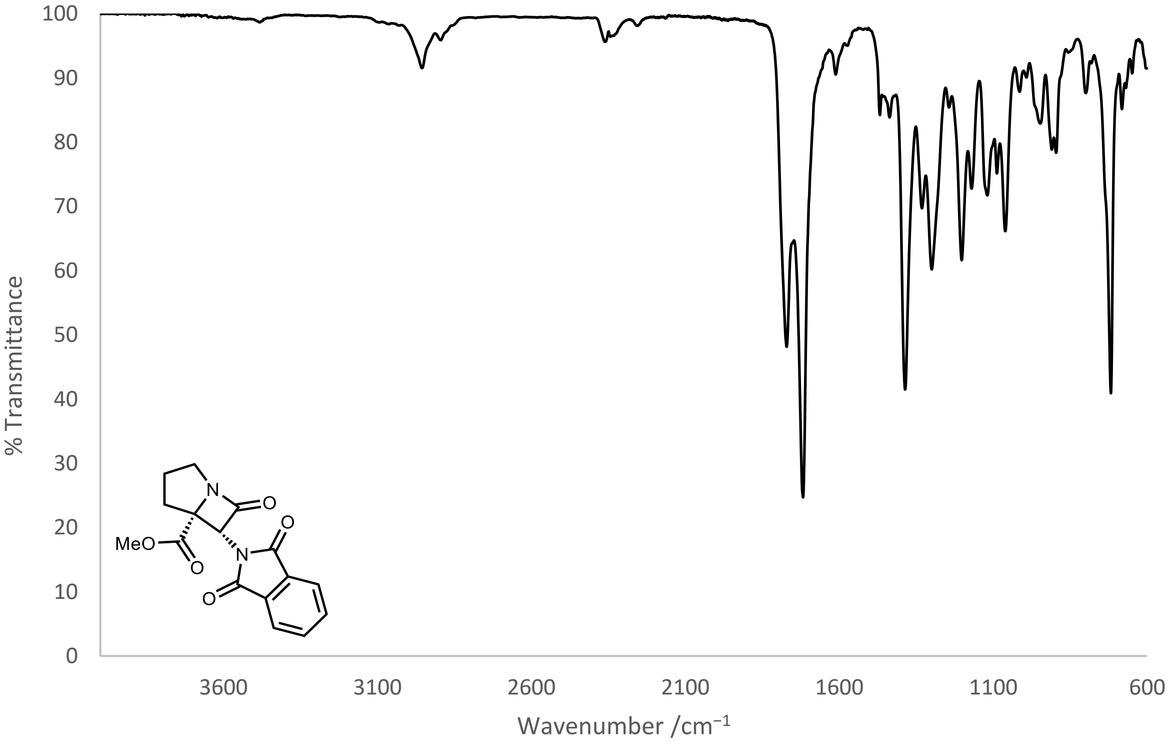

3.17. IR Spectrum for Iodide S-2

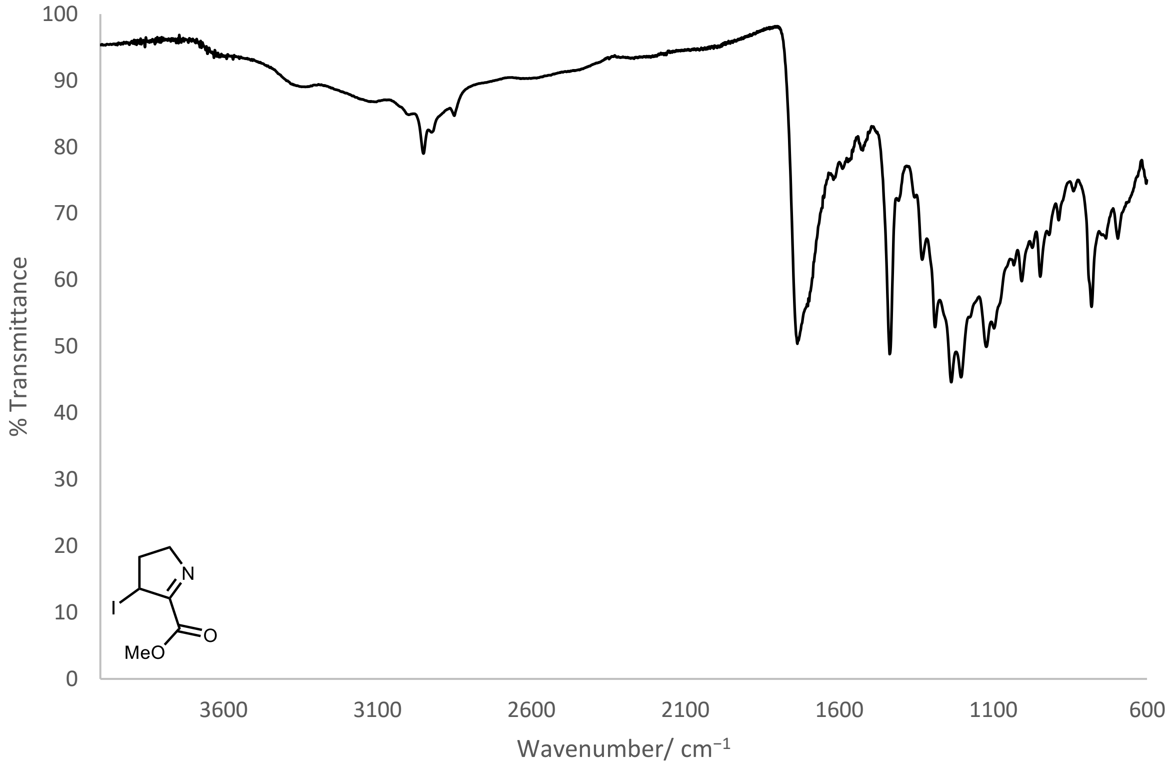

3.18. IR Spectrum for β-Lactam (±)-S-3

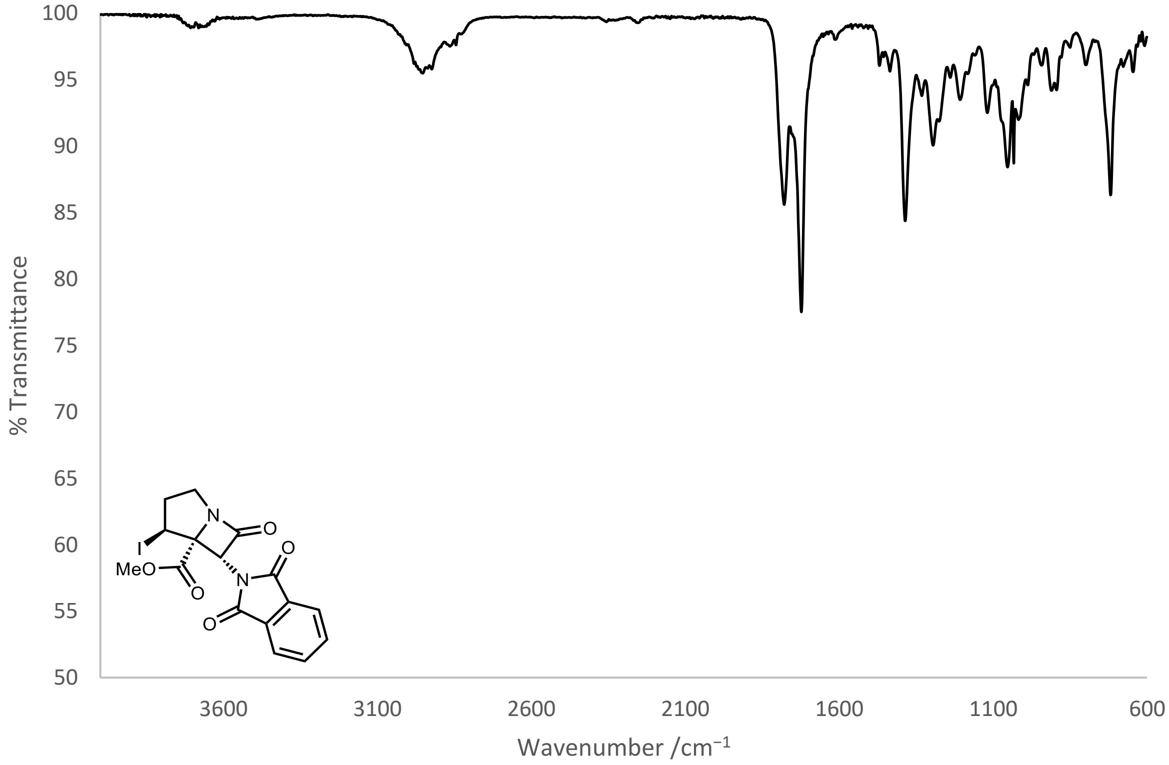

3.19. IR Spectrum for Hemiaminal S-4

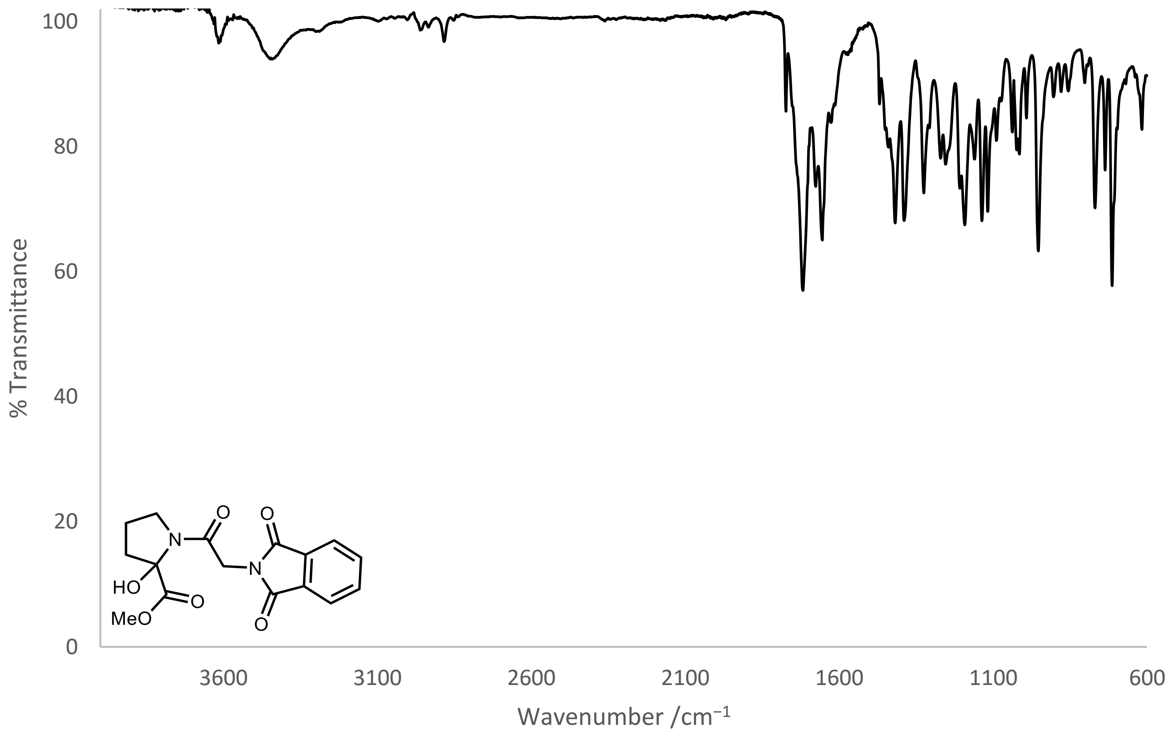

3.20. IR Spectrum for Lactim Ether S-5

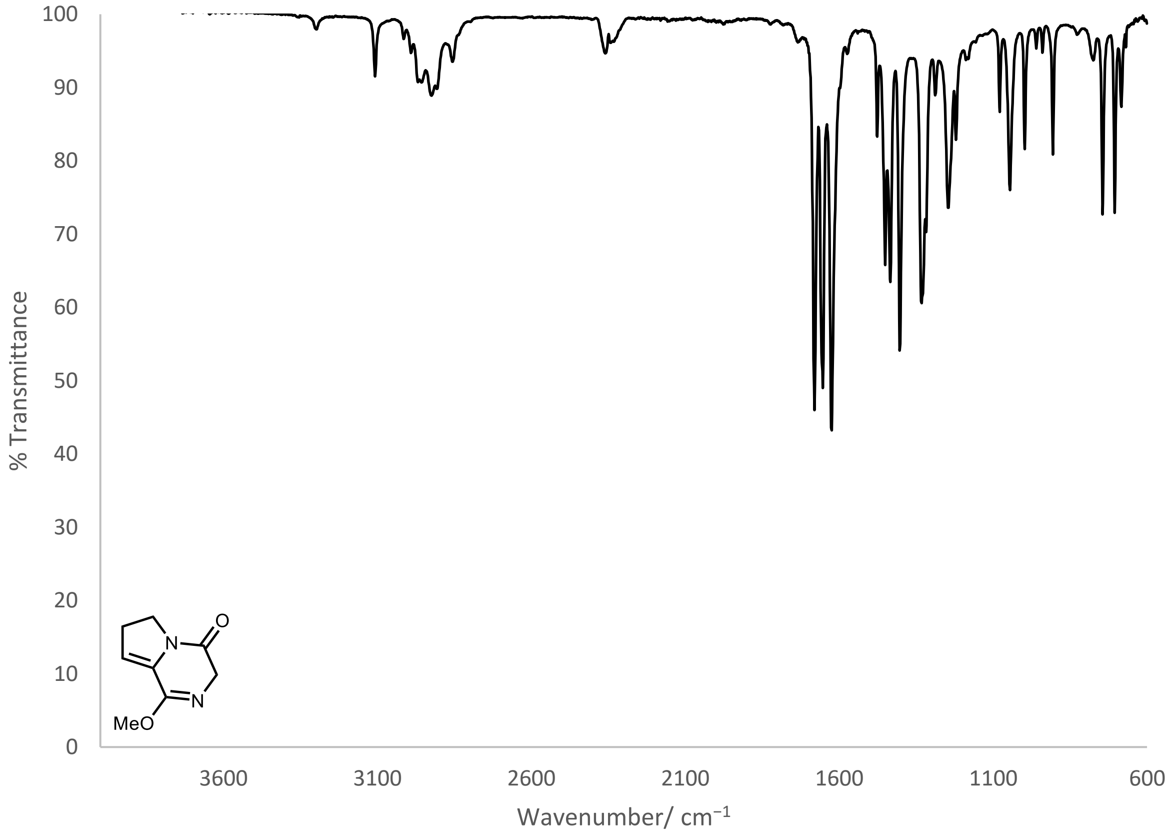

3.21. IR Spectrum for Pyrazinone 18

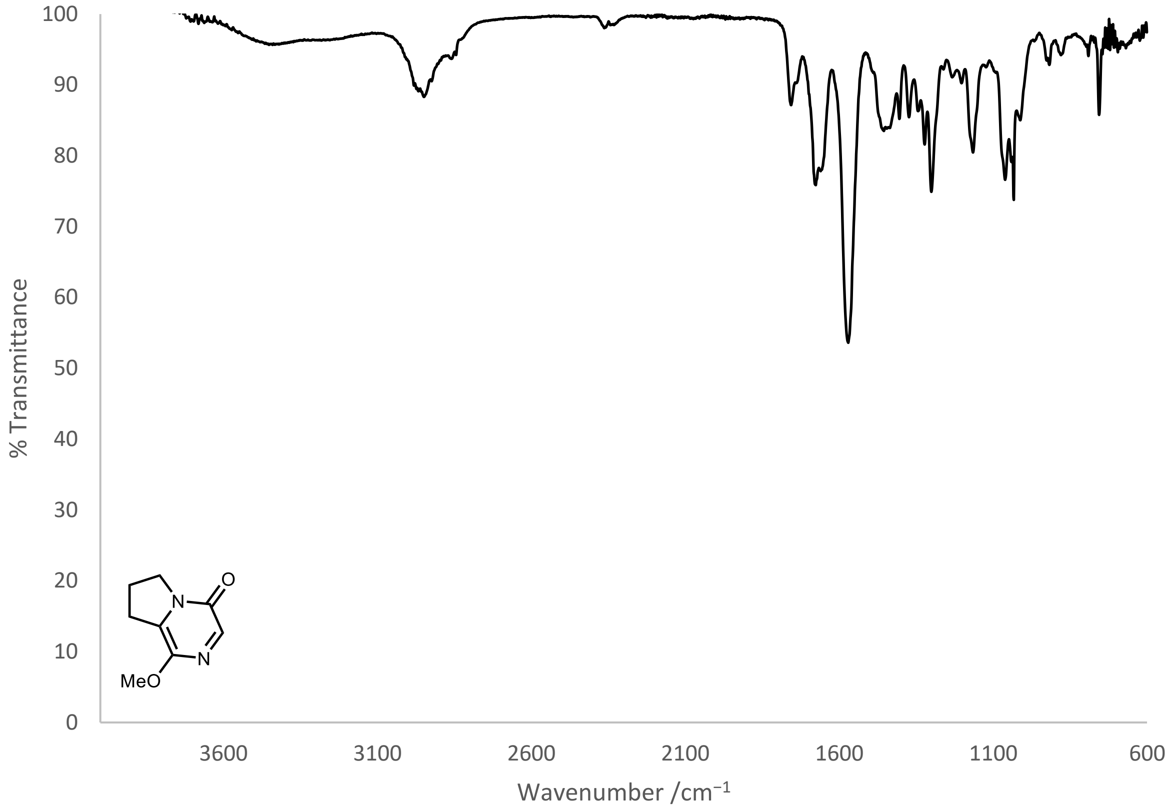

3.22. IR Spectrum for Organotin S-9

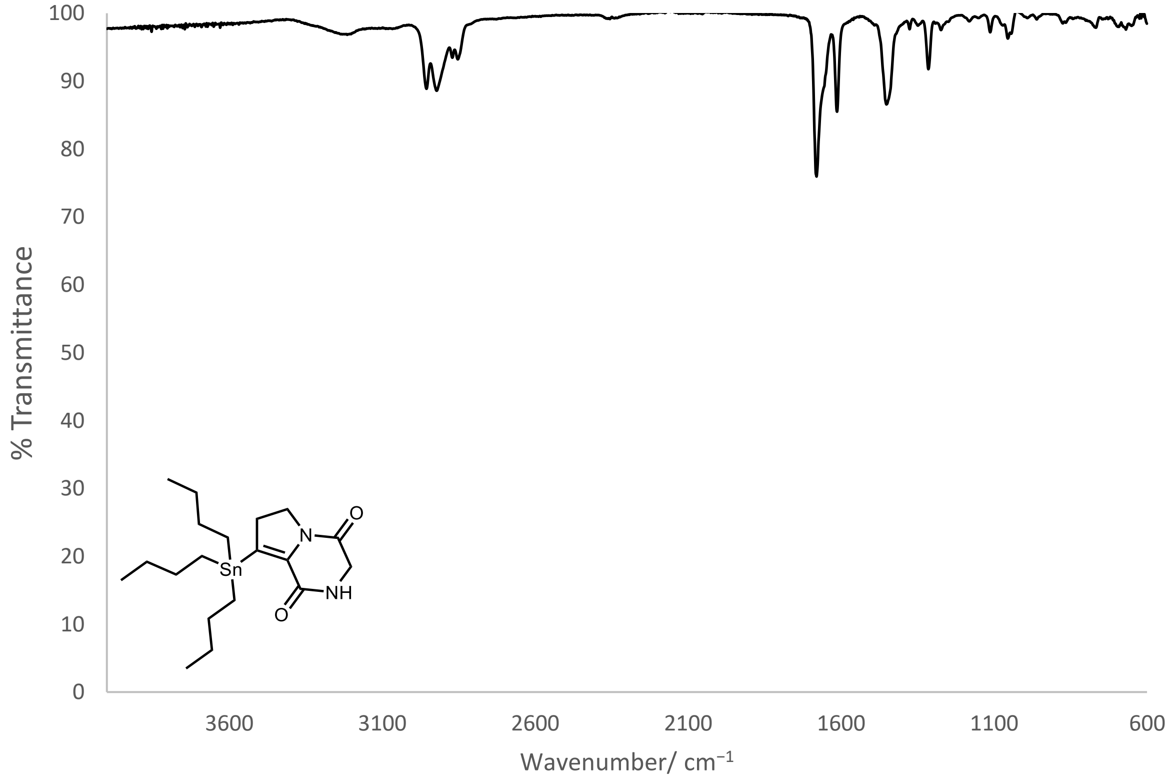

3.23. IR Spectrum for Organotin 15

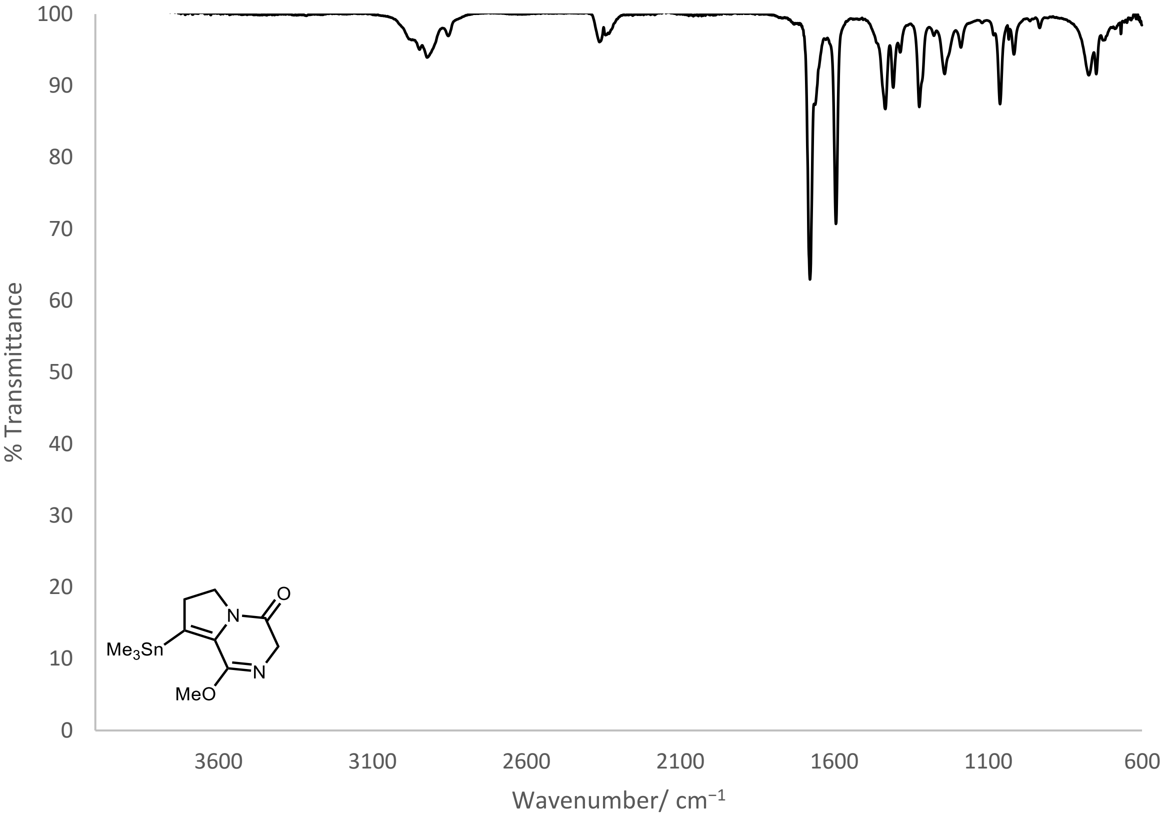

3.24. IR Spectrum for Mono-Adduct 21

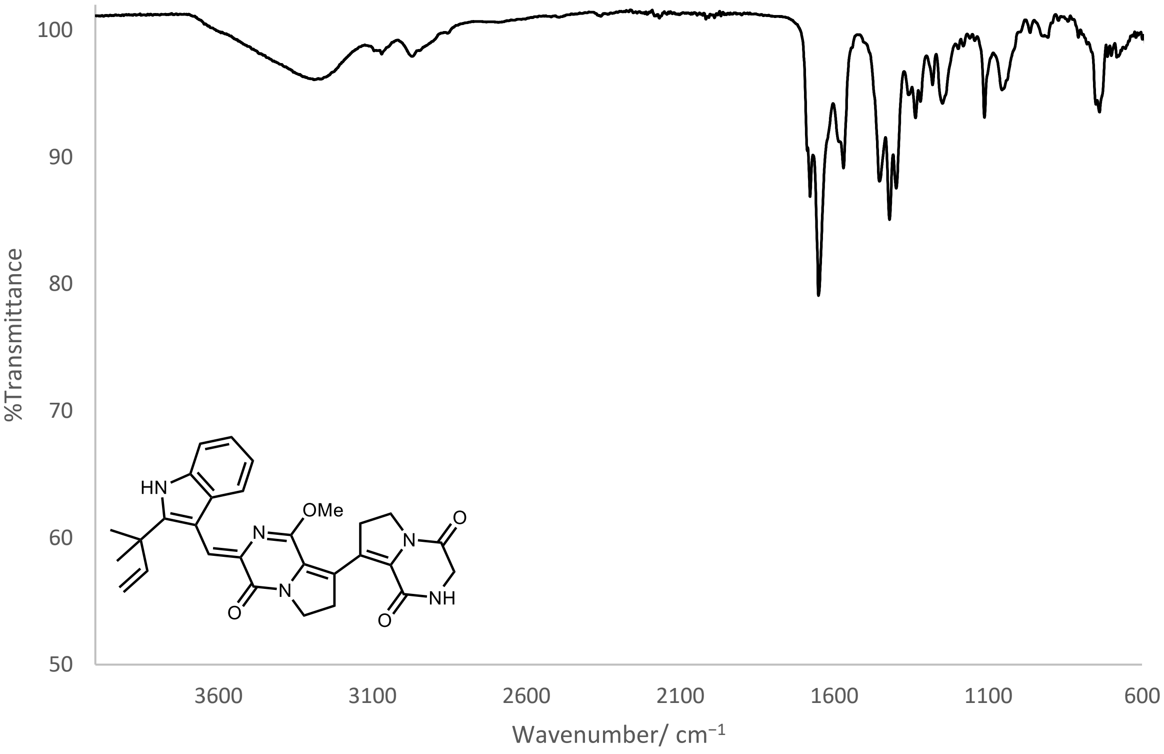

4. X-ray Crystal Structures

4.1. X-ray Crystal Structure Data of Enamide 8: CCDC number 2426509

Submitted by: **Adam Lockyer**

Solved by: **Gary S Nichol**

Sample ID: **AL-045-Fr3**

Fomblin oil was used to coat a selection of crystals which were then mounted on MiTeGen kapton loops and frozen in liquid nitrogen. The loops were stored in a MiTeGen Unipuck and transported to Diamond Light Source. Data were collected remotely at beam line I-19 of Diamond Light Source (award CY30280). Please cite Johnson NT, Waddell PG, Clegg W, Probert MR, Remote access revolution: chemical crystallographers enter a new era at Diamond Light Source beamline I19. Crystals, 2017, 7, (12), 360 in any publication.

Crystal Data and Experimental

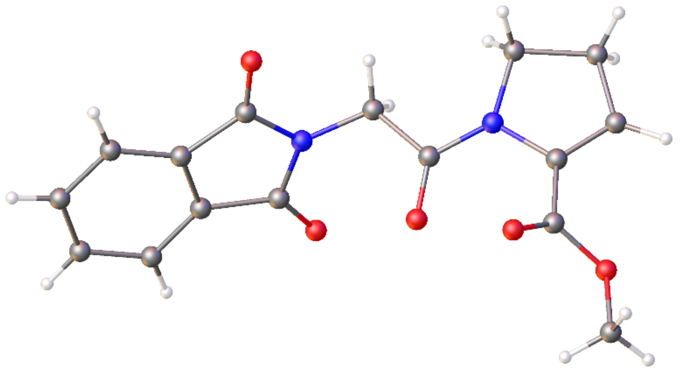

Experimental. Single colourless plate-shaped crystals of **al22005** recrystallised from diethyl ether by slow evaporation. A suitable crystal with dimensions 0.05 × 0.04 × 0.02 mm^3 was selected and mounted on a MITIGEN holder in Paratone oil on a Diamond Light Source I19-1 diffractometer. The crystal was kept at a steady T = 100.0 K during data collection. The structure was solved with the **ShelXT** 2018/2 (Sheldrick, 2018) solution program using dual methods and by using **Olex2** 1.5-beta (Dolomanov et al., 2009) as the graphical interface. The model was refined with **ShelXL** 2018/3 (Sheldrick, 2015) using full matrix least squares minimisation on $\boldsymbol{F}^2$.

Crystal Data. $C_{16}H_{14}N_2O_5$, M_r = 314.29, triclinic, P-1 (No. 2), a = 8.2654(10) Å, b = 8.3867(9) Å, c = 11.3945(13) Å, α = 88.144(3)°, β = 87.718(3)°, γ = 61.737(3)°, V = 695.06(14) Å^3, T = 100.0 K, Z = 2, Z' = 1, μ(synchrotron) = 0.106, 6528 reflections measured, 3717 unique (R_{int} = 0.0382) which were used in all calculations. The final wR_2 was 0.1881 (all data) and R_1 was 0.0660 (I≥2 σ(I)).

**Compound**	**al22005**
Formula	$C_{16}H_{14}N_2O_5$
$D_{calc.}$/ g cm^{-3}	1.502
μ/mm^{-1}	0.106
Formula Weight	314.29
Colour	colourless
Shape	plate-shaped
Size/mm^3	0.05×0.04×0.02
T/K	100.0
Crystal System	triclinic
Space Group	P-1
a/Å	8.2654(10)
b/Å	8.3867(9)
c/Å	11.3945(13)
α/°	88.144(3)
β/°	87.718(3)
γ/°	61.737(3)
V/Å^3	695.06(14)
Z	2
Z'	1
Wavelength/Å	0.6889
Radiation type	synchrotron
Θ_{min}/°	1.734
Θ_{max}/°	31.876
Measured Refl's.	6528
Indep't Refl's	3717
Refl's I≥2 σ(I)	3009
R_{int}	0.0382
Parameters	264
Restraints	0
Largest Peak	0.876
Deepest Hole	-0.396
GooF	1.033
wR_2 (all data)	0.1881
wR_2	0.1792
R_1 (all data)	0.0757
R_1	0.0660

Structure Quality Indicators

**Reflections:**	d min (0.6889) 2Θ=63.8° 0.65	I/σ(I) 16.3	Rint 3.82%	Full 48.8° 71% to 63.8° 91.0
**Refinement:**	Shift 0.000	Max Peak 0.9	Min Peak -0.4	GooF 1.033

A colourless plate-shaped-shaped crystal with dimensions 0.05 × 0.04 × 0.02 mm^3 was mounted on a MITIGEN holder in Paratone oil. Data were collected using a Diamond Light Source I19-1 diffractometer equipped with an Oxford Cryosystems Cryostream 800 low-temperature device operating at T = 100.0 K.

Data were measured using ϕ and ω scans with synchrotron radiation. The diffraction pattern was indexed and the total number of runs and images was based on the strategy calculation from the program Xia2. The maximum resolution that was achieved was Θ = 31.876° (0.65 Å).

The unit cell was refined using SAINT V8.40A (Bruker, 2019) on 3471 reflections, 53% of the observed reflections.

Data reduction, scaling and absorption corrections were performed using SAINT V8.40A (Bruker, 2019). The final completeness is 91.00 % out to 31.876° in Θ. SADABS-2016/2 (Bruker, 2016/2) was used for absorption correction. wR_2(int) was 0.1715 before and 0.0482 after correction. The Ratio of minimum to maximum transmission is 0.7701. The λ/2 correction factor is Not present. The absorption coefficient μ of this material is 0.106 mm^{-1} at this wavelength (λ = 0.68890Å) and the minimum and maximum transmissions are 0.575 and 0.746.

The structure was solved and the space group P-1 (# 2) determined by the ShelXT 2018/2 (Sheldrick, 2018) structure solution program using using dual methods and refined by full matrix least squares minimisation on $\boldsymbol{F^2}$ using version 2018/3 of **ShelXL** 2018/3 (Sheldrick, 2015). All non-hydrogen atoms were refined anisotropically. Hydrogen atom positions were calculated geometrically and refined using the riding model.

_refine_special_details: H atoms were identified from a difference map and freely refined. Data from the first two runs were used. The remaining data were omitted from the final reflection file due to radiation damage.

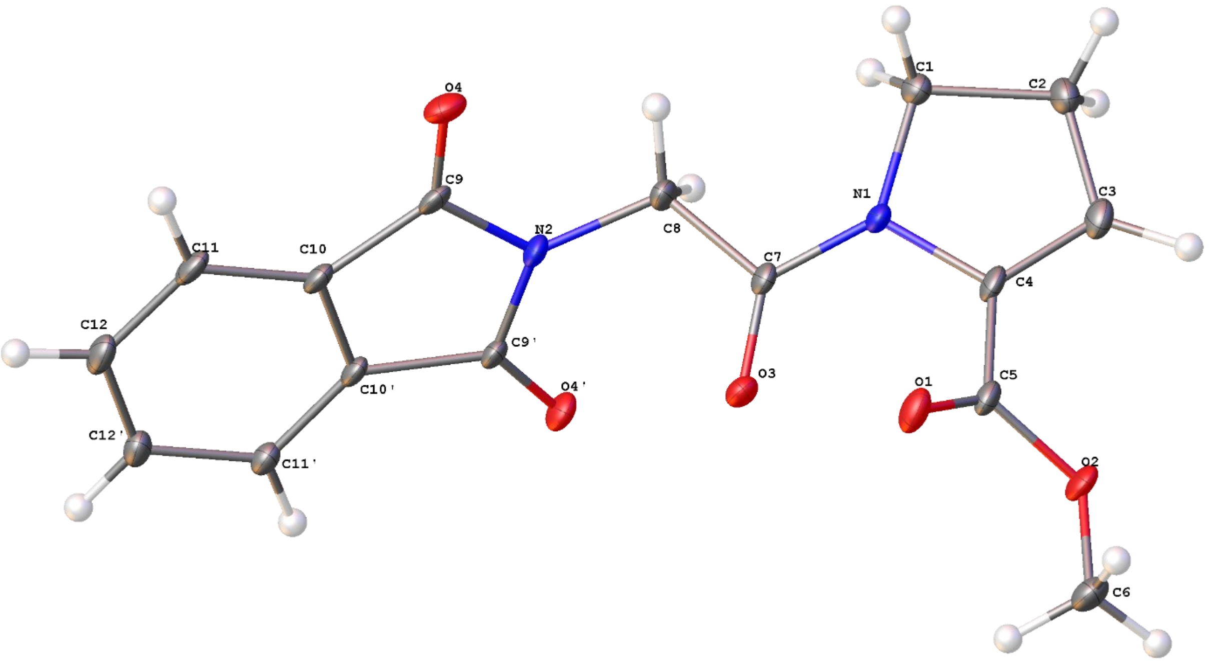

Figure 1: The molecular structure of AL22005. Displacement ellipsoids are at the 50% probability level.

Data Plots: Diffraction Data

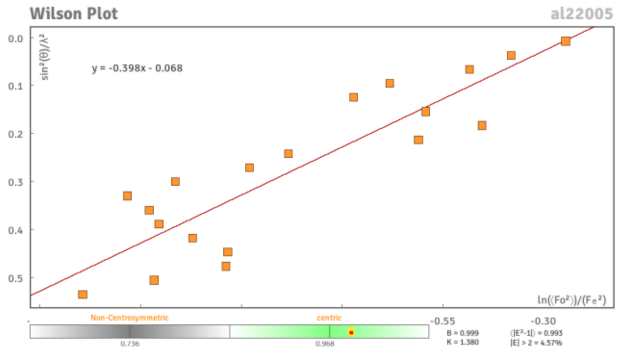

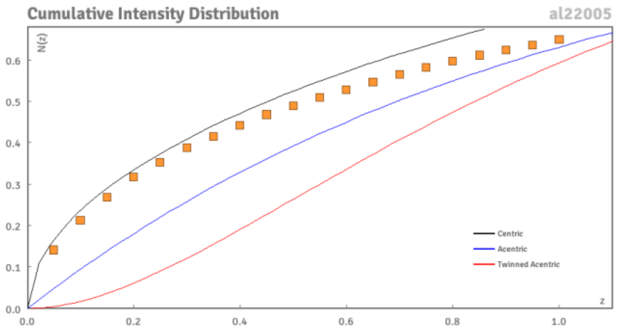

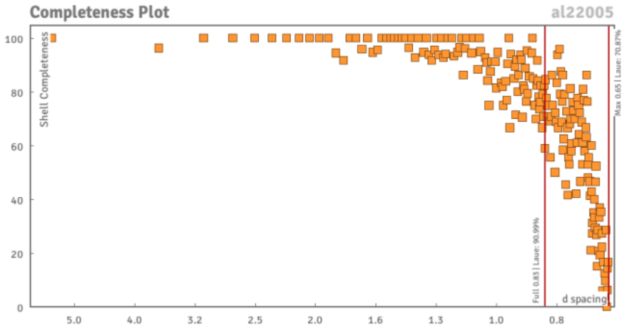

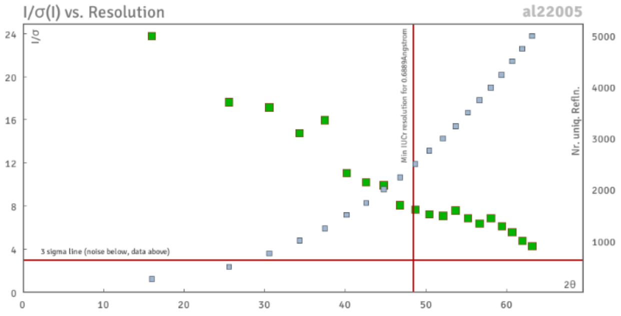

Data Plots: Refinement and Data

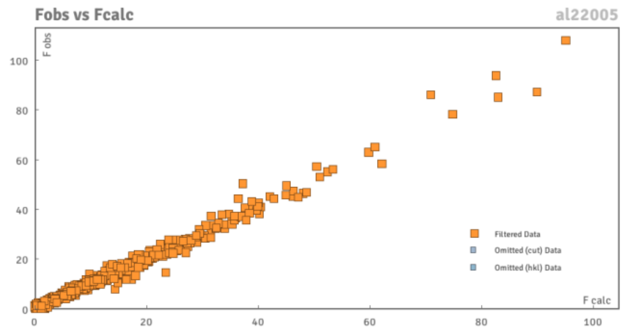

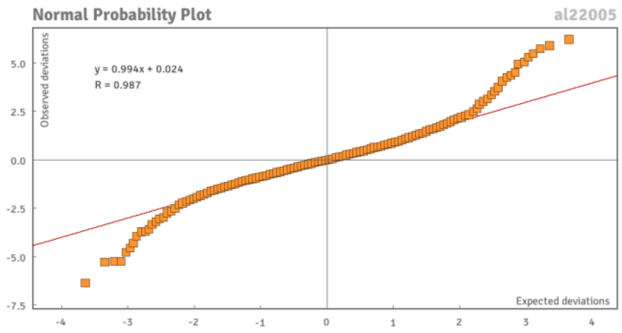

Reflection Statistics

Total reflections (after filtering)	6528	Unique reflections	3717
Completeness	0.709	Mean I/σ	11.32
hkl_{max} collected	(11, 11, 16)	hkl_{min} collected	(-9, -12, -16)
hkl_{max} used	(11, 11, 16)	hkl_{min} used	(-11, -12, 0)
Lim d_{max} collected	100.0	Lim d_{min} collected	0.34
d_{max} used	11.38	d_{min} used	0.65
Friedel pairs	1760	Friedel pairs merged	1
Inconsistent equivalents	1	R_{int}	0.0382
R_{sigma}	0.0613	Intensity transformed	0
Omitted reflections	0	Omitted by user (OMIT hkl)	0
Multiplicity	(4505, 893, 79)	Maximum multiplicity	4
Removed systematic absences	0	Filtered off (Shel/OMIT)	0

Table 1: Fractional Atomic Coordinates (×10⁴) and Equivalent Isotropic Displacement Parameters (Å²×10³) for **al22005**. U_{eq} is defined as 1/3 of the trace of the orthogonalised U_{ij}.

Atom	x	y	z	U_{eq}
O1	10100.8(16)	1662.7(16)	4460.8(8)	20.5(3)
O2	8595.7(16)	2604.5(15)	6204.1(8)	18.8(3)
O3	7888.4(15)	5022.2(15)	3281.1(8)	17.3(3)
O4	7598.9(17)	4434.6(16)	-407.1(9)	21.2(3)
O4'	5575.7(16)	8993.8(16)	2265.9(8)	18.6(3)
N1	6288.4(18)	3435.4(18)	3474.3(9)	14.6(3)
N2	6470.8(18)	6456.1(18)	1129.0(9)	16.0(3)
C1	4683(2)	3181(2)	3186.6(11)	16.5(3)
C2	4134(2)	2576(2)	4357.0(12)	20.0(3)
C3	5705(2)	2193(2)	5139.7(11)	18.5(3)
C4	6862(2)	2699(2)	4617.9(10)	15.1(3)
C5	8697(2)	2289(2)	5045.2(11)	16.0(3)
C6	10349(3)	2124(3)	6718.5(12)	23.5(4)
C7	6741(2)	4642(2)	2928.2(10)	13.5(3)
C8	5660(2)	5540(2)	1829.9(11)	17.4(3)
C9	7355(2)	5826(2)	38.6(11)	14.9(3)
C9'	6336(2)	8130(2)	1399.0(10)	14.2(3)
C10	7843(2)	7225(2)	-427.1(11)	14.8(3)
C10'	7250(2)	8597(2)	393.3(10)	13.9(3)
C11	8689(2)	7315(2)	-1482.7(11)	17.7(3)
C11'	7498(2)	10101(2)	196.0(11)	15.5(3)
C12	8926(2)	8842(2)	-1694.9(12)	19.5(3)
C12'	8347(2)	10208(2)	-867.1(12)	18.3(3)

Table 2: Anisotropic Displacement Parameters (×10⁴) for **al22005**. The anisotropic displacement factor

exponent takes the form: $-2\pi^2[h^2a^{*2} \times U_{11} + ... +2hka^* \times b^* \times U_{12}]$

Atom	U_{11}	U_{22}	U_{33}	U_{23}	U_{13}	U_{12}
O1	24.2(6)	28.1(7)	9.0(4)	1.4(4)	1.0(4)	-12.5(5)
O2	26.5(6)	28.6(7)	6.0(4)	-1.6(4)	-0.5(4)	-16.8(5)
O3	23.0(6)	26.2(7)	9.3(4)	1.6(4)	-3.7(4)	-16.8(5)
O4	30.2(6)	26.3(7)	13.5(5)	-2.9(4)	-4.9(4)	-18.0(6)
O4'	25.5(6)	26.5(7)	7.4(4)	-1.4(4)	2.0(4)	-15.4(5)
N1	21.4(7)	23.4(8)	5.9(4)	0.7(4)	-1.0(4)	-16.3(6)
N2	24.8(7)	23.9(8)	5.6(5)	1.1(4)	-0.6(4)	-16.8(6)
C1	22.1(8)	24.3(9)	9.9(5)	-0.6(5)	0.3(5)	-16.6(7)
C2	28.3(9)	28.7(10)	12.7(6)	0.0(5)	2.3(5)	-21.6(8)
C3	26.6(8)	22.8(9)	9.3(5)	-0.3(5)	2.7(5)	-14.6(7)
C4	22.7(7)	19.6(8)	5.2(5)	0.1(4)	0.5(5)	-11.9(7)
C5	25.5(8)	20.4(9)	6.2(5)	1.5(4)	-1.1(5)	-14.4(7)
C6	31.9(9)	37.8(11)	10.5(6)	1.1(5)	-4.8(6)	-24.1(9)
C7	18.7(7)	19.1(8)	5.8(5)	-0.1(4)	0.8(5)	-11.5(6)
C8	24.6(8)	27.8(9)	9.0(5)	5.2(5)	-5.0(5)	-19.8(8)
C9	18.3(7)	23.3(9)	6.3(5)	0.6(4)	-4.1(5)	-12.3(7)
C9'	19.6(7)	23.0(9)	5.5(5)	1.8(4)	-2.6(5)	-14.5(7)
C10	18.4(7)	24.1(8)	5.6(5)	0.1(4)	-2.2(5)	-13.0(7)
C10'	16.8(7)	24.0(9)	4.8(5)	-0.1(4)	-0.7(5)	-13.0(7)
C11	20.1(7)	30.1(9)	5.8(5)	-1.3(5)	-1.2(5)	-14.0(7)
C11'	19.7(7)	23.5(9)	7.8(5)	1.0(5)	-1.4(5)	-13.8(7)
C12	20.6(8)	34.0(10)	8.2(5)	2.3(5)	0.3(5)	-16.6(8)
C12'	23.1(8)	28.6(10)	9.5(5)	4.2(5)	-2.2(5)	-17.7(8)

Table 3: Bond Lengths in Å for **al22005**.

Atom	Atom	Length/Å
O1	C5	1.2042(19)
O2	C5	1.3467(15)
O2	C6	1.451(2)
O3	C7	1.2199(17)
O4	C9	1.2113(18)
O4'	C9'	1.2088(16)
N1	C1	1.4906(19)
N1	C4	1.4226(17)
N1	C7	1.3603(18)
N2	C8	1.4429(18)
N2	C9	1.4006(17)
N2	C9'	1.3985(19)
C1	C2	1.539(2)

Atom	Atom	Length/Å
C2	C3	1.505(2)
C3	C4	1.331(2)
C4	C5	1.486(2)
C7	C8	1.5239(19)
C9	C10	1.488(2)
C9'	C10'	1.4895(18)
C10	C10'	1.3917(18)
C10	C11	1.3848(18)
C10'	C11'	1.380(2)
C11	C12	1.397(2)
C11'	C12'	1.3976(18)
C12	C12'	1.395(2)

Table 4: Bond Angles in ° for **al22005**.

Atom	Atom	Atom	Angle/°
C5	O2	C6	114.36(12)
C4	N1	C1	108.20(11)
C7	N1	C1	124.92(12)
C7	N1	C4	123.83(12)
C9	N2	C8	123.00(13)
C9'	N2	C8	124.62(11)
C9'	N2	C9	112.17(12)
N1	C1	C2	104.37(11)
C3	C2	C1	103.61(12)
C4	C3	C2	110.84(13)
N1	C4	C5	121.65(12)

Atom	Atom	Atom	Angle/°
C3	C4	N1	111.67(13)
C3	C4	C5	125.89(13)
O1	C5	O2	124.29(14)
O1	C5	C4	125.20(12)
O2	C5	C4	110.37(12)
O3	C7	N1	123.98(13)
O3	C7	C8	121.52(13)
N1	C7	C8	114.50(12)
N2	C8	C7	112.40(12)
O4	C9	N2	124.93(14)
O4	C9	C10	129.30(12)

Atom	Atom	Atom	Angle/°
N2	C9	C10	105.75(12)
O4'	C9'	N2	125.20(13)
O4'	C9'	C10'	129.14(14)
N2	C9'	C10'	105.62(11)
C10'	C10	C9	108.15(11)
C11	C10	C9	130.21(13)
C11	C10	C10'	121.63(14)
C10	C10'	C9'	108.30(13)
C11'	C10'	C9'	130.28(12)
C11'	C10'	C10	121.40(12)
C10	C11	C12	117.33(13)
C10'	C11'	C12'	117.52(13)
C12'	C12	C11	120.99(13)
C12	C12'	C11'	121.14(14)

Table 5: Torsion Angles in ° for **al22005**.

Atom	Atom	Atom	Atom	Angle/°
O3	C7	C8	N2	15.3(2)
O4	C9	C10	C10'	178.69(16)
O4	C9	C10	C11	0.3(3)
O4'	C9'	C10'	C10	-176.84(15)
O4'	C9'	C10'	C11'	1.3(3)
N1	C1	C2	C3	-10.73(16)
N1	C4	C5	O1	-38.7(2)
N1	C4	C5	O2	145.30(14)
N1	C7	C8	N2	-165.93(12)
N2	C9	C10	C10'	0.35(16)
N2	C9	C10	C11	-178.06(16)
N2	C9'	C10'	C10	1.20(16)
N2	C9'	C10'	C11'	179.38(14)
C1	N1	C4	C3	-6.86(17)
C1	N1	C4	C5	163.51(13)
C1	N1	C7	O3	168.84(13)
C1	N1	C7	C8	-9.92(19)
C1	C2	C3	C4	7.33(18)
C2	C3	C4	N1	-0.55(18)
C2	C3	C4	C5	-170.42(13)
C3	C4	C5	O1	130.20(17)
C3	C4	C5	O2	-45.8(2)
C4	N1	C1	C2	10.92(16)
C4	N1	C7	O3	11.0(2)
C4	N1	C7	C8	-167.81(13)
C6	O2	C5	O1	0.0(2)
C6	O2	C5	C4	176.00(13)
C7	N1	C1	C2	-149.86(13)
C7	N1	C4	C3	154.18(14)
C7	N1	C4	C5	-35.5(2)
C8	N2	C9	O4	-3.1(2)
C8	N2	C9	C10	175.36(13)
C8	N2	C9'	O4'	2.3(2)
C8	N2	C9'	C10'	-175.83(13)
C9	N2	C8	C7	109.18(15)
C9	N2	C9'	O4'	177.14(14)
C9	N2	C9'	C10'	-1.00(17)
C9	C10	C10'	C9'	-0.95(16)
C9	C10	C10'	C11'	-179.33(13)
C9	C10	C11	C12	178.25(15)
C9'	N2	C8	C7	-76.53(19)
C9'	N2	C9	O4	-177.99(14)
C9'	N2	C9	C10	0.44(17)
C9'	C10'	C11'	C12'	-177.15(15)
C10	C10'	C11'	C12'	0.8(2)
C10	C11	C12	C12'	0.6(2)
C10'	C10	C11	C12	0.0(2)
C10'	C11'	C12'	C12	-0.2(2)

Atom	Atom	Atom	Atom	Angle/°
C11	C10	C10'	C9'	177.63(13)
C11	C10	C10'	C11'	-0.7(2)
C11	C12	C12'	C11'	-0.5(2)

Table 6: Hydrogen Fractional Atomic Coordinates (×10[4]) and Equivalent Isotropic Displacement Parameters (Å[2]×10[3]) for **al22005**. U_{eq} is defined as 1/3 of the trace of the orthogonalised U_{ij}.

Atom	x	y	z	U_{eq}
H1A	3700(30)	4310(30)	2845(19)	27(5)
H1B	5020(30)	2210(30)	2632(18)	19(5)
H2A	3990(30)	1530(30)	4255(19)	30(6)
H2B	2950(30)	3570(30)	4720(20)	40(6)
H3	5880(30)	1620(30)	5938(19)	25(5)
H6A	11220(30)	670(30)	6660(20)	37(6)
H6B	10820(30)	2860(30)	6320(20)	40(6)
H6C	10010(30)	2480(30)	7510(20)	37(6)
H8A	4360(30)	6500(30)	2106(17)	19(5)
H8B	5550(30)	4690(30)	1310(20)	39(6)
H11	9130(30)	6350(30)	-2100(20)	33(6)
H11'	7080(30)	11030(30)	776(18)	22(5)
H12	9520(30)	8950(30)	-2381(18)	24(5)
H12'	8450(30)	11270(30)	-989(18)	24(5)

Citations

O.V. Dolomanov and L.J. Bourhis and R.J. Gildea and J.A.K. Howard and H. Puschmann, Olex2: A complete structure solution, refinement and analysis program, *J. Appl. Cryst.*, (2009), **42**, 339-341.

SADABS, Bruker axs, Madison, WI (?).

SAINT - Software for the Integration of CCD Detector System Bruker Analytical X-ray Systems, Bruker axs, Madison, WI (?).

Sheldrick, G.M., Crystal structure refinement with ShelXL, *Acta Cryst.*, (2015), **C71**, 3-8.

Sheldrick, G.M., ShelXT-Integrated space-group and crystal-structure determination, *Acta Cryst.*, (2015), **A71**, 3-8.

Xia2

```
#==============================================================================
# PLATON/CHECK-(301021) versus check.def version 210713, Entry: al22005
# Data: al22005.cif - Type: CIF                  Bond Precision   C-C = 0.0021 A
# Refl: al22005.fcf - Type: LIST4                                   Temp = 100 K
# Audit:OLEX2 1.5-BETA (COMPILED 2022.04.07 SVN.RCA3783A0 FOR OLEXSYS, GUI SVN.R
# Refin:SHELXL 2018/3 (SHELDRICK, 2015)
# X-ray Synchrotron       R(int) = 0.038,  wR2/R(int) =  4.9,  Nref/Npar =  14.1
# Cell  8.2654(10)   8.3867(9) 11.3945(13)    88.144(3)    87.718(3)   61.737(3)
# Wavelength 0.68890  Volume Reported      695.06(14) Calculated      695.06(14)
# SpaceGroup from Symmetry P -1         Hall: -P 1                     triclinic
#                 Reported P -1               -P 1                     triclinic
# MoietyFormula C16 H14 N2 O5
#      Reported C16 H14 N2 O5
#    SumFormula C16 H14 N2 O5
#      Reported C16 H14 N2 O5
# Mr         =    314.29[Calc],    314.29[Rep]       Volume/NonHatoms = 15 Ang**3
# Dx,gcm-3  =     1.502[Calc],     1.502[Rep]
# Z          =         2[Calc],         2[Rep]
# Mu (mm-1) =     0.105[Calc],     0.106[Rep]  Xtal Size =  0.023x0.043x0.050 mm
# F000      =     328.0[Calc],     328.0[Rep]  or F000' =    328.16[Calc]
# Reported   T Limits: Tmin=0.575              Tmax=0.746  AbsCorr = MULTI-SCAN
# Measured   HKL: Reported   6528, Embedded   6528, <Mult>  1.8
# Reported   Hmax= 11, Kmax= 12, Lmax= 16, Nref=   3717        , Th(max)= 31.876
# Obs in FCF Hmax= 11, Kmax= 12, Lmax= 16, Nref=   3717[  3717], Th(max)= 31.876
# Calculated Hmax= 12, Kmax= 12, Lmax= 17, Nref=   5244        , Ratio  =  0.709
# Reported   Rho(min) = -0.40, Rho(max) =  0.88 e/Ang**3 (From CIF)
# Calculated Rho(min) = -0.38, Rho(max) =  0.86 e/Ang**3 (From CIF+FCF data)
# w=1/[<sup>2</sup>(Fo<sup>2</sup>)+(0.1435P)<sup>2</sup>],
P=(Fo<sup>2</sup>+2Fc<sup>2</sup>)/3
# R= 0.0660(  3008), wR2= 0.1883(  3717), S = 1.034      (From CIF+FCF data)
# R= 0.0660(  3008), wR2= 0.1881(  3717), S = 1.033      (From FCF data only)
# R= 0.0660(  3009), wR2= 0.1881(  3717), S = 1.033, Npar=  264
#==============================================================================
# For Documentation:http://www.platonsoft.nl/CIF-VALIDATION.pdf
#==============================================================================
*
#==============================================================================
#>>> The Following Improvement and Query ALERTS were generated - (Acta-Mode) <<<
#==============================================================================
Format: alert-number_ALERT_alert-type_alert-level text

029_ALERT_3_A _diffrn_measured_fraction_theta_full value Low .      0.910 Why?
#==============================================================================
097_ALERT_2_B Large Reported Max.  (Positive) Residual Density       0.88 eA-3
#==============================================================================
094_ALERT_2_C Ratio of Maximum / Minimum Residual Density ....       2.21 Report
250_ALERT_2_C Large U3/U1 Ratio for Average U(i,j) Tensor ....        3.6 Note
911_ALERT_3_C Missing FCF Refl Between Thmin & STh/L=    0.600        226 Report
#==============================================================================
072_ALERT_2_G SHELXL First  Parameter in WGHT  Unusually Large       0.14 Report
092_ALERT_4_G Check: Wavelength Given is not Cu,Ga,Mo,Ag,In Ka    0.68890 Ang.
154_ALERT_1_G The s.u.'s on the Cell Angles are Equal ..(Note)      0.003 Degree
432_ALERT_2_G Short Inter X...Y Contact  O1       ..C5               3.02 Ang.
2-x,-y,1-z  =      2_756 Check
912_ALERT_4_G Missing # of FCF Reflections Above STh/L=  0.600       1214 Note
913_ALERT_3_G Missing # of Very Strong Reflections in FCF ....          1 Note
941_ALERT_3_G Average HKL Measurement Multiplicity ..........         1.8 Low
978_ALERT_2_G Number C-C Bonds with Positive Residual Density.         12 Info
984_ALERT_1_G The  N-f'=    0.0037 Deviates from the B&C-Value     0.0056 Check
984_ALERT_1_G The  O-f'=    0.0071 Deviates from the B&C-Value     0.0101 Check
#==============================================================================

ALERT_Level and ALERT_Type Summary
==================================
1 ALERT_Level_A = Most Likely a Serious Problem - Resolve or Explain
1 ALERT_Level_B = A Potentially Serious Problem - Consider Carefully
3 ALERT_Level_C = Check. Ensure it is Not caused by an Omission or Oversight
10 ALERT_Level_G = General Info/Check that it is not Something Unexpected
```

```
3 ALERT_Type_1 CIF Construction/Syntax Error, Inconsistent or Missing Data.
6 ALERT_Type_2 Indicator that the Structure Model may be Wrong or Deficient.
4 ALERT_Type_3 Indicator that the Structure Quality may be Low.
2 ALERT_Type_4 Improvement, Methodology, Query or Suggestion.
#==============================================================================

0 Missing  Experimental  Info Issue(s) (Out of  64 Tests) -  100 % Satisfied
2 Experimental  Data  Related Issue(s) (Out of  35 Tests) -   94 % Satisfied
4 Structural  Model   Related Issue(s) (Out of 136 Tests) -   97 % Satisfied
9 Unresolved or to be Checked Issue(s) (Out of 271 Tests) -   97 % Satisfied

*
```

4.2. X-ray Crystal Structure Data of Alkenyl Iodide 11: CCDC number 2426508

Submitted by: **Adam Lockyer**

Solved by: **Gary S Nichol**

Sample ID: **AL-034-Fr1-A-A**

Compound AL-034-Fr1-A-A was provided as colourless crystals suitable for single crystal X-ray diffraction, yielding structure AL22004.

Crystal Data and Experimental

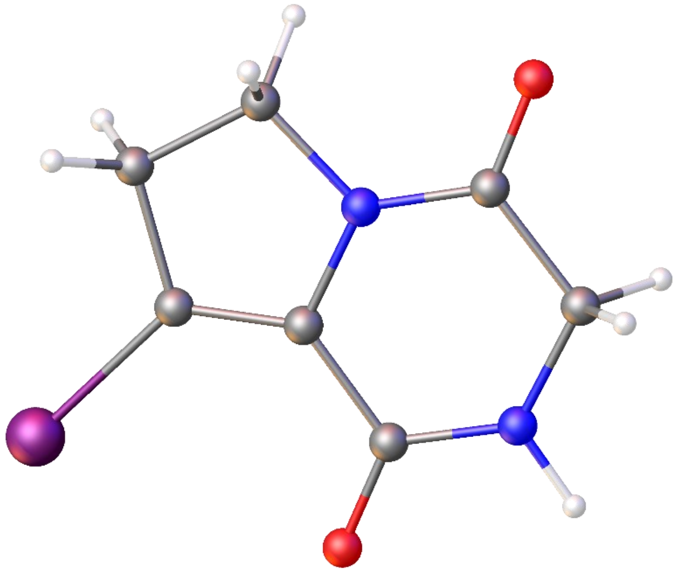

Experimental. Single colourless rod-shaped crystals of **AL22004** recrystallised from dichloromethane by slow evaporation. A suitable crystal with dimensions 0.26 × 0.07 × 0.07 mm^3 was selected and mounted on a MITIGEN holder in Paratone oil on a Bruker D8 VENTURE diffractometer. The crystal was kept at a steady T = 100.00 K during data collection. The structure was solved with the **ShelXS** (Sheldrick, 2008) solution program using direct methods and by using **Olex2** 1.5-beta (Dolomanov et al., 2009) as the graphical interface. The model was refined with **olex2.refine** 1.5-beta (Bourhis et al., 2015) using full matrix least squares minimisation on $\boldsymbol{F^2}$.

Crystal Data. $C_7H_7IN_2O_2$, M_r = 278.050, monoclinic, $P2_1/n$ (No. 14), a = 5.1110(7) Å, b = 9.4118(13) Å, c = 17.588(2) Å, β = 91.965(5)°, $\alpha = \gamma$ = 90°, V = 845.5(2) Å^3, T = 100.00 K, Z = 4, Z' = 1, μ(Mo K$_\alpha$) = 3.746, 100680 reflections measured, 7045 unique (R_{int} = 0.0316) which were used in all calculations. The final wR_2 was 0.0351 (all data) and R_1 was 0.0191 (I≥2 σ(I)).

**Compound**	**AL22004**
Formula	$C_7H_7IN_2O_2$
$D_{calc.}$/ g cm^{-3}	2.184
μ/mm^{-1}	3.746
Formula Weight	278.050
Colour	colourless
Shape	rod-shaped
Size/mm^3	0.26×0.07×0.07
T/K	100.00
Crystal System	monoclinic
Space Group	$P2_1/n$
a/Å	5.1110(7)
b/Å	9.4118(13)
c/Å	17.588(2)
α/°	90
β/°	91.965(5)
γ/°	90
V/Å^3	845.5(2)
Z	4
Z'	1
Wavelength/Å	0.71073
Radiation type	Mo K$_\alpha$
Θ_{min}/°	2.32
Θ_{max}/°	45.33
Measured Refl's.	100680
Indep't Refl's	7045
Refl's I≥2 σ(I)	5988
R_{int}	0.0316
Parameters	172
Restraints	0
Largest Peak	1.6653
Deepest Hole	-1.6608
GooF	1.0405
wR_2 (all data)	0.0351
wR_2	0.0324
R_1 (all data)	0.0271
R_1	0.0191

Structure Quality Indicators

**Reflections:**	d min (Mo) 2Θ=90.7° 0.50	I/σ(I) 58.1	Rint 3.16%	Full 50.5° 99% to 90.7° 100
**Refinement:**	Shift -0.001	Max Peak 1.7	Min Peak -1.7	GooF 1.040

A colourless rod-shaped-shaped crystal with dimensions 0.26 × 0.07 × 0.07 mm^3 was mounted on a MITIGEN holder in Paratone oil. Data were collected using a Bruker D8 VENTURE diffractometer equipped with an Oxford Cryosystems Cryostream 800 low-temperature device operating at T = 100.00 K.

Data were measured using ϕ and ω scans with Mo K_α radiation. The diffraction pattern was indexed and the total number of runs and images was based on the strategy calculation from the program APEX4. The maximum resolution that was achieved was Θ = 45.33° (0.50 Å).

The unit cell was refined using SAINT V8.40B (Bruker, 2016) on 9017 reflections, 9% of the observed reflections.

Data reduction, scaling and absorption corrections were performed using SAINT V8.40B (Bruker, 2016). The final completeness is 100.00 % out to 45.33° in Θ. SADABS-2016/2 (Bruker, 2016/2) was used for absorption correction. wR_2(int) was 0.0832 before and 0.0508 after correction. The Ratio of minimum to maximum transmission is 0.8048. The $\lambda/2$ correction factor is Not present. The absorption coefficient μ of this material is 3.746 mm^{-1} at this wavelength (λ = 0.71073Å) and the minimum and maximum transmissions are 0.603 and 0.749.

The structure was solved and the space group $P2_1/n$ (# 14) determined by the ShelXS (Sheldrick, 2008) structure solution program using using direct methods and refined by full matrix least squares minimisation on F^2 using version of **olex2.refine** 1.5-beta (Bourhis et al., 2015). All non-hydrogen atoms were refined anisotropically. Hydrogen atom positions were calculated geometrically and refined using the riding model.

_*refine_special_details*: Refinement using NoSpherA2, an implementation ofNOn-SPHERical Atom-form-factors in Olex2.Please cite:F. Kleemiss et al. Chem. Sci. DOI 10.1039/D0SC05526C - 2021NoSpherA2 implementation of HAR makes use oftailor-made aspherical atomic form factors calculatedon-the-fly from a Hirshfeld-partitioned electron density (ED) - not fromspherical-atom form factors.The ED is calculated from a gaussian basis set single determinant SCFwavefunction - either Hartree-Fock or DFT using selected funtionals - for a fragment of the crystal.This fragment can be embedded in an electrostatic crystal field by employing cluster chargesor modelled using implicit solvation models, depending on the software used.The following options were used: SOFTWARE: ORCA 5.0 PARTITIONING: NoSpherA2 INT ACCURACY: Normal METHOD: PBE BASIS SET: x2c-TZVP CHARGE: 0 MULTIPLICITY: 1 RELATIVISTIC: DKH2 DATE: 2022-06-09_17-22-39

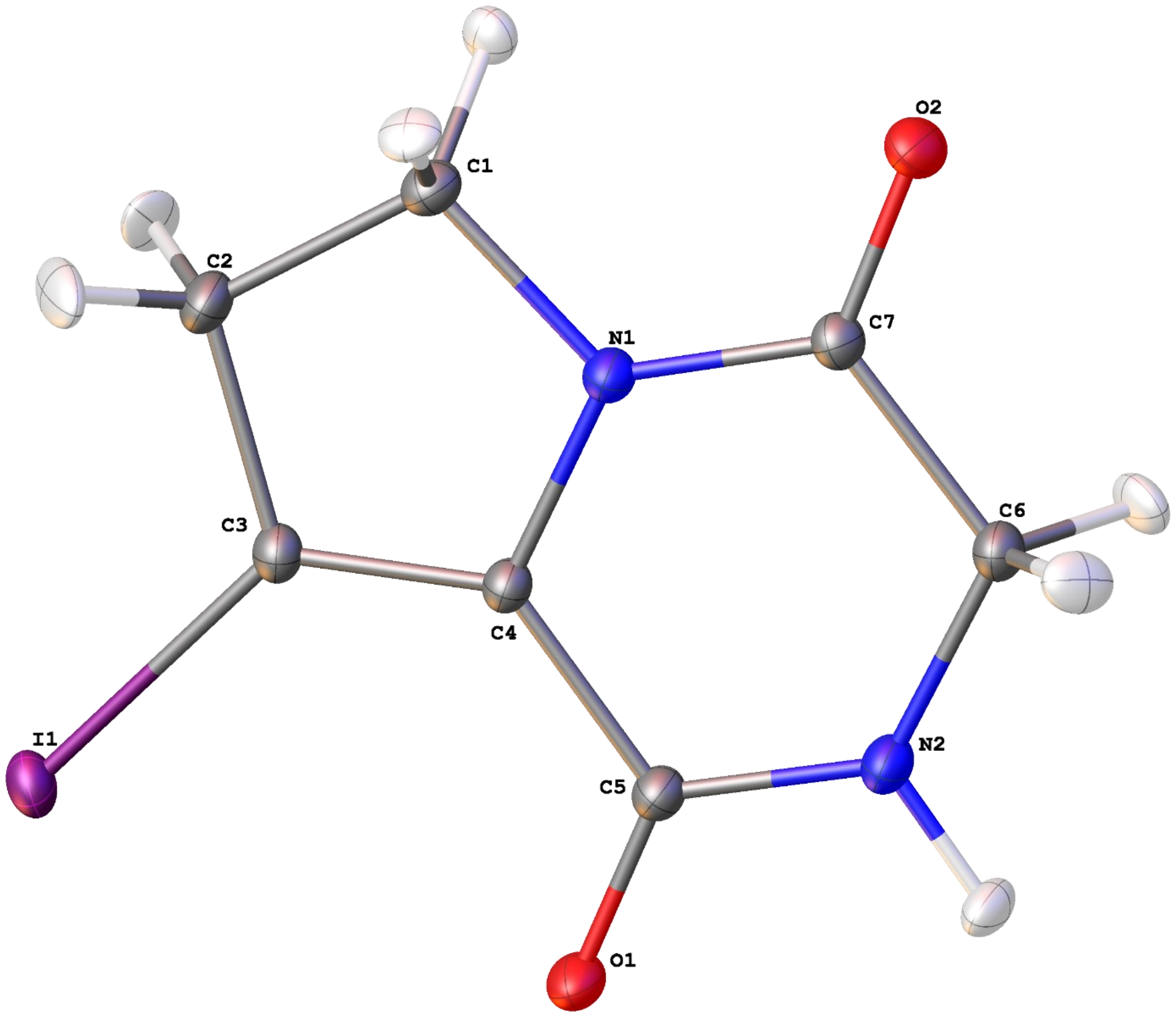

Figure 2: The molecular structure of AL22004. Displacement ellipsoids are at the 50% probability level.

Data Plots: Diffraction Data

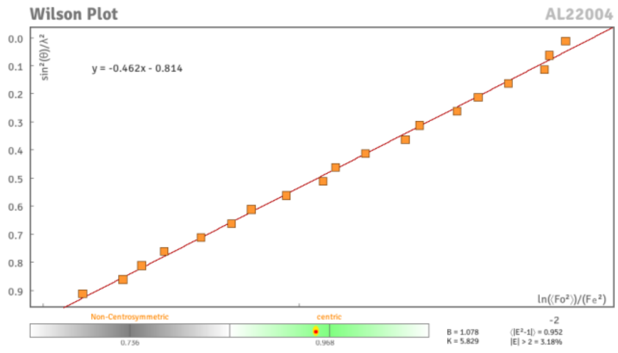

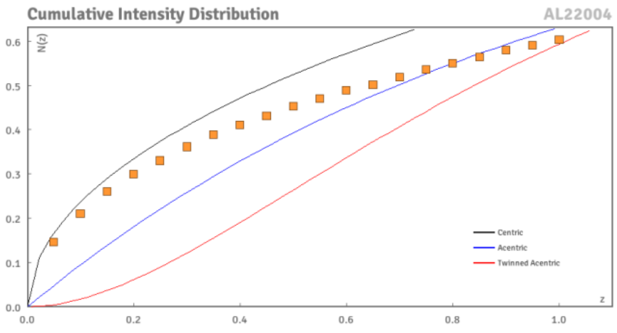

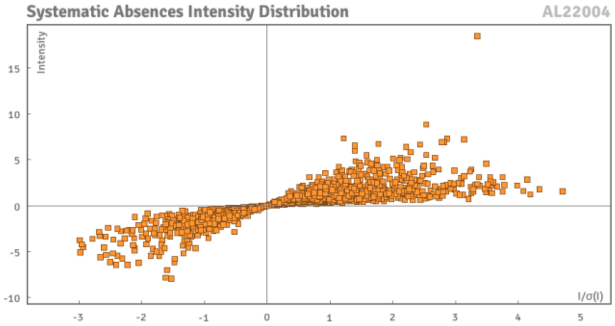

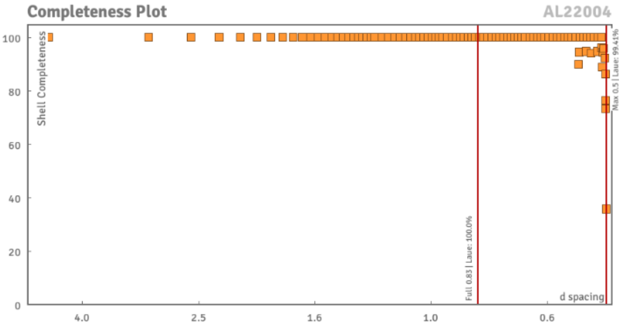

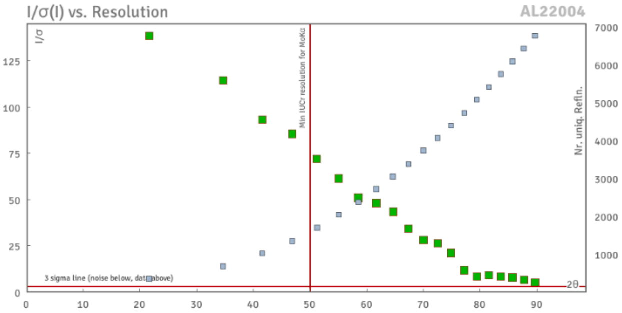

Data Plots: Refinement and Data

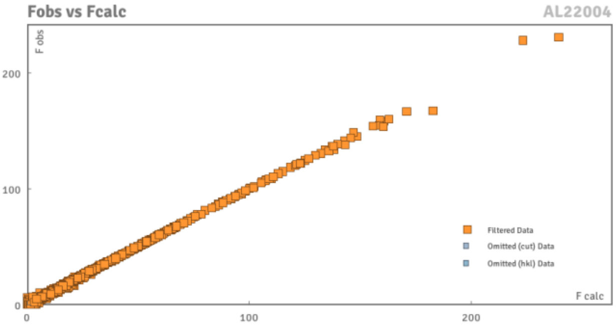

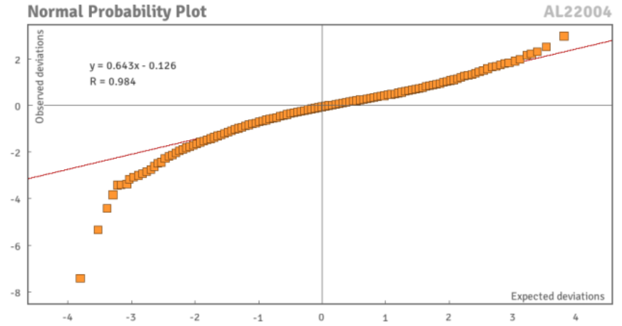

Reflection Statistics

Total reflections (after filtering)	103397	Unique reflections	7045
Completeness	0.994	Mean I/σ	43.97
hkl_{max} collected	(10, 16, 35)	hkl_{min} collected	(-10, -18, -35)
hkl_{max} used	(10, 18, 35)	hkl_{min} used	(-10, 0, 0)
Lim d_{max} collected	100.0	Lim d_{min} collected	0.36
d_{max} used	9.41	d_{min} used	0.5
Friedel pairs	11040	Friedel pairs merged	1
Inconsistent equivalents	0	R_{int}	0.0316
R_{sigma}	0.0172	Intensity transformed	0
Omitted reflections	0	Omitted by user (OMIT hkl)	0
Multiplicity	(5121, 2482, 2608, 4358, 3314, 2678, 2138, 1255, 525, 276, 149, 70, 30, 2, 2)	Maximum multiplicity	50
Removed systematic absences	2717	Filtered off (Shel/OMIT)	0

Table 7: Fractional Atomic Coordinates (×10^4) and Equivalent Isotropic Displacement Parameters (Å^2×10^3) for **AL22004**. U_{eq} is defined as 1/3 of the trace of the orthogonalised U_{ij}.

Atom	x	y	z	U_{eq}
I1	6180.79(10)	8353.34(6)	2460.30(3)	14.116(13)
O1	8540.7(13)	9548.7(8)	847.6(4)	18.16(11)

Atom	x	y	z	U_{eq}
O2	2628.4(14)	6074.8(7)	-933.0(4)	17.60(11)
N1	3707.5(13)	6964.0(8)	225.1(4)	12.7(1)
N2	7392.3(14)	8759.5(8)	-335.0(4)	14.21(11)
C1	1896.2(16)	6062.0(9)	642.8(4)	13.39(11)
C2	2589.9(18)	6380.5(10)	1483.7(5)	16.71(13)
C3	4630.4(15)	7531.7(8)	1455.5(4)	12.34(11)
C4	5219.4(14)	7816.6(8)	732.6(4)	11.18(10)
C5	7172.8(15)	8783.3(8)	421.6(4)	12.48(11)
C6	5868.1(17)	7904.4(9)	-876.8(4)	14.37(12)
C7	3931.8(15)	6905.0(8)	-531.8(4)	12.49(11)

Table 8: Anisotropic Displacement Parameters (×10[4]) for **AL22004.** The anisotropic displacement factor exponent takes the form: $-2\pi^2[h^2a^{*2} \times U_{11} + \ldots +2hka^* \times b^* \times U_{12}]$

Atom	U_{11}	U_{22}	U_{33}	U_{23}	U_{13}	U_{12}
I1	15.87(2)	17.68(2)	8.864(18)	-0.847(16)	1.374(13)	-2.059(15)
O1	19.9(3)	22.6(3)	12.1(2)	-11.4(2)	1.99(19)	-1.6(2)
O2	21.6(3)	19.9(3)	11.2(2)	-7.7(2)	-1.17(19)	-1.18(19)
N1	13.6(2)	14.8(2)	9.7(2)	-4.3(2)	0.92(18)	-0.42(18)
N2	15.7(3)	17.1(3)	9.9(2)	-5.4(2)	1.78(19)	0.60(19)
H2	41(11)	46(12)	21(9)	-21(9)	1(8)	7(8)
C1	12.9(3)	14.2(3)	13.1(3)	-3.6(2)	1.1(2)	0.6(2)
H1a	12(7)	33(9)	35(9)	-3(7)	-1(7)	-1(7)
H1b	29(9)	24(8)	26(8)	3(7)	4(7)	-2(7)
C2	19.2(3)	19.6(3)	11.6(3)	-7.4(3)	4.1(2)	0.6(2)
H2a	30(10)	66(14)	33(10)	-14(9)	19(8)	-21(9)
H2b	66(13)	23(9)	22(9)	-7(9)	1(8)	14(7)
C3	13.3(3)	14.4(3)	9.4(2)	-2.0(2)	2.0(2)	-0.5(2)
C4	11.8(3)	12.9(3)	8.9(2)	-2.7(2)	1.37(19)	0.07(19)
C5	13.3(3)	14.0(3)	10.2(2)	-3.8(2)	1.3(2)	-0.0(2)
C6	17.6(3)	16.2(3)	9.4(2)	-4.5(2)	1.8(2)	-0.2(2)
H6a	35(10)	33(10)	33(10)	0(8)	10(8)	-16(8)
H6b	43(11)	34(10)	28(9)	-4(8)	-17(8)	9(7)
C7	14.0(3)	14.0(3)	9.4(2)	-2.5(2)	0.2(2)	-0.1(2)

Table 9: Bond Lengths in Å for **AL22004**.

Atom	Atom	Length/Å
I1	C3	2.0622(8)
O1	C5	1.2382(10)
O2	C7	1.2329(10)
N1	C1	1.4711(10)
N1	C4	1.4108(10)
N1	C7	1.3411(10)
N2	C5	1.3394(10)

Atom	Atom	Length/Å
N2	C6	1.4531(10)
C1	C2	1.5384(12)
C2	C3	1.5059(11)
C3	C4	1.3437(10)
C4	C5	1.4701(10)
C6	C7	1.5078(11)

Table 10: Bond Angles in ° for **AL22004.**

Atom	Atom	Atom	Angle/°
C4	N1	C1	110.65(6)
C7	N1	C1	123.27(6)
C7	N1	C4	126.01(7)
C6	N2	C5	126.83(7)
C2	C1	N1	103.86(6)

Atom	Atom	Atom	Angle/°
C3	C2	C1	104.21(6)
C2	C3	I1	119.20(5)
C4	C3	I1	130.10(6)
C4	C3	C2	110.65(7)
C3	C4	N1	110.46(6)

Atom	Atom	Atom	Angle/°
C5	C4	N1	118.74(6)
C5	C4	C3	130.74(7)
N2	C5	O1	123.10(7)
C4	C5	O1	120.76(7)
C4	C5	N2	116.13(7)

Atom	Atom	Atom	Angle/°
C7	C6	N2	115.22(6)
N1	C7	O2	122.10(7)
C6	C7	O2	120.95(7)
C6	C7	N1	116.95(7)

Table 11: Torsion Angles in ° for **AL22004**.

Atom	Atom	Atom	Atom	Angle/°
I1	C3	C2	C1	179.27(7)
I1	C3	C4	N1	178.13(7)
I1	C3	C4	C5	1.04(10)
O1	C5	N2	C6	-179.67(8)
O1	C5	C4	N1	-179.95(8)
O1	C5	C4	C3	-3.05(11)
O2	C7	N1	C1	-0.41(11)
O2	C7	N1	C4	176.06(8)
O2	C7	C6	N2	-176.20(8)
N1	C1	C2	C3	4.03(7)
N1	C4	C3	C2	1.00(8)
N1	C4	C5	N2	-1.03(8)
N1	C7	C6	N2	3.68(8)
N2	C5	C4	C3	175.86(7)
C1	C2	C3	C4	-3.24(8)
C2	C3	C4	C5	-176.09(7)

Table 12: Hydrogen Fractional Atomic Coordinates (×10^4) and Equivalent Isotropic Displacement Parameters (Å^2×10^3) for **AL22004**. U_{eq} is defined as 1/3 of the trace of the orthogonalised U_{ij}.

Atom	x	y	z	U_{eq}
H2	8800(30)	9400(20)	-530(9)	36(5)
H1a	-130(30)	6354(17)	485(9)	26(4)
H1b	2210(30)	4951(16)	489(8)	27(3)
H2a	900(30)	6723(19)	1790(10)	43(5)
H2b	3420(40)	5463(17)	1781(9)	37(4)
H6a	7200(30)	7279(18)	-1208(9)	34(4)
H6b	4790(30)	8578(17)	-1273(9)	35(4)

Citations

APEX4

L.J. Bourhis and O.V. Dolomanov and R.J. Gildea and J.A.K. Howard and H. Puschmann, The Anatomy of a Comprehensive Constrained, Restrained, Refinement Program for the Modern Computing Environment - Olex2 Disected, *Acta Cryst. A*, (2015), **A71**, 59-71.

O.V. Dolomanov and L.J. Bourhis and R.J. Gildea and J.A.K. Howard and H. Puschmann, Olex2: A complete structure solution, refinement and analysis program, *J. Appl. Cryst.*, (2009), **42**, 339-341.

SADABS, Bruker axs, Madison, WI (?).

SAINT - Software for the Integration of CCD Detector System Bruker Analytical X-ray Systems, Bruker axs, Madison, WI (?).

Sheldrick, G.M., A short history of ShelX, *Acta Cryst.*, (2008), **A64**, 339-341.

```
#==============================================================================
# PLATON/CHECK-(181221) versus check.def version 211218, Entry: AL22004
# Data: AL22004.cif - Type: CIF                  Bond Precision   C-C = 0.0011 A
# Refl: AL22004.fcf - Type: LIST4                                   Temp = 100 K
# Audit:OLEX2 1.5-BETA (COMPILED 2022.04.07 SVN.RCA3783A0 FOR OLEXSYS, GUI SVN.R
# Refin:OLEX2.REFINE 1.5-BETA (BOURHIS ET AL., 2015)
# X-ray MoKa              R(int) = 0.032,  wR2/R(int) =  1.1,  Nref/Npar =  41.0
# Cell   5.1110(7)  9.4118(13)   17.588(2)           90    91.965(5)          90
# Wavelength 0.71073  Volume Reported        845.5(2) Calculated      845.55(19)
# SpaceGroup from Symmetry P 21/n       Hall: -P 2yn                  monoclinic
#                   Reported P 1 21/n 1         -P 2ybc (x-z,y,z)       monoclinic
# MoietyFormula C7 H7 I N2 O2
#      Reported C7 H7 I N2 O2
#    SumFormula C7 H7 I N2 O2
#      Reported C7 H7 I N2 O2
# Mr        =    278.05[Calc],    278.05[Rep]       Volume/NonHatoms = 18 Ang**3
# Dx,gcm-3  =     2.184[Calc],     2.184[Rep]
# Z         =         4[Calc],         4[Rep]
# Mu (mm-1) =     3.746[Calc],     3.746[Rep]  Xtal Size =  0.065x0.065x0.255 mm
# F000      =     528.0[Calc],     526.7[Rep]  or F000' =    526.31[Calc]
# Reported   T Limits: Tmin=0.603              Tmax=0.749  AbsCorr = MULTI-SCAN
# Calculated T Limits: Tmin=0.747 Tmin'=0.385  Tmax=0.784
# Measured   HKL: Reported 100680, CIF-loop 103397, <Mult> 14.3
# Reported   Hmax= 10, Kmax= 18, Lmax= 35, Nref=   7045        , Th(max)= 45.330
# Obs in FCF Hmax= 10, Kmax= 18, Lmax= 35, Nref=   7045[  7045], Th(max)= 45.330
# Calculated Hmax= 10, Kmax= 18, Lmax= 35, Nref=   7086        , Ratio  =  0.994
# Reported   Rho(min) = -1.66, Rho(max) =  1.67 e/Ang**3 (From CIF)
# Calculated Rho(min) = -1.86, Rho(max) =  1.33 e/Ang**3 (From CIF+FCF data)
# w=1/[<sup>2</sup>(Fo<sup>2</sup>)+(0.0070P)<sup>2</sup>+0.5607P],
P=(Fo<sup>2</sup>+2Fc<sup>2</sup>)/3
# R= 0.0216(  5988), wR2= 0.0411(  7045), S = 1.218      (From CIF+FCF data)
# R= 0.0191(  5988), wR2= 0.0351(  7045), S = 1.040      (From FCF data only)
# R= 0.0191(  5988), wR2= 0.0351(  7045), S = 1.041, Npar=  172
#==============================================================================
# For Documentation:http://www.platonsoft.nl/CIF-VALIDATION.pdf
#==============================================================================
*
#==============================================================================
#>>> The Following Improvement and Query ALERTS were generated - (Acta-Mode) <<<
#==============================================================================
Format: alert-number_ALERT_alert-type_alert-level text

126_ALERT_1_C Error in or Uninterpretable Hall Symbol  ....... -P 2YBC (X Check
353_ALERT_3_C Long   N-H (N0.87,N1.01A)  N2       - H2       .       1.01 Ang.
#==============================================================================
068_ALERT_1_G Reported F000 Differs from Calcd (or Missing)...     Please Check
164_ALERT_4_G Nr. of Refined C-H H-Atoms in Heavy-Atom Struct.          6 Note
431_ALERT_2_G Short Inter HL..A Contact  I1       ..O2       .       2.95 Ang.
1/2+x,3/2-y,1/2+z  =      4_676 Check
802_ALERT_4_G CIF Input Record(s) with more than 80 Characters          1 Info
912_ALERT_4_G Missing # of FCF Reflections Above STh/L=  0.600         42 Note
960_ALERT_3_G Number of Intensities with I  <    - 2*sig(I) ...         11 Check
978_ALERT_2_G Number C-C Bonds with Positive Residual Density.          2 Info
979_ALERT_1_G NoSpherA2 Scattering Factors Used .............     Please Note
#==============================================================================

ALERT_Level and ALERT_Type Summary
==================================
2 ALERT_Level_C = Check. Ensure it is Not caused by an Omission or Oversight
8 ALERT_Level_G = General Info/Check that it is not Something Unexpected

3 ALERT_Type_1 CIF Construction/Syntax Error, Inconsistent or Missing Data.
2 ALERT_Type_2 Indicator that the Structure Model may be Wrong or Deficient.
2 ALERT_Type_3 Indicator that the Structure Quality may be Low.
3 ALERT_Type_4 Improvement, Methodology, Query or Suggestion.
#==============================================================================

0 Missing  Experimental  Info Issue(s) (Out of  64 Tests) -  100 % Satisfied
0 Experimental  Data  Related Issue(s) (Out of  35 Tests) -  100 % Satisfied
```

```
3 Structural  Model   Related Issue(s) (Out of 136 Tests) -   98 % Satisfied
7 Unresolved or to be Checked Issue(s) (Out of 272 Tests) -   97 % Satisfied

*
```

4.3. X-ray Crystal Structure Data of Organotin 12: CCDC number 2426510

Submitted by: **Adam Lockyer**

Solved by: **Gary S Nichol**

Sample ID: **AL-4027-Fr1.5-3-FrOv-Fr3-5**

Compound AL-4027-Fr1.5-3-FrOv-Fr3-5 was provided as crystals suitable for single crystal X-ray diffraction. The crystals degrade when flash-cooled, characterised by a split diffraction which does not yield an acceptable model refinement. Data sets were measured at 298, 250, 200, 150 and 100 K; the best data were obtained at 200 K, structure AL23001b.

Crystal Data and Experimental

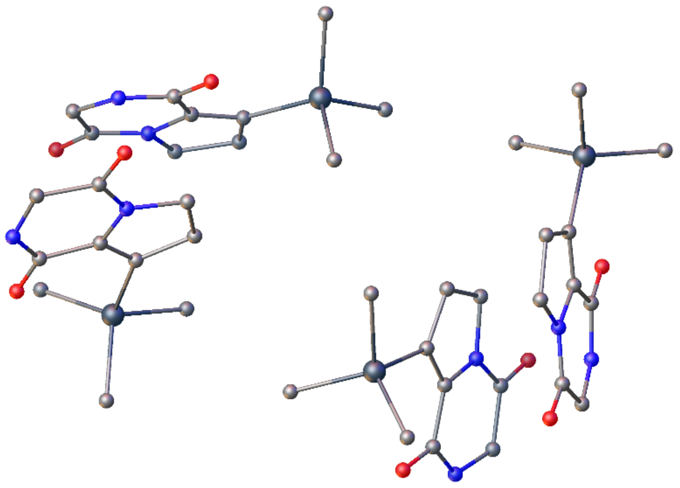

Experimental. Single colourless plate-shaped crystals of **AL23001b** recrystallised from a mixture of ethyl acetate and dichloromethane by slow evaporation. A suitable crystal with dimensions 0.50 × 0.38 × 0.03 mm^3 was selected and mounted on a MITIGEN holder in Paratone oil on a Bruker D8 VENTURE diffractometer. The crystal was kept at a steady T = 200.00 K during data collection. The structure was solved with the **ShelXT** 2018/2 (Sheldrick, 2018) solution program using dual methods and by using **Olex2** 1.5-beta (Dolomanov et al., 2009) as the graphical interface. The model was refined with **ShelXL** 2018/3 (Sheldrick, 2015) using full matrix least squares minimisation on $\boldsymbol{F}^2$.

Crystal Data. $C_{10}H_{16}N_2O_2Sn$, M_r = 314.94, triclinic, P-1 (No. 2), a = 9.7396(5) Å, b = 9.7651(4) Å, c = 27.0075(12) Å, α = 91.9780(10)°, β = 93.727(2)°, γ = 90.119(2)°, V = 2561.6(2) Å^3, T = 200.00 K, Z = 8, Z' = 4, $\mu(MoK_\alpha)$ = 1.980, 115276 reflections measured, 12636 unique (R_{int} = 0.0432) which were used in all calculations. The final wR_2 was 0.0920 (all data) and R_1 was 0.0443 (I≥2 σ(I)).

**Compound**	**AL23001b**
Formula	$C_{10}H_{16}N_2O_2Sn$
$D_{calc.}$/ g cm^{-3}	1.633
μ/mm^{-1}	1.980
Formula Weight	314.94
Colour	colourless
Shape	plate-shaped
Size/mm^3	0.50×0.38×0.03
T/K	200.00
Crystal System	triclinic
Space Group	P-1
a/Å	9.7396(5)
b/Å	9.7651(4)
c/Å	27.0075(12)
α/°	91.9780(10)
β/°	93.727(2)
γ/°	90.119(2)
V/Å^3	2561.6(2)
Z	8
Z'	4
Wavelength/Å	0.71073
Radiation type	MoK_α
Θ_{min}/°	0.756
Θ_{max}/°	28.298
Measured Refl's.	115276
Indep't Refl's	12636
Refl's I≥2 σ(I)	10261
R_{int}	0.0432
Parameters	569
Restraints	0
Largest Peak	0.884
Deepest Hole	-1.180
GooF	1.167
wR_2 (all data)	0.0920
wR_2	0.0866
R_1 (all data)	0.0583
R_1	0.0443

Structure Quality Indicators

**Reflections:**	d min (Mo) 2Θ=56.6° 0.75	I/σ(I) 40.6	Rint m=9.13 4.32%	Full 50.5° 99.9
**Refinement:**	Shift -0.001	Max Peak 0.9	Min Peak -1.2	GooF 1.167

A colourless plate-shaped-shaped crystal with dimensions 0.50 × 0.38 × 0.03 mm^3 was mounted on a MITIGEN holder in Paratone oil. Data were collected using a Bruker D8 VENTURE diffractometer equipped with an Oxford Cryosystems Cryostream 800 low-temperature device operating at T = 200.00 K.

Data were measured using ϕ and ω scans with MoK_α radiation. The diffraction pattern was indexed and the total number of runs and images was based on the strategy calculation from the program APEX4. The maximum resolution that was achieved was Θ = 28.298° (0.75 Å).

The unit cell was refined using SAINT V8.40B (?, 2016) on 9369 reflections, 8% of the observed reflections.

Data reduction, scaling and absorption corrections were performed using SAINT V8.40B (?, 2016). The final completeness is 99.90 % out to 28.298° in Θ. SADABS-2016/2 (Bruker, 2016/2) was used for absorption correction. wR_2(int) was 0.1528 before and 0.0613 after correction. The Ratio of minimum to maximum transmission is 0.8198. The $\lambda/2$ correction factor is Not present. The absorption coefficient μ of this material is 1.980 mm^{-1} at this wavelength (λ = 0.71073Å) and the minimum and maximum transmissions are 0.611 and 0.746.

The structure was solved and the space group P-1 (# 2) determined by the ShelXT 2018/2 (Sheldrick, 2018) structure solution program using using dual methods and refined by full matrix least squares minimisation on $\boldsymbol{F^2}$ using version 2018/3 of **ShelXL** 2018/3 (Sheldrick, 2015). All non-hydrogen atoms were refined anisotropically. Hydrogen atom positions were calculated geometrically and refined using the riding model. Most hydrogen atom positions were calculated geometrically and refined using the riding model, but some hydrogen atoms were refined freely.

_refine_special_details: The crystal degrades when flash-cooled, characterised by a split diffraction which does not yield an acceptable model refinement. Data sets were measured at 298, 250, 200, 150 and 100 K; the best data were obtained at 200 K.N-bound H atoms were identified from a difference map and freely refined. All others were placed using HADD in Olex2.

The value of Z' is 4.

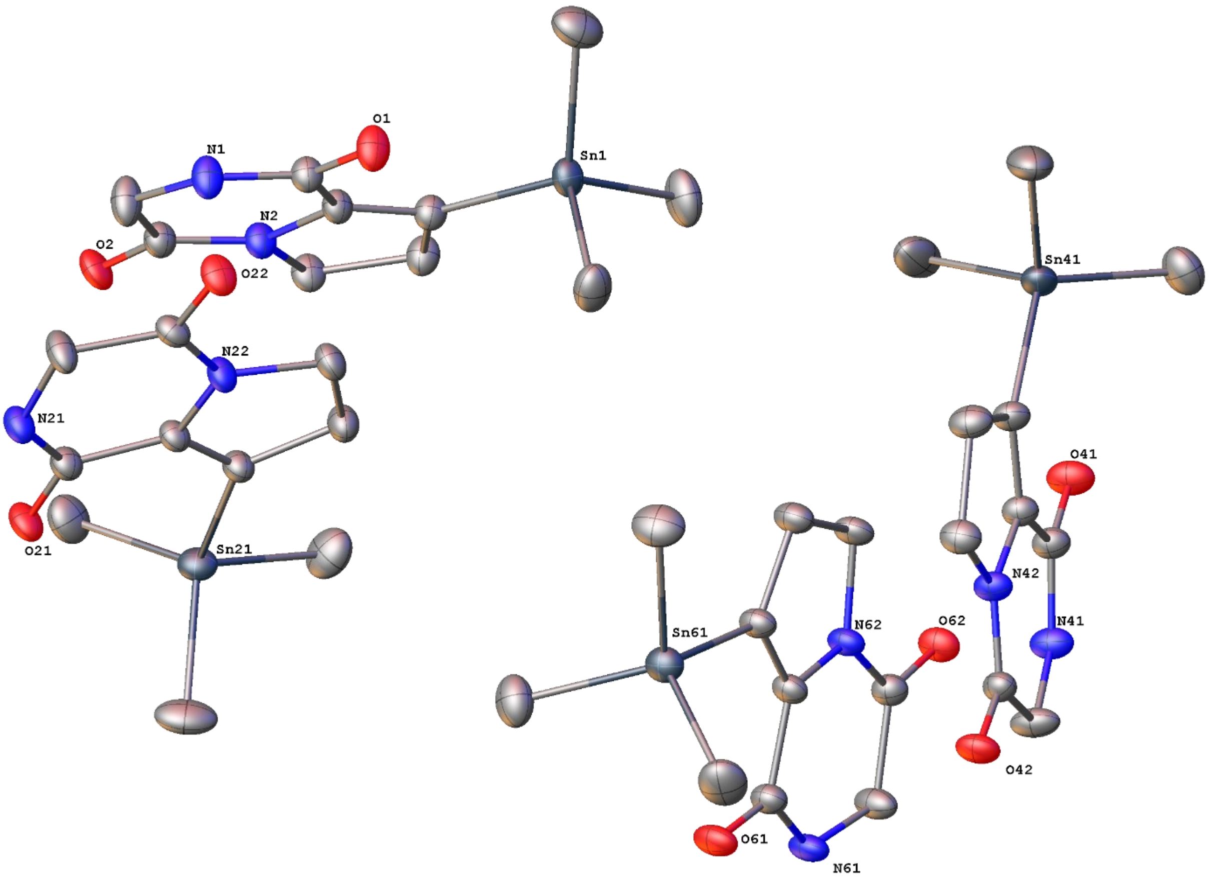

Figure 3: The asymmetric unit of AL23001b. Displacement ellipsoids are at the 30% probability level. C-bound H atoms are not shown.

Data Plots: Diffraction Data

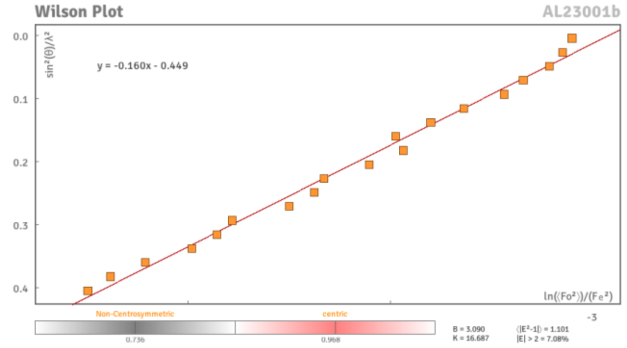

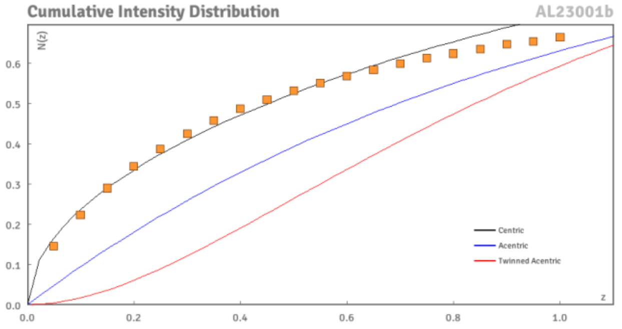

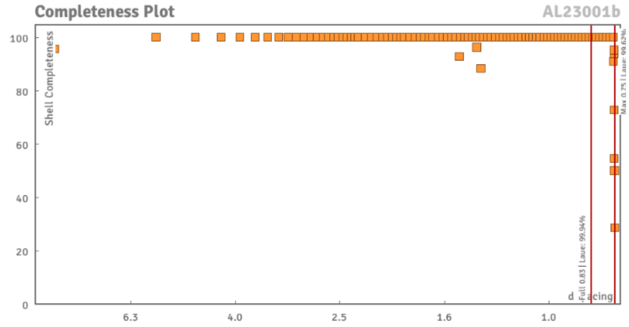

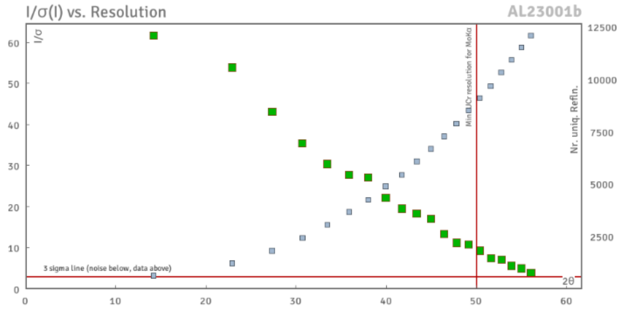

Data Plots: Refinement and Data

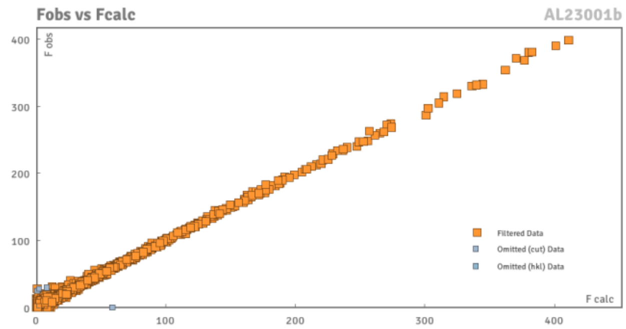

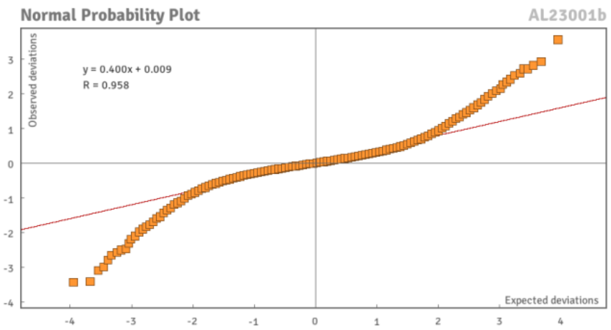

Reflection Statistics

Total reflections (after filtering)	115322	Unique reflections	12636
Completeness	0.996	Mean I/σ	21.58
hkl_{max} collected	(12, 13, 35)	hkl_{min} collected	(-12, -12, -35)
hkl_{max} used	(12, 13, 35)	hkl_{min} used	(-12, -13, 0)
Lim d_{max} collected	100.0	Lim d_{min} collected	0.36
d_{max} used	26.93	d_{min} used	0.75
Friedel pairs	12508	Friedel pairs merged	1
Inconsistent equivalents	0	R_{int}	0.0432
R_{sigma}	0.0246	Intensity transformed	0
Omitted reflections	0	Omitted by user (OMIT hkl)	46
Multiplicity	(578, 1917, 4846, 6353, 4884, 2822, 1576, 1275, 667, 178, 43, 10)	Maximum multiplicity	21
Removed systematic absences	0	Filtered off (Shel/OMIT)	0

Table 13: Fractional Atomic Coordinates (×10[4]) and Equivalent Isotropic Displacement Parameters (Å^2×10^3) for **AL23001b**. U_{eq} is defined as 1/3 of the trace of the orthogonalised U_{ij}.

Atom	x	y	z	U_{eq}
Sn(1)	10344.3(3)	-401.7(3)	1591.9(2)	38.01(8)
O(1)	9973(3)	1495(4)	724.7(13)	44.7(8)
O(2)	4475(3)	1144(3)	372.7(13)	42.2(8)
N(1)	8051(4)	2140(5)	275.8(16)	40.0(10)
N(2)	6422(4)	546(4)	814.4(14)	33.7(8)
C(1)	5779(4)	-355(5)	1160.8(17)	36.6(10)
C(2)	7005(5)	-873(5)	1488.0(18)	38.9(10)
C(3)	8271(5)	-220(5)	1291.3(17)	34.4(9)
C(4)	7866(4)	548(5)	917.7(16)	31.9(9)
C(5)	8716(4)	1430(5)	628.7(17)	34.4(9)
C(6)	6598(5)	2039(6)	127(2)	49.5(13)
C(7)	5740(5)	1202(5)	447.7(17)	35.3(10)
C(8)	10999(7)	1488(6)	1953(2)	58.1(15)
C(9)	10168(7)	-1905(6)	2149(3)	65.5(18)
C(10)	11783(6)	-1082(7)	1083(3)	65.7(17)
Sn(21)	5179.3(3)	4589.7(3)	1575.0(2)	39.22(8)
O(21)	5299(3)	6504(4)	726.4(13)	42.4(8)
O(22)	10650(3)	6115(4)	381.9(13)	41.5(8)
N(21)	7063(4)	7128(4)	274.0(15)	37.3(9)
N(22)	8877(4)	5560(4)	828.2(14)	33.5(8)
C(21)	9635(5)	4690(5)	1182.0(18)	38.1(10)
C(22)	8510(5)	4168(5)	1504.7(18)	41.4(11)
C(23)	7181(5)	4798(5)	1299.8(16)	34.3(9)
C(24)	7467(4)	5564(4)	922.4(16)	31.2(9)
C(25)	6515(4)	6429(5)	627.5(17)	34.9(9)

Atom	x	y	z	U_{eq}
C(26)	8459(5)	7017(6)	127(2)	45.3(12)
C(27)	9410(4)	6204(4)	453.7(17)	33.2(9)
C(28)	3569(6)	4024(7)	1042(2)	61.7(16)
C(29)	4721(7)	6384(7)	1998(3)	70.5(19)
C(30)	5474(7)	2932(6)	2069(2)	65.4(17)
Sn(41)	4714.3(4)	10218.8(3)	6556.5(2)	41.46(9)
O(41)	6590(4)	9913(3)	5705.9(13)	47.3(9)
O(42)	6215(4)	4468(3)	5356.2(13)	41.8(8)
N(41)	7222(5)	8029(4)	5261.9(16)	39.7(9)
N(42)	5671(4)	6354(3)	5799.4(14)	33.8(8)
C(41)	4810(5)	5676(5)	6147.8(18)	40.6(11)
C(42)	4308(6)	6880(5)	6474.0(19)	44.5(12)
C(43)	4908(5)	8158(4)	6270.8(17)	35.1(10)
C(44)	5677(5)	7785(4)	5899.3(16)	33.0(9)
C(45)	6520(5)	8665(4)	5607.5(17)	36.2(10)
C(46)	7120(6)	6587(5)	5115.2(19)	46.6(13)
C(47)	6295(5)	5716(4)	5433.7(16)	34.0(9)
C(48)	6561(7)	10827(7)	6978(3)	78(2)
C(49)	3124(7)	10028(7)	7062(2)	70.9(19)
C(50)	4105(7)	11699(6)	6031(2)	63.2(16)
Sn(61)	10267.4(4)	4946.5(3)	3388.3(2)	39.13(8)
O(61)	8473(4)	4772(3)	4253.3(13)	46.0(9)
O(62)	8883(4)	-604(3)	4597.4(12)	40.3(8)
N(61)	7910(5)	2984(4)	4711.4(15)	39.1(9)
N(62)	9419(4)	1216(3)	4156.2(14)	33.7(8)
C(61)	10268(5)	473(4)	3801.9(18)	37.4(10)
C(62)	10749(6)	1630(5)	3473.9(19)	42.4(11)
C(63)	10127(5)	2932(4)	3674.2(17)	35.2(10)
C(64)	9398(5)	2624(4)	4056.3(17)	33.0(9)
C(65)	8565(5)	3552(4)	4352.1(17)	36.4(10)
C(66)	8029(6)	1569(5)	4856.8(19)	45.5(12)
C(67)	8814(5)	633(4)	4526.4(17)	34.8(9)
C(68)	11769(7)	4704(6)	2843(2)	62.6(16)
C(69)	8348(6)	5481(7)	3026(2)	61.0(16)
C(70)	10960(7)	6512(6)	3911(3)	65.9(17)

Table 14: Anisotropic Displacement Parameters (×10^4) for **AL23001b.** The anisotropic displacement factor exponent takes the form: $-2\pi^2[h^2a^{*2} \times U_{11} + \ldots + 2hka^* \times b^* \times U_{12}]$

Atom	U_{11}	U_{22}	U_{33}	U_{23}	U_{13}	U_{12}
Sn(1)	30.94(16)	39.53(17)	43.04(18)	6.33(14)	-4.11(13)	-1.43(13)
O(1)	27.0(16)	61(2)	47(2)	18.6(17)	1.0(14)	-7.0(15)
O(2)	23.2(15)	46.3(19)	58(2)	12.7(16)	3.8(14)	8.9(14)
N(1)	25.6(19)	53(3)	42(2)	19.1(19)	3.3(16)	-3.5(17)
N(2)	24.3(18)	40(2)	37(2)	8.5(16)	4.5(15)	-0.9(15)
C(1)	25(2)	44(3)	42(2)	9(2)	6.6(18)	-4.5(19)
C(2)	33(2)	46(3)	38(2)	11(2)	2.7(19)	-6(2)
C(3)	29(2)	40(2)	35(2)	5.3(19)	3.0(18)	-3.1(18)
C(4)	25(2)	37(2)	34(2)	5.5(18)	1.9(17)	-1.4(17)
C(5)	29(2)	39(2)	36(2)	7.8(19)	4.0(18)	-2.2(18)
C(6)	29(2)	70(4)	51(3)	27(3)	-3(2)	-6(2)
C(7)	29(2)	40(2)	38(2)	5.2(19)	1.9(18)	-1.3(18)
C(8)	64(4)	50(3)	57(3)	1(3)	-18(3)	-6(3)
C(9)	60(4)	59(4)	80(4)	38(3)	-4(3)	-1(3)
C(10)	43(3)	68(4)	86(5)	-9(3)	14(3)	4(3)
Sn(21)	36.87(18)	41.65(18)	40.71(18)	1.64(14)	14.48(14)	-2.76(14)
O(21)	26.4(16)	52(2)	51(2)	14.9(16)	10.1(14)	10.4(14)
O(22)	23.8(15)	50(2)	52(2)	14.9(16)	8.2(14)	-2.0(14)
N(21)	27.4(19)	45(2)	41(2)	14.5(18)	6.6(16)	6.9(16)

Atom	U_{11}	U_{22}	U_{33}	U_{23}	U_{13}	U_{12}
N(22)	23.4(17)	40(2)	38(2)	10.2(16)	3.8(15)	4.5(15)
C(21)	29(2)	41(2)	45(3)	11(2)	3.0(19)	7.4(19)
C(22)	36(2)	52(3)	37(2)	12(2)	4(2)	8(2)
C(23)	30(2)	39(2)	34(2)	5.5(18)	6.6(18)	6.0(18)
C(24)	24(2)	35(2)	36(2)	4.7(18)	5.3(17)	3.4(17)
C(25)	27(2)	41(2)	38(2)	4.5(19)	4.7(18)	2.5(18)
C(26)	28(2)	61(3)	50(3)	26(2)	10(2)	11(2)
C(27)	25(2)	33(2)	43(2)	3.3(19)	8.4(18)	2.3(17)
C(28)	37(3)	70(4)	78(4)	5(3)	-4(3)	-1(3)
C(29)	65(4)	64(4)	86(5)	-13(3)	48(4)	-8(3)
C(30)	86(5)	60(4)	52(3)	18(3)	7(3)	-14(3)
Sn(41)	48.0(2)	36.23(17)	39.09(18)	-8.79(14)	-0.21(15)	7.34(15)
O(41)	62(2)	28.5(16)	53(2)	-2.6(15)	19.8(18)	-7.4(16)
O(42)	54(2)	23.6(15)	50(2)	-2.9(14)	18.7(16)	2.1(14)
N(41)	52(3)	28.3(19)	41(2)	0.1(16)	19.1(19)	-5.5(18)
N(42)	44(2)	22.7(17)	35.9(19)	-1.2(14)	11.8(16)	-3.5(15)
C(41)	47(3)	33(2)	44(3)	5(2)	16(2)	-3(2)
C(42)	55(3)	40(3)	41(3)	0(2)	18(2)	-4(2)
C(43)	41(3)	30(2)	34(2)	-3.5(18)	8.6(19)	-1.0(19)
C(44)	38(2)	27(2)	34(2)	-1.5(17)	7.5(18)	-3.6(18)
C(45)	44(3)	29(2)	37(2)	-1.3(18)	9(2)	-3.3(19)
C(46)	68(4)	28(2)	47(3)	-5(2)	27(3)	-5(2)
C(47)	44(3)	27(2)	32(2)	0.9(17)	5.1(19)	4.4(18)
C(48)	65(4)	79(5)	83(5)	-40(4)	-23(4)	17(4)
C(49)	76(5)	85(5)	54(4)	-1(3)	21(3)	32(4)
C(50)	66(4)	46(3)	77(4)	12(3)	-1(3)	8(3)
Sn(61)	46.0(2)	30.34(16)	42.24(18)	5.86(13)	9.21(15)	-2.82(14)
O(61)	64(2)	26.5(16)	51(2)	5.7(14)	22.6(18)	10.7(15)
O(62)	50(2)	24.0(15)	48.3(19)	4.3(13)	14.0(16)	-1.4(14)
N(61)	52(3)	26.7(18)	41(2)	2.9(16)	19.5(19)	10.0(17)
N(62)	42(2)	23.0(17)	37(2)	2.0(15)	13.4(16)	4.8(15)
C(61)	44(3)	25(2)	44(3)	-2.1(18)	14(2)	4.4(19)
C(62)	53(3)	33(2)	43(3)	3(2)	16(2)	8(2)
C(63)	43(3)	26(2)	38(2)	2.5(17)	12(2)	3.8(18)
C(64)	38(2)	25(2)	38(2)	2.7(17)	9.7(19)	3.8(17)
C(65)	43(3)	30(2)	38(2)	1.4(18)	10(2)	4.7(19)
C(66)	63(3)	32(2)	45(3)	9(2)	24(2)	11(2)
C(67)	40(2)	28(2)	37(2)	0.1(18)	7.3(19)	-2.2(18)
C(68)	66(4)	56(3)	69(4)	9(3)	31(3)	-8(3)
C(69)	53(3)	68(4)	63(4)	28(3)	1(3)	-3(3)
C(70)	78(5)	38(3)	80(4)	-8(3)	-4(4)	-6(3)

Table 15: Bond Lengths in Å for **AL23001b**.

Atom	Atom	Length/Å
Sn(1)	C(3)	2.137(4)
Sn(1)	C(8)	2.134(5)
Sn(1)	C(9)	2.153(5)
Sn(1)	C(10)	2.118(6)
O(1)	C(5)	1.235(5)
O(2)	C(7)	1.237(5)
N(1)	C(5)	1.332(6)
N(1)	C(6)	1.448(6)
N(2)	C(1)	1.474(5)
N(2)	C(4)	1.415(5)
N(2)	C(7)	1.339(6)
C(1)	C(2)	1.535(6)
C(2)	C(3)	1.521(6)
C(3)	C(4)	1.318(6)

Atom	Atom	Length/Å
C(4)	C(5)	1.471(6)
C(6)	C(7)	1.502(6)
Sn(21)	C(23)	2.143(4)
Sn(21)	C(28)	2.119(6)
Sn(21)	C(29)	2.123(6)
Sn(21)	C(30)	2.142(6)
O(21)	C(25)	1.233(5)
O(22)	C(27)	1.238(5)
N(21)	C(25)	1.331(6)
N(21)	C(26)	1.444(6)
N(22)	C(21)	1.465(5)
N(22)	C(24)	1.412(5)
N(22)	C(27)	1.341(5)
C(21)	C(22)	1.540(6)

Atom	Atom	Length/Å
C(22)	C(23)	1.515(6)
C(23)	C(24)	1.329(6)
C(24)	C(25)	1.471(6)
C(26)	C(27)	1.487(6)
Sn(41)	C(43)	2.144(4)
Sn(41)	C(48)	2.137(6)
Sn(41)	C(49)	2.142(6)
Sn(41)	C(50)	2.119(6)
O(41)	C(45)	1.239(5)
O(42)	C(47)	1.230(5)
N(41)	C(45)	1.330(6)
N(41)	C(46)	1.452(6)
N(42)	C(41)	1.473(5)
N(42)	C(44)	1.415(5)
N(42)	C(47)	1.330(5)
C(41)	C(42)	1.544(6)
C(42)	C(43)	1.511(6)
C(43)	C(44)	1.332(6)

Atom	Atom	Length/Å
C(44)	C(45)	1.470(6)
C(46)	C(47)	1.499(6)
Sn(61)	C(63)	2.145(4)
Sn(61)	C(68)	2.150(5)
Sn(61)	C(69)	2.127(6)
Sn(61)	C(70)	2.124(6)
O(61)	C(65)	1.232(5)
O(62)	C(67)	1.230(5)
N(61)	C(65)	1.332(6)
N(61)	C(66)	1.452(6)
N(62)	C(61)	1.478(5)
N(62)	C(64)	1.410(5)
N(62)	C(67)	1.337(5)
C(61)	C(62)	1.549(6)
C(62)	C(63)	1.509(6)
C(63)	C(64)	1.333(6)
C(64)	C(65)	1.468(6)
C(66)	C(67)	1.500(6)

Table 16: Bond Angles in ° for **AL23001b**.

Atom	Atom	Atom	Angle/°
C(3)	Sn(1)	C(9)	102.6(2)
C(8)	Sn(1)	C(3)	109.7(2)
C(8)	Sn(1)	C(9)	108.3(3)
C(10)	Sn(1)	C(3)	115.5(2)
C(10)	Sn(1)	C(8)	110.8(3)
C(10)	Sn(1)	C(9)	109.5(3)
C(5)	N(1)	C(6)	126.0(4)
C(4)	N(2)	C(1)	109.2(3)
C(7)	N(2)	C(1)	124.8(4)
C(7)	N(2)	C(4)	126.0(4)
N(2)	C(1)	C(2)	103.6(3)
C(3)	C(2)	C(1)	105.5(4)
C(2)	C(3)	Sn(1)	126.4(3)
C(4)	C(3)	Sn(1)	125.3(3)
C(4)	C(3)	C(2)	108.3(4)
N(2)	C(4)	C(5)	118.5(4)
C(3)	C(4)	N(2)	113.5(4)
C(3)	C(4)	C(5)	127.9(4)
O(1)	C(5)	N(1)	123.8(4)
O(1)	C(5)	C(4)	119.9(4)
N(1)	C(5)	C(4)	116.3(4)
N(1)	C(6)	C(7)	116.2(4)
O(2)	C(7)	N(2)	122.9(4)
O(2)	C(7)	C(6)	120.8(4)
N(2)	C(7)	C(6)	116.3(4)
C(28)	Sn(21)	C(23)	116.1(2)
C(28)	Sn(21)	C(29)	112.6(3)
C(28)	Sn(21)	C(30)	107.6(3)
C(29)	Sn(21)	C(23)	109.2(2)
C(29)	Sn(21)	C(30)	108.5(3)
C(30)	Sn(21)	C(23)	102.1(2)
C(25)	N(21)	C(26)	126.1(4)
C(24)	N(22)	C(21)	109.7(3)
C(27)	N(22)	C(21)	125.9(4)
C(27)	N(22)	C(24)	124.4(4)
N(22)	C(21)	C(22)	103.5(3)
C(23)	C(22)	C(21)	105.6(4)

Atom	Atom	Atom	Angle/°
C(22)	C(23)	Sn(21)	127.2(3)
C(24)	C(23)	Sn(21)	124.7(3)
C(24)	C(23)	C(22)	108.1(4)
N(22)	C(24)	C(25)	119.2(4)
C(23)	C(24)	N(22)	113.2(4)
C(23)	C(24)	C(25)	127.5(4)
O(21)	C(25)	N(21)	124.2(4)
O(21)	C(25)	C(24)	119.7(4)
N(21)	C(25)	C(24)	116.0(4)
N(21)	C(26)	C(27)	115.8(4)
O(22)	C(27)	N(22)	120.9(4)
O(22)	C(27)	C(26)	121.4(4)
N(22)	C(27)	C(26)	117.7(4)
C(48)	Sn(41)	C(43)	109.9(2)
C(48)	Sn(41)	C(49)	107.9(3)
C(49)	Sn(41)	C(43)	102.3(2)
C(50)	Sn(41)	C(43)	116.2(2)
C(50)	Sn(41)	C(48)	111.3(3)
C(50)	Sn(41)	C(49)	108.6(3)
C(45)	N(41)	C(46)	126.0(4)
C(44)	N(42)	C(41)	109.6(3)
C(47)	N(42)	C(41)	124.8(4)
C(47)	N(42)	C(44)	125.5(4)
N(42)	C(41)	C(42)	103.2(4)
C(43)	C(42)	C(41)	105.7(4)
C(42)	C(43)	Sn(41)	126.9(3)
C(44)	C(43)	Sn(41)	124.6(3)
C(44)	C(43)	C(42)	108.4(4)
N(42)	C(44)	C(45)	119.0(4)
C(43)	C(44)	N(42)	113.0(4)
C(43)	C(44)	C(45)	128.0(4)
O(41)	C(45)	N(41)	124.0(4)
O(41)	C(45)	C(44)	119.8(4)
N(41)	C(45)	C(44)	116.1(4)
N(41)	C(46)	C(47)	115.9(4)
O(42)	C(47)	N(42)	122.4(4)
O(42)	C(47)	C(46)	120.6(4)

Atom	Atom	Atom	Angle/°
N(42)	C(47)	C(46)	117.0(4)
C(63)	Sn(61)	C(68)	103.1(2)
C(69)	Sn(61)	C(63)	109.6(2)
C(69)	Sn(61)	C(68)	109.0(3)
C(70)	Sn(61)	C(63)	115.7(2)
C(70)	Sn(61)	C(68)	108.4(3)
C(70)	Sn(61)	C(69)	110.7(3)
C(65)	N(61)	C(66)	126.0(4)
C(64)	N(62)	C(61)	109.8(3)
C(67)	N(62)	C(61)	124.4(4)
C(67)	N(62)	C(64)	125.7(4)
N(62)	C(61)	C(62)	102.8(3)
C(63)	C(62)	C(61)	105.9(4)

Atom	Atom	Atom	Angle/°
C(62)	C(63)	Sn(61)	127.4(3)
C(64)	C(63)	Sn(61)	124.2(3)
C(64)	C(63)	C(62)	108.3(4)
N(62)	C(64)	C(65)	119.1(4)
C(63)	C(64)	N(62)	113.2(4)
C(63)	C(64)	C(65)	127.6(4)
O(61)	C(65)	N(61)	123.8(4)
O(61)	C(65)	C(64)	120.1(4)
N(61)	C(65)	C(64)	116.0(4)
N(61)	C(66)	C(67)	116.2(4)
O(62)	C(67)	N(62)	122.6(4)
O(62)	C(67)	C(66)	120.9(4)
N(62)	C(67)	C(66)	116.4(4)

Table 17: Torsion Angles in ° for **AL23001b**.

Atom	Atom	Atom	Atom	Angle/°
Sn(1)	C(3)	C(4)	N(2)	-176.2(3)
Sn(1)	C(3)	C(4)	C(5)	0.1(7)
N(1)	C(6)	C(7)	O(2)	174.3(5)
N(1)	C(6)	C(7)	N(2)	-5.2(7)
N(2)	C(1)	C(2)	C(3)	-0.7(5)
N(2)	C(4)	C(5)	O(1)	177.1(4)
N(2)	C(4)	C(5)	N(1)	-2.0(7)
C(1)	N(2)	C(4)	C(3)	-0.7(6)
C(1)	N(2)	C(4)	C(5)	-177.4(4)
C(1)	N(2)	C(7)	O(2)	2.2(8)
C(1)	N(2)	C(7)	C(6)	-178.3(5)
C(1)	C(2)	C(3)	Sn(1)	176.7(3)
C(1)	C(2)	C(3)	C(4)	0.3(5)
C(2)	C(3)	C(4)	N(2)	0.3(6)
C(2)	C(3)	C(4)	C(5)	176.6(5)
C(3)	C(4)	C(5)	O(1)	0.9(8)
C(3)	C(4)	C(5)	N(1)	-178.1(5)
C(4)	N(2)	C(1)	C(2)	0.8(5)
C(4)	N(2)	C(7)	O(2)	179.0(4)
C(4)	N(2)	C(7)	C(6)	-1.5(7)
C(5)	N(1)	C(6)	C(7)	8.9(8)
C(6)	N(1)	C(5)	O(1)	175.8(5)
C(6)	N(1)	C(5)	C(4)	-5.2(8)
C(7)	N(2)	C(1)	C(2)	178.1(4)
C(7)	N(2)	C(4)	C(3)	-178.0(5)
C(7)	N(2)	C(4)	C(5)	5.3(7)
Sn(21)	C(23)	C(24)	N(22)	177.9(3)
Sn(21)	C(23)	C(24)	C(25)	1.3(7)
N(21)	C(26)	C(27)	O(22)	-175.3(5)
N(21)	C(26)	C(27)	N(22)	5.2(7)
N(22)	C(21)	C(22)	C(23)	0.2(5)
N(22)	C(24)	C(25)	O(21)	-175.7(4)
N(22)	C(24)	C(25)	N(21)	2.1(6)
C(21)	N(22)	C(24)	C(23)	0.1(6)
C(21)	N(22)	C(24)	C(25)	176.9(4)
C(21)	N(22)	C(27)	O(22)	-0.9(7)
C(21)	N(22)	C(27)	C(26)	178.6(5)
C(21)	C(22)	C(23)	Sn(21)	-177.9(3)
C(21)	C(22)	C(23)	C(24)	-0.1(6)
C(22)	C(23)	C(24)	N(22)	0.1(6)
C(22)	C(23)	C(24)	C(25)	-176.4(5)
C(23)	C(24)	C(25)	O(21)	0.7(8)

Atom	Atom	Atom	Atom	Angle/˚
C(23)	C(24)	C(25)	N(21)	178.4(5)
C(24)	N(22)	C(21)	C(22)	-0.1(5)
C(24)	N(22)	C(27)	O(22)	-177.3(4)
C(24)	N(22)	C(27)	C(26)	2.3(7)
C(25)	N(21)	C(26)	C(27)	-9.7(8)
C(26)	N(21)	C(25)	O(21)	-176.4(5)
C(26)	N(21)	C(25)	C(24)	6.0(7)
C(27)	N(22)	C(21)	C(22)	-176.9(4)
C(27)	N(22)	C(24)	C(23)	176.9(4)
C(27)	N(22)	C(24)	C(25)	-6.3(7)
Sn(41)	C(43)	C(44)	N(42)	177.3(3)
Sn(41)	C(43)	C(44)	C(45)	-0.9(8)
N(41)	C(46)	C(47)	O(42)	-175.8(5)
N(41)	C(46)	C(47)	N(42)	3.9(7)
N(42)	C(41)	C(42)	C(43)	1.4(5)
N(42)	C(44)	C(45)	O(41)	-176.6(4)
N(42)	C(44)	C(45)	N(41)	0.3(7)
C(41)	N(42)	C(44)	C(43)	-0.8(6)
C(41)	N(42)	C(44)	C(45)	177.6(4)
C(41)	N(42)	C(47)	O(42)	-0.8(8)
C(41)	N(42)	C(47)	C(46)	179.5(5)
C(41)	C(42)	C(43)	Sn(41)	-177.4(3)
C(41)	C(42)	C(43)	C(44)	-2.0(6)
C(42)	C(43)	C(44)	N(42)	1.7(6)
C(42)	C(43)	C(44)	C(45)	-176.5(5)
C(43)	C(44)	C(45)	O(41)	1.5(8)
C(43)	C(44)	C(45)	N(41)	178.4(5)
C(44)	N(42)	C(41)	C(42)	-0.5(5)
C(44)	N(42)	C(47)	O(42)	-177.7(5)
C(44)	N(42)	C(47)	C(46)	2.7(7)
C(45)	N(41)	C(46)	C(47)	-9.1(8)
C(46)	N(41)	C(45)	O(41)	-176.5(5)
C(46)	N(41)	C(45)	C(44)	6.8(8)
C(47)	N(42)	C(41)	C(42)	-177.8(5)
C(47)	N(42)	C(44)	C(43)	176.5(5)
C(47)	N(42)	C(44)	C(45)	-5.1(7)
Sn(61)	C(63)	C(64)	N(62)	177.1(3)
Sn(61)	C(63)	C(64)	C(65)	0.2(8)
N(61)	C(66)	C(67)	O(62)	-174.5(5)
N(61)	C(66)	C(67)	N(62)	5.4(7)
N(62)	C(61)	C(62)	C(63)	0.1(5)
N(62)	C(64)	C(65)	O(61)	-176.1(5)
N(62)	C(64)	C(65)	N(61)	2.1(7)
C(61)	N(62)	C(64)	C(63)	0.3(6)
C(61)	N(62)	C(64)	C(65)	177.5(4)
C(61)	N(62)	C(67)	O(62)	-2.0(8)
C(61)	N(62)	C(67)	C(66)	178.1(5)
C(61)	C(62)	C(63)	Sn(61)	-177.1(3)
C(61)	C(62)	C(63)	C(64)	0.0(6)
C(62)	C(63)	C(64)	N(62)	-0.2(6)
C(62)	C(63)	C(64)	C(65)	-177.1(5)
C(63)	C(64)	C(65)	O(61)	0.7(8)
C(63)	C(64)	C(65)	N(61)	178.8(5)
C(64)	N(62)	C(61)	C(62)	-0.3(5)
C(64)	N(62)	C(67)	O(62)	-179.2(5)
C(64)	N(62)	C(67)	C(66)	1.0(7)
C(65)	N(61)	C(66)	C(67)	-8.6(8)
C(66)	N(61)	C(65)	O(61)	-177.2(5)
C(66)	N(61)	C(65)	C(64)	4.8(8)
C(67)	N(62)	C(61)	C(62)	-177.8(4)
C(67)	N(62)	C(64)	C(63)	177.8(5)
C(67)	N(62)	C(64)	C(65)	-5.0(7)

Table 18: Hydrogen Fractional Atomic Coordinates (×10[4]) and Equivalent Isotropic Displacement Parameters (Å[2]×10[3]) for **AL23001b**. U_{eq} is defined as 1/3 of the trace of the orthogonalised U_{ij}.

Atom	x	y	z	U_{eq}
H(1A)	5284.2	-1126.06	979.91	44
H(1B)	5127.19	160.4	1362.17	44
H(2A)	6916.7	-589.96	1839.97	47
H(2B)	7061.51	-1884.49	1461.88	47
H(6A)	6499.49	1641.24	-215.76	59
H(6B)	6215.93	2976.81	118.6	59
H(8A)	11218.72	2145.7	1703.91	87
H(8B)	11817.61	1331.65	2174.26	87
H(8C)	10259.58	1855.41	2148.22	87
H(9A)	9708.34	-1496.26	2430.66	98
H(9B)	11087.6	-2214.48	2263.54	98
H(9C)	9627.36	-2688.31	2007.89	98
H(10A)	11292.39	-1517.54	789.97	99
H(10B)	12408.05	-1744.97	1240.94	99
H(10C)	12313.23	-297.07	981.01	99
H(21A)	10343.62	5224.75	1385.3	46
H(21B)	10082.93	3920.06	1008.33	46
H(22A)	8451.38	3155.23	1481.4	50
H(22B)	8708.27	4460.05	1856.76	50
H(26A)	8843.76	7953.38	112.55	54
H(26B)	8435.66	6600.62	-212.71	54
H(28A)	3072.38	4845.47	934.96	93
H(28B)	2932.55	3400.09	1189.36	93
H(28C)	3955.56	3563.52	754.63	93
H(29A)	5533	6662.9	2211.65	106
H(29B)	3955.88	6195.06	2204.34	106
H(29C)	4463.98	7122.05	1773.94	106
H(30A)	5807.75	2126.95	1887.16	98
H(30B)	4598.51	2709.48	2207.62	98
H(30C)	6152	3200.12	2339.67	98
H(41A)	5353.44	5017.69	6350.04	49
H(41B)	4025.05	5186.63	5968.81	49
H(42A)	3290.63	6920.27	6452.87	53
H(42B)	4636.98	6778.2	6825.39	53
H(46A)	8062.24	6208.22	5113.79	56
H(46B)	6708.32	6509.07	4770.39	56
H(48A)	7346.19	10712.39	6771.2	117
H(48B)	6691.14	10257.67	7268.71	117
H(48C)	6492.35	11791.12	7086.71	117
H(49A)	3481.67	9541.37	7353.96	106
H(49B)	2347.56	9513.83	6897.51	106
H(49C)	2814.54	10942.16	7165.52	106
H(50A)	3805.01	11235.92	5714.73	95
H(50B)	4884.17	12305.38	5981.97	95
H(50C)	3343.94	12241.36	6153.57	95
H(61A)	9718.68	-226.07	3601.75	45
H(61B)	11061.66	22.86	3976.26	45
H(62A)	11765.55	1696.66	3495.41	51
H(62B)	10424.28	1450.86	3122.61	51
H(66A)	7090.36	1189.25	4872.76	55
H(66B)	8479.39	1559.13	5196.02	55
H(68A)	12533.98	4147.92	2976.06	94
H(68B)	12114.52	5606.43	2761.11	94
H(68C)	11341.9	4247.18	2542.33	94
H(69A)	8002.77	4710.85	2810.88	92
H(69B)	8471.55	6284.21	2825.66	92

Atom	x	y	z	U_{eq}
H(69C)	7685.04	5694.62	3275.4	92
H(70A)	10236.17	7200.4	3941.71	99
H(70B)	11786.38	6946.95	3798.6	99
H(70C)	11175.81	6112.22	4234.77	99
H(21)	6510(50)	7740(50)	82(19)	44(14)
H(41)	7630(50)	8470(50)	5079(19)	40(15)
H(1)	8480(70)	2610(70)	110(20)	70(20)
H(61)	7410(60)	3470(50)	4890(20)	47(15)

Citations

APEX4

O.V. Dolomanov and L.J. Bourhis and R.J. Gildea and J.A.K. Howard and H. Puschmann, Olex2: A complete structure solution, refinement and analysis program, *J. Appl. Cryst.*, (2009), **42**, 339-341.

SADABS, Bruker axs, Madison, WI (?).

SAINT - Software for the Integration of CCD Detector System Bruker Analytical X-ray Systems, Bruker axs, Madison, WI (?).

Sheldrick, G.M., Crystal structure refinement with ShelXL, *Acta Cryst.*, (2015), **C71**, 3-8.

Sheldrick, G.M., ShelXT-Integrated space-group and crystal-structure determination, *Acta Cryst.*, (2015), **A71**, 3-8.

```
#==============================================================================
# PLATON/CHECK-(260223) versus check.def version 221222, Entry: al23001b
# Data: AL23001b.cif - Type: CIF                Bond Precision   C-C = 0.0066 A
# Refl: AL23001b.fcf - Type: LIST4                                Temp = 200 K
# Audit:OLEX2 1.5-BETA (COMPILED 2023.03.06 SVN.RBB2C1857 FOR OLEXSYS, GUI SVN.R
# Refin:SHELXL 2018/3 (SHELDRICK, 2015)
# X-ray MoKa              R(int) = 0.043,  wR2/R(int) =  2.1,  Nref/Npar =  22.2
# Cell   9.7396(5)   9.7651(4) 27.0075(12)    91.978(1)    93.727(2)   90.119(2)
# Wavelength 0.71073  Volume Reported       2561.6(2) Calculated       2561.7(2)
# SpaceGroup from Symmetry P -1         Hall: -P 1                     triclinic
#                 Reported P -1               -P 1                     triclinic
# MoietyFormula C10 H16 N2 O2 Sn
#      Reported C10 H16 N2 O2 Sn
#    SumFormula C10 H16 N2 O2 Sn
#      Reported C10 H16 N2 O2 Sn
# Mr        =    314.96[Calc],    314.94[Rep]       Volume/NonHatoms = 21 Ang**3
# Dx,gcm-3  =     1.633[Calc],     1.633[Rep]
# Z         =         8[Calc],         8[Rep]
# Mu (mm-1) =     1.980[Calc],     1.980[Rep]  Xtal Size =  0.033x0.382x0.504 mm
# F000      =    1248.0[Calc],    1248.0[Rep]  or F000' =   1243.16[Calc]
# Reported   T Limits: Tmin=0.611              Tmax=0.746  AbsCorr = MULTI-SCAN
# Calculated T Limits: Tmin=0.420 Tmin'=0.354  Tmax=0.937
# Measured   HKL: Reported 115276, Embedded 115322, <Mult>  9.1
# Reported   Hmax= 12, Kmax= 13, Lmax= 35, Nref=  12636        , Th(max)= 28.298
# Obs in FCF Hmax= 12, Kmax= 13, Lmax= 35, Nref=  12636[ 12636], Th(max)= 28.298
# Calculated Hmax= 12, Kmax= 13, Lmax= 36, Nref=  12684        , Ratio  =  0.996
# Reported   Rho(min) = -1.18, Rho(max) = 0.88 e/Ang**3 (From CIF)
# Calculated Rho(min) = -1.08, Rho(max) = 1.12 e/Ang**3 (From CIF+FCF data)
# w=1/[<sup>2</sup>(Fo<sup>2</sup>)+10.3642P], P=(Fo<sup>2</sup>+2Fc<sup>2</sup>)/3
# R= 0.0444( 10261), wR2= 0.0920( 12636), S = 1.168     (From CIF+FCF data)
# R= 0.0444( 10261), wR2= 0.0920( 12636), S = 1.168     (From FCF data only)
# R= 0.0443( 10261), wR2= 0.0920( 12636), S = 1.167, Npar=  569
#==============================================================================
# For Documentation:http://www.platonsoft.nl/CIF-VALIDATION.pdf
#==============================================================================
#                                                                             *
#==============================================================================
#>>> The Following Improvement and Query ALERTS were generated - (Acta-Mode) <<<
#==============================================================================
Format: alert-number_ALERT_alert-type_alert-level text

242_ALERT_2_C Low    'MainMol' Ueq as Compared to Neighbors of        Sn1 Check
242_ALERT_2_C Low    'MainMol' Ueq as Compared to Neighbors of       Sn21 Check
242_ALERT_2_C Low    'MainMol' Ueq as Compared to Neighbors of       Sn41 Check
242_ALERT_2_C Low    'MainMol' Ueq as Compared to Neighbors of       Sn61 Check
906_ALERT_3_C Large K Value in the Analysis of Variance ......      5.165 Check
911_ALERT_3_C Missing FCF Refl Between Thmin & STh/L=    0.600          6 Report
977_ALERT_2_C Check Negative Difference Density on H66B      .      -0.36 eA-3
#==============================================================================
083_ALERT_2_G SHELXL Second Parameter in WGHT  Unusually Large      10.36 Why ?
802_ALERT_4_G CIF Input Record(s) with more than 80 Characters          2 Info
912_ALERT_4_G Missing # of FCF Reflections Above STh/L=  0.600         41 Note
933_ALERT_2_G Number of HKL-OMIT Records in Embedded .res File          5 Note
978_ALERT_2_G Number C-C Bonds with Positive Residual Density.          0 Info
#==============================================================================

ALERT_Level and ALERT_Type Summary
==================================
7 ALERT_Level_C = Check. Ensure it is Not caused by an Omission or Oversight
5 ALERT_Level_G = General Info/Check that it is not Something Unexpected

8 ALERT_Type_2 Indicator that the Structure Model may be Wrong or Deficient.
2 ALERT_Type_3 Indicator that the Structure Quality may be Low.
2 ALERT_Type_4 Improvement, Methodology, Query or Suggestion.
#==============================================================================

0 Missing  Experimental  Info Issue(s) (Out of  65 Tests) -  100 % Satisfied
0 Experimental  Data  Related Issue(s) (Out of  35 Tests) -  100 % Satisfied
8 Structural  Model   Related Issue(s) (Out of 144 Tests) -   94 % Satisfied
```

4 Unresolved or to be Checked Issue(s) (Out of 277 Tests) -   99 % Satisfied

4.4. X-ray Crystal Structure Data of Bis-Diketopiperazine 14: CCDC number 2426513

Submitted by: **Adam Lockyer**
The University of Edinburgh

Solved by: **Gary S Nichol**

Sample ID: **AL-6082-Fr2**

Compound AL-6082-Fr2 crystallized as very fine, flexible, colourless needles. Fomblin oil was used to coat a selection of crystals which were then mounted on MiTeGen kapton loops and frozen in liquid nitrogen. The loops were stored in a MiTeGen Unipuck and transported to Diamond Light Source. Data were collected remotely at beam line I-19 of Diamond Light Source (award CY35994), yielding structure AL24015. Please cite Johnson NT, Waddell PG, Clegg W, Probert MR, Remote access revolution: chemical crystallographers enter a new era at Diamond Light Source beamline I19. Crystals, 2017, 7, (12), 360 in any publication.

Crystal Data and Experimental

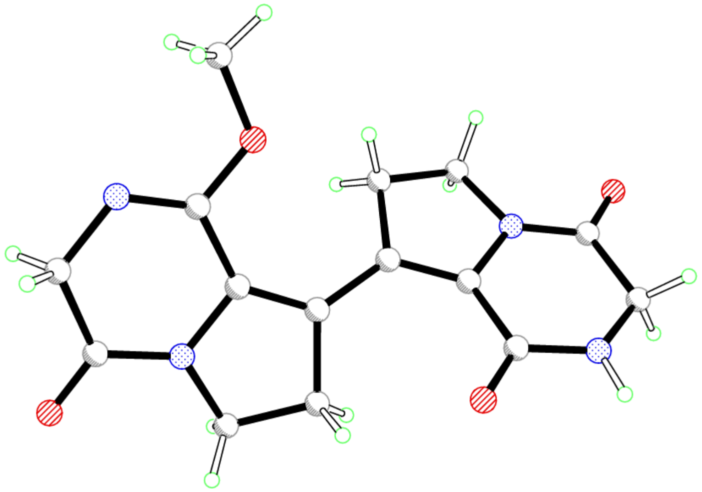

Experimental. Single colourless needle-shaped crystals of **AL24015_xia** recrystallised from hot methanol by slow cooling. A suitable crystal with dimensions 0.10 × 0.02 × 0.01 mm^3 was selected and mounted on a mitegen tip in Paratone oil. on a Diamond Light Source I-19 diffractometer. The crystal was kept at a steady T = 100.00 K during data collection. The structure was solved with the ShelXS (Sheldrick, 2008) solution program using direct methods and by using Olex2 1.5-beta (Dolomanov et al., 2009) as the graphical interface. The model was refined with ShelXL 2019/3 (Sheldrick, 2015) using full matrix least squares minimisation on $\boldsymbol{F}^2$.

Crystal Data. $C_{15}H_{16}N_4O_4$, M_r = 316.32, triclinic, P-1 (No. 2), a = 4.07290(16) Å, b = 9.2147(3) Å, c = 18.7073(7) Å, α = 78.519(3)°, β = 86.169(3)°, γ = 79.135(3)°, V = 675.40(4) Å^3, T = 100.00 K, Z = 2, Z' = 1, μ(synchrotron) = 0.109, 10667 reflections measured, 3351 unique (R_{int} = 0.0770) which were used in all calculations. The final wR_2 was 0.2710 (all data) and R_1 was 0.1099 (I≥2 σ(I)).

**Compound**	**AL24015_xia**
Formula	$C_{15}H_{16}N_4O_4$
$D_{calc.}$/ g cm^{-3}	1.555
μ/mm^{-1}	0.109
Formula Weight	316.32
Colour	colourless
Shape	needle-shaped
Size/mm^3	0.10×0.02×0.01
T/K	100.00
Crystal System	triclinic
Space Group	P-1
a/Å	4.07290(16)
b/Å	9.2147(3)
c/Å	18.7073(7)
α/°	78.519(3)
β/°	86.169(3)
γ/°	79.135(3)
V/Å^3	675.40(4)
Z	2
Z'	1
Wavelength/Å	0.6889
Radiation type	synchrotron
Θ_{min}/°	1.077
Θ_{max}/°	27.365
Measured Refl's.	10667
Indep't Refl's	3351
Refl's I≥2 σ(I)	2168
R_{int}	0.0770
Parameters	213
Restraints	0
Largest Peak	1.134
Deepest Hole	-0.505
GooF	0.986
wR_2 (all data)	0.2710
wR_2	0.2665
R_1 (all data)	0.1209
R_1	0.1099

Structure Quality Indicators

**Reflections:**	d min (0.6889) 2Θ=54.7° 0.75	I/σ(I) 11.1	Rint m=3.18 7.70%	Full 48.8° 99% to 54.7° 99.6
**Refinement:**	Shift 0.000	Max Peak 1.1	Min Peak -0.5	GooF 0.986

A colourless needle-shaped-shaped crystal with dimensions 0.10 × 0.02 × 0.01 mm^3 was mounted on a mitegen tip in Paratone oil.. Data were collected using a Diamond Light Source I-19 diffractometer equipped with an Oxford Cryosystems Cryostream 700+ low-temperature device operating at T = 100.00 K.

Data were measured using shutterless scans with synchrotron radiation. The diffraction pattern was indexed and the total number of runs and images was based on the strategy calculation from the program GDA - generic data acquisition software. The maximum resolution that was achieved was Θ = 27.365° (0.75 Å).

The unit cell was refined using Allan, D. R. et al. (2017) Crystals, 7(11), 336.DIALS 3.21.1.1-g807743295-release (Winter, G. et al., 2018)dials.scale (Beilsten-Edmands, J. et al., 2020)XIA2 3.21.1 (Winter, G., 2010) on 3467 reflections, 33% of the observed reflections.

Data reduction, scaling and absorption corrections were performed using Allan, D. R. et al. (2017) Crystals, 7(11), 336.DIALS 3.21.1.1-g807743295-release (Winter, G. et al., 2018)dials.scale (Beilsten-Edmands, J. et al., 2020)XIA2 3.21.1 (Winter, G., 2010). The final completeness is 99.60 % out to 27.365° in Θ. A multi-scan absorption correction was performed using DIALS 3.21.1.1-g807743295-release Scaling & analysis of unmerged intensities, absorption correction using spherical harmonics. The absorption coefficient μ of this material is 0.109 mm^{-1} at this wavelength (λ = 0.68890Å) and the minimum and maximum transmissions are 0.997 and 1.000.

The structure was solved and the space group *P*-1 (# 2) determined by the ShelXS (Sheldrick, 2008) structure solution program using using direct methods and refined by full matrix least squares minimisation on $\boldsymbol{F}^2$ using version 2019/3 of ShelXL 2019/3 (Sheldrick, 2015). All non-hydrogen atoms were refined anisotropically. Hydrogen atom positions were calculated geometrically and refined using the riding model. Most hydrogen atom positions were calculated geometrically and refined using the riding model, but some hydrogen atoms were refined freely.

_refine_special_details: The compound crystallized as very long, thin, flexible needles which did not cleanly extinguish polarized light. Some streaking between the Bragg peaks was observed, as were peaks corresponding to a second orientation of the lattice, but data processing as a "twin" brings no improvement to the refinement. Nevertheless the structure is chemically unambiguous.

_exptl_absorpt_process_details: DIALS 3.21.1.1-g807743295-releaseScaling & analysis of unmerged intensities, absorption correction using spherical harmonics

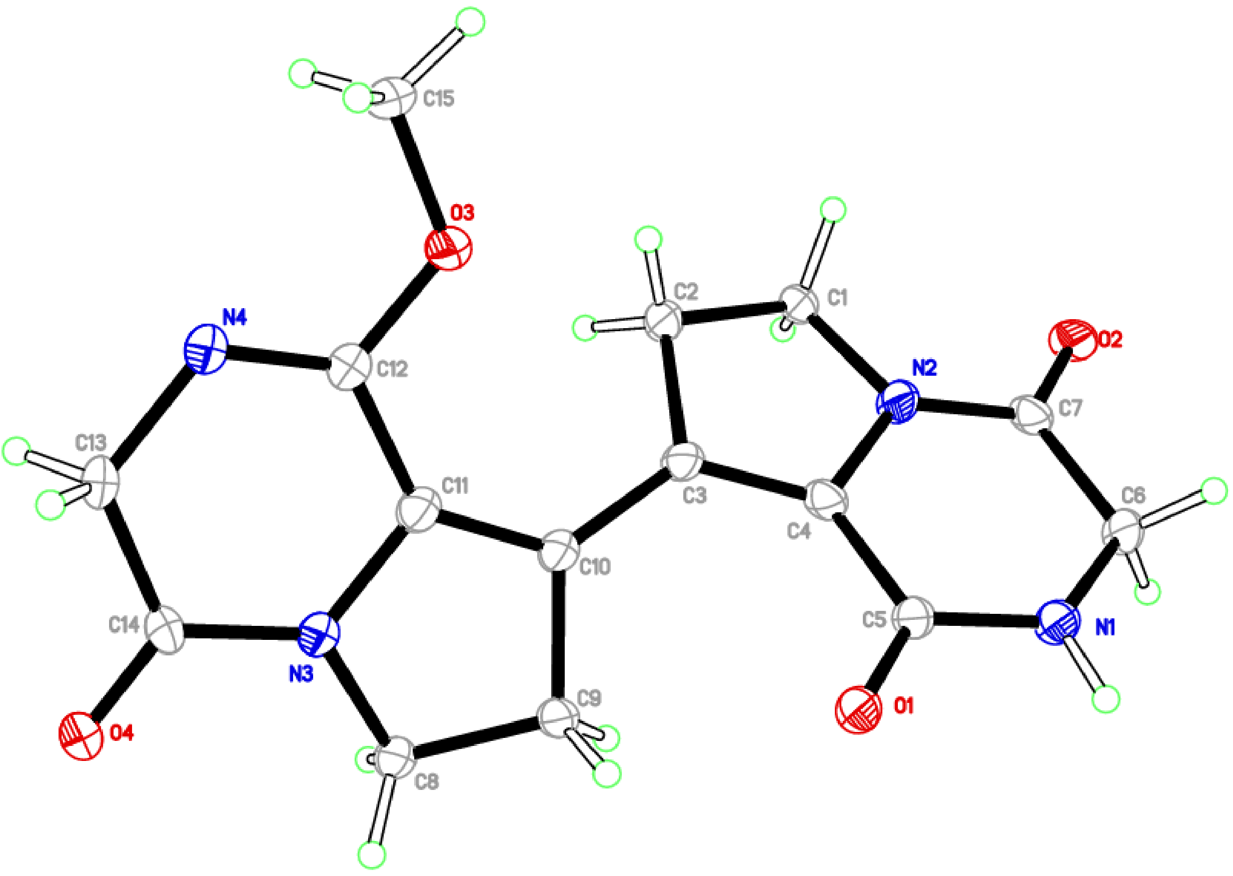

Figure 4 The molecular structure of AL24015. Displacement ellipsoids are at the 50% probablity level.

Reflection Statistics

Total reflections (after filtering)	10667	Unique reflections	3351
Completeness	0.993	Mean I/σ	6.68
hkl$_{max}$ collected	(5, 12, 24)	hkl$_{min}$ collected	(-5, -12, -24)
hkl$_{max}$ used	(5, 12, 24)	hkl$_{min}$ used	(-5, -11, 0)
Lim d$_{max}$ collected	100.0	Lim d$_{min}$ collected	0.34
d$_{max}$ used	18.32	d$_{min}$ used	0.75
Friedel pairs	2888	Friedel pairs merged	1
Inconsistent equivalents	9	R$_{int}$	0.077
R$_{sigma}$	0.0903	Intensity transformed	0
Omitted reflections	0	Omitted by user (OMIT hkl)	0
Multiplicity	(3055, 2194, 765, 199, 23, 3)	Maximum multiplicity	8
Removed systematic absences	0	Filtered off (Shel/OMIT)	0

Table 19: Fractional Atomic Coordinates (×10^4) and Equivalent Isotropic Displacement Parameters (Å^2×10^3) for **AL24015_xia**. U_{eq} is defined as 1/3 of the trace of the orthogonalised U_{ij}.

Atom	x	y	z	U_{eq}
O1	8718(6)	-927(2)	847.6(12)	20.4(5)
O2	671(6)	4372(2)	992.4(13)	23.9(6)
O3	7212(6)	-1366(3)	3758.5(12)	22.8(6)
O4	5699(6)	-6900(3)	3466.1(13)	25.7(6)
N1	6682(7)	1471(3)	315.6(15)	20.4(6)
N2	3032(7)	2021(3)	1539.8(15)	19.2(6)
N3	5866(7)	-4469(3)	2956.4(14)	19.5(6)
N4	7840(7)	-3871(3)	4262.1(15)	22.1(6)

Atom	x	y	z	U_{eq}
C1	1103(8)	2059(3)	2229.7(17)	18.2(7)
C2	2578(9)	565(3)	2714.5(17)	20.9(7)
C3	4502(8)	-375(3)	2186.8(17)	17.9(7)
C4	4800(8)	548(3)	1529.4(17)	17.2(6)
C5	6841(8)	291(3)	873.6(16)	17.6(6)
C6	4514(9)	2928(3)	269.7(17)	20.8(7)
C7	2572(8)	3180(3)	959.6(17)	18.4(7)
C8	4483(8)	-4488(4)	2251.5(17)	19.8(7)
C9	4986(8)	-2974(3)	1795.0(17)	19.4(7)
C10	5328(8)	-2019(3)	2357.7(17)	17.8(6)
C11	6010(8)	-2970(4)	3009.1(17)	19.0(7)
C12	7049(8)	-2799(4)	3720.9(17)	19.5(7)
C13	7607(9)	-5404(4)	4204.8(18)	23.9(7)
C14	6299(8)	-5668(3)	3515.5(17)	20.1(7)
C15	8351(9)	-1116(4)	4425.1(18)	24.6(7)

Table 20: Anisotropic Displacement Parameters (×10[4]) for **AL24015_xia.** The anisotropic displacement factor exponent takes the form: $-2\pi^2[h^2a^{*2} \times U_{11} + ... +2hka^* \times b^* \times U_{12}]$

Atom	U_{11}	U_{22}	U_{33}	U_{23}	U_{13}	U_{12}
O1	28.2(12)	12.9(11)	16.2(11)	-1.3(8)	0.9(9)	3.7(9)
O2	37.7(14)	10.5(11)	20.1(12)	-3.9(9)	-3.3(10)	6.1(10)
O3	36.4(14)	16.1(12)	16.6(12)	-2.5(9)	-6.8(9)	-5.4(10)
O4	43.8(15)	14.3(12)	18.5(12)	-1.6(9)	-2.9(10)	-5.0(10)
N1	29.2(15)	13.2(13)	15.5(13)	-1.6(10)	0.3(11)	2.8(11)
N2	26.1(14)	14.2(13)	14.5(13)	-1.4(10)	-1.6(10)	2.1(10)
N3	28.7(15)	15.5(13)	13.3(13)	-1.4(10)	-2.6(10)	-2.3(11)
N4	33.1(16)	16.6(14)	15.4(13)	-0.5(10)	-3.7(11)	-3.3(12)
C1	26.5(16)	10.4(14)	16.0(15)	-2.7(11)	0.6(12)	0.3(12)
C2	33.7(18)	11.6(14)	14.8(15)	-2.0(11)	0.1(12)	1.5(13)
C3	25.7(16)	13.7(15)	12.9(14)	-2.8(11)	-3.1(11)	1.4(12)
C4	21.4(15)	11.2(14)	17.7(15)	-3.0(11)	-3.7(11)	1.4(11)
C5	24.9(16)	12.6(14)	13.9(14)	-1.3(11)	-3.5(11)	-0.1(12)
C6	32.0(17)	11.4(14)	14.6(15)	1.6(11)	-2.0(12)	3.0(12)
C7	25.4(16)	10.8(14)	18.5(16)	-3.1(11)	-4.6(12)	0.1(12)
C8	28.9(17)	15.4(15)	13.8(15)	-2.0(11)	-5.4(12)	0.5(12)
C9	30.3(17)	12.3(14)	13.9(14)	-2.3(11)	-4.4(12)	1.7(12)
C10	23.9(16)	15.5(15)	12.9(14)	-1.7(11)	-1.4(11)	-1.2(12)
C11	23.4(16)	17.8(16)	14.4(15)	-2.4(12)	-1.8(11)	-0.5(12)
C12	25.4(16)	18.1(16)	14.6(15)	-2.0(12)	-1.2(12)	-4.3(13)
C13	39(2)	15.4(16)	15.4(15)	2.5(12)	-4.4(13)	-3.4(13)
C14	28.4(17)	11.8(14)	18.0(16)	-0.6(12)	-0.4(12)	-0.9(12)
C15	40(2)	19.6(16)	15.4(15)	-5.1(12)	-6.7(13)	-5.5(14)

Table 21: Bond Lengths in Å for **AL24015_xia**.

Atom	Atom	Length/Å
O1	C5	1.241(4)
O2	C7	1.229(4)
O3	C12	1.350(4)
O3	C15	1.434(4)
O4	C14	1.227(4)
N1	C5	1.344(4)
N1	C6	1.451(4)
N2	C1	1.468(4)
N2	C4	1.414(4)

Atom	Atom	Length/Å
N2	C7	1.358(4)
N3	C8	1.473(4)
N3	C11	1.417(4)
N3	C14	1.355(4)
N4	C12	1.277(4)
N4	C13	1.459(4)
C1	C2	1.530(4)
C2	C3	1.523(4)
C3	C4	1.361(4)

Atom	Atom	Length/Å	Atom	Atom	Length/Å
C3	C10	1.463(4)	C9	C10	1.528(4)
C4	C5	1.469(4)	C10	C11	1.363(4)
C6	C7	1.503(4)	C11	C12	1.470(4)
C8	C9	1.526(4)	C13	C14	1.507(5)

Table 22: Bond Angles in ° for **AL24015_xia**.

Atom	Atom	Atom	Angle/°	Atom	Atom	Atom	Angle/°
C12	O3	C15	117.0(3)	N1	C6	C7	115.2(2)
C5	N1	C6	127.0(3)	O2	C7	N2	122.2(3)
C4	N2	C1	110.2(2)	O2	C7	C6	121.5(3)
C7	N2	C1	122.4(3)	N2	C7	C6	116.4(3)
C7	N2	C4	126.1(3)	N3	C8	C9	102.6(3)
C11	N3	C8	109.8(2)	C8	C9	C10	104.3(2)
C14	N3	C8	123.8(3)	C3	C10	C9	121.5(3)
C14	N3	C11	125.2(3)	C11	C10	C3	130.1(3)
C12	N4	C13	119.5(3)	C11	C10	C9	107.7(3)
N2	C1	C2	103.3(2)	N3	C11	C12	114.5(3)
C3	C2	C1	104.6(2)	C10	C11	N3	110.8(3)
C4	C3	C2	108.3(3)	C10	C11	C12	134.5(3)
C4	C3	C10	127.8(3)	O3	C12	C11	113.7(3)
C10	C3	C2	123.2(3)	N4	C12	O3	120.9(3)
N2	C4	C5	117.9(3)	N4	C12	C11	125.3(3)
C3	C4	N2	111.0(3)	N4	C13	C14	118.8(3)
C3	C4	C5	130.9(3)	O4	C14	N3	122.5(3)
O1	C5	N1	121.8(3)	O4	C14	C13	121.7(3)
O1	C5	C4	121.9(3)	N3	C14	C13	115.9(3)
N1	C5	C4	116.3(3)				

Table 23: Torsion Angles in ° for **AL24015_xia**.

Atom	Atom	Atom	Atom	Angle/°
N1	C6	C7	O2	179.4(3)
N1	C6	C7	N2	-0.7(4)
N2	C1	C2	C3	-15.9(3)
N2	C4	C5	O1	172.9(3)
N2	C4	C5	N1	-4.1(4)
N3	C8	C9	C10	-21.3(3)
N3	C11	C12	O3	179.2(3)
N3	C11	C12	N4	1.2(5)
N4	C13	C14	O4	-171.8(3)
N4	C13	C14	N3	8.8(4)
C1	N2	C4	C3	-6.5(4)
C1	N2	C4	C5	179.2(3)
C1	N2	C7	O2	4.9(5)
C1	N2	C7	C6	-174.9(3)
C1	C2	C3	C4	13.0(4)
C1	C2	C3	C10	-158.0(3)
C2	C3	C4	N2	-4.5(4)
C2	C3	C4	C5	168.9(3)
C2	C3	C10	C9	137.6(3)
C2	C3	C10	C11	-32.1(5)
C3	C4	C5	O1	-0.1(6)
C3	C4	C5	N1	-177.1(3)
C3	C10	C11	N3	165.6(3)
C3	C10	C11	C12	-19.2(6)
C4	N2	C1	C2	14.1(3)

Atom	Atom	Atom	Atom	Angle/°
C4	N2	C7	O2	171.0(3)
C4	N2	C7	C6	-8.9(5)
C4	C3	C10	C9	-31.5(5)
C4	C3	C10	C11	158.7(3)
C5	N1	C6	C7	8.0(5)
C6	N1	C5	O1	177.6(3)
C6	N1	C5	C4	-5.4(5)
C7	N2	C1	C2	-177.8(3)
C7	N2	C4	C3	-173.9(3)
C7	N2	C4	C5	11.7(5)
C8	N3	C11	C10	-9.3(4)
C8	N3	C11	C12	174.4(3)
C8	N3	C14	O4	3.3(5)
C8	N3	C14	C13	-177.4(3)
C8	C9	C10	C3	-154.8(3)
C8	C9	C10	C11	17.0(3)
C9	C10	C11	N3	-5.3(4)
C9	C10	C11	C12	170.0(3)
C10	C3	C4	N2	166.0(3)
C10	C3	C4	C5	-20.6(6)
C10	C11	C12	O3	4.1(5)
C10	C11	C12	N4	-174.0(3)
C11	N3	C8	C9	19.3(3)
C11	N3	C14	O4	169.7(3)
C11	N3	C14	C13	-10.9(5)
C12	N4	C13	C14	-2.5(5)
C13	N4	C12	O3	179.5(3)
C13	N4	C12	C11	-2.6(5)
C14	N3	C8	C9	-172.4(3)
C14	N3	C11	C10	-177.4(3)
C14	N3	C11	C12	6.3(4)
C15	O3	C12	N4	0.9(5)
C15	O3	C12	C11	-177.3(3)

Table 24: Hydrogen Fractional Atomic Coordinates (×10^4) and Equivalent Isotropic Displacement Parameters (Å^2×10^3) for **AL24015_xia**. U_{eq} is defined as 1/3 of the trace of the orthogonalised U_{ij}.

Atom	x	y	z	U_{eq}
H1	8180(120)	1180(50)	-40(30)	41(12)
H1A	-1309.48	2120.73	2158.72	22
H1B	1409.5	2924.54	2442.29	22
H2A	4090.32	726	3072.96	25
H2B	782.96	66.95	2980.52	25
H6A	2910.08	3048.8	-122.05	25
H6B	5896.66	3720.5	125.09	25
H8A	2083	-4563.91	2305.95	24
H8B	5717.6	-5333.67	2032.5	24
H9A	3042.18	-2512.27	1485.11	23
H9B	7029.9	-3091.23	1478.82	23
H13A	6164.12	-5796.71	4617.26	29
H13B	9869.35	-6023.69	4275.59	29
H15A	8244.54	-34.59	4396.91	37
H15B	10664.15	-1640.88	4500.09	37
H15C	6921.81	-1501.16	4834.12	37

Citations

Allan, D. R. et al. (2017) Crystals, 7(11), 336. DIALS 3.21.1.1-g807743295-release (Winter, G. et al., 2018) dials.scale (Beilsten-Edmands, J. et al., 2020) XIA2 3.21.1 (Winter, G., 2010)

GDA - generic data acquisition software

O.V. Dolomanov and L.J. Bourhis and R.J. Gildea and J.A.K. Howard and H. Puschmann, Olex2: A complete structure solution, refinement and analysis program, *J. Appl. Cryst.*, (2009), **42**, 339-341.

Sheldrick, G.M., A short history of ShelX, *Acta Cryst.*, (2008), **A64**, 339-341.

Sheldrick, G.M., Crystal structure refinement with ShelXL, *Acta Cryst.*, (2015), **C71**, 3-8.

```
#==============================================================================
# PLATON/CHECK-(250424) versus check.def version 240202, Entry: al24015_xia
# Data: AL24015_xia.cif - Type: CIF              Bond Precision   C-C = 0.0043 A
# Refl: AL24015_xia.fcf - Type: LIST4                                Temp = 100 K
# Audit:OLEX2 1.5-BETA (COMPILED 2024.02.16 SVN.R378C4104 FOR OLEXSYS, GUI SVN.R
# Refin:SHELXL 2019/3 (SHELDRICK, 2015)
# X-ray Synchrotron       R(int) = 0.077,  wR2/R(int) =  3.5,  Nref/Npar =  15.7
# Cell 4.07290(16)   9.2147(3)  18.7073(7)    78.519(3)    86.169(3)   79.135(3)
# Wavelength 0.68890  Volume Reported       675.40(4) Calculated       675.40(4)
# SpaceGroup from Symmetry P -1        Hall: -P 1                     triclinic
#                 Reported P -1              -P 1                     triclinic
# MoietyFormula C15 H16 N4 O4
#      Reported C15 H16 N4 O4
#    SumFormula C15 H16 N4 O4
#      Reported C15 H16 N4 O4
# Mr        =    316.32[Calc],    316.32[Rep]       Volume/NonHatoms = 15 Ang**3
# Dx,gcm-3  =     1.555[Calc],     1.555[Rep]
# Z         =         2[Calc],         2[Rep]
# Mu (mm-1) =     0.107[Calc],     0.109[Rep]  Xtal Size =  0.010x0.023x0.102 mm
# F000      =     332.0[Calc],     332.0[Rep]  or F000' =    332.14[Calc]
# Reported   T Limits: Tmin=0.997              Tmax=1.000  AbsCorr = MULTI-SCAN
# Calculated T Limits: Tmin=0.997 Tmin'=0.989  Tmax=0.999
# Measured   HKL: Reported  10667, Embedded  10667, <Mult>  3.2
# Reported   Hmax=  5, Kmax= 12, Lmax= 24, Nref=   3351        , Th(max)= 27.365
# Obs in FCF Hmax=  5, Kmax= 12, Lmax= 24, Nref=   3351[  3351], Th(max)= 27.365
# Expected   Hmax=  5, Kmax= 12, Lmax= 24, Nref=   3375        , Ratio  =  0.993
# Reported   Rho(min) = -0.50, Rho(max) = 1.13 e/Ang**3 (From CIF)
# Calculated Rho(min) = -0.50, Rho(max) = 1.15 e/Ang**3 (From CIF+FCF data)
# w=1/[<sup>2</sup>(Fo<sup>2</sup>)+(0.1852P)<sup>2</sup>],
P=(Fo<sup>2</sup>+2Fc<sup>2</sup>)/3
# R= 0.1100(  2168), wR2= 0.2710(  3351), S = 0.986     (From CIF+FCF data)
# R= 0.1099(  2168), wR2= 0.2710(  3351), S = 0.986     (From FCF data only)
# R= 0.1099(  2168), wR2= 0.2710(  3351), S = 0.986, Npar=  213
#==============================================================================
# For Documentation:http://www.platonsoft.nl/CIF-VALIDATION.pdf
#==============================================================================
#                                                                             *
#==============================================================================
#>>> The Following Improvement and Query ALERTS were generated - (Acta-Mode) <<<
#==============================================================================
Format: alert-number_ALERT_alert-type_alert-level text

097_ALERT_2_B Large Reported Max.  (Positive) Residual Density       1.13 eA-3
#==============================================================================
082_ALERT_2_C High R1 Value .................................       0.11 Report
084_ALERT_3_C High wR2 Value (i.e. > 0.25) ..................       0.27 Report
094_ALERT_2_C Ratio of Maximum / Minimum Residual Density ....       2.25 Report
250_ALERT_2_C Large U3/U1 Ratio for <U(i,j)> Tensor(Resd    1)        2.4 Note
340_ALERT_3_C Low Bond Precision on  C-C Bonds ..............    0.00427 Ang.
790_ALERT_4_C Centre of Gravity not Within Unit Cell: Resd.  #          1 Note
C15 H16 N4 O4
911_ALERT_3_C Missing FCF Refl Between Thmin & STh/L=    0.600         10 Report
-3  7  0,  -1  8  0,  -1-10  1,  -2  7  3,   2  8  4,   2  9  5,
-2 -5  8,   3  8  8,  -2  7 11,   1 -5 14,
975_ALERT_2_C Check Calcd Resid. Dens.  1.07Ang From O4      .       1.13 eA-3
975_ALERT_2_C Check Calcd Resid. Dens.  1.07Ang From O2      .       0.99 eA-3
975_ALERT_2_C Check Calcd Resid. Dens.  1.06Ang From O1      .       0.99 eA-3
975_ALERT_2_C Check Calcd Resid. Dens.  1.10Ang From N1      .       0.83 eA-3
#==============================================================================
066_ALERT_1_G Predicted and Reported Tmin&Tmax Range Identical          ? Check
072_ALERT_2_G SHELXL First  Parameter in WGHT  Unusually Large       0.19 Report
092_ALERT_4_G Check: Wavelength Given is not Cu,Ga,Mo,Ag,In Ka    0.68890 Ang.
154_ALERT_1_G The s.u.'s on the Cell Angles are Equal ..(Note)      0.003 Degree
802_ALERT_4_G CIF Input Record(s) with more than 80 Characters          2 Info
912_ALERT_4_G Missing # of FCF Reflections Above STh/L=  0.600         14 Note
941_ALERT_3_G Average HKL Measurement Multiplicity ..........        3.2 Low
969_ALERT_5_G The 'Henn et al.' R-Factor-gap value ..........       2.77 Note
Predicted wR2: Based on SigI**2  9.79 or SHELX Weight 28.41
978_ALERT_2_G Number C-C Bonds with Positive Residual Density.          0 Info
```

```
#==============================================================================
#                                                                             *
#==============================================================================
ALERT_Level and ALERT_Type Summary
==================================
1 ALERT_Level_B = A Potentially Serious Problem - Consider Carefully
11 ALERT_Level_C = Check. Ensure it is Not caused by an Omission or Oversight
9 ALERT_Level_G = General Info/Check that it is not Something Unexpected

2 ALERT_Type_1 CIF Construction/Syntax Error, Inconsistent or Missing Data.
10 ALERT_Type_2 Indicator that the Structure Model may be Wrong or Deficient.
4 ALERT_Type_3 Indicator that the Structure Quality may be Low.
4 ALERT_Type_4 Improvement, Methodology, Query or Suggestion.
1 ALERT_Type_5 Informative Message, Check.
#==============================================================================

0 Missing  Experimental  Info Issue(s) (Out of  65 Tests) -  100 % Satisfied
1 Experimental  Data  Related Issue(s) (Out of  35 Tests) -   97 % Satisfied
11 Structural  Model   Related Issue(s) (Out of 144 Tests) -   92 % Satisfied
8 Unresolved or to be Checked Issue(s) (Out of 281 Tests) -   97 % Satisfied

#                                                                             *
```

4.5. X-ray Crystal Structure Data of Bis-Lactim Ether 16: CCDC number 2426512

Submitted by: **Adam Lockyer**

The University of Edinburgh

Solved by: **Gary S Nichol**

Sample ID: **AL-5076-Fr1-Fr2**

Compound AL-5076-Fr1-Fr2 was provided as crystals suitable for single crystal X-ray diffraction, yielding structure AL24004.

Crystal Data and Experimental

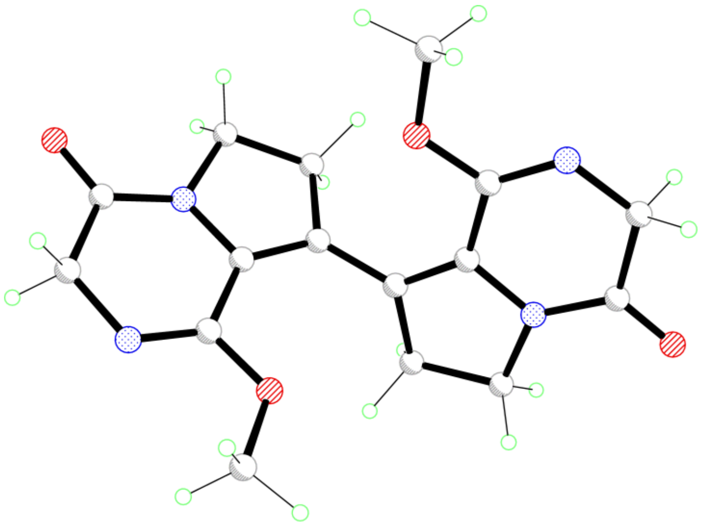

Experimental. Single clear colourless block-shaped crystals of **AL24004** recrystallised from dichloromethane by slow evaporation. A suitable crystal with dimensions 0.12 × 0.10 × 0.07 mm^3 was selected and mounted on a MITIGEN holder in perfluoroether oil on a Rigaku Oxford Diffraction SuperNova diffractometer. The crystal was kept at a steady T = 120.00(10) K during data collection. The structure was solved with the ShelXT 2018/2 (Sheldrick, 2018) solution program using dual methods and by using Olex2 1.5-beta (Dolomanov et al., 2009) as the graphical interface. The model was refined with olex2.refine 1.5-beta (Bourhis et al., 2015) using full matrix least squares minimisation on $\boldsymbol{F}^2$.

Crystal Data. $C_{16}H_{18}N_4O_4$, M_r = 330.346, monoclinic, $P2_1/n$ (No. 14), a = 12.0665(2) Å, b = 7.8998(1) Å, c = 16.6591(3) Å, β = 106.566(2)°, α = γ = 90°, V = 1522.08(5) Å^3, T = 120.00(10) K, Z = 4, Z' = 1, μ(Cu K$_\alpha$) = 0.882, 26671 reflections measured, 3169 unique (R_{int} = 0.0321) which were used in all calculations. The final wR_2 was 0.0469 (all data) and R_1 was 0.0186 (I≥2 σ(I)).

**Compound**	**AL24004**
Formula	$C_{16}H_{18}N_4O_4$
$D_{calc.}$/ g cm^{-3}	1.442
μ/mm^{-1}	0.882
Formula Weight	330.346
Colour	clear colourless
Shape	block-shaped
Size/mm^3	0.12×0.10×0.07
T/K	120.00(10)
Crystal System	monoclinic
Space Group	$P2_1/n$
a/Å	12.0665(2)
b/Å	7.8998(1)
c/Å	16.6591(3)
α/°	90
β/°	106.566(2)
γ/°	90
V/Å^3	1522.08(5)
Z	4
Z'	1
Wavelength/Å	1.54184
Radiation type	Cu K$_\alpha$
Θ_{min}/°	4.03
Θ_{max}/°	76.05
Measured Refl's.	26671
Indep't Refl's	3169
Refl's I≥2 σ(I)	2972
R_{int}	0.0321
Parameters	379
Restraints	0
Largest Peak	0.1046
Deepest Hole	-0.1361
GooF	1.0903
wR_2 (all data)	0.0469
wR_2	0.0460
R_1 (all data)	0.0206
R_1	0.0186

Structure Quality Indicators

**Reflections:**	d min (CuKα) 2Θ=152.1° 0.79	I/σ(I) 67.8	Rint m=8.77 3.21%	Full 135.4° 100
**Refinement:**	Shift 0.001	Max Peak 0.1	Min Peak -0.1	GooF 1.090

A clear colourless block-shaped-shaped crystal with dimensions 0.12 × 0.10 × 0.07 mm^3 was mounted on a MITIGEN holder in perfluoroether oil. Data were collected using a Rigaku Oxford Diffraction SuperNova diffractometer equipped with an Oxford Cryosystems Cryostream 700+ low-temperature device operating at T = 120.00(10) K.

Data were measured using ω scans with Cu K_α radiation. The diffraction pattern was indexed and the total number of runs and images was based on the strategy calculation from the program CrysAlisPro 1.171.42.81a (Rigaku OD, 2023). The maximum resolution that was achieved was Θ = 76.05° (0.79 Å).

The unit cell was refined using CrysAlisPro 1.171.42.81a (Rigaku OD, 2023) on 15418 reflections, 58% of the observed reflections.

Data reduction, scaling and absorption corrections were performed using CrysAlisPro 1.171.42.81a (Rigaku OD, 2023). The final completeness is 100.00 % out to 76.05° in Θ. A multi-scan absorption correction was performed using CrysAlisPro 1.171.42.81a (Rigaku Oxford Diffraction, 2023) Empirical absorption correction using spherical harmonics, implemented in SCALE3 ABSPACK scaling algorithm.. The absorption coefficient μ of this material is 0.882 mm^{-1} at this wavelength (λ = 1.54184Å) and the minimum and maximum transmissions are 0.843 and 1.000.

The structure was solved and the space group $P2_1/n$ (# 14) determined by the ShelXT 2018/2 (Sheldrick, 2018) structure solution program using using dual methods and refined by full matrix least squares minimisation on $\boldsymbol{F}^2$ using version of olex2.refine 1.5-beta (Bourhis et al., 2015). All non-hydrogen atoms were refined anisotropically. Hydrogen atom positions were calculated geometrically and refined using the riding model.

_refine_special_details: NoSpherA2 refinement

_olex2_refine_details: Refinement using NoSpherA2, an implementation ofNOn-SPHERical Atom-form-factors in Olex2.Please cite:F. Kleemiss et al. Chem. Sci. DOI 10.1039/D0SC05526C - 2021NoSpherA2 implementation of HAR makes use oftailor-made aspherical atomic form factors calculatedon-the-fly from a Hirshfeld-partitioned electron density (ED) - not fromspherical-atom form factors.The ED is calculated from a gaussian basis set single determinant SCFwavefunction - either Hartree-Fock or DFT using selected funtionals - for a fragment of the crystal.This fragment can be embedded in an electrostatic crystal field by employing cluster chargesor modelled using implicit solvation models, depending on the software used.The following options were used: SOFTWARE: ORCA 5.0 PARTITIONING: NoSpherA2 INT ACCURACY: Normal METHOD: R2SCAN BASIS SET: cc-pVTZ CHARGE: 0 MULTIPLICITY: 1 DATE: 2024-01-30_18-10-15

_exptl_absorpt_process_details: CrysAlisPro 1.171.42.81a (Rigaku Oxford Diffraction, 2023) using spherical harmonics,implemented in SCALE3 ABSPACK scaling algorithm.

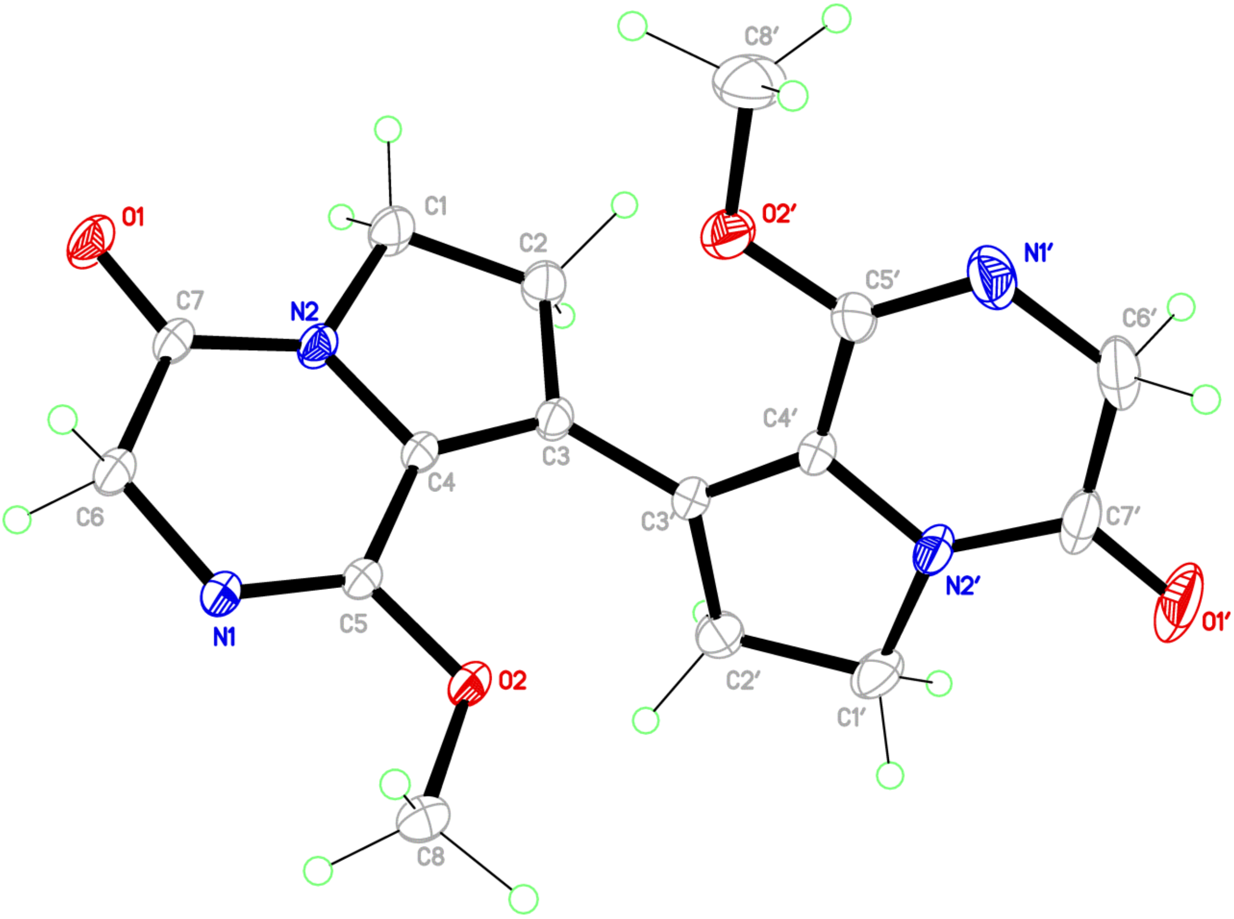

Figure 5 The molecular structure of AL24004. Displacement ellipsoids are at the 50% probability level.

Data Plots: Diffraction Data

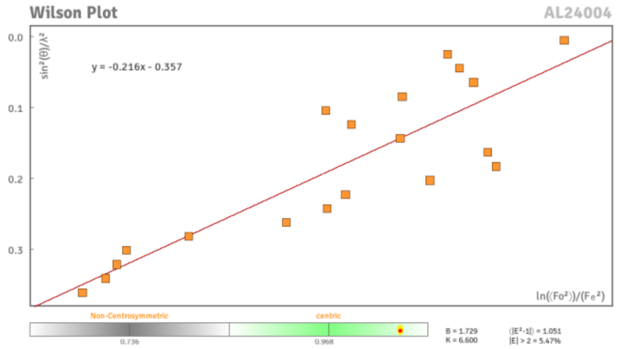

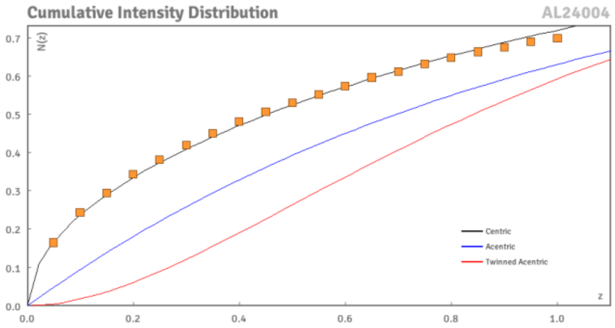

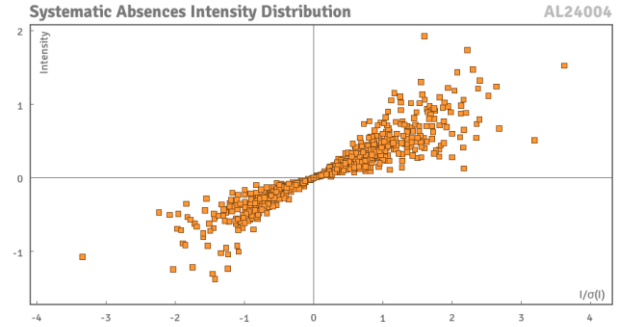

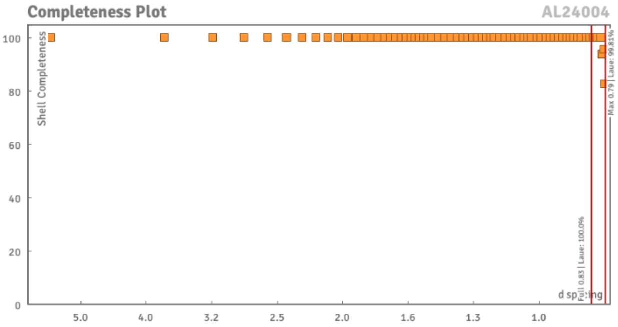

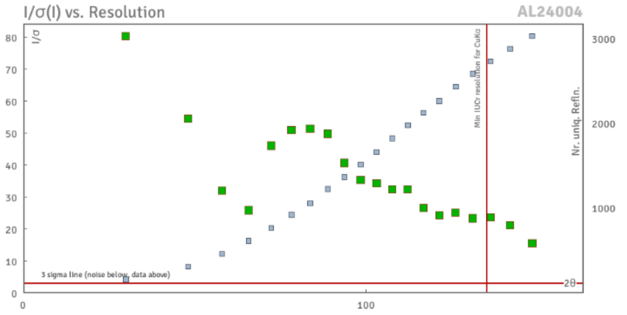

Data Plots: Refinement and Data

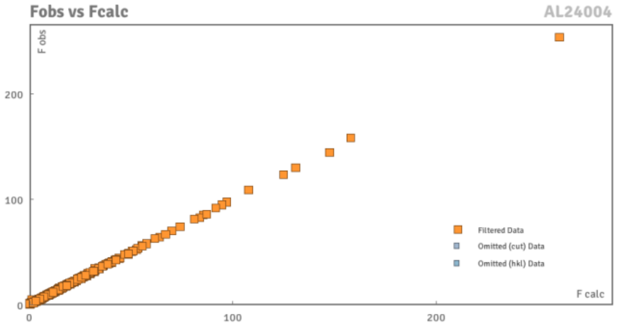

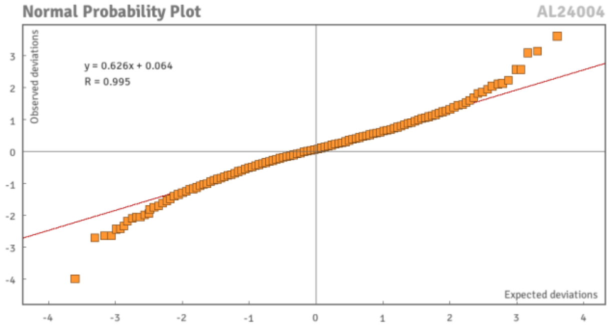

Reflection Statistics

Total reflections (after filtering)	27786	Unique reflections	3169
Completeness	0.998	Mean I/σ	36.33
hkl_{max} collected	(15, 9, 20)	hkl_{min} collected	(-15, -9, -18)
hkl_{max} used	(14, 9, 20)	hkl_{min} used	(-15, 0, 0)
Lim d_{max} collected	100.0	Lim d_{min} collected	0.77
d_{max} used	11.57	d_{min} used	0.79
Friedel pairs	4280	Friedel pairs merged	1
Inconsistent equivalents	0	R_{int}	0.0321
R_{sigma}	0.0148	Intensity transformed	0
Omitted reflections	0	Omitted by user (OMIT hkl)	0
Multiplicity	(2852, 3028, 1944, 1272, 821, 359, 134, 43, 33, 12)	Maximum multiplicity	22
Removed systematic absences	1115	Filtered off (Shel/OMIT)	0

Table 25: Fractional Atomic Coordinates (×10^4) and Equivalent Isotropic Displacement Parameters (Å^2×10^3) for **AL24004**. U_{eq} is defined as 1/3 of the trace of the orthogonalised U_{ij}.

Atom	x	y	z	U_{eq}
O1	3000.5(4)	7610.0(6)	1995.8(3)	25.00(11)
O1'	10380.7(5)	2066.6(8)	5908.1(4)	41.85(15)
O2	6611.2(4)	8067.8(5)	4770.9(3)	18.54(10)
O2'	7619.9(4)	4453.9(7)	3000.7(3)	24.87(11)
N1	5133.0(4)	9451.7(7)	3828.2(3)	17.86(11)
N1'	9338.9(5)	3148.0(8)	3663.5(4)	26.86(13)
N2	4430.0(4)	6364.0(6)	3000.3(3)	16.60(11)
N2'	8759.6(5)	3426.3(7)	5183.8(3)	23.01(12)
C1	4309.9(6)	4662.3(8)	2629.7(4)	22.58(14)
C1'	8265.5(6)	3738.8(10)	5879.2(4)	27.28(15)
C2	5233.8(6)	3621.9(8)	3259.5(4)	21.80(14)
C2'	7017.8(6)	4310.6(9)	5447.0(4)	23.39(14)
C3	5931.4(5)	4922.2(8)	3866.7(4)	15.83(12)
C3'	6969.7(5)	4460.7(7)	4530.7(4)	16.63(12)

Atom	x	y	z	U_{eq}
C4	5411.6(5)	6438.2(7)	3701.0(3)	14.34(12)
C4'	7976.6(5)	3929.5(8)	4424.4(4)	17.74(12)
C5	5689.5(5)	8096.8(8)	4092.1(4)	14.73(12)
C5'	8381.1(5)	3802.3(8)	3678.8(4)	20.55(13)
C6	4177.4(6)	9399.6(8)	3067.5(4)	21.51(14)
C6'	10074.2(6)	2394.1(10)	4426.5(5)	30.41(16)
C7	3812.8(5)	7713.9(8)	2638.1(4)	17.56(12)
C7'	9762.4(6)	2615.4(9)	5243.0(5)	27.93(16)
C8	6988.5(6)	9671.0(9)	5150.2(4)	23.29(14)
C8'	7887.4(7)	4307.9(13)	2220.7(5)	33.03(17)

Table 26: Anisotropic Displacement Parameters (×10[4]) for **AL24004.** The anisotropic displacement factor exponent takes the form: $-2\pi^2[h^2a^{*2} \times U_{11}+ \ldots +2hka^* \times b^* \times U_{12}]$

Atom	U_{11}	U_{22}	U_{33}	U_{23}	U_{13}	U_{12}
O1	19.4(2)	26.7(2)	20.9(2)	1.20(18)	-7.11(18)	-0.73(19)
O1'	28.2(3)	41.2(3)	43.5(3)	12.3(2)	-10.1(2)	10.6(3)
O2	15.80(19)	19.6(2)	15.8(2)	1.85(16)	-2.62(16)	-1.05(16)
O2'	19.5(2)	37.8(3)	17.1(2)	3.39(19)	4.87(17)	-0.56(19)
N1	17.7(2)	17.2(3)	15.1(2)	3.26(19)	-1.10(19)	-0.30(19)
N1'	17.2(2)	29.5(3)	34.8(3)	3.4(2)	8.8(2)	-4.1(2)
N2	12.2(2)	18.3(3)	16.2(2)	0.19(18)	-0.95(19)	-0.19(19)
N2'	19.3(3)	23.1(3)	20.8(3)	6.0(2)	-3.7(2)	0.3(2)
C1	18.1(3)	20.2(3)	23.7(3)	-1.8(2)	-3.4(3)	-4.1(3)
H1a	18(5)	31(6)	87(8)	-6(4)	-8(5)	-6(6)
H1b	73(8)	43(6)	20(5)	8(6)	9(5)	-1(4)
C1'	31.4(4)	28.8(4)	16.3(3)	4.4(3)	-1.8(3)	3.8(3)
H1'a	51(7)	59(7)	36(6)	-4(6)	0(5)	-14(6)
H1'b	66(8)	46(7)	35(6)	17(6)	7(5)	19(5)
C2	19.3(3)	16.8(3)	25.2(3)	-0.5(2)	-0.4(3)	-2.6(3)
H2a	36(6)	43(6)	47(6)	9(5)	0(5)	-25(5)
H2b	48(6)	36(6)	48(6)	-17(5)	2(5)	7(5)
C2'	28.0(3)	25.6(3)	16.4(3)	5.9(3)	6.0(3)	2.8(3)
H2'a	58(7)	40(6)	33(5)	27(5)	12(5)	-4(5)
H2'b	34(5)	59(7)	39(6)	0(5)	14(5)	7(5)
C3	12.9(2)	16.6(3)	16.2(3)	1.2(2)	1.3(2)	0.3(2)
C3'	15.6(3)	18.0(3)	14.7(3)	3.4(2)	1.8(2)	0.8(2)
C4	11.6(2)	16.0(3)	13.6(3)	0.5(2)	0.8(2)	-0.2(2)
C4'	14.5(3)	19.7(3)	16.4(3)	3.4(2)	0.3(2)	-0.3(2)
C5	12.8(2)	16.2(3)	13.1(3)	0.8(2)	0.5(2)	-0.1(2)
C5'	14.8(3)	24.3(3)	22.1(3)	1.9(2)	4.4(2)	-3.3(2)
C6	20.9(3)	20.5(3)	17.2(3)	4.3(2)	-4.0(2)	-0.4(2)
H6a	25(5)	47(6)	46(6)	13(5)	-9(5)	-17(5)
H6b	61(7)	45(6)	27(5)	-13(5)	-1(5)	18(5)
C6'	16.6(3)	25.8(4)	46.6(4)	5.8(3)	5.4(3)	-0.8(3)
H6'a	22(6)	58(7)	74(8)	0(5)	14(5)	9(6)
H6'b	64(8)	29(6)	67(7)	9(5)	20(6)	-5(5)
C7	13.5(2)	20.8(3)	15.2(3)	1.3(2)	-1.0(2)	0.6(2)
C7'	17.9(3)	22.9(3)	35.0(4)	4.6(2)	-5.3(3)	2.5(3)
C8	20.9(3)	23.0(3)	20.9(3)	0.0(3)	-2.2(3)	-5.2(3)
H8a	44(6)	47(7)	40(6)	9(5)	-19(5)	-9(5)
H8b	29(5)	58(7)	53(7)	16(5)	6(5)	-14(6)
H8c	69(8)	45(6)	33(5)	-21(6)	7(5)	-1(5)
C8'	32.5(4)	46.8(5)	23.0(4)	-2.1(4)	13.2(3)	-3.1(3)
H8'a	85(9)	40(7)	66(8)	6(6)	40(7)	-4(6)
H8'b	45(7)	74(8)	70(8)	-17(6)	23(6)	-14(7)
H8'c	51(7)	121(12)	26(5)	32(7)	5(5)	25(6)

Table 27: Bond Lengths in Å for **AL24004**.

Atom	Atom	Length/Å
O1	C7	1.2306(7)
O1'	C7'	1.2251(9)
O2	C5	1.3413(7)
O2	C8	1.4305(8)
O2'	C5'	1.3390(8)
O2'	C8'	1.4301(9)
N1	C5	1.2733(8)
N1	C6	1.4505(7)
N1'	C5'	1.2730(8)
N1'	C6'	1.4541(10)
N2	C1	1.4691(8)
N2	C4	1.4073(7)
N2	C7	1.3416(8)
N2'	C1'	1.4670(10)

Atom	Atom	Length/Å
N2'	C4'	1.4035(7)
N2'	C7'	1.3476(9)
C1	C2	1.5336(9)
C1'	C2'	1.5399(9)
C2	C3	1.5171(8)
C2'	C3'	1.5155(9)
C3	C3'	1.4615(8)
C3	C4	1.3435(8)
C3'	C4'	1.3440(8)
C4	C5	1.4592(8)
C4'	C5'	1.4617(9)
C6	C7	1.5162(9)
C6'	C7'	1.5213(12)

Table 28: Bond Angles in ° for **AL24004**.

Atom	Atom	Atom	Angle/°
C8	O2	C5	116.12(5)
C8'	O2'	C5'	117.21(6)
C6	N1	C5	119.17(5)
C6'	N1'	C5'	118.69(6)
C4	N2	C1	110.22(5)
C7	N2	C1	124.25(5)
C7	N2	C4	124.61(5)
C4'	N2'	C1'	110.09(5)
C7'	N2'	C1'	125.51(6)
C7'	N2'	C4'	124.07(6)
C2	C1	N2	103.74(5)
C2'	C1'	N2'	104.09(5)
C3	C2	C1	104.49(5)
C3'	C2'	C1'	104.21(5)
C3'	C3	C2	121.92(5)
C4	C3	C2	109.19(5)
C4	C3	C3'	128.84(5)
C3	C3'	C2'	124.07(5)
C4'	C3'	C2'	109.39(5)
C4'	C3'	C3	126.17(6)

Atom	Atom	Atom	Angle/°
C3	C4	N2	111.50(5)
C5	C4	N2	116.08(5)
C5	C4	C3	132.40(5)
C3'	C4'	N2'	111.78(6)
C5'	C4'	N2'	116.39(5)
C5'	C4'	C3'	131.83(6)
N1	C5	O2	122.30(5)
C4	C5	O2	113.01(5)
C4	C5	N1	124.69(5)
N1'	C5'	O2'	123.20(6)
C4'	C5'	O2'	112.05(5)
C4'	C5'	N1'	124.75(6)
C7	C6	N1	119.24(5)
C7'	C6'	N1'	119.32(6)
N2	C7	O1	122.71(6)
C6	C7	O1	121.39(6)
C6	C7	N2	115.90(5)
N2'	C7'	O1'	122.48(8)
C6'	C7'	O1'	121.73(7)
C6'	C7'	N2'	115.79(6)

Table 29: Torsion Angles in ° for **AL24004**.

Atom	Atom	Atom	Atom	Angle/°
O1	C7	N2	C1	7.66(8)
O1	C7	N2	C4	175.53(6)
O1	C7	C6	N1	179.36(6)
O1'	C7'	N2'	C1'	-1.02(9)
O1'	C7'	N2'	C4'	-173.69(7)
O1'	C7'	C6'	N1'	-176.83(7)
O2	C5	N1	C6	177.18(6)
O2	C5	C4	N2	177.99(5)
O2	C5	C4	C3	-3.72(7)
O2'	C5'	N1'	C6'	-176.70(6)
O2'	C5'	C4'	N2'	-174.22(5)
O2'	C5'	C4'	C3'	5.34(7)
N1	C5	C4	N2	-2.25(7)

Atom	Atom	Atom	Atom	Angle/°
N1	C5	C4	C3	176.04(6)
N1	C6	C7	N2	-0.81(7)
N1'	C5'	C4'	N2'	6.03(8)
N1'	C5'	C4'	C3'	-174.41(6)
N1'	C6'	C7'	N2'	4.14(8)
N2	C1	C2	C3	-9.07(6)
N2	C4	C3	C2	-2.93(6)
N2	C4	C3	C3'	179.61(5)
N2'	C1'	C2'	C3'	-6.39(6)
N2'	C4'	C3'	C2'	-0.78(6)
N2'	C4'	C3'	C3	-173.94(5)
C1	C2	C3	C3'	-174.67(5)
C1	C2	C3	C4	7.66(6)
C1'	C2'	C3'	C3	177.94(5)
C1'	C2'	C3'	C4'	4.60(6)
C2	C3	C3'	C2'	-104.58(7)
C2	C3	C3'	C4'	67.63(7)
C2	C3	C4	C5	178.72(5)
C2'	C3'	C3	C4	72.59(7)
C2'	C3'	C4'	C5'	179.65(5)
C3	C3'	C4'	C5'	6.49(8)

Table 30: Hydrogen Fractional Atomic Coordinates (×10^4) and Equivalent Isotropic Displacement Parameters (Å^2×10^3) for **AL24004.** U_{eq} is defined as 1/3 of the trace of the orthogonalised U_{ij}.

Atom	x	y	z	U_{eq}
H1a	3442(8)	4187(12)	2549(7)	50(3)
H1b	4491(10)	4720(13)	2023(6)	46(3)
H1'a	8772(9)	4719(14)	6283(6)	51(3)
H1'b	8322(10)	2581(14)	6254(6)	51(3)
H2a	5791(9)	2903(13)	2952(6)	45(3)
H2b	4852(9)	2712(13)	3606(6)	47(3)
H2'a	6813(9)	5521(13)	5691(6)	44(3)
H2'b	6384(8)	3377(14)	5523(6)	43(3)
H6a	3407(8)	9983(13)	3182(6)	43(3)
H6b	4405(10)	10214(13)	2596(6)	47(3)
H6'a	10964(8)	2876(14)	4535(7)	51(3)
H6'b	10116(10)	1041(13)	4328(7)	53(3)
H8a	7664(9)	9373(13)	5721(6)	50(3)
H8b	6275(8)	10343(14)	5285(6)	48(3)
H8c	7296(10)	10424(13)	4728(6)	51(3)
H8'a	8087(11)	3013(14)	2120(7)	60(3)
H8'b	8632(10)	5059(16)	2250(7)	62(3)
H8'c	7129(10)	4782(18)	1747(6)	67(4)

Citations

CrysAlisPro Software System, Rigaku Oxford Diffraction, (2023).

L.J. Bourhis and O.V. Dolomanov and R.J. Gildea and J.A.K. Howard and H. Puschmann, The Anatomy of a Comprehensive Constrained, Restrained, Refinement Program for the Modern Computing Environment - Olex2 Disected, *Acta Cryst. A*, (2015), **A71**, 59-71.

O.V. Dolomanov and L.J. Bourhis and R.J. Gildea and J.A.K. Howard and H. Puschmann, Olex2: A complete structure solution, refinement and analysis program, *J. Appl. Cryst.*, (2009), **42**, 339-341.

Sheldrick, G.M., ShelXT-Integrated space-group and crystal-structure determination, *Acta Cryst.*, (2015), **A71**, 3-8.

```
#==============================================================================
# PLATON/CHECK-(120923) versus check.def version 230825, Entry: AL24004
# Data: AL24004.cif - Type: CIF                  Bond Precision   C-C = 0.0009 A
# Refl: AL24004.fcf - Type: LIST4                                  Temp = 120 K
# Audit:OLEX2 1.5-BETA (COMPILED 2023.08.24 SVN.RE1EC1418 FOR OLEXSYS, GUI SVN.R
# Refin:OLEX2.REFINE 1.5-BETA (BOURHIS ET AL., 2015)
# X-ray CuKa             R(int) = 0.032,  wR2/R(int) =  1.5,  Nref/Npar =   8.4
# Cell  12.0665(2)   7.8998(1)  16.6591(3)          90   106.566(2)          90
# Wavelength 1.54184  Volume Reported      1522.08(5) Calculated      1522.08(4)
# SpaceGroup from Symmetry P 21/n       Hall: -P 2yn                 monoclinic
#                Reported P 1 21/n 1         -P 2yn                 monoclinic
# MoietyFormula C16 H18 N4 O4
#      Reported C16 H18 N4 O4
#    SumFormula C16 H18 N4 O4
#      Reported C16 H18 N4 O4
# Mr        =    330.34[Calc],    330.35[Rep]       Volume/NonHatoms = 16 Ang**3
# Dx,gcm-3  =     1.442[Calc],     1.442[Rep]
# Z         =         4[Calc],         4[Rep]
# Mu (mm-1) =     0.883[Calc],     0.882[Rep]  Xtal Size =  0.066x0.101x0.122 mm
# F000      =     696.0[Calc],     698.6[Rep]  or F000' =    698.29[Calc]
# Reported   T Limits: Tmin=0.843              Tmax=1.000  AbsCorr = MULTI-SCAN
# Calculated T Limits: Tmin=0.899 Tmin'=0.898  Tmax=0.943
# Measured   HKL: Reported  26671, Embedded      0, <Mult>  0.0
# Reported   Hmax= 15, Kmax=  9, Lmax= 20, Nref=   3169        , Th(max)= 76.050
# Obs in FCF Hmax= 15, Kmax=  9, Lmax= 20, Nref=   3169[  3169], Th(max)= 76.054
# Expected   Hmax= 15, Kmax=  9, Lmax= 20, Nref=   3174        , Ratio  =  0.998
# Reported   Rho(min) = -0.14, Rho(max) = 0.10 e/Ang**3 (From CIF)
# Calculated Rho(min) = -0.20, Rho(max) = 0.55 e/Ang**3 (From CIF+FCF data)
# w=1/[<sup>2</sup>(Fo<sup>2</sup>)+(0.0218P)<sup>2</sup>+0.1002P],
P=(Fo<sup>2</sup>+2Fc<sup>2</sup>)/3
# R= 0.0459(  2972), wR2= 0.1117(  3169), S = 2.597     (From CIF+FCF data)
# R= 0.0186(  2972), wR2= 0.0469(  3169), S = 1.090     (From FCF data only)
# R= 0.0186(  2972), wR2= 0.0469(  3169), S = 1.090, Npar=  379
#==============================================================================
# For Documentation:http://www.platonsoft.nl/CIF-VALIDATION.pdf
#==============================================================================
#                                                                             *
#==============================================================================
#>>> The Following Improvement and Query ALERTS were generated - (Acta-Mode) <<<
#==============================================================================
Format: alert-number_ALERT_alert-type_alert-level text

088_ALERT_3_C Poor Data / Parameter Ratio ....................       8.36 Note
351_ALERT_3_C Long   C-H (X0.96,N1.08A)  C2       - H2A      .       1.11 Ang.
351_ALERT_3_C Long   C-H (X0.96,N1.08A)  C6       - H6B      .       1.11 Ang.
411_ALERT_2_C Short Inter H...H Contact  H1'A     ..H6B      .       2.10 Ang.
1/2+x,3/2-y,1/2+z  =      4_676 Check
#==============================================================================
068_ALERT_1_G Reported F000 Differs from Calcd (or Missing)...     Please Check
720_ALERT_4_G Number of Unusual/Non-Standard Labels ..........          9 Note
H1'A    H1'B    H2'A    H2'B    H6'A    H6'B    H8'A    H8'B
H8'C
802_ALERT_4_G CIF Input Record(s) with more than 80 Characters          1 Info
912_ALERT_4_G Missing # of FCF Reflections Above STh/L=  0.600          6 Note
960_ALERT_3_G Number of Intensities with I  <   - 2*sig(I) ...          1 Check
978_ALERT_2_G Number C-C Bonds with Positive Residual Density.          8 Info
979_ALERT_1_G NoSpherA2 Scattering Factors Used .............     Please Note
#==============================================================================

ALERT_Level and ALERT_Type Summary
==================================
4 ALERT_Level_C = Check. Ensure it is Not caused by an Omission or Oversight
7 ALERT_Level_G = General Info/Check that it is not Something Unexpected

2 ALERT_Type_1 CIF Construction/Syntax Error, Inconsistent or Missing Data.
2 ALERT_Type_2 Indicator that the Structure Model may be Wrong or Deficient.
4 ALERT_Type_3 Indicator that the Structure Quality may be Low.
3 ALERT_Type_4 Improvement, Methodology, Query or Suggestion.
#==============================================================================
```

```
0 Missing  Experimental  Info Issue(s) (Out of  65 Tests) -  100 % Satisfied
0 Experimental  Data  Related Issue(s) (Out of  35 Tests) -  100 % Satisfied
4 Structural  Model   Related Issue(s) (Out of 144 Tests) -   97 % Satisfied
7 Unresolved or to be Checked Issue(s) (Out of 279 Tests) -   97 % Satisfied

#                                                                              *
```

4.6. X-ray Crystal Structure Data of β-Lactam 9: CCDC number 2426514

Submitted by: **Adam Lockyer**

Solved by: **Gary S Nichol**

Sample ID: **AL-014-Fr1**

Compound AL-014-Fr1 was provided as crystals suitable for single crystal X-ray diffraction, yielding structure AL22001.

Crystal Data and Experimental

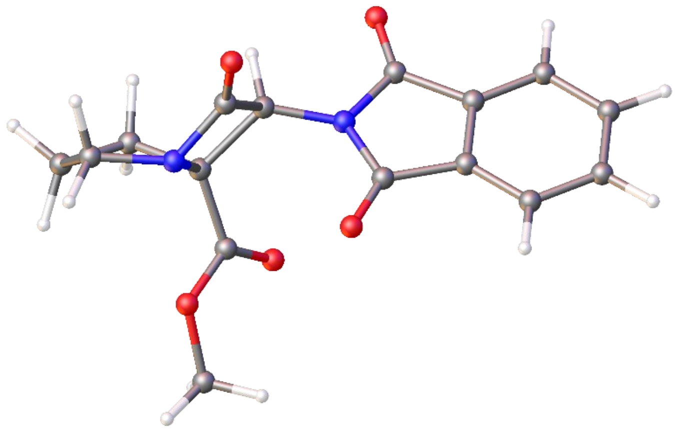

Experimental. Single colourless prism-shaped crystals of **AL22001** recrystallised from diethyl ether by slow evaporation. A suitable crystal with dimensions 0.19 × 0.14 × 0.11 mm^3 was selected and mounted on a MITIGEN holder in Paratone oil on a Rigaku Oxford Diffraction XCalibur diffractometer. The crystal was kept at a steady T = 120.01(10) K during data collection. The structure was solved with the **ShelXT** 2018/2 (Sheldrick, 2018) solution program using dual methods and by using **Olex2** 1.5-beta (Dolomanov et al., 2009) as the graphical interface. The model was refined with **olex2.refine** 1.5-beta (Bourhis et al., 2015) using full matrix least squares minimisation on $\boldsymbol{F}^2$.

Crystal Data. $C_{16}H_{14}N_2O_5$, M_r = 314.300, monoclinic, $C2/c$ (No. 15), a = 20.0240(4) Å, b = 9.8782(3) Å, c = 14.8097(3) Å, β = 98.623(2)°, α = γ = 90°, V = 2896.26(12) Å^3, T = 120.01(10) K, Z = 8, Z' = 1, μ(Mo K_α) = 0.109, 45005 reflections measured, 5076 unique (R_{int} = 0.0494) which were used in all calculations. The final wR_2 was 0.0521 (all data) and R_1 was 0.0360 (I≥2 σ(I)).

**Compound**	**AL22001**
Formula	$C_{16}H_{14}N_2O_5$
$D_{calc.}$/ g cm^{-3}	1.442
μ/mm^{-1}	0.109
Formula Weight	314.300
Colour	colourless
Shape	prism-shaped
Size/mm^3	0.19×0.14×0.11
T/K	120.01(10)
Crystal System	monoclinic
Space Group	$C2/c$
a/Å	20.0240(4)
b/Å	9.8782(3)
c/Å	14.8097(3)
α/°	90
β/°	98.623(2)
γ/°	90
V/Å^3	2896.26(12)
Z	8
Z'	1
Wavelength/Å	0.71073
Radiation type	Mo K_α
Θ_{min}/°	3.49
Θ_{max}/°	32.84
Measured Refl's.	45005
Indep't Refl's	5076
Refl's I≥2 σ(I)	4229
R_{int}	0.0494
Parameters	334
Restraints	0
Largest Peak	0.2849
Deepest Hole	-0.2801
GooF	1.1022
wR_2 (all data)	0.0521
wR_2	0.0484
R_1 (all data)	0.0518
R_1	0.0360

Structure Quality Indicators

**Reflections:**	d min (Mo) 2Θ=65.7° 0.66	I/σ(I) 30.4	Rint 4.94%	Full 50.5° 94% to 65.7° 99.8
**Refinement:**	Shift -0.001	Max Peak 0.3	Min Peak -0.3	GooF 1.102

A colourless prism-shaped-shaped crystal with dimensions 0.19 × 0.14 × 0.11 mm^3 was mounted on a MITIGEN holder in Paratone oil. Data were collected using a Rigaku Oxford Diffraction XCalibur diffractometer equipped with an Oxford Cryosystems Cryostream 700+ low-temperature device operating at T = 120.01(10) K.

Data were measured using ω scans with Mo K_α radiation. The diffraction pattern was indexed and the total number of runs and images was based on the strategy calculation from the program CrysAlisPro 1.171.41.123a (Rigaku OD, 2022). The maximum resolution that was achieved was Θ = 32.84° (0.66 Å).

The unit cell was refined using CrysAlisPro 1.171.41.123a (Rigaku OD, 2022) on 10831 reflections, 24% of the observed reflections.

Data reduction, scaling and absorption corrections were performed using CrysAlisPro 1.171.41.123a (Rigaku OD, 2022). The final completeness is 99.77 % out to 32.84° in Θ. A multi-scan absorption correction was performed using CrysAlisPro 1.171.41.123a (Rigaku Oxford Diffraction, 2022) Empirical absorption correction using spherical harmonics, implemented in SCALE3 ABSPACK scaling algorithm.. The absorption coefficient μ of this material is 0.109 mm^{-1} at this wavelength (λ = 0.71073Å) and the minimum and maximum transmissions are 0.984 and 1.000.

The structure was solved and the space group $C2/c$ (# 15) determined by the ShelXT 2018/2 (Sheldrick, 2018) structure solution program using using dual methods and refined by full matrix least squares minimisation on $\boldsymbol{F}^2$ using version of **olex2.refine** 1.5-beta (Bourhis et al., 2015). All non-hydrogen atoms were refined anisotropically. Hydrogen atom positions were calculated geometrically and refined using the riding model.

_refine_special_details: Refinement using NoSpherA2, an implementation ofNOn-SPHERical Atom-form-factors in Olex2.Please cite:F. Kleemiss et al. Chem. Sci. DOI 10.1039/D0SC05526C - 2021NoSpherA2 implementation of HAR makes use oftailor-made aspherical atomic form factors calculatedon-the-fly from a Hirshfeld-partitioned electron density (ED) - not fromspherical-atom form factors.The ED is calculated from a gaussian basis set single determinant SCFwavefunction - either Hartree-Fock or DFT using selected funtionals - for a fragment of the crystal.This fragment can be embedded in an electrostatic crystal field by employing cluster chargesor modelled using implicit solvation models, depending on the software used.The following options were used: SOFTWARE: ORCA PARTITIONING: NoSpherA2 INT ACCURACY: Normal METHOD: PBE BASIS SET: cc-pVTZ CHARGE: 0 MULTIPLICITY: 1 DATE: 2022-04-28_17-23-26

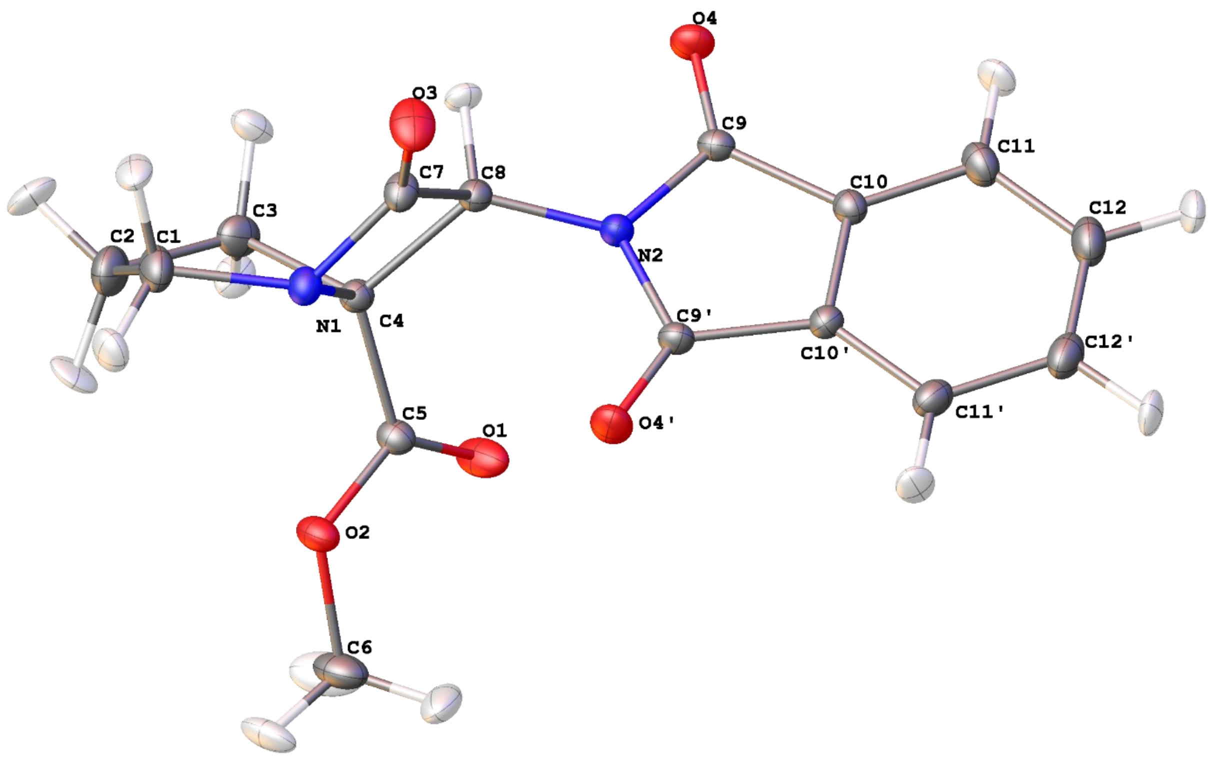

Figure 6: The molecular structure of AL22001. Displacement ellipsoids are at the 50% probability level.

Data Plots: Diffraction Data

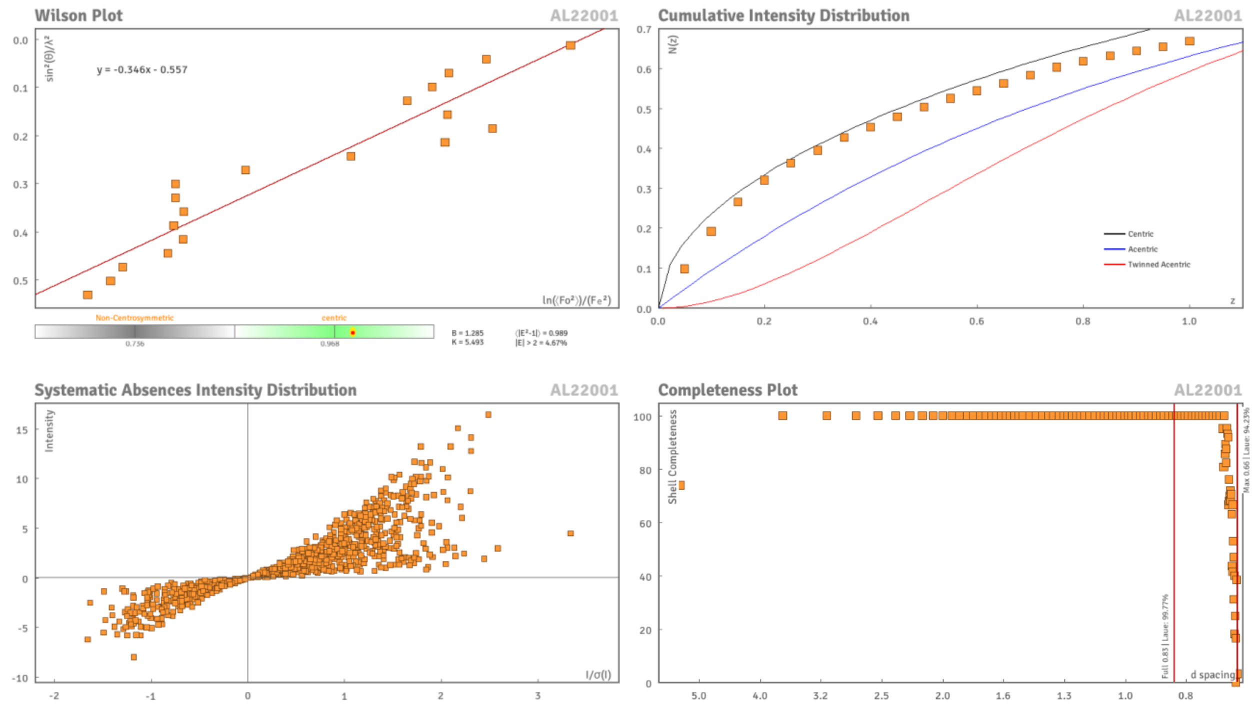

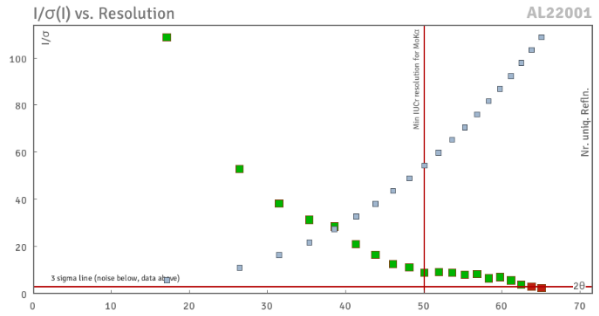

Data Plots: Refinement and Data

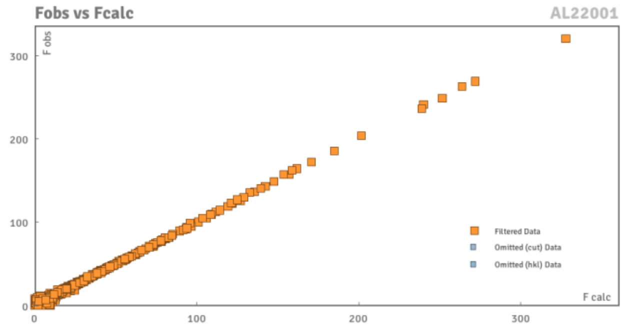

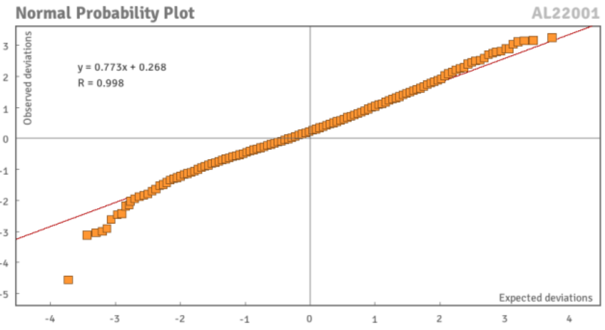

Reflection Statistics

Total reflections (after filtering)	46373	Unique reflections	5076
Completeness	0.942	Mean I/σ	20.58
hkl_{max} collected	(30, 14, 21)	hkl_{min} collected	(-29, -14, -22)
hkl_{max} used	(29, 14, 22)	hkl_{min} used	(-30, 0, 0)
Lim d_{max} collected	100.0	Lim d_{min} collected	0.36
d_{max} used	5.83	d_{min} used	0.66
Friedel pairs	9150	Friedel pairs merged	1
Inconsistent equivalents	1	R_{int}	0.0494
R_{sigma}	0.0329	Intensity transformed	0
Omitted reflections	0	Omitted by user (OMIT hkl)	0
Multiplicity	(5202, 5787, 4550, 2266, 860, 226, 87, 37, 21, 11, 1, 1)	Maximum multiplicity	38
Removed systematic absences	1368	Filtered off (Shel/OMIT)	0

Table 31: Fractional Atomic Coordinates (×10^4) and Equivalent Isotropic Displacement Parameters (Å^2×10^3) for **AL22001**. U_{eq} is defined as 1/3 of the trace of the orthogonalised U_{ij}.

Atom	x	y	z	U_{eq}
O1	6931.3(2)	5580.8(6)	5862.6(4)	22.95(12)
O2	7705.6(2)	6779.6(5)	6782.4(3)	17.27(11)
O3	5798.7(3)	8594.9(5)	8153.1(4)	23.11(12)
O4	4696.1(2)	5426.5(5)	6177.3(3)	17.40(11)
O4'	6295.8(2)	8619.3(5)	5857.1(3)	19.81(11)
N1	6788.9(3)	7435.9(6)	7934.2(4)	14.28(12)
N2	5599.8(3)	6901.6(6)	6209.1(4)	12.87(12)
C1	7205.1(4)	7200.4(9)	8821.9(5)	20.85(16)
C2	7407.7(4)	5699.4(9)	8776.1(6)	24.85(17)
C3	6887.9(4)	5056.5(8)	8025.4(6)	19.53(15)
C4	6699.1(3)	6225.3(7)	7344.6(5)	12.46(13)
C5	7109.2(3)	6176.0(7)	6569.1(5)	13.80(13)
C6	8153.4(4)	6670.8(11)	6108.1(7)	25.50(18)
C7	6113.4(3)	7764.4(7)	7794.3(5)	14.56(14)

Atom	x	y	z	U_{eq}
C8	5929.0(3)	6595.4(7)	7108.2(5)	12.55(13)
C9	4979.6(3)	6326.1(7)	5831.0(5)	12.90(13)
C9'	5786.3(3)	7957.8(7)	5667.9(5)	13.25(13)
C10	4763.9(3)	7059.7(7)	4964.4(5)	13.39(13)
C10'	5242.6(3)	8049.8(7)	4870.2(5)	13.29(13)
C11	4191.5(4)	6888.5(8)	4325.6(5)	18.92(15)
C11'	5163.0(4)	8935.3(8)	4137.3(5)	17.71(15)
C12	4110.4(4)	7769.9(9)	3580.2(5)	22.45(17)
C12'	4584.2(4)	8780.7(9)	3488.9(5)	21.58(16)

Table 32: Anisotropic Displacement Parameters (×10[4]) for **AL22001.** The anisotropic displacement factor exponent takes the form: $-2\pi^2[h^2a^{*2} \times U_{11} + ... + 2hka^* \times b^* \times U_{12}]$

Atom	U_{11}	U_{22}	U_{33}	U_{23}	U_{13}	U_{12}
O1	19.8(3)	30.5(3)	20.0(3)	-7.1(2)	7.6(2)	-12.7(2)
O2	12.8(2)	22.1(3)	17.6(3)	-2.1(2)	4.25(19)	-4.7(2)
O3	23.8(3)	24.3(3)	21.1(3)	10.3(2)	2.9(2)	-7.0(2)
O4	16.5(2)	16.0(3)	20.2(3)	-4.62(19)	4.3(2)	1.4(2)
O4'	16.4(2)	21.4(3)	21.0(3)	-7.6(2)	0.9(2)	2.8(2)
N1	14.6(3)	16.0(3)	12.0(3)	1.7(2)	1.1(2)	-1.1(2)
N2	11.4(3)	14.8(3)	12.4(3)	-1.4(2)	1.7(2)	1.0(2)
C1	18.6(4)	29.1(4)	13.7(4)	2.9(3)	-1.4(3)	-1.3(3)
H1a	33(6)	50(7)	44(7)	-11(5)	-7(5)	-1(5)
H1b	36(6)	76(8)	24(6)	14(5)	12(5)	-2(5)
C2	22.8(4)	29.6(5)	21.0(4)	6.5(3)	-0.5(3)	8.8(3)
H2a	16(5)	57(7)	73(8)	12(5)	12(5)	-1(6)
H2b	73(8)	57(7)	36(6)	-6(6)	24(6)	20(6)
C3	18.6(4)	17.0(4)	23.3(4)	4.2(3)	4.2(3)	6.8(3)
H3a	51(6)	25(6)	41(6)	17(5)	11(5)	3(5)
H3b	24(5)	40(6)	57(7)	-2(5)	8(5)	10(5)
C4	12.3(3)	12.7(3)	12.7(3)	1.5(2)	2.8(2)	0.6(3)
C5	12.9(3)	14.2(3)	14.7(3)	0.3(2)	3.4(3)	-2.6(3)
C6	16.9(4)	34.9(5)	26.9(5)	-4.2(4)	10.1(3)	-6.1(4)
H6a	27(6)	67(8)	71(8)	-19(5)	18(5)	-24(6)
H6b	74(8)	42(7)	108(10)	-9(6)	51(7)	-34(7)
H6c	50(7)	123(11)	35(7)	21(7)	18(6)	24(7)
C7	16.4(3)	15.3(3)	12.0(3)	4.3(3)	1.8(3)	-1.0(3)
C8	11.7(3)	14.0(3)	12.2(3)	1.0(3)	2.7(2)	0.5(3)
H8	32(5)	26(5)	30(5)	-4(4)	11(4)	8(4)
C9	12.1(3)	13.2(3)	13.9(3)	-1.1(2)	3.6(2)	-0.8(3)
C9'	12.1(3)	14.2(3)	13.7(3)	-2.2(2)	2.6(2)	0.6(3)
C10	12.1(3)	15.2(3)	12.7(3)	-0.4(2)	1.3(2)	-1.2(3)
C10'	13.1(3)	15.2(3)	11.8(3)	0.1(2)	2.5(2)	1.1(3)
C11	14.8(3)	22.9(4)	18.0(4)	-0.4(3)	-1.3(3)	-2.5(3)
H11	27(5)	39(6)	44(6)	-17(5)	-2(5)	-1(5)
C11'	18.9(4)	19.4(4)	15.6(4)	2.7(3)	5.0(3)	4.2(3)
H11'	39(6)	31(6)	46(6)	-7(5)	5(5)	19(5)
C12	18.9(4)	30.8(4)	16.1(4)	4.7(3)	-2.6(3)	-0.2(3)
H12	36(6)	63(7)	22(6)	1(5)	-9(5)	1(5)
C12'	22.0(4)	27.8(4)	14.8(4)	8.0(3)	2.5(3)	4.9(3)
H12'	43(6)	49(7)	27(6)	7(5)	-10(5)	16(5)

Table 33: Bond Lengths in Å for **AL22001**.

Atom	Atom	Length/Å	Atom	Atom	Length/Å
O1	C5	1.2065(8)	O2	C5	1.3299(8)

Atom	Atom	Length/Å
O2	C6	1.4426(9)
O3	C7	1.2053(8)
O4	C9	1.2093(8)
O4'	C9'	1.2085(8)
N1	C1	1.4658(9)
N1	C4	1.4758(8)
N1	C7	1.3761(8)
N2	C8	1.4269(9)
N2	C9	1.4037(8)
N2	C9'	1.4000(8)
C1	C2	1.5414(12)
C2	C3	1.5414(12)
C3	C4	1.5421(10)
C4	C5	1.5094(9)
C4	C8	1.5723(9)
C7	C8	1.5459(10)
C9	C10	1.4807(10)
C9'	C10'	1.4844(9)
C10	C10'	1.3911(9)
C10	C11	1.3828(10)
C10'	C11'	1.3844(10)
C11	C12	1.3961(11)
C11'	C12'	1.3982(11)
C12	C12'	1.3978(12)

Table 34: Bond Angles in ° for **AL22001**.

Atom	Atom	Atom	Angle/°
C6	O2	C5	115.56(6)
C4	N1	C1	113.86(6)
C7	N1	C1	125.90(6)
C7	N1	C4	94.14(5)
C9	N2	C8	122.94(6)
C9'	N2	C8	124.52(6)
C9'	N2	C9	111.68(5)
C2	C1	N1	103.37(6)
C3	C2	C1	105.92(6)
C4	C3	C2	103.65(6)
C3	C4	N1	102.92(5)
C5	C4	N1	116.38(5)
C5	C4	C3	111.42(6)
C8	C4	N1	88.38(5)
C8	C4	C3	117.12(6)
C8	C4	C5	117.73(6)
O2	C5	O1	124.21(6)
C4	C5	O1	123.51(6)
C4	C5	O2	112.12(6)
N1	C7	O3	131.39(7)
C8	C7	O3	135.16(6)
C8	C7	N1	93.17(5)
C4	C8	N2	123.89(6)
C7	C8	N2	119.11(6)
C7	C8	C4	84.13(5)
N2	C9	O4	124.88(6)
C10	C9	O4	129.25(6)
C10	C9	N2	105.85(5)
N2	C9'	O4'	123.96(6)
C10'	C9'	O4'	130.11(6)
C10'	C9'	N2	105.93(5)
C10'	C10	C9	108.37(6)
C11	C10	C9	129.59(6)
C11	C10	C10'	122.03(7)
C10	C10'	C9'	108.13(6)
C11'	C10'	C9'	130.33(6)
C11'	C10'	C10	121.52(6)
C12	C11	C10	116.80(7)
C12'	C11'	C10'	117.11(7)
C12'	C12	C11	121.45(7)
C12	C12'	C11'	121.08(7)

Table 35: Torsion Angles in ° for **AL22001**.

Atom	Atom	Atom	Atom	Angle/°
O1	C5	O2	C6	-0.85(10)
O1	C5	C4	N1	-151.54(7)
O1	C5	C4	C3	90.89(8)
O1	C5	C4	C8	-48.53(8)
O2	C5	C4	N1	32.93(6)
O2	C5	C4	C3	-84.64(6)
O2	C5	C4	C8	135.94(6)
O3	C7	N1	C1	47.52(10)
O3	C7	N1	C4	171.10(9)
O3	C7	C8	N2	63.31(9)
O3	C7	C8	C4	-170.94(10)
O4	C9	N2	C8	-7.86(8)
O4	C9	N2	C9'	-177.63(7)
O4	C9	C10	C10'	177.48(8)
O4	C9	C10	C11	-1.22(9)
O4'	C9'	N2	C8	9.51(9)

Atom	Atom	Atom	Atom	Angle/°
O4'	C9'	N2	C9	179.09(7)
O4'	C9'	C10'	C10	179.87(8)
O4'	C9'	C10'	C11'	-1.65(10)
N1	C1	C2	C3	-20.44(7)
N1	C4	C3	C2	-29.71(6)
N1	C4	C8	N2	118.38(4)
N1	C4	C8	C7	-2.95(5)
N1	C7	C8	N2	-122.58(5)
N1	C7	C8	C4	3.17(5)
N2	C8	C4	C3	-137.92(7)
N2	C8	C4	C5	-0.79(8)
N2	C9	C10	C10'	-1.59(6)
N2	C9	C10	C11	179.71(5)
N2	C9'	C10'	C10	-0.21(6)
N2	C9'	C10'	C11'	178.27(5)
C1	C2	C3	C4	31.54(7)
C2	C3	C4	C5	95.73(6)
C2	C3	C4	C8	-124.58(6)
C3	C4	C8	C7	100.75(7)
C9	C10	C10'	C9'	1.11(6)
C9	C10	C10'	C11'	-177.53(5)
C9	C10	C11	C12	177.87(8)
C9'	C10'	C10	C11	179.92(5)
C9'	C10'	C11'	C12'	-179.09(8)
C10	C10'	C11'	C12'	-0.79(8)
C10	C11	C12	C12'	-0.36(8)
C10'	C11'	C12'	C12	-0.23(8)
C11	C12	C12'	C11'	0.82(9)

Table 36: Hydrogen Fractional Atomic Coordinates (×10[4]) and Equivalent Isotropic Displacement Parameters (Å^2×10^3) for **AL22001**. U_{eq} is defined as 1/3 of the trace of the orthogonalised U_{ij}.

Atom	x	y	z	U_{eq}
H1a	7636(4)	7876(10)	8920(6)	44(3)
H1b	6903(4)	7409(10)	9360(6)	45(3)
H2a	7918(4)	5627(10)	8580(7)	48(3)
H2b	7392(5)	5183(10)	9414(6)	54(3)
H3a	7079(4)	4176(9)	7715(6)	38(2)
H3b	6439(4)	4748(9)	8298(6)	40(2)
H6a	8612(4)	7142(10)	6384(7)	54(3)
H6b	8244(5)	5649(11)	5970(8)	71(4)
H6c	7943(5)	7149(13)	5493(7)	68(4)
H8	5621(4)	5838(8)	7395(6)	29(2)
H11	3837(4)	6110(9)	4401(6)	38(2)
H11'	5521(4)	9717(9)	4075(6)	39(2)
H12	3669(4)	7666(10)	3067(6)	42(3)
H12'	4507(4)	9470(9)	2915(6)	41(3)

Citations

CrysAlisPro (Rigaku, V1.171.41.123a, 2022)

CrysAlisPro (ROD), Rigaku Oxford Diffraction, Poland (?).

L.J. Bourhis and O.V. Dolomanov and R.J. Gildea and J.A.K. Howard and H. Puschmann, The Anatomy of a Comprehensive Constrained, Restrained, Refinement Program for the Modern Computing Environment - Olex2 Disected, *Acta Cryst. A*, (2015), **A71**, 59-71.

O.V. Dolomanov and L.J. Bourhis and R.J. Gildea and J.A.K. Howard and H. Puschmann, Olex2: A complete structure solution, refinement and analysis program, *J. Appl. Cryst.*, (2009), **42**, 339-341.

Sheldrick, G.M., ShelXT-Integrated space-group and crystal-structure determination, *Acta Cryst.*, (2015), **A71**, 3-8.

```
#==============================================================================
# PLATON/CHECK-(301021) versus check.def version 210713, Entry: AL22001
# Data: AL22001.cif - Type: CIF                  Bond Precision   C-C = 0.0011 A
# Refl: AL22001.fcf - Type: LIST4                                 Temp = 120 K
# Audit:OLEX2 1.5-BETA (COMPILED 2022.04.07 SVN.RCA3783A0 FOR OLEXSYS, GUI SVN.R
# Refin:OLEX2.REFINE 1.5-BETA (BOURHIS ET AL., 2015)
# X-ray MoKa              R(int) = 0.049,  wR2/R(int) =  1.1,  Nref/Npar =  15.2
# Cell  20.0240(4)   9.8782(3)  14.8097(3)           90    98.623(2)          90
# Wavelength 0.71073  Volume Reported     2896.26(12) Calculated     2896.26(12)
# SpaceGroup from Symmetry C 2/c        Hall: -C 2yc                  monoclinic
#                Reported C 1 2/c 1          -C 2yc                  monoclinic
# MoietyFormula C16 H14 N2 O5
#      Reported C16 H14 N2 O5
#    SumFormula C16 H14 N2 O5
#      Reported C16 H14 N2 O5
# Mr        =    314.29[Calc],    314.30[Rep]       Volume/NonHatoms = 16 Ang**3
# Dx,gcm-3  =     1.442[Calc],     1.442[Rep]
# Z         =         8[Calc],         8[Rep]
# Mu (mm-1) =     0.109[Calc],     0.109[Rep]  Xtal Size =  0.110x0.140x0.190 mm
# F000      =    1312.0[Calc],    1313.0[Rep]  or F000' =   1312.73[Calc]
# Reported   T Limits: Tmin=0.984              Tmax=1.000  AbsCorr = MULTI-SCAN
# Calculated T Limits: Tmin=0.982 Tmin'=0.980  Tmax=0.988
# Measured   HKL: Reported  45005, Embedded      0, <Mult>  0.0
# Reported   Hmax= 30, Kmax= 14, Lmax= 22, Nref=   5076        , Th(max)= 32.840
# Obs in FCF Hmax= 30, Kmax= 14, Lmax= 22, Nref=   5076[  5076], Th(max)= 32.842
# Calculated Hmax= 30, Kmax= 15, Lmax= 22, Nref=   5389        , Ratio  =  0.942
# Reported   Rho(min) = -0.28, Rho(max) =  0.28 e/Ang**3 (From CIF)
# Calculated Rho(min) = -0.26, Rho(max) =  0.51 e/Ang**3 (From CIF+FCF data)
# w=1/[<sup>2</sup>(Fo<sup>2</sup>)+(0.0132P)<sup>2</sup>+0.3044P],
P=(Fo<sup>2</sup>+2Fc<sup>2</sup>)/3
# R= 0.0579(  4229), wR2= 0.1098(  5076), S = 2.319      (From CIF+FCF data)
# R= 0.0360(  4229), wR2= 0.0522(  5076), S = 1.102      (From FCF data only)
# R= 0.0360(  4229), wR2= 0.0521(  5076), S = 1.102, Npar=  334
#==============================================================================
# For Documentation:http://www.platonsoft.nl/CIF-VALIDATION.pdf
#==============================================================================
*
#==============================================================================
#>>> The Following Improvement and Query ALERTS were generated - (Acta-Mode) <<<
#==============================================================================
Format: alert-number_ALERT_alert-type_alert-level text

351_ALERT_3_C Long   C-H (X0.96,N1.08A)  C2       - H2A      .       1.11 Ang.
906_ALERT_3_C Large K Value in the Analysis of Variance ......      7.786 Check
910_ALERT_3_C Missing # of FCF Reflection(s) Below Theta(Min).          6 Note
#==============================================================================
068_ALERT_1_G Reported F000 Differs from Calcd (or Missing)...     Please Check
793_ALERT_4_G Model has Chirality at C4          (Centro SPGR)          S Verify
793_ALERT_4_G Model has Chirality at C8          (Centro SPGR)          S Verify
802_ALERT_4_G CIF Input Record(s) with more than 80 Characters          1 Info
912_ALERT_4_G Missing # of FCF Reflections Above STh/L=  0.600        306 Note
978_ALERT_2_G Number C-C Bonds with Positive Residual Density.          9 Info
979_ALERT_1_G NoSpherA2 Scattering Factors Used .............     Please Note
#==============================================================================

ALERT_Level and ALERT_Type Summary
==================================
3 ALERT_Level_C = Check. Ensure it is Not caused by an Omission or Oversight
7 ALERT_Level_G = General Info/Check that it is not Something Unexpected

2 ALERT_Type_1 CIF Construction/Syntax Error, Inconsistent or Missing Data.
1 ALERT_Type_2 Indicator that the Structure Model may be Wrong or Deficient.
3 ALERT_Type_3 Indicator that the Structure Quality may be Low.
4 ALERT_Type_4 Improvement, Methodology, Query or Suggestion.
#==============================================================================

0 Missing  Experimental  Info Issue(s) (Out of  64 Tests) -  100 % Satisfied
0 Experimental  Data  Related Issue(s) (Out of  35 Tests) -  100 % Satisfied
3 Structural  Model   Related Issue(s) (Out of 136 Tests) -   98 % Satisfied
```

7 Unresolved or to be Checked Issue(s) (Out of 271 Tests) -   97 % Satisfied

*

4.7. X-ray Crystal Structure Data of β-Lactam S-3: CCDC number 2426515

Submitted by: **Adam Lockyer**

Solved by: **Gary S Nichol**

Sample ID: **AL-016-Fr1-Fr3**

Compound AL-016-Fr1-Fr3 was provided as crystals suitable for single crystal X-ray diffraction, yielding structure AL22002.

Crystal Data and Experimental

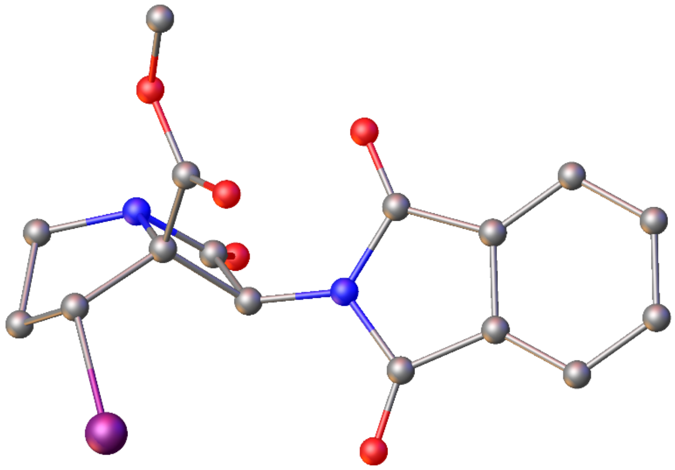

**Compound**	**AL22002**
Formula	$C_{16}H_{13}IN_2O_5$
$D_{calc.}$/ g cm^{-3}	1.827
μ/mm^{-1}	2.030
Formula Weight	440.18
Colour	colourless
Shape	plate-shaped
Size/mm^3	0.44×0.09×0.03
T/K	120.00(10)
Crystal System	monoclinic
Space Group	$P2_1/c$
a/Å	16.1416(5)
b/Å	8.06030(10)
c/Å	13.3185(4)
$\alpha/^\circ$	90
$\beta/^\circ$	112.548(3)
$\gamma/^\circ$	90
V/Å^3	1600.36(8)
Z	4
Z'	1
Wavelength/Å	0.71073
Radiation type	Mo K_α
$\Theta_{min}/^\circ$	3.312
$\Theta_{max}/^\circ$	29.129
Measured Refl's.	43718
Indep't Refl's	4301
Refl's I≥2 σ(I)	3790
R_{int}	0.0541
Parameters	269
Restraints	0
Largest Peak	1.366
Deepest Hole	-0.686
GooF	1.106
wR_2 (all data)	0.0924
wR_2	0.0888
R_1 (all data)	0.0446
R_1	0.0369

Experimental. Single colourless plate-shaped crystals of **AL22002** recrystallised from diethyl ether by slow evaporation. A suitable crystal with dimensions 0.44 × 0.09 × 0.03 mm^3 was selected and mounted on a MITIGEN holder in Paratone oil on a Rigaku Oxford Diffraction SuperNova diffractometer. The crystal was kept at a steady T = 120.00(10) K during data collection. The structure was solved with the **ShelXT** 2018/2 (Sheldrick, 2018) solution program using dual methods and by using **Olex2** 1.5-beta (Dolomanov et al., 2009) as the graphical interface. The model was refined with **ShelXL** 2018/3 (Sheldrick, 2015) using full matrix least squares minimisation on $\boldsymbol{F^2}$.

Crystal Data. $C_{16}H_{13}IN_2O_5$, M_r = 440.18, monoclinic, $P2_1/c$ (No. 14), a = 16.1416(5) Å, b = 8.06030(10) Å, c = 13.3185(4) Å, β = 112.548(3)°, α = γ = 90°, V = 1600.36(8) Å^3, T = 120.00(10) K, Z = 4, Z' = 1, μ(Mo K_α) = 2.030, 43718 reflections measured, 4301 unique (R_{int} = 0.0541) which were used in all calculations. The final wR_2 was 0.0924 (all data) and R_1 was 0.0369 (I≥2 σ(I)).

Structure Quality Indicators

**Reflections:**	d min (Mo) 2Θ=58.3° 0.73	I/σ(I) 35.1	Rint 5.41%	Full 50.5° 99.8
**Refinement:**	Shift 0.001	Max Peak 1.4	Min Peak -0.7	GooF 1.106

A colourless plate-shaped-shaped crystal with dimensions 0.44 × 0.09 × 0.03 mm^3 was mounted on a MITIGEN holder in Paratone oil. Data were collected using a Rigaku Oxford Diffraction SuperNova diffractometer equipped with an Oxford Cryosystems Cryostream 700+ low-temperature device operating at T = 120.00(10) K.

Data were measured using ω scans with Mo K_α radiation. The diffraction pattern was indexed and the total number of runs and images was based on the strategy calculation from the program CrysAlisPro 1.171.41.123a (Rigaku OD, 2022). The maximum resolution that was achieved was Θ = 29.129° (0.73 Å).

The unit cell was refined using CrysAlisPro 1.171.41.123a (Rigaku OD, 2022) on 14991 reflections, 34% of the observed reflections.

Data reduction, scaling and absorption corrections were performed using CrysAlisPro 1.171.41.123a (Rigaku OD, 2022). The final completeness is 99.80 % out to 29.129° in Θ. A multi-scan absorption correction was performed using CrysAlisPro 1.171.41.123a (Rigaku Oxford Diffraction, 2022) Spherical absorption correction using equivalent radius and absorption coefficient. Empirical absorption correction using spherical harmonics, implemented in SCALE3 ABSPACK scaling algorithm.. The absorption coefficient μ of this material is 2.030 mm^{-1} at this wavelength (λ = 0.71073Å) and the minimum and maximum transmissions are 0.819 and 0.822.

The structure was solved and the space group $P2_1/c$ (# 14) determined by the ShelXT 2018/2 (Sheldrick, 2018) structure solution program using using dual methods and refined by full matrix least squares minimisation on $\boldsymbol{F}^2$ using version 2018/3 of **ShelXL** 2018/3 (Sheldrick, 2015). All non-hydrogen atoms were refined anisotropically. Hydrogen atom positions were calculated geometrically and refined using the riding model.

_refine_special_details: H atoms were identified in a difference map and freely refined.

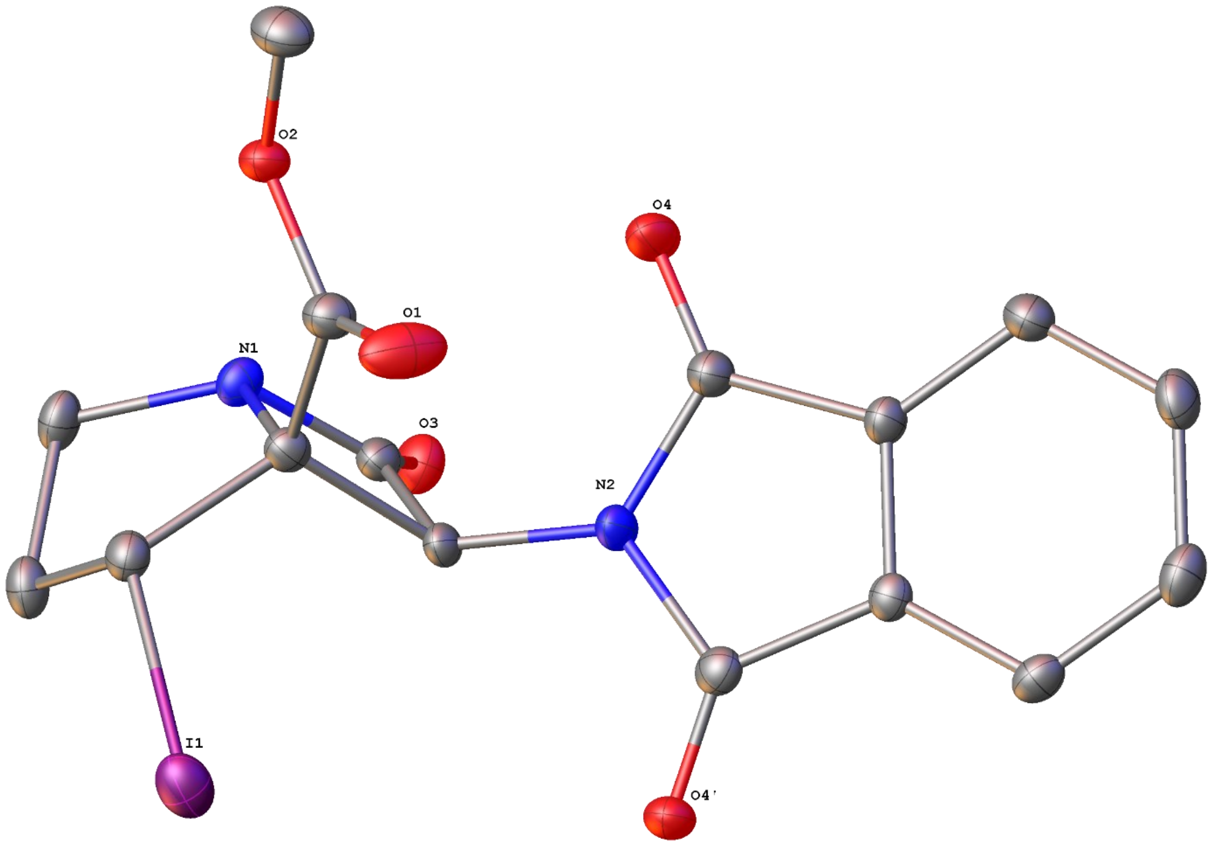

Figure 7: The molecular structure of AL22002. Displacement ellipsoids are at the 50% probability level and H atoms are not shown.

Data Plots: Diffraction Data

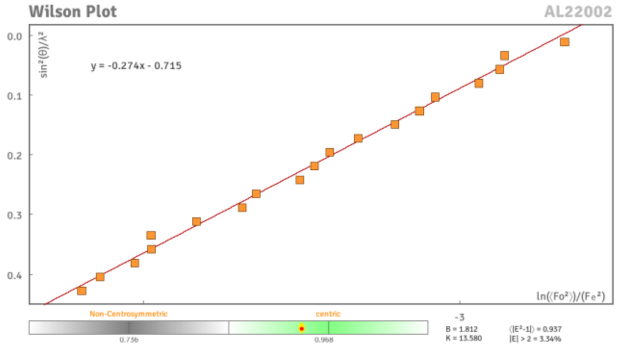

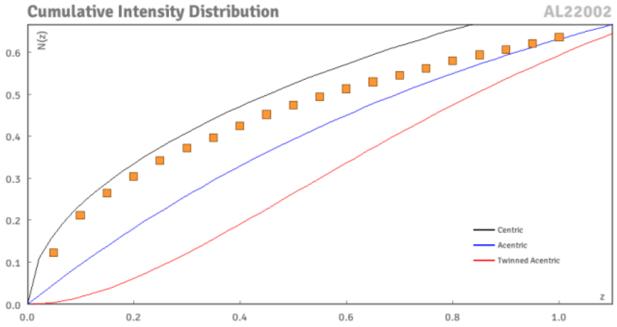

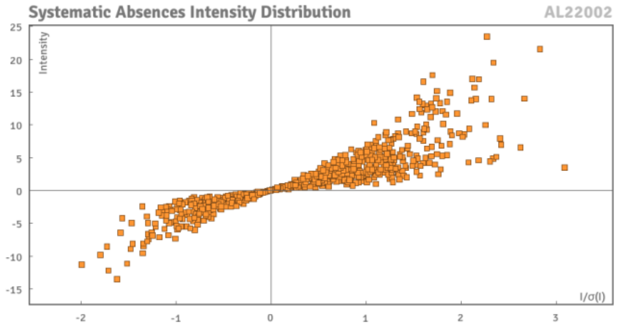

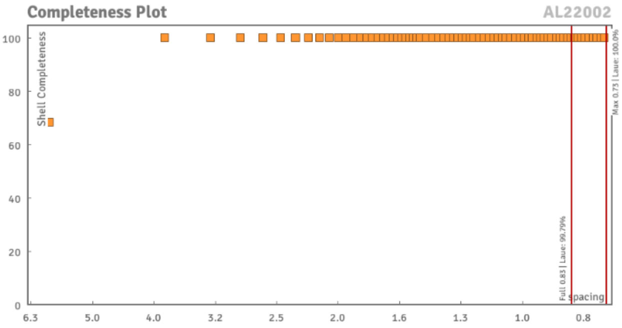

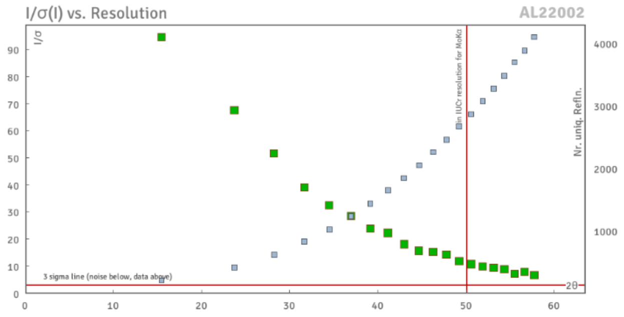

Data Plots: Refinement and Data

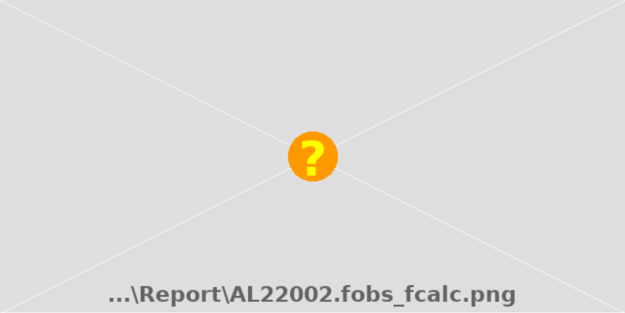

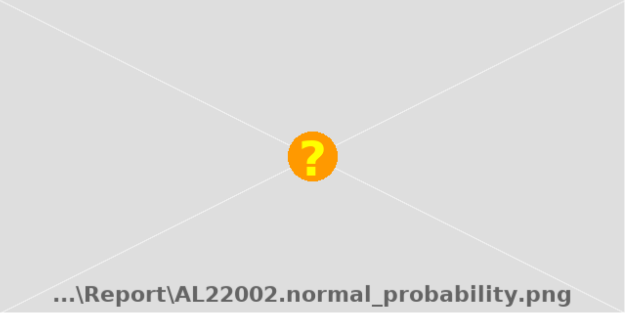

Reflection Statistics

Total reflections (after filtering)	44873	Unique reflections	4301
Completeness	1.0	Mean I/σ	24.76
hkl_{max} collected	(22, 11, 18)	hkl_{min} collected	(-22, -11, -18)
hkl_{max} used	(20, 11, 18)	hkl_{min} used	(-22, 0, 0)
Lim d_{max} collected	100.0	Lim d_{min} collected	0.36
d_{max} used	6.15	d_{min} used	0.73
Friedel pairs	8457	Friedel pairs merged	1
Inconsistent equivalents	0	R_{int}	0.0541
R_{sigma}	0.0285	Intensity transformed	0
Omitted reflections	0	Omitted by user (OMIT hkl)	0
Multiplicity	(3252, 6167, 4307, 1799, 750, 402, 181, 105, 55, 25, 12, 2)	Maximum multiplicity	34
Removed systematic absences	1155	Filtered off (Shel/OMIT)	0

Table 37: Fractional Atomic Coordinates (×10^4) and Equivalent Isotropic Displacement Parameters (Å^2×10^3) for **AL22002**. U_{eq} is defined as 1/3 of the trace of the orthogonalised U_{ij}.

Atom	x	y	z	U_{eq}
I1	2892.1(2)	10384.6(3)	2534.7(2)	24.26(8)
O1	2602.2(18)	5726(3)	2023(2)	33.4(6)
O2	1121.4(15)	5236(3)	1368.6(18)	17.7(4)
O3	882.8(15)	7213(3)	4577.0(18)	20.7(5)
O4	1996.6(15)	3797(3)	3944(2)	24.8(5)
O4'	3963.5(15)	8052(3)	5408.2(19)	20.2(5)
N1	812.9(17)	7360(3)	2772(2)	15.1(5)
N2	2838.0(17)	6182(3)	4475(2)	16.9(5)
C1	219(2)	8734(4)	2200(3)	18.9(6)
C2	897(2)	10085(4)	2215(3)	19.4(6)
C3	1641(2)	9097(4)	2050(3)	16.5(6)
C4	1682(2)	7428(3)	2642(2)	14.5(5)
C5	1868(2)	6018(4)	2003(3)	18.0(6)
C6	1228(3)	4004(4)	651(3)	22.2(6)
C7	1207(2)	7249(3)	3900(2)	16.1(6)
C8	2164(2)	7365(3)	3908(2)	14.7(5)

Atom	x	y	z	U_{eq}
C9	2703(2)	4463(4)	4459(3)	17.2(6)
C9'	3706(2)	6646(4)	5206(2)	15.6(6)
C10	3575.9(19)	3761(4)	5198(2)	14.9(5)
C10'	4170(2)	5056(4)	5645(2)	13.4(5)
C11	3824(2)	2122(4)	5466(3)	19.2(6)
C11'	5033(2)	4776(4)	6380(3)	17.3(6)
C12	4698(2)	1840(4)	6200(3)	21.1(6)
C12'	5283(2)	3132(4)	6654(3)	20.9(6)

Table 38: Anisotropic Displacement Parameters (×10[4]) for **AL22002.** The anisotropic displacement factor exponent takes the form: $-2\pi^2[h^2a^{*2} \times U_{11} + \ldots + 2hka^* \times b^* \times U_{12}]$

Atom	U_{11}	U_{22}	U_{33}	U_{23}	U_{13}	U_{12}
I1	20.82(13)	21.56(12)	32.20(14)	5.19(8)	12.16(9)	1.72(8)
O1	18.9(13)	31.7(13)	49.7(17)	-20.1(12)	13.3(12)	-1.3(10)
O2	15.8(11)	13.3(9)	20.6(11)	-1.7(8)	3.2(8)	-1.9(8)
O3	20.0(12)	23.9(11)	16.6(11)	1.6(9)	5.3(9)	1.7(9)
O4	15.4(11)	16.8(10)	33.3(13)	0.5(9)	-0.6(9)	-1.9(9)
O4'	18.3(11)	13.5(10)	23.3(11)	-0.7(8)	2.0(9)	-3.0(8)
N1	10.7(12)	17.5(11)	15.9(12)	-0.7(9)	4.0(9)	1.2(9)
N2	11.6(12)	12.8(11)	21.3(13)	1.4(10)	0.7(10)	1.5(9)
C1	12.8(14)	23.3(15)	17.7(15)	4.0(12)	2.6(12)	4.3(12)
C2	17.7(16)	19.3(14)	19.8(16)	5.4(12)	5.8(12)	6.0(12)
C3	16.5(15)	16.1(13)	14.8(14)	0.6(11)	3.8(11)	1.8(11)
C4	11.5(13)	13.4(12)	17.6(14)	0.0(10)	4.7(11)	-0.3(10)
C5	16.8(15)	15.1(13)	20.5(15)	-2.2(11)	5.6(12)	-0.8(11)
C6	26.9(18)	17.4(14)	22.9(16)	-4.5(13)	10.2(14)	-2.0(13)
C7	14.9(14)	11.7(12)	18.4(14)	-0.4(10)	2.6(11)	0.5(10)
C8	12.2(14)	10.6(12)	17.5(14)	1.3(10)	1.6(11)	2.1(10)
C9	15.1(14)	14.2(13)	19.0(14)	-0.2(11)	3.0(11)	0.2(11)
C9'	15.5(14)	16.8(13)	13.6(13)	0.5(10)	4.7(11)	-0.4(11)
C10	11.6(14)	14.7(13)	16.3(13)	1.4(10)	3.1(11)	1.6(10)
C10'	12.7(13)	15.2(12)	12.6(13)	0.6(10)	5.0(10)	1.1(10)
C11	20.1(16)	14.2(13)	20.4(15)	-0.8(11)	4.6(12)	-0.6(11)
C11'	12.9(14)	20.3(14)	16.9(14)	-1.4(11)	3.7(11)	-2.2(11)
C12	21.2(16)	17.3(14)	22.1(16)	3.4(12)	5.4(13)	7.3(12)
C12'	15.3(16)	26.1(16)	17.9(15)	2.5(12)	2.7(12)	5.0(12)

Table 39: Bond Lengths in Å for **AL22002.**

Atom	Atom	Length/Å
I1	C3	2.139(3)
O1	C5	1.198(4)
O2	C5	1.336(4)
O2	C6	1.433(4)
O3	C7	1.204(4)
O4	C9	1.207(4)
O4'	C9'	1.202(4)
N1	C1	1.471(4)
N1	C4	1.480(4)
N1	C7	1.392(4)
N2	C8	1.426(4)
N2	C9	1.402(4)
N2	C9'	1.416(4)
C1	C2	1.538(5)

Atom	Atom	Length/Å
C2	C3	1.525(4)
C3	C4	1.547(4)
C4	C5	1.517(4)
C4	C8	1.563(4)
C7	C8	1.544(4)
C9	C10	1.487(4)
C9'	C10'	1.486(4)
C10	C10'	1.388(4)
C10	C11	1.387(4)
C10'	C11'	1.380(4)
C11	C12	1.393(5)
C11'	C12'	1.392(4)
C12	C12'	1.381(5)

Table 40: Bond Angles in ° for **AL22002**.

Atom	Atom	Atom	Angle/°
C5	O2	C6	116.1(3)
C1	N1	C4	111.8(2)
C7	N1	C1	123.0(2)
C7	N1	C4	93.8(2)
C9	N2	C8	124.8(2)
C9	N2	C9'	112.2(2)
C9'	N2	C8	122.7(2)
N1	C1	C2	101.9(2)
C3	C2	C1	102.9(3)
C2	C3	I1	114.1(2)
C2	C3	C4	105.3(2)
C4	C3	I1	114.6(2)
N1	C4	C3	103.5(2)
N1	C4	C5	116.0(2)
N1	C4	C8	88.6(2)
C3	C4	C8	119.0(2)
C5	C4	C3	110.2(2)
C5	C4	C8	117.2(2)
O1	C5	O2	124.5(3)
O1	C5	C4	122.8(3)
O2	C5	C4	112.6(3)
O3	C7	N1	131.3(3)

Atom	Atom	Atom	Angle/°
O3	C7	C8	135.9(3)
N1	C7	C8	92.6(2)
N2	C8	C4	123.8(3)
N2	C8	C7	121.4(2)
C7	C8	C4	85.0(2)
O4	C9	N2	123.8(3)
O4	C9	C10	131.0(3)
N2	C9	C10	105.3(2)
O4'	C9'	N2	124.7(3)
O4'	C9'	C10'	130.3(3)
N2	C9'	C10'	105.0(2)
C10'	C10	C9	108.8(2)
C11	C10	C9	129.7(3)
C11	C10	C10'	121.5(3)
C10	C10'	C9'	108.6(3)
C11'	C10'	C9'	129.8(3)
C11'	C10'	C10	121.6(3)
C10	C11	C12	116.8(3)
C10'	C11'	C12'	117.0(3)
C12'	C12	C11	121.5(3)
C12	C12'	C11'	121.5(3)

Table 41: Torsion Angles in ° for **AL22002**.

Atom	Atom	Atom	Atom	Angle/°
I1	C3	C4	N1	143.88(19)
I1	C3	C4	C5	-91.5(3)
I1	C3	C4	C8	47.9(3)
O3	C7	C8	N2	56.9(5)
O3	C7	C8	C4	-176.2(4)
O4	C9	C10	C10'	-176.6(4)
O4	C9	C10	C11	2.5(6)
O4'	C9'	C10'	C10	177.1(3)
O4'	C9'	C10'	C11'	-2.1(6)
N1	C1	C2	C3	37.4(3)
N1	C4	C5	O1	-164.2(3)
N1	C4	C5	O2	20.3(4)
N1	C4	C8	N2	126.4(3)
N1	C4	C8	C7	1.58(19)
N1	C7	C8	N2	-128.6(3)
N1	C7	C8	C4	-1.7(2)
N2	C9	C10	C10'	2.3(3)
N2	C9	C10	C11	-178.7(3)
N2	C9'	C10'	C10	-1.6(3)
N2	C9'	C10'	C11'	179.2(3)
C1	N1	C4	C3	6.7(3)
C1	N1	C4	C5	-114.1(3)
C1	N1	C4	C8	126.2(2)
C1	N1	C7	O3	57.6(5)
C1	N1	C7	C8	-117.4(3)
C1	C2	C3	I1	-160.9(2)
C1	C2	C3	C4	-34.4(3)
C2	C3	C4	N1	17.7(3)
C2	C3	C4	C5	142.4(3)
C2	C3	C4	C8	-78.2(3)
C3	C4	C5	O1	78.7(4)
C3	C4	C5	O2	-96.8(3)

Atom	Atom	Atom	Atom	Angle/°
C3	C4	C8	N2	-129.0(3)
C3	C4	C8	C7	106.2(3)
C4	N1	C1	C2	-27.9(3)
C4	N1	C7	O3	176.7(3)
C4	N1	C7	C8	1.8(2)
C5	C4	C8	N2	7.7(4)
C5	C4	C8	C7	-117.1(3)
C6	O2	C5	O1	-1.9(5)
C6	O2	C5	C4	173.5(3)
C7	N1	C1	C2	82.3(3)
C7	N1	C4	C3	-121.2(2)
C7	N1	C4	C5	118.0(3)
C7	N1	C4	C8	-1.7(2)
C8	N2	C9	O4	1.2(5)
C8	N2	C9	C10	-177.7(3)
C8	N2	C9'	O4'	-1.2(5)
C8	N2	C9'	C10'	177.6(3)
C8	C4	C5	O1	-61.6(4)
C8	C4	C5	O2	123.0(3)
C9	N2	C8	C4	-62.9(4)
C9	N2	C8	C7	43.6(4)
C9	N2	C9'	O4'	-175.6(3)
C9	N2	C9'	C10'	3.2(3)
C9	C10	C10'	C9'	-0.4(3)
C9	C10	C10'	C11'	178.8(3)
C9	C10	C11	C12	-179.3(3)
C9'	N2	C8	C4	123.4(3)
C9'	N2	C8	C7	-130.1(3)
C9'	N2	C9	O4	175.5(3)
C9'	N2	C9	C10	-3.4(3)
C9'	C10'	C11'	C12'	179.2(3)
C10	C10'	C11'	C12'	0.1(5)
C10	C11	C12	C12'	1.2(5)
C10'	C10	C11	C12	-0.4(5)
C10'	C11'	C12'	C12	0.7(5)
C11	C10	C10'	C9'	-179.5(3)
C11	C10	C10'	C11'	-0.3(5)
C11	C12	C12'	C11'	-1.4(5)

Table 42: Hydrogen Fractional Atomic Coordinates (×10^4) and Equivalent Isotropic Displacement Parameters (Å^2×10^3) for **AL22002.** U_{eq} is defined as 1/3 of the trace of the orthogonalised U_{ij}.

Atom	x	y	z	U_{eq}
H11A	3440(30)	1230(50)	5170(30)	23(10)
H11'	5380(30)	5600(50)	6680(30)	21(10)
H3	1500(20)	8910(50)	1360(30)	14(9)
H2A	1120(20)	10520(40)	2820(30)	9(8)
H2B	690(30)	10800(50)	1730(30)	19(9)
H6A	1610(30)	3120(50)	1030(30)	23(10)
H1A	-130(30)	8340(50)	1500(30)	19(9)
H6B	1460(30)	4500(60)	240(40)	36(13)
H1B	-160(30)	9100(50)	2580(30)	27(10)
H12'	5760(30)	2940(50)	7060(30)	17(9)
H6C	690(30)	3530(50)	320(30)	23(10)
H11B	4840(30)	780(50)	6350(30)	24(10)
H8	2400(30)	8370(50)	4130(30)	23(10)

Citations

CrysAlisPro (Rigaku, V1.171.41.123a, 2022)

CrysAlisPro (ROD), Rigaku Oxford Diffraction, Poland (?).

O.V. Dolomanov and L.J. Bourhis and R.J. Gildea and J.A.K. Howard and H. Puschmann, Olex2: A complete structure solution, refinement and analysis program, *J. Appl. Cryst.*, (2009), **42**, 339-341.

Sheldrick, G.M., Crystal structure refinement with ShelXL, *Acta Cryst.*, (2015), **C71**, 3-8.

Sheldrick, G.M., ShelXT-Integrated space-group and crystal-structure determination, *Acta Cryst.*, (2015), **A71**, 3-8.

```
#==============================================================================
# PLATON/CHECK-(181221) versus check.def version 211218, Entry: al22002
# Data: AL22002.cif - Type: CIF                  Bond Precision   C-C = 0.0046 A
# Refl: AL22002.fcf - Type: LIST4                                  Temp = 120 K
# Audit:OLEX2 1.5-BETA (COMPILED 2022.04.07 SVN.RCA3783A0 FOR OLEXSYS, GUI SVN.R
# Refin:SHELXL 2018/3 (SHELDRICK, 2015)
# X-ray MoKa              R(int) = 0.054,  wR2/R(int) =  1.7,  Nref/Npar =  16.0
# Cell  16.1416(5)   8.0603(1)  13.3185(4)          90   112.548(3)         90
# Wavelength 0.71073  Volume Reported      1600.36(8) Calculated      1600.36(8)
# SpaceGroup from Symmetry P 21/c       Hall: -P 2ybc                 monoclinic
#                 Reported P 1 21/c 1         -P 2ybc                 monoclinic
# MoietyFormula C16 H13 I N2 O5
#      Reported C16 H13 I N2 O5
#    SumFormula C16 H13 I N2 O5
#      Reported C16 H13 I N2 O5
# Mr         =    440.18[Calc],    440.18[Rep]       Volume/NonHatoms = 17 Ang**3
# Dx,gcm-3  =     1.827[Calc],     1.827[Rep]
# Z          =         4[Calc],         4[Rep]
# Mu (mm-1) =     2.030[Calc],     2.030[Rep]  Xtal Size =  0.030x0.090x0.440 mm
# F000      =     864.0[Calc],     864.0[Rep]  or F000' =    862.50[Calc]
# Reported   T Limits: Tmin=0.819              Tmax=0.822  AbsCorr = MULTI-SCAN
# Calculated T Limits: Tmin=0.803 Tmin'=0.409  Tmax=0.941
# Measured   HKL: Reported  43718, Embedded  44873, <Mult> 10.2
# Reported   Hmax= 22, Kmax= 11, Lmax= 18, Nref=   4301        , Th(max)= 29.129
# Obs in FCF Hmax= 22, Kmax= 11, Lmax= 18, Nref=   4301[  4301], Th(max)= 29.129
# Calculated Hmax= 22, Kmax= 11, Lmax= 18, Nref=   4307        , Ratio  =  0.999
# Reported   Rho(min) = -0.69, Rho(max) =  1.37 e/Ang**3 (From CIF)
# Calculated Rho(min) = -0.73, Rho(max) =  1.59 e/Ang**3 (From CIF+FCF data)
# w=1/[<sup>2</sup>(Fo<sup>2</sup>)+(0.0407P)<sup>2</sup>+3.2543P],
P=(Fo<sup>2</sup>+2Fc<sup>2</sup>)/3
# R= 0.0369(  3790), wR2= 0.0925(  4301), S = 1.106      (From CIF+FCF data)
# R= 0.0369(  3790), wR2= 0.0924(  4301), S = 1.106      (From FCF data only)
# R= 0.0369(  3790), wR2= 0.0924(  4301), S = 1.106, Npar=  269
#==============================================================================
# For Documentation:http://www.platonsoft.nl/CIF-VALIDATION.pdf
#==============================================================================
*
#==============================================================================
#>>> The Following Improvement and Query ALERTS were generated - (Acta-Mode) <<<
#==============================================================================
Format: alert-number_ALERT_alert-type_alert-level text

350_ALERT_3_C Short  C-H (X0.96,N1.08A)  C2       - H2A      .       0.82 Ang.
350_ALERT_3_C Short  C-H (X0.96,N1.08A)  C2       - H2B      .       0.83 Ang.
350_ALERT_3_C Short  C-H (X0.96,N1.08A)  C12'     - H12'     .       0.77 Ang.
906_ALERT_3_C Large K Value in the Analysis of Variance ......      3.850 Check
910_ALERT_3_C Missing # of FCF Reflection(s) Below Theta(Min).          6 Note
971_ALERT_2_C Check Calcd Resid. Dens.  0.93Ang From I1             1.59 eA-3
#==============================================================================
164_ALERT_4_G Nr. of Refined C-H H-Atoms in Heavy-Atom Struct.         13 Note
793_ALERT_4_G Model has Chirality at C3          (Centro SPGR)          R Verify
793_ALERT_4_G Model has Chirality at C4          (Centro SPGR)          S Verify
793_ALERT_4_G Model has Chirality at C8          (Centro SPGR)          S Verify
978_ALERT_2_G Number C-C Bonds with Positive Residual Density.          8 Info
#==============================================================================

ALERT_Level and ALERT_Type Summary
==================================
6 ALERT_Level_C = Check. Ensure it is Not caused by an Omission or Oversight
5 ALERT_Level_G = General Info/Check that it is not Something Unexpected

2 ALERT_Type_2 Indicator that the Structure Model may be Wrong or Deficient.
5 ALERT_Type_3 Indicator that the Structure Quality may be Low.
4 ALERT_Type_4 Improvement, Methodology, Query or Suggestion.
#==============================================================================

0 Missing  Experimental  Info Issue(s) (Out of  64 Tests) -  100 % Satisfied
0 Experimental  Data  Related Issue(s) (Out of  35 Tests) -  100 % Satisfied
7 Structural  Model   Related Issue(s) (Out of 136 Tests) -   95 % Satisfied
```

4 Unresolved or to be Checked Issue(s) (Out of 272 Tests) -   99 % Satisfied

*

4.8. X-ray Crystal Structure Data of Hemiaminal S-4: CCDC number 2426516

Submitted by: **Adam Lockyer**

Solved by: **Gary S Nichol**

Sample ID: **AL-032-Fr3**

Compound AL-032-Fr3 was provided as crystals suitable for single crystal X-ray diffraction, yielding structure AL22003.

Crystal Data and Experimental

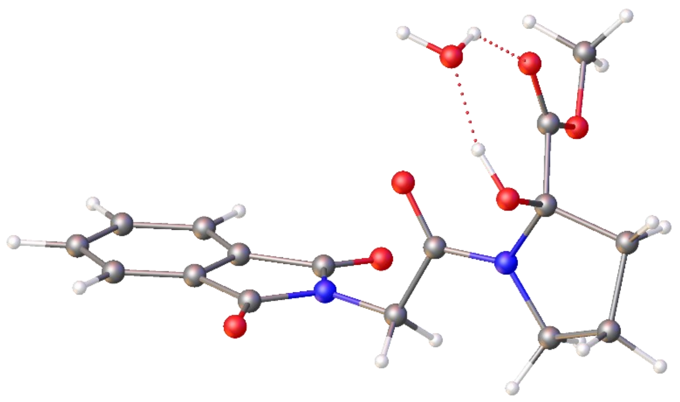

Experimental. Single colourless block-shaped crystals of **AL22003** recrystallised from diethyl ether by slow evaporation. A suitable crystal with dimensions 0.39 × 0.19 × 0.12 mm³ was selected and mounted on a MITIGEN holder in Paratone oil on a Rigaku Oxford Diffraction XCalibur diffractometer. The crystal was kept at a steady T = 120.00(10) K during data collection. The structure was solved with the **ShelXS** (Sheldrick, 2008) solution program using direct methods and by using **Olex2** 1.5-beta (Dolomanov et al., 2009) as the graphical interface. The model was refined with **olex2.refine** 1.5-beta (Bourhis et al., 2015) using full matrix least squares minimisation on $\boldsymbol{F}^2$.

Crystal Data. $C_{16}H_{18}N_2O_7$, M_r = 350.331, triclinic, P-1 (No. 2), a = 8.1206(3) Å, b = 8.8968(5) Å, c = 12.1035(6) Å, α = 78.275(5)°, β = 87.172(4)°, γ = 67.777(5)°, V = 792.23(8) Å³, T = 120.00(10) K, Z = 2, Z' = 1, μ(Mo K$_\alpha$) = 0.117, 30748 reflections measured, 5485 unique (R_{int} = 0.0407) which were used in all calculations. The final wR_2 was 0.0555 (all data) and R_1 was 0.0335 (I≥2 σ(I)).

**Compound**	**AL22003**
Formula	$C_{16}H_{18}N_2O_7$
$D_{calc.}$/ g cm^{-3}	1.469
μ/mm^{-1}	0.117
Formula Weight	350.331
Colour	colourless
Shape	block-shaped
Size/mm³	0.39×0.19×0.12
T/K	120.00(10)
Crystal System	triclinic
Space Group	P-1
a/Å	8.1206(3)
b/Å	8.8968(5)
c/Å	12.1035(6)
α/°	78.275(5)
β/°	87.172(4)
γ/°	67.777(5)
V/Å³	792.23(8)
Z	2
Z'	1
Wavelength/Å	0.71073
Radiation type	Mo K$_\alpha$
Θ_{min}/°	3.44
Θ_{max}/°	32.79
Measured Refl's.	30748
Indep't Refl's	5485
Refl's I≥2 σ(I)	4803
R_{int}	0.0407
Parameters	388
Restraints	0
Largest Peak	0.2637
Deepest Hole	-0.2922
GooF	1.1318
wR_2 (all data)	0.0555
wR_2	0.0529
R_1 (all data)	0.0431
R_1	0.0335

Structure Quality Indicators

**Reflections:**	d min (Mo) 2Θ=65.6° 0.66	I/σ(I) 32.2	Rint 4.07%	Full 50.5° 93% to 65.6° 99.7
**Refinement:**	Shift 0.000	Max Peak 0.3	Min Peak -0.3	GooF 1.132

A colourless block-shaped-shaped crystal with dimensions 0.39 × 0.19 × 0.12 mm^3 was mounted on a MITIGEN holder in Paratone oil. Data were collected using a Rigaku Oxford Diffraction XCalibur diffractometer equipped with an Oxford Cryosystems Cryostream 700+ low-temperature device operating at T = 120.00(10) K.

Data were measured using ω scans with Mo K_α radiation. The diffraction pattern was indexed and the total number of runs and images was based on the strategy calculation from the program CrysAlisPro 1.171.41.123a (Rigaku OD, 2022). The maximum resolution that was achieved was Θ = 32.79° (0.66 Å).

The unit cell was refined using CrysAlisPro 1.171.41.123a (Rigaku OD, 2022) on 11079 reflections, 36% of the observed reflections.

Data reduction, scaling and absorption corrections were performed using CrysAlisPro 1.171.41.123a (Rigaku OD, 2022). The final completeness is 99.65 % out to 32.79° in Θ. A multi-scan absorption correction was performed using CrysAlisPro 1.171.41.123a (Rigaku Oxford Diffraction, 2022) Empirical absorption correction using spherical harmonics, implemented in SCALE3 ABSPACK scaling algorithm.. The absorption coefficient μ of this material is 0.117 mm^{-1} at this wavelength (λ = 0.71073Å) and the minimum and maximum transmissions are 0.995 and 1.000.

The structure was solved and the space group *P*-1 (# 2) determined by the ShelXS (Sheldrick, 2008) structure solution program using using direct methods and refined by full matrix least squares minimisation on $\boldsymbol{F}^2$ using version of **olex2.refine** 1.5-beta (Bourhis et al., 2015). All non-hydrogen atoms were refined anisotropically. Hydrogen atom positions were calculated geometrically and refined using the riding model.

_refine_special_details: Refinement using NoSpherA2, an implementation ofNOn-SPHERical Atom-form-factors in Olex2.Please cite:F. Kleemiss et al. Chem. Sci. DOI 10.1039/D0SC05526C - 2021NoSpherA2 implementation of HAR makes use oftailor-made aspherical atomic form factors calculatedon-the-fly from a Hirshfeld-partitioned electron density (ED) - not fromspherical-atom form factors.The ED is calculated from a gaussian basis set single determinant SCFwavefunction - either Hartree-Fock or DFT using selected funtionals - for a fragment of the crystal.This fragment can be embedded in an electrostatic crystal field by employing cluster chargesor modelled using implicit solvation models, depending on the software used.The following options were used: SOFTWARE: ORCA PARTITIONING: NoSpherA2 INT ACCURACY: Normal METHOD: PBE BASIS SET: cc-pVTZ CHARGE: 0 MULTIPLICITY: 1 DATE: 2022-05-19_12-04-46

There is a single molecule in the asymmetric unit, which is represented by the reported sum formula. In other words: Z is 2 and Z' is 1.

Data Plots: Diffraction Data

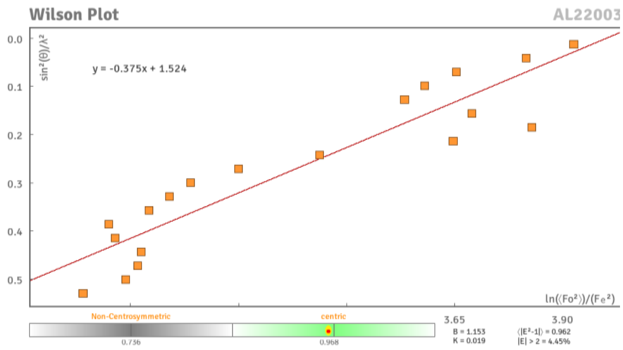

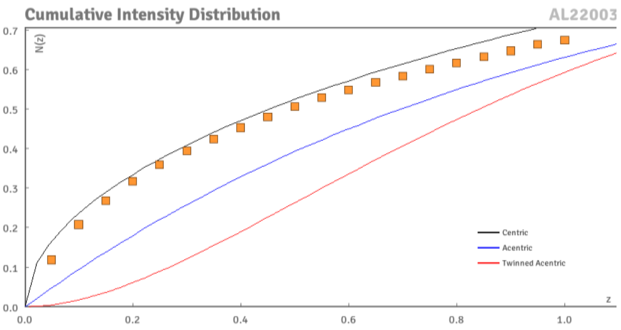

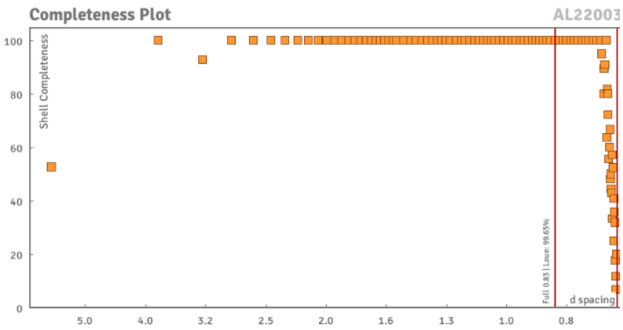

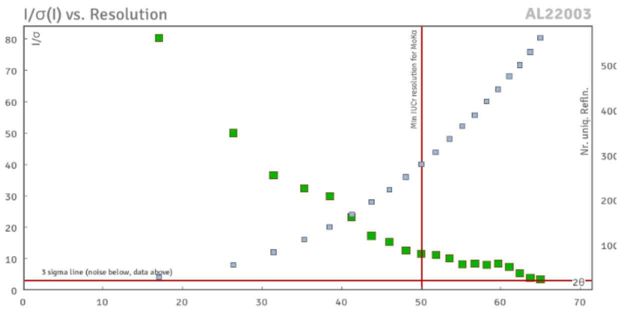

Data Plots: Refinement and Data

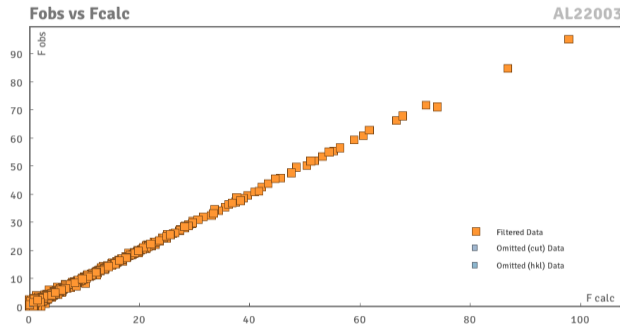

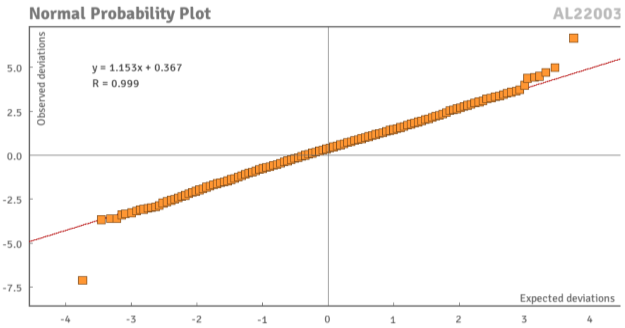

Reflection Statistics

Total reflections (after filtering)	30748	Unique reflections	5485
Completeness	0.932	Mean I/σ	20.18
hkl_{max} collected	(12, 13, 18)	hkl_{min} collected	(-11, -13, -18)
hkl_{max} used	(12, 13, 18)	hkl_{min} used	(-12, -12, 0)
Lim d_{max} collected	100.0	Lim d_{min} collected	0.36
d_{max} used	5.92	d_{min} used	0.66
Friedel pairs	5156	Friedel pairs merged	1
Inconsistent equivalents	0	R_{int}	0.0405
R_{sigma}	0.031	Intensity transformed	0
Omitted reflections	0	Omitted by user (OMIT hkl)	5
Multiplicity	(1261, 3428, 3234, 1534, 699, 263, 127, 55, 25, 11, 4, 1)	Maximum multiplicity	22
Removed systematic absences	0	Filtered off (Shel/OMIT)	0

Table 43: Fractional Atomic Coordinates (×10⁴) and Equivalent Isotropic Displacement Parameters (Å²×10³) for **AL22003**. U_{eq} is defined as 1/3 of the trace of the orthogonalised U_{ij}.

Atom	x	y	z	U_{eq}
O1	2931.0(7)	11572.9(6)	10351.9(4)	16.0(1)
O2	843.6(7)	14775.4(6)	7987.5(4)	16.99(11)
O3	-308.3(7)	13695.3(6)	9518.2(4)	16.96(11)
O4	1856.8(7)	10936.3(6)	8143.4(4)	16.70(11)
O5	4735.5(8)	6700.2(7)	7660.9(4)	22.72(12)
O6	2789.3(7)	11465.0(7)	4990.2(4)	22.28(12)
N1	3916.0(7)	11923.7(7)	8475.0(5)	11.68(11)
N2	3807.6(8)	9305.9(7)	6523.2(5)	14.78(12)
C1	5644.6(9)	12108.8(9)	8322.0(6)	13.33(13)
C2	5840.2(10)	12728.5(9)	9371.7(6)	15.39(13)
C3	3919.4(9)	13781.7(9)	9590.7(6)	13.59(13)
C4	2889.5(9)	12733.3(8)	9370.4(5)	11.55(12)
C5	951.8(9)	13764.4(8)	8973.6(5)	12.35(12)
C6	-936.2(11)	15844.8(10)	7572.1(7)	20.39(15)
C7	3332.0(9)	11011.0(8)	7965.0(5)	11.73(12)
C8	4613.0(10)	10060.6(10)	7160.1(6)	15.90(14)
C9	3975.7(9)	7652.2(9)	6817.4(6)	15.08(13)
C9'	2980.5(9)	10072.0(9)	5464.2(6)	14.82(13)
C10	3087.7(9)	7362.0(9)	5882.5(5)	14.53(13)
C10'	2482.6(9)	8816.7(9)	5069.2(5)	14.23(13)
C11	2846.1(10)	5951.3(10)	5750.0(7)	19.99(15)
C11'	1597.6(10)	8925.3(11)	4090.6(6)	18.57(15)
C12	1959.3(11)	6043.7(11)	4763.9(7)	22.48(16)
C12'	1343.7(10)	7506.8(11)	3949.6(6)	21.47(16)
O7	-99.8(9)	11234.8(10)	11546.1(6)	32.85(15)

Table 44: Anisotropic Displacement Parameters (×10[4]) for **AL22003**. The anisotropic displacement factor exponent takes the form: $-2\pi^2[h^2a^{*2} \times U_{11} + ... + 2hka^* \times b^* \times U_{12}]$

Atom	U_{11}	U_{22}	U_{33}	U_{23}	U_{13}	U_{12}
O1	16.9(3)	15.6(3)	14.2(2)	-5.8(2)	-0.31(19)	-0.43(18)
H1	21(6)	42(7)	47(7)	-29(6)	0(5)	-2(5)
O2	14.4(2)	16.5(2)	15.6(2)	-2.8(2)	0.14(18)	0.73(19)
O3	12.0(2)	19.3(3)	19.0(2)	-5.9(2)	2.85(19)	-3.1(2)
O4	13.7(2)	20.1(3)	21.1(2)	-9.3(2)	2.85(19)	-9.4(2)
O5	29.2(3)	22.6(3)	16.7(2)	-11.9(2)	-9.4(2)	1.9(2)
O6	26.8(3)	16.1(3)	21.3(3)	-6.5(2)	-2.4(2)	-0.3(2)
N1	10.8(3)	12.3(3)	13.0(3)	-4.5(2)	1.0(2)	-4.6(2)
N2	17.3(3)	15.7(3)	11.8(3)	-5.4(2)	-1.0(2)	-5.1(2)
C1	10.6(3)	13.7(3)	15.9(3)	-4.7(3)	1.0(2)	-3.2(3)
H1a	25(5)	20(5)	36(5)	1(4)	5(4)	-11(4)
H1b	38(6)	36(6)	29(5)	-21(5)	-4(5)	5(5)
C2	12.2(3)	15.0(3)	20.5(4)	-5.8(3)	-2.1(3)	-5.1(3)
H2a	30(6)	38(6)	48(6)	-23(5)	7(5)	-16(5)
H2b	29(6)	19(5)	29(5)	4(4)	-15(4)	0(4)
C3	14.2(3)	12.7(3)	15.7(3)	-6.0(3)	0.2(3)	-5.1(3)
H3a	32(6)	23(6)	35(6)	-11(5)	0(4)	2(5)
H3b	31(6)	34(6)	28(5)	-16(5)	6(4)	-17(5)
C4	11.1(3)	11.3(3)	12.3(3)	-4.0(2)	0.4(2)	-2.8(2)
C5	10.7(3)	12.1(3)	13.8(3)	-3.5(2)	0.2(2)	-3.2(2)
C6	17.7(4)	17.9(4)	20.4(4)	-1.0(3)	-5.7(3)	-2.3(3)
H6a	32(6)	38(7)	56(7)	9(5)	-8(5)	-30(6)
H6b	57(7)	55(7)	17(5)	-2(6)	-12(5)	23(5)
H6c	41(7)	40(7)	66(8)	-20(6)	-6(6)	-18(6)
C7	11.6(3)	11.9(3)	12.3(3)	-4.5(2)	0.1(2)	-3.4(2)
C8	15.3(3)	18.7(4)	16.1(3)	-6.8(3)	1.7(3)	-8.0(3)
H8a	45(7)	42(7)	34(6)	-32(6)	12(5)	-15(5)
H8b	33(6)	31(6)	44(6)	1(5)	-16(5)	-19(5)
C9	16.7(3)	17.3(3)	12.0(3)	-6.9(3)	-2.2(2)	-2.8(3)

Atom	U_{11}	U_{22}	U_{33}	U_{23}	U_{13}	U_{12}
C9'	14.8(3)	15.4(3)	13.0(3)	-3.6(3)	0.4(2)	-4.2(2)
C10	15.4(3)	17.4(3)	11.9(3)	-6.7(3)	-0.7(2)	-4.0(2)
C10'	13.1(3)	18.0(3)	11.0(3)	-4.3(3)	-0.1(2)	-4.4(2)
C11	23.5(4)	21.2(4)	18.7(4)	-11.5(3)	-2.2(3)	-4.6(3)
H11	62(8)	39(7)	26(6)	-22(6)	-14(5)	5(5)
C11'	16.0(3)	25.9(4)	12.4(3)	-5.6(3)	-2.3(3)	-4.6(3)
H11'	42(7)	31(6)	30(6)	-8(5)	-10(5)	1(5)
C12	24.2(4)	29.0(4)	21.1(4)	-14.8(3)	-0.5(3)	-10.4(3)
H12	58(8)	47(7)	50(7)	-23(6)	-19(5)	-23(5)
C12'	18.6(4)	33.2(5)	16.2(3)	-11.0(3)	-2.1(3)	-9.7(3)
H12'	50(7)	69(8)	36(6)	-27(6)	-21(5)	-14(5)
O7	29.1(3)	33.4(4)	38.4(4)	-21.5(3)	-8.5(3)	9.3(3)
H7a	74(10)	55(9)	65(9)	-35(8)	-45(7)	57(7)
H7b	71(9)	40(8)	88(10)	-39(8)	-40(8)	4(7)

Table 45: Bond Lengths in Å for **AL22003**.

Atom	Atom	Length/Å
O1	C4	1.3990(8)
O2	C5	1.3249(8)
O2	C6	1.4437(9)
O3	C5	1.2042(8)
O4	C7	1.2308(8)
O5	C9	1.2066(8)
O6	C9'	1.2097(9)
N1	C1	1.4726(8)
N1	C4	1.4703(8)
N1	C7	1.3376(8)
N2	C8	1.4372(9)
N2	C9	1.3964(9)
N2	C9'	1.3936(9)

Atom	Atom	Length/Å
C1	C2	1.5212(10)
C2	C3	1.5283(10)
C3	C4	1.5343(9)
C4	C5	1.5335(9)
C7	C8	1.5217(10)
C9	C10	1.4856(9)
C9'	C10'	1.4889(10)
C10	C10'	1.3908(10)
C10	C11	1.3822(10)
C10'	C11'	1.3834(9)
C11	C12	1.3965(10)
C11'	C12'	1.3972(11)
C12	C12'	1.3967(12)

Table 46: Bond Angles in ° for **AL22003.**

Atom	Atom	Atom	Angle/°
C6	O2	C5	115.53(6)
C4	N1	C1	112.54(5)
C7	N1	C1	126.59(6)
C7	N1	C4	120.58(5)
C9	N2	C8	123.88(6)
C9'	N2	C8	123.54(6)
C9'	N2	C9	112.03(6)
C2	C1	N1	102.62(5)
C3	C2	C1	102.74(6)
C4	C3	C2	102.87(6)
N1	C4	O1	111.38(5)
C3	C4	O1	109.42(5)
C3	C4	N1	102.17(5)
C5	C4	O1	109.48(5)
C5	C4	N1	110.82(5)
C5	C4	C3	113.42(6)
O3	C5	O2	124.57(6)
C4	C5	O2	111.82(5)
C4	C5	O3	123.56(6)
N1	C7	O4	122.01(6)

Atom	Atom	Atom	Angle/°
C8	C7	O4	122.22(6)
C8	C7	N1	115.76(6)
C7	C8	N2	112.61(6)
N2	C9	O5	124.65(6)
C10	C9	O5	129.50(7)
C10	C9	N2	105.83(6)
N2	C9'	O6	124.55(6)
C10'	C9'	O6	129.52(6)
C10'	C9'	N2	105.90(6)
C10'	C10	C9	108.20(6)
C11	C10	C9	130.08(7)
C11	C10	C10'	121.71(6)
C10	C10'	C9'	107.93(6)
C11'	C10'	C9'	130.50(7)
C11'	C10'	C10	121.56(7)
C12	C11	C10	117.27(7)
C12'	C11'	C10'	117.17(7)
C12'	C12	C11	121.03(7)
C12	C12'	C11'	121.26(7)

Table 47: Torsion Angles in ° for **AL22003**.

Atom	Atom	Atom	Atom	Angle/°
O1	C4	N1	C1	-104.38(6)
O1	C4	N1	C7	69.90(6)
O1	C4	C3	C2	85.25(6)
O1	C4	C5	O2	-174.00(5)
O1	C4	C5	O3	8.43(6)
O2	C5	C4	N1	-50.75(6)
O2	C5	C4	C3	63.48(6)
O3	C5	C4	N1	131.67(7)
O3	C5	C4	C3	-114.09(7)
O4	C7	N1	C1	-179.71(6)
O4	C7	N1	C4	6.87(8)
O4	C7	C8	N2	9.00(7)
O5	C9	N2	C8	3.79(9)
O5	C9	N2	C9'	175.52(7)
O5	C9	C10	C10'	-176.92(8)
O5	C9	C10	C11	2.43(10)
O6	C9'	N2	C8	-3.06(9)
O6	C9'	N2	C9	-174.83(7)
O6	C9'	C10'	C10	175.87(8)
O6	C9'	C10'	C11'	-3.64(10)
N1	C1	C2	C3	-33.52(6)
N1	C4	C3	C2	-32.88(5)
N1	C7	C8	N2	-171.54(6)
N2	C9	C10	C10'	1.64(6)
N2	C9	C10	C11	-179.00(6)
N2	C9'	C10'	C10	-2.15(6)
N2	C9'	C10'	C11'	178.34(5)
C1	C2	C3	C4	41.73(6)
C2	C3	C4	C5	-152.20(5)
C9	C10	C10'	C9'	0.30(6)
C9	C10	C10'	C11'	179.87(6)
C9	C10	C11	C12	-179.51(8)
C9'	C10'	C10	C11	-179.11(6)
C9'	C10'	C11'	C12'	179.14(8)
C10	C10'	C11'	C12'	-0.32(8)
C10	C11	C12	C12'	-0.08(9)
C10'	C11'	C12'	C12	-0.01(8)
C11	C12	C12'	C11'	0.21(9)

Table 48: Hydrogen Fractional Atomic Coordinates (×10[4]) and Equivalent Isotropic Displacement Parameters (Å[2]×10[3]) for **AL22003.** U_{eq} is defined as 1/3 of the trace of the orthogonalised U_{ij}.

Atom	x	y	z	U_{eq}
H1	1781(13)	11452(13)	10410(8)	33(3)
H1a	6711(11)	10969(11)	8258(7)	29(2)
H1b	5599(12)	13023(11)	7565(7)	34(2)
H2a	6693(12)	13412(11)	9243(7)	35(2)
H2b	6391(12)	11696(11)	10077(7)	30(2)
H3a	3477(12)	14942(11)	8983(7)	31(2)
H3b	3700(11)	14002(11)	10430(7)	28(2)
H6a	-1570(12)	16675(12)	8131(8)	46(3)
H6b	-818(13)	16480(13)	6748(7)	53(3)
H6c	-1738(13)	15135(13)	7512(9)	46(3)
H8a	5095(13)	10884(12)	6572(7)	35(2)
H8b	5748(13)	9114(12)	7636(8)	38(3)
H11	3306(14)	4834(13)	6389(7)	42(3)
H11'	1117(13)	10062(12)	3471(7)	36(2)

Atom	x	y	z	U_{eq}
H12	1747(14)	4962(13)	4634(8)	48(3)
H12'	658(13)	7546(13)	3212(8)	49(3)
H7a	-725(18)	12175(16)	10975(11)	70(5)
H7b	-716(17)	10553(14)	11564(10)	62(4)

Table 49: Hydrogen Bond information for **AL22003**.

D	H	A	d(D-H)/Å	d(H-A)/Å	d(D-A)/Å	D-H-A/deg
O1	H1	O7	0.977(9)	2.042(10)	2.8758(9)	142.0(8)
O7	H7b	O4[1]	0.918(10)	1.856(11)	2.7649(8)	170.4(11)

––––
[1]-x,2-y,2-z

Citations

CrysAlisPro (Rigaku, V1.171.41.123a, 2022)

CrysAlisPro (ROD), Rigaku Oxford Diffraction, Poland (?).

L.J. Bourhis and O.V. Dolomanov and R.J. Gildea and J.A.K. Howard and H. Puschmann, The Anatomy of a Comprehensive Constrained, Restrained, Refinement Program for the Modern Computing Environment - Olex2 Disected, *Acta Cryst. A*, (2015), **A71**, 59-71.

O.V. Dolomanov and L.J. Bourhis and R.J. Gildea and J.A.K. Howard and H. Puschmann, Olex2: A complete structure solution, refinement and analysis program, *J. Appl. Cryst.*, (2009), **42**, 339-341.

Sheldrick, G.M., A short history of ShelX, *Acta Cryst.*, (2008), **A64**, 339-341.

```
#==============================================================================
# PLATON/CHECK-(181221) versus check.def version 211218, Entry: AL22003
# Data: AL22003.cif - Type: CIF                  Bond Precision   C-C = 0.0011 A
# Refl: AL22003.fcf - Type: LIST4                                  Temp = 120 K
# Audit:OLEX2 1.5-BETA (COMPILED 2022.04.07 SVN.RCA3783A0 FOR OLEXSYS, GUI SVN.R
# Refin:OLEX2.REFINE 1.5-BETA (BOURHIS ET AL., 2015)
# X-ray MoKa              R(int) = 0.041,  wR2/R(int) =  1.4,  Nref/Npar =  14.1
# Cell   8.1206(3)   8.8968(5)  12.1035(6)    78.275(5)    87.172(4)   67.777(5)
# Wavelength 0.71073  Volume Reported       792.23(8) Calculated       792.24(7)
# SpaceGroup from Symmetry P -1         Hall: -P 1                     triclinic
#                Reported P -1                -P 1                     triclinic
# MoietyFormula C16 H16 N2 O6, H2 O
#      Reported C16 H16 N2 O6, H2 O
#    SumFormula C16 H18 N2 O7
#      Reported C16 H18 N2 O7
# Mr        =    350.32[Calc],    350.33[Rep]       Volume/NonHatoms = 16 Ang**3
# Dx,gcm-3  =     1.469[Calc],     1.469[Rep]
# Z         =         2[Calc],         2[Rep]
# Mu (mm-1) =     0.117[Calc],     0.117[Rep]  Xtal Size =  0.120x0.190x0.390 mm
# F000      =     368.0[Calc],     368.3[Rep]  or F000' =    368.22[Calc]
# Reported   T Limits: Tmin=0.995              Tmax=1.000  AbsCorr = MULTI-SCAN
# Calculated T Limits: Tmin=0.974 Tmin'=0.955  Tmax=0.986
# Measured   HKL: Reported  30748, CIF-loop  30748, <Mult>  5.6
# Reported   Hmax= 12, Kmax= 13, Lmax= 18, Nref=   5485        , Th(max)= 32.790
# Obs in FCF Hmax= 12, Kmax= 13, Lmax= 18, Nref=   5485[  5485], Th(max)= 32.795
# Calculated Hmax= 12, Kmax= 13, Lmax= 18, Nref=   5892        , Ratio  =  0.931
# Reported   Rho(min) = -0.29, Rho(max) =  0.26 e/Ang**3 (From CIF)
# Calculated Rho(min) = -0.26, Rho(max) =  0.52 e/Ang**3 (From CIF+FCF data)
# w=1/[<sup>2</sup>(Fo<sup>2</sup>)+(0.0138P)<sup>2</sup>+0.0555P],
P=(Fo<sup>2</sup>+2Fc<sup>2</sup>)/3
# R= 0.0553(  4802), wR2= 0.1119(  5485), S = 2.282      (From CIF+FCF data)
# R= 0.0335(  4802), wR2= 0.0555(  5485), S = 1.132      (From FCF data only)
# R= 0.0335(  4803), wR2= 0.0555(  5485), S = 1.132, Npar=  388
#==============================================================================
# For Documentation:http://www.platonsoft.nl/CIF-VALIDATION.pdf
#==============================================================================
*
#==============================================================================
#>>> The Following Improvement and Query ALERTS were generated - (Acta-Mode) <<<
#==============================================================================
Format: alert-number_ALERT_alert-type_alert-level text

417_ALERT_2_B Short Inter D-H..H-D       H1       ..H7A      .       2.02 Ang.
x,y,z  =      1_555 Check
#==============================================================================
906_ALERT_3_C Large K Value in the Analysis of Variance ......      6.065 Check
910_ALERT_3_C Missing # of FCF Reflection(s) Below Theta(Min).          9 Note
#==============================================================================
790_ALERT_4_G Centre of Gravity not Within Unit Cell: Resd.  #          2 Note
H2 O
793_ALERT_4_G Model has Chirality at C4          (Centro SPGR)          R Verify
802_ALERT_4_G CIF Input Record(s) with more than 80 Characters          1 Info
912_ALERT_4_G Missing # of FCF Reflections Above STh/L=  0.600        401 Note
913_ALERT_3_G Missing # of Very Strong Reflections in FCF ....          1 Note
933_ALERT_2_G Number of HKL-OMIT Records in Embedded .res File          1 Note
978_ALERT_2_G Number C-C Bonds with Positive Residual Density.         11 Info
979_ALERT_1_G NoSpherA2 Scattering Factors Used .............     Please Note
#==============================================================================

ALERT_Level and ALERT_Type Summary
==================================
1 ALERT_Level_B = A Potentially Serious Problem - Consider Carefully
2 ALERT_Level_C = Check. Ensure it is Not caused by an Omission or Oversight
8 ALERT_Level_G = General Info/Check that it is not Something Unexpected

1 ALERT_Type_1 CIF Construction/Syntax Error, Inconsistent or Missing Data.
3 ALERT_Type_2 Indicator that the Structure Model may be Wrong or Deficient.
3 ALERT_Type_3 Indicator that the Structure Quality may be Low.
4 ALERT_Type_4 Improvement, Methodology, Query or Suggestion.
```

```
#===============================================================================

0 Missing  Experimental  Info Issue(s) (Out of  64 Tests) -  100 % Satisfied
0 Experimental  Data  Related Issue(s) (Out of  35 Tests) -  100 % Satisfied
3 Structural  Model   Related Issue(s) (Out of 136 Tests) -   98 % Satisfied
8 Unresolved or to be Checked Issue(s) (Out of 272 Tests) -   97 % Satisfied

*
```

4.9. X-ray Crystal Structure Data of Tetrafluoroborate S-6: CCDC number 2426511

Submitted by: **Adam Lockyer**

Solved by: **Gary S Nichol**

Sample ID: **AL-4038-CrB2**

Compound AL-4038-CrB2 was provided as crystals suitable for single crystal X-ray diffraction, yielding structure AL23003.

Crystal Data and Experimental

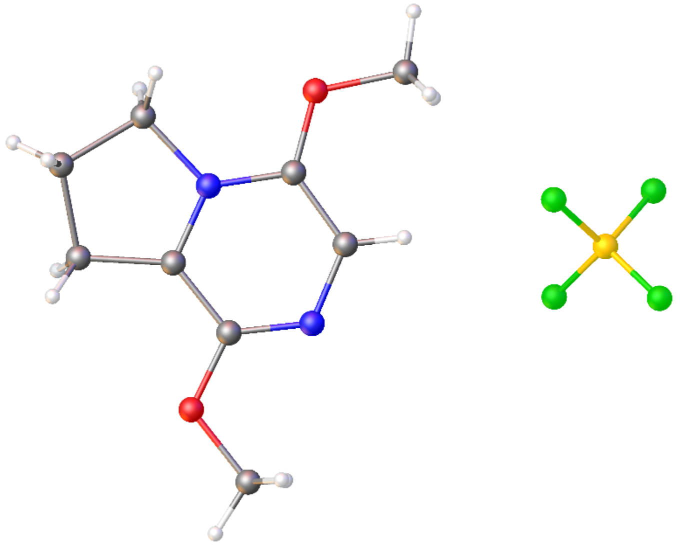

Experimental. Single clear colourless rod-shaped crystals of **AL23003** recrystallised from CDCl3 by slow evaporation. A suitable crystal with dimensions 0.51 × 0.05 × 0.04 mm^3 was selected and mounted on a mitegen tip in Paratone oil on a Rigaku Oxford Diffraction SuperNova diffractometer. The crystal was kept at a steady T = 120.01(10) K during data collection. The structure was solved with the **olex2.solve** 1.5-beta (Bourhis et al., 2015) solution program using iterative methods and by using **Olex2** 1.5-beta (Dolomanov et al., 2009) as the graphical interface. The model was refined with **ShelXL** 2018/3 (Sheldrick, 2015) using full matrix least squares minimisation on $\boldsymbol{F}^2$.

Crystal Data. $C_9H_{13}BF_4N_2O_2$, M_r = 268.02, orthorhombic, $P2_12_12_1$ (No. 19), a = 7.2726(2) Å, b = 9.6396(2) Å, c = 16.8013(4) Å, $\alpha = \beta = \gamma =$ 90°, V = 1177.85(5) Å^3, T = 120.01(10) K, Z = 4, Z' = 1, μ(Cu K$_\alpha$) = 1.292, 19906 reflections measured, 2439 unique (R_{int} = 0.0403) which were used in all calculations. The final wR_2 was 0.0949 (all data) and R_1 was 0.0358 (I≥2 σ(I)).

**Compound**	**AL23003**
Formula	$C_9H_{13}BF_4N_2O_2$
$D_{calc.}$/ g cm^{-3}	1.511
μ/mm^{-1}	1.292
Formula Weight	268.02
Colour	clear colourless
Shape	rod-shaped
Size/mm^3	0.51×0.05×0.04
T/K	120.01(10)
Crystal System	orthorhombic
Flack Parameter	0.42(7)
Hooft Parameter	0.43(7)
Space Group	$P2_12_12_1$
a/Å	7.2726(2)
b/Å	9.6396(2)
c/Å	16.8013(4)
α/°	90
β/°	90
γ/°	90
V/Å^3	1177.85(5)
Z	4
Z'	1
Wavelength/Å	1.54178
Radiation type	Cu K$_\alpha$
Θ_{min}/°	5.265
Θ_{max}/°	75.999
Measured Refl's.	19906
Indep't Refl's	2439
Refl's I≥2 σ(I)	2279
R_{int}	0.0403
Parameters	212
Restraints	83
Largest Peak	0.278
Deepest Hole	-0.199
GooF	1.061
wR_2 (all data)	0.0949
wR_2	0.0929
R_1 (all data)	0.0387
R_1	0.0358

Structure Quality Indicators

**Reflections:**	d min (Cu\a) 2Θ=152.0° 0.79	I/σ(I) 37.5	Rint m=8.19 4.03%	Full 135.4° 100	
**Refinement:**	Shift 0.000	Max Peak 0.3	Min Peak -0.2	GooF 1.061	Hooft .43(7)

A clear colourless rod-shaped-shaped crystal with dimensions 0.51 × 0.05 × 0.04 mm^3 was mounted on a mitegen tip in Paratone oil. Data were collected using a Rigaku Oxford Diffraction SuperNova diffractometer equipped with an Oxford Cryosystems Cryostream 700+ low-temperature device operating at T = 120.01(10) K.

Data were measured using ω scans with Cu K_α radiation. The diffraction pattern was indexed and the total number of runs and images was based on the strategy calculation from the program CrysAlisPro 1.171.42.81a (Rigaku OD, 2023). The maximum resolution that was achieved was Θ = 75.999° (0.79 Å).

The unit cell was refined using CrysAlisPro 1.171.42.81a (Rigaku OD, 2023) on 7950 reflections, 40% of the observed reflections.

Data reduction, scaling and absorption corrections were performed using CrysAlisPro 1.171.42.81a (Rigaku OD, 2023). The final completeness is 100.00 % out to 75.999° in Θ. SADABS-2016/2 (Bruker, 2016/2) was used for absorption correction. wR_2(int) was 0.0894 before and 0.0566 after correction. The Ratio of minimum to maximum transmission is 0.8660. The λ/2 correction factor is Not present. The absorption coefficient μ of this material is 1.292 mm^{-1} at this wavelength (λ = 1.54178Å) and the minimum and maximum transmissions are 0.653 and 0.754.

The structure was solved and the space group $P2_12_12_1$ (# 19) determined by the olex2.solve 1.5-beta (Bourhis et al., 2015) structure solution program using using iterative methods and refined by full matrix least squares minimisation on F^2 using version 2018/3 of **ShelXL** 2018/3 (Sheldrick, 2015). All non-hydrogen atoms were refined anisotropically. Hydrogen atom positions were calculated geometrically and refined using the riding model. Hydrogen atom positions were calculated geometrically and refined using the riding model.

_*refine_special_details*: C2 and the BF4- anion were both modelled as disordered over two positions, consistent with peaks in a difference map.

The Flack parameter was refined to 0.42(7). Determination of absolute structure using Bayesian statistics on Bijvoet differences using the Olex2 results in 0.43(7). Note: The Flack parameter is used to determine chirality of the crystal studied, the value should be near 0, a value of 1 means that the stereochemistry is wrong and the model should be inverted. A value of 0.5 means that the crystal consists of a racemic mixture of the two enantiomers.

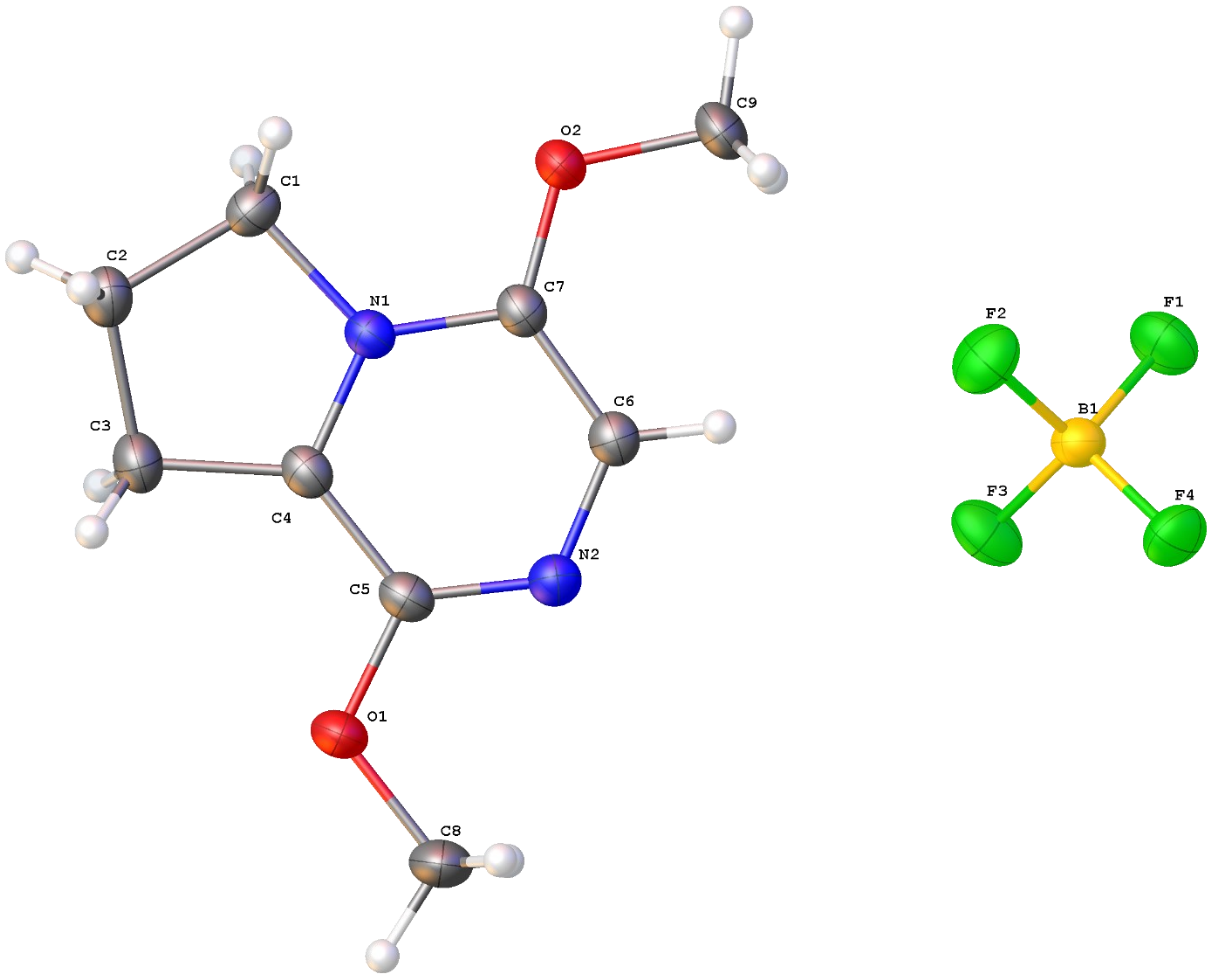

Figure 8: The asymmetric unit of AL23003. Displacement ellipsoids are at the 50% probability level. H atoms are not shown.

Data Plots: Diffraction Data

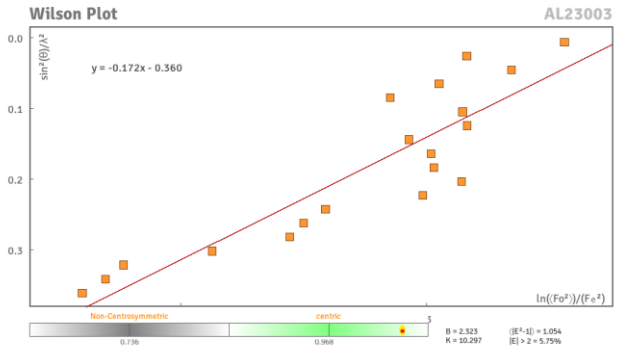

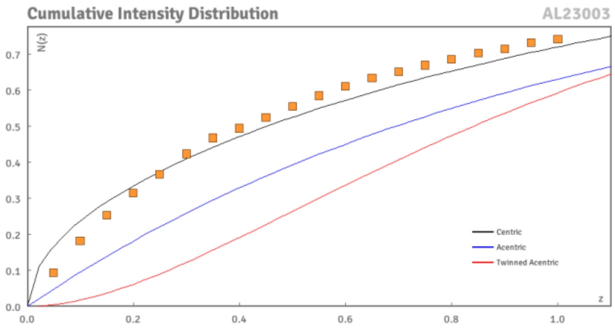

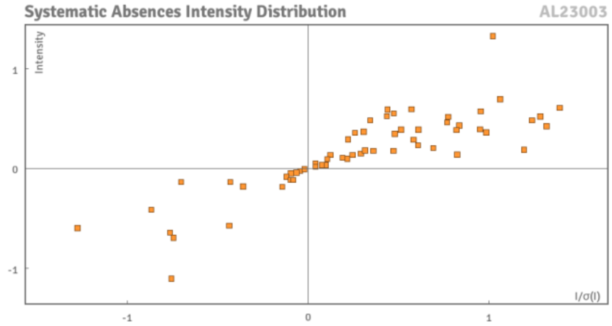

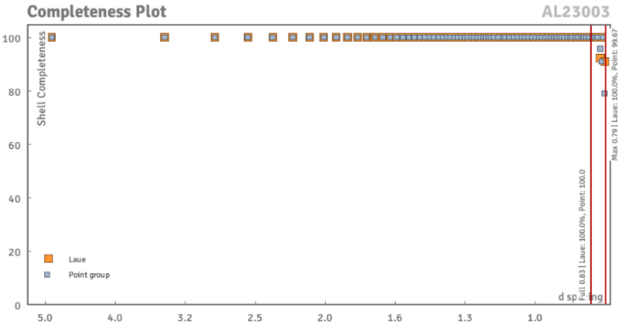

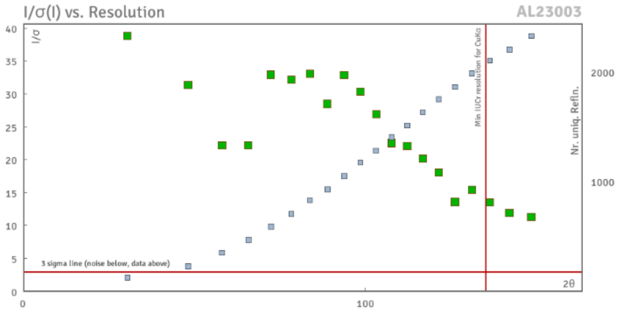

Data Plots: Refinement and Data

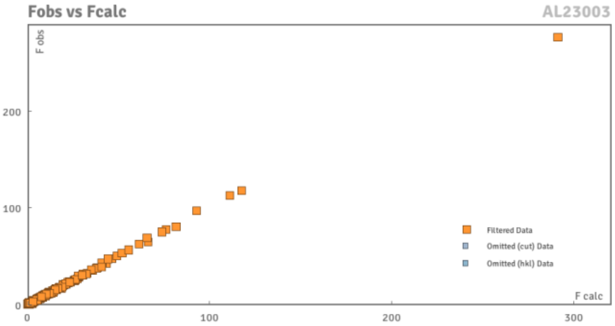

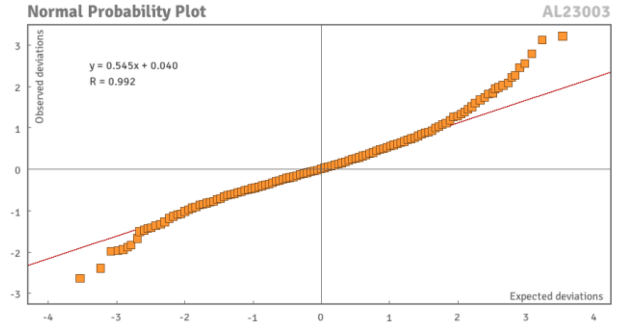

Reflection Statistics

Total reflections (after filtering)	19970	Unique reflections	2439
Completeness	0.997	Mean I/σ	24.11
hkl_{max} collected	(7, 12, 21)	hkl_{min} collected	(-9, -12, -21)
hkl_{max} used	(9, 12, 21)	hkl_{min} used	(-9, 0, 0)
Lim d_{max} collected	100.0	Lim d_{min} collected	0.77
d_{max} used	9.64	d_{min} used	0.79
Friedel pairs	2313	Friedel pairs merged	0
Inconsistent equivalents	0	R_{int}	0.0403
R_{sigma}	0.0267	Intensity transformed	0
Omitted reflections	0	Omitted by user (OMIT hkl)	0
Multiplicity	(2601, 1540, 1205, 755, 411, 217, 123, 91, 61, 37, 24, 24, 21, 6, 15, 11, 16, 4, 6, 0, 1)	Maximum multiplicity	36
Removed systematic absences	64	Filtered off (Shel/OMIT)	0

Table 50: Fractional Atomic Coordinates (×10^4) and Equivalent Isotropic Displacement Parameters (Å^2×10^3) for **AL23003**. U_{eq} is defined as 1/3 of the trace of the orthogonalised U_{ij}.

Atom	x	y	z	U_{eq}
F1	6283(2)	-573(2)	6595.1(15)	34.7(5)
F1A	8664(8)	-559(6)	6606(4)	36.4(15)

Atom	x	y	z	U_{eq}
F2	8583(3)	536(3)	5938.8(13)	35.8(5)
F2A	6338(8)	528(8)	5934(4)	36.2(15)
F3	6256(2)	1783(2)	6483.5(15)	35.7(5)
F3A	8650(8)	1799(7)	6473(4)	35.5(15)
F4	8358(3)	730(2)	7282.6(12)	35.0(5)
F4A	6593(9)	727(7)	7283(3)	35.7(16)
B1	7411(3)	616(2)	6577.1(12)	23.4(4)
O1	7420(3)	6878.5(14)	4491.9(9)	37.2(4)
O2	7371(3)	1559.5(14)	3552.8(8)	31.6(4)
N1	7423(3)	3848.8(15)	3320.7(9)	21.0(3)
N2	7454(3)	4560.2(16)	4876.6(9)	26.3(4)
C1	7453(4)	3622(2)	2439.1(11)	26.7(4)
C2	7934(12)	5064(4)	2126(3)	31.5(12)
C3	7395(4)	6078(2)	2785.1(12)	32.1(5)
C4	7421(3)	5187.3(18)	3509.0(12)	23.4(4)
C5	7432(4)	5517.4(19)	4322.1(11)	25.8(4)
C6	7436(3)	3214.9(19)	4661.7(11)	25.4(4)
C7	7407(3)	2822.2(19)	3867.5(11)	23.8(4)
C8	7435(5)	7228(2)	5328.2(13)	43.5(6)
C9	7395(5)	410(2)	4102.4(13)	37.6(5)
C2A	7010(40)	5056(13)	2132(8)	28(4)

Table 51: Anisotropic Displacement Parameters (×10^4) for **AL23003.** The anisotropic displacement factor exponent takes the form: $-2\pi^2[h^2a^{*2} \times U_{11}+ ... +2hka^* \times b^* \times U_{12}]$

Atom	U_{11}	U_{22}	U_{33}	U_{23}	U_{13}	U_{12}
F1	32.2(9)	26.9(9)	45.1(12)	-0.5(11)	2.1(9)	-6.5(8)
F1A	44(3)	24(3)	41(4)	-4(3)	-3(3)	9(3)
F2	29.1(9)	48.1(13)	30.3(10)	-0.8(11)	5.8(8)	-1.6(10)
F2A	37(3)	51(4)	20(3)	-2(3)	-8(2)	-1(3)
F3	29.3(9)	25.0(9)	52.7(14)	0.8(10)	-4.7(9)	5.3(7)
F3A	36(3)	33(3)	38(4)	2(3)	3(3)	-7(3)
F4	40.5(11)	39.0(12)	25.6(10)	-2.4(9)	-8.2(8)	0.9(9)
F4A	56(4)	33(3)	18(3)	-1(3)	6(3)	-2(3)
B1	24.6(10)	22.4(9)	23.1(9)	-2.1(8)	0.5(11)	-0.7(11)
O1	64.9(11)	18.4(6)	28.2(7)	-3.0(5)	1.9(10)	-0.6(9)
O2	52.6(9)	17.8(6)	24.4(6)	0.5(5)	0.5(9)	0.2(8)
N1	21.6(7)	18.7(7)	22.6(8)	-0.3(5)	0.4(9)	-0.1(8)
N2	32.7(8)	23.2(7)	22.9(7)	-0.7(6)	-0.3(9)	-1.3(11)
C1	31.4(10)	28.2(9)	20.5(8)	-1.4(7)	-0.8(11)	0.9(10)
C2	40(3)	28.1(15)	25.8(18)	3.2(13)	0(2)	-1.9(17)
C3	44.8(12)	23.8(9)	27.8(9)	5.8(7)	2.0(13)	1.1(11)
C4	24.8(8)	19.1(8)	26.4(9)	1.4(7)	0.7(11)	-0.7(9)
C5	31.1(10)	18.3(8)	28.1(9)	-1.4(7)	0.3(10)	-0.1(11)
C6	29.2(10)	22.5(8)	24.3(9)	1.7(7)	0.5(11)	-0.2(10)
C7	27.0(9)	19.8(8)	24.7(8)	1.7(7)	0.6(10)	0.7(10)
C8	77.7(17)	23.1(9)	29.8(10)	-6.6(8)	1.4(18)	-0.2(16)
C9	62.7(15)	17.9(8)	32.3(10)	4.3(8)	1.6(15)	1.5(14)
C2A	31(10)	42(6)	9(5)	3(4)	0(7)	0(6)

Table 52: Bond Lengths in Å for **AL23003**.

Atom	Atom	Length/Å	Atom	Atom	Length/Å
F1	B1	1.410(3)	F2A	B1	1.335(6)
F1A	B1	1.455(6)	F3	B1	1.412(3)
F2	B1	1.372(3)	F3A	B1	1.464(6)

Atom	Atom	Length/Å
F4	B1	1.375(3)
F4A	B1	1.332(6)
O1	C5	1.343(2)
O1	C8	1.445(2)
O2	C7	1.327(2)
O2	C9	1.442(2)
N1	C1	1.497(2)
N1	C4	1.328(2)
N1	C7	1.350(2)

Atom	Atom	Length/Å
N2	C5	1.311(3)
N2	C6	1.346(2)
C1	C2	1.527(4)
C1	C2A	1.509(13)
C2	C3	1.528(5)
C3	C4	1.489(3)
C3	C2A	1.500(13)
C4	C5	1.403(3)
C6	C7	1.387(3)

Table 53: Bond Angles in ° for **AL23003**.

Atom	Atom	Atom	Angle/°
F1	B1	F3	107.69(19)
F1A	B1	F3A	103.0(4)
F2	B1	F1	109.42(19)
F2	B1	F3	109.1(2)
F2	B1	F4	111.5(2)
F2A	B1	F1A	110.1(4)
F2A	B1	F3A	108.2(4)
F4	B1	F1	109.71(19)
F4	B1	F3	109.30(19)
F4A	B1	F1A	108.2(4)
F4A	B1	F2A	117.7(4)
F4A	B1	F3A	108.6(4)
C5	O1	C8	115.74(16)
C7	O2	C9	116.70(15)
C4	N1	C1	112.18(15)
C4	N1	C7	123.35(17)
C7	N1	C1	124.47(15)

Atom	Atom	Atom	Angle/°
C5	N2	C6	119.15(17)
N1	C1	C2	102.2(2)
N1	C1	C2A	101.6(5)
C1	C2	C3	105.9(3)
C4	C3	C2	102.7(2)
C4	C3	C2A	102.8(5)
N1	C4	C3	111.44(17)
N1	C4	C5	116.89(17)
C5	C4	C3	131.67(17)
O1	C5	C4	115.38(17)
N2	C5	O1	122.46(17)
N2	C5	C4	122.16(17)
N2	C6	C7	121.41(17)
O2	C7	N1	113.66(16)
O2	C7	C6	129.32(17)
N1	C7	C6	117.02(16)
C3	C2A	C1	108.2(8)

Table 54: Torsion Angles in ° for **AL23003**.

Atom	Atom	Atom	Atom	Angle/°
N1	C1	C2	C3	-22.0(5)
N1	C1	C2A	C3	19.1(17)
N1	C4	C5	O1	-179.7(2)
N1	C4	C5	N2	0.4(4)
N2	C6	C7	O2	-179.4(3)
N2	C6	C7	N1	0.9(4)
C1	N1	C4	C3	1.7(3)
C1	N1	C4	C5	-178.7(2)
C1	N1	C7	O2	-1.7(4)
C1	N1	C7	C6	178.1(2)
C1	C2	C3	C4	23.0(5)
C2	C3	C4	N1	-15.7(4)
C2	C3	C4	C5	164.8(4)
C3	C4	C5	O1	-0.2(4)
C3	C4	C5	N2	179.9(3)
C4	N1	C1	C2	13.1(4)
C4	N1	C1	C2A	-12.9(11)
C4	N1	C7	O2	178.6(2)
C4	N1	C7	C6	-1.6(4)
C4	C3	C2A	C1	-18.4(17)
C5	N2	C6	C7	0.4(4)
C6	N2	C5	O1	179.0(3)

Atom	Atom	Atom	Atom	Angle/˚
C6	N2	C5	C4	-1.1(4)
C7	N1	C1	C2	-166.7(4)
C7	N1	C1	C2A	167.3(11)
C7	N1	C4	C3	-178.6(2)
C7	N1	C4	C5	1.0(4)
C8	O1	C5	N2	0.1(4)
C8	O1	C5	C4	-179.8(3)
C9	O2	C7	N1	178.6(2)
C9	O2	C7	C6	-1.1(4)
C2A	C3	C4	N1	10.5(11)
C2A	C3	C4	C5	-169.0(11)

Table 55: Hydrogen Fractional Atomic Coordinates (×10^4) and Equivalent Isotropic Displacement Parameters (Å^2×10^3) for **AL23003.** U_{eq} is defined as 1/3 of the trace of the orthogonalised U_{ij}.

Atom	x	y	z	U_{eq}
H1AA	6239.24	3311.04	2241.02	32
H1AB	8398.34	2931.72	2286.95	32
H1BC	6511.1	2939.33	2273.08	32
H1BD	8677.47	3305.88	2255.83	32
H2A	7241.42	5266.17	1632.01	38
H2B	9266.08	5131.68	2010.41	38
H3AA	8293.65	6845.43	2828.64	39
H3AB	6155.38	6469.54	2691.12	39
H3BC	8593.05	6544.88	2704.96	39
H3BD	6413.76	6787.41	2818.16	39
H6	7444.29	2519.68	5062.41	30
H8A	7422.05	8238.92	5389.21	65
H8B	8547.36	6850.02	5576.28	65
H8C	6347.12	6831.71	5586.46	65
H9A	7360.24	-463.92	3804.77	56
H9B	6320.29	465.56	4453.04	56
H9C	8519.56	447.36	4422.9	56
H2AA	7783.82	5268.6	1662.01	33
H2AB	5705.98	5107.78	1972.07	33

Table 56: Atomic Occupancies for all atoms that are not fully occupied in **AL23003**.

Atom	Occupancy	Atom	Occupancy
F1	0.752(3)	H3BC	0.21(2)
F1A	0.248(3)	H3BD	0.21(2)
F2	0.752(3)	C2A	0.21(2)
F2A	0.248(3)	H2AA	0.21(2)
F3	0.752(3)	H2AB	0.21(2)
F3A	0.248(3)		
F4	0.752(3)		
F4A	0.248(3)		
H1AA	0.79(2)		
H1AB	0.79(2)		
H1BC	0.21(2)		
H1BD	0.21(2)		
C2	0.79(2)		
H2A	0.79(2)		
H2B	0.79(2)		
H3AA	0.79(2)		
H3AB	0.79(2)		

Citations

CrysAlisPro (Rigaku, V1.171.42.81a, 2023)

L.J. Bourhis and O.V. Dolomanov and R.J. Gildea and J.A.K. Howard and H. Puschmann, The Anatomy of a Comprehensive Constrained, Restrained, Refinement Program for the Modern Computing Environment - Olex2 Disected, *Acta Cryst. A*, (2015), **A71**, 59-71.

O.V. Dolomanov and L.J. Bourhis and R.J. Gildea and J.A.K. Howard and H. Puschmann, Olex2: A complete structure solution, refinement and analysis program, *J. Appl. Cryst.*, (2009), **42**, 339-341.

SADABS, Bruker axs, Madison, WI (?).

Sheldrick, G.M., Crystal structure refinement with ShelXL, *Acta Cryst.*, (2015), **C71**, 3-8.

```
#==============================================================================
# PLATON/CHECK-(181221) versus check.def version 211218, Entry: al23003
# Data: AL23003.cif - Type: CIF                  Bond Precision   C-C = 0.0030 A
# Refl: AL23003.fcf - Type: LIST4                                  Temp = 120 K
# Audit:OLEX2 1.5-BETA (COMPILED 2023.03.06 SVN.RBB2C1857 FOR OLEXSYS, GUI SVN.R
# Refin:SHELXL 2018/3 (SHELDRICK, 2015)
# X-ray CuKa             R(int) = 0.040,  wR2/R(int) =  2.4,  Nref/Npar =   6.7
# Cell   7.2726(2)   9.6396(2)  16.8013(4)          90          90          90
# Wavelength 1.54178  Volume Reported      1177.85(5) Calculated      1177.85(5)
# SpaceGroup from Symmetry P 21 21 21   Hall: P 2ac 2ab             orthorhombic
#                 Reported P 21 21 21         P 2ac 2ab             orthorhombic
# MoietyFormula C9 H13 N2 O2, B F4
#      Reported B F4, C9 H13 N2 O2
#    SumFormula C9 H13 B F4 N2 O2
#      Reported C9 H13 B F4 N2 O2
# Mr        =    268.02[Calc],    268.02[Rep]       Volume/NonHatoms = 16 Ang**3
# Dx,gcm-3  =     1.512[Calc],     1.511[Rep]
# Z         =         4[Calc],         4[Rep]
# Mu (mm-1) =     1.292[Calc],     1.292[Rep]  Xtal Size =  0.038x0.046x0.507 mm
# F000      =     552.0[Calc],     552.0[Rep]  or F000' =    554.40[Calc]
# Reported   T Limits: Tmin=0.653              Tmax=0.754  AbsCorr = MULTI-SCAN
# Calculated T Limits: Tmin=0.931 Tmin'=0.519  Tmax=0.952
# Measured   HKL: Reported  19906, Embedded  19970, <Mult> 13.9
# Reported   Hmax=  9, Kmax= 12, Lmax= 21, Nref=   2439        , Th(max)= 75.999
# Obs in FCF Hmax=  9, Kmax= 12, Lmax= 21, Nref=   2439[  1430], Th(max)= 75.999
# Calculated Hmax=  9, Kmax= 12, Lmax= 21, Nref=   2447[  1433], Ratio=1.70/1.00
# Reported   Rho(min) = -0.20, Rho(max) =  0.28 e/Ang**3 (From CIF)
# Calculated Rho(min) = -0.19, Rho(max) =  0.27 e/Ang**3 (From CIF+FCF data)
# w=1/[<sup>2</sup>(Fo<sup>2</sup>)+(0.0498P)<sup>2</sup>+0.2552P],
P=(Fo<sup>2</sup>+2Fc<sup>2</sup>)/3
# R= 0.0358(  2279), wR2= 0.0949(  2439), S = 1.060      (From CIF+FCF data)
# R= 0.0358(  2279), wR2= 0.0949(  2439), S = 1.061      (From FCF data only)
# R= 0.0358(  2279), wR2= 0.0949(  2439), S = 1.061, Npar=  212, Flack   0.42(7)
# Number Bijvoet Pairs = 1009 (100%),       897 Selected for:  Parsons   0.41(7)
#              P3(tr) 0.000, P3(tw) 1.000, Student-T Nu = 100,  Hooft   0.43(7)
#==============================================================================
# For Documentation:http://www.platonsoft.nl/CIF-VALIDATION.pdf
#==============================================================================
*
#==============================================================================
#>>> The Following Improvement and Query ALERTS were generated - (Acta-Mode) <<<
#==============================================================================
Format: alert-number_ALERT_alert-type_alert-level text

089_ALERT_3_C Poor Data / Parameter Ratio (Zmax < 18) ........       6.75 Note
#==============================================================================
002_ALERT_2_G Number of Distance or Angle Restraints on AtSite         12 Note
033_ALERT_4_G Flack x Value Deviates > 3.0 * sigma from Zero .      0.420 Note
042_ALERT_1_G Calc. and Reported Moiety Formula Strings Differ     Please Check
176_ALERT_4_G The CIF-Embedded .res File Contains SADI Records          3 Report
187_ALERT_4_G The CIF-Embedded .res File Contains RIGU Records          2 Report
301_ALERT_3_G Main Residue  Disorder .............(Resd  1  )          8% Note
302_ALERT_4_G Anion/Solvent/Minor-Residue Disorder (Resd  2  )        80% Note
432_ALERT_2_G Short Inter X...Y Contact  F1       ..C4               2.84 Ang.
-1/2+x,1/2-y,1-z  =      4_456 Check
432_ALERT_2_G Short Inter X...Y Contact  F3       ..C7               2.89 Ang.
-1/2+x,1/2-y,1-z  =      4_456 Check
432_ALERT_2_G Short Inter X...Y Contact  F1A      ..C4               2.76 Ang.
1/2+x,1/2-y,1-z  =      4_556 Check
432_ALERT_2_G Short Inter X...Y Contact  F1A      ..C3               2.94 Ang.
1/2+x,1/2-y,1-z  =      4_556 Check
432_ALERT_2_G Short Inter X...Y Contact  F3A      ..C7               2.82 Ang.
1/2+x,1/2-y,1-z  =      4_556 Check
720_ALERT_4_G Number of Unusual/Non-Standard Labels ..........         10 Note
802_ALERT_4_G CIF Input Record(s) with more than 80 Characters          1 Info
811_ALERT_5_G No ADDSYM Analysis: Too Many Excluded Atoms ....          ! Info
860_ALERT_3_G Number of Least-Squares Restraints ............          83 Note
912_ALERT_4_G Missing # of FCF Reflections Above STh/L=  0.600          3 Note
978_ALERT_2_G Number C-C Bonds with Positive Residual Density.          3 Info
```

```
#==============================================================================

ALERT_Level and ALERT_Type Summary
==================================
1 ALERT_Level_C = Check. Ensure it is Not caused by an Omission or Oversight
18 ALERT_Level_G = General Info/Check that it is not Something Unexpected

1 ALERT_Type_1 CIF Construction/Syntax Error, Inconsistent or Missing Data.
7 ALERT_Type_2 Indicator that the Structure Model may be Wrong or Deficient.
3 ALERT_Type_3 Indicator that the Structure Quality may be Low.
7 ALERT_Type_4 Improvement, Methodology, Query or Suggestion.
1 ALERT_Type_5 Informative Message, Check.
#==============================================================================

0 Missing  Experimental  Info Issue(s) (Out of  64 Tests) -  100 % Satisfied
0 Experimental  Data  Related Issue(s) (Out of  35 Tests) -  100 % Satisfied
6 Structural  Model   Related Issue(s) (Out of 136 Tests) -   96 % Satisfied
12 Unresolved or to be Checked Issue(s) (Out of 272 Tests) -   96 % Satisfied

*
```

5. References

1. Liu, J.; Parker, M. F. L.; Wang, S.; Flavell, R. R.; Toste, F. D.; Wilson, D. M. Synthesis of *N*-trifluoromethyl amides from carboxylic acids. *Chem* **2021**, *7*, 2245–2255.
2. Huy, P.; Neudörfl, J-M.; Schmalz, H-G. A practical synthesis of trans-3-substituted proline derivatives through 1, 4-addition. *Org. Lett.* **2011**, *13*, 216–219.
3. Fan, X.; and Smith, M. W. A general approach to 2, 2-disubstituted indoxyls: total synthesis of brevianamide A and trigonoliimine C. *Chem. Sci.* **2021**, *12*, 13756–13763.
4. Johnson, N. T.; Waddell, P. G.; Clegg, W.; Probert, M. R. Remote Access Revolution: Chemical Crystallographers Enter a New Era at Diamond Light Source Beamline I19, *Crystals*, **2017**, *7*, 360–368.
5. Godfrey, R. C.; Green, N. J.; Nichol, G. S.; Lawrence, A. L. Total synthesis of brevianamide A. *Nat. Chem.* **2020**, *12*, 615–619.
6. Medvecký, M.; Linder, I.; Schefzig, L.; Reissig, H-U.; Zimmer, R. Iodination of carbohydrate-derived 1, 2-oxazines to enantiopure 5-iodo-3, 6-dihydro-2H-1, 2-oxazines and subsequent palladium-catalyzed cross-coupling reactions. *Beilstein J. Org. Chem.* **2016**, *12*, 2898–2905.
7. Greshock, T. J.; Grubbs, A. W.; Jiao, P.; Wicklow, D. T.; Gloer, J. B.; Williams, R. M. Isolation, Structure Elucidation, and Biomimetic Total Synthesis of Versicolamide B, and the Isolation of Antipodal (−)-Stephacidin A and (+)-Notoamide B from Aspergillus versicolor NRRL 35600. *Angew. Chem.* **2008**, *120*, 3629–3633.
8. Koldobskii, A. B.; Solodova, E. V.; Verteletskii, P. V.; Godovikov, I. A.; Kalinin, V. N. The General Approach for the Synthesis of Substituted Cyclobutenyl- and Norbornadienyllithiums Containing Masked Trifluoromethyl Group. *Tetrahedron*. **2010**, *66*, 9589–9595.
9. Lichtscheidl, A. G.; Janicke, M. T.; Scott, B. L.; Nelson, A. T.; Kiplinger, J. L. Synthesis, structures, and ^{1}H, $^{13}C\{^{1}H\}$ and $^{119}Sn\{^{1}H\}$ NMR chemical shifts of a family of trimethyltin alkoxide, amide, halide and cyclopentadienyl compounds. *Dalton Transactions*. **2015**, *44*, 16156–16163.
10. Paterson, I.; Paquet, T; Dalby, S. M. Synthesis of the macrocyclic core of leiodermatolide. *Org. Lett*. **2011**, *13*, 4398–4401.
11. Wagger, J.; Grošelj, U.; Svete, J.; Stanovnik, B. Synthesis of Racemic, *N*-Benzylated Neoechinulin A and Isoneoechinulin A, *synlett*. **2010**, *8*, 1197–1200.
12. Chengebroyen, J.; Linke, M.; Robitzer, M.; Sirlin, C; Pfeffer, M. Palladium-mediated intramolecular C-N bond formation involving allyl substituted pyridines. Application to a novel strategy for the synthesis of the skeleton of berberinium derivatives, *J. Organomet. Chem*. **2003**, *687*, 313–321.

13. Kuttruff, C. A.; Zipse, H.; Trauner, D. Concise Total Synthesis of Variecolortides A and B through an Unusual Hetero-Diels–Alder Reaction. *Angew. Chem. Int. Ed.* **2011**, *6*, 1402–1405.

14. Cledera, P.; Avendaño, C.; Menéndez, J. C. Comparative Study of Synthetic Approaches to 1-arylmethylenepyrazino[2,1-*b*]quinazoline-3,6-diones, *Tetrahedron*, **1998**, *54*, 12349–12360.

15. Song, F.; Liu, X.; Guo, H.; Ren, B.; Chen, C.; Piggott, A. M.; Yu, K.; Gao, H.; Wang, Q.; Liu, M.; Liu, X.; Dai, H.; Zhang, L.; Capon, R. J. Brevianamides with antitubercular potential from a marine-derived isolate of Aspergillus versicolor. *Org. Lett*. **2012**, *14*, 4770–4773.

16. Sezen, B.; Dalibor Sames. Oxidative C-Arylation of Free (NH)-Heterocycles via Direct (sp3) C− H Bond Functionalization. *J. Am. Chem. Soc.* **2004**, *126*, 13244–13246.

17. Häusler, J. Darstellung von cis-und trans-C-3-substituierten Prolinverbindungen. *Liebigs Annalen der Chemie* **1981**, *6*, 1073–1088.

18. Jorge, B.; Hamilton, A. D. Helix mimetics as inhibitors of the interaction of the estrogen receptor with coactivator peptides. *Angew. Chem. Int. Ed.* **2007**, *119*, 4471–4473.

19. Seger, H.; Geyer, A. Synthesis and Diversification of Pyridone Dipeptide Chromophores. *Synthesis.* **2006**, *19*, 3224–3230.
